# Identification of Potential Biomarkers for Progression and Prognosis of Bladder Cancer by Comprehensive Bioinformatics Analysis

**DOI:** 10.1155/2022/1802706

**Published:** 2022-04-19

**Authors:** Zhiyong Tan, Shi Fu, Runlin Feng, Yinglong Huang, Ning Li, Haifeng Wang, Jiansong Wang

**Affiliations:** ^1^Department of Urology, The Second Affiliated Hospital of Kunming Medical University, No. 347, Dianmian Street, Wuhua District, Kunming, 650101 Yunnan, China; ^2^Urological Disease Clinical Medical Center of Yunnan Province, The Second Affiliated Hospital of Kunming Medical University, No. 347, Dianmian Street, Wuhua District, Kunming, 650101 Yunnan, China; ^3^Scientific and Technological Innovation Team of Basic and Clinical Research of Bladder Cancer in Yunnan Universities, The Second Affiliated Hospital of Kunming Medical University, No. 347, Dianmian Street, Wuhua District, Kunming, 650101 Yunnan, China; ^4^Department of Pathology, The Second Affiliated Hospital of Kunming Medical University, No. 347, Dianmian Street, Wuhua District, Kunming, 650101 Yunnan, China

## Abstract

*Background*. Bladder cancer (BLCA) is a highly malignant tumor that develops in the urinary system. Identification of biomarkers in progression and prognosis is crucial for the treatment of BLCA. BLCA-related differentially expressed genes (DEGs) were authenticated by screening the DEGs and weighted gene coexpression network analysis (WGCNA). LASSO and SVM-RFE algorithms were utilized to screen the feature genes in BLCA. Survival analysis was performed using the Kaplan–Meier curve provided by the ‘survival' R package. The BLCA samples were clustered by hclust based on the immune score matrix calculated by the single-sample GSEA (ssGSEA) algorithm. The immune, stromal, and ESTIMATE scores of each BLCA patient were calculated by applying the ESTIMATE algorithm. ssGSEA was conducted to explore the function of characteristic genes in BLCA. The expression of characteristic genes in clinical cancer tissue, and the pericancerous tissue of BLCA patients was verified using qRT-PCR assays. A total of 189 BLCA-related DEGs were identified. Fourteen feature genes were defined by LASSO and SVM-RFE algorithms. Five characteristic genes, including SMYD2, GAPDHP1, ATP1A2, CILP, and THSD4, were related to the OS of BLCA. The correlation analysis of five characteristic genes and clinicopathological factors showed that five genes played a role in the progression of BLCA. Additionally, the expression of five characteristic genes in clinical cancer tissues and pericarcinomatous tissues from BLCA patients was verified by qRT-PCR, which was consistent with the result from the public database. Finally, we discovered five prognostic genes linked to BLCA progression, which might serve as a theoretical basis for prognosis and treatment targets for BLCA patients.

## 1. Introduction

Bladder cancer (BLCA) is the most common malignant carcinoma of the genitourinary system, with a high rate of recurrence [1]. BLCA is the world's fifth most prevalent malignant malignancy, with an estimated 81,180 new cases and 17,100 deaths in the United States in 2022, posing a serious threat to people's lives and health [2]. It also imposes a great financial burden on patients with this disease. The pathophysiology of BLCA is widely recognized to be complicated, as it is caused by a combination of intrinsic genetic factors and extrinsic environmental factors [3]. Urothelial carcinoma (also known as transitional cell carcinoma) is the most common histological subtype of BLCA, accounting for more than 90% of all BLCA [4]. BLCA is divided into two types based on the degree of muscle invasion: nonmuscle invasive bladder cancer (NMIBC) and muscle-invasive bladder cancer (MIBC) [5]. In the treatment of MIBC, recurrence is still a major issue. Approximately 75% of patients present with NMIBC initially. Although these patients often get rigorous therapies such as surgery, immunotherapy, chemotherapy, and radiotherapy, their responses are variable and unpredictable. About 10-30% of NMIBC individuals may relapse and progress to MIBC, and the 5-year overall survival (OS) rate remains unsatisfactory, with a median OS of about 14 months [6]. Since there are no clinical biomarkers or parameters that can consistently represent disease development, the prognosis of BLCA patients is difficult to predict. Individual differences also have a significant impact in determining the efficacy of BLCA treatment. Clarifying the potential molecular pathways involved in BLCA carcinogenesis, proliferation, and recurrence, as well as identifying novel potential biomarkers, is critical for early diagnosis, prognostic evaluation, and treatment of BLCA.

Bioinformatics has been widely utilized to identify and evaluate genes linked with the development and progression of cancers as genome sequencing technology progresses [7]. Most previous research has focused on identifying a single gene or protein, rather than describing the relationship between genes and interaction pathways. Throughout the network, several genes with similar expression patterns interact with others. Weighted gene coexpression network analysis (WGCNA) is a systematical biology method for describing gene connection patterns across models. Based on the endogeneity of the gene set and the link between the gene set and the phenotype, this method may be used to find highly synergistic gene sets and candidate biomarker genes or therapeutic targets [8]. WGCNA has been frequently used to explore genes linked to cancer phenotypes such as breast cancer, colon cancer, and castration-resistant prostate cancer [9–11]. We can use WGCNA to build coexpression networks and perform gene-specific analysis, as well as find differently linked gene clusters. Several potential molecular indicators linked to the development of BLCA have been found in previous studies. Droop et al. used RT-qPCR to detect lncRNA UCA1 expression levels in cell lines and tissues, which showed that OS was much better in patients with high lncRNA UCA1 expression levels compared to those with low expression levels [12]. Overexpression of UCA1 frequently exhibited a low Ki-67 proliferation index and a p53 “wild-type” immune profile [13]. 5-Hydroxymethylcytosine (5hmC) deletion has been recognized as a characteristic of most cancers. Peng et al. found that lower 5hmC levels are associated not only with the poor OS but also with higher tumor stage and lymphatic metastasis [14]. In addition, Zhang et al. found GTPase RAN binding protein 1 (RanBP1) is an early diagnostic biomarker for BLCA and a candidate pharmacological target for treatment [15]. Although there are many studies on the prognostic biomarkers of BLCA, the mechanisms of poor diagnosis and prognosis of BLCA still need further exploration.

The Cancer Genome Atlas (TCGA) and Gene Expression Omnibus (GEO) datasets were used in this study to identify possible biomarkers and biological functions linked with BLCA using the comprehensive bioinformatics method. Our findings might provide a theoretical foundation for further research into clinical prognosis and treatment targets for BLCA.

## 2. Materials and Methods

### 2.1. Data Source

This study included two BLCA datasets, TCGA-BLCA and GSE133624. The Cancer Genome Atlas (TCGA) database (https://portal.gdc.cancer.gov/) provided the TCGA-BLCA dataset, which had 19 normal and 408 BLCA samples. For the prognostic analysis, 403 patients with survival data were selected. The Gene Expression Omnibus (GEO) database (https://www.ncbi.nlm.nih.gov/geo/) contained the GSE133624 dataset, which included 29 normal and 36 BLCA samples.

### 2.2. Sample Sources for Quantitative Real-Time PCR Validation

We took 10 cancer tissues and 10 pericarcinomatous tissues from BLCA patients who had radical cystectomy at The Second Affiliated Hospital of Kunming Medical University. All of the patients had uroepithelial carcinoma, which was identified pathologically. Pericarcinomatous tissues were classified as those removed from the lesion at a distance of 2.5 cm. We gave individual numbers to the samples after collecting them and promptly stored them in liquid nitrogen chambers for preservation. All participants gave their informed consent, and the study was authorized by the Ethics Committee at The Second Affiliated Hospital of Kunming Medical University.

### 2.3. Identification of Differentially Expressed Genes (DEGs)

The ‘limma' R package and the ‘DESeq2' R package were used to identify DEGs between normal and BLCA samples in the TCGA-BLCA and GSE133624 datasets, respectively. The cut-off criteria were |log2 fold change (FC)| > 1 and *P* value < 0.05. The results were drawn into a volcano plot, and the Top 100 DEGs were drawn into a heatmap.

### 2.4. WGCNA Identifies BLCA-Related DEGs

The WGCNA coexpression system was established using the ‘WGCNA' R package [16] with DEG expression data from the TCGA-BLCA and GSE81558 datasets, with normal control and BLCA disease as clinical features. The ‘goodSamplesGenes' function was used to perform sample clustering to identify and remove outliers. A soft-thresholding power was computed using the pickSoftThreshold function and validated by the correlation between *k* and *p* (*k*) to make the coexpression network fulfilled the distribution of a scale-free network. The dynamic tree cutting approach was utilized to identify various modules, with each module containing a minimum of 30 genes. Following that, a 0.2 merging threshold was chosen to combine related modules. The relationship between these modules and two clinical characteristics was investigated further. Finally, for further investigation, the module with the greatest Pearson correlation coefficient was picked.

### 2.5. Screening Feature Genes by Machine Learning

We employed LASSO and SVM-RFE algorithms to screen the feature genes in BLCA by using the ‘glmnet' and ‘e1071' packages, respectively. The least absolute shrinkage and selection operator (LASSO) is a model refinement algorithm that creates a penalty function [17]. Support vector machine-recursive feature elimination (SVM-RFE) is a support vector machine-based feature selection technique that ranks features based on a recursive feature deletion sequence [18]. These two methods discovered overlapping genes, which were referred to as feature genes.

### 2.6. Survival Analysis

To explore the effect of feature genes on patient survival, BLCA patients were divided into two groups, the high- and low-expression groups, based on the optimal gene expression cut-off value. For survival analysis, the ‘survival' R package was used, and the Kaplan–Meier curve was constructed. A significant difference in survival between the high- and low-expression groups was defined as a *P* value ≤ 0.05.

### 2.7. The Correlation Analysis between Characteristic Genes and Clinicopathological Characteristics

The expression of characteristic genes was analyzed and compared across different subgroups of clinicopathological features to better understand the function of characteristic genes in the progression of BLCA.

### 2.8. The Correlation Analysis between Characteristic Genes and Immune Infiltration

A single-sample GSEA (ssGSEA) method was used to generate the immune score of each BLCA sample in the TCGA dataset, which was based on the genetic markers of 28 tumor-infiltrating immune cells. Based on the immune score matrix, the BLCA samples were grouped by hclust into high-immunity group (Immunity H), medium-immunity group (Immunity M), and low-immunity group (Immunity L). The ‘Estimation of Stromal and Immune cells in MAlignant Tumours using Expression data' (ESTIMATE) algorithm was used to compute the immunological, stromal, and ESTIMATE scores of each BLCA patient in the various immune groups. Different immunological groups had their expression of distinctive genes evaluated and compared. It was also evaluated at the Pearson association between characteristic genes and immune infiltration cells. |Correlation coefficient (cor)| > 0.3 and *P* value < 0.05 were considered to be significantly correlated.

### 2.9. Single-Gene Gene Set Enrichment Analysis

Gene set enrichment analysis (GSEA) for single gene based on ‘c5.go.v7.4.symbols.gmt' and ‘c2.cp.kegg.v7.4.symbols.gmt' gene sets was performed in GSEA software. The expression value of each gene was used as phenotype files, and the correlation coefficients of each gene with all genes in the gene sets were ranked. |NES| > 1, NOM *P* value < 0.05, and FDR *q* value < 0.25 were used as enrichment significance thresholds.

### 2.10. Quantitative Real-Time PCR (qRT-PCR) Assay

Total RNAs from the 20 samples were extracted using TRIzol reagent (Life Technology, CA, USA) according to the manufacturer's protocol to further investigate the functions of genes in BLCA. The PrimeScript RT Master Mix was used for reverse transcription (Takara, Tokyo, Japan). An ABI 7700 machine was used to perform quantitative real-time PCR (Applied Biosystems, CA, USA). As an internal control, the transcription level of GAPDH was employed. The 2^-*ΔΔ*Ct^ method was used to determine relative gene expression levels. Supplementary Table 1 lists the PCR primer sequences.

### 2.11. Statistical Analysis

All analyses were carried out using the R programming language, and the Wilcoxon and Kruskal-Wallis tests were used to compare data from different groups. If not specified above, statistical significance was defined as a *P* value < 0.05.

## 3. Results

### 3.1. BLCA-Related DEGs

**Figure 1:**
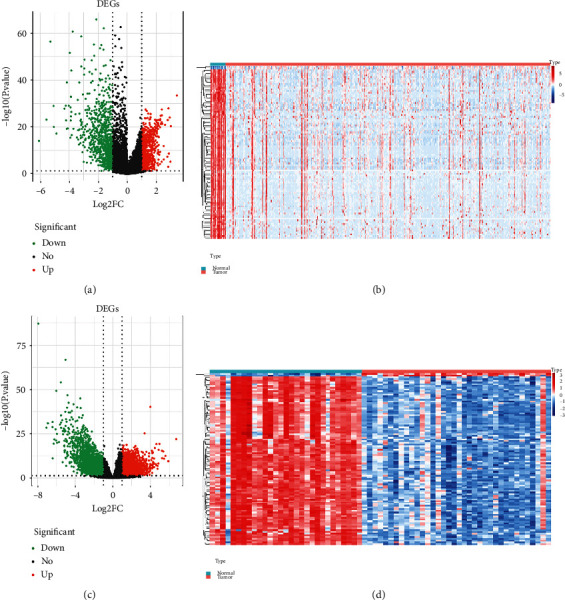
Differentially expressed genes (DEGs) are identified. (a) The volcano plot of DEGs in the TCGA-BLCA dataset. (b) The heatmap of TOP100 DEGs in the TCGA-BLCA dataset. (c) The volcano plot of DEGs in the GSE133624 dataset. (d) The heatmap of TOP100 DEGs in the GSE133624 dataset.

First, we used differential expression analysis to compare the gene expression profiles from the TCGA-BLCA and GSE133624 datasets. Based on the given criteria, a total of 1642 DEGs (including 763 upregulated and 879 downregulated genes) were identified from the TCGA-BLCA dataset between normal and BLCA samples (Figure 1(a), Supplementary Table 2). Meanwhile, in the GSE133624 dataset, a total of 5453 DEGs were identified, with 2200 upregulated genes and 3253 downregulated genes between normal and BLCA samples (Figure 1(c), Supplementary Table 3). A heatmap showed the expression of the top 100 DEGs (Figures 1(b) and 1(d)).

**Figure 2:**
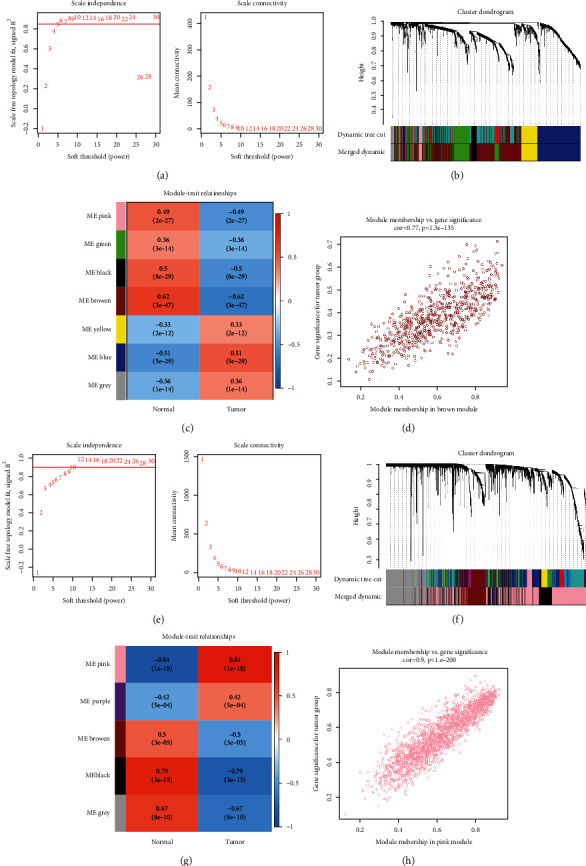
WGCNA was used to identify BLCA-related DEGs. (a) In the TCGA-BLCA dataset, analysis of the scale-free fit index (left) and mean connectivity (right) for various soft-thresholding powers. *β* = 6 was selected as the optimal soft-thresholding parameter. (b) In the TCGA-BLCA dataset, a dendrogram of all DEGs is clustered based on a dissimilarity score (different colors indicate different modules). (c) A heatmap showing the relationship between module eigengenes and BLCA clinical characteristics. (d) In the brown module, a scatter plot of module eigengenes is shown. (e) In the GSE133624 dataset, analysis of the scale-free fit index (left) and mean connectivity (right) for various soft-thresholding powers. *β* = 12 was selected as the optimal soft-thresholding parameter. (f) In the GSE133624 dataset, a dendrogram showing all DEGs grouped using a dissimilarity measure (different colors indicate distinct modules). (g) A heatmap showing the relationship between module eigengenes and BLCA clinical characteristics. (h) In the pink module, a scatter plot of module eigengenes is shown.

**Figure 3:**
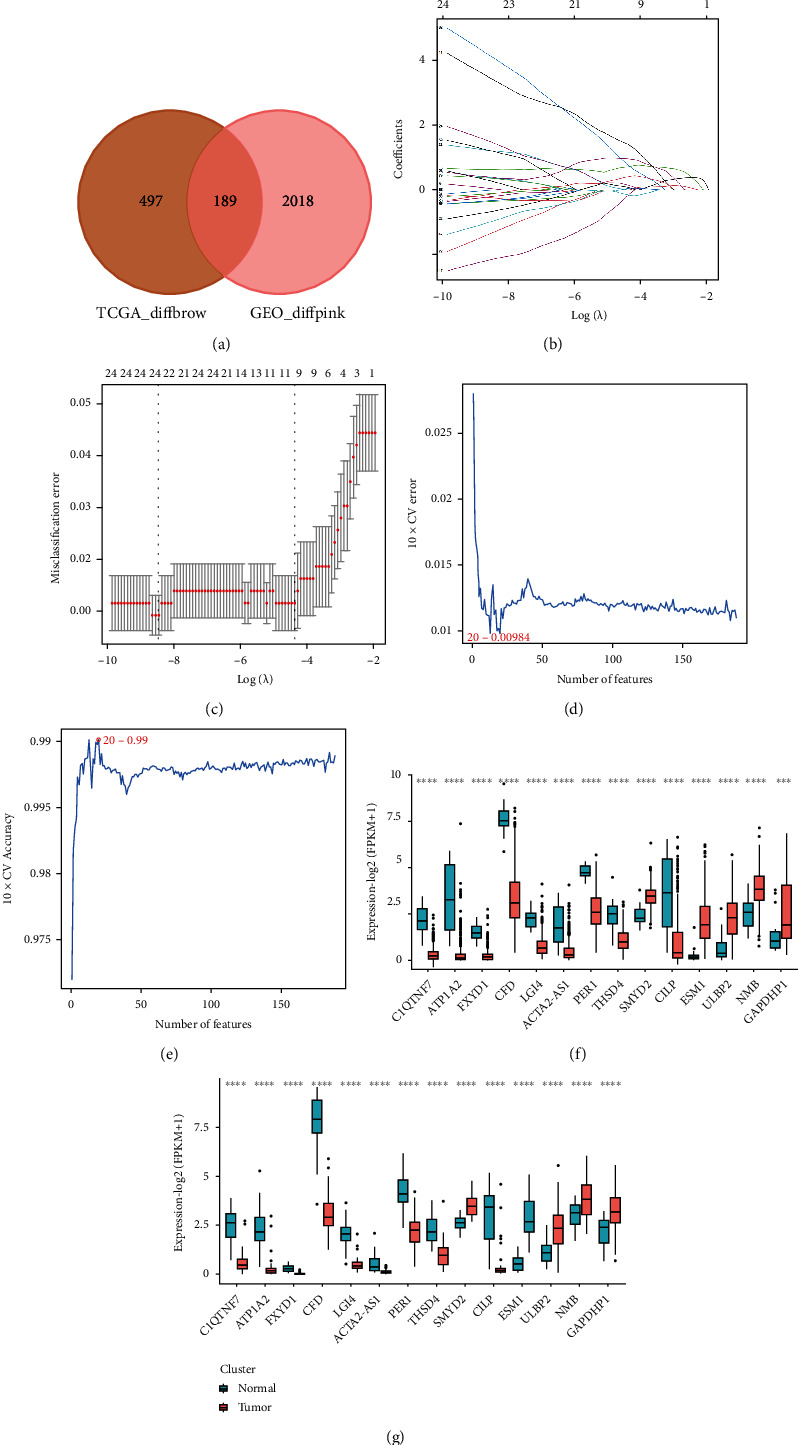
Screening feature genes by machine learning. (a) The Venn diagram of key module genes in the TCGA-BLCA and GSE133624 datasets. (b) Penalty graph of twenty-four characteristic variable coefficients. As the penalty coefficient lambda changes, the coefficients of most variables are compressed to zero. (c) In the LASSO logistic regression model, the best lambda value is selected when the 10-fold cross-validation error is minimal. (d, e) Feature genes were selected with the SVM-RFE algorithm at the optimal point 0.00984. (f) Candidate signature genes expression in the TCGA dataset. (g) Expression of candidate signature genes in the GSE133624 dataset.

Subsequently, we uncovered the BLCA-related DEGs by WGCNA. In the TCGA-BLCA dataset, no obvious outliers were removed by clustering (Fig. S1A) and *β* = 6 (scale-free *R*^2^ = 0.85) was selected to construct a scale-free network (Figure 2(a), Fig. S1B). Then, a cluster dendrogram was constructed and a dynamic tree cut was performed. Nine modules were eventually developed after merging (Figure 2(b), Fig. S1C). We then analyzed the correlation of each module with two clinical traits (normal and tumor). The results indicated that the brown module (|cor| = 0.62, *P* < 0.0001) had the highest correlation with BLCA (Figure 2(c)). Thus, we extracted 686 genes from the brown module as BLCA-related DEGs in the TCGA-BLCA dataset (Figure 2(d), supplementary Table 4). Meanwhile, in the GSE133624 dataset, no obvious outliers were removed by clustering (Fig. S2A) and *β* = 12 (scale-free *R*^2^ = 0.9) was selected to construct a scale-free network (Figure 2(e), Fig. S2B). Eventually, five modules were generated after merging (Figure 2(f), Fig. S2C). The correlation of each module with two clinical features (normal and tumor) was calculated, and the pink module had the strongest correlation with BLCA (|cor| = 0.84, *P* = 0.0001) (Figure 2(g)). In the GSE133624 dataset, the 2207 genes in the pink module were classified as BLCA-related DEGs (Figure 2(h), Supplementary Table 4). Finally, by overlapping the BLCA-related DEGs in the TCGA-BLCA dataset and the GSE133624 dataset, we were able to obtain 189 BLCA-related DEGs (Figure 3(a), Supplementary Table 4).

### 3.2. Screening Feature Genes by Machine Learning

To filter out the feature genes based on 159 BLCA-related DEGs, we adopted the LASSO regression and SVM-RFP algorithms. 24 feature genes were determined by LASSO when lambda was close to 0 (Figures 3(b) and 3(c), Supplementary Table 5). In the meantime, 20 feature genes were selected with the SVM-RFE algorithm at the optimal point 0.00984 (Figures 3(d) and 3(e), Supplementary Table 5). Fourteen feature genes were defined by overlapping the genes derived from these two algorithms, including C1QTNF7, ATP1A2, FXYD1, CFD, LGI4, ACTA2-AS1, PER1, THSD4, SMYD2, CILP, ESM1, ULBP2, NMB, and GAPDHP1. Then, we visualized the expression of feature genes in Figures 3(f) and 3(g). We noted that SMYD2, ESM1, ULBP2, NMB, and GAPDHP1 were upregulated in BLCA samples, and C1QTNF7, ATP1A2, FXYD1, CFD, LGI4, ACTA2-AS1, PER1, THSD4, and CILP were downregulated in BLCA samples both in the TCGA-BLCA and GSE133624 datasets.

### 3.3. Characteristic Genes Related to Survival

**Figure 4:**
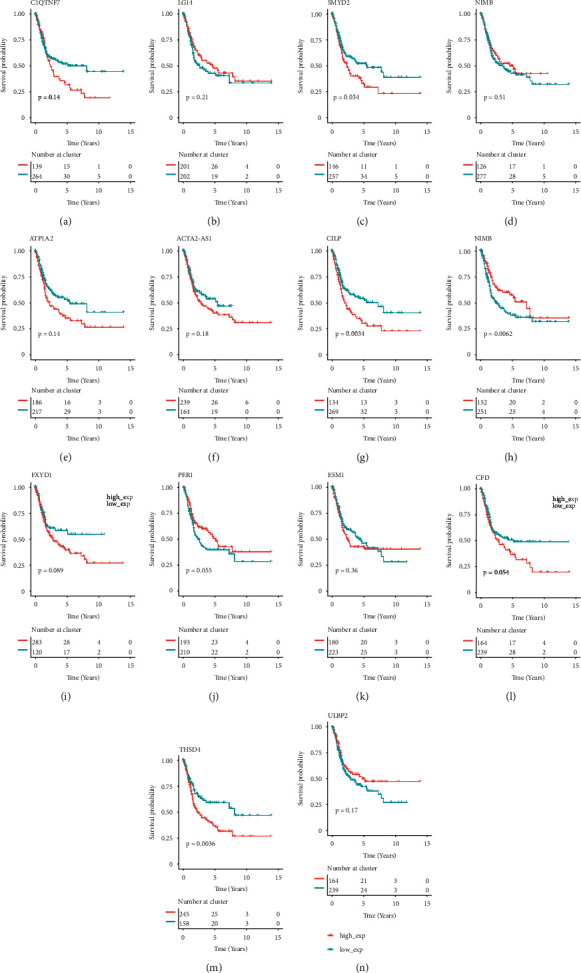
The Kaplan–Meier curve of feature genes.

We investigated the overall survival (OS) of BLCA patients in the TCGA dataset and plotted survival curves for each gene to investigate the effect of potential feature genes on BLCA patients' overall survival (OS). As shown in Figure 4, five distinct genes, including SMYD2, GAPDHP1, ATP1A2, CILP, and THSD4, were linked to BLCA OS with a *P* value of <0.05. The high-expression group had a greater survival rate than the low-expression group for GAPDHP1. The low-expression group had a greater survival rate than the high-expression group for SMYD2, ATP1A2, CILP, and THSD4.

**Figure 5:**
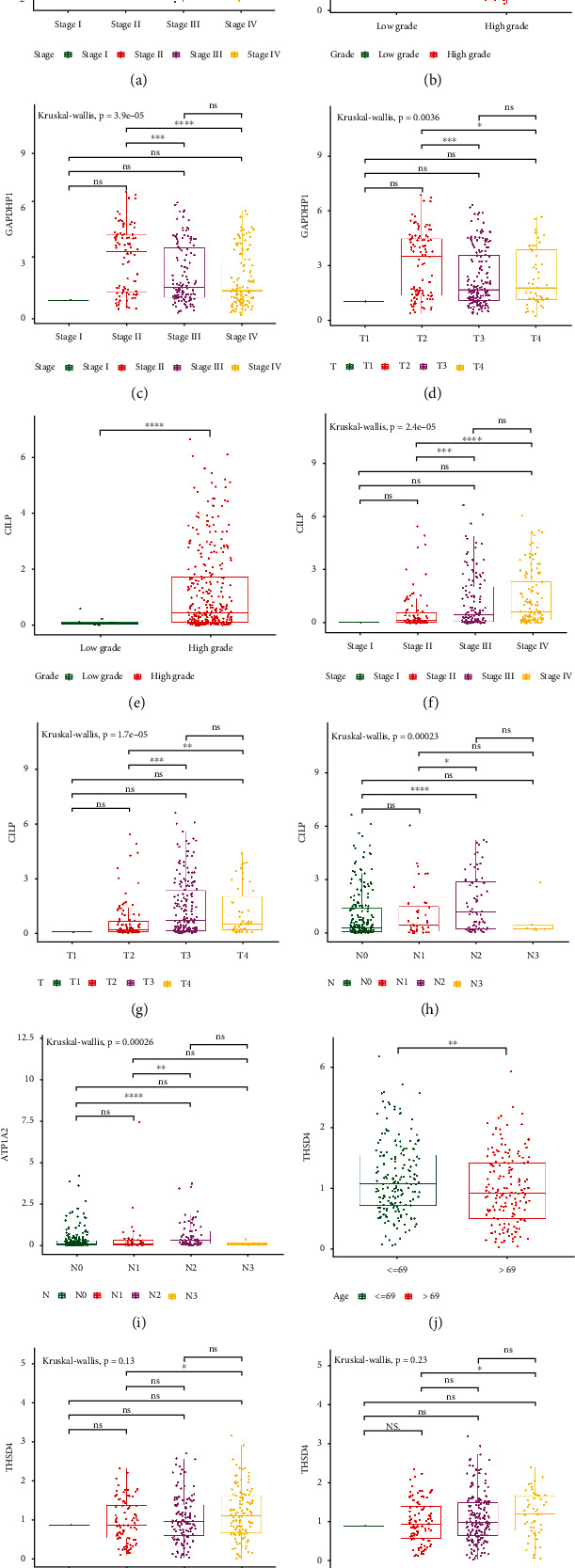
The correlation analysis of characteristic genes with clinicopathological features. (a) Expression of SMYD2 between different pathological grades. (b–d) GAPDHP1 expression between different clinical stages, pathological grades, and T stages. (e–h) Expression of CILP between different clinical stages, pathological grades, T stages, and N stages. (i) Expression of ATP1A2 in different N stages. (j–l) Expression of THSD4 in different ages, clinical stages, and T stages.

To investigate the role of the five genes in the progression of BLCA, we examined the relationships between the expression of characteristic genes and clinicopathological features (age, gender, grade, stage, T stage, and N stage). As shown in Fig. S3 and Figure 5(a), SMYD2 was not related to age, gender, grade, T stage, and N stage, but related to stage. The expression of SMYD2 tended to increase with the increase of stage. As SMYD2 was downregulated in BLCA, we speculated that SMYD2 played a promoting role in the occurrence and progression of BLCA. For GAPDHP1, GAPDHP1 was not related to age, gender, and N stage, but related to the grade, stage, and T stage (Fig. S4, Figures 5(b)–5(d)). We noted that the expression of GAPDHP1 was significantly lower in high-grade BLCA patients than in low-grade BLCA patients. The expression of GAPDHP1 was significantly lower in T3 and T4 stage BLCA patients than in T2 stage BLCA patients. The expression of GAPDHP1 was significantly lower in stage III and stage IV BLCA patients than in stage II BLCA patients. As GAPDHP1 was upregulated in BLCA, these results implied that GAPDHP1 played a promoting role in BLCA genesis and an inhibitory role in BLCA progression. CILP was not associated with age or gender, but rather with the grade, stage, T stage, and N stage (Fig. S5, Figures 5(e)–5(h)). We found that patients with high-grade BLCA had significantly higher CILP expression than those with low-grade BLCA. Patients with stage III and stage IV BLCA had significantly higher CILP expression than those with stage II BLCA. Since CILP was downregulated in BLCA, we presumed that CILP acted an inhibitory role in BLCA development and a facilitative role in BLCA progression. For ATP1A2, as shown in Fig. S6 and Figure 5(i), ATP1A2 was not associated with age, gender, grade, stage, and T stage, but related to N stage. As revealed in Fig. S7 and Figures 5(j)–5(l), THSD4 was correlated with age, stage, and T stage, but not related to gender, grade, and N stage. The expression of THSD4 tended to increase with the increasing stage.

### 3.4. Characteristic Genes and Immune Infiltration

**Figure 6:**
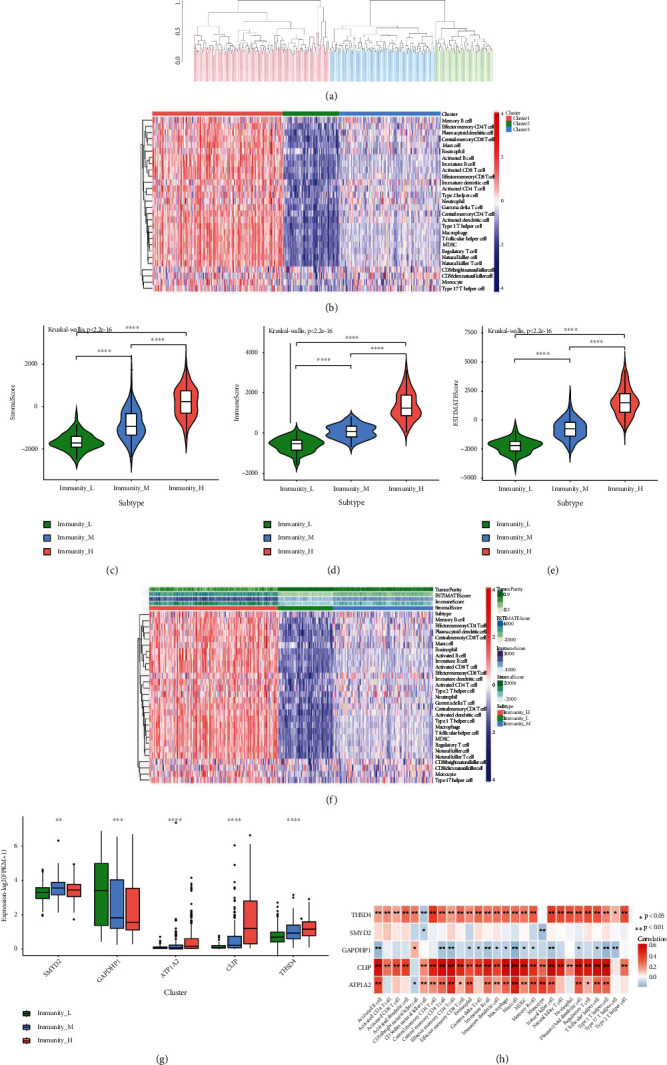
The correlation analysis between characteristic genes and immune infiltration. (a, b) Immune score clustering and heatmap of BLCA samples. Groups with high immunity (red), medium immunity (blue), and low immunity (green). (c–f) Immune score, stromal score, and ESTIMATE score variations among high-, medium-, and low-immunity groups. (g) Differential expression of characteristic genes in the groups with high, medium, and low immunity. (h) The correlation of characteristic genes with immune infiltrating cells.

We separated the BLCA patients in the TCGA database into three groups according to the immunological scores computed by ssGSEA: high immunity, medium immunity, and low immunity (Figures 6(a) and 6(b)). With the ESTIMATE algorithm, we found that the immune score, stromal score, and ESTIMATE score significantly increased among the three immunity groups with the increase of immune scores (Figures 6(c)–6(f)). Next, we analyzed the differential expression of five characteristic genes among the high-immunity group, medium-immunity group, and low-immunity group. The results showed that the expression of ATP1A2, CILP, and THSD4 was significantly increased, the expression of GAPDHP1 was significantly decreased, and the expression of SMYD2 was first increased and then decreased with the increase of immune score (Figure 6(g)). We also calculated the correlation between the characteristic genes and immune cells (Supplementary Table 6). Activated B cells, effector memory CD4 T cells, mast cells, memory B cells, and monocytes were all related to ATP1A2. The central memory CD4 T cell, natural killer cell, natural killer T cell, neutrophil, regulatory T cell, T follicular helper cell, and Type 2 T helper cell were all shown to be positively linked with THSD4. CILP was linked to activated B cells, activated CD8 T cells, activated dendritic cells, central memory CD4 T cells, central memory CD8 T cells, effector memory CD4 T cells, effector memory CD8 T cells, eosinophil, gamma delta T cells, immature B cells, immature dendritic cells, macrophage, mast cell, MDSC, memory B cell, natural killer cell, natural killer T cell, and plasmacytoid (Figure 6(h)).

### 3.5. Single-Gene GSEA

**Figure 7:**
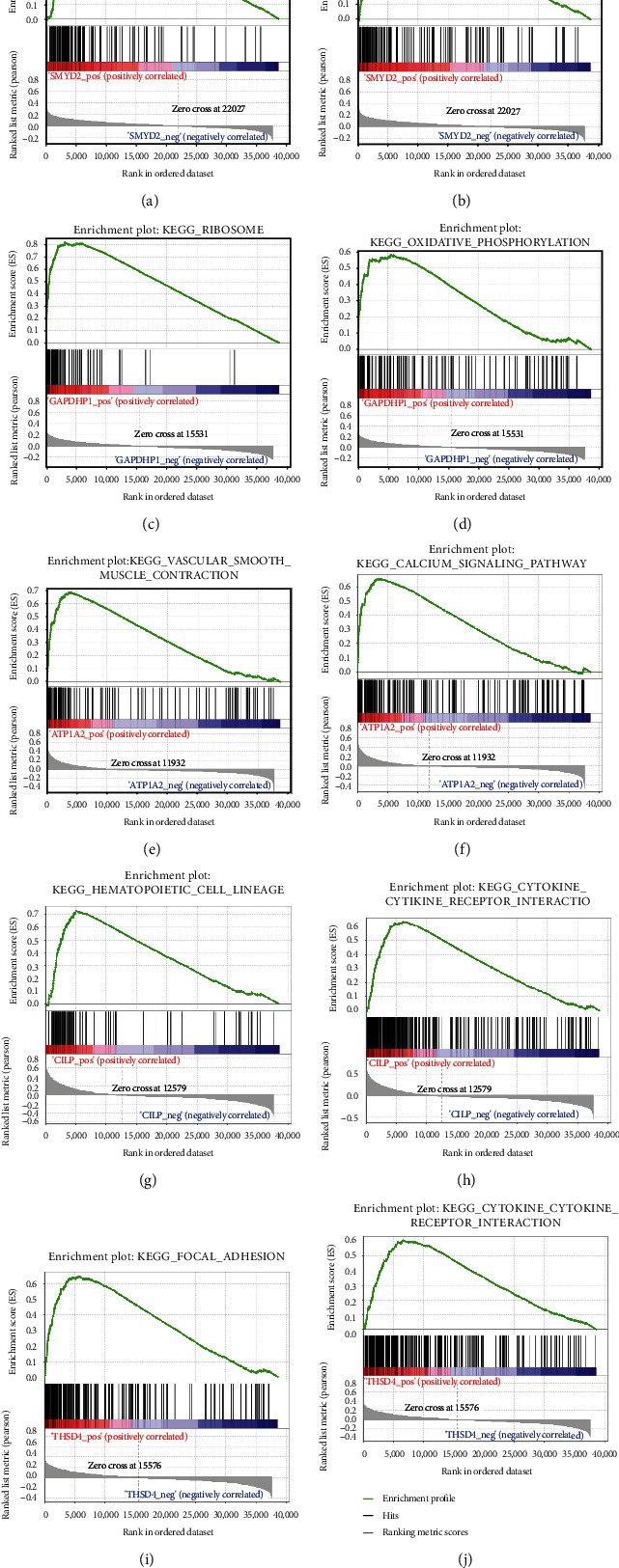
Enrichment plots from gene set enrichment analysis (GSEA). (a, b) Top 2 KEGG pathways enriched by SMYD2. (c, d) Top 2 KEGG pathways enriched by GAPDHP1. (e, f) Top 2 KEGG pathway enriched by ATP1A2. (g, h) Top 2 KEGG pathways enriched by SMYD2. (i, j) Top 2 KEGG pathways enriched by THSD4.

To probe the possible mechanism of the five characteristic genes in BLCA, we proceeded with the single-gene GSEA for each gene. Top 10 GO (BP) terms and KEGG pathways positively and negatively associated with each gene were listed in Supplementary Table 7. SMYD2 was most related to ‘spliceosome' and ‘cell cycle' (Figures 7(a) and 7(b)). GAPDHP1 was most associated with ‘ribosome' and ‘oxidative phosphorylation' (Figures 7(c) and 7(d)). ATP1A2 was most correlated with ‘vascular smooth muscle contraction' and ‘calcium signaling pathway' (Figures 7(e) and 7(f)). ‘Hematopoietic cell lineage' and ‘cytokine-cytokine receptor interaction' were most commonly associated with CILP (Figure 7(g) and 7(h)). ‘Focal adhesion' and ‘cytokine-cytokine receptor interaction' were most commonly related to THSD4 (Figures 7(i) and 7(j)).

### 3.6. Quantitative Real-Time PCR Validation

**Figure 8:**
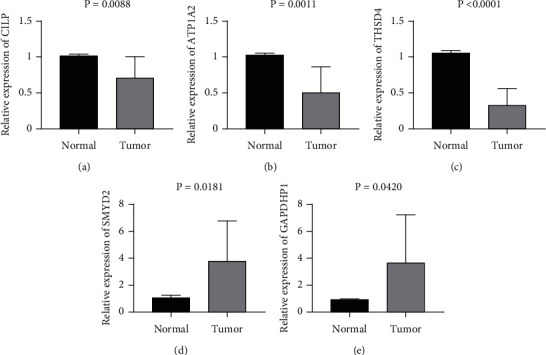
The expression of SMYD2, GAPDHP1, ATP1A2, CILP, and THSD4 in clinical BLCA tissues detected by qRT-PCR.

Quantitative real-time PCR was used to confirm the expression levels of SMYD2, GAPDHP1, ATP1A2, CILP, and THSD4 in BLCA tissues. We discovered that the expression levels of ATP1A2, CILP, and THSD4 were downregulated in malignant tissues compared to paraneoplastic tissues, which was consistent with the TCGA and GEO findings (Figures 8(a)–8(c)). Meanwhile, SMYD2 and GAPDHP1 expression levels in cancer tissues were higher than in paracancerous tissues (Figures 8(d) and 8(e)). As a result, we suggest ATP1A2, CILP, THSD4, SMYD2, and GAPDHP1 might be potential biomarkers for BLCA.

## 4. Discussion

The incidence and mortality rates of BLCA, one of the most frequent malignancies of the urinary tract, are increasing each year. It was reported that approximately 430,000 patients are diagnosed with BLCA each year and about 165,000 patients die [19]. Due to BLCA having no obvious symptoms in the early stage, the difficulty of diagnosis is increased. In addition, high recurrence and metastasis rates contribute to the low 5-year overall survival of BLCA patients, which has become a major challenge for global health [20]. Up to now, the main treatment for BLCA is surgery, chemotherapy, and immunotherapy. Although the clinical management of BLCA patients has improved after a series of rigorous treatments. However, the survival rate of patients with advanced stage was still low, and the effectiveness of immunotherapy still needs to be improved, as it benefits only a small proportion of patients [21]. Therefore, in order to combat this disease, screening for novel and promising diagnostic biomarkers and regulatory pathways for BLCA remains urgent and challenging.

In this study, we explore the underlying molecules and potential mechanisms that affect the prognosis of BLCA patients through a combination of bioinformatics and experimental validation. A total of 189 BLCA-related DEGs were identified. Then, after a series of bioinformatics analyses, we identified 5 especially outstanding characteristic genes, including SMYD2, GAPDHP1, ATP1A2, CILP, and THSD4, which were tightly linked to the progression and prognosis of BLCA. Moreover, we validated the expression levels of SMYD2, GAPDHP1, ATP1A2, CILP, and THSD4 in clinical samples. Interestingly, the results were consistent with those in the TCGA and GEO databases. Namely, the expression levels of ATP1A2, CILP, and THSD4 were downregulated in cancer tissues compared with paracancerous tissues, whereas the expression levels of SMYD2 and GAPDHP1 were upregulated. These findings may help improve treatment decisions, risk stratification, and prognosis prediction for BLCA patients.

In combination with the Sin3A and HDAC1 histone deacetylase complexes, SMYD2 is a lysine methyltransferase that not only methylates H3K36 but also acts as a transcriptional regulator [22]. Previous research has shown that SMYD2 is involved in the occurrence and progression of a variety of cancers [23]. SMYD2 expression was found to be highly upregulated in lung adenocarcinoma, and high SMYD2 expression was linked to shorter overall and disease-free survival. Mechanically, SMYD2 may promote carcinogenesis and metastasis by activating RPS7 transcription through binding to its promoter [24]. Inhibition of SMYD2 expression has been shown to significantly reduce cervical cancer proliferation in vivo and in vitro [25]. Furthermore, Meng et al. found that SMYD2 inhibits the expression of APC2, thereby activating the Wnt/*β*-catenin signaling pathway and promoting the epithelial-mesenchymal transition in colorectal cancer [26]. We discovered that high SMYD2 expression was not only related to poor survival in BLCA but also that SMYD2 expression increased with stage, which was consistent with our findings. Finally, we hypothesized that SMYD2 may promote the onset and development of BLCA.

As a well-known membrane protein, Na+/K+-ATPase is composed of an *α* subunit and a *β* subunit and is widely involved in various processes of human physiology and pathology [27]. The ATP1A2 gene encodes the *α*-2 subunit of Na+/K+-ATPase, a transmembrane protein that is responsible for creating and sustaining Na and K ion electrochemical gradients across the plasma membrane. Previously, ATP1A2 expression was found to be downregulated in breast cancer [28]. Another evidence for the participation of ATP1A2 in ovarian serous cystadenocarcinoma pathophysiology comes from Huang et al., which revealed that the expression of ATP1A2 was higher in adjacent normal bladder tissues than in tumor tissues [29]. Additionally, Zhang et al. found that ATP1A2 was regulated by the lncRNA FLJ42875 in laryngeal squamous cell carcinoma [30]. Herein, our analysis and experimental verification demonstrated that ATP1A2 is lowly expressed in BLCA, and overexpression of ATP1A2 was associated with unfavorable outcomes in BLCA patients.

THSD4 encodes thrombospondin type 1 domain containing 4, and its methylation status has been linked to poor survival in glioblastoma patients [31]. Furthermore, GATA3 regulates THSD4 expression and promotes the transition of normal cells into breast cancer through THSD4 dysregulation [32]. The expression of THSD4 mRNA was discovered to be downregulated in colorectal cancer samples by Liu et al. THSD4 expression was found to be downregulated in patients with poorly differentiated colorectal cancer, and patients with low THSD4 expression had lower survival rates [33]. Our findings, together with previous research, revealed the role of THSD4 in BLCA tumorigenesis regulation. However, more research into the particular molecular pathways of THSD4 in the evolution of BLCA is required. As for CILP and GAPDHP1, they were not reported to be involved in the BLCA progression. Therefore, more research into these two genes is required in the future.

The tumor microenvironment has an impact on tumor occurrence and recurrence, as well as tumor immunotherapy outcomes. Tumor-infiltrating immune cells are an important part of the tumor microenvironment, and the composition and distribution of these cells are linked to cancer prognosis [34]. Inflammatory infiltrating cells' location, type, and density in colorectal cancer are stronger predictors of survival than clinical and histological variables in previous research [35]. Given the importance of the tumor microenvironment in cancer progression and the fact that tumor-infiltrating immune cells are an important component of the tumor microenvironment, we used the TCGA dataset to conduct immune infiltration analysis to investigate the relationship between signature genes and immune cells. ATP1A2, CILP, and THSD4 were found to be considered positively connected with most immune cells, GAPDH1 was found to be significantly negatively correlated with most immune cells, and SMYD2 was shown to be strongly negatively correlated with natural killer cells and monocytes. As a result, we postulated that those genes have an impact on the immune microenvironment of BLCA, play a major role in BLCA tumor immunity regulation, and can reflect the immune status of BLCA patients.

Moreover, the current research also explored the possible mechanism of the five characteristic genes in BLCA, we proceeded with the single-gene GSEA for each gene. Our results demonstrate that SMYD2 was most related to spliceosome and cell cycle. GAPDHP1 was most associated with ribosome and oxidative phosphorylation. ATP1A2 was most correlated with vascular smooth muscle contraction and calcium signaling pathway. CILP was most related to hematopoietic cell lineage and cytokine-cytokine receptor interaction. THSD4 was most associated with focal adhesion and cytokine-cytokine receptor interaction. These data provided a basis for further investigation of the underlying molecular mechanism of these five genes.

## 5. Conclusions

In conclusion, our comprehensive bioinformatics analysis of BLCA datasets from TCGA and GEO revealed DEGs, molecular processes, and key pathways associated with BLCA. In addition, quantitative real-time PCR results showed that the expression levels of ATP1A2, CILP, and THSD4 were downregulated and the expressions of SMYD2 and GAPDHP1 were upregulated in cancer tissues compared with normal tissues. Therefore, this information may help to identify new biomarkers to effectively assess tumor staging, improve treatment, and aid in drug development. However, further research is needed to elucidate their specific mechanisms and biological roles in the occurrence and development of BLCA.

## Data Availability

On reasonable request, the corresponding author will provide the analyzed datasets generated during the study.

## Supplementary Materials

Supplementary Materials Supplementary Figure 1: WGCNA analysis of the TCGA dataset. Supplementary Figure 2: WGCNA analysis of the GSE133624 dataset. Supplementary Figures 3–7: clinical relevance of SMYD2, GAPDHP1, CILP, ATP1A2, and THSD4. Supplementary Table 1: primer sequences in the study. Supplementary Table 2: DEGs in the TCGA dataset. Supplementary Table 3: DEGs in the GSE133624 dataset. Supplementary Table 4: DEGs coexisting in the TCGA and GSE133624 datasets. Supplementary Table 5: feature genes were selected with the SVM-RFE algorithm. Supplementary Table 6: the correlation between the characteristic genes and immune cells. Supplementary Table 7: single-gene GSEA for prognostic genes.
