# Supplementary material for: Identification of Potential Biomarkers for Progression and Prognosis of Bladder Cancer by Comprehensive Bioinformatics Analysis
Source: J Oncol. 2022 Apr 19;2022:1802706. doi: 10.1155/2022/1802706 (PMC9042640; doi:10.1155/2022/1802706)
Supplement: Supplementary Materials — Supplementary Figure 1: WGCNA analysis of the TCGA dataset. Supplementary Figure 2: WGCNA analysis of the GSE133624 dataset. Supplementary Figures 3–7: clinical relevance of SMYD2, GAPDHP1, CILP, ATP1A2, and THSD4. Supplementary Table 1: primer sequences in the study. Supplementary Table 2: DEGs in the TCGA dataset. Supplementary Table 3: DEGs in the GSE133624 dataset. Supplementary Table 4: DEGs coexisting in the TCGA and GSE133624 datasets. Supplementary Table 5: feature genes were selected with the SVM-RFE algorithm. Supplementary Table 6: the correlation between the characteristic genes and immune cells. Supplementary Table 7: single-gene GSEA for prognostic genes. [file 1802706.f1.zip › 1802706.f1/Supplementary Table 1.docx]

**Primer sequences in the study**

| SMYD2 | Forward (5’-3’) | CTTCGGAAAAATTGTTAGCT |
| --- | --- | --- |
|  | Reverse (5’-3’) | TATCCTTGTCCTTGGTGGTA |
| ATP1A2 | Forward (5’-3’) | GGCACTGTGACCATCCTTTG |
|  | Reverse (5’-3’) | GAACTCCACCACCTTCCGCT |
| THSD4 | Forward (5’-3’) | TTGGGGATGTGGTTGACGAT |
|  | Reverse (5’-3’) | AGGCTTGAGGTGGCAGGATT |
| GAPDHP1 | Forward (5’-3’) | TGCTGATGCCCCAATGTTT |
|  | Reverse (5’-3’) | ATGTTCTGGAGAGCCCCGT |
| CILP | Forward (5’-3’) | CGGACGTATGGCATGTTCT |
|  | Reverse (5’-3’) | GCCCTGTGTCTGGATTGAG |
| GAPDH | Forward (5’-3’) | CCCATCACCATCTTCCAGG |
|  | Reverse (5’-3’) | CATCACGCCACAGTTTCCC |
