# Supplementary material for: Identification of Potential Biomarkers for Progression and Prognosis of Bladder Cancer by Comprehensive Bioinformatics Analysis
Source: J Oncol. 2022 Apr 19;2022:1802706. doi: 10.1155/2022/1802706 (PMC9042640; doi:10.1155/2022/1802706)
Supplement: Supplementary Materials — Supplementary Figure 1: WGCNA analysis of the TCGA dataset. Supplementary Figure 2: WGCNA analysis of the GSE133624 dataset. Supplementary Figures 3–7: clinical relevance of SMYD2, GAPDHP1, CILP, ATP1A2, and THSD4. Supplementary Table 1: primer sequences in the study. Supplementary Table 2: DEGs in the TCGA dataset. Supplementary Table 3: DEGs in the GSE133624 dataset. Supplementary Table 4: DEGs coexisting in the TCGA and GSE133624 datasets. Supplementary Table 5: feature genes were selected with the SVM-RFE algorithm. Supplementary Table 6: the correlation between the characteristic genes and immune cells. Supplementary Table 7: single-gene GSEA for prognostic genes. [file 1802706.f1.zip › 1802706.f1/Supplementary Table 2 .pdf]

Symbol	logFC	AveExpr	t	P.Value	adj.P.Val	B
HLF	-2.13094	0.36233	-20.7534	1.33E-66	5.13E-62	140.5525
PLP1	-1.61129	0.222337	-19.9165	7.60E-63	9.77E-59	132.0184
SCARA5	-3.7571	0.698794	-19.5988	2.02E-61	1.95E-57	128.7801
C16orf89	-3.16418	0.60165	-19.1442	2.21E-59	1.42E-55	124.1496
PI16	-5.29415	0.859107	-18.6459	3.75E-57	1.81E-53	119.0826
F10	-2.29651	0.631839	-18.3677	6.56E-56	2.81E-52	116.2582
C1QTNF7	-1.77911	0.433141	-18.3138	1.14E-55	4.40E-52	115.7116
PGM5P4	-1.87973	0.25347	-18.0068	2.66E-54	7.91E-51	112.6018
ADAMTS(	-1.63629	0.212348	-17.8719	1.06E-53	2.92E-50	111.2371
CLEC3B	-3.95559	1.619069	-17.5658	2.43E-52	6.25E-49	108.1468
MYOC	-2.27454	0.242743	-17.2717	4.88E-51	1.18E-47	105.1844
FAM107A	-2.9317	0.834148	-16.9051	2.03E-49	4.61E-46	101.5034
TMEM13	-1.15124	0.154369	-16.849	3.59E-49	7.69E-46	100.9418
TCF21	-2.44671	0.75416	-16.8324	4.25E-49	8.62E-46	100.7749
ADAMTS	-1.47618	0.281138	-16.3762	4.28E-47	7.86E-44	96.21982
GLP2R	-1.61376	0.212589	-16.3091	8.41E-47	1.48E-43	95.55225
LINC0108	-3.16298	0.954078	-16.2516	1.50E-46	2.42E-43	94.97992
ATP1A2	-2.95307	0.486487	-16.0546	1.09E-45	1.50E-42	93.02494
PYGM	-2.16886	0.468102	-15.9587	2.85E-45	3.79E-42	92.0753
ADH1B	-3.84638	0.801537	-15.8565	7.92E-45	9.86E-42	91.06475
TNXB	-2.59542	0.641225	-15.7196	3.11E-44	3.75E-41	89.7128
BHMT2	-1.8448	0.450278	-15.5724	1.35E-43	1.53E-40	88.26355
MYOCD	-2.4305	0.514562	-15.5317	2.03E-43	2.17E-40	87.86329
ECRG4	-2.96262	0.584737	-15.4459	4.76E-43	4.71E-40	87.02067
ASPA	-1.31079	0.316042	-15.3716	9.95E-43	9.60E-40	86.29158
RERGL	-1.46701	0.336432	-15.0689	1.99E-41	1.71E-38	83.33406
FXYD1	-1.21473	0.324861	-15.0595	2.18E-41	1.83E-38	83.24168
VIT	-1.91316	0.282358	-15.0144	3.41E-41	2.80E-38	82.80238
TMEM25	-1.72806	0.248554	-14.9566	6.02E-41	4.84E-38	82.24001
RBFOX3	-1.85408	0.252871	-14.9195	8.68E-41	6.83E-38	81.8796
NEGR1	-1.86486	0.461032	-14.8753	1.34E-40	1.03E-37	81.45066
ITGA8	-2.38575	0.968424	-14.8068	2.63E-40	1.99E-37	80.78576
KLHL41	-1.48815	0.285921	-14.7417	4.97E-40	3.69E-37	80.15541
PGM5-AS	-4.16264	0.785455	-14.672	9.84E-40	7.17E-37	79.48157
OGN	-3.30135	0.718444	-14.6675	1.03E-39	7.36E-37	79.43748
SLC2A4	-2.48951	0.546655	-14.647	1.26E-39	8.82E-37	79.24028
SCN7A	-1.19156	0.1794	-14.4754	6.72E-39	4.55E-36	77.58546
MYOM1	-1.98712	0.535951	-14.3903	1.54E-38	9.89E-36	76.76767
MICU3	-1.03143	0.306962	-14.3865	1.60E-38	1.01E-35	76.7305
FGF10	-1.28828	0.220482	-14.1878	1.10E-37	6.72E-35	74.82765
ADGRD1	-1.65401	0.351231	-14.172	1.28E-37	7.52E-35	74.67663
SGCG	-1.26489	0.209768	-14.1714	1.29E-37	7.52E-35	74.67076
MIR1-1HC	-1.24759	0.20442	-14.0269	5.19E-37	2.99E-34	73.29265
FAIM2	-1.43028	0.306435	-13.9368	1.24E-36	6.91E-34	72.43635
HAND2-A	-1.17693	0.205909	-13.9138	1.54E-36	8.50E-34	72.2179
TPPP	-2.24508	0.807094	-13.8978	1.80E-36	9.77E-34	72.06639
CHRM2	-1.74299	0.300287	-13.8247	3.63E-36	1.95E-33	71.37313
ABCA8	-1.54929	0.399894	-13.7779	5.68E-36	2.96E-33	70.93077
AFF3	-1.34759	0.258111	-13.5919	3.36E-35	1.71E-32	69.17639
ITIH5	-2.60731	1.130256	-13.541	5.45E-35	2.70E-32	68.69838
FENDRR	-2.73028	0.948349	-13.5153	6.96E-35	3.36E-32	68.45676

NR4A3	-3.0451	1.170465	-13.4401	1.42E-34	6.70E-32	67.75147
PDZRN4	-1.89835	0.35264	-13.4327	1.53E-34	7.10E-32	67.68207
FILIP1	-1.93087	0.576327	-13.4126	1.85E-34	8.48E-32	67.49451
UBE2C	3.421685	5.701118	13.33264	3.94E-34	1.77E-31	66.74715
CCL14	-1.27453	0.311838	-13.3326	3.94E-34	1.77E-31	66.747
SVEP1	-1.62607	0.739679	-13.3267	4.16E-34	1.85E-31	66.69175
PGM5	-3.87788	1.244396	-13.204	1.32E-33	5.62E-31	65.54915
PLCD4	-1.20149	0.660546	-13.1256	2.77E-33	1.16E-30	64.82105
EGR3	-2.85852	1.267228	-13.0015	8.88E-33	3.50E-30	63.67157
CPED1	-2.10357	0.754934	-12.9857	1.03E-32	4.01E-30	63.52616
CAVIN2	-3.40415	1.778412	-12.8776	2.82E-32	1.08E-29	62.53016
RBM24	-1.60626	0.400464	-12.8047	5.57E-32	2.07E-29	61.85958
AOX1	-1.97304	0.564249	-12.7112	1.33E-31	4.87E-29	61.00372
ANGPTL7	-1.09934	0.185467	-12.665	2.03E-31	7.34E-29	60.58109
NPY6R	-1.2547	0.17804	-12.6584	2.16E-31	7.72E-29	60.52115
ASB5	-2.12256	0.364844	-12.6529	2.28E-31	8.06E-29	60.47032
GFRA1	-1.40846	0.343554	-12.6487	2.36E-31	8.30E-29	60.43243
VEGFD	-1.64556	0.478643	-12.6283	2.85E-31	9.93E-29	60.2466
XPNPEP2	-1.76715	0.378167	-12.594	3.92E-31	1.34E-28	59.93377
GNG7	-1.77621	0.808269	-12.5478	6.00E-31	1.96E-28	59.51306
PID1	-2.11883	0.918129	-12.5281	7.20E-31	2.33E-28	59.33435
GPRASP1	-1.68402	0.84678	-12.5216	7.64E-31	2.46E-28	59.27475
SHISAL1	-1.92864	0.533888	-12.4543	1.42E-30	4.49E-28	58.66461
RANBP3L	-1.47462	0.300337	-12.4352	1.69E-30	5.31E-28	58.49125
PRIMA1	-1.52714	0.444806	-12.4189	1.97E-30	6.12E-28	58.34325
MBNL1-A	-1.73388	0.649535	-12.3511	3.66E-30	1.10E-27	57.73088
JAM2	-1.76813	0.963476	-12.3408	4.02E-30	1.20E-27	57.63809
FAXDC2	-1.58647	0.875265	-12.2943	6.15E-30	1.82E-27	57.21841
CFD	-4.23093	3.565964	-12.2605	8.37E-30	2.45E-27	56.91407
SYNPO2	-4.02923	1.656162	-12.252	9.04E-30	2.62E-27	56.83764
HSPB6	-5.07906	2.267111	-12.2338	1.07E-29	3.05E-27	56.67437
PPP1R12E	-2.68382	1.716246	-12.2192	1.22E-29	3.43E-27	56.54277
NR4A1AS	-1.84957	0.779707	-12.2081	1.35E-29	3.77E-27	56.44356
MORN5	-1.12284	0.174469	-12.2042	1.40E-29	3.88E-27	56.40857
KCNMA1	-1.67784	0.539038	-12.1846	1.67E-29	4.60E-27	56.23234
ADAM33	-2.21281	0.829081	-12.1702	1.90E-29	5.21E-27	56.10308
ABI3BP	-2.21258	0.8729	-12.1676	1.95E-29	5.30E-27	56.07967
ADRB3	-1.11513	0.220074	-12.145	2.39E-29	6.46E-27	55.87759
ZBTB16	-1.15149	0.269061	-12.1289	2.77E-29	7.42E-27	55.73325
HMCN2	-1.00006	0.264825	-12.0928	3.84E-29	1.02E-26	55.41078
NR4A1	-3.90239	3.070119	-12.0818	4.24E-29	1.11E-26	55.31205
PDE2A	-1.71654	0.92522	-12.0438	5.99E-29	1.56E-26	54.97257
CGNL1	-1.77899	0.833645	-12.016	7.69E-29	1.99E-26	54.72537
RCAN2	-2.61012	1.77787	-11.9975	9.10E-29	2.34E-26	54.55999
SYNE1	-1.1822	0.638648	-11.9959	9.23E-29	2.36E-26	54.54608
SOBP	-1.49763	0.640418	-11.9945	9.35E-29	2.37E-26	54.53313
SOX17	-1.76438	0.981273	-11.9803	1.06E-28	2.68E-26	54.40744
TK1	2.842874	5.411014	11.96681	1.20E-28	3.01E-26	54.28723
SYNM	-4.32321	1.882363	-11.9452	1.46E-28	3.60E-26	54.09515
LRFN5	-1.21991	0.287761	-11.9445	1.47E-28	3.60E-26	54.08919
C5orf66-A	-1.92067	0.599603	-11.9119	1.97E-28	4.78E-26	53.79913
ACACB	-1.48585	0.850534	-11.905	2.09E-28	5.05E-26	53.73828

P2RY14	-1.32661	0.547658	-11.8905	2.39E-28	5.68E-26	53.60941
GREM2	-1.52627	0.423742	-11.8769	2.69E-28	6.38E-26	53.48902
CDK1	2.391805	3.744677	11.85175	3.38E-28	7.95E-26	53.26655
MEN1	1.268704	3.887637	11.85	3.43E-28	8.02E-26	53.251
TMOD1	-2.12454	0.781849	-11.8313	4.06E-28	9.43E-26	53.08535
PLAC9	-2.75034	1.738772	-11.804	5.18E-28	1.20E-25	52.84433
PCOLCE2	-2.64271	0.932815	-11.7995	5.39E-28	1.24E-25	52.80491
P2RX1	-2.87581	0.875734	-11.7888	5.94E-28	1.36E-25	52.70996
PTGFR	-1.63016	0.495073	-11.7504	8.37E-28	1.89E-25	52.37149
PAMR1	-2.27442	1.535052	-11.7378	9.37E-28	2.09E-25	52.26033
IGSF10	-1.10968	0.227983	-11.7317	9.88E-28	2.19E-25	52.20724
CBX7	-1.92876	1.976066	-11.7279	1.02E-27	2.26E-25	52.17301
MOGS	1.348895	5.018846	11.69356	1.39E-27	3.03E-25	51.87129
NR3C2	-1.66	0.636978	-11.6874	1.47E-27	3.18E-25	51.81697
CSRNP1	-2.43838	3.584652	-11.6864	1.48E-27	3.19E-25	51.80802
PRDM6	-1.44754	0.55677	-11.6588	1.89E-27	4.06E-25	51.56592
ANK2	-1.21662	0.373372	-11.6459	2.12E-27	4.50E-25	51.45274
C7	-3.84947	1.227784	-11.576	3.95E-27	8.20E-25	50.8409
RNF150	-1.52251	0.538778	-11.55	4.97E-27	1.03E-24	50.6138
CRY2	-1.30184	2.336485	-11.5476	5.08E-27	1.04E-24	50.59247
LRRN4CL	-1.83244	0.900162	-11.5458	5.16E-27	1.05E-24	50.57703
ADCY5	-2.24045	0.796837	-11.5018	7.62E-27	1.55E-24	50.19322
GNAL	-1.55664	0.766577	-11.5	7.75E-27	1.57E-24	50.17724
ATP1B2	-1.18298	0.554173	-11.4828	9.01E-27	1.81E-24	50.02769
FOXF1	-2.76331	1.869836	-11.466	1.05E-26	2.09E-24	49.88087
ASB2	-2.13156	0.899839	-11.4479	1.23E-26	2.42E-24	49.72344
SLIT3	-2.56821	1.523954	-11.4428	1.28E-26	2.51E-24	49.6794
SORBS2	-2.10919	0.960752	-11.4285	1.46E-26	2.84E-24	49.55488
RCC2	1.446335	5.969465	11.3999	1.87E-26	3.63E-24	49.30687
ADAMTS	-3.29121	2.856243	-11.3929	1.99E-26	3.84E-24	49.24619
EPM2A	-1.08248	0.839324	-11.3532	2.82E-26	5.39E-24	48.90216
SLC52A2	1.612289	5.100158	11.34966	2.91E-26	5.54E-24	48.87155
DIXDC1	-1.85472	1.105605	-11.3485	2.94E-26	5.57E-24	48.86139
PDK4	-3.55861	2.190495	-11.3328	3.38E-26	6.36E-24	48.72527
KCNMB1	-2.48697	1.029904	-11.3308	3.44E-26	6.44E-24	48.70811
ODF3L1	-1.12578	0.433293	-11.3274	3.54E-26	6.60E-24	48.67922
GPIHBP1	-1.63428	0.649293	-11.3186	3.83E-26	7.10E-24	48.60257
NCAM1	-2.17764	0.558936	-11.2906	4.89E-26	8.94E-24	48.36108
DOK6	-1.18378	0.301358	-11.2822	5.26E-26	9.57E-24	48.28881
SLC25A2[illegible]	-1.9313	2.26228	-11.2544	6.71E-26	1.21E-23	48.04917
CADM3	-2.14658	0.515383	-11.247	7.15E-26	1.28E-23	47.98551
GALNT17	-1.74873	0.52863	-11.2281	8.44E-26	1.50E-23	47.82215
DTNA	-1.68322	0.580574	-11.2165	9.34E-26	1.65E-23	47.72258
ZWINT	2.392876	4.218002	11.20104	1.07E-25	1.88E-23	47.58983
ADAMTS[illegible]	-1.57131	0.51598	-11.1934	1.14E-25	2.00E-23	47.52389
ASF1B	2.314061	3.899115	11.15608	1.58E-25	2.75E-23	47.20383
KCND3	-1.70795	0.709095	-11.123	2.11E-25	3.63E-23	46.92037
KLF2	-2.75528	2.819087	-11.1206	2.15E-25	3.69E-23	46.89977
VSTM4	-1.63587	1.030437	-11.1083	2.39E-25	4.07E-23	46.79481
MIR100H[illegible]	-2.01837	0.918895	-11.1073	2.42E-25	4.09E-23	46.78574
PER2	-1.53073	1.742216	-11.0855	2.92E-25	4.92E-23	46.59951
FHL1	-3.87617	2.191309	-11.0757	3.18E-25	5.33E-23	46.5157

AGTR1	-1.53574	0.435702	-11.0288	4.77E-25	7.90E-23	46.11626
DMD	-1.67641	0.619078	-11.026	4.89E-25	8.06E-23	46.09173
TMEM22(	-1.04311	0.609344	-11.0218	5.07E-25	8.32E-23	46.05643
SRF	-1.34646	4.107764	-11.0156	5.34E-25	8.74E-23	46.00376
NFASC	-1.20981	0.54615	-10.9945	6.41E-25	1.04E-22	45.82416
NME1	1.609661	4.576142	10.98684	6.85E-25	1.11E-22	45.75869
GATA5	-1.76862	0.443795	-10.9671	8.12E-25	1.31E-22	45.59131
CDC20	2.922933	5.000658	10.95482	9.03E-25	1.45E-22	45.48661
RAB9B	-1.46555	0.623853	-10.9505	9.38E-25	1.50E-22	45.44974
LIMS2	-2.43656	1.618099	-10.9272	1.15E-24	1.83E-22	45.25199
PSD	-2.40157	1.113452	-10.9163	1.26E-24	1.99E-22	45.15962
PDE1A	-1.29571	0.56122	-10.9134	1.29E-24	2.03E-22	45.13554
HGH1	1.415844	3.999297	10.89487	1.51E-24	2.34E-22	44.97851
POLR2H	1.222798	4.493326	10.86939	1.88E-24	2.90E-22	44.76295
SMTN	-2.28303	3.155249	-10.8693	1.88E-24	2.90E-22	44.76187
ALG3	1.285359	4.43521	10.85948	2.05E-24	3.13E-22	44.67926
TCEAL2	-2.04061	0.553254	-10.8554	2.12E-24	3.23E-22	44.64459
CHRDL1	-2.60802	0.789227	-10.8327	2.58E-24	3.89E-22	44.45326
MAMDC2	-2.25594	0.890528	-10.8274	2.70E-24	4.03E-22	44.40877
HPSE2	-2.21931	0.61625	-10.8221	2.82E-24	4.19E-22	44.36371
CSRP1	-2.42047	4.487847	-10.8153	2.99E-24	4.42E-22	44.30671
LDB3	-1.05649	0.340292	-10.7812	4.00E-24	5.85E-22	44.01935
RCC1	1.574048	4.520965	10.77772	4.12E-24	6.01E-22	43.98999
KIF2C	2.282915	3.563315	10.77107	4.37E-24	6.33E-22	43.93406
TMEM10(	-1.35967	0.438529	-10.7647	4.61E-24	6.66E-22	43.88055
RAI2	-2.18894	1.486784	-10.748	5.31E-24	7.60E-22	43.74036
FBXL22	-1.52818	0.694776	-10.7224	6.61E-24	9.38E-22	43.52564
PSMG3	1.532606	4.729796	10.71991	6.75E-24	9.55E-22	43.5044
PTH1R	-1.13157	0.497169	-10.7189	6.81E-24	9.60E-22	43.49564
ACOX2	-1.32097	0.565426	-10.7143	7.08E-24	9.94E-22	43.45759
SGCA	-2.49897	0.94017	-10.7134	7.14E-24	9.98E-22	43.45002
CASQ2	-3.18198	0.95397	-10.7098	7.36E-24	1.03E-21	43.41928
MYH11	-5.5591	2.936255	-10.708	7.47E-24	1.04E-21	43.4045
TACR2	-2.10238	0.679203	-10.707	7.54E-24	1.04E-21	43.39614
NR4A2	-2.48949	2.146575	-10.7069	7.54E-24	1.04E-21	43.39537
JPT1	1.92363	5.93857	10.6942	8.40E-24	1.15E-21	43.28898
TCEAL7	-1.87239	1.041395	-10.6727	1.01E-23	1.38E-21	43.10901
TROAP	2.139251	2.864561	10.66413	1.08E-23	1.47E-21	43.03732
FAM189A	-1.40621	0.735626	-10.6628	1.10E-23	1.48E-21	43.02619
WFDC1	-1.80274	1.123567	-10.6601	1.12E-23	1.51E-21	43.0038
TPX2	2.796444	4.660332	10.65637	1.16E-23	1.55E-21	42.97249
UBE2T	2.046599	4.290889	10.64703	1.25E-23	1.67E-21	42.89439
CDCA8	2.274633	3.572915	10.63402	1.40E-23	1.86E-21	42.78575
LMOD1	-4.21348	2.47616	-10.6156	1.64E-23	2.16E-21	42.63217
TBX20	-1.28536	0.317249	-10.61	1.72E-23	2.25E-21	42.58539
LGI4	-1.44438	0.854337	-10.61	1.72E-23	2.25E-21	42.58538
GATA6	-2.00332	1.177619	-10.6062	1.77E-23	2.30E-21	42.55373
FLNC	-3.89342	1.846091	-10.6058	1.78E-23	2.30E-21	42.55031
TNS1	-3.21734	2.496178	-10.573	2.35E-23	3.02E-21	42.27738
CFL2	-2.0031	1.72701	-10.5491	2.87E-23	3.67E-21	42.07875
HMGB3	2.006225	4.526535	10.54257	3.03E-23	3.86E-21	42.02426
AARD	-1.43394	0.310137	-10.5101	3.99E-23	5.06E-21	41.75486

PHYHIP	-1.89176	0.802159	-10.5023	4.26E-23	5.39E-21	41.68982
HAND2	-2.00399	0.717314	-10.4447	6.91E-23	8.69E-21	41.21319
PRUNE2	-2.22499	0.707241	-10.4413	7.11E-23	8.91E-21	41.18532
CACNA1I	-2.4187	1.378776	-10.4107	9.19E-23	1.14E-20	40.93308
MYLK	-3.10719	2.006504	-10.4093	9.30E-23	1.15E-20	40.92104
AURKB	2.387894	3.746472	10.40798	9.40E-23	1.16E-20	40.9106
SORBS1	-3.1382	1.667348	-10.4023	9.85E-23	1.22E-20	40.86414
SLIT2	-1.56742	0.677065	-10.3902	1.09E-22	1.34E-20	40.76415
PLPPR4	-1.25134	0.460665	-10.384	1.15E-22	1.41E-20	40.71316
RECQL4	2.063067	3.39655	10.34292	1.62E-22	1.97E-20	40.37518
EIF2AK1	1.111374	5.145482	10.34237	1.63E-22	1.97E-20	40.37067
REEP1	-1.83763	0.576089	-10.3379	1.69E-22	2.04E-20	40.33425
FBXL6	1.541913	3.426632	10.32088	1.95E-22	2.34E-20	40.19432
AURKA	2.127966	3.406841	10.30752	2.17E-22	2.61E-20	40.08478
EBF1	-1.42914	0.809658	-10.3037	2.24E-22	2.68E-20	40.05373
HJURP	1.90537	2.676293	10.25091	3.48E-22	4.11E-20	39.62154
CALR	1.273356	8.684056	10.22988	4.14E-22	4.88E-20	39.44985
BIRC5	2.425882	3.956178	10.2232	4.38E-22	5.14E-20	39.39532
PRELP	-3.1357	1.720687	-10.2134	4.75E-22	5.55E-20	39.31568
CSGALNA	-1.48875	1.381069	-10.206	5.05E-22	5.89E-20	39.25537
CCNB1	2.323389	4.585925	10.20277	5.19E-22	6.03E-20	39.22884
HAAO	-1.71935	1.351136	-10.1987	5.37E-22	6.22E-20	39.1956
CACNB2	-1.13918	0.484733	-10.1959	5.49E-22	6.34E-20	39.17316
EPHA3	-1.61298	0.851917	-10.1794	6.29E-22	7.25E-20	39.03855
LTBP4	-2.35658	3.183497	-10.1711	6.74E-22	7.74E-20	38.97072
COL14A1	-2.75704	1.972593	-10.1685	6.89E-22	7.89E-20	38.94996
PLCB4	-1.71767	0.800637	-10.1585	7.48E-22	8.51E-20	38.86857
NECAB1	-1.19147	0.46388	-10.1348	9.09E-22	1.03E-19	38.67645
CDT1	2.133493	3.321877	10.13183	9.32E-22	1.05E-19	38.65214
SLC66A1	1.142647	3.235946	10.11425	1.08E-21	1.22E-19	38.50964
FGF2	-1.51522	0.639481	-10.1077	1.14E-21	1.28E-19	38.4569
PPP1R14E	1.558351	6.230282	10.09722	1.24E-21	1.39E-19	38.37172
KIF18B	1.984568	2.530736	10.09567	1.26E-21	1.40E-19	38.35911
TOP2A	2.617067	4.476294	10.08966	1.32E-21	1.46E-19	38.3105
PDE4D	-1.2932	1.15759	-10.0876	1.34E-21	1.48E-19	38.29352
CELF2	-1.70608	1.072612	-10.0719	1.53E-21	1.68E-19	38.16656
SPARCL1	-3.16724	4.589447	-10.058	1.71E-21	1.87E-19	38.05498
MIR27A	-1.90104	0.625332	-10.0543	1.76E-21	1.93E-19	38.02466
TNS2	-1.74239	2.355379	-10.0378	2.02E-21	2.19E-19	37.89166
CCNB2	2.165591	3.746818	10.01122	2.51E-21	2.69E-19	37.67734
CCDC69	-2.29826	2.78366	-9.99802	2.79E-21	2.99E-19	37.57105
PLPP3	-2.00039	3.190395	-9.99651	2.83E-21	3.02E-19	37.55895
MMRN1	-1.40298	0.515765	-9.98219	3.18E-21	3.38E-19	37.44379
KIF20A	2.136445	3.208324	9.968121	3.57E-21	3.78E-19	37.33073
FGL2	-2.88448	2.034719	-9.96437	3.68E-21	3.89E-19	37.30062
DACT3	-1.9863	0.982617	-9.96203	3.75E-21	3.95E-19	37.28183
FBXL7	-1.61924	1.120402	-9.95857	3.86E-21	4.06E-19	37.25405
MYBL2	3.003624	4.755837	9.93719	4.59E-21	4.79E-19	37.08256
RASL12	-2.3589	1.767572	-9.92576	5.04E-21	5.24E-19	36.99099
GNAO1	-1.33672	0.447582	-9.91028	5.72E-21	5.88E-19	36.86706
PDE5A	-1.59504	1.173774	-9.90946	5.75E-21	5.91E-19	36.86045
CLEC3A	-1.53718	0.265779	-9.90772	5.84E-21	5.97E-19	36.84656

EPHA7	-1.56088	0.571661	-9.90275	6.08E-21	6.20E-19	36.80678
MRGPRF	-2.86179	2.014183	-9.88534	7.00E-21	7.11E-19	36.66761
APOLD1	-1.99373	2.024651	-9.87907	7.37E-21	7.46E-19	36.61752
LEPR	-1.02945	0.70816	-9.87423	7.66E-21	7.74E-19	36.57889
RNASEH2	1.627885	4.367927	9.868183	8.05E-21	8.11E-19	36.53061
FAM180A	-1.06414	0.371743	-9.86759	8.08E-21	8.12E-19	36.52586
SLC25A39	1.236473	5.885267	9.8645	8.29E-21	8.31E-19	36.50122
PRKG1	-1.40536	0.746556	-9.86282	8.40E-21	8.40E-19	36.48781
SLMAP	-1.52339	2.352304	-9.86119	8.51E-21	8.49E-19	36.47483
CKS2	2.06185	5.93167	9.859219	8.65E-21	8.61E-19	36.45908
LINC0064	-1.03667	0.922566	-9.85462	8.98E-21	8.91E-19	36.42243
SPAG5	1.915467	3.194038	9.851764	9.19E-21	9.08E-19	36.39963
IRAG1	-2.31318	1.678257	-9.85162	9.20E-21	9.08E-19	36.39848
STUM	-1.29441	0.317927	-9.8454	9.68E-21	9.53E-19	36.34886
ACTA2-A	-1.37265	0.554432	-9.84427	9.77E-21	9.57E-19	36.33994
AOC3	-3.04862	2.493159	-9.83818	1.03E-20	1.00E-18	36.2914
CNN1	-5.05665	3.338115	-9.83407	1.06E-20	1.03E-18	36.25862
SKA3	1.67212	2.402034	9.829602	1.10E-20	1.07E-18	36.22306
LIG1	1.345542	3.350101	9.827526	1.12E-20	1.08E-18	36.20654
LMO3	-1.77072	0.598016	-9.82231	1.17E-20	1.13E-18	36.16505
NUSAP1	2.26504	4.01272	9.811476	1.27E-20	1.23E-18	36.07883
PRICKLE2	-1.45009	1.185234	-9.8087	1.30E-20	1.25E-18	36.05672
BMP5	-1.6096	0.537531	-9.78973	1.52E-20	1.45E-18	35.90601
KLF9	-2.2626	2.417343	-9.76301	1.88E-20	1.79E-18	35.69399
PDZRN3	-2.14869	1.245213	-9.75652	1.98E-20	1.88E-18	35.64254
ITPKB	-1.37699	2.634794	-9.75649	1.98E-20	1.88E-18	35.64236
HIF3A	-1.4796	0.422033	-9.72801	2.50E-20	2.36E-18	35.41688
COASY	1.078881	4.672536	9.688218	3.44E-20	3.23E-18	35.10253
SHMT2	1.441139	4.775867	9.676854	3.76E-20	3.52E-18	35.01291
GSN	-2.20391	5.01872	-9.67643	3.78E-20	3.52E-18	35.00955
MIR221	-1.72407	0.750169	-9.67537	3.81E-20	3.54E-18	35.00118
H2AX	1.795913	5.621692	9.667872	4.04E-20	3.75E-18	34.94212
IQGAP3	1.999653	2.680249	9.651588	4.61E-20	4.26E-18	34.8139
UHRF1	1.771975	2.474603	9.651207	4.62E-20	4.27E-18	34.8109
FOSB	-4.09167	3.490519	-9.64651	4.80E-20	4.42E-18	34.77395
NXPH3	-1.45538	0.680826	-9.64224	4.96E-20	4.56E-18	34.74036
LAMA2	-1.70342	1.232465	-9.62892	5.52E-20	5.06E-18	34.63564
PAFAH1B	2.082855	5.42151	9.626167	5.64E-20	5.16E-18	34.614
ITPR1	-1.65115	1.3343	-9.62023	5.92E-20	5.40E-18	34.56739
DPT	-3.69995	1.755297	-9.61775	6.04E-20	5.49E-18	34.54787
TARBP2	1.101634	3.289302	9.617521	6.05E-20	5.49E-18	34.54609
JAM3	-2.08784	1.882586	-9.6141	6.22E-20	5.62E-18	34.51925
ACTC1	-4.16593	1.400921	-9.61404	6.22E-20	5.62E-18	34.51876
POP7	1.207715	5.225603	9.609009	6.47E-20	5.84E-18	34.47927
C11orf96	-3.11547	3.250939	-9.59648	7.15E-20	6.42E-18	34.38098
KIFC1	2.125254	3.696716	9.580331	8.14E-20	7.27E-18	34.25442
CDC6	1.811463	2.900232	9.567494	9.01E-20	8.00E-18	34.15391
TACC3	1.93675	3.818725	9.560797	9.51E-20	8.41E-18	34.10152
DUSP1	-3.27589	6.131833	-9.55423	1.00E-19	8.84E-18	34.05015
GEM	-2.92092	2.326926	-9.55283	1.01E-19	8.91E-18	34.03922
LAMC3	-1.82047	1.316695	-9.55267	1.01E-19	8.91E-18	34.03798
FXYD6	-2.5786	1.439502	-9.53408	1.18E-19	1.03E-17	33.89274

COX7A1	-2.33276	2.4067	-9.51914	1.32E-19	1.15E-17	33.77612
CIAO2A	1.023128	3.913119	9.515509	1.36E-19	1.18E-17	33.74779
SH3BGR	-1.60456	1.681964	-9.50342	1.50E-19	1.29E-17	33.65355
RAD51	1.532694	2.462244	9.499536	1.55E-19	1.33E-17	33.62328
EGR1	-3.30725	5.461457	-9.49346	1.62E-19	1.39E-17	33.57599
DUS1L	1.326872	4.669616	9.488786	1.68E-19	1.43E-17	33.53957
SGCD	-1.27714	0.530231	-9.48875	1.68E-19	1.43E-17	33.53933
MEF2D	-1.01754	3.23741	-9.48721	1.70E-19	1.45E-17	33.52732
RBMS3	-1.16247	0.687399	-9.47829	1.83E-19	1.55E-17	33.4579
C19orf48	1.567184	4.678155	9.476931	1.85E-19	1.56E-17	33.44732
GTSE1	1.674063	2.317019	9.474633	1.88E-19	1.59E-17	33.42945
BCHE	-1.79653	0.599301	-9.46991	1.95E-19	1.64E-17	33.39271
PER1	-2.071	2.774602	-9.46313	2.06E-19	1.73E-17	33.34001
NBEA	-1.21074	0.547015	-9.45045	2.28E-19	1.90E-17	33.2415
ITGB1BP2	-1.07107	0.702284	-9.44989	2.29E-19	1.90E-17	33.23719
YDJC	1.613481	4.257813	9.445422	2.37E-19	1.97E-17	33.2025
NEK2	1.927493	2.736572	9.443427	2.41E-19	1.99E-17	33.18702
PTGS1	-3.3804	2.155109	-9.44094	2.46E-19	2.03E-17	33.16772
NACC2	-1.55618	2.03716	-9.43988	2.48E-19	2.04E-17	33.15951
HSPB2	-1.07026	0.558526	-9.43705	2.53E-19	2.08E-17	33.13751
TEDC2	1.525305	2.047135	9.435595	2.56E-19	2.10E-17	33.12626
DSTN	-1.42779	6.586601	-9.4297	2.68E-19	2.20E-17	33.08058
LPP	-1.75096	2.507853	-9.42542	2.78E-19	2.26E-17	33.0474
CCDC137	1.156291	4.245781	9.41901	2.92E-19	2.37E-17	32.9977
PARD3B	-1.07921	0.729875	-9.41487	3.02E-19	2.45E-17	32.96561
OMD	-1.45872	0.478858	-9.41317	3.06E-19	2.47E-17	32.95244
MPZ	-1.26651	0.936079	-9.40821	3.18E-19	2.56E-17	32.91403
AQP1	-2.41444	4.284741	-9.39744	3.46E-19	2.78E-17	32.83072
C1QTNF2	-1.04127	0.621929	-9.39432	3.55E-19	2.85E-17	32.8066
DDX39A	1.247335	4.697531	9.391071	3.64E-19	2.91E-17	32.78146
AUP1	1.05096	6.202689	9.386758	3.76E-19	3.01E-17	32.74811
CDCA3	1.642603	2.346675	9.378011	4.03E-19	3.22E-17	32.68052
CDC45	1.891153	2.88568	9.374863	4.13E-19	3.29E-17	32.6562
ZFPM2	-1.06018	0.536349	-9.37439	4.15E-19	3.29E-17	32.65256
PELI2	-1.52787	1.051239	-9.37288	4.20E-19	3.33E-17	32.64086
MKI67	2.039829	3.197666	9.362315	4.56E-19	3.61E-17	32.55933
LSM4	1.137474	5.301238	9.361126	4.60E-19	3.63E-17	32.55016
ANGPTL1	-1.815	0.618187	-9.35797	4.72E-19	3.72E-17	32.52584
CASP2	1.100296	2.972611	9.357549	4.74E-19	3.72E-17	32.52256
TBX5	-1.09289	0.44987	-9.35381	4.88E-19	3.83E-17	32.49375
ZFP36	-2.68954	6.722259	-9.34314	5.30E-19	4.15E-17	32.41143
PCLAF	1.813771	3.02591	9.342699	5.32E-19	4.16E-17	32.40807
RPN1	1.04581	6.665432	9.326922	6.02E-19	4.69E-17	32.28655
NEXN	-2.20359	1.639169	-9.32611	6.06E-19	4.71E-17	32.28034
MIR23A	-2.09496	1.052922	-9.31842	6.44E-19	4.97E-17	32.22114
THSD4	-1.36229	1.130068	-9.31839	6.44E-19	4.97E-17	32.22091
CEP55	2.042944	3.282125	9.297614	7.57E-19	5.83E-17	32.06118
CD34	-1.70766	2.271547	-9.28993	8.04E-19	6.18E-17	32.00219
DNAJB5	-1.77978	1.436237	-9.28868	8.12E-19	6.22E-17	31.99256
POPDC2	-2.13956	1.266534	-9.28854	8.13E-19	6.22E-17	31.99148
MAOB	-2.77613	1.5104	-9.28671	8.25E-19	6.30E-17	31.97749
GINS1	1.721726	2.75178	9.28501	8.36E-19	6.37E-17	31.96441

ZCCHC24	-2.13523	2.12842	-9.28429	8.40E-19	6.40E-17	31.95892
KIF4A	1.891349	2.776316	9.281964	8.56E-19	6.49E-17	31.94104
PDIA4	1.515619	6.559354	9.281724	8.57E-19	6.49E-17	31.93919
MAP3K20	-1.8842	2.052116	-9.27525	9.02E-19	6.81E-17	31.88954
FANCI	1.450847	2.861274	9.271297	9.30E-19	6.98E-17	31.85922
TONSL	1.390812	2.595434	9.271044	9.32E-19	6.98E-17	31.85727
PDZD4	-1.49035	0.63196	-9.26533	9.74E-19	7.29E-17	31.81346
SPC24	1.751745	2.836306	9.253687	1.07E-18	7.96E-17	31.72429
STON1	-1.47869	1.099425	-9.25308	1.07E-18	7.99E-17	31.7196
FLAD1	1.137556	3.997273	9.246978	1.12E-18	8.33E-17	31.67292
ITM2A	-2.45783	2.026531	-9.23602	1.22E-18	9.05E-17	31.58909
TMEM132	2.038517	3.390666	9.230616	1.28E-18	9.42E-17	31.54776
LRRC45	1.470294	3.577729	9.220149	1.38E-18	1.01E-16	31.46777
CCNA2	2.014575	3.541685	9.201002	1.61E-18	1.17E-16	31.3216
FNBP1	-1.67791	2.822228	-9.19523	1.68E-18	1.22E-16	31.27755
CRYAB	-2.98999	2.349681	-9.19146	1.73E-18	1.25E-16	31.24885
CBX3	1.047522	5.534029	9.191116	1.74E-18	1.25E-16	31.24621
CMA1	-1.80611	0.507456	-9.17766	1.93E-18	1.39E-16	31.14372
OLFM1	-1.4963	0.639621	-9.17344	1.99E-18	1.43E-16	31.11154
OLFML1	-1.53877	1.143649	-9.1715	2.02E-18	1.45E-16	31.09681
MGME1	1.144348	3.814024	9.170539	2.04E-18	1.46E-16	31.08947
KANK2	-2.07557	3.158202	-9.16632	2.10E-18	1.50E-16	31.05737
TP53INP2	-1.91669	3.159806	-9.16172	2.18E-18	1.55E-16	31.02237
RHOB	-2.03985	6.408194	-9.14833	2.42E-18	1.71E-16	30.92054
PPP1R14B	1.606834	5.527576	9.148268	2.42E-18	1.71E-16	30.92008
DPP3	1.155614	4.322652	9.145295	2.47E-18	1.75E-16	30.89749
ZEB1	-1.74962	1.445609	-9.11376	3.16E-18	2.21E-16	30.65816
ROR1	-1.02052	0.459225	-9.08323	3.99E-18	2.78E-16	30.42704
RERG	-1.51738	0.933669	-9.08262	4.01E-18	2.79E-16	30.42236
SMUG1	1.024181	3.127586	9.072022	4.35E-18	3.01E-16	30.34227
GHR	-1.02047	0.4113	-9.07145	4.37E-18	3.01E-16	30.33793
RBPMS2	-2.55005	1.800777	-9.06647	4.54E-18	3.13E-16	30.30031
BDKRB1	-1.49026	0.815546	-9.06043	4.76E-18	3.27E-16	30.25469
TTYH3	1.545338	4.438418	9.056573	4.90E-18	3.36E-16	30.2256
NCAPH	1.852444	2.805441	9.053001	5.04E-18	3.44E-16	30.19864
CDCA5	2.03581	3.272308	9.042119	5.48E-18	3.74E-16	30.11655
MAP1B	-2.24596	1.595125	-9.04039	5.55E-18	3.78E-16	30.10349
ACTG2	-4.87972	4.039985	-9.02909	6.05E-18	4.11E-16	30.01834
PLK1	2.049372	3.29663	9.018979	6.54E-18	4.43E-16	29.94223
OXLD1	1.362442	4.230779	9.017709	6.61E-18	4.46E-16	29.93266
SCN4B	-1.02322	0.570428	-9.00834	7.10E-18	4.77E-16	29.86217
TTK	1.645701	2.2765	9.001448	7.48E-18	5.02E-16	29.81035
CENPM	1.764834	3.104343	8.999783	7.58E-18	5.08E-16	29.79784
RECK	-1.1745	0.959107	-8.96278	1.01E-17	6.70E-16	29.52013
FAM110A	1.519617	4.025537	8.954588	1.07E-17	7.11E-16	29.45872
HAND1	-1.29122	0.282769	-8.95383	1.08E-17	7.14E-16	29.45303
NDNF	-1.32246	0.493207	-8.94878	1.12E-17	7.41E-16	29.41519
SNRPB	1.28134	7.69027	8.935644	1.24E-17	8.14E-16	29.31692
SKA1	1.643367	2.344976	8.934184	1.25E-17	8.22E-16	29.306
MIX23	1.051599	3.572752	8.926849	1.32E-17	8.68E-16	29.25116
SEMA3E	-1.16831	0.540098	-8.92197	1.37E-17	8.99E-16	29.21469
DDR2	-1.92594	1.348381	-8.9139	1.46E-17	9.54E-16	29.1544

CH25H	-1.9753	1.342088	-8.90062	1.61E-17	1.05E-15	29.05532
BCL2L12	1.353735	3.70643	8.896482	1.67E-17	1.08E-15	29.02444
INMT	-1.4701	1.021693	-8.8918	1.73E-17	1.12E-15	28.98955
CENPA	1.885414	2.52347	8.888365	1.77E-17	1.15E-15	28.96393
BUB1B	1.674458	2.494779	8.886276	1.80E-17	1.17E-15	28.94836
SMOC2	-2.98783	2.318104	-8.88529	1.81E-17	1.17E-15	28.94101
CYB561	1.369014	4.519084	8.866846	2.09E-17	1.34E-15	28.80369
PACC1	1.229052	2.559327	8.86323	2.14E-17	1.37E-15	28.77679
PLN	-3.04064	1.526461	-8.85932	2.21E-17	1.41E-15	28.74769
RGS2	-2.9389	3.888603	-8.84885	2.39E-17	1.52E-15	28.6699
MSRB3	-2.50522	1.974581	-8.83553	2.64E-17	1.68E-15	28.57096
MFAP4	-3.8229	3.904331	-8.83156	2.72E-17	1.73E-15	28.5415
MRGBP	1.054962	3.588017	8.829599	2.76E-17	1.75E-15	28.52696
PJA2	-1.37534	3.677107	-8.82558	2.85E-17	1.80E-15	28.49717
ARHGAP:	1.143664	2.074003	8.825238	2.86E-17	1.80E-15	28.49461
AGRN	1.800366	5.366951	8.821348	2.94E-17	1.85E-15	28.46576
NUF2	1.938687	2.706791	8.814295	3.10E-17	1.95E-15	28.41348
POLD1	1.29426	3.244519	8.813117	3.13E-17	1.96E-15	28.40476
TNFAIP8I	-1.80958	1.210779	-8.81111	3.18E-17	1.99E-15	28.3899
P4HB	1.120784	7.729707	8.809384	3.22E-17	2.01E-15	28.3771
LYVE1	-1.94938	0.904288	-8.7993	3.47E-17	2.16E-15	28.30247
EFNA4	1.531741	3.915649	8.797776	3.51E-17	2.18E-15	28.29116
PPP1R14A	-2.41316	2.385263	-8.79556	3.57E-17	2.21E-15	28.27479
PRKCB	-1.41007	0.766002	-8.78366	3.91E-17	2.42E-15	28.18673
CHTF18	1.408153	2.616663	8.766957	4.43E-17	2.73E-15	28.06336
DNAJB11	1.031689	4.070181	8.763682	4.54E-17	2.79E-15	28.03918
ORC6	1.453254	2.082125	8.761771	4.61E-17	2.82E-15	28.02508
FOS	-3.2041	6.281611	-8.76142	4.62E-17	2.83E-15	28.02247
TMUB1	1.196292	4.968216	8.759821	4.68E-17	2.85E-15	28.01069
NFIA	-1.51612	1.8783	-8.7334	5.70E-17	3.47E-15	27.81597
CTSG	-2.22634	0.913324	-8.72851	5.92E-17	3.59E-15	27.77994
MAP1A	-1.69007	1.052948	-8.71642	6.48E-17	3.91E-15	27.69101
FBLN5	-2.11856	2.135339	-8.70676	6.96E-17	4.19E-15	27.61998
HROB	1.25599	2.02311	8.68744	8.05E-17	4.81E-15	27.47817
HSD17B6	-1.90784	1.451248	-8.68576	8.15E-17	4.86E-15	27.46583
SPRY1	-1.83969	2.922042	-8.67971	8.52E-17	5.08E-15	27.42146
PTTG1	1.997004	4.192738	8.672025	9.03E-17	5.36E-15	27.36516
MELK	2.10177	3.100259	8.669739	9.18E-17	5.44E-15	27.3484
BLOC1S3	1.094233	3.196037	8.656288	1.02E-16	6.01E-15	27.24993
RGN	-1.09151	0.471989	-8.65515	1.02E-16	6.05E-15	27.24158
FILIP1L	-2.26727	2.433637	-8.64915	1.07E-16	6.31E-15	27.19769
KLF4	-2.32318	3.364989	-8.64517	1.10E-16	6.48E-15	27.16861
TACC2	-1.38138	1.855142	-8.6447	1.11E-16	6.49E-15	27.16517
BUB1	1.625231	2.656955	8.638922	1.16E-16	6.77E-15	27.12294
BDKRB2	-1.72906	1.615475	-8.63543	1.19E-16	6.92E-15	27.09746
HSPB7	-2.62241	1.217427	-8.63542	1.19E-16	6.92E-15	27.09733
FOXM1	2.165812	3.407212	8.627954	1.25E-16	7.31E-15	27.04282
SPEG	-1.5018	0.750658	-8.62541	1.28E-16	7.44E-15	27.02424
CPEB2	-1.15342	1.429387	-8.62511	1.28E-16	7.44E-15	27.02205
ATP5MF	1.095061	5.782407	8.617909	1.35E-16	7.84E-15	26.96951
LINC0086	-1.44425	1.190998	-8.60984	1.43E-16	8.27E-15	26.91071
TPM1	-2.4122	3.744872	-8.60454	1.49E-16	8.59E-15	26.87201

KIF11	1.684476	3.05616	8.604169	1.50E-16	8.60E-15	26.86934
PPP1CA	1.086166	6.453494	8.598483	1.56E-16	8.96E-15	26.82792
PKMYT1	1.640979	2.39872	8.594218	1.61E-16	9.22E-15	26.79686
NUDT5	1.162323	3.904549	8.59393	1.61E-16	9.23E-15	26.79476
MTURN	-1.45667	2.258592	-8.5904	1.66E-16	9.46E-15	26.76908
MEIS1	-1.17718	1.391766	-8.58886	1.68E-16	9.55E-15	26.75785
RAD54L	1.494782	2.105743	8.584045	1.74E-16	9.89E-15	26.72282
LYPLA2	1.167961	5.817567	8.581426	1.77E-16	1.01E-14	26.70377
SLC24A3	-1.93834	1.465971	-8.55809	2.11E-16	1.19E-14	26.5342
MTFR2	1.24929	1.821303	8.557609	2.11E-16	1.19E-14	26.53071
SLC9A9	-1.51477	1.360553	-8.55644	2.13E-16	1.20E-14	26.52222
RRM2	2.18133	3.943881	8.55567	2.14E-16	1.20E-14	26.51663
AP1S1	1.171772	5.473026	8.54804	2.27E-16	1.27E-14	26.46128
KCNH2	-1.67381	0.687729	-8.54668	2.29E-16	1.28E-14	26.4514
CCN1	-3.10601	5.442996	-8.54596	2.30E-16	1.28E-14	26.44619
TIMELES:	1.421824	3.422533	8.544189	2.33E-16	1.30E-14	26.43335
POC1A	1.545754	3.102421	8.534996	2.50E-16	1.38E-14	26.36671
DTYMK	1.286399	3.962673	8.532129	2.55E-16	1.41E-14	26.34594
SNHG1	1.36022	3.883189	8.529759	2.60E-16	1.43E-14	26.32878
CENPF	1.812141	2.830559	8.528694	2.62E-16	1.44E-14	26.32107
MEOX1	-1.39299	0.771838	-8.52681	2.65E-16	1.46E-14	26.30742
NCAPG	1.624632	2.379237	8.525337	2.68E-16	1.47E-14	26.29676
MYL9	-3.46677	5.888652	-8.52387	2.71E-16	1.49E-14	26.28615
KIF23	1.575005	2.471823	8.520842	2.77E-16	1.52E-14	26.26422
NCALD	-1.30659	1.011284	-8.51603	2.87E-16	1.57E-14	26.22941
POLR2G	1.002971	5.187691	8.51013	3.00E-16	1.64E-14	26.18673
ZNF710-A	-1.14119	1.036935	-8.50749	3.06E-16	1.67E-14	26.16762
TMEM22:	1.040357	3.426504	8.504345	3.13E-16	1.70E-14	26.14491
FERMT2	-1.9659	1.997506	-8.50041	3.22E-16	1.75E-14	26.11649
SLC26A6	1.250855	2.143692	8.497355	3.30E-16	1.79E-14	26.09441
CCNF	1.234248	2.561678	8.496917	3.31E-16	1.79E-14	26.09125
BAX	1.084082	5.076005	8.49597	3.33E-16	1.80E-14	26.08441
LMNB1	1.778641	4.129405	8.487395	3.55E-16	1.91E-14	26.0225
PDLIM3	-2.6148	1.85592	-8.48183	3.69E-16	1.98E-14	25.98236
PCP4	-3.9189	1.670941	-8.48057	3.73E-16	2.00E-14	25.97329
STARD13	-1.26687	1.222456	-8.47753	3.81E-16	2.04E-14	25.9513
GSTM5	-1.47202	0.677511	-8.47104	4.00E-16	2.13E-14	25.90452
CACNA1(	-1.08776	0.571225	-8.46636	4.14E-16	2.21E-14	25.87083
SFRP1	-2.65118	1.313626	-8.4571	4.43E-16	2.36E-14	25.80413
NIBAN1	-2.39748	2.427226	-8.45104	4.63E-16	2.46E-14	25.7605
CENPX	1.321695	5.169749	8.445546	4.82E-16	2.56E-14	25.72101
LRCH2	-1.12299	0.626213	-8.44239	4.94E-16	2.60E-14	25.69829
PDIA3	1.042029	7.067705	8.440876	4.99E-16	2.62E-14	25.68743
PFDN6	1.132332	4.128771	8.440687	5.00E-16	2.62E-14	25.68607
ACKR1	-3.19736	2.101819	-8.43853	5.08E-16	2.66E-14	25.67054
RHOJ	-1.46098	1.497004	-8.43197	5.33E-16	2.79E-14	25.6234
RASGRP2	-1.35206	0.789804	-8.42997	5.41E-16	2.82E-14	25.60906
CNTN1	-2.1919	0.980023	-8.42448	5.63E-16	2.93E-14	25.56962
CDC25C	1.261885	1.709853	8.420139	5.81E-16	3.02E-14	25.53848
CLIP3	-2.24511	2.025151	-8.40342	6.56E-16	3.40E-14	25.41861
TMC6	1.50266	3.654797	8.399685	6.75E-16	3.49E-14	25.39182
TMEM35/	-1.79326	0.716279	-8.38934	7.27E-16	3.76E-14	25.31775

FBXW9	1.015187	2.987935	8.378904	7.85E-16	4.05E-14	25.24308
SRPX	-2.85671	2.395142	-8.37724	7.95E-16	4.09E-14	25.2312
SLC35A2	1.263091	4.155378	8.376228	8.00E-16	4.12E-14	25.22395
PRC1	1.739293	3.298351	8.375333	8.06E-16	4.14E-14	25.21755
MEF2C	-1.20354	1.105255	-8.3745	8.11E-16	4.16E-14	25.21163
EBP	1.342602	4.80435	8.364437	8.72E-16	4.45E-14	25.13969
NAA40	1.034179	2.824624	8.364048	8.75E-16	4.46E-14	25.13691
SAMD4A	-1.21035	1.042628	-8.35941	9.05E-16	4.61E-14	25.10381
CHRM3	-1.05947	0.463248	-8.34884	9.77E-16	4.96E-14	25.02835
TRAIP	1.174109	2.019658	8.348135	9.82E-16	4.98E-14	25.02333
MYO19	1.066785	2.343615	8.321833	1.19E-15	5.99E-14	24.83595
DLGAP5	1.80803	2.77411	8.319148	1.21E-15	6.10E-14	24.81685
GINS2	1.557174	2.801756	8.314053	1.26E-15	6.32E-14	24.78061
EMCN	-1.36585	1.210426	-8.30426	1.35E-15	6.78E-14	24.71096
ACTL6A	1.178134	4.270623	8.304027	1.35E-15	6.78E-14	24.70934
BAG2	-1.61033	1.535633	-8.29373	1.46E-15	7.28E-14	24.63617
PLK4	1.202674	1.972827	8.287873	1.52E-15	7.58E-14	24.59464
SBSPON	-2.07088	1.240478	-8.28682	1.53E-15	7.63E-14	24.58718
ZEB2	-1.11363	0.888443	-8.28203	1.59E-15	7.89E-14	24.55318
DDOST	1.027033	6.84463	8.280977	1.60E-15	7.94E-14	24.54572
MIR22HG	-1.6359	2.795981	-8.24556	2.06E-15	1.02E-13	24.29493
ACTA2	-3.37193	5.763108	-8.24221	2.11E-15	1.04E-13	24.27126
ATP6V0B	1.057326	5.407812	8.240891	2.14E-15	1.05E-13	24.26196
UNG	1.114065	4.675698	8.238473	2.17E-15	1.07E-13	24.24488
SPC25	1.523043	2.363099	8.232546	2.27E-15	1.11E-13	24.20301
FGF7	-1.90987	1.018111	-8.2228	2.43E-15	1.19E-13	24.13426
TSC22D1	-1.53795	4.300653	-8.22086	2.47E-15	1.20E-13	24.12054
SLC5A6	1.581381	3.666963	8.220832	2.47E-15	1.20E-13	24.12034
TSPAN18	-1.75047	1.501575	-8.21377	2.60E-15	1.26E-13	24.07053
MFSD3	1.513585	3.772301	8.205903	2.75E-15	1.33E-13	24.0151
ITGA9	-1.2486	1.053543	-8.20434	2.78E-15	1.35E-13	24.00411
ITGA7	-2.05249	1.718333	-8.20297	2.81E-15	1.36E-13	23.99445
IER5L	1.696222	3.255047	8.201027	2.84E-15	1.38E-13	23.98076
PRKAG2	-1.06594	1.897773	-8.20028	2.86E-15	1.38E-13	23.9755
DNAJB4	-1.38488	2.296959	-8.19462	2.98E-15	1.44E-13	23.93569
SAC3D1	1.44049	3.314764	8.19375	3.00E-15	1.44E-13	23.92954
IL6	-2.87543	1.63462	-8.19189	3.04E-15	1.46E-13	23.91643
JPH2	-2.05168	1.210874	-8.19098	3.06E-15	1.47E-13	23.91003
ATF3	-2.904	3.771213	-8.18315	3.23E-15	1.55E-13	23.85496
PPP4C	1.046773	5.889259	8.176321	3.40E-15	1.62E-13	23.80698
FYCO1	-1.30305	2.625979	-8.17364	3.46E-15	1.65E-13	23.78817
MATN2	-2.45501	2.350482	-8.17354	3.46E-15	1.65E-13	23.78745
NAP1L5	-1.18809	1.586863	-8.16686	3.63E-15	1.73E-13	23.74057
TSPYL2	-1.32252	2.629228	-8.16673	3.64E-15	1.73E-13	23.73964
STXBP2	1.283908	3.827943	8.165508	3.67E-15	1.74E-13	23.73104
UBALD2	1.433992	5.941926	8.160576	3.80E-15	1.80E-13	23.69643
NR2C2AP	1.080675	4.2135	8.158305	3.86E-15	1.83E-13	23.68049
SLC39A4	1.677186	3.130704	8.150951	4.07E-15	1.92E-13	23.62893
PALLD	-2.35837	3.550032	-8.14729	4.18E-15	1.97E-13	23.60324
SAPCD2	1.954482	2.857564	8.126986	4.83E-15	2.27E-13	23.4611
ITPA	1.045217	5.082482	8.122025	5.01E-15	2.35E-13	23.4264
KCNK3	-1.26616	0.495894	-8.12187	5.01E-15	2.35E-13	23.42534

NAP1L2	-1.13947	0.78764	-8.12107	5.04E-15	2.36E-13	23.41972
RFC2	1.117121	4.299385	8.119164	5.11E-15	2.39E-13	23.40641
CRYM	-1.57651	0.723678	-8.11546	5.25E-15	2.45E-13	23.38052
EZH2	1.524334	2.779097	8.107937	5.54E-15	2.58E-13	23.32796
COL21A1	-1.0985	0.469187	-8.08964	6.31E-15	2.93E-13	23.2003
CKS1B	1.574876	3.91378	8.088564	6.36E-15	2.95E-13	23.19279
HMMR	1.617564	2.410376	8.08441	6.55E-15	3.04E-13	23.16384
TUBG1	1.123785	4.780566	8.084128	6.56E-15	3.04E-13	23.16187
CLDN5	-1.99244	1.899371	-8.08404	6.56E-15	3.04E-13	23.16129
BOC	-1.49715	0.832451	-8.08376	6.58E-15	3.04E-13	23.15932
COA6	1.060554	4.341273	8.082423	6.64E-15	3.06E-13	23.14999
PRR11	1.68247	2.720092	8.082302	6.65E-15	3.06E-13	23.14915
ILK	-1.18126	1.801986	-8.0799	6.76E-15	3.11E-13	23.13239
KNSTRN	1.248646	2.706456	8.078193	6.84E-15	3.14E-13	23.12053
ATP13A2	1.06489	3.867481	8.077173	6.89E-15	3.16E-13	23.11342
MXRA7	-1.56748	2.611256	-8.07686	6.91E-15	3.17E-13	23.11123
RAB23	-1.53511	1.535836	-8.06843	7.33E-15	3.36E-13	23.0526
KLHL13	-1.08772	0.645297	-8.06749	7.38E-15	3.38E-13	23.04604
PRAC1	-1.88042	0.450253	-8.06152	7.70E-15	3.52E-13	23.00451
C3orf70	-1.37526	0.911085	-8.0588	7.85E-15	3.58E-13	22.98556
CENPU	1.60032	3.052693	8.058503	7.87E-15	3.59E-13	22.98351
DES	-6.07724	4.121053	-8.05546	8.04E-15	3.66E-13	22.96235
DTL	1.415354	2.162121	8.053811	8.14E-15	3.69E-13	22.9509
KIF22	1.341748	4.350332	8.053467	8.16E-15	3.70E-13	22.94851
KAT2A	1.225812	4.672047	8.049238	8.40E-15	3.80E-13	22.91913
PPP1R12A	-1.08785	2.597871	-8.04438	8.70E-15	3.93E-13	22.88538
PCNA	1.561643	6.638447	8.039653	8.99E-15	4.06E-13	22.85258
TCF3	1.060853	4.232405	8.039268	9.02E-15	4.06E-13	22.84991
FCER1A	-1.58864	0.917987	-8.03467	9.32E-15	4.18E-13	22.81799
NFIX	-1.74078	2.660624	-8.02777	9.78E-15	4.38E-13	22.77014
MCM2	1.810928	4.036295	8.020859	1.03E-14	4.59E-13	22.72225
EDEM2	1.009495	4.535093	8.020649	1.03E-14	4.59E-13	22.7208
RTL5	-1.28539	0.849078	-8.01241	1.09E-14	4.86E-13	22.66372
MIIP	1.145396	3.872994	8.008906	1.12E-14	4.95E-13	22.63949
NDC80	1.665719	2.756292	8.007125	1.13E-14	5.01E-13	22.62716
GFUS	1.218641	4.96592	8.005599	1.14E-14	5.05E-13	22.61661
TOMM40	1.093789	4.884919	8.002519	1.17E-14	5.16E-13	22.5953
KPNA2	1.731671	5.553948	7.9998	1.19E-14	5.25E-13	22.57649
MGLL	-1.83628	2.056016	-7.98777	1.30E-14	5.71E-13	22.49334
STMN1	1.891729	5.332823	7.986056	1.31E-14	5.77E-13	22.48151
MIS18A	1.253541	3.648388	7.978368	1.39E-14	6.08E-13	22.42844
RCAN1	-1.79123	2.72384	-7.97485	1.42E-14	6.22E-13	22.40415
ANTXR2	-1.80231	2.26237	-7.96422	1.53E-14	6.68E-13	22.3309
FANCD2	1.176762	2.121056	7.962719	1.55E-14	6.74E-13	22.32052
FKBPL	1.092986	3.141936	7.960705	1.57E-14	6.83E-13	22.30664
WDR4	1.035723	3.077988	7.958844	1.59E-14	6.91E-13	22.29382
CDCA4	1.426042	3.711316	7.957948	1.60E-14	6.94E-13	22.28765
FEN1	1.653234	4.450772	7.955373	1.63E-14	7.06E-13	22.26991
XRCC2	1.046461	1.366611	7.953404	1.65E-14	7.14E-13	22.25635
CENPO	1.003258	1.888572	7.952431	1.66E-14	7.17E-13	22.24966
C2CD4B	-1.59262	0.911831	-7.95062	1.69E-14	7.25E-13	22.23715
DENND2A	-1.39086	1.244699	-7.95029	1.69E-14	7.25E-13	22.23488

OCIAD2	1.46528	4.844291	7.939691	1.82E-14	7.80E-13	22.162
AKAP12	-2.20731	2.149234	-7.93656	1.86E-14	7.96E-13	22.1405
FAM83H	1.775625	5.125649	7.921915	2.06E-14	8.76E-13	22.03986
DEPDC1B	1.344564	1.890548	7.915115	2.16E-14	9.17E-13	21.99319
A2M	-2.4985	5.635981	-7.91098	2.23E-14	9.42E-13	21.96485
GSS	1.015225	5.518196	7.909639	2.25E-14	9.50E-13	21.95563
ECE2	1.016354	1.692932	7.907293	2.28E-14	9.65E-13	21.93955
PDE4B	-1.30085	0.972753	-7.90682	2.29E-14	9.67E-13	21.93631
SLC37A4	1.064093	2.59249	7.893685	2.51E-14	1.06E-12	21.84632
RPS6KA2	-1.34992	1.962634	-7.89332	2.52E-14	1.06E-12	21.84384
ADGRA2	-1.88741	2.096191	-7.89054	2.57E-14	1.08E-12	21.82479
MRPS26	1.127544	5.940251	7.885751	2.66E-14	1.11E-12	21.79201
TAMALIN	-1.46108	1.798121	-7.88268	2.71E-14	1.14E-12	21.77099
TSEN54	1.010934	3.932316	7.882575	2.72E-14	1.14E-12	21.77028
ATAD3A	1.095977	3.909714	7.881871	2.73E-14	1.14E-12	21.76547
PTGIS	-3.01236	1.89461	-7.87862	2.79E-14	1.16E-12	21.7432
UBE2S	1.580393	4.091578	7.874993	2.86E-14	1.19E-12	21.71844
SGO1	1.257364	1.691911	7.873905	2.89E-14	1.20E-12	21.711
CNPY2	1.039071	3.604727	7.873409	2.90E-14	1.20E-12	21.70761
BOP1	1.303642	4.639791	7.872228	2.92E-14	1.21E-12	21.69954
FAM110D	-1.06009	0.961013	-7.86965	2.97E-14	1.23E-12	21.6819
PPP1R35	1.225498	4.256565	7.868067	3.01E-14	1.24E-12	21.67112
EME1	1.103862	1.396301	7.860754	3.16E-14	1.31E-12	21.62118
FANCA	1.11061	1.6272	7.855766	3.27E-14	1.35E-12	21.58714
WWTR1	-1.46531	2.719748	-7.85436	3.31E-14	1.36E-12	21.57754
ZDHHC12	1.301299	4.532276	7.853266	3.33E-14	1.37E-12	21.57009
STIL	1.145794	1.617641	7.852155	3.36E-14	1.38E-12	21.56251
TENT5B	-2.31747	1.658841	-7.84303	3.58E-14	1.46E-12	21.50029
TRIP13	1.824196	2.991822	7.843014	3.58E-14	1.46E-12	21.5002
H2AW	2.066335	4.029769	7.840281	3.65E-14	1.49E-12	21.48158
BID	1.177344	3.730767	7.83854	3.69E-14	1.51E-12	21.46971
FOXN3	-1.11226	2.416771	-7.8343	3.80E-14	1.55E-12	21.44081
CKAP2L	1.460951	1.96075	7.822546	4.13E-14	1.68E-12	21.36086
OIP5	1.349046	2.039804	7.820477	4.19E-14	1.70E-12	21.34679
MCM4	1.524598	4.386469	7.818657	4.24E-14	1.72E-12	21.33442
EGR2	-1.83298	1.553618	-7.81499	4.35E-14	1.76E-12	21.30948
PYCR3	1.189109	3.035413	7.813822	4.38E-14	1.77E-12	21.30155
NPR2	-1.05623	1.328162	-7.80662	4.61E-14	1.85E-12	21.25265
MANF	1.149513	5.456234	7.804112	4.69E-14	1.88E-12	21.2356
BMERB1	-1.73352	1.912743	-7.80127	4.78E-14	1.92E-12	21.21632
SLC19A1	1.153981	1.957637	7.799425	4.84E-14	1.94E-12	21.20379
FGFR1	-1.97501	1.748279	-7.79869	4.87E-14	1.95E-12	21.19883
SSPN	-1.36449	1.793629	-7.79761	4.91E-14	1.96E-12	21.19145
AUNIP	1.258059	1.835831	7.795187	4.99E-14	1.99E-12	21.17503
BRMS1	1.08768	4.850131	7.793313	5.05E-14	2.01E-12	21.16233
RNF122	-1.34428	2.537621	-7.79209	5.10E-14	2.03E-12	21.15402
CLN6	1.051078	3.808161	7.780479	5.52E-14	2.19E-12	21.07534
PKIG	-1.70301	3.841279	-7.7727	5.83E-14	2.31E-12	21.02267
SPON1	-2.56133	2.129168	-7.77163	5.87E-14	2.32E-12	21.0154
LDB2	-1.13096	1.277204	-7.76153	6.30E-14	2.48E-12	20.9471
FZD7	-1.68361	2.629741	-7.75904	6.41E-14	2.51E-12	20.93025
SKP2	1.540131	3.522523	7.751836	6.73E-14	2.63E-12	20.88159

LRRC4B	-1.05849	0.559091	-7.74775	6.92E-14	2.70E-12	20.85399
SNHG12	1.214833	2.95253	7.741726	7.22E-14	2.81E-12	20.81333
EXO1	1.329596	1.868552	7.738157	7.40E-14	2.88E-12	20.78925
HSPB8	-3.21312	3.550598	-7.73626	7.50E-14	2.91E-12	20.77645
SNORD99	1.75296	3.21848	7.714271	8.72E-14	3.37E-12	20.6283
PNKP	1.021994	3.698028	7.706276	9.21E-14	3.55E-12	20.57451
DSN1	1.174554	3.46566	7.695613	9.91E-14	3.81E-12	20.50284
RSPO3	-1.4048	0.703259	-7.69372	1.00E-13	3.85E-12	20.49012
GNG11	-1.64575	2.591437	-7.69008	1.03E-13	3.95E-12	20.46567
CBX8	1.020562	1.948678	7.685743	1.06E-13	4.06E-12	20.43656
TTLL7	-1.04752	0.766832	-7.6837	1.08E-13	4.11E-12	20.42284
SLC50A1	1.342096	5.003837	7.683425	1.08E-13	4.12E-12	20.42101
OXER1	-1.14681	0.96386	-7.68319	1.08E-13	4.12E-12	20.41946
RANBP1	1.134274	4.435766	7.677961	1.12E-13	4.26E-12	20.38436
GPM6B	-1.0078	0.866165	-7.67297	1.16E-13	4.41E-12	20.35087
RASL11A	-1.82937	1.961702	-7.66989	1.18E-13	4.50E-12	20.33023
PLA2G5	-1.26172	0.633113	-7.66717	1.20E-13	4.57E-12	20.31205
ORC1	1.339581	2.001584	7.662499	1.24E-13	4.72E-12	20.28074
SEMA3G	-1.1595	1.245654	-7.66181	1.25E-13	4.73E-12	20.27613
RFC5	1.041478	3.097866	7.659182	1.27E-13	4.81E-12	20.25853
ARHGAP(	-1.32824	0.72782	-7.65812	1.28E-13	4.84E-12	20.25144
DHCR7	1.525806	4.11665	7.655191	1.31E-13	4.93E-12	20.23182
HIPK3	-1.13793	3.059119	-7.65342	1.32E-13	4.98E-12	20.21996
SIRT7	1.016207	3.037572	7.644755	1.40E-13	5.27E-12	20.16203
NUDT1	1.217454	3.74075	7.643729	1.41E-13	5.30E-12	20.15517
CDC25A	1.243628	2.046009	7.639081	1.46E-13	5.46E-12	20.12411
GYPC	-1.9894	1.989599	-7.63719	1.48E-13	5.53E-12	20.11149
CNRIP1	-1.39315	1.295455	-7.63673	1.48E-13	5.54E-12	20.10843
SMYD2	1.041601	3.389769	7.636252	1.49E-13	5.56E-12	20.10522
CENPH	1.206898	2.752345	7.635623	1.49E-13	5.57E-12	20.10101
MYADM	-2.54221	4.089203	-7.63372	1.51E-13	5.64E-12	20.08832
CYTL1	-1.60892	0.925063	-7.63185	1.53E-13	5.70E-12	20.07581
TPSD1	-1.29512	0.555422	-7.62609	1.60E-13	5.91E-12	20.03737
TGFBR3	-1.93727	2.247931	-7.62526	1.60E-13	5.94E-12	20.03183
KNTC1	1.087788	1.946281	7.622163	1.64E-13	6.06E-12	20.01118
ABL1	-1.10787	3.760315	-7.60902	1.79E-13	6.60E-12	19.92358
CHEK1	1.128969	2.118003	7.602324	1.88E-13	6.90E-12	19.879
SNRPF	1.109311	4.537909	7.602297	1.88E-13	6.90E-12	19.87881
HAS1	-1.55019	0.573105	-7.59816	1.93E-13	7.05E-12	19.85131
MGAT4B	1.048592	4.577606	7.594671	1.98E-13	7.21E-12	19.82807
RAC3	1.913334	3.002628	7.593931	1.99E-13	7.24E-12	19.82315
JAZF1	-1.30649	1.641313	-7.58837	2.06E-13	7.50E-12	19.78619
OSBPL10	-1.18624	1.502097	-7.58492	2.11E-13	7.66E-12	19.76323
ENTPD6	1.013361	4.102595	7.58275	2.14E-13	7.76E-12	19.74883
TPSAB1	-2.40567	2.093823	-7.58206	2.15E-13	7.79E-12	19.74427
CPEB4	-1.04676	1.668018	-7.57985	2.19E-13	7.90E-12	19.72957
RGS5	-2.29053	3.359037	-7.57747	2.22E-13	8.03E-12	19.71377
PSMB3	1.073955	7.039221	7.574272	2.27E-13	8.19E-12	19.69253
ZMYND1(	1.028663	3.739587	7.570169	2.33E-13	8.41E-12	19.6653
DDX11	1.05845	2.375959	7.568256	2.36E-13	8.51E-12	19.65261
EMP1	-2.2895	3.810755	-7.5665	2.39E-13	8.58E-12	19.64095
DCHS1	-1.35866	1.28646	-7.56024	2.50E-13	8.92E-12	19.59947

NDC1	1.034122	3.309665	7.559587	2.51E-13	8.94E-12	19.59513
GGCT	1.295477	5.43477	7.556006	2.57E-13	9.14E-12	19.5714
YIF1B	1.185441	3.929155	7.550939	2.66E-13	9.44E-12	19.53784
RHBDF2	1.198603	2.941734	7.545253	2.76E-13	9.80E-12	19.5002
TAGLN	-3.32113	5.478645	-7.545	2.77E-13	9.80E-12	19.49855
PRKAR2E	-1.67333	1.507159	-7.54492	2.77E-13	9.80E-12	19.49797
STK40	-1.02288	3.232086	-7.52701	3.13E-13	1.10E-11	19.37959
ARHGEF2	-1.74459	2.156535	-7.52309	3.21E-13	1.13E-11	19.35365
MRM1	1.086729	2.971929	7.518069	3.32E-13	1.17E-11	19.32053
BCL2	-1.05866	1.072668	-7.51798	3.32E-13	1.17E-11	19.31994
SYNGR2	1.214083	5.98623	7.50286	3.68E-13	1.28E-11	19.22023
MITF	-1.02222	0.917283	-7.5002	3.75E-13	1.31E-11	19.20268
CCDC167	1.209279	4.870244	7.487054	4.09E-13	1.42E-11	19.11615
NPR1	-1.36307	1.165621	-7.48282	4.21E-13	1.46E-11	19.08833
IL6ST	-1.43034	3.091956	-7.4822	4.23E-13	1.46E-11	19.08422
CCDC80	-2.55452	2.186657	-7.47562	4.42E-13	1.52E-11	19.04095
CILP	-2.50938	1.154017	-7.47502	4.44E-13	1.52E-11	19.03702
PAQR4	1.534415	2.518073	7.472338	4.52E-13	1.55E-11	19.0194
SDF2L1	1.340241	4.686524	7.471702	4.54E-13	1.56E-11	19.01523
KIAA0513	-1.1576	1.488914	-7.46372	4.79E-13	1.64E-11	18.96282
SPINT1	2.169123	6.381397	7.462128	4.84E-13	1.65E-11	18.95237
TUB	-1.0468	0.731448	-7.45828	4.96E-13	1.69E-11	18.92713
PGP	1.024176	3.348684	7.454912	5.08E-13	1.73E-11	18.90503
PDRG1	1.080374	4.007525	7.451653	5.19E-13	1.76E-11	18.88366
ARHGAP1	1.263353	2.211914	7.451192	5.21E-13	1.77E-11	18.88064
RASSF7	1.717607	4.562495	7.450314	5.24E-13	1.78E-11	18.87488
BVES	-1.31121	0.792647	-7.44251	5.52E-13	1.87E-11	18.82372
PPM1L	-1.26779	1.430539	-7.44204	5.54E-13	1.87E-11	18.82068
PBK	1.619479	2.685112	7.438663	5.66E-13	1.91E-11	18.79856
CHAF1A	1.100932	3.438032	7.414277	6.67E-13	2.24E-11	18.63912
DCN	-3.02642	3.584418	-7.41177	6.78E-13	2.27E-11	18.62274
RFC4	1.318795	3.320175	7.411768	6.78E-13	2.27E-11	18.62274
RUVBL1	1.009183	3.506763	7.408721	6.92E-13	2.31E-11	18.60284
TPM3	1.014823	5.514946	7.406814	7.01E-13	2.34E-11	18.5904
GNAZ	-1.27901	0.770728	-7.4047	7.11E-13	2.37E-11	18.57661
LIFR	-1.31184	1.018482	-7.40453	7.11E-13	2.37E-11	18.5755
LPAR2	1.380396	3.663564	7.404011	7.14E-13	2.37E-11	18.57212
CCNE1	1.775273	3.157667	7.403666	7.16E-13	2.38E-11	18.56986
TIPARP	-1.50527	3.066466	-7.40247	7.21E-13	2.39E-11	18.56207
MYDGF	1.006786	5.920598	7.393935	7.64E-13	2.53E-11	18.50642
MAPK13	1.429714	4.082101	7.393064	7.68E-13	2.54E-11	18.50075
GALNT15	-1.15929	0.619918	-7.3803	8.36E-13	2.75E-11	18.4176
E2F1	1.8631	3.547781	7.378451	8.47E-13	2.79E-11	18.4056
THRA	-1.1902	2.236362	-7.37465	8.68E-13	2.85E-11	18.38087
KIF15	1.262118	1.871448	7.373977	8.72E-13	2.86E-11	18.3765
NT5DC3	-1.06276	1.177364	-7.36946	8.99E-13	2.94E-11	18.34714
RAD51AP	1.481585	2.435203	7.358629	9.66E-13	3.16E-11	18.27678
PSRC1	1.26341	2.356669	7.358548	9.66E-13	3.16E-11	18.27626
TWIST2	-1.5574	0.977654	-7.35098	1.02E-12	3.32E-11	18.22716
CDCA2	1.223397	1.729277	7.348815	1.03E-12	3.37E-11	18.21309
APBB1	-1.54994	1.561922	-7.34765	1.04E-12	3.39E-11	18.20551
RASSF3	-1.34344	3.255503	-7.34437	1.06E-12	3.45E-11	18.18425

CENPK	1.100007	1.618257	7.343721	1.07E-12	3.46E-11	18.18007
NCBP2AS	1.000047	4.738351	7.342193	1.08E-12	3.49E-11	18.17016
PABPC1L	1.76169	2.992523	7.33787	1.11E-12	3.59E-11	18.14215
ECT2	1.548281	3.049489	7.332006	1.15E-12	3.72E-11	18.10418
LAMTOR:	1.083832	5.360689	7.32854	1.18E-12	3.81E-11	18.08174
EDNRB	-1.56489	1.445059	-7.32835	1.18E-12	3.81E-11	18.08051
KRT18	2.525858	7.790056	7.318223	1.26E-12	4.06E-11	18.01501
MAD2L1	1.356166	2.427543	7.309068	1.34E-12	4.31E-11	17.95585
MYCT1	-1.11091	1.299687	-7.30847	1.35E-12	4.32E-11	17.95202
SVIL	-1.74286	3.283345	-7.30481	1.38E-12	4.41E-11	17.92837
KCNE4	-1.52586	1.157113	-7.29877	1.44E-12	4.59E-11	17.88936
CXCL12	-2.30514	2.020993	-7.29608	1.46E-12	4.66E-11	17.87201
MRPL12	1.19654	5.155023	7.295889	1.46E-12	4.66E-11	17.87079
MCM7	1.194671	5.357561	7.286417	1.56E-12	4.95E-11	17.80973
H2BC5	2.08406	4.126982	7.283768	1.59E-12	5.03E-11	17.79267
POLE2	1.006369	1.692573	7.277783	1.65E-12	5.22E-11	17.75413
CKLF	1.064711	3.333608	7.27187	1.71E-12	5.41E-11	17.71608
DAAM2	-1.06497	0.897927	-7.26945	1.74E-12	5.50E-11	17.70051
MEIS2	-1.05646	1.313001	-7.26544	1.79E-12	5.63E-11	17.67474
PIMREG	1.602896	2.25378	7.263967	1.81E-12	5.69E-11	17.66527
TLN1	-1.2647	4.861345	-7.26344	1.81E-12	5.70E-11	17.66185
CEP131	1.167329	3.247809	7.260928	1.84E-12	5.79E-11	17.64573
ACYP1	1.005056	2.594112	7.260806	1.84E-12	5.79E-11	17.64495
TRAF2	1.027277	3.339055	7.258922	1.87E-12	5.85E-11	17.63285
FAM149A	-1.18672	0.860521	-7.25603	1.90E-12	5.96E-11	17.61428
RGS19	1.119051	3.481554	7.249472	1.99E-12	6.20E-11	17.57217
DMPK	-1.24066	3.214967	-7.24439	2.05E-12	6.40E-11	17.53954
SQLE	1.647687	4.10528	7.24317	2.07E-12	6.45E-11	17.53174
GAS7	-1.51176	1.287955	-7.24282	2.08E-12	6.46E-11	17.52947
HMGA1	1.719109	7.003826	7.241519	2.09E-12	6.51E-11	17.52115
MCM10	1.294215	1.819479	7.234263	2.20E-12	6.81E-11	17.47465
PFKFB4	1.309299	1.95082	7.232725	2.22E-12	6.86E-11	17.4648
C4orf48	2.007468	3.339715	7.223995	2.35E-12	7.26E-11	17.4089
C21orf58	1.161705	1.584948	7.223764	2.35E-12	7.26E-11	17.40742
CDKN3	1.568172	2.852833	7.214003	2.51E-12	7.71E-11	17.34498
ABRACL	1.298002	5.36755	7.210852	2.56E-12	7.84E-11	17.32484
TPSB2	-2.32388	2.128321	-7.20732	2.62E-12	8.02E-11	17.30225
PCDH18	-1.14896	1.042818	-7.20648	2.63E-12	8.05E-11	17.29693
SLC25A2:	-1.59601	2.857656	-7.20612	2.64E-12	8.07E-11	17.29462
CYBRD1	-2.11609	3.021387	-7.20473	2.66E-12	8.13E-11	17.28574
PKDCC	-2.02082	1.205248	-7.20327	2.69E-12	8.20E-11	17.27638
UQCC3	1.117504	3.655325	7.19884	2.77E-12	8.43E-11	17.24812
CRISPLD:	-2.04775	2.698265	-7.1941	2.86E-12	8.68E-11	17.21785
TSHZ3	-1.37464	1.24367	-7.19276	2.88E-12	8.75E-11	17.20933
SLC39A11	1.134224	3.404425	7.189474	2.94E-12	8.93E-11	17.18838
MYZAP	-1.70227	1.878492	-7.16874	3.37E-12	1.02E-10	17.05632
HDGF	1.001694	6.752785	7.165491	3.44E-12	1.04E-10	17.03566
MTHFD1I	1.250017	2.911991	7.161128	3.54E-12	1.06E-10	17.00792
BIN1	-1.8432	2.082527	-7.15657	3.65E-12	1.09E-10	16.97896
MRPS12	1.172009	4.670541	7.145826	3.91E-12	1.17E-10	16.91074
CCN2	-2.92433	5.160115	-7.14431	3.95E-12	1.18E-10	16.90115
CENPE	1.118972	1.73393	7.142691	3.99E-12	1.19E-10	16.89085

FANCG	1.109954	3.099089	7.142226	4.00E-12	1.19E-10	16.88789
PODN	-2.25137	2.209239	-7.13957	4.07E-12	1.21E-10	16.87107
SLC25A22	1.011029	3.14809	7.121744	4.57E-12	1.35E-10	16.75812
DUSP8	-1.13422	1.101053	-7.12048	4.61E-12	1.36E-10	16.75014
ASPM	1.290231	1.770438	7.118364	4.67E-12	1.38E-10	16.73674
DONSON	1.184873	2.822638	7.116967	4.72E-12	1.39E-10	16.72789
MRPL14	1.032655	5.997946	7.116785	4.72E-12	1.39E-10	16.72675
SOCS3	-2.23492	5.170954	-7.11091	4.91E-12	1.44E-10	16.68962
EIF4EBP1	1.626731	5.780916	7.109079	4.96E-12	1.45E-10	16.67802
CKAP2	1.25088	3.2131	7.10583	5.07E-12	1.48E-10	16.65749
PIF1	1.019176	1.539838	7.103984	5.13E-12	1.50E-10	16.64583
S1PR1	-1.52531	2.169236	-7.10295	5.17E-12	1.51E-10	16.63928
LMNB2	1.214991	4.575374	7.100142	5.26E-12	1.53E-10	16.62157
EML1	-1.19773	1.153365	-7.08219	5.91E-12	1.71E-10	16.50834
GFRA3	-1.34902	0.726732	-7.07948	6.01E-12	1.74E-10	16.49124
PDE7B	-1.11958	0.922068	-7.07452	6.21E-12	1.79E-10	16.46003
VAMP8	1.328982	7.280097	7.070946	6.35E-12	1.83E-10	16.43753
SHCBP1	1.273657	2.133541	7.07005	6.39E-12	1.84E-10	16.43189
PDXK	1.127988	4.040793	7.06411	6.64E-12	1.91E-10	16.39452
PLEKHJ1	1.014164	3.904114	7.063149	6.68E-12	1.92E-10	16.38848
CPA3	-2.17205	1.794543	-7.05808	6.90E-12	1.98E-10	16.35662
BRI3BP	1.048684	2.417632	7.052161	7.17E-12	2.06E-10	16.31943
LSR	1.972122	6.44354	7.04825	7.35E-12	2.10E-10	16.29488
TPM2	-2.62942	5.169678	-7.04546	7.48E-12	2.14E-10	16.27737
MIR4653	1.751701	2.761779	7.029205	8.31E-12	2.37E-10	16.17545
NR2F1	-1.61859	1.893316	-7.02915	8.31E-12	2.37E-10	16.17512
TSPAN2	-1.83572	1.684489	-7.01884	8.88E-12	2.52E-10	16.11056
FLNA	-2.42193	6.127841	-7.01511	9.09E-12	2.58E-10	16.08721
SLC25A4	-1.09839	2.639562	-6.99731	1.02E-11	2.88E-10	15.97602
CPXM2	-2.02714	1.612633	-6.99543	1.03E-11	2.91E-10	15.9643
CASP6	1.023648	3.75235	6.995128	1.03E-11	2.91E-10	15.9624
SNORD10	1.931626	4.464544	6.99508	1.03E-11	2.91E-10	15.9621
LPAR1	-1.39471	2.083983	-6.98975	1.07E-11	3.00E-10	15.92882
CHAF1B	1.17486	2.137678	6.975848	1.17E-11	3.27E-10	15.84222
FAM43A	-1.47256	2.002634	-6.96848	1.23E-11	3.42E-10	15.79636
INPP5A	-1.00546	3.101239	-6.9588	1.30E-11	3.62E-10	15.73616
TGFB1I1	-1.66809	2.576029	-6.95629	1.32E-11	3.67E-10	15.72057
ARID5B	-1.27115	2.969254	-6.95112	1.37E-11	3.79E-10	15.68845
CKS1BP1	-1.14276	0.750749	-6.94331	1.44E-11	3.98E-10	15.64001
DEPDC1	1.248203	1.77092	6.941924	1.45E-11	4.01E-10	15.6314
PDGFD	-1.37351	1.398273	-6.94	1.47E-11	4.05E-10	15.61947
HBEGF	-1.97491	3.195051	-6.93701	1.50E-11	4.12E-10	15.60096
RPA3	1.01474	3.237674	6.930947	1.56E-11	4.28E-10	15.56336
KHDC4	1.102604	3.265102	6.93012	1.56E-11	4.30E-10	15.55824
PLA2G15	1.002805	3.073452	6.93003	1.57E-11	4.30E-10	15.55768
AP2S1	1.01297	5.942886	6.923806	1.63E-11	4.46E-10	15.51915
TEAD1	-1.2014	2.619198	-6.9196	1.67E-11	4.57E-10	15.49311
COLEC12	-1.6609	1.170428	-6.91662	1.70E-11	4.65E-10	15.47469
CDO1	-1.09306	0.508751	-6.90458	1.84E-11	5.00E-10	15.40029
PLSCR4	-1.27152	1.438316	-6.89183	1.99E-11	5.40E-10	15.32159
ANLN	1.741062	3.219001	6.888352	2.04E-11	5.51E-10	15.30016
ARID5A	-1.29571	2.970966	-6.88127	2.13E-11	5.75E-10	15.25654

ST14	1.883392	5.935496	6.87641	2.20E-11	5.92E-10	15.2266
JUN	-1.88053	5.778738	-6.87321	2.24E-11	6.02E-10	15.20691
FBLN2	-2.522	3.105333	-6.86153	2.41E-11	6.45E-10	15.13511
TBX4	-1.16388	0.556342	-6.85721	2.48E-11	6.61E-10	15.10854
HSPA2	-1.56969	1.980242	-6.85373	2.54E-11	6.75E-10	15.08716
BTG2	-2.24632	6.169699	-6.85065	2.59E-11	6.87E-10	15.06826
EDNRA	-1.45829	1.666765	-6.84286	2.72E-11	7.18E-10	15.02048
CDC7	1.158951	2.226205	6.83973	2.77E-11	7.32E-10	15.00129
LSM7	1.056894	5.134321	6.834972	2.85E-11	7.52E-10	14.97213
ADARB1	-1.34009	1.665022	-6.82622	3.02E-11	7.92E-10	14.91854
C8orf88	-1.5375	1.007792	-6.82568	3.03E-11	7.94E-10	14.91523
TYMS	1.660592	3.967537	6.813789	3.26E-11	8.51E-10	14.84253
NCS1	-1.43919	3.173245	-6.81278	3.28E-11	8.55E-10	14.83638
MCM5	1.153209	3.728775	6.810974	3.32E-11	8.64E-10	14.82533
DARS2	1.107418	3.292464	6.810015	3.34E-11	8.68E-10	14.81947
CAP2	-1.39474	1.600265	-6.80772	3.39E-11	8.79E-10	14.80546
CYGB	-1.61663	2.754817	-6.80174	3.52E-11	9.11E-10	14.76898
PPP1R16A	1.063545	2.722886	6.793192	3.71E-11	9.58E-10	14.71682
EPHX2	-1.39043	1.549321	-6.79162	3.75E-11	9.65E-10	14.70723
EVA1C	-1.54921	1.713645	-6.78733	3.85E-11	9.89E-10	14.6811
PARVA	-1.07653	3.004038	-6.78276	3.96E-11	1.02E-09	14.65323
CYYR1	-1.10597	1.73348	-6.7795	4.04E-11	1.04E-09	14.63342
PLXNA1	1.19844	3.633347	6.77286	4.21E-11	1.08E-09	14.59302
PDGFRA	-1.51727	1.578498	-6.76964	4.30E-11	1.10E-09	14.57345
ADCK5	1.038991	2.935678	6.763539	4.46E-11	1.14E-09	14.53638
SH3D19	-1.00674	2.656363	-6.75983	4.57E-11	1.16E-09	14.51383
CALD1	-2.17365	4.017235	-6.74514	5.00E-11	1.27E-09	14.42476
GALE	1.216262	4.148386	6.744867	5.01E-11	1.27E-09	14.42307
GADD45B	-1.85511	4.212198	-6.73423	5.36E-11	1.35E-09	14.35865
MAFF	-1.45607	3.393453	-6.73145	5.45E-11	1.38E-09	14.34183
ANXA6	-2.14325	3.641565	-6.72751	5.58E-11	1.41E-09	14.318
LATS2	-1.0764	2.097016	-6.7262	5.63E-11	1.42E-09	14.31006
SOX4	1.70973	4.976416	6.719356	5.87E-11	1.47E-09	14.26869
RMI2	1.442302	3.203126	6.707297	6.33E-11	1.58E-09	14.19587
PYCR1	2.134897	3.8382	6.706186	6.37E-11	1.59E-09	14.18917
MARCKS	1.753572	6.939194	6.705195	6.41E-11	1.60E-09	14.18319
PCK2	1.181625	3.859803	6.702437	6.52E-11	1.63E-09	14.16656
TCEAL1	-1.16453	3.527133	-6.70166	6.56E-11	1.63E-09	14.16189
RACGAP1	1.33281	3.410422	6.695257	6.82E-11	1.70E-09	14.12328
TSPAN11	-1.02145	0.818379	-6.69195	6.96E-11	1.73E-09	14.10338
KIF14	1.043911	1.418595	6.689423	7.07E-11	1.76E-09	14.08814
KANK1	-1.30902	2.403177	-6.68719	7.17E-11	1.78E-09	14.0747
EXOSC4	1.079621	4.496812	6.681581	7.42E-11	1.84E-09	14.04095
RASD1	-2.01424	1.942362	-6.67837	7.57E-11	1.87E-09	14.02166
ESM1	1.888395	2.067937	6.677945	7.59E-11	1.87E-09	14.01908
VCL	-1.2101	4.140267	-6.67713	7.63E-11	1.88E-09	14.01418
WDHD1	1.002057	1.870913	6.673369	7.81E-11	1.92E-09	13.99158
CKB	-2.43765	4.303702	-6.673	7.83E-11	1.92E-09	13.98936
TMEM88	-1.24799	2.061738	-6.66861	8.04E-11	1.97E-09	13.96299
KDF1	1.527995	3.924945	6.664228	8.26E-11	2.03E-09	13.93668
CIP2A	1.076381	1.921727	6.663437	8.30E-11	2.03E-09	13.93193
TUBB	1.011877	8.134271	6.655329	8.73E-11	2.13E-09	13.88329

PLIN4	-2.06843	2.082602	-6.64618	9.23E-11	2.25E-09	13.82848
CSF3	-2.2334	1.002357	-6.63832	9.69E-11	2.35E-09	13.78144
PTGDS	-2.73968	2.904213	-6.63349	9.98E-11	2.42E-09	13.75253
MEDAG	-1.86459	1.573535	-6.61649	1.11E-10	2.67E-09	13.65099
TMEM16(	1.256847	3.758671	6.613512	1.13E-10	2.72E-09	13.6332
CACNA2I	-1.18184	0.826444	-6.60654	1.18E-10	2.82E-09	13.59165
TLCD1	1.566879	3.829311	6.603359	1.20E-10	2.87E-09	13.57267
SYNPO	-1.37637	2.71893	-6.60326	1.20E-10	2.87E-09	13.57207
SLC12A8	1.319088	1.972565	6.602671	1.21E-10	2.88E-09	13.56857
ROMO1	1.087749	6.812541	6.602002	1.21E-10	2.89E-09	13.56459
ALDH1B1	-1.61744	3.861209	-6.59517	1.26E-10	3.01E-09	13.52389
DYNC2I2	1.424066	5.078555	6.591772	1.29E-10	3.06E-09	13.50369
RBPMS	-1.44316	3.006936	-6.57983	1.39E-10	3.29E-09	13.4327
ETV4	1.929221	3.011006	6.578995	1.39E-10	3.30E-09	13.42773
KIF18A	1.004825	1.431209	6.577149	1.41E-10	3.33E-09	13.41676
FAM111B	1.452054	2.142206	6.567041	1.50E-10	3.53E-09	13.35678
B4GALT3	1.16556	4.362429	6.56061	1.56E-10	3.66E-09	13.31865
NFIC	-1.05363	3.179963	-6.55741	1.59E-10	3.73E-09	13.29967
TENT5A	-1.14903	2.305911	-6.54619	1.70E-10	3.98E-09	13.23324
DLC1	-1.09843	1.4439	-6.54065	1.76E-10	4.10E-09	13.20052
CYS1	-1.01319	0.75458	-6.53859	1.78E-10	4.15E-09	13.18833
C1QTNF6	1.684877	2.754179	6.535825	1.81E-10	4.22E-09	13.17199
PAXX	1.110147	4.893466	6.529501	1.88E-10	4.37E-09	13.13464
KLF6	-1.3441	4.960493	-6.52846	1.89E-10	4.40E-09	13.12852
TNNT2	-1.07745	0.637391	-6.51523	2.05E-10	4.74E-09	13.05047
ABLIM1	-1.41915	3.258825	-6.51359	2.07E-10	4.79E-09	13.04081
ELN	-2.17214	2.230874	-6.51326	2.08E-10	4.79E-09	13.03885
ARL6IP1	1.103095	6.214538	6.510901	2.11E-10	4.85E-09	13.02496
NFATC4	-1.23953	1.888651	-6.50703	2.16E-10	4.96E-09	13.00215
SYT11	-1.35472	1.572402	-6.50522	2.18E-10	5.01E-09	12.99154
WEE1	-1.12148	2.762244	-6.50462	2.19E-10	5.03E-09	12.98799
COMTD1	1.369844	3.716235	6.48936	2.40E-10	5.44E-09	12.89826
S100A11	1.498409	10.67599	6.48901	2.41E-10	5.45E-09	12.8962
SSC5D	-1.49493	1.250422	-6.48692	2.44E-10	5.51E-09	12.88393
RUSC2	-1.17394	2.16458	-6.48493	2.47E-10	5.57E-09	12.87226
RPS6KA1	1.07284	3.448794	6.477368	2.58E-10	5.81E-09	12.82787
C1orf21	-1.1231	2.435904	-6.47642	2.60E-10	5.84E-09	12.82228
SELE	-1.78797	1.166225	-6.47224	2.66E-10	5.97E-09	12.79778
REEP4	1.117963	4.854402	6.471758	2.67E-10	5.98E-09	12.79498
CCL2	-2.24953	3.003959	-6.46283	2.82E-10	6.29E-09	12.7427
MFAP5	-2.11045	1.380109	-6.44394	3.16E-10	6.97E-09	12.63225
C1orf53	1.100286	2.209312	6.435882	3.31E-10	7.30E-09	12.5852
MFSD10	1.039631	5.330828	6.435407	3.32E-10	7.31E-09	12.58242
JUNB	-1.40333	7.495981	-6.42968	3.44E-10	7.56E-09	12.54905
ESPL1	1.137861	1.937277	6.428794	3.46E-10	7.59E-09	12.54386
PCGF2	1.049601	4.113268	6.426768	3.50E-10	7.67E-09	12.53205
CNKSR1	1.411039	3.483989	6.425184	3.53E-10	7.74E-09	12.52282
RAD9A	1.019484	2.922779	6.42441	3.55E-10	7.77E-09	12.51831
PARM1	-1.93607	2.543901	-6.42401	3.56E-10	7.78E-09	12.51599
SMIM10	-1.28045	1.873668	-6.41465	3.76E-10	8.21E-09	12.46152
OLFML3	-2.23793	2.840612	-6.41067	3.85E-10	8.39E-09	12.43837
UCK2	1.008355	3.001538	6.402836	4.04E-10	8.78E-09	12.3928

ALDH2	-1.79838	2.314614	-6.39778	4.16E-10	9.03E-09	12.36346
SERINC2	2.282699	6.704394	6.397411	4.17E-10	9.05E-09	12.3613
DSCC1	1.101726	2.166362	6.392059	4.31E-10	9.30E-09	12.33024
ADRA2A	-1.18628	0.949426	-6.3865	4.45E-10	9.59E-09	12.29798
RAMP1	-2.52396	2.751819	-6.38462	4.50E-10	9.68E-09	12.28713
CLEC10A	-1.34184	1.020481	-6.37115	4.88E-10	1.04E-08	12.20912
CCND2	-1.82476	2.32914	-6.37092	4.88E-10	1.04E-08	12.20776
BAIAP2L1	1.183886	3.950354	6.370562	4.89E-10	1.05E-08	12.2057
MND1	1.049459	1.978709	6.366883	5.00E-10	1.07E-08	12.18442
SOD3	-2.33997	3.305308	-6.36624	5.02E-10	1.07E-08	12.18068
ATAD2	1.231869	3.027143	6.36324	5.11E-10	1.09E-08	12.16336
MBOAT7	1.203925	5.312102	6.362543	5.13E-10	1.09E-08	12.15933
PFDN2	1.161048	6.829412	6.361716	5.16E-10	1.09E-08	12.15455
TSPAN7	-1.96483	1.635304	-6.36163	5.16E-10	1.09E-08	12.15407
UTRN	-1.0684	1.709401	-6.36034	5.20E-10	1.10E-08	12.14661
ISG15	2.227098	6.865069	6.359256	5.24E-10	1.11E-08	12.14034
GGH	1.692021	3.650943	6.349598	5.54E-10	1.17E-08	12.08457
CES1	-3.0772	1.867112	-6.3469	5.63E-10	1.19E-08	12.06901
HEPH	-1.3808	1.318152	-6.33322	6.11E-10	1.28E-08	11.99017
H1-2	2.125006	6.16052	6.330613	6.21E-10	1.30E-08	11.97517
ARHGEF2	-1.10661	1.167575	-6.32566	6.39E-10	1.33E-08	11.94666
NFIL3	-1.37061	3.811665	-6.32298	6.49E-10	1.35E-08	11.93127
SNHG3	1.05733	3.211553	6.320125	6.60E-10	1.37E-08	11.91485
NCAPG2	1.117081	2.451392	6.305903	7.18E-10	1.49E-08	11.83319
PER3	-1.07809	1.495635	-6.28379	8.18E-10	1.69E-08	11.70653
AP1M2	1.860064	5.242491	6.280462	8.34E-10	1.72E-08	11.68748
PTPRN2	-1.12339	0.866461	-6.28021	8.36E-10	1.72E-08	11.68604
TMTC1	-1.07253	0.809951	-6.27026	8.86E-10	1.82E-08	11.62919
IGSF8	1.130763	4.906623	6.267238	9.02E-10	1.85E-08	11.61194
PMP22	-2.07046	3.806937	-6.26371	9.21E-10	1.89E-08	11.59178
NCAPD2	1.153595	4.217048	6.249987	9.98E-10	2.04E-08	11.51358
RND1	-1.07929	1.116547	-6.24211	1.05E-09	2.13E-08	11.46874
CCN5	-1.48919	0.870825	-6.23819	1.07E-09	2.17E-08	11.44646
MIF	1.167044	5.722288	6.22705	1.14E-09	2.31E-08	11.38317
MT1A	-1.88379	1.128368	-6.19761	1.36E-09	2.71E-08	11.21637
TOMM34	1.108625	5.353897	6.194534	1.38E-09	2.76E-08	11.19897
SNHG25	1.258731	2.138757	6.189911	1.42E-09	2.83E-08	11.17284
PCAT6	1.416008	2.884319	6.180131	1.50E-09	2.98E-08	11.11763
FBXO6	1.049368	3.613059	6.177704	1.52E-09	3.02E-08	11.10394
APOC1	2.094591	4.372706	6.17147	1.58E-09	3.12E-08	11.0688
HOXB7	1.398915	4.073854	6.168787	1.60E-09	3.17E-08	11.05368
PPP1R14B	1.381379	2.466978	6.14954	1.79E-09	3.50E-08	10.9454
ARHGEF3	-1.12266	1.405329	-6.1401	1.89E-09	3.69E-08	10.89241
TGFBR2	-1.32763	3.997959	-6.14	1.89E-09	3.69E-08	10.89183
INCENP	1.077705	2.897953	6.136135	1.94E-09	3.76E-08	10.87016
MMP11	2.8744	3.530055	6.114894	2.19E-09	4.22E-08	10.75121
PPP1R3B	-1.21645	2.620602	-6.11453	2.19E-09	4.23E-08	10.74915
TSC22D3	-1.45743	4.176257	-6.11371	2.20E-09	4.25E-08	10.74462
CHMP4C	1.440526	3.771192	6.108971	2.26E-09	4.36E-08	10.7181
LHFPL6	-1.50048	3.138321	-6.10401	2.33E-09	4.48E-08	10.69038
IL33	-2.10561	1.84434	-6.09576	2.44E-09	4.67E-08	10.64435
ATP2B4	-1.44522	3.557236	-6.09536	2.45E-09	4.68E-08	10.64215

CLSTN3	1.118697	3.646866	6.09256	2.49E-09	4.76E-08	10.62652
ARMCX1	-1.5178	2.038663	-6.08663	2.58E-09	4.90E-08	10.59348
ITGA1	-1.07295	1.589194	-6.06106	2.98E-09	5.63E-08	10.45131
FASN	1.367946	5.267906	6.060479	2.99E-09	5.65E-08	10.44807
P3H4	1.26018	3.358551	6.059799	3.00E-09	5.67E-08	10.4443
ULBP2	1.582512	2.230925	6.053235	3.12E-09	5.86E-08	10.40789
MIR7111	1.116389	1.553518	6.052872	3.12E-09	5.87E-08	10.40588
HILPDA	1.686333	4.162589	6.049233	3.19E-09	5.98E-08	10.3857
DCST1-AS	1.001112	1.519502	6.040127	3.36E-09	6.27E-08	10.33528
SDC2	-1.65375	3.026862	-6.03681	3.43E-09	6.38E-08	10.31691
CENPW	1.435191	4.357184	6.028446	3.59E-09	6.67E-08	10.27068
IER3	-1.87652	5.493627	-6.02682	3.63E-09	6.72E-08	10.26171
CPQ	-1.38904	2.77201	-6.02144	3.74E-09	6.91E-08	10.23197
JUND	-1.18932	6.399772	-6.01614	3.85E-09	7.10E-08	10.20275
NTN1	-1.3066	0.997819	-6.01328	3.92E-09	7.20E-08	10.187
PBXIP1	-1.0499	5.336627	-6.01037	3.98E-09	7.32E-08	10.17095
NFIB	-1.18688	2.032095	-5.98784	4.53E-09	8.25E-08	10.04694
HOMER3	1.147711	3.947419	5.985142	4.59E-09	8.36E-08	10.03214
CD200	-1.40712	1.805887	-5.97112	4.97E-09	9.00E-08	9.955177
EPCAM	2.168778	5.341925	5.970392	4.99E-09	9.02E-08	9.951215
PSME2	1.121786	5.560181	5.968525	5.05E-09	9.12E-08	9.940984
ARRDC1	1.023837	4.167704	5.967769	5.07E-09	9.15E-08	9.936841
LMCD1	-1.42146	1.878137	-5.96744	5.08E-09	9.16E-08	9.935065
PKP3	1.729076	4.899226	5.966684	5.10E-09	9.20E-08	9.9309
TNFSF12	-1.20089	3.159263	-5.95746	5.37E-09	9.65E-08	9.880421
PTGS2	-2.32712	2.988861	-5.94976	5.61E-09	1.00E-07	9.838321
UGCG	-1.034	3.874808	-5.94385	5.80E-09	1.04E-07	9.806004
PNRC1	-1.00819	4.513669	-5.93661	6.04E-09	1.08E-07	9.76652
NMB	1.365076	3.797806	5.927421	6.36E-09	1.13E-07	9.716428
TNFRSF2	1.057781	2.491398	5.927351	6.37E-09	1.13E-07	9.716046
DNMT3B	1.283119	2.036523	5.926946	6.38E-09	1.13E-07	9.713841
COL4A6	-1.80676	1.898973	-5.92433	6.48E-09	1.15E-07	9.699613
PRSS8	2.218161	4.31045	5.910863	6.98E-09	1.23E-07	9.626342
NUDT8	1.233684	2.964221	5.907385	7.12E-09	1.25E-07	9.607444
ADAMTS	-1.46634	1.690941	-5.90703	7.14E-09	1.25E-07	9.605493
ITGA5	-1.88137	3.870155	-5.90126	7.37E-09	1.29E-07	9.574193
TRAF4	1.271377	5.067288	5.890629	7.82E-09	1.36E-07	9.516547
DPYSL2	-1.3531	2.629776	-5.88029	8.29E-09	1.44E-07	9.460542
CSDC2	-1.25731	0.877685	-5.87882	8.36E-09	1.45E-07	9.452631
NR2F6	1.082443	5.050118	5.875673	8.51E-09	1.47E-07	9.435595
CCT5	1.025779	5.467908	5.859497	9.31E-09	1.60E-07	9.348235
HIP1R	1.040744	3.587311	5.857475	9.41E-09	1.61E-07	9.337329
GPR183	-1.65331	2.193839	-5.85403	9.60E-09	1.64E-07	9.318734
AKT3	-1.2225	1.572621	-5.85354	9.62E-09	1.64E-07	9.316108
SELP	-1.28494	1.13496	-5.85269	9.67E-09	1.65E-07	9.31156
ZNF692	1.09316	3.325518	5.843615	1.02E-08	1.73E-07	9.262666
MARVEL	1.00558	2.003064	5.829273	1.10E-08	1.87E-07	9.185562
RSRP1	1.06814	3.088388	5.820504	1.16E-08	1.95E-07	9.138499
HSBP1L1	1.021066	3.054109	5.820348	1.16E-08	1.95E-07	9.137661
HENMT1	1.06025	3.042561	5.816737	1.18E-08	1.99E-07	9.118295
MCAM	-1.35609	3.581379	-5.81557	1.19E-08	2.00E-07	9.112017
ECSCR	-1.06833	1.619661	-5.80501	1.26E-08	2.11E-07	9.055467

F11R	1.340293	5.649148	5.794652	1.33E-08	2.22E-07	9.000102
PARP12	1.105152	3.264521	5.792922	1.35E-08	2.24E-07	8.990856
MIR4477E	1.526767	3.019531	5.775932	1.48E-08	2.45E-07	8.900209
SLC25A1(	1.244371	3.391664	5.769964	1.53E-08	2.52E-07	8.868427
SLC29A2	1.316298	2.542511	5.768798	1.54E-08	2.53E-07	8.862215
PITX2	-1.05263	1.002329	-5.76707	1.55E-08	2.56E-07	8.852996
ENO1	1.00575	8.536664	5.760696	1.61E-08	2.64E-07	8.819118
EFNA1	1.506879	5.087622	5.760676	1.61E-08	2.64E-07	8.819012
GMNN	1.106697	3.932851	5.755855	1.65E-08	2.71E-07	8.793387
MEX3A	1.721531	2.443901	5.753929	1.67E-08	2.74E-07	8.783157
ESRP1	1.628986	4.699661	5.745272	1.75E-08	2.86E-07	8.737208
CCDC3	-1.26467	2.301165	-5.73657	1.84E-08	2.98E-07	8.691073
KLRB1	-1.03321	1.00173	-5.73464	1.86E-08	3.01E-07	8.680876
IRF5	1.30228	3.079461	5.733059	1.87E-08	3.03E-07	8.672484
AGTRAP	1.026476	4.576542	5.731104	1.89E-08	3.06E-07	8.662132
EMILIN1	-2.1981	3.767866	-5.7305	1.90E-08	3.07E-07	8.658921
RCSD1	-1.14292	1.294021	-5.73006	1.90E-08	3.08E-07	8.656606
SGCB	-1.09363	2.655891	-5.72975	1.91E-08	3.08E-07	8.65496
SLC22A3	-1.20834	0.913959	-5.72888	1.91E-08	3.09E-07	8.650368
PCCA-DT	1.046731	3.226821	5.726298	1.94E-08	3.14E-07	8.636703
PPP1R15A	-1.15816	5.385351	-5.7258	1.95E-08	3.14E-07	8.634084
H3C4	1.482717	1.87964	5.72228	1.99E-08	3.20E-07	8.615459
MIR25	1.402084	2.682907	5.720875	2.00E-08	3.22E-07	8.608034
CHAC1	1.125009	1.698112	5.715628	2.06E-08	3.31E-07	8.580312
TBL1X	-1.02464	2.462736	-5.71031	2.12E-08	3.39E-07	8.552225
PHYHD1	-1.34426	1.318534	-5.71003	2.12E-08	3.40E-07	8.550769
MAPRE2	-1.09089	2.197799	-5.6897	2.37E-08	3.77E-07	8.443643
FZD2	1.315225	2.386003	5.686623	2.41E-08	3.82E-07	8.427473
MAD2L2	1.038436	3.598282	5.673464	2.59E-08	4.08E-07	8.358349
GRB7	1.733278	4.154767	5.67287	2.60E-08	4.09E-07	8.355234
KIFC2	1.475684	3.197275	5.665755	2.70E-08	4.24E-07	8.317921
SH3BGRL	-1.26039	5.075337	-5.65768	2.82E-08	4.41E-07	8.275617
AMOTL1	-1.23079	2.487364	-5.6534	2.89E-08	4.50E-07	8.253237
MIEN1	1.131427	4.831912	5.640337	3.10E-08	4.81E-07	8.184952
TACC1	-1.37069	3.00113	-5.63848	3.13E-08	4.85E-07	8.175244
H4C9	1.44018	2.984658	5.635198	3.19E-08	4.93E-07	8.158129
H2AC8	1.811277	2.473616	5.634011	3.21E-08	4.96E-07	8.151936
GABARAI	-1.0174	3.678459	-5.63399	3.21E-08	4.96E-07	8.151801
AHNAK	-1.40442	5.056925	-5.62881	3.30E-08	5.09E-07	8.124829
STAP2	1.421462	4.634873	5.610806	3.63E-08	5.58E-07	8.031109
MREG	1.028921	2.807516	5.600963	3.83E-08	5.86E-07	7.979987
MAFG-DT	1.07019	1.795724	5.595874	3.94E-08	6.01E-07	7.953585
E2F8	1.123422	2.009973	5.591001	4.04E-08	6.16E-07	7.928325
CLU	-2.56987	4.947017	-5.58851	4.10E-08	6.23E-07	7.915446
PBX1	-1.16545	2.276162	-5.58733	4.12E-08	6.27E-07	7.909327
PLEKHO2	-1.02347	3.197638	-5.58501	4.17E-08	6.34E-07	7.897279
DUSP5	-1.76107	4.478182	-5.57591	4.38E-08	6.62E-07	7.850235
PCDH7	-1.44049	1.382816	-5.57193	4.48E-08	6.76E-07	7.829637
MGP	-2.49807	4.833484	-5.56188	4.72E-08	7.10E-07	7.777749
PPP1R13L	1.296491	4.439924	5.560208	4.77E-08	7.16E-07	7.769141
GPRIN1	1.139319	1.500043	5.554347	4.92E-08	7.38E-07	7.738929
MIR647	1.320283	2.699838	5.549493	5.05E-08	7.55E-07	7.713926

IGSF9	1.58034	2.782359	5.548002	5.09E-08	7.61E-07	7.706249
CXCL2	-1.89865	1.837676	-5.54505	5.17E-08	7.71E-07	7.691043
SPP1	2.776836	4.686372	5.543589	5.21E-08	7.76E-07	7.683543
REEP2	-1.0621	0.963863	-5.54241	5.24E-08	7.80E-07	7.677455
CDH24	1.022069	2.268913	5.541839	5.26E-08	7.82E-07	7.674544
ATP8B2	-1.13582	1.614962	-5.52067	5.88E-08	8.70E-07	7.565864
IGFBP5	-2.44995	4.448425	-5.51398	6.10E-08	8.98E-07	7.531578
HACD1	-1.02776	1.085429	-5.51181	6.17E-08	9.08E-07	7.520472
TRIB1	-1.21006	4.561099	-5.5113	6.18E-08	9.10E-07	7.517855
TMEM238	1.341779	2.787382	5.505061	6.39E-08	9.39E-07	7.485956
TRIB3	1.391162	3.127613	5.502696	6.47E-08	9.50E-07	7.473866
CX3CL1	-1.74583	2.311141	-5.49724	6.66E-08	9.76E-07	7.44599
ABHD12	1.076476	5.244801	5.482034	7.22E-08	1.05E-06	7.368433
TRPA1	-1.15657	0.749589	-5.47722	7.40E-08	1.08E-06	7.343922
JUP	1.447957	7.32482	5.474776	7.50E-08	1.09E-06	7.331479
MAP3K8	-1.01863	2.639814	-5.46567	7.87E-08	1.14E-06	7.285195
KCNJ8	-1.17901	1.558343	-5.46319	7.97E-08	1.15E-06	7.272579
TCF19	1.276521	3.527043	5.459172	8.14E-08	1.17E-06	7.252173
JDP2	-1.01466	2.085557	-5.44934	8.58E-08	1.23E-06	7.202302
CDC42EP:	-1.08477	2.293641	-5.44747	8.66E-08	1.24E-06	7.192813
CDIP1	-1.31304	2.070444	-5.44251	8.89E-08	1.27E-06	7.167733
TPPP3	-1.49861	2.269103	-5.43875	9.07E-08	1.30E-06	7.148699
CD69	-1.34203	1.315019	-5.43424	9.28E-08	1.33E-06	7.125875
PRRG2	1.175387	3.168468	5.429911	9.50E-08	1.35E-06	7.103991
SLC35F2	1.071145	2.728946	5.427446	9.62E-08	1.37E-06	7.091538
SPAG4	1.459611	3.029524	5.426548	9.66E-08	1.38E-06	7.087004
CAVIN1	-1.85945	5.505973	-5.42177	9.91E-08	1.41E-06	7.062877
DBI	1.004431	5.847574	5.418042	1.01E-07	1.43E-06	7.044083
MELTF-A	1.002231	1.824391	5.407627	1.07E-07	1.51E-06	6.991603
CCNL2	1.127039	4.525444	5.40609	1.08E-07	1.52E-06	6.98387
H2BC9	1.753669	2.229497	5.399513	1.11E-07	1.57E-06	6.950786
TCIM	-2.21986	4.758013	-5.39899	1.12E-07	1.57E-06	6.94813
NFE2L3	1.33221	3.047933	5.394864	1.14E-07	1.60E-06	6.927417
TMSB10	1.204866	11.25154	5.394694	1.14E-07	1.60E-06	6.926566
MSX1	-1.17987	1.427232	-5.3934	1.15E-07	1.61E-06	6.920073
IQANK1	1.542023	3.882773	5.389755	1.17E-07	1.64E-06	6.90176
FAM241B	1.072226	3.168084	5.384871	1.20E-07	1.68E-06	6.877253
GPC6	-1.2245	1.158204	-5.35701	1.39E-07	1.92E-06	6.737844
CDCA7	1.273215	2.437558	5.343377	1.49E-07	2.05E-06	6.669826
CDC42EP:	-1.14012	2.9193	-5.34118	1.51E-07	2.07E-06	6.658859
SELENOP	-1.61355	2.199479	-5.34042	1.51E-07	2.08E-06	6.655122
TSTD1	1.477129	5.273982	5.328165	1.61E-07	2.21E-06	6.594135
ITPR3	1.180101	3.175733	5.320915	1.67E-07	2.28E-06	6.558123
AP1G2	1.007253	3.357377	5.31398	1.73E-07	2.36E-06	6.523719
THBS1	-1.876	4.589256	-5.31145	1.76E-07	2.39E-06	6.511179
RHPN1	1.587053	2.909442	5.298957	1.87E-07	2.54E-06	6.449327
TAGLN2	1.096406	8.550103	5.288592	1.98E-07	2.66E-06	6.398104
TENT5C	-1.0497	1.58985	-5.28839	1.98E-07	2.67E-06	6.397119
H2BC11	1.515595	2.075852	5.286934	1.99E-07	2.68E-06	6.38992
SCD	1.794631	5.688113	5.274792	2.12E-07	2.84E-06	6.330047
JCAD	-1.18821	1.831852	-5.27314	2.14E-07	2.87E-06	6.321889
ZFP36L1	-1.01313	6.169703	-5.26663	2.21E-07	2.95E-06	6.289855

RTKN	1.182121	2.983401	5.266204	2.22E-07	2.96E-06	6.28777
MIR621	1.09981	3.411271	5.262817	2.25E-07	3.01E-06	6.271115
SLC2A3	-1.57145	2.559513	-5.26073	2.28E-07	3.03E-06	6.260841
WNT9A	-1.03857	1.367912	-5.25911	2.30E-07	3.05E-06	6.252876
SNORD14	1.612278	2.366825	5.257029	2.32E-07	3.08E-06	6.242673
DPYSL3	-1.92632	3.481538	-5.25664	2.33E-07	3.09E-06	6.240783
DYNC1I1	-1.04285	1.161833	-5.24694	2.44E-07	3.22E-06	6.193166
MDK	1.691807	6.719743	5.244897	2.47E-07	3.26E-06	6.183149
PALM	-1.36378	1.593289	-5.23457	2.60E-07	3.42E-06	6.132557
STEAP3	1.19363	3.764601	5.224976	2.73E-07	3.57E-06	6.085663
RGCC	-1.13397	3.779739	-5.21858	2.82E-07	3.68E-06	6.054447
ROR2	-1.26473	1.378433	-5.2165	2.85E-07	3.71E-06	6.044268
MEG3	-1.00622	0.88842	-5.21513	2.87E-07	3.74E-06	6.037627
ADAM8	1.467571	3.524516	5.209249	2.96E-07	3.83E-06	6.008935
F13A1	-1.74589	1.990169	-5.19769	3.14E-07	4.05E-06	5.952676
CDCP1	1.339373	3.567533	5.190389	3.26E-07	4.19E-06	5.91719
GPT2	1.109837	2.874017	5.174385	3.53E-07	4.51E-06	5.839567
KCTD12	-1.29273	3.090406	-5.16188	3.76E-07	4.77E-06	5.779047
GRHL2	1.300934	3.657524	5.161762	3.76E-07	4.77E-06	5.778493
LAD1	1.804948	6.208571	5.145986	4.07E-07	5.14E-06	5.702347
FYN	-1.04374	2.129668	-5.14314	4.13E-07	5.21E-06	5.688612
CBX2	1.384288	2.002944	5.141121	4.17E-07	5.26E-06	5.678907
SPINT2	1.358806	6.848345	5.136475	4.27E-07	5.38E-06	5.656537
GPX3	-2.05653	3.997996	-5.12958	4.42E-07	5.55E-06	5.623398
NDN	-1.51107	2.786426	-5.12826	4.45E-07	5.59E-06	5.617045
MIR23AH	-1.29687	3.516348	-5.12121	4.61E-07	5.77E-06	5.583179
HMGB2	1.114355	5.592993	5.115037	4.75E-07	5.93E-06	5.55356
SNX10	1.127779	2.187315	5.110912	4.85E-07	6.04E-06	5.533788
ARRDC4	-1.02444	2.294807	-5.10496	5.00E-07	6.20E-06	5.505272
CD248	-1.58723	3.616143	-5.1022	5.07E-07	6.28E-06	5.492083
H2AC7	1.253129	1.386952	5.094494	5.27E-07	6.51E-06	5.455239
SGCE	-1.42069	2.479856	-5.08417	5.54E-07	6.83E-06	5.405946
CAPG	1.287926	6.61188	5.083525	5.56E-07	6.85E-06	5.402887
KIAA1522	1.143599	5.511788	5.078533	5.70E-07	7.00E-06	5.379091
CYP27A1	-1.40156	2.284816	-5.076	5.77E-07	7.08E-06	5.367024
RBP7	-1.33178	1.957432	-5.07047	5.93E-07	7.26E-06	5.340687
GAS6	-1.63824	3.541215	-5.0571	6.34E-07	7.71E-06	5.277174
SDC1	1.877975	8.12578	5.052182	6.49E-07	7.89E-06	5.253833
SYNE4	1.49014	2.401671	5.048607	6.61E-07	8.02E-06	5.236887
SGK1	-1.50567	2.947605	-5.04727	6.65E-07	8.06E-06	5.230544
NT5DC2	1.055377	4.382086	5.042997	6.80E-07	8.23E-06	5.21031
RND3	-1.26682	4.203062	-5.04006	6.90E-07	8.34E-06	5.196415
CLIC4	-1.50004	4.543417	-5.03736	6.99E-07	8.45E-06	5.183635
LLGL2	1.259918	3.754943	5.029784	7.25E-07	8.73E-06	5.147824
MUC21	-1.07121	0.300931	-5.02915	7.28E-07	8.76E-06	5.144809
UNC93B1	1.03597	4.412167	5.024843	7.43E-07	8.94E-06	5.124493
PYGB	-1.01194	5.141376	-5.02443	7.45E-07	8.95E-06	5.122558
CST6	2.527573	3.608304	5.023326	7.49E-07	9.00E-06	5.117333
H2BC12	1.617066	5.508074	5.022053	7.53E-07	9.04E-06	5.111325
TGFB3	-1.30164	2.095952	-5.01688	7.73E-07	9.26E-06	5.086959
NAT14	1.019454	3.307184	5.011925	7.92E-07	9.46E-06	5.063593
CD55	-1.29614	4.343589	-5.01065	7.97E-07	9.52E-06	5.057596

WLS	-1.37607	3.770698	-5.00523	8.18E-07	9.76E-06	5.032098
GRIN2D	1.590471	1.912759	5.000765	8.36E-07	9.94E-06	5.011091
SFN	2.15566	8.396523	4.992018	8.73E-07	1.03E-05	4.970018
PROS1	-1.04954	1.909047	-4.98985	8.82E-07	1.04E-05	4.959862
SPINT1-A	1.225462	3.406546	4.980127	9.25E-07	1.09E-05	4.914281
SELENOM	-1.56068	3.581792	-4.97864	9.32E-07	1.10E-05	4.907331
SNORA33	1.016504	3.108988	4.961453	1.01E-06	1.19E-05	4.826988
PAM	-1.06694	3.129835	-4.96141	1.01E-06	1.19E-05	4.826802
FERMT1	1.280699	3.542471	4.949284	1.08E-06	1.26E-05	4.770259
SNORA26	1.029367	2.004792	4.948181	1.08E-06	1.26E-05	4.765124
NES	-1.31037	2.821659	-4.94168	1.12E-06	1.30E-05	4.734857
ETS2	-1.18395	5.476627	-4.93551	1.15E-06	1.33E-05	4.706221
ADGRG1	1.158207	4.63161	4.93172	1.17E-06	1.36E-05	4.688603
CYP1B1	-1.56245	1.570224	-4.93125	1.17E-06	1.36E-05	4.686402
H1-12P	1.237618	1.410556	4.929948	1.18E-06	1.37E-05	4.680378
APOD	-2.37773	2.940535	-4.92766	1.19E-06	1.38E-05	4.669777
SPRY2	-1.11521	2.346877	-4.9237	1.22E-06	1.41E-05	4.651415
PTX3	-1.41112	1.07647	-4.92334	1.22E-06	1.41E-05	4.649737
MPP7	-1.05269	2.151292	-4.91331	1.28E-06	1.47E-05	4.603289
LYNX1	-1.36864	1.575034	-4.91279	1.28E-06	1.47E-05	4.600879
LAMB2	-1.0894	4.360301	-4.91216	1.29E-06	1.47E-05	4.597985
CDH1	1.76472	5.937361	4.91045	1.30E-06	1.49E-05	4.59006
PSAT1	1.270744	3.789847	4.899455	1.37E-06	1.56E-05	4.53927
EPS8L2	1.160235	4.54435	4.897231	1.38E-06	1.57E-05	4.529009
PROSER2	1.15204	2.513425	4.895163	1.40E-06	1.59E-05	4.519474
ODC1	-1.34884	4.585251	-4.8947	1.40E-06	1.59E-05	4.517352
PLA2G4A	-1.21705	2.322182	-4.86291	1.63E-06	1.83E-05	4.371201
H2BC8	1.495588	1.926353	4.862755	1.63E-06	1.83E-05	4.370477
DUSP23	1.096005	5.49378	4.845291	1.77E-06	1.98E-05	4.290553
TMEM74I	1.194134	1.614842	4.841104	1.81E-06	2.01E-05	4.271429
FCHO1	1.020095	1.818219	4.834234	1.87E-06	2.07E-05	4.240081
COL7A1	1.804811	3.714279	4.832519	1.89E-06	2.09E-05	4.232264
TUBA5P	1.238013	1.849593	4.830648	1.90E-06	2.11E-05	4.223738
ARHGEF1	1.138107	3.487618	4.829936	1.91E-06	2.11E-05	4.220491
TMEM119	-1.42952	2.217611	-4.8297	1.91E-06	2.11E-05	4.2194
DBNDD1	1.20208	2.300079	4.821964	1.98E-06	2.19E-05	4.1842
PDLIM7	-1.19315	4.007407	-4.81946	2.01E-06	2.21E-05	4.172823
NEURL1E	-1.03545	2.284495	-4.81925	2.01E-06	2.21E-05	4.171834
NCOA7	-1.04133	2.730298	-4.81748	2.03E-06	2.23E-05	4.163793
IGFBP6	-1.95305	3.85507	-4.80329	2.17E-06	2.37E-05	4.099395
PLEKHO1	-1.13732	3.05907	-4.77142	2.52E-06	2.71E-05	3.95531
PNMA1	-1.12492	3.470877	-4.77136	2.52E-06	2.71E-05	3.955044
EFEMP1	-2.09627	2.794751	-4.76891	2.55E-06	2.74E-05	3.944042
DHCR24	1.624684	5.650579	4.760751	2.65E-06	2.84E-05	3.907299
ABCC4	-1.00236	1.890867	-4.75682	2.70E-06	2.89E-05	3.889635
H3C10	1.44457	2.073152	4.75603	2.71E-06	2.90E-05	3.886073
ID4	-1.67413	2.771349	-4.7556	2.71E-06	2.90E-05	3.88414
NCKAP1L	-1.08226	1.276193	-4.75309	2.75E-06	2.93E-05	3.872868
RAB25	1.749477	6.19182	4.749801	2.79E-06	2.97E-05	3.858102
CBX6	-1.09644	2.845676	-4.73896	2.93E-06	3.11E-05	3.809516
CSPG4	-1.30248	2.227122	-4.73825	2.94E-06	3.12E-05	3.806324
GALNT6	1.126791	1.973497	4.727492	3.10E-06	3.27E-05	3.758185

DUX4L50	1.048552	2.512638	4.725822	3.12E-06	3.29E-05	3.750721
TUBA1A	-1.53146	4.727738	-4.72484	3.13E-06	3.30E-05	3.746331
SREBF1	1.080206	4.365619	4.720554	3.20E-06	3.36E-05	3.727193
TACSTD2	1.906424	8.088614	4.717993	3.24E-06	3.40E-05	3.715765
MT1M	-1.45351	1.28046	-4.71551	3.27E-06	3.43E-05	3.704678
ENPP2	-1.15878	1.748939	-4.70934	3.37E-06	3.53E-05	3.677211
H2BC4	1.36414	2.061813	4.709046	3.37E-06	3.53E-05	3.675887
PGAP4	-1.04992	1.316906	-4.70771	3.39E-06	3.55E-05	3.669942
SLC9A3R	1.148212	5.023725	4.704537	3.45E-06	3.60E-05	3.655812
CFH	-1.39533	2.891124	-4.70298	3.47E-06	3.63E-05	3.648871
SNORD60	1.041045	1.806612	4.693553	3.63E-06	3.77E-05	3.606989
NFKBIZ	-1.04076	2.546706	-4.68769	3.73E-06	3.87E-05	3.580962
OAS3	1.22765	3.825392	4.687622	3.73E-06	3.87E-05	3.580665
PECAM1	-1.09656	3.494682	-4.68539	3.77E-06	3.91E-05	3.570779
SOWAHC	-1.07043	3.448989	-4.68531	3.77E-06	3.91E-05	3.570429
C3	-2.14261	4.225143	-4.67842	3.89E-06	4.02E-05	3.539882
MAPK15	1.117136	1.519883	4.651077	4.41E-06	4.52E-05	3.419146
FAM50B	-1.09937	1.548469	-4.6447	4.55E-06	4.64E-05	3.391094
S1PR5	1.333692	2.277467	4.639992	4.65E-06	4.73E-05	3.370378
PODXL2	1.68329	3.965201	4.627214	4.93E-06	4.99E-05	3.314293
KLC3	1.15025	1.548992	4.626009	4.95E-06	5.02E-05	3.309009
FNDC10	1.202599	3.032788	4.622907	5.03E-06	5.08E-05	3.295416
SELENBP	-1.6025	2.987116	-4.62283	5.03E-06	5.08E-05	3.295074
SHH	-1.8576	1.388913	-4.62163	5.05E-06	5.10E-05	3.289816
ANGPTL2	-1.41551	3.249478	-4.6195	5.10E-06	5.15E-05	3.280501
EPHA1	1.181537	3.331671	4.619448	5.11E-06	5.15E-05	3.280274
SPTBN2	1.074272	2.524323	4.610171	5.33E-06	5.36E-05	3.239702
HBA2	-1.5318	2.329338	-4.60688	5.41E-06	5.43E-05	3.225331
AR	-1.02593	0.961227	-4.60436	5.47E-06	5.49E-05	3.214336
MAL2	1.741814	5.688194	4.581687	6.07E-06	6.03E-05	3.115597
MIR200CI	1.541787	3.958826	4.579549	6.13E-06	6.09E-05	3.106311
GLIPR2	-1.24196	2.558964	-4.57908	6.14E-06	6.10E-05	3.104292
CTSF	-1.09925	3.71415	-4.5772	6.19E-06	6.14E-05	3.09613
PTK7	1.028839	3.787608	4.57152	6.36E-06	6.29E-05	3.071469
MYC	-1.5374	4.623556	-4.56753	6.47E-06	6.39E-05	3.054187
CDC42BP	1.014169	3.354357	4.566475	6.50E-06	6.42E-05	3.049603
GNA14	-1.04387	1.238185	-4.5639	6.58E-06	6.48E-05	3.038442
KRT7	2.976529	7.757696	4.561744	6.64E-06	6.54E-05	3.02912
ELMO3	1.276895	4.043247	4.561629	6.65E-06	6.54E-05	3.028624
MICB	1.030063	1.819293	4.543908	7.20E-06	7.03E-05	2.952071
INHBB	-1.01661	1.708058	-4.53998	7.33E-06	7.15E-05	2.935156
S100B	-1.12391	1.474949	-4.53747	7.42E-06	7.22E-05	2.92432
NXPH4	1.46628	1.669697	4.536959	7.43E-06	7.24E-05	2.922127
CD93	-1.06613	2.864905	-4.53649	7.45E-06	7.25E-05	2.920125
H2BC21	1.317622	3.619073	4.535549	7.48E-06	7.28E-05	2.916056
DDR1	1.025403	5.167609	4.534164	7.53E-06	7.32E-05	2.910091
CST1	2.384497	2.475826	4.530437	7.66E-06	7.43E-05	2.894057
MEST	1.575807	3.1744	4.520754	8.00E-06	7.73E-05	2.852456
FBXO32	-1.20698	2.9139	-4.51936	8.05E-06	7.78E-05	2.846491
SFRP2	-2.99121	4.000173	-4.51904	8.06E-06	7.79E-05	2.845091
CBLC	1.590046	4.877023	4.507864	8.48E-06	8.15E-05	2.797198
PLAU	1.710245	5.331638	4.505535	8.57E-06	8.23E-05	2.787231

DSG2	1.295734	4.571881	4.504781	8.59E-06	8.25E-05	2.784003
LAMA4	-1.05835	2.466025	-4.50381	8.63E-06	8.28E-05	2.779837
SERPINF1	-1.66061	4.312876	-4.46366	1.03E-05	9.75E-05	2.608783
TNFAIP3	-1.30785	3.283472	-4.45288	1.08E-05	0.000102	2.563092
CCNO	1.064423	2.425119	4.451793	1.09E-05	0.000102	2.558501
KIT	-1.22102	1.291192	-4.45128	1.09E-05	0.000102	2.556325
CAV1	-1.74968	4.50955	-4.44407	1.13E-05	0.000105	2.52584
HSPG2	-1.1124	3.243759	-4.44033	1.15E-05	0.000107	2.510031
COL6A2	-1.86657	6.042458	-4.4389	1.15E-05	0.000108	2.504006
NECTIN4	1.734888	5.387417	4.435801	1.17E-05	0.000109	2.490922
CRNN	-1.12913	0.244634	-4.42526	1.23E-05	0.000114	2.446488
ID2	-1.27429	4.043576	-4.42458	1.23E-05	0.000114	2.443637
LPCAT1	1.082971	4.094589	4.422151	1.24E-05	0.000115	2.433413
CLDN7	1.422322	5.536674	4.419887	1.25E-05	0.000116	2.423892
MROH6	1.14262	2.586713	4.416475	1.27E-05	0.000118	2.409551
CDH3	1.922745	3.832155	4.416295	1.27E-05	0.000118	2.408793
NRP2	-1.0596	1.711155	-4.41364	1.29E-05	0.000119	2.397649
HBB	-1.71098	2.951448	-4.41147	1.30E-05	0.00012	2.388518
UNC13D	1.311869	3.230585	4.410282	1.31E-05	0.00012	2.383543
APOBEC3	1.393253	2.918923	4.397756	1.38E-05	0.000127	2.331039
PDLIM4	-1.35828	2.402186	-4.39622	1.39E-05	0.000127	2.324593
EVPL	1.532723	4.811511	4.390169	1.43E-05	0.00013	2.299307
ESRP2	1.239505	3.899023	4.380254	1.49E-05	0.000136	2.257913
RBBP8NL	1.303366	2.319056	4.373252	1.54E-05	0.000139	2.228727
STEAP4	-1.03339	1.204696	-4.37137	1.55E-05	0.000141	2.220882
TFAP2C	1.067743	3.106361	4.362236	1.62E-05	0.000146	2.182905
COL10A1	1.723396	1.721026	4.358032	1.65E-05	0.000148	2.165445
FABP4	-2.77128	3.14873	-4.35778	1.65E-05	0.000148	2.164387
CPE	-1.28052	2.852224	-4.34794	1.72E-05	0.000154	2.1236
NUP210	1.305573	3.165371	4.34646	1.73E-05	0.000155	2.117462
EPN3	1.37301	3.678537	4.339986	1.78E-05	0.000159	2.090671
MDFI	1.401656	4.085311	4.332997	1.84E-05	0.000164	2.061789
APCDD1	-1.20162	2.522142	-4.33299	1.84E-05	0.000164	2.061768
LRRC32	-1.13152	2.616091	-4.32945	1.87E-05	0.000166	2.047154
DUSP2	-1.65102	3.478681	-4.32621	1.89E-05	0.000168	2.033779
NRN1	-1.15522	1.505265	-4.31246	2.01E-05	0.000177	1.977168
CDKN1A	-1.12596	6.081777	-4.30927	2.04E-05	0.00018	1.964042
FSCN1	1.446554	5.81002	4.308562	2.04E-05	0.00018	1.96114
AXL	-1.33543	2.786662	-4.30163	2.10E-05	0.000185	1.932702
PLEK2	1.333691	3.933933	4.300074	2.12E-05	0.000186	1.926303
SOX7	-1.1272	1.571203	-4.28764	2.24E-05	0.000196	1.875397
PYCARD	1.065574	4.317366	4.282746	2.28E-05	0.000199	1.855375
OAS1	1.215795	4.352595	4.276546	2.35E-05	0.000204	1.830059
VPS9D1-A	1.025686	1.984064	4.273451	2.38E-05	0.000207	1.817434
STC1	-1.19469	2.696696	-4.26808	2.43E-05	0.000211	1.795539
SLC16A3	1.142154	3.899754	4.267838	2.44E-05	0.000211	1.794559
PLA2G2A	-2.50562	3.434414	-4.26728	2.44E-05	0.000211	1.792276
KRT19	2.104946	9.563263	4.244444	2.69E-05	0.000231	1.699524
RIPPLY3	1.03845	1.569553	4.242728	2.71E-05	0.000233	1.692574
ATOH8	-1.13426	1.136207	-4.21214	3.09E-05	0.000262	1.569082
CAMSAP3	1.006249	3.044795	4.208081	3.14E-05	0.000266	1.552757
GALNT3	1.053865	3.213306	4.204154	3.20E-05	0.00027	1.536976

CD79B	-1.13845	1.271109	-4.20223	3.22E-05	0.000272	1.529239
NGFR	-1.17363	1.178488	-4.19967	3.26E-05	0.000275	1.518984
TINAGL1	1.457532	5.73334	4.198546	3.27E-05	0.000276	1.514465
CA9	2.078167	2.757776	4.188787	3.41E-05	0.000286	1.475355
LASTR	1.373799	1.521288	4.16935	3.70E-05	0.000308	1.397709
TIMP2	-1.52759	4.716765	-4.15577	3.92E-05	0.000324	1.343643
MFAP2	1.37894	3.604338	4.139942	4.19E-05	0.000345	1.280864
VIM	-1.35287	5.947165	-4.13393	4.30E-05	0.000353	1.257071
RGS1	-1.42081	3.064836	-4.13247	4.32E-05	0.000355	1.251311
LIF	-1.20321	2.147628	-4.13057	4.36E-05	0.000357	1.243779
H2AC6	1.17972	4.269588	4.107423	4.80E-05	0.00039	1.152546
CREB3L1	-1.27507	2.605833	-4.09694	5.01E-05	0.000406	1.111384
LRIG1	-1.04741	1.646676	-4.08842	5.19E-05	0.000419	1.077988
PTGER4	-1.00538	2.137498	-4.0744	5.50E-05	0.000442	1.023197
C1R	-1.45257	5.063723	-4.06487	5.73E-05	0.000458	0.986028
TUBB6	-1.49891	3.848766	-4.05333	6.00E-05	0.000478	0.941186
FBN1	-1.15882	1.95329	-4.04958	6.10E-05	0.000484	0.926621
C19orf33	1.705744	6.733502	4.047987	6.14E-05	0.000487	0.920431
KLHDC7E	2.259664	3.333091	4.035768	6.45E-05	0.00051	0.8731
ETV7	1.025922	2.627775	4.035492	6.46E-05	0.000511	0.872033
MANEAL	1.040193	2.486004	4.032232	6.55E-05	0.000517	0.859428
SCARA3	-1.28644	2.828604	-4.01333	7.07E-05	0.000554	0.786544
CD24	1.651863	6.748659	4.006911	7.26E-05	0.000567	0.761849
C15orf48	1.765041	3.661079	4.000053	7.47E-05	0.000581	0.735519
CYBA	1.016313	5.298822	3.993365	7.67E-05	0.000596	0.709878
TNNT1	1.437012	1.640119	3.979818	8.11E-05	0.000626	0.658069
TFAP2A	1.063846	2.376036	3.968101	8.50E-05	0.000653	0.613391
EMP3	-1.33246	3.646399	-3.96628	8.56E-05	0.000657	0.606457
STOM	-1.16732	5.145448	-3.95706	8.89E-05	0.000681	0.571411
LAMC2	1.872728	4.053385	3.950143	9.14E-05	0.000698	0.545152
IFI6	1.795513	7.269935	3.940115	9.52E-05	0.000724	0.507172
SERPINE2	-1.1707	1.678442	-3.93547	9.70E-05	0.000736	0.489625
WNT5B	-1.12385	1.658276	-3.93466	9.73E-05	0.000738	0.486554
C1S	-1.63368	4.644486	-3.93072	9.88E-05	0.000749	0.471673
EHD2	-1.1192	4.634862	-3.92915	9.95E-05	0.000752	0.465753
KDELR3	1.088588	3.448182	3.92745	0.0001	0.000756	0.45933
DAPL1	-1.34069	1.005947	-3.92093	0.000103	0.000774	0.434759
FKBP5	-1.03199	2.237886	-3.91568	0.000105	0.000789	0.414984
LDLR	-1.07494	3.01798	-3.91513	0.000105	0.00079	0.412922
SMPDL3B	1.037339	1.834909	3.901343	0.000111	0.000831	0.361166
SLC2A6	1.039968	2.511872	3.901312	0.000111	0.000831	0.361053
ALDH1A1	-1.69094	2.524621	-3.88996	0.000116	0.000866	0.318542
ACKR3	-1.28515	4.25357	-3.88442	0.000119	0.000883	0.29788
H4C5	1.05912	1.288714	3.865617	0.000128	0.000947	0.227822
KRT8	1.509527	7.934132	3.865391	0.000128	0.000947	0.226983
COL11A1	1.336969	1.390446	3.850503	0.000136	0.000999	0.171755
PLPP2	1.079659	3.551791	3.847111	0.000138	0.001011	0.1592
RHOV	1.454774	4.938288	3.845515	0.000139	0.001017	0.153297
SBK1	1.266114	1.799202	3.834799	0.000145	0.001056	0.113715
FOSL1	-1.37052	3.859524	-3.82882	0.000148	0.001078	0.091684
CSTB	1.014467	7.617335	3.812092	0.000158	0.001143	0.030187
THEM6	1.090532	4.616312	3.810744	0.000159	0.001148	0.025241

CTSV	1.158676	1.590217	3.798693	0.000167	0.001197	-0.01889
FXYD3	1.740153	6.860442	3.798543	0.000167	0.001197	-0.01943
TYMP	1.486611	4.763181	3.780974	0.000179	0.001273	-0.08353
TXNIP	-1.0585	6.704795	-3.77724	0.000181	0.00129	-0.09713
ZNF488	1.060236	1.527204	3.774185	0.000183	0.001304	-0.10823
TREM2	1.140995	2.514558	3.761543	0.000193	0.001364	-0.1541
IRF7	1.029168	3.667861	3.759284	0.000194	0.001375	-0.16228
FCRLB	1.336795	2.066892	3.749721	0.000202	0.001421	-0.19687
OSR1	-1.15125	1.919087	-3.74458	0.000206	0.001447	-0.21542
ITGB6	1.346085	3.998616	3.743644	0.000206	0.001451	-0.2188
PITX1	-1.25286	3.345996	-3.74088	0.000208	0.001464	-0.22875
PLPP4	1.039106	1.153898	3.728222	0.000219	0.001528	-0.27432
CCL21	-1.81955	2.415303	-3.7274	0.00022	0.001533	-0.27726
INHBA	1.202785	1.98608	3.723654	0.000223	0.00155	-0.29072
FBLN1	-1.57079	5.994417	-3.72148	0.000225	0.001562	-0.29853
PCP4L1	-1.86112	2.847927	-3.71634	0.000229	0.00159	-0.31696
WDR72	1.14815	2.057708	3.715419	0.00023	0.001594	-0.32024
ADAMTS	-1.13345	2.013025	-3.7054	0.000239	0.00165	-0.35609
HCAR1	1.042121	1.845724	3.703954	0.00024	0.001658	-0.36124
CLCA4	-1.99152	2.161166	-3.70018	0.000244	0.00168	-0.3747
S100A14	2.140337	6.323108	3.698539	0.000245	0.001689	-0.38057
PRR36	1.093769	1.498371	3.68601	0.000257	0.001764	-0.42518
HOXB5	1.054095	1.797174	3.683251	0.00026	0.001782	-0.43498
GALNT14	1.104643	2.202809	3.679317	0.000264	0.001804	-0.44895
CDKN2A	1.845249	2.503016	3.67421	0.000269	0.001835	-0.46706
FOLR2	-1.26141	2.483534	-3.67315	0.00027	0.001841	-0.47083
NUDT19-I	1.041883	2.394753	3.669487	0.000274	0.001863	-0.48379
TMEM18	1.093811	2.92909	3.659857	0.000284	0.001925	-0.51784
COL16A1	-1.17644	2.751155	-3.6524	0.000292	0.001971	-0.54414
ARL4D	-1.16717	3.880996	-3.64377	0.000302	0.00203	-0.57451
FSTL1	-1.00795	4.544751	-3.64222	0.000304	0.00204	-0.57997
ISYNA1	1.190057	4.618818	3.636702	0.00031	0.002077	-0.59936
CLMP	-1.07757	1.923519	-3.63396	0.000313	0.002096	-0.60896
HES4	1.086433	3.873756	3.615573	0.000336	0.002229	-0.67332
CDKN1C	-1.05255	2.869752	-3.61313	0.000339	0.002249	-0.68186
PRSS22	1.21239	3.898814	3.612223	0.00034	0.002255	-0.68501
TINCR	1.145907	2.323769	3.611259	0.000341	0.002261	-0.68837
MPEG1	-1.00209	1.960625	-3.59609	0.000361	0.002377	-0.74116
TMEM23	-1.31273	1.615467	-3.59581	0.000361	0.002378	-0.74214
DTX4	1.052904	3.701689	3.595009	0.000362	0.002384	-0.74491
HOXB6	1.070456	1.98157	3.592364	0.000366	0.002406	-0.7541
NNAT	-1.54009	1.562457	-3.58935	0.00037	0.002431	-0.76454
F3	-1.56692	3.581634	-3.5825	0.00038	0.002491	-0.78827
CASC9	1.054425	2.309668	3.567294	0.000402	0.002621	-0.8408
CXCR4	-1.06733	3.786288	-3.54867	0.00043	0.00279	-0.90485
SEZ6L2	1.17327	3.035239	3.535491	0.000452	0.002912	-0.94997
CLIC3	1.772716	4.000651	3.52361	0.000472	0.003027	-0.99051
COL6A1	-1.38772	5.740882	-3.52174	0.000475	0.003046	-0.99687
NECTIN1	1.120941	5.006258	3.520918	0.000477	0.003054	-0.99968
CRTAC1	-1.745	1.553288	-3.5193	0.000479	0.003067	-1.0052
METTL7E	1.008057	1.488397	3.515254	0.000486	0.003107	-1.01895
TMEM47	-1.11214	3.115189	-3.51344	0.00049	0.003122	-1.02513

GJB2	1.836932	5.139747	3.50323	0.000508	0.003225	-1.05976
ITGB4	1.15939	5.994922	3.492545	0.000529	0.003333	-1.09592
VGLL1	1.814258	4.36583	3.489002	0.000535	0.003372	-1.10789
FMOD	-1.14255	3.017079	-3.48484	0.000544	0.003416	-1.12192
COLCA1	-1.01211	1.198007	-3.47703	0.000559	0.0035	-1.14825
ARHGAP4	1.069935	2.028105	3.472886	0.000568	0.003546	-1.16217
FUT3	1.280658	2.733906	3.465514	0.000583	0.00363	-1.18692
IGFL2-AS	1.276024	1.46874	3.461262	0.000592	0.00368	-1.20118
PERP	1.077443	7.056377	3.457265	0.000601	0.003725	-1.21456
LYPD6B	1.070177	3.027467	3.449239	0.000618	0.003816	-1.24139
KLHDC7E	1.042465	1.511977	3.44751	0.000622	0.003839	-1.24716
MMP2	-1.35287	5.671544	-3.44742	0.000622	0.00384	-1.24747
PPP1R14C	1.360038	1.983533	3.445913	0.000626	0.003857	-1.25249
OAT	-1.12693	3.45621	-3.43443	0.000652	0.003997	-1.29073
ADH1C	-1.33555	1.310773	-3.43106	0.00066	0.004038	-1.30194
RRAD	-1.39895	2.352656	-3.43083	0.000661	0.004041	-1.30271
ERBB3	1.070823	4.16532	3.421322	0.000683	0.004165	-1.33426
CPXM1	-1.3059	2.84522	-3.4066	0.00072	0.004367	-1.38294
B3GNT3	1.318668	4.504068	3.404108	0.000727	0.004402	-1.39117
B4GALNT	1.168008	1.766028	3.394329	0.000752	0.004541	-1.42337
PPP1R1B	-1.29954	1.449794	-3.39397	0.000753	0.004546	-1.42456
RARRES2	-1.32626	4.147228	-3.37868	0.000795	0.004773	-1.47475
GJB6	1.677486	4.2038	3.372137	0.000814	0.004872	-1.49614
FER1L4	1.642394	3.596412	3.371851	0.000815	0.004876	-1.49707
AREG	-1.64105	3.089965	-3.3617	0.000845	0.005026	-1.53019
MT-ND6	-1.14422	10.36491	-3.34867	0.000884	0.005226	-1.57259
SMIM22	1.397045	4.185092	3.346729	0.00089	0.005257	-1.57889
KCNG1	1.28897	2.152791	3.343716	0.0009	0.005303	-1.58867
CCL19	-1.54334	2.103527	-3.34163	0.000906	0.005336	-1.59544
LSP1	-1.19381	3.28021	-3.33909	0.000915	0.005375	-1.60365
IRF6	1.107218	5.201439	3.338614	0.000916	0.005383	-1.6052
KRT14	2.82907	4.014929	3.311484	0.001007	0.005846	-1.69271
GAS1	-1.11937	1.829705	-3.28497	0.001104	0.006351	-1.77757
IGFBP2	-1.28314	3.143648	-3.28028	0.001122	0.006444	-1.79252
GATA3	1.61887	5.394818	3.277685	0.001132	0.006489	-1.80077
IGFL1P1	1.098038	1.299973	3.274933	0.001143	0.006541	-1.80952
CRISP3	-1.18758	1.01162	-3.27268	0.001152	0.006581	-1.81667
MMP1	1.834008	3.718931	3.269845	0.001164	0.006635	-1.82569
ALDH1L1	-1.03738	1.630077	-3.24423	0.001271	0.007161	-1.90671
KRTCAP3	1.225012	4.148668	3.226524	0.00135	0.007534	-1.96234
CHRDL2	-1.13686	1.405835	-3.2254	0.001355	0.007558	-1.96587
CXADR	1.00502	3.790739	3.218959	0.001385	0.007701	-1.98603
SRGN	-1.22833	5.19917	-3.21757	0.001392	0.007734	-1.99037
LUM	-1.4327	5.802069	-3.20947	0.00143	0.007923	-2.01566
TBX1	1.246979	2.231399	3.196066	0.001497	0.008245	-2.05738
EPS8L1	1.018117	3.338947	3.182626	0.001566	0.008574	-2.09905
ISLR	-1.49372	3.684303	-3.17615	0.001601	0.008739	-2.11906
ALDH3B2	1.281905	3.640268	3.16731	0.001649	0.008969	-2.14633
RHBG	1.052484	1.463189	3.15549	0.001716	0.00929	-2.18266
SP6	1.076982	2.890695	3.141682	0.001797	0.009659	-2.22495
LINC0151	1.003894	1.086877	3.126251	0.001892	0.0101	-2.27199
CASP14	1.856229	2.21282	3.125476	0.001897	0.010123	-2.27434

SERPING1	-1.17597	5.35713	-3.11663	0.001953	0.010393	-2.30119
DSP	1.347747	4.849592	3.113835	0.001971	0.010477	-2.30968
KRT7-AS	1.174861	2.804192	3.103108	0.002043	0.010808	-2.34213
FABP6	1.291633	2.605034	3.101136	0.002056	0.010866	-2.34808
AHNAK2	-1.03517	1.576102	-3.09067	0.002128	0.011194	-2.37961
AATBC	1.007882	1.8827	3.076057	0.002233	0.011679	-2.42348
RBP1	-1.03619	2.258306	-3.07131	0.002268	0.011844	-2.4377
LINC0181	1.106086	1.720134	3.0682	0.002291	0.011948	-2.44699
SERPINA1	1.306692	2.653339	3.064028	0.002323	0.012088	-2.45944
LY6E	1.159671	6.519948	3.06225	0.002336	0.012152	-2.46475
PSORS1C2	1.601517	2.044726	3.050514	0.002427	0.012564	-2.49968
CXCL10	1.679069	3.705756	3.040331	0.002509	0.012913	-2.52988
PALM3	1.328739	2.523826	3.032358	0.002575	0.013198	-2.55347
LINC0113	-1.07206	1.435058	-3.0213	0.002669	0.013612	-2.58606
MAGEA6	1.490514	1.722829	3.016368	0.002712	0.0138	-2.60059
MYCL	1.271284	3.841587	3.010852	0.00276	0.014018	-2.61678
TMEM139	1.04535	1.786565	3.010311	0.002765	0.014039	-2.61837
PROM2	1.186616	4.778639	3.010192	0.002766	0.014039	-2.61872
LINC0198	1.134039	1.772977	2.991935	0.002933	0.014763	-2.67212
COL6A3	-1.18413	4.002244	-2.99087	0.002943	0.014797	-2.67524
TLE2	-1.03902	3.361827	-2.97955	0.003052	0.015279	-2.70817
CTSK	-1.15702	4.68492	-2.97554	0.003091	0.015429	-2.71981
TMC4	1.066378	4.649203	2.973779	0.003109	0.015499	-2.72492
MMP13	1.31985	1.92546	2.954566	0.003305	0.016358	-2.78045
TNFAIP2	1.044565	6.218162	2.949079	0.003363	0.016615	-2.79625
DKK1	-1.36694	2.703803	-2.94464	0.003411	0.016807	-2.809
SLC1A6	1.165011	1.187913	2.930812	0.003563	0.017456	-2.84863
CHI3L1	1.404888	2.889068	2.930388	0.003568	0.017468	-2.84984
HES2	1.130828	1.859515	2.929316	0.00358	0.01752	-2.85291
IFI27	1.438114	5.184848	2.923605	0.003645	0.017793	-2.86921
MAGEA3	1.526981	1.883202	2.920475	0.003681	0.017945	-2.87814
PTK6	1.087865	4.253349	2.908988	0.003816	0.018503	-2.91081
GPC3	-1.04909	1.846719	-2.89559	0.003979	0.019196	-2.94875
CXCL14	-1.3997	3.605529	-2.89552	0.00398	0.019196	-2.94895
CXCL11	1.283971	1.946629	2.883875	0.004127	0.019795	-2.9818
PLA2G2F	1.43548	2.688484	2.876303	0.004226	0.020199	-3.00309
GPR87	1.037295	3.970105	2.861002	0.004431	0.021011	-3.04595
SOX9	1.052694	2.788001	2.852418	0.00455	0.021507	-3.0699
MYH14	1.069258	4.268389	2.849466	0.004592	0.021674	-3.07811
KCNN4	1.043414	4.17314	2.845622	0.004647	0.021882	-3.08881
MIR205H(	1.179799	4.105961	2.845407	0.00465	0.021891	-3.0894
TMEM97	1.108942	4.781693	2.820096	0.005025	0.023414	-3.15944
IGF2BP2	1.028041	2.003811	2.799229	0.005355	0.024707	-3.21673
MMP9	1.339676	3.068488	2.796421	0.005401	0.024901	-3.22441
FAM83A	1.392427	3.084399	2.789342	0.005519	0.025354	-3.24373
GAPDHP1	1.062101	2.481864	2.766297	0.005917	0.026898	-3.30631
IFIT3	1.033648	3.584823	2.75563	0.006109	0.02766	-3.33511
CA2	1.036108	2.618485	2.731128	0.006574	0.029405	-3.40085
LGALS4	-1.16634	1.372667	-2.72615	0.006672	0.029768	-3.41414
IGFL1	1.774521	3.055164	2.720152	0.006792	0.030235	-3.43011
AEBP1	-1.25622	4.881698	-2.71498	0.006898	0.030643	-3.44386
SCUBE2	-1.17334	2.305586	-2.70585	0.007087	0.031303	-3.46807

KRT17	1.743029	8.338806	2.693716	0.007345	0.032214	-3.50013
MYEOV	1.085141	1.828328	2.674007	0.007784	0.033759	-3.5519
CYP4F23I	1.015511	1.834129	2.673251	0.007801	0.033805	-3.55388
PSPHP1	1.435747	1.851547	2.661179	0.008081	0.034784	-3.5854
OLR1	1.028552	1.966637	2.650524	0.008336	0.035695	-3.6131
IGFBP3	1.172666	6.956782	2.64592	0.008449	0.036084	-3.62504
KRT16	1.875267	4.590146	2.605608	0.009493	0.039837	-3.72871
OLFM4	-1.44691	1.58861	-2.60406	0.009535	0.03998	-3.73266
CSAG1	1.237146	1.470038	2.600618	0.00963	0.040306	-3.74144
FCGR3A	1.087895	3.169447	2.597606	0.009713	0.040599	-3.74911
PRR15L	1.008487	2.536781	2.555625	0.010946	0.04495	-3.85511
AKR1B10	-1.55277	2.578215	-2.55518	0.01096	0.044997	-3.85622
INA	1.180774	2.423235	2.530643	0.011745	0.04771	-3.9174
GDPD3	1.03572	3.330919	2.484538	0.013355	0.053252	-4.0308
PDZK1IP1	1.271785	2.906233	2.395107	0.017047	0.065369	-4.24497
HTRA3	-1.02018	3.403223	-2.39146	0.017215	0.065935	-4.25355
AIM2	1.039861	2.417626	2.380045	0.01775	0.067662	-4.28029
CXCL9	1.193642	2.782506	2.354854	0.018983	0.071529	-4.33888
MAGEA4	1.04488	1.248857	2.277633	0.023244	0.08468	-4.51468
MUC1	1.040641	4.65665	2.270018	0.023706	0.08608	-4.53171
PKP1	1.130314	3.519094	2.269192	0.023756	0.086239	-4.53355
KRT6A	2.038051	4.264237	2.205443	0.027957	0.098414	-4.67386
S100A9	1.53564	8.530936	2.14699	0.032358	0.110983	-4.79908
HPGD	-1.24696	3.904555	-2.13313	0.033485	0.113996	-4.82828
UCA1	1.332275	3.718515	2.116392	0.034891	0.117837	-4.86332
EEF1A2	1.198753	2.979371	2.112736	0.035205	0.118596	-4.87093
COMP	1.094231	2.530623	2.086504	0.037528	0.124894	-4.92519
TCN1	1.035479	1.908978	2.041274	0.04184	0.136008	-5.01718
PI3	1.66281	3.741757	2.003438	0.045764	0.145972	-5.09262
KRT13	-1.77588	5.801145	-1.98983	0.047249	0.149709	-5.11941
S100A7	1.625262	3.073917	1.988209	0.047429	0.15008	-5.12259
