# Supplementary material for: Identification of Potential Biomarkers for Progression and Prognosis of Bladder Cancer by Comprehensive Bioinformatics Analysis
Source: J Oncol. 2022 Apr 19;2022:1802706. doi: 10.1155/2022/1802706 (PMC9042640; doi:10.1155/2022/1802706)
Supplement: Supplementary Materials — Supplementary Figure 1: WGCNA analysis of the TCGA dataset. Supplementary Figure 2: WGCNA analysis of the GSE133624 dataset. Supplementary Figures 3–7: clinical relevance of SMYD2, GAPDHP1, CILP, ATP1A2, and THSD4. Supplementary Table 1: primer sequences in the study. Supplementary Table 2: DEGs in the TCGA dataset. Supplementary Table 3: DEGs in the GSE133624 dataset. Supplementary Table 4: DEGs coexisting in the TCGA and GSE133624 datasets. Supplementary Table 5: feature genes were selected with the SVM-RFE algorithm. Supplementary Table 6: the correlation between the characteristic genes and immune cells. Supplementary Table 7: single-gene GSEA for prognostic genes. [file 1802706.f1.zip › 1802706.f1/Supplementary Table 3.pdf]

ID	baseMean	log2FoldChange	lfcSE	stat	p value	p adjust
PI16	2012.98795	-7.927798976	0.398844	-19.877	6.44E-88	1.57E-83
CFD	4693.18146	-5.036730888	0.290148	-17.3592	1.68E-67	2.05E-63
SCARA5	1908.61549	-5.535605002	0.355469	-15.5727	1.12E-54	9.08E-51
GLP2R	163.223674	-6.022981957	0.40525	-14.8624	5.78E-50	3.53E-46
FAM107A	895.797213	-4.829136689	0.333674	-14.4726	1.80E-47	8.81E-44
LRRN4CL	252.798769	-3.457454437	0.243822	-14.1802	1.21E-45	4.94E-42
CLEC3B	213.916065	-4.712521427	0.342061	-13.7769	3.51E-43	1.22E-39
GFRA1	277.830376	-4.180686004	0.306586	-13.6363	2.44E-42	7.44E-39
ESM1	377.519916	3.997829439	0.29871	13.38363	7.54E-41	2.04E-37
TCF21	801.960894	-3.791038849	0.284445	-13.3279	1.59E-40	3.89E-37
TNXB	2939.46577	-4.868650518	0.365692	-13.3135	1.93E-40	4.29E-37
PID1	427.411397	-3.422090207	0.257847	-13.2718	3.38E-40	6.86E-37
LINC0108	172.474618	-4.352318921	0.335376	-12.9774	1.64E-38	3.08E-35
C16orf89	207.985268	-5.053588794	0.391546	-12.9068	4.12E-38	7.19E-35
FHL1	5001.87395	-4.691466497	0.365383	-12.8398	9.81E-38	1.60E-34
PLAC9	381.322606	-4.53398939	0.353497	-12.8261	1.17E-37	1.79E-34
PGR	147.230011	-3.191926091	0.254401	-12.5468	4.14E-36	5.94E-33
SYNM	8582.4836	-5.514148101	0.440041	-12.531	5.05E-36	6.85E-33
ADCY5	655.218871	-4.388285661	0.355441	-12.346	5.12E-35	6.57E-32
C1QTNF7	396.234596	-3.581770088	0.290253	-12.3402	5.50E-35	6.71E-32
ITIH5	2107.10149	-3.295765051	0.26822	-12.2876	1.06E-34	1.23E-31
SYNPO2	9540.50594	-4.98343087	0.410448	-12.1414	6.37E-34	7.07E-31
MFAP4	5348.88841	-4.250508142	0.350351	-12.1321	7.14E-34	7.57E-31
F10	157.470041	-4.56712659	0.379323	-12.0402	2.18E-33	2.22E-30
ECRG4	236.584847	-5.387510905	0.447651	-12.0351	2.32E-33	2.27E-30
AFF3	290.446196	-3.767330372	0.314117	-11.9934	3.85E-33	3.61E-30
TMEM10(	160.291091	-3.965996682	0.333002	-11.9098	1.05E-32	9.51E-30
ADGRD1	496.375913	-4.92804113	0.415218	-11.8686	1.72E-32	1.50E-29
VIT	146.119691	-6.424776494	0.542959	-11.8329	2.64E-32	2.22E-29
CNN1	9459.31509	-5.515038875	0.468289	-11.777	5.13E-32	4.17E-29
MYOC	159.302687	-6.910815744	0.590706	-11.6993	1.29E-31	1.01E-28
TNXA	35.3230454	-4.438919267	0.383994	-11.5599	6.58E-31	5.02E-28
ADH1B	2279.84555	-5.632303292	0.487492	-11.5536	7.08E-31	5.23E-28
FENDRR	1038.72024	-3.097924457	0.270415	-11.4562	2.19E-30	1.57E-27
SCN7A	231.982974	-5.030564987	0.441192	-11.4022	4.08E-30	2.84E-27
FBLN5	1442.94942	-3.132156556	0.275842	-11.3549	7.01E-30	4.75E-27
HAND2	269.799037	-4.244915284	0.374404	-11.3378	8.53E-30	5.62E-27
INMT	247.845433	-3.210483738	0.284364	-11.2901	1.47E-29	9.44E-27
ANGPTL7	30.5018442	-6.431974523	0.570458	-11.2751	1.74E-29	1.09E-26
FOXF1	1105.60454	-2.916319797	0.260461	-11.1967	4.23E-29	2.53E-26
NPY6R	68.3849851	-7.053055043	0.629953	-11.1962	4.26E-29	2.53E-26
MYH11	67601.649	-5.5084975	0.492401	-11.187	4.72E-29	2.74E-26
LMOD1	4973.93236	-4.728809621	0.423563	-11.1643	6.09E-29	3.46E-26
NALCN-A	18.2588931	-3.780121743	0.338695	-11.1609	6.34E-29	3.52E-26
MATN2	2899.57849	-3.150158037	0.283472	-11.1128	1.09E-28	5.90E-26
CHRM2	301.908933	-5.216888251	0.469581	-11.1097	1.13E-28	5.97E-26
ADRB3	43.6430711	-5.203707507	0.469857	-11.0751	1.66E-28	8.61E-26
HAND2-A	208.774859	-4.659856545	0.421887	-11.0453	2.31E-28	1.17E-25
ITGA8	910.85421	-2.751302249	0.249129	-11.0437	2.35E-28	1.17E-25
ACTG2	21054.6145	-5.19354561	0.471542	-11.014	3.27E-28	1.60E-25
PRAC1	58.4006274	-6.098309064	0.553953	-11.0087	3.47E-28	1.66E-25

GPBAR1	66.2634897	-3.200077518	0.291189	-10.9897	4.28E-28	2.01E-25
TARID	10.1866967	-3.291450727	0.299568	-10.9873	4.40E-28	2.02E-25
PPP1R12E	5632.28032	-2.924102899	0.267284	-10.9401	7.41E-28	3.35E-25
HSPB6	3149.49502	-5.115781411	0.468339	-10.9233	8.92E-28	3.96E-25
ELANE	20.0227466	-4.154523242	0.380565	-10.9167	9.59E-28	4.18E-25
FLNC	6941.19889	-5.322430076	0.491895	-10.8203	2.76E-27	1.18E-24
MRGPRF	678.565197	-3.698503801	0.34206	-10.8124	3.01E-27	1.26E-24
GSN	17113.9056	-2.674527138	0.247852	-10.7908	3.80E-27	1.56E-24
SFRP1	1408.14898	-5.039784988	0.467117	-10.7891	3.87E-27	1.56E-24
CAVIN2	1331.14773	-3.283706749	0.304366	-10.7887	3.89E-27	1.56E-24
PGM5P4	31.6715788	-5.155227306	0.47946	-10.7522	5.79E-27	2.28E-24
PLPP3	3147.78977	-2.243973731	0.209017	-10.7358	6.91E-27	2.68E-24
PAMR1	889.419936	-3.018973454	0.281351	-10.7303	7.34E-27	2.80E-24
LRRC3B	22.6966693	-4.751204871	0.442992	-10.7253	7.75E-27	2.91E-24
PRELP	1488.45398	-4.2617977	0.397776	-10.7141	8.74E-27	3.23E-24
MYOCD	717.206864	-4.235309838	0.397259	-10.6613	1.54E-26	5.62E-24
ADAMTS	269.675932	-4.012740669	0.37669	-10.6526	1.70E-26	6.08E-24
HLF	235.681279	-3.900085886	0.366894	-10.63	2.16E-26	7.64E-24
SVEP1	864.10903	-3.223755212	0.303679	-10.6157	2.52E-26	8.79E-24
MBNL1-A	264.635593	-3.425043046	0.322719	-10.6131	2.59E-26	8.90E-24
ELN	2564.86358	-3.637584329	0.345275	-10.5353	5.94E-26	2.01E-23
KLC3	143.215496	3.38303253	0.32139	10.52625	6.54E-26	2.19E-23
NTRK3	205.965364	-4.61995505	0.439	-10.5238	6.71E-26	2.19E-23
LIMS2	1300.12963	-3.457832191	0.328579	-10.5236	6.73E-26	2.19E-23
LGI4	306.945828	-2.931479731	0.279184	-10.5002	8.62E-26	2.77E-23
ADAMTS	237.907821	-3.450296284	0.328757	-10.495	9.11E-26	2.89E-23
DES	35091.6158	-5.951400968	0.568889	-10.4614	1.30E-25	4.06E-23
LINC0108	4.79032356	-3.702757518	0.354456	-10.4463	1.52E-25	4.71E-23
ADAMTS	122.804219	-3.779265183	0.36275	-10.4184	2.04E-25	6.24E-23
SCN4B	179.017922	-2.923369818	0.280651	-10.4164	2.09E-25	6.29E-23
FBXL21P	30.399255	-5.295162039	0.508482	-10.4137	2.15E-25	6.39E-23
SORBS1	4541.4969	-4.049535314	0.388987	-10.4105	2.22E-25	6.53E-23
ADRA1A	7.82028252	-4.839527282	0.466094	-10.3832	2.96E-25	8.59E-23
HMGCLL	28.3502112	-2.735949497	0.263835	-10.3699	3.40E-25	9.76E-23
MIR1-1HC	139.818748	-4.796549875	0.463261	-10.3539	4.02E-25	1.14E-22
LTBP4	6075.52803	-2.633486393	0.255508	-10.3069	6.56E-25	1.84E-22
RCAN2	1011.73214	-3.067681316	0.298959	-10.2612	1.05E-24	2.90E-22
PPP1R14A	766.766743	-3.373594123	0.328782	-10.2609	1.06E-24	2.90E-22
SPARCL1	9884.68509	-2.830917084	0.27802	-10.1824	2.38E-24	6.44E-22
HAAO	244.0356	-2.476606004	0.244041	-10.1483	3.37E-24	9.04E-22
SGCA	238.688815	-4.140280222	0.408411	-10.1375	3.76E-24	9.99E-22
RBPMS2	563.666983	-4.207433542	0.415905	-10.1163	4.68E-24	1.23E-21
SLC2A4	252.384569	-4.373653763	0.434359	-10.0692	7.56E-24	1.96E-21
WFDC1	911.902965	-2.698866039	0.268147	-10.0649	7.90E-24	2.03E-21
ABI3BP	1579.43326	-3.424504145	0.340454	-10.0586	8.41E-24	2.14E-21
CPEB1	26.7138086	-3.853954873	0.383209	-10.0571	8.55E-24	2.15E-21
CNTN2	16.3008941	-3.90430681	0.389584	-10.0217	1.22E-23	3.05E-21
CRISPLD2	3582.68728	-3.003270757	0.299931	-10.0132	1.33E-23	3.29E-21
LRRC4B	125.807448	-2.98209379	0.297959	-10.0084	1.40E-23	3.42E-21
ODAD2	25.4863241	-3.7140476	0.371223	-10.0049	1.45E-23	3.50E-21
PYGM	182.926722	-3.426273784	0.342782	-9.99548	1.60E-23	3.82E-21
PODN	1799.8125	-3.311843223	0.331475	-9.99123	1.67E-23	3.94E-21

PGM5	1586.53329	-4.610553829	0.46458	-9.92414	3.27E-23	7.67E-21
ADAM33	1627.86128	-4.181434693	0.421507	-9.9202	3.40E-23	7.90E-21
PRRG3	69.8771374	-3.556696844	0.358704	-9.9154	3.57E-23	8.21E-21
SRPX	902.517518	-3.540516354	0.35771	-9.89773	4.26E-23	9.71E-21
STON1	691.164505	-2.540590038	0.256893	-9.88969	4.61E-23	1.04E-20
LINC0288	5.37292352	-4.297359141	0.43457	-9.88877	4.66E-23	1.04E-20
C11orf96	3417.49879	-3.192334568	0.32305	-9.88187	4.99E-23	1.11E-20
CSRP1	13486.1238	-2.877883024	0.291575	-9.87013	5.61E-23	1.23E-20
FXYD1	5.25148959	-3.951352936	0.400631	-9.86283	6.03E-23	1.31E-20
NR2F1	1234.31676	-2.447773736	0.249015	-9.82981	8.38E-23	1.81E-20
HPGDS	101.304834	-2.355014413	0.239662	-9.82641	8.67E-23	1.85E-20
SOX10	73.6078193	-5.01530723	0.510719	-9.8201	9.23E-23	1.96E-20
PCOLCE2	581.892962	-4.378446629	0.446145	-9.81395	9.81E-23	2.06E-20
ENPP6	43.146375	-3.345607976	0.341523	-9.79613	1.17E-22	2.44E-20
BARX1	53.4800649	6.744966487	0.689131	9.78764	1.27E-22	2.63E-20
MYL9	18962.9807	-3.737030515	0.382385	-9.77296	1.47E-22	3.02E-20
KANK2	4166.24921	-2.562227566	0.262213	-9.77156	1.49E-22	3.03E-20
CRB2	19.6886994	-3.938438917	0.40339	-9.76334	1.62E-22	3.26E-20
COL14A1	3889.01847	-3.404488222	0.348854	-9.75907	1.69E-22	3.37E-20
RBFOX3	337.874952	-6.23756507	0.639611	-9.75213	1.81E-22	3.58E-20
CYP4F24I	48.7254144	-3.631003684	0.372979	-9.73515	2.13E-22	4.20E-20
CCDC39	41.078279	-3.227211113	0.331677	-9.72997	2.25E-22	4.39E-20
POPDC2	258.643806	-3.379101477	0.350063	-9.65285	4.78E-22	9.26E-20
CFL2	1332.50283	-2.614808823	0.271275	-9.63895	5.47E-22	1.05E-19
SLIT3	3263.83593	-3.1006679	0.321937	-9.6313	5.90E-22	1.12E-19
FBXL22	157.082261	-3.43824703	0.357577	-9.6154	6.88E-22	1.30E-19
OSTN	18.1911316	-4.911930233	0.510971	-9.61294	7.05E-22	1.32E-19
ACTC1	3522.99427	-6.046162729	0.629217	-9.60902	7.32E-22	1.36E-19
EFCC1	230.292618	-2.460020077	0.256669	-9.5844	9.30E-22	1.72E-19
MAS1L	6.11720404	-5.167474153	0.540128	-9.56712	1.10E-21	2.02E-19
PTGS1	2997.88277	-3.997302633	0.418109	-9.56044	1.17E-21	2.14E-19
TNS1	9016.75428	-3.819030202	0.400692	-9.53109	1.56E-21	2.80E-19
BDKRB2	1185.58604	-2.55579892	0.268165	-9.53069	1.56E-21	2.80E-19
TACR3	42.2049469	-4.31408119	0.453118	-9.52087	1.72E-21	3.06E-19
AOC3	2043.77225	-3.243409406	0.340989	-9.51177	1.87E-21	3.31E-19
OR7E47P	79.6123597	-2.850912399	0.29985	-9.50779	1.95E-21	3.42E-19
PLP1	234.57033	-4.979102891	0.525031	-9.48345	2.46E-21	4.29E-19
TAGLN	25189.331	-3.674151015	0.38758	-9.47973	2.55E-21	4.40E-19
PDGFRA	2149.98487	-2.570023861	0.271123	-9.47918	2.56E-21	4.40E-19
MAMDC2	355.672029	-3.173076439	0.335382	-9.46107	3.05E-21	5.20E-19
PGM5P4-/	14.0724869	-4.433491301	0.469276	-9.44751	3.47E-21	5.88E-19
HSPB7	868.242751	-4.881272029	0.51785	-9.42603	4.26E-21	7.17E-19
ACTA2	17842.1087	-3.43556915	0.365422	-9.40166	5.37E-21	8.98E-19
RASGEF1	11.1518202	-3.687355718	0.392902	-9.38492	6.30E-21	1.05E-18
PCP4	1445.15049	-5.733688884	0.611371	-9.37842	6.70E-21	1.10E-18
LDB3	228.603382	-3.642380596	0.388806	-9.36813	7.38E-21	1.21E-18
GAS6	2730.19067	-2.741600139	0.293237	-9.34944	8.81E-21	1.43E-18
FGF10-AS	3.58847261	-4.197417849	0.449145	-9.34535	9.16E-21	1.48E-18
SOD3	2072.70738	-3.298239104	0.353424	-9.33224	1.04E-20	1.66E-18
BHMT2	175.288624	-3.249121957	0.348212	-9.33087	1.05E-20	1.67E-18
LINC0179	21.0626989	-3.344228779	0.35845	-9.32969	1.06E-20	1.68E-18
ZCCHC24	1526.05016	-2.588086154	0.277588	-9.32349	1.13E-20	1.77E-18

RGS13	61.0876327	-3.303252005	0.35438	-9.32122	1.15E-20	1.80E-18
ADAMTS:	315.701733	-2.130039719	0.228781	-9.3104	1.27E-20	1.98E-18
PLPPR4	225.835326	-3.085299145	0.331532	-9.30619	1.33E-20	2.05E-18
SALL3	12.3563677	-5.935628278	0.638643	-9.29413	1.48E-20	2.28E-18
BMP5	462.046779	-2.860497655	0.307895	-9.2905	1.54E-20	2.34E-18
MMP23B	68.8349782	-2.767222707	0.297929	-9.2882	1.57E-20	2.38E-18
GALNT17	160.99252	-4.284759701	0.46213	-9.27175	1.83E-20	2.76E-18
CPED1	657.035575	-2.811278356	0.304124	-9.24384	2.38E-20	3.56E-18
MYLK	9595.23015	-3.711523774	0.40173	-9.23886	2.49E-20	3.71E-18
FAIM2	177.079938	-4.542845016	0.492796	-9.21852	3.01E-20	4.43E-18
SCUBE1	176.350198	-2.88810784	0.313297	-9.21845	3.01E-20	4.43E-18
NXPH3	204.540123	-3.545084399	0.384884	-9.21079	3.24E-20	4.73E-18
IGSF10	288.216784	-4.078371927	0.443074	-9.20472	3.43E-20	4.98E-18
COX7A1	362.444228	-2.800985876	0.304408	-9.20141	3.53E-20	5.10E-18
GFRA2	81.0873547	-3.203154364	0.348497	-9.19134	3.88E-20	5.57E-18
KCNMB1	491.843859	-3.752362887	0.408486	-9.18603	4.08E-20	5.82E-18
FBLN2	3464.29167	-3.524548359	0.38442	-9.16848	4.80E-20	6.81E-18
ADGRG4	7.86663258	-6.296132352	0.687275	-9.16101	5.14E-20	7.25E-18
ACACB	952.321056	-2.155766161	0.235537	-9.15258	5.56E-20	7.80E-18
HPSE2	135.606939	-4.688007118	0.513125	-9.13619	6.47E-20	9.02E-18
CA9	391.198901	4.96625722	0.544106	9.127376	7.02E-20	9.73E-18
TRPA1	1061.30321	-2.682666855	0.294116	-9.12113	7.43E-20	1.03E-17
TNNT1	193.957328	4.642203506	0.509766	9.106537	8.51E-20	1.17E-17
KCNS2	23.6237995	-4.143814683	0.456022	-9.08688	1.02E-19	1.39E-17
PLCD4	161.750253	-3.518282986	0.388139	-9.06449	1.25E-19	1.70E-17
TPM2	15835.0892	-3.174721619	0.350789	-9.05023	1.43E-19	1.92E-17
CYGB	1333.66605	-2.467704241	0.273044	-9.03775	1.60E-19	2.14E-17
NFIA	2355.68269	-1.981330479	0.219342	-9.03308	1.67E-19	2.23E-17
CRYAB	949.407969	-3.185675554	0.353018	-9.02411	1.81E-19	2.40E-17
PTGIS	3186.5428	-4.112790725	0.456184	-9.01565	1.96E-19	2.58E-17
PRCD	60.2520309	-2.459317151	0.272883	-9.01233	2.02E-19	2.65E-17
OLFM1	400.490688	-3.218851011	0.3574	-9.0063	2.13E-19	2.77E-17
CSRNP1	2133.39194	-1.984087848	0.220379	-9.00309	2.19E-19	2.83E-17
MCM4	2437.42119	1.46983475	0.163284	9.001711	2.22E-19	2.85E-17
ITM2A	827.479904	-2.965875361	0.329803	-8.99286	2.41E-19	3.08E-17
TMEM11‹	850.654792	-2.760872534	0.307855	-8.96808	3.02E-19	3.84E-17
GPRASP1	556.536129	-1.966975772	0.219598	-8.95718	3.33E-19	4.21E-17
LAMC3	848.135369	-2.736570555	0.305637	-8.95368	3.44E-19	4.33E-17
AOX1	393.857503	-3.959971839	0.442591	-8.94725	3.64E-19	4.56E-17
CASQ2	587.883447	-5.170034012	0.579925	-8.915	4.88E-19	6.07E-17
KDM5B	2913.46039	1.150607541	0.129097	8.91272	4.98E-19	6.17E-17
ZBTB16	265.586383	-3.741118548	0.420165	-8.90392	5.39E-19	6.64E-17
CCDC80	6156.38579	-3.539757176	0.398437	-8.8841	6.44E-19	7.90E-17
GYPC	774.941551	-2.588024351	0.291352	-8.88281	6.52E-19	7.96E-17
MT1A	38.7192552	-4.09750898	0.462163	-8.86594	7.59E-19	9.21E-17
CBX7	1365.18707	-1.867572907	0.210744	-8.86183	7.87E-19	9.51E-17
FOXD3	21.21583	-4.283343048	0.483477	-8.85946	8.04E-19	9.63E-17
PAPPA	562.398842	-2.68720239	0.303318	-8.85936	8.05E-19	9.63E-17
KLF2	1730.69718	-2.667998591	0.301329	-8.8541	8.44E-19	1.00E-16
GPR17	3.9391545	-3.716813205	0.420054	-8.84843	8.88E-19	1.05E-16
DCN	19648.542	-3.101614395	0.3509	-8.83904	9.65E-19	1.14E-16
TPM1	13995.0668	-2.856521433	0.323235	-8.83728	9.81E-19	1.15E-16

ORC6	395.515974	2.398430155	0.27153	8.833012	1.02E-18	1.19E-16
CARMN	2220.48115	-3.994967207	0.453369	-8.81173	1.23E-18	1.42E-16
ADAMTS!	84.2736247	-5.169233427	0.586654	-8.81139	1.24E-18	1.42E-16
MYOM1	356.921261	-3.068579486	0.348304	-8.81005	1.25E-18	1.43E-16
PMP2	39.1651494	-4.793085824	0.544534	-8.80217	1.34E-18	1.53E-16
ADAMTS	5486.33711	-2.446904005	0.278409	-8.78888	1.51E-18	1.71E-16
LAMA2	1143.12218	-2.341570427	0.266677	-8.78053	1.63E-18	1.84E-16
KLF9	1637.35694	-2.284236156	0.260207	-8.77853	1.66E-18	1.86E-16
TMOD1	345.942972	-3.086228492	0.351749	-8.77396	1.72E-18	1.93E-16
AARD	57.4534519	-4.567370995	0.520627	-8.77283	1.74E-18	1.94E-16
NR4A1	12308.1799	-2.90321154	0.330966	-8.77195	1.76E-18	1.95E-16
TMEM88	174.354804	-1.897266675	0.216423	-8.76648	1.84E-18	2.04E-16
CYS1	119.515344	-2.976440192	0.339823	-8.75879	1.97E-18	2.17E-16
MUSK	17.3588975	-3.886646386	0.444482	-8.74421	2.25E-18	2.46E-16
ERVFRD-	12.3067255	-3.370588448	0.385701	-8.73886	2.35E-18	2.57E-16
CYTL1	114.58274	-3.19953619	0.366244	-8.73609	2.41E-18	2.62E-16
A2M	26770.9117	-2.611693657	0.299054	-8.73319	2.48E-18	2.67E-16
SORBS2	1569.79798	-2.910110478	0.333669	-8.72154	2.74E-18	2.95E-16
ITGA7	1329.23464	-2.975480588	0.341651	-8.70913	3.06E-18	3.28E-16
TNS2	1864.57837	-2.104282749	0.241834	-8.70137	3.28E-18	3.49E-16
PNMA8B	41.7487806	-2.583952665	0.29705	-8.69872	3.36E-18	3.56E-16
VIPR2	180.305867	-3.732677933	0.429215	-8.69653	3.42E-18	3.62E-16
OLFML3	1126.76578	-2.765860231	0.318265	-8.69044	3.61E-18	3.80E-16
CEP85	470.463795	1.109998717	0.127759	8.688226	3.68E-18	3.86E-16
IRAG1	1598.97277	-2.796590364	0.322042	-8.68392	3.82E-18	3.99E-16
FILIP1	523.092687	-3.328121262	0.383942	-8.66829	4.39E-18	4.56E-16
GPR146	133.637951	-1.863308176	0.21504	-8.66493	4.52E-18	4.67E-16
HEPACAI	2.75190476	-4.019300318	0.463889	-8.66436	4.54E-18	4.68E-16
PER1	2202.71011	-2.315923528	0.26764	-8.65313	5.01E-18	5.14E-16
RPS6KA2	1298.23233	-1.852796833	0.214327	-8.64472	5.39E-18	5.51E-16
LRFN5	145.590731	-3.437424778	0.397676	-8.64378	5.44E-18	5.53E-16
TEDC2	103.774446	2.25734136	0.261352	8.637159	5.76E-18	5.84E-16
SMOC2	917.581729	-3.189473936	0.369352	-8.63532	5.86E-18	5.91E-16
ROR1	206.053184	-2.620484298	0.303534	-8.63326	5.96E-18	5.99E-16
C1QTNF9	11.0529308	-3.717418011	0.431351	-8.61808	6.81E-18	6.81E-16
LYVE1	727.3145	-3.41395079	0.396309	-8.61437	7.03E-18	7.00E-16
LINC0193	15.7162949	-2.788768972	0.325025	-8.58017	9.47E-18	9.40E-16
LRRC2	124.462788	-3.934077026	0.458571	-8.579	9.57E-18	9.46E-16
PDE2A	403.923528	-2.643936157	0.308332	-8.57495	9.91E-18	9.74E-16
IL1RAPL1	26.5930345	-3.164403415	0.369039	-8.5747	9.93E-18	9.74E-16
EDNRB	940.784665	-2.38173872	0.278295	-8.55833	1.15E-17	1.12E-15
DACT3	416.711114	-3.385476402	0.39559	-8.55805	1.15E-17	1.12E-15
CALML3-	131.637667	4.475000472	0.522951	8.557201	1.16E-17	1.12E-15
P2RX1	1141.39617	-4.277832426	0.500025	-8.55524	1.18E-17	1.13E-15
PKIG	1544.35673	-2.007886974	0.235052	-8.54231	1.32E-17	1.26E-15
RUNDC3I	77.2656482	-2.108710963	0.247565	-8.51781	1.63E-17	1.56E-15
MIR100H(	978.835264	-3.072077453	0.361259	-8.50381	1.83E-17	1.75E-15
LINC0009	13.8798744	-3.175722874	0.373594	-8.50047	1.89E-17	1.79E-15
NDNF	250.588159	-2.792109337	0.329101	-8.48404	2.18E-17	2.06E-15
CD302	878.374997	-1.881481947	0.221948	-8.47713	2.31E-17	2.17E-15
SNED1	661.181559	-2.708732095	0.319662	-8.47373	2.38E-17	2.23E-15
NEXN	1988.31493	-3.176072552	0.375182	-8.46541	2.55E-17	2.38E-15

OXER1	88.9036639	-2.49481382	0.295102	-8.45407	2.81E-17	2.61E-15
SELENOI	1264.65648	1.028009993	0.121719	8.445741	3.02E-17	2.79E-15
PHYHIP	196.585914	-3.07583407	0.364475	-8.43908	3.20E-17	2.95E-15
VEGFD	69.2707767	-2.550520991	0.302261	-8.43814	3.22E-17	2.96E-15
FOLR2	411.866568	-2.36419363	0.2802	-8.43753	3.24E-17	2.96E-15
TDRD10	50.7634921	-2.48501657	0.294657	-8.43359	3.35E-17	3.05E-15
TRIP13	482.53595	2.622044711	0.311025	8.430329	3.45E-17	3.13E-15
PRKAR2E	790.418459	-2.176647258	0.258351	-8.42515	3.60E-17	3.26E-15
TSC22D3	3337.72631	-1.919624239	0.227934	-8.42184	3.71E-17	3.34E-15
CDC25C	99.855414	2.501552337	0.297275	8.414932	3.93E-17	3.53E-15
CCDC69	2081.61485	-2.30990461	0.27477	-8.40668	4.22E-17	3.77E-15
RASL12	452.735688	-2.791939857	0.332444	-8.39823	4.53E-17	4.04E-15
CYBRD1	2963.67404	-2.463354455	0.293358	-8.3971	4.58E-17	4.06E-15
AQP1	3047.1097	-2.536367702	0.302236	-8.39201	4.78E-17	4.21E-15
NDC1	972.7667	1.265283669	0.150945	8.382428	5.18E-17	4.55E-15
ASB2	394.709522	-3.172514468	0.379573	-8.35812	6.37E-17	5.55E-15
APOLD1	1067.51106	-2.030685666	0.243081	-8.35396	6.60E-17	5.73E-15
MT1M	80.377575	-3.44263545	0.412184	-8.35217	6.70E-17	5.80E-15
TAMALIN	682.866574	-2.345604661	0.280869	-8.35125	6.75E-17	5.82E-15
DIXDC1	869.69341	-2.323345195	0.278809	-8.33311	7.87E-17	6.77E-15
TONSL	578.610179	1.597472024	0.191719	8.332355	7.93E-17	6.79E-15
LIN9	202.415893	1.3919537	0.167127	8.328696	8.17E-17	6.97E-15
LINC0171	31.962348	2.334019238	0.280792	8.31228	9.39E-17	7.98E-15
PRICKLE2	719.064441	-1.993584026	0.240173	-8.30063	1.04E-16	8.78E-15
FGL2	2598.19295	-2.70510613	0.326507	-8.28498	1.18E-16	9.97E-15
TWIST2	73.6557271	-3.75333557	0.453284	-8.28032	1.23E-16	1.03E-14
KRT16	3174.98701	4.886083443	0.590461	8.275038	1.28E-16	1.08E-14
P2RY12	33.7044769	-2.756255238	0.333607	-8.26197	1.43E-16	1.20E-14
HIF3A	400.259508	-3.745691226	0.453787	-8.25429	1.53E-16	1.27E-14
LINC0248	8.98757833	-3.528126265	0.427788	-8.24736	1.62E-16	1.34E-14
TNFAIP8L	169.339932	-3.028305657	0.367387	-8.24282	1.68E-16	1.39E-14
METTL24	22.9763902	-3.075924554	0.373412	-8.23735	1.76E-16	1.45E-14
ADGRA2	2293.06037	-2.551697249	0.310203	-8.22589	1.94E-16	1.59E-14
HSD17B6	288.798644	-2.657433433	0.323059	-8.22585	1.94E-16	1.59E-14
IL6ST	4271.47632	-1.715289854	0.208756	-8.21672	2.09E-16	1.71E-14
FAM110D	130.645944	-2.522881052	0.307259	-8.21093	2.19E-16	1.79E-14
DMD	848.196362	-2.866257346	0.34918	-8.20854	2.24E-16	1.82E-14
MTBP	213.075247	1.633122419	0.199108	8.202201	2.36E-16	1.91E-14
RPL7P3	4.2736215	-5.083766735	0.620279	-8.19594	2.49E-16	2.00E-14
SNORD11	12.7520692	-3.739706367	0.456365	-8.19455	2.52E-16	2.02E-14
DNMT3B	355.347979	1.986608884	0.242437	8.194345	2.52E-16	2.02E-14
ANLN	765.967067	2.561214	0.312593	8.193434	2.54E-16	2.02E-14
FRMD6-A	7.20072244	-4.200388008	0.513027	-8.18745	2.67E-16	2.12E-14
MIR133A1	13.2462393	-4.169916648	0.509687	-8.18133	2.81E-16	2.22E-14
CIDEC	8.24340126	-5.152679191	0.630018	-8.17862	2.87E-16	2.27E-14
NFIX	2515.45429	-2.457794825	0.300729	-8.17279	3.01E-16	2.37E-14
RNF112	112.014151	-2.279570217	0.278964	-8.17155	3.04E-16	2.39E-14
ATAD2	1266.74275	1.636724247	0.200584	8.159806	3.36E-16	2.62E-14
TPSAB1	1022.71424	-2.94586273	0.361277	-8.15403	3.52E-16	2.74E-14
BIN1	1122.37517	-2.453347721	0.301003	-8.15057	3.62E-16	2.82E-14
FXYD6	682.409743	-3.333415705	0.409517	-8.13987	3.96E-16	3.07E-14
SPEG	1468.65666	-3.39491541	0.417125	-8.13885	3.99E-16	3.08E-14

NPR1	311.842465	-2.571794381	0.316114	-8.13566	4.10E-16	3.15E-14
NXPH4	105.23777	4.153266413	0.510789	8.131087	4.25E-16	3.27E-14
JAM2	570.507358	-2.746379332	0.337845	-8.1291	4.32E-16	3.31E-14
RASA3-IT	6.88161012	-2.839181808	0.34943	-8.12519	4.47E-16	3.41E-14
TM4SF19	69.6099508	5.231004214	0.643899	8.123955	4.51E-16	3.43E-14
DARS2	611.754539	1.102361347	0.135756	8.120191	4.65E-16	3.53E-14
RPH3AL-/	16.1123227	-2.748722448	0.338684	-8.11589	4.82E-16	3.64E-14
MEF2C-A	26.8762383	-2.789400023	0.344694	-8.09241	5.85E-16	4.41E-14
CSGALN/	746.04666	-2.50061471	0.309115	-8.08959	5.99E-16	4.50E-14
FNBP1	2444.48904	-1.860634746	0.230241	-8.08124	6.41E-16	4.80E-14
LINC0177	8.23781603	-3.291860459	0.407938	-8.06951	7.06E-16	5.26E-14
AURKA	396.948827	2.132256278	0.26424	8.069384	7.07E-16	5.26E-14
CNRIP1	347.029494	-2.089058937	0.258973	-8.06669	7.22E-16	5.36E-14
FGF10	82.5486795	-4.088514208	0.507365	-8.05833	7.73E-16	5.72E-14
PREX2	372.417955	-1.928014099	0.239307	-8.05664	7.84E-16	5.78E-14
MEX3A	1063.17161	2.694198422	0.334526	8.053768	8.03E-16	5.90E-14
CLIP3	740.971697	-2.539733175	0.315367	-8.05325	8.06E-16	5.91E-14
TNFSF12	389.784574	-1.631421673	0.20266	-8.05002	8.28E-16	6.05E-14
NEGR1	890.042583	-3.411065163	0.424441	-8.0366	9.24E-16	6.73E-14
MCM3AP-	89.152213	1.741928106	0.216903	8.030904	9.68E-16	7.03E-14
CD34	1666.78726	-2.128790164	0.265098	-8.0302	9.73E-16	7.05E-14
CALML3	903.064551	5.377626559	0.670449	8.020928	1.05E-15	7.58E-14
DNAJB5	493.976853	-2.463622002	0.307264	-8.01794	1.08E-15	7.74E-14
PSRC1	171.387404	1.654578667	0.206464	8.013879	1.11E-15	7.95E-14
SMYD2	1042.99048	1.025164637	0.127924	8.013871	1.11E-15	7.95E-14
HIC1	738.175401	-2.419276362	0.301929	-8.01273	1.12E-15	8.01E-14
IGF1	1013.58625	-3.153726804	0.393714	-8.0102	1.15E-15	8.13E-14
PLK1	453.483601	1.85039013	0.231007	8.010093	1.15E-15	8.13E-14
EME1	111.691164	2.399663078	0.299645	8.008349	1.16E-15	8.22E-14
MKX	81.8378369	-3.774328268	0.471575	-8.00366	1.21E-15	8.52E-14
KCNAB1	57.2675885	-2.338210109	0.292191	-8.00232	1.22E-15	8.58E-14
KCNB1	51.9726265	-4.491718336	0.56132	-8.00206	1.22E-15	8.58E-14
RXRG	18.6820747	-3.710028898	0.46368	-8.00127	1.23E-15	8.61E-14
TESMIN	125.63859	1.783000448	0.223146	7.990272	1.35E-15	9.39E-14
SLC44A5	512.488544	3.079378394	0.386027	7.977116	1.50E-15	1.04E-13
MASP1	169.36144	-3.211060441	0.402565	-7.9765	1.51E-15	1.04E-13
GBP6	156.538584	3.578290456	0.449073	7.968175	1.61E-15	1.11E-13
EXO1	199.326303	2.653311857	0.333108	7.965321	1.65E-15	1.14E-13
OLFML1	311.092234	-2.734697709	0.344064	-7.94823	1.89E-15	1.30E-13
OGN	880.350273	-4.836459536	0.608603	-7.94682	1.91E-15	1.31E-13
LPP	11577.7614	-2.056831623	0.258906	-7.94433	1.95E-15	1.33E-13
CLDN5	644.352596	-2.788029688	0.351355	-7.93507	2.10E-15	1.43E-13
GSTM5	200.900481	-2.98029733	0.376018	-7.92594	2.26E-15	1.54E-13
CPXM2	899.468716	-3.433409623	0.433188	-7.92591	2.26E-15	1.54E-13
MORC1	5.24384379	-5.142726036	0.649244	-7.92109	2.35E-15	1.59E-13
CCN1	10177.8035	-2.614439374	0.330093	-7.92031	2.37E-15	1.59E-13
CCT5	6599.76443	1.396296271	0.17634	7.918184	2.41E-15	1.62E-13
SLC47A1I	1.77337392	-3.813582021	0.481892	-7.91376	2.50E-15	1.67E-13
INTS8	1509.12814	1.019295329	0.128801	7.913727	2.50E-15	1.67E-13
FGFR1	2204.10002	-2.53862413	0.321078	-7.90657	2.65E-15	1.76E-13
PSD	460.639221	-2.858715174	0.361757	-7.90231	2.74E-15	1.82E-13
DIO3OS	248.292875	-2.986741678	0.378086	-7.89964	2.80E-15	1.85E-13

CRTAP	2988.59413	-1.230758358	0.15581	-7.89912	2.81E-15	1.85E-13
CDO1	54.6715124	-3.324873242	0.420977	-7.89799	2.83E-15	1.86E-13
NR4A3	1259.92362	-3.113972821	0.394479	-7.89389	2.93E-15	1.92E-13
DUSP1	10247.8392	-2.105240013	0.266835	-7.88967	3.03E-15	1.98E-13
C21orf58	275.800792	1.57034857	0.199253	7.881192	3.24E-15	2.12E-13
MSRB3	1804.13665	-2.97558387	0.377591	-7.88044	3.26E-15	2.12E-13
KIF20A	377.494168	2.41593886	0.306702	7.877157	3.35E-15	2.17E-13
LINC0214	7.60041478	-3.171421855	0.402988	-7.86977	3.55E-15	2.30E-13
GYPE	8.90281774	-2.729673846	0.346991	-7.86671	3.64E-15	2.35E-13
AVPR2	21.0716116	-2.494727405	0.317304	-7.86226	3.77E-15	2.43E-13
ABCA8	830.557924	-2.682798794	0.34131	-7.8603	3.83E-15	2.46E-13
DMRTA2	58.015687	4.284026444	0.545199	7.857736	3.91E-15	2.51E-13
HMCN2	334.618034	-3.154438754	0.401811	-7.85056	4.14E-15	2.64E-13
LHX5	18.5569058	4.204817975	0.535621	7.850358	4.15E-15	2.64E-13
TPSG1	7.22808123	-2.862128407	0.364756	-7.8467	4.27E-15	2.71E-13
PPFIA4	185.772669	2.163632422	0.275914	7.841699	4.44E-15	2.81E-13
WDFY3-A	101.581605	-1.256291246	0.160249	-7.83961	4.52E-15	2.85E-13
PDK4	2035.61887	-2.788137028	0.355656	-7.83942	4.53E-15	2.85E-13
MAP3K20	2836.76037	-2.044960416	0.260903	-7.83802	4.58E-15	2.87E-13
MGLL	1736.94908	-2.168283523	0.276854	-7.83186	4.81E-15	3.01E-13
TACR2	122.886636	-3.785648982	0.483563	-7.82867	4.93E-15	3.08E-13
CDCA4	438.1046	1.220258388	0.156035	7.820411	5.27E-15	3.28E-13
MAP3K21	233.772486	1.639455137	0.20965	7.819954	5.28E-15	3.28E-13
GJB6	1692.20264	3.216297918	0.411685	7.812516	5.61E-15	3.46E-13
CCDC150	119.255949	2.157998509	0.276407	7.807314	5.84E-15	3.60E-13
SLC38A1	4427.7128	1.116118918	0.142965	7.806959	5.86E-15	3.60E-13
TGFBR2	3951.92512	-1.430562289	0.183347	-7.80248	6.07E-15	3.71E-13
ENPP3	27.8010069	-2.905279666	0.373062	-7.78766	6.83E-15	4.16E-13
C1QTNF2	74.212393	-2.752688579	0.353815	-7.78002	7.25E-15	4.41E-13
MREG	183.354191	1.179462629	0.151805	7.769614	7.87E-15	4.78E-13
TTK	384.004569	2.6196779	0.337555	7.760741	8.44E-15	5.11E-13
CRY2	916.459259	-1.05526629	0.136015	-7.75845	8.60E-15	5.18E-13
HMGN2P	10.8726579	-4.864320652	0.627339	-7.75389	8.91E-15	5.36E-13
UBE2T	388.793627	2.056750176	0.265334	7.751542	9.08E-15	5.44E-13
CEP72	205.540891	1.593985333	0.205788	7.745747	9.50E-15	5.68E-13
MMP13	888.60538	4.205509838	0.543457	7.738447	1.01E-14	6.00E-13
NLN	617.985019	1.068366862	0.138228	7.729026	1.08E-14	6.45E-13
ADRA2A	297.243592	-3.03766489	0.393055	-7.72835	1.09E-14	6.47E-13
TPSB2	1318.94714	-2.955646162	0.382459	-7.728	1.09E-14	6.47E-13
NEK2	291.173298	2.724569368	0.352677	7.725401	1.12E-14	6.59E-13
C6orf223	63.6635322	3.864637974	0.500811	7.716766	1.19E-14	7.03E-13
CENPF	1475.08644	2.323500252	0.301844	7.697691	1.39E-14	8.15E-13
WNT2B	194.629223	-1.683954851	0.21877	-7.69737	1.39E-14	8.15E-13
WDHD1	433.742465	1.720300676	0.223558	7.69511	1.41E-14	8.27E-13
MMP27	3.29432867	-4.460613955	0.579837	-7.69287	1.44E-14	8.40E-13
LGI1	10.4572415	-3.470162889	0.452114	-7.67541	1.65E-14	9.60E-13
SKP2	721.245896	1.393331623	0.181602	7.672437	1.69E-14	9.78E-13
PDGFD	566.527575	-1.803844402	0.23529	-7.66646	1.77E-14	1.02E-12
RASGRP2	501.309061	-2.822836983	0.368303	-7.66444	1.80E-14	1.04E-12
ACTA2-A	23.74613	-2.750778093	0.359053	-7.66121	1.84E-14	1.06E-12
ALKAL2	55.1291699	-2.577069719	0.336403	-7.66067	1.85E-14	1.06E-12
C5orf66-A	44.8549356	-2.783700835	0.363667	-7.65453	1.94E-14	1.11E-12

OAF	1301.45913	-1.622577328	0.212075	-7.65094	2.00E-14	1.14E-12
MXRA7	2864.58237	-1.834766363	0.240588	-7.62618	2.42E-14	1.36E-12
HPD	16.136323	-3.051308764	0.400111	-7.62615	2.42E-14	1.36E-12
LCN6	6.09988922	-4.145827536	0.543885	-7.62262	2.49E-14	1.39E-12
RECQL4	525.487072	2.019262437	0.26494	7.62157	2.51E-14	1.40E-12
E2F7	167.492329	1.90166787	0.249692	7.616055	2.62E-14	1.46E-12
KIF14	269.001462	2.298732377	0.301864	7.615129	2.63E-14	1.47E-12
GREM2	242.511295	-3.403082404	0.447047	-7.61235	2.69E-14	1.50E-12
PTGFR	269.088381	-3.21460491	0.422319	-7.6118	2.70E-14	1.50E-12
CIT	396.192272	1.728586078	0.227233	7.607119	2.80E-14	1.54E-12
RBPMS	3546.08245	-1.527598676	0.200813	-7.60706	2.80E-14	1.54E-12
FAM227A	204.582016	1.778581473	0.23418	7.594932	3.08E-14	1.69E-12
ASPM	540.060437	2.386767382	0.314258	7.594925	3.08E-14	1.69E-12
SMTN	3700.13268	-2.234633229	0.294325	-7.59241	3.14E-14	1.72E-12
SOX6	126.271408	-1.6018101	0.211017	-7.5909	3.18E-14	1.73E-12
BUB1	542.204927	1.988822306	0.262028	7.590117	3.20E-14	1.74E-12
CENPO	294.719688	1.085126809	0.142976	7.589569	3.21E-14	1.74E-12
ATP1B2	102.401973	-2.629133588	0.346449	-7.58881	3.23E-14	1.75E-12
ATP1A2	538.724986	-3.800896792	0.501467	-7.57955	3.47E-14	1.88E-12
KIF2C	477.148747	2.270274001	0.299734	7.574293	3.61E-14	1.95E-12
P2RY14	173.056724	-2.741247999	0.361977	-7.57298	3.65E-14	1.96E-12
TCF23	2.91133495	-4.839327366	0.639125	-7.5718	3.68E-14	1.98E-12
LINC0179	2.39061224	-4.357861759	0.575547	-7.57168	3.68E-14	1.98E-12
LHFPL6	1034.8923	-2.025304819	0.267624	-7.56774	3.80E-14	2.03E-12
LMF1-AS1	4.36967787	-2.850563817	0.376695	-7.56731	3.81E-14	2.03E-12
KNSTRN	370.78237	1.461257203	0.193138	7.565866	3.85E-14	2.05E-12
RYR3-DT	2.95578445	-4.089516844	0.540854	-7.56121	3.99E-14	2.12E-12
NAV3	143.429559	-2.715222192	0.359663	-7.54935	4.37E-14	2.32E-12
SOX17	201.517291	-2.478091277	0.328428	-7.54532	4.51E-14	2.39E-12
SLC35F1	36.1286783	-2.23810055	0.296656	-7.54442	4.54E-14	2.40E-12
RAD54L	224.089494	2.281285258	0.30286	7.532478	4.98E-14	2.62E-12
B3GNT4	17.4237143	2.003358913	0.265976	7.532092	4.99E-14	2.62E-12
RNU1-149	5.16801928	-2.978044803	0.395521	-7.52942	5.10E-14	2.67E-12
CENPI	120.763974	2.028933982	0.269544	7.527282	5.18E-14	2.71E-12
DMGDH	38.1431072	-1.695910573	0.225464	-7.52186	5.40E-14	2.82E-12
PALLD	7889.46658	-2.33737608	0.310799	-7.52053	5.46E-14	2.84E-12
MAST1	88.8912507	3.40250743	0.45286	7.513374	5.76E-14	2.98E-12
DTL	292.024346	2.168196549	0.288582	7.513288	5.77E-14	2.98E-12
SLMAP	3603.80795	-1.649937088	0.219775	-7.5074	6.03E-14	3.11E-12
GGTA1	288.132244	-2.125824167	0.283255	-7.50497	6.14E-14	3.16E-12
RECK	385.057254	-2.085970454	0.278148	-7.4995	6.41E-14	3.28E-12
ZWILCH	572.318461	1.198275958	0.159853	7.496094	6.57E-14	3.36E-12
GINS1	370.854254	2.067542992	0.2761	7.488379	6.97E-14	3.56E-12
ARHGAP2	114.925905	-2.490131701	0.332561	-7.48774	7.01E-14	3.57E-12
BOC	685.989904	-2.852799697	0.381279	-7.48217	7.31E-14	3.72E-12
SQLE	1740.3506	1.880529549	0.251417	7.479729	7.45E-14	3.78E-12
KCNIP1-C	8.71712038	-4.001757821	0.535204	-7.47707	7.60E-14	3.85E-12
RGS5	5683.75023	-2.040708367	0.272938	-7.47681	7.61E-14	3.85E-12
FZD7	764.437823	-1.757102448	0.235046	-7.47556	7.69E-14	3.88E-12
PTTG1	577.155347	2.092068528	0.279882	7.47481	7.73E-14	3.89E-12
SLC39A4	503.210798	1.546078291	0.20687	7.47367	7.80E-14	3.92E-12
HMMR	376.018059	2.13599327	0.286023	7.467898	8.15E-14	4.08E-12

GAS7	1081.15648	-2.660058982	0.356473	-7.46216	8.51E-14	4.26E-12
C6	18.9549857	-3.277120885	0.439428	-7.4577	8.80E-14	4.39E-12
EGR3	1063.06651	-2.84097034	0.381123	-7.45422	9.04E-14	4.50E-12
TSPAN2	555.673553	-2.191927723	0.294125	-7.45237	9.17E-14	4.56E-12
C12orf73	226.699005	1.060613421	0.142426	7.446789	9.56E-14	4.74E-12
MEG8	68.5088296	-3.107231147	0.417321	-7.44567	9.65E-14	4.77E-12
SPRY1	1918.34525	-1.700923078	0.228489	-7.44424	9.75E-14	4.82E-12
TOX2	213.655657	-2.167724189	0.291359	-7.44006	1.01E-13	4.95E-12
IQGAP3	604.518817	2.212171976	0.297338	7.439935	1.01E-13	4.95E-12
ARHGEF3	161.260005	2.006843286	0.269777	7.438888	1.02E-13	4.98E-12
CHRNA5	152.501002	1.605211882	0.216023	7.43076	1.08E-13	5.28E-12
CALD1	21543.9349	-2.743971842	0.369307	-7.43005	1.09E-13	5.30E-12
GATA5	90.1314068	-3.980041397	0.535825	-7.42788	1.10E-13	5.38E-12
SYNC	359.171734	-2.314913671	0.311718	-7.4263	1.12E-13	5.43E-12
PACC1	337.012529	1.340935733	0.180593	7.425201	1.13E-13	5.46E-12
PDZRN3	755.808443	-2.757556678	0.372264	-7.40752	1.29E-13	6.23E-12
CDC42EP:	467.890831	-1.713121577	0.231282	-7.40708	1.29E-13	6.24E-12
TSPAN11	419.745602	-2.583318668	0.349041	-7.40119	1.35E-13	6.51E-12
RCOR2	106.826356	1.915853255	0.259013	7.396751	1.40E-13	6.72E-12
JUND	6625.49893	-1.352342834	0.182923	-7.39297	1.44E-13	6.90E-12
ANGPTL5	4.64062676	-4.292580902	0.580962	-7.38875	1.48E-13	7.11E-12
PLA2G5	78.7908837	-3.450525495	0.467522	-7.38046	1.58E-13	7.53E-12
RFC5	613.215379	1.234462954	0.167295	7.378973	1.60E-13	7.60E-12
LINC0189	11.3578822	-4.75786685	0.644801	-7.37882	1.60E-13	7.60E-12
PTGDS	2476.02017	-3.321683869	0.450205	-7.37817	1.60E-13	7.62E-12
PDLIM3	2750.02139	-2.888085332	0.391934	-7.36881	1.72E-13	8.16E-12
FCER1A	192.381989	-2.746261495	0.372722	-7.36812	1.73E-13	8.18E-12
ZNF117	443.347947	1.941171148	0.263497	7.366945	1.75E-13	8.24E-12
RASSF3	1794.76435	-1.578080492	0.214251	-7.36558	1.76E-13	8.31E-12
AATBC	545.443837	2.787985551	0.378677	7.362443	1.81E-13	8.49E-12
CCNB1	848.240472	2.088599696	0.283737	7.361043	1.82E-13	8.56E-12
FANCD2	567.178506	1.275101249	0.173227	7.360855	1.83E-13	8.56E-12
MCM10	282.207768	2.752957816	0.374199	7.35694	1.88E-13	8.80E-12
TIMELES:	892.636594	1.292251977	0.175766	7.352109	1.95E-13	9.10E-12
TBC1D31	257.572522	1.101406443	0.150071	7.339221	2.15E-13	1.00E-11
TROAP	366.796486	2.293350788	0.312678	7.33455	2.22E-13	1.03E-11
NKAPL	19.9340134	-2.183029446	0.297676	-7.33358	2.24E-13	1.04E-11
RPL7L1P9	17.4052282	-3.035735877	0.414012	-7.33248	2.26E-13	1.05E-11
GATA6	399.815162	-2.645922797	0.361051	-7.3284	2.33E-13	1.07E-11
SORCS1	152.219966	-3.795365104	0.517902	-7.32835	2.33E-13	1.07E-11
NCAPD3	725.292624	1.16437022	0.158993	7.323416	2.42E-13	1.11E-11
PKDCC	376.934682	-3.14062879	0.428904	-7.32245	2.43E-13	1.12E-11
SBSPON	763.912043	-3.211041806	0.438597	-7.32117	2.46E-13	1.13E-11
TSPYL2	2118.39807	-1.245663897	0.170179	-7.31971	2.48E-13	1.14E-11
FOSB	10660.4109	-2.711113855	0.370445	-7.31854	2.51E-13	1.15E-11
RPS20P22	39.2292937	-3.207039309	0.438351	-7.31615	2.55E-13	1.16E-11
UBE2C	914.021846	2.636564024	0.360379	7.316087	2.55E-13	1.16E-11
PLK4	264.354002	1.686239968	0.230592	7.312661	2.62E-13	1.19E-11
CD1C	115.678153	-2.59057236	0.35428	-7.31221	2.63E-13	1.19E-11
LCN10	17.5526309	-4.141938845	0.566558	-7.31071	2.66E-13	1.20E-11
MAGI2-A:	479.862166	-2.135083513	0.292176	-7.30752	2.72E-13	1.23E-11
BRIP1	260.508695	2.29318377	0.313884	7.305839	2.76E-13	1.24E-11

GALNT16	94.8040011	-3.047539302	0.417385	-7.30151	2.85E-13	1.28E-11
RUFY1-A!	54.8071411	-2.096339652	0.28728	-7.2972	2.94E-13	1.32E-11
SEL1L2	6.24908246	-3.841041415	0.526815	-7.29106	3.08E-13	1.37E-11
PRDM6	162.674165	-2.577802437	0.353846	-7.28509	3.21E-13	1.43E-11
CC2D2A	381.235466	-1.113448028	0.152857	-7.28425	3.23E-13	1.44E-11
LINC0198	2.60029488	-3.398393536	0.466833	-7.27967	3.35E-13	1.48E-11
EFEMP1	3135.72152	-3.145625669	0.432307	-7.27637	3.43E-13	1.52E-11
LINC0215	37.6580716	5.606285355	0.770752	7.273782	3.50E-13	1.55E-11
PXDC1	983.758424	-1.400222239	0.192533	-7.27264	3.53E-13	1.55E-11
SRD5A1	368.262976	1.657055608	0.227868	7.272014	3.54E-13	1.56E-11
TGFB1I1	1233.22715	-2.139669033	0.294587	-7.26329	3.78E-13	1.65E-11
CADM3	444.462657	-4.265003789	0.587268	-7.26245	3.80E-13	1.66E-11
MIR433	1.94948245	-3.644634524	0.501871	-7.26209	3.81E-13	1.66E-11
ZBTB20	595.981675	-1.785747036	0.245905	-7.26193	3.82E-13	1.66E-11
MAPK13	1735.23063	1.24685049	0.171733	7.260409	3.86E-13	1.68E-11
TRABD2E	6.91054185	-2.912978582	0.401338	-7.25817	3.92E-13	1.70E-11
RAB23	1129.89328	-2.052019301	0.282961	-7.25196	4.11E-13	1.78E-11
RNASEH2	515.671493	1.47441911	0.203325	7.251554	4.12E-13	1.78E-11
EGR1	10078.7585	-2.203201317	0.30384	-7.25119	4.13E-13	1.78E-11
VSTM4	185.875946	-2.303878269	0.317735	-7.25095	4.14E-13	1.78E-11
PPAT	453.246627	1.227159239	0.169254	7.250412	4.16E-13	1.79E-11
ARID5A	1235.93527	-1.772392237	0.244535	-7.24802	4.23E-13	1.81E-11
ACOX2	137.951873	-2.509039291	0.346196	-7.24746	4.25E-13	1.82E-11
PKD1L2	77.9079019	-2.385446765	0.32933	-7.24334	4.38E-13	1.87E-11
RYR3	128.771574	-3.461095411	0.477882	-7.24257	4.40E-13	1.88E-11
TICRR	138.090746	2.394038877	0.330708	7.239136	4.52E-13	1.92E-11
GCSAML	40.1484239	-2.603365637	0.359683	-7.23795	4.56E-13	1.93E-11
ANO2	42.4509344	-1.348118861	0.186324	-7.23535	4.64E-13	1.96E-11
RBMS3	620.204212	-2.040540731	0.282095	-7.23353	4.71E-13	1.99E-11
NFIC	2934.26576	-1.638838361	0.226871	-7.22366	5.06E-13	2.13E-11
CORO2B	92.4461125	-2.346469183	0.324838	-7.22351	5.07E-13	2.13E-11
ALDH1B1	1946.82138	-2.281878587	0.315917	-7.22303	5.08E-13	2.14E-11
PDE1C	178.560979	-2.48250288	0.343705	-7.22276	5.09E-13	2.14E-11
BDKRB1	104.609634	-2.421496425	0.335324	-7.22136	5.15E-13	2.15E-11
CDCA2	145.928144	2.142439031	0.296947	7.214888	5.40E-13	2.25E-11
TMC2	5.09881663	-3.338492297	0.462836	-7.21312	5.47E-13	2.28E-11
FGF2	358.673917	-2.580268387	0.357731	-7.21286	5.48E-13	2.28E-11
KIF4A	348.929591	2.173198549	0.301404	7.210245	5.59E-13	2.32E-11
TOP2A	3205.35756	2.306029205	0.319887	7.208886	5.64E-13	2.34E-11
CCN2	9265.28267	-2.587621321	0.359039	-7.20707	5.72E-13	2.36E-11
ST6GALN	96.4680044	-1.775654705	0.24639	-7.20669	5.73E-13	2.37E-11
FLNA	52075.0471	-2.574564174	0.357303	-7.20555	5.78E-13	2.38E-11
ACY1	107.535383	1.457491266	0.202281	7.205291	5.79E-13	2.38E-11
KREMEN	53.2280776	3.847570563	0.534101	7.203828	5.85E-13	2.41E-11
RERG	270.700487	-2.071014054	0.287508	-7.20333	5.88E-13	2.41E-11
KIF15	319.419745	2.342183538	0.325483	7.196028	6.20E-13	2.54E-11
TCF24	12.5330328	3.18158368	0.442236	7.194304	6.28E-13	2.57E-11
MELK	423.798806	2.487451207	0.345896	7.191337	6.42E-13	2.62E-11
PPP1R1A	22.3124128	-3.487787142	0.485123	-7.18949	6.50E-13	2.65E-11
DNAH9	5.68979027	-2.091826488	0.291172	-7.18417	6.76E-13	2.75E-11
GGH	1031.57107	2.571471784	0.358031	7.18225	6.86E-13	2.78E-11
TBX1	557.427517	2.339623949	0.32577	7.181821	6.88E-13	2.79E-11

ZFPM2	195.682757	-3.069688544	0.427489	-7.18075	6.93E-13	2.81E-11
MIR1-1HC	4.92317521	-4.847012001	0.675171	-7.17894	7.03E-13	2.84E-11
FERMT2	2066.17703	-2.118187304	0.295072	-7.17855	7.05E-13	2.84E-11
MEF2C	994.056512	-1.896334753	0.264322	-7.17433	7.27E-13	2.93E-11
BCL2	531.011651	-1.632562332	0.227576	-7.17371	7.30E-13	2.93E-11
DNA2	308.245652	1.717950592	0.239498	7.173132	7.33E-13	2.94E-11
NOSTRIN	386.968801	-1.598831446	0.222916	-7.17234	7.37E-13	2.95E-11
BAG2	689.135774	-2.115536974	0.295162	-7.16739	7.64E-13	3.05E-11
PTH1R	76.5336593	-2.292999159	0.320652	-7.15105	8.61E-13	3.42E-11
AOC4P	36.9899582	-2.691809042	0.376465	-7.15022	8.66E-13	3.44E-11
HROB	104.114053	1.616854623	0.226261	7.145967	8.94E-13	3.54E-11
PMP22	2747.18111	-2.240638875	0.313804	-7.14024	9.32E-13	3.68E-11
XRCC2	231.763939	2.083979014	0.292028	7.136235	9.59E-13	3.79E-11
PLN	893.04478	-3.669842249	0.514539	-7.13229	9.87E-13	3.89E-11
MTFR1	763.328479	1.054314469	0.147849	7.131005	9.96E-13	3.92E-11
VASN	639.768374	-2.102850988	0.295037	-7.12741	1.02E-12	4.02E-11
CD1E	59.8700712	-2.592730062	0.363887	-7.12509	1.04E-12	4.08E-11
FILIP1L	4258.50887	-2.508123637	0.352176	-7.1218	1.07E-12	4.17E-11
HMGB3	1684.6519	1.561027631	0.219208	7.121214	1.07E-12	4.18E-11
SNORA72	60.7510473	1.415738048	0.198837	7.120107	1.08E-12	4.21E-11
ECT2	945.753494	1.969201644	0.276584	7.119716	1.08E-12	4.22E-11
DCAF13	1660.11778	1.01947918	0.143203	7.119141	1.09E-12	4.23E-11
SLC9A9	379.328637	-1.819525866	0.255615	-7.11823	1.09E-12	4.25E-11
TSPAN4	720.640148	-1.793599646	0.251993	-7.11765	1.10E-12	4.26E-11
KCNK2	75.4258645	-3.327729562	0.468035	-7.11	1.16E-12	4.49E-11
NR4A2	1984.94022	-2.107321878	0.296431	-7.10898	1.17E-12	4.51E-11
CSE1L	2756.75579	1.067965708	0.150228	7.108955	1.17E-12	4.51E-11
SRF	2182.67621	-1.162269245	0.163511	-7.10822	1.18E-12	4.53E-11
ZBTB47	997.593466	-1.341668203	0.188821	-7.10552	1.20E-12	4.61E-11
FANCB	48.9309831	1.890321774	0.26604	7.105412	1.20E-12	4.61E-11
RNF122	360.785368	-1.537209371	0.216356	-7.10499	1.20E-12	4.62E-11
LINC0070	29.1796853	-3.455968022	0.486451	-7.10445	1.21E-12	4.63E-11
CDK1	933.786645	2.304701837	0.324421	7.104037	1.21E-12	4.63E-11
CD40LG	42.8315508	-2.93499452	0.413191	-7.10324	1.22E-12	4.65E-11
EPHA3	469.482619	-2.281940859	0.321464	-7.09859	1.26E-12	4.81E-11
EBF1	541.943716	-2.471594642	0.348268	-7.09682	1.28E-12	4.86E-11
CRYM	237.079728	-2.970767382	0.418679	-7.09557	1.29E-12	4.89E-11
C1orf112	345.482989	1.498105713	0.211238	7.092025	1.32E-12	5.01E-11
BRCA1	475.374684	1.467996551	0.207034	7.090607	1.34E-12	5.05E-11
GADD45E	2110.87992	-1.813301569	0.255972	-7.08399	1.40E-12	5.28E-11
GRK5	698.130906	-1.79071268	0.252964	-7.07892	1.45E-12	5.47E-11
E2F3	1164.77114	1.27431442	0.180094	7.075842	1.49E-12	5.57E-11
PFKFB4	400.190485	1.582541805	0.223656	7.075798	1.49E-12	5.57E-11
EVA1C	536.737785	-1.829530147	0.258639	-7.07369	1.51E-12	5.65E-11
RGS9	53.9466047	-2.441695118	0.345217	-7.07292	1.52E-12	5.67E-11
EZH2	729.733382	1.680162691	0.237596	7.07152	1.53E-12	5.72E-11
RPS17P1	1.8364134	-3.681407908	0.520925	-7.06706	1.58E-12	5.90E-11
PIGR	9438.33056	-3.918972441	0.554655	-7.06561	1.60E-12	5.95E-11
RAB37	190.116545	-2.289305841	0.32402	-7.06533	1.60E-12	5.95E-11
DEPDC1	298.897912	2.515284485	0.356019	7.065026	1.61E-12	5.96E-11
ACTL6A	1688.62082	1.494575395	0.211592	7.063491	1.62E-12	6.01E-11
NFASC	1052.75341	-2.551901507	0.361336	-7.0624	1.64E-12	6.04E-11

FANCI	892.926384	1.560688561	0.220988	7.06231	1.64E-12	6.04E-11
NCAPG2	674.944396	1.510515007	0.213886	7.062256	1.64E-12	6.04E-11
CLEC10A	319.635957	-2.710695746	0.383895	-7.06103	1.65E-12	6.08E-11
GNAL	441.809937	-2.562174782	0.362892	-7.06043	1.66E-12	6.10E-11
PAICS	3027.53535	1.157546865	0.163957	7.060057	1.66E-12	6.11E-11
FCER2	56.4495629	-3.714770743	0.52629	-7.05841	1.68E-12	6.17E-11
ODF3L1	21.8070247	-1.99092848	0.282194	-7.05519	1.72E-12	6.30E-11
CMA1	91.1117336	-3.719029349	0.527251	-7.05363	1.74E-12	6.36E-11
CHTF18	704.269088	1.133842922	0.160955	7.044463	1.86E-12	6.78E-11
SMAP2	1694.0172	-1.166337684	0.165611	-7.04265	1.89E-12	6.86E-11
LINC0176	3.72337617	-2.345658104	0.333121	-7.04145	1.90E-12	6.89E-11
HJURP	308.194015	2.106751482	0.299198	7.041333	1.90E-12	6.89E-11
CLEC3A	71.7884184	-4.488297975	0.637546	-7.03996	1.92E-12	6.95E-11
HMGB1P3	9.39563148	-3.582603035	0.509032	-7.03807	1.95E-12	7.02E-11
LILRB5	370.426231	-2.39239858	0.339988	-7.03672	1.97E-12	7.07E-11
MN1	202.14209	-2.75280291	0.391209	-7.03666	1.97E-12	7.07E-11
RACGAP1	740.906967	1.54431027	0.219519	7.034966	1.99E-12	7.12E-11
CD300LG	15.6519651	-3.478302462	0.494431	-7.03496	1.99E-12	7.12E-11
STIL	287.462232	1.730387979	0.245971	7.034927	1.99E-12	7.12E-11
SERPINF1	3402.81666	-2.179939551	0.309889	-7.03459	2.00E-12	7.13E-11
ACKR1	1125.94307	-3.282769749	0.46695	-7.03023	2.06E-12	7.35E-11
GPD2	1634.29682	1.096536549	0.156019	7.028238	2.09E-12	7.44E-11
SOX5	96.7046799	-2.058259095	0.292865	-7.02802	2.09E-12	7.44E-11
DSC2	1472.5773	2.83604644	0.403731	7.024591	2.15E-12	7.60E-11
RBM38-A	11.2813926	-2.598705864	0.369947	-7.02454	2.15E-12	7.60E-11
HSD11B1	175.128385	-2.07986452	0.296212	-7.02154	2.19E-12	7.75E-11
SPAG5	152.108675	1.748756003	0.249345	7.013394	2.33E-12	8.19E-11
FLJ40194	2.28588238	-2.883138338	0.411185	-7.01178	2.35E-12	8.27E-11
NUP155	1135.10258	1.043973721	0.148914	7.010591	2.37E-12	8.33E-11
PDE5A	719.492043	-1.929881428	0.275429	-7.00683	2.44E-12	8.55E-11
CDC6	446.628378	2.033472929	0.290594	6.997638	2.60E-12	9.09E-11
CHEK1	464.5396	1.902952279	0.271957	6.997241	2.61E-12	9.09E-11
PRC1	817.33882	1.860706663	0.265922	6.997179	2.61E-12	9.09E-11
PUS7	588.759591	1.186930355	0.169631	6.99715	2.61E-12	9.09E-11
MIR770	14.520549	-3.370901003	0.481825	-6.99612	2.63E-12	9.15E-11
EMILIN1	3295.16138	-2.494617504	0.356634	-6.9949	2.65E-12	9.21E-11
SEMA3G	355.830887	-1.859536216	0.266085	-6.9885	2.78E-12	9.63E-11
NACC2	994.096084	-1.779497488	0.2547	-6.98665	2.82E-12	9.75E-11
SHE	343.134567	-2.043938186	0.292661	-6.98397	2.87E-12	9.92E-11
MT1JP	4.5696552	-4.113336247	0.589086	-6.98257	2.90E-12	1.00E-10
ABL1	2507.36819	-1.383270656	0.198138	-6.98135	2.92E-12	1.01E-10
LINC0246	2.15269913	-3.185685195	0.45633	-6.9811	2.93E-12	1.01E-10
ZBTB4	3030.48206	-1.038525396	0.148769	-6.98081	2.93E-12	1.01E-10
NCS1	1187.241	-1.906035313	0.273093	-6.97945	2.96E-12	1.02E-10
ZNF93	327.659212	1.188374242	0.170344	6.976316	3.03E-12	1.04E-10
COL7A1	7197.60261	2.081811867	0.298456	6.975268	3.05E-12	1.04E-10
RHOJ	511.950377	-1.977340155	0.283486	-6.9751	3.06E-12	1.04E-10
SCD	3700.90579	2.369095054	0.3397	6.974085	3.08E-12	1.05E-10
CAVIN1	7464.80262	-2.33304763	0.335114	-6.96196	3.36E-12	1.14E-10
ILK	3023.31052	-1.279355611	0.183769	-6.96175	3.36E-12	1.14E-10
PRKG1	396.570948	-2.407154119	0.345799	-6.96114	3.38E-12	1.15E-10
LINC0220	54.0807229	-2.233153494	0.320884	-6.95938	3.42E-12	1.16E-10

SAPCD2	424.192547	2.250120016	0.323382	6.958079	3.45E-12	1.17E-10
MAP3K3	1271.77059	-1.052325571	0.151257	-6.95719	3.47E-12	1.17E-10
SYNE1	1865.13909	-1.956458805	0.281238	-6.95659	3.49E-12	1.18E-10
DNAH3	50.8882298	1.886356946	0.271181	6.956074	3.50E-12	1.18E-10
KLF17	18.9744218	-5.036187587	0.724039	-6.95568	3.51E-12	1.18E-10
SELENOM	1565.1978	-1.929504142	0.277408	-6.95547	3.51E-12	1.18E-10
CKS1B	853.766937	1.553102174	0.22343	6.951195	3.62E-12	1.22E-10
ZNF724	62.8193032	1.644648752	0.236656	6.949547	3.66E-12	1.23E-10
GJB2	2432.10823	3.043898276	0.438129	6.947493	3.72E-12	1.24E-10
RCAN1	1961.60144	-1.726308819	0.248638	-6.94307	3.84E-12	1.28E-10
C1R	6019.39467	-2.056413591	0.296332	-6.93955	3.93E-12	1.31E-10
PAFAH1B	792.800825	1.352295822	0.194938	6.937056	4.00E-12	1.33E-10
CDCA3	333.496067	2.060833925	0.297356	6.930523	4.19E-12	1.39E-10
ZNF572	96.7137206	1.471074317	0.212402	6.925886	4.33E-12	1.44E-10
RAI2	304.032111	-2.215444459	0.320046	-6.92227	4.44E-12	1.47E-10
ANGPTL1	190.376796	-3.523068404	0.509141	-6.91963	4.53E-12	1.50E-10
GLB1L	187.605092	-1.01309684	0.146422	-6.91903	4.55E-12	1.50E-10
SYNDIG1	4.38633474	-2.201357498	0.318211	-6.91792	4.58E-12	1.51E-10
GNG11	1014.38088	-1.728461039	0.249859	-6.91774	4.59E-12	1.51E-10
C7	2794.2633	-3.709788249	0.536275	-6.9177	4.59E-12	1.51E-10
CABP1	64.828965	-1.992051669	0.288158	-6.91305	4.74E-12	1.56E-10
EIF4EBP1	802.825981	1.575403858	0.22802	6.909072	4.88E-12	1.60E-10
LDB2	593.090277	-1.721958298	0.24926	-6.90827	4.91E-12	1.60E-10
RCVRN	5.88172991	-2.611552274	0.378252	-6.90427	5.05E-12	1.65E-10
ARL6IP1	4658.47404	1.001444637	0.145058	6.903738	5.07E-12	1.65E-10
LMX1A	4.14508924	-4.315197737	0.625087	-6.90336	5.08E-12	1.65E-10
CDC45	264.682314	2.382811964	0.345337	6.899966	5.20E-12	1.69E-10
CKAP2	890.899748	1.313493381	0.190366	6.899841	5.21E-12	1.69E-10
MYT1	140.127435	4.212023273	0.61098	6.893875	5.43E-12	1.76E-10
CENPH	275.763963	1.373783681	0.199286	6.893529	5.44E-12	1.76E-10
MIR25	22.6946975	1.65173422	0.239629	6.892894	5.47E-12	1.76E-10
GINS2	278.603228	1.904340976	0.276279	6.892821	5.47E-12	1.76E-10
TMEM22(	180.840032	-1.850045733	0.268534	-6.88943	5.60E-12	1.80E-10
LATS2	903.769488	-1.648760748	0.239427	-6.88629	5.73E-12	1.84E-10
RGL1	839.485496	-1.455798901	0.211415	-6.88598	5.74E-12	1.84E-10
NT5DC2	1544.0887	1.084939559	0.157598	6.884237	5.81E-12	1.86E-10
RNF139-A	119.415856	1.036655124	0.15067	6.880288	5.97E-12	1.91E-10
CKS1BP1	17.7824302	-2.21286889	0.321678	-6.87915	6.02E-12	1.92E-10
NFATC2	732.813442	-1.882727504	0.273817	-6.87587	6.16E-12	1.96E-10
PBK	265.629832	2.443365441	0.35537	6.875558	6.17E-12	1.96E-10
KPNA2	2479.9906	1.606161115	0.233707	6.872551	6.31E-12	2.00E-10
ZNF695	24.7255009	3.174320468	0.462167	6.868342	6.50E-12	2.06E-10
PRKAG2-.	33.8890476	-1.878135533	0.273468	-6.86784	6.52E-12	2.06E-10
MCM7	2907.83627	1.046564759	0.15239	6.867657	6.53E-12	2.06E-10
DENND2/	471.466428	-2.056624588	0.299591	-6.86478	6.66E-12	2.10E-10
REV3L	1112.19517	-1.130202631	0.164683	-6.86292	6.75E-12	2.12E-10
KRT6A	8928.80515	5.252195379	0.765944	6.857151	7.02E-12	2.21E-10
AKAP6	221.211479	-2.599585284	0.379252	-6.8545	7.16E-12	2.24E-10
CCDC141	29.0889948	-2.424639647	0.353739	-6.85433	7.16E-12	2.24E-10
CNTN4	147.375011	-2.029457486	0.296102	-6.85391	7.19E-12	2.25E-10
MAP1A	843.754859	-2.586272708	0.377381	-6.85322	7.22E-12	2.26E-10
CD22	278.352958	-3.295512696	0.481315	-6.8469	7.55E-12	2.36E-10

XRCC3	365.431443	1.032963267	0.150894	6.84563	7.61E-12	2.37E-10
MIR125B1	1.87199007	-3.873444236	0.56598	-6.84378	7.71E-12	2.40E-10
FAXDC2	437.272862	-2.017710621	0.294891	-6.84222	7.80E-12	2.42E-10
ACSM5	24.1846871	-2.044021114	0.29892	-6.83801	8.03E-12	2.49E-10
NR3C2	226.734956	-2.085589004	0.30521	-6.8333	8.30E-12	2.57E-10
PGK1	10367.8747	1.188194686	0.173887	6.833136	8.31E-12	2.57E-10
RAMP1	458.659746	-2.692325186	0.394067	-6.83215	8.37E-12	2.58E-10
ASB5	261.510666	-4.992036765	0.73116	-6.82756	8.64E-12	2.66E-10
RAD51AP	298.938639	1.947806844	0.28536	6.825796	8.74E-12	2.69E-10
MYH2	10.9527603	-6.404900521	0.938767	-6.82267	8.94E-12	2.75E-10
CD164L2	13.0170523	2.449827324	0.359116	6.821835	8.99E-12	2.76E-10
HBA2	844.906163	-2.587662543	0.379342	-6.82144	9.01E-12	2.77E-10
CKS2	755.343496	1.857519531	0.272366	6.819928	9.11E-12	2.79E-10
CD248	1244.15814	-2.468161011	0.362006	-6.818	9.23E-12	2.83E-10
VSIG1	272.600164	-2.371993757	0.347911	-6.81781	9.24E-12	2.83E-10
RNA5SP2(	1.84835996	-3.863830138	0.567019	-6.81429	9.47E-12	2.89E-10
OTC	2.51756064	-4.246605498	0.623197	-6.81423	9.48E-12	2.89E-10
STARD13	674.075468	-1.703357447	0.24999	-6.81371	9.51E-12	2.90E-10
TEKT3	10.3765425	-1.677424907	0.246196	-6.81338	9.53E-12	2.90E-10
CREB5	479.553019	-2.453192639	0.360105	-6.81243	9.60E-12	2.92E-10
TBX5	81.8969989	-2.499431082	0.366962	-6.81114	9.68E-12	2.94E-10
BORA	295.170882	1.416859144	0.20808	6.809196	9.81E-12	2.98E-10
MMRN1	480.908138	-2.881348815	0.423257	-6.80756	9.93E-12	3.01E-10
CDH3	1937.77871	2.321121153	0.341107	6.804669	1.01E-11	3.06E-10
LINC0088	23.6864244	2.829270066	0.415818	6.804105	1.02E-11	3.07E-10
KIF11	557.358406	1.829565823	0.268919	6.803411	1.02E-11	3.08E-10
SYT15	81.9109921	-2.46917133	0.36305	-6.80118	1.04E-11	3.13E-10
SSC5D	595.778251	-2.856168618	0.419988	-6.8006	1.04E-11	3.14E-10
TADA1	473.559883	1.14337707	0.168138	6.80024	1.04E-11	3.14E-10
MAPK15	136.688112	2.555140928	0.375796	6.799278	1.05E-11	3.15E-10
SIGLEC17	38.2250454	-2.334098169	0.34329	-6.79921	1.05E-11	3.15E-10
HSPH1	3860.90922	1.040533191	0.153072	6.797679	1.06E-11	3.18E-10
IGSF9B	98.1705175	-3.011739677	0.443315	-6.79368	1.09E-11	3.27E-10
SLC25A2:	1118.98945	-1.438644204	0.211853	-6.79078	1.12E-11	3.33E-10
H2BU1	13.4114235	2.484335336	0.366048	6.786917	1.15E-11	3.41E-10
NPR2	353.993183	-1.189394884	0.175278	-6.78575	1.15E-11	3.44E-10
GRK5-IT1	5.60875668	-2.094933248	0.308816	-6.78376	1.17E-11	3.48E-10
ASB16	11.0600573	-3.066254845	0.452043	-6.78311	1.18E-11	3.49E-10
BEND3	150.18605	1.039220157	0.153226	6.782284	1.18E-11	3.50E-10
CDCA8	305.61799	2.02617227	0.298797	6.781107	1.19E-11	3.53E-10
CCN6	106.314289	4.394532815	0.648148	6.780134	1.20E-11	3.55E-10
SIGLEC11	16.4413297	-2.285877315	0.337203	-6.77894	1.21E-11	3.57E-10
STK32B	74.656273	-2.327586039	0.343435	-6.77737	1.22E-11	3.61E-10
SYPL2	50.8878149	-2.915793403	0.430303	-6.77615	1.23E-11	3.63E-10
CACNA1F	1380.89692	-2.52886882	0.373314	-6.77411	1.25E-11	3.68E-10
EPB41L2	1937.88171	-1.483083119	0.218956	-6.77343	1.26E-11	3.69E-10
CR2	124.551775	-4.176324094	0.617025	-6.76848	1.30E-11	3.82E-10
MIR22HG	1475.3853	-1.468142285	0.216923	-6.76802	1.31E-11	3.82E-10
MYL3	10.7696589	-2.682046654	0.396317	-6.76743	1.31E-11	3.84E-10
RAB3IL1	274.804084	-2.011971123	0.29731	-6.76725	1.31E-11	3.84E-10
CEP55	474.254969	2.176346205	0.321639	6.766434	1.32E-11	3.85E-10
DNAH11	60.8363332	3.252504082	0.480786	6.764968	1.33E-11	3.89E-10

CACYBP	2060.40171	1.083246587	0.160171	6.763061	1.35E-11	3.93E-10
NR4A1AS	8.04044364	-3.194948144	0.472471	-6.76221	1.36E-11	3.95E-10
ITPR1	1229.58137	-1.503088535	0.22236	-6.75969	1.38E-11	4.02E-10
RBL1	419.734369	1.24204335	0.18384	6.756112	1.42E-11	4.11E-10
TGM1	123.288018	2.4991796	0.369919	6.756018	1.42E-11	4.11E-10
TLN1	9036.88233	-1.518926835	0.224895	-6.75393	1.44E-11	4.17E-10
LNCAROI	21.5768307	5.457527912	0.808359	6.751366	1.46E-11	4.23E-10
ERCC6L	99.9266283	2.395345773	0.354838	6.750531	1.47E-11	4.25E-10
PDCD2L	106.910257	1.137971675	0.168618	6.748809	1.49E-11	4.30E-10
FZD6	1987.8451	1.073459844	0.159084	6.747752	1.50E-11	4.33E-10
LRRK2	335.554565	-1.946783358	0.288539	-6.74703	1.51E-11	4.34E-10
ABCB1	259.245514	-2.032897759	0.30137	-6.74553	1.52E-11	4.38E-10
ASPA	289.403133	-2.899929364	0.430163	-6.74146	1.57E-11	4.50E-10
GPC2	189.833749	2.073427582	0.307569	6.741334	1.57E-11	4.50E-10
KIF24	106.364372	1.802318218	0.267442	6.739104	1.59E-11	4.56E-10
XPNPEP2	134.065792	-3.710086005	0.550777	-6.7361	1.63E-11	4.65E-10
DPYSL2	1739.32487	-1.913176992	0.284088	-6.73444	1.65E-11	4.70E-10
JDP2	651.747197	-1.41553658	0.210324	-6.73025	1.69E-11	4.83E-10
ARHGAP1	550.943002	1.605257063	0.238746	6.723693	1.77E-11	5.05E-10
CRHBP	23.2214183	-2.336400192	0.347846	-6.71677	1.86E-11	5.28E-10
NIBAN1	3595.81824	-2.541826612	0.37854	-6.71481	1.88E-11	5.35E-10
REEP1	226.871332	-3.398740236	0.506216	-6.71401	1.89E-11	5.37E-10
SCN1B	186.266622	-1.893443979	0.282148	-6.71082	1.94E-11	5.48E-10
CTSG	328.224493	-3.35285673	0.499781	-6.70865	1.96E-11	5.55E-10
PTGER2	138.275492	-1.979149936	0.295037	-6.70814	1.97E-11	5.56E-10
MTFR2	106.055842	1.721276609	0.256623	6.707402	1.98E-11	5.58E-10
BUB1B	304.681721	2.019711342	0.301233	6.704806	2.02E-11	5.68E-10
KIF18A	171.111543	2.116060037	0.315622	6.704408	2.02E-11	5.69E-10
THOC3	827.641569	1.071734273	0.159916	6.701837	2.06E-11	5.77E-10
PARPBP	223.862422	1.572315963	0.234673	6.700033	2.08E-11	5.84E-10
RBP7	117.372939	-1.906372026	0.284575	-6.69903	2.10E-11	5.87E-10
MOXD1	452.573341	-2.315677952	0.345743	-6.69769	2.12E-11	5.92E-10
H2BC20P	238.841148	1.242387664	0.185542	6.696006	2.14E-11	5.98E-10
ADCY9	625.528633	-1.674448749	0.25011	-6.69484	2.16E-11	6.01E-10
KLHDC7E	1140.70034	3.564423623	0.532829	6.68962	2.24E-11	6.21E-10
TPX2	1016.91307	2.177780283	0.325676	6.686953	2.28E-11	6.32E-10
MEF2D	2181.34372	-1.04172628	0.155942	-6.68022	2.39E-11	6.59E-10
STARD8	295.992507	-1.950359747	0.291988	-6.6796	2.40E-11	6.61E-10
LINC0087	27.8305981	-2.387425535	0.357537	-6.67742	2.43E-11	6.71E-10
CENPA	165.108134	2.178586843	0.326338	6.67585	2.46E-11	6.77E-10
PER2	998.812357	-1.286476462	0.192811	-6.67223	2.52E-11	6.92E-10
DLC1	1189.13631	-1.641123057	0.246044	-6.67005	2.56E-11	7.01E-10
ITPKB	1309.80559	-1.459718667	0.219096	-6.66246	2.69E-11	7.37E-10
PDZRN4	166.340511	-4.032998127	0.605374	-6.662	2.70E-11	7.38E-10
TPSD1	69.5191702	-3.16745068	0.475798	-6.65713	2.79E-11	7.62E-10
SH3D19	1872.7346	-1.170869208	0.175899	-6.65647	2.80E-11	7.65E-10
HCN3	259.929134	1.107414423	0.166405	6.654917	2.83E-11	7.72E-10
COL25A1	14.1770677	-2.546506415	0.382807	-6.6522	2.89E-11	7.86E-10
KCNA5	24.7185028	-2.772786319	0.416832	-6.65205	2.89E-11	7.86E-10
CEROX1	56.8122905	-1.942873384	0.292184	-6.64948	2.94E-11	7.98E-10
ZSCAN2	260.495331	1.13264538	0.170352	6.648843	2.95E-11	8.01E-10
OMG	13.5242081	-3.043455119	0.457747	-6.64877	2.96E-11	8.01E-10

LINC0135	542.680081	1.193619463	0.179573	6.646982	2.99E-11	8.09E-10
ATAD5	296.182358	1.499136534	0.225639	6.643946	3.05E-11	8.25E-10
MROH8	50.7740561	-1.149350332	0.173007	-6.64338	3.07E-11	8.28E-10
LINC0214	1.25218705	-3.171759794	0.477804	-6.63821	3.18E-11	8.56E-10
RNU6-117	7.01864324	-2.656733576	0.400229	-6.63803	3.18E-11	8.56E-10
F8	273.503194	-1.315718173	0.19833	-6.63399	3.27E-11	8.79E-10
KIFC1	432.672511	1.911503344	0.288408	6.627783	3.41E-11	9.16E-10
FABP7P1	13.0198656	-2.702137875	0.407791	-6.62628	3.44E-11	9.24E-10
RFC4	598.318138	1.556194487	0.234865	6.625902	3.45E-11	9.26E-10
CFP	185.828119	-2.194510898	0.331413	-6.62169	3.55E-11	9.50E-10
HOOK1	656.54376	1.516853357	0.22908	6.621491	3.56E-11	9.50E-10
TFR2	90.6336628	1.58099885	0.238807	6.620396	3.58E-11	9.57E-10
CDCA5	343.77269	1.999373371	0.302091	6.618439	3.63E-11	9.68E-10
SETBP1-D	28.6609817	-1.959746325	0.296118	-6.61812	3.64E-11	9.69E-10
LMCD1	956.677044	-2.262857178	0.342055	-6.61548	3.70E-11	9.86E-10
RN7SL749	8.2194245	-2.364287761	0.357534	-6.61277	3.77E-11	1.00E-09
LINC0228	8.63734311	-1.6646056	0.251783	-6.61126	3.81E-11	1.01E-09
MS4A2	76.1743136	-2.199959078	0.332861	-6.60925	3.86E-11	1.02E-09
LINC0141	360.687245	1.647453497	0.249341	6.607242	3.92E-11	1.04E-09
FAM72D	81.4813989	2.083179489	0.315492	6.602957	4.03E-11	1.07E-09
CKAP2L	295.827903	2.150962064	0.325981	6.598437	4.16E-11	1.10E-09
SH3BGR	145.359062	-1.435277624	0.217608	-6.59572	4.23E-11	1.12E-09
ESPL1	357.16701	1.770587158	0.268465	6.595228	4.25E-11	1.12E-09
SOX18	390.65782	-1.983732903	0.300807	-6.5947	4.26E-11	1.12E-09
CDC20	656.713926	2.261688975	0.34296	6.594618	4.26E-11	1.12E-09
SPDL1	333.619805	1.051518345	0.159518	6.591858	4.34E-11	1.14E-09
SGO1	217.825647	2.11768154	0.321259	6.591817	4.34E-11	1.14E-09
RORB	23.3703142	-2.686891065	0.407745	-6.58964	4.41E-11	1.16E-09
ZFP36	13878.0771	-1.642612551	0.249343	-6.58777	4.46E-11	1.17E-09
AXL	1627.48254	-1.806716106	0.274663	-6.57793	4.77E-11	1.25E-09
KIF23	597.000127	1.81395738	0.27583	6.576363	4.82E-11	1.26E-09
EMCN	686.613212	-2.053577866	0.312292	-6.57582	4.84E-11	1.26E-09
GTF2IP5	15.557904	1.915595016	0.291332	6.575299	4.86E-11	1.27E-09
LINC0063	12.4897207	-1.481936703	0.225386	-6.57509	4.86E-11	1.27E-09
STON1-G	8.13918929	-3.854211915	0.58624	-6.57446	4.88E-11	1.27E-09
TRAIP	149.70317	1.528169774	0.232531	6.571887	4.97E-11	1.29E-09
SH3RF3-A	13.8875675	-1.993002253	0.303288	-6.57131	4.99E-11	1.29E-09
LTF	182.974014	-3.41464613	0.51995	-6.56726	5.12E-11	1.33E-09
CACNB2	217.228761	-2.935173193	0.446952	-6.56709	5.13E-11	1.33E-09
TEX45	53.528866	2.85919889	0.435594	6.563915	5.24E-11	1.36E-09
MICU3	131.082064	-2.13504978	0.325356	-6.5622	5.30E-11	1.37E-09
TUBA5P	217.517564	2.269359694	0.345856	6.561574	5.32E-11	1.37E-09
FBXO45	721.136957	1.160349911	0.17696	6.557117	5.49E-11	1.42E-09
INSYN1	76.6946503	-2.410985894	0.367754	-6.55597	5.53E-11	1.42E-09
ACVRL1	728.316005	-1.752124866	0.26732	-6.55441	5.59E-11	1.44E-09
ADARB1	1503.70656	-2.026435447	0.30924	-6.55294	5.64E-11	1.45E-09
ZC3H8	427.586889	1.091254516	0.166534	6.552738	5.65E-11	1.45E-09
C19orf48	842.933835	1.033725662	0.157801	6.550837	5.72E-11	1.47E-09
RAD51	181.959036	1.766866929	0.269745	6.550135	5.75E-11	1.47E-09
ZDHHC23	389.26324	1.25463698	0.191593	6.548434	5.81E-11	1.49E-09
NME1	603.248279	1.512266322	0.230958	6.547791	5.84E-11	1.49E-09
CCNB2	518.988248	2.189558354	0.334408	6.547571	5.85E-11	1.49E-09

ADCYAP1	30.3255258	-3.392749539	0.518219	-6.54694	5.87E-11	1.50E-09
LINC0169	9.83529566	-2.342195759	0.357757	-6.5469	5.87E-11	1.50E-09
IQCC	115.380273	1.097332051	0.16767	6.544592	5.97E-11	1.52E-09
KNL1	283.981156	1.753339765	0.268167	6.538232	6.23E-11	1.58E-09
EBP	953.252058	1.185281995	0.181303	6.537581	6.25E-11	1.59E-09
DUXAP8	276.058813	3.150024795	0.481953	6.535953	6.32E-11	1.60E-09
RHOB	8799.84765	-1.453203807	0.222519	-6.53068	6.55E-11	1.65E-09
TMEM52	38.3282477	2.609488083	0.399761	6.527615	6.68E-11	1.69E-09
NSUN2	2800.94452	1.000892724	0.153387	6.525288	6.79E-11	1.71E-09
LINC0092	15.671617	-2.491455165	0.381982	-6.52244	6.92E-11	1.74E-09
CHAF1B	251.737762	1.420179154	0.217789	6.520903	6.99E-11	1.75E-09
ORC1	133.377571	1.740733892	0.266953	6.520738	7.00E-11	1.75E-09
CENPL	204.127633	1.057534489	0.162184	6.520572	7.00E-11	1.75E-09
PLXNA4	124.292301	-3.295872369	0.505489	-6.52017	7.02E-11	1.76E-09
LRP8	288.504339	1.920739928	0.294639	6.518949	7.08E-11	1.77E-09
MASTL	526.699861	1.085156802	0.16652	6.516682	7.19E-11	1.79E-09
ALDH1A3	1986.28981	-1.833222954	0.281334	-6.51619	7.21E-11	1.80E-09
PRX	160.506252	-1.392879917	0.213786	-6.5153	7.25E-11	1.80E-09
GDPD2	186.009917	3.341234335	0.512929	6.514028	7.32E-11	1.82E-09
LINC0095	254.871756	2.902649363	0.445685	6.51278	7.38E-11	1.83E-09
CDT1	316.654317	1.904670555	0.292709	6.507055	7.66E-11	1.90E-09
CDK15	35.9073245	-2.08944318	0.321153	-6.50607	7.71E-11	1.91E-09
ULBP2	137.107274	2.207429651	0.339328	6.505292	7.75E-11	1.92E-09
FCN2	3.06612187	-3.845114411	0.591165	-6.5043	7.81E-11	1.93E-09
SKA1	192.12964	2.19038122	0.33676	6.504287	7.81E-11	1.93E-09
ECE2	153.998124	1.561327342	0.240141	6.501721	7.94E-11	1.96E-09
NTN1	422.74309	-2.809003866	0.432078	-6.50114	7.97E-11	1.96E-09
S1PR1	677.621552	-1.990485071	0.306248	-6.49958	8.05E-11	1.98E-09
PKMP3	15.6643414	-1.7706947	0.272461	-6.49889	8.09E-11	1.99E-09
ESRP1	3022.56517	1.500201865	0.23087	6.498044	8.14E-11	2.00E-09
KCP	283.45247	1.999890302	0.307843	6.496458	8.22E-11	2.01E-09
ANK2	469.901137	-2.95347876	0.454655	-6.49609	8.24E-11	2.02E-09
CENPU	365.362364	1.787829941	0.275258	6.495107	8.30E-11	2.03E-09
PSMC3IP	204.761409	1.244024688	0.191667	6.490539	8.55E-11	2.09E-09
PGLYRP4	40.8178774	4.094746441	0.630902	6.490309	8.57E-11	2.09E-09
GATA6-A	35.3682997	-2.275608723	0.350781	-6.48726	8.74E-11	2.12E-09
RSPO1	27.9435223	-3.258180406	0.50227	-6.48692	8.76E-11	2.13E-09
CILP	913.443289	-3.464293886	0.534109	-6.48612	8.81E-11	2.14E-09
HAS1	82.9972118	-3.761933525	0.580083	-6.48516	8.86E-11	2.15E-09
SDC3	2253.49474	-1.569984334	0.242224	-6.48155	9.08E-11	2.20E-09
STMN1	4306.40636	1.977254965	0.305092	6.48084	9.12E-11	2.20E-09
ST6GALN	763.215601	-1.052517152	0.162534	-6.47569	9.44E-11	2.28E-09
IL22RA1	61.0921844	2.027483322	0.313131	6.474872	9.49E-11	2.29E-09
CAPN6	61.3914278	-3.225679982	0.498334	-6.47293	9.61E-11	2.31E-09
THRA	918.098099	-1.197754115	0.185059	-6.47228	9.65E-11	2.32E-09
MYLK4	171.487215	2.82644513	0.436779	6.471112	9.73E-11	2.34E-09
KNTC1	852.055192	1.376737494	0.212762	6.470786	9.75E-11	2.34E-09
APBB1	465.224553	-1.973815723	0.305129	-6.46878	9.88E-11	2.37E-09
SYNPO	2655.10012	-1.856792388	0.287058	-6.46836	9.91E-11	2.37E-09
SNORD73	5.72837815	2.072673917	0.320557	6.465856	1.01E-10	2.41E-09
PABPC5	23.5993111	-1.998841811	0.309202	-6.46451	1.02E-10	2.43E-09
DLGAP5	404.595464	2.266578345	0.350641	6.464101	1.02E-10	2.43E-09

MXRA7P1	173.529803	-1.46600238	0.226837	-6.4628	1.03E-10	2.45E-09
FAM180B	5.60170136	-3.441142382	0.532479	-6.4625	1.03E-10	2.45E-09
LINC0134	7.96557874	-3.540053057	0.548057	-6.45927	1.05E-10	2.50E-09
DIPK2B	596.212484	-1.769512554	0.274097	-6.45579	1.08E-10	2.56E-09
VPS9D1-A	158.202502	2.093421701	0.324273	6.45574	1.08E-10	2.56E-09
POU6F1	275.351871	-1.655431228	0.256526	-6.45328	1.09E-10	2.59E-09
ZNF738	421.196514	1.299390846	0.201373	6.452657	1.10E-10	2.60E-09
KLHDC1	178.10453	-1.038827981	0.161002	-6.45225	1.10E-10	2.61E-09
NCAPG	396.75882	2.068633591	0.320613	6.452126	1.10E-10	2.61E-09
CTSV	171.309375	3.141321635	0.487232	6.447282	1.14E-10	2.68E-09
MCMDC2	46.0421361	1.511959998	0.234697	6.442167	1.18E-10	2.77E-09
LINC0123	33.1927848	-1.572547556	0.24416	-6.44063	1.19E-10	2.80E-09
FRGCA	15.5265907	2.754596917	0.427696	6.440541	1.19E-10	2.80E-09
RUSC2	758.980675	-1.73309007	0.269142	-6.43933	1.20E-10	2.82E-09
RFX2	573.634176	-1.196239927	0.18578	-6.43902	1.20E-10	2.82E-09
SNORD11	4.07727792	-3.437304371	0.533888	-6.43825	1.21E-10	2.83E-09
GRIK3	65.3706504	-3.017012799	0.468637	-6.43785	1.21E-10	2.84E-09
SYTL3	236.313572	-1.282872137	0.199285	-6.43739	1.22E-10	2.84E-09
WDR62	238.717652	1.702328118	0.264559	6.43459	1.24E-10	2.89E-09
SKA3	185.199245	2.189817875	0.34032	6.434587	1.24E-10	2.89E-09
TRPC3	52.1370613	-2.499556136	0.388763	-6.42951	1.28E-10	2.98E-09
LAMB2	4450.65057	-1.12328355	0.174708	-6.42951	1.28E-10	2.98E-09
GDNF	50.6387295	-1.977595173	0.307626	-6.42858	1.29E-10	2.99E-09
NLRP6	15.2907333	-2.408857802	0.374813	-6.42682	1.30E-10	3.02E-09
TMEM63C	48.847734	3.592589301	0.559011	6.426685	1.30E-10	3.02E-09
PPP1R14E	2760.3196	1.039899509	0.161912	6.422628	1.34E-10	3.10E-09
ARHGAP3	374.339272	1.171743348	0.182457	6.422029	1.34E-10	3.11E-09
BIRC5	727.988812	2.250809062	0.35059	6.42006	1.36E-10	3.15E-09
DDX12P	442.547078	1.07362266	0.167262	6.418808	1.37E-10	3.17E-09
ZNF736	101.741562	1.62412002	0.253049	6.418206	1.38E-10	3.17E-09
SASS6	277.992189	1.209488695	0.188489	6.416747	1.39E-10	3.20E-09
CENPW	316.83338	1.841692167	0.287039	6.41617	1.40E-10	3.20E-09
STARD9	622.580037	-2.109399792	0.328766	-6.41612	1.40E-10	3.20E-09
TMEM74E	172.03988	1.906102016	0.297417	6.408856	1.47E-10	3.35E-09
LEMD1	18.7636602	3.83971961	0.599163	6.408469	1.47E-10	3.36E-09
SCIRT	19.5412205	2.283742722	0.356568	6.404794	1.51E-10	3.43E-09
KCNMA1	699.951184	-2.598518075	0.40584	-6.40282	1.53E-10	3.47E-09
CCM2L	128.659173	-1.710257612	0.26713	-6.40233	1.53E-10	3.48E-09
C1QL1	81.8790304	-2.605968302	0.407129	-6.40084	1.55E-10	3.51E-09
AADACL4	1.39356681	-3.091109064	0.482993	-6.3999	1.55E-10	3.52E-09
INA	597.6409	2.90161291	0.453466	6.398749	1.57E-10	3.55E-09
CHRD	184.550708	-2.1563897	0.337011	-6.39857	1.57E-10	3.55E-09
PKP1	1947.67272	2.839342633	0.44376	6.398371	1.57E-10	3.55E-09
CPA3	970.441576	-2.458884294	0.384408	-6.39655	1.59E-10	3.59E-09
MEIS2	1096.14785	-1.437969052	0.224826	-6.39591	1.60E-10	3.60E-09
HIF1A-AS	7.16040624	2.662226154	0.416258	6.39561	1.60E-10	3.60E-09
SLC8A1	590.409953	-1.934128044	0.302478	-6.39427	1.61E-10	3.62E-09
SLC17A8	9.46101198	-3.871786381	0.60572	-6.39204	1.64E-10	3.67E-09
VSIR	1790.08648	-1.39129275	0.21766	-6.39204	1.64E-10	3.67E-09
ESCO2	145.97999	2.074566963	0.324603	6.391088	1.65E-10	3.68E-09
ENAM	26.6793932	-4.121918084	0.645054	-6.39004	1.66E-10	3.71E-09
CREB3L4	467.583259	1.192595532	0.18665	6.389464	1.66E-10	3.72E-09

TMEM132	1140.54692	1.721433245	0.269628	6.384474	1.72E-10	3.83E-09
ZEB1	1224.44804	-1.858631817	0.29112	-6.38441	1.72E-10	3.83E-09
DUXAP10	346.659879	3.041496896	0.476603	6.381615	1.75E-10	3.90E-09
CLEC9A	29.2753576	-2.385204952	0.37381	-6.38079	1.76E-10	3.91E-09
COL21A1	254.071472	-2.008755257	0.314813	-6.38079	1.76E-10	3.91E-09
ZNF681	232.071108	1.68931281	0.264757	6.380608	1.76E-10	3.91E-09
ZNF74	383.708504	1.05879151	0.165945	6.380364	1.77E-10	3.92E-09
FOXM1	722.93838	2.047794247	0.321029	6.37884	1.78E-10	3.95E-09
PLEKHO2	926.370985	-1.442798997	0.226468	-6.37089	1.88E-10	4.16E-09
HDC	289.061267	-2.6493229	0.416108	-6.36691	1.93E-10	4.26E-09
TMEM220	24.667628	-1.782758693	0.280026	-6.3664	1.94E-10	4.27E-09
DIO3	18.1746175	-2.706626481	0.425194	-6.36563	1.94E-10	4.29E-09
LY6K	268.67803	2.337047836	0.367207	6.364396	1.96E-10	4.32E-09
NPM1P9	4.60444902	2.309128219	0.36296	6.361945	1.99E-10	4.39E-09
MTTP	19.9984299	-3.426306232	0.538573	-6.36183	1.99E-10	4.39E-09
RPL23AP	21.4033476	-2.023379215	0.318093	-6.36097	2.00E-10	4.41E-09
PAPOLA-	22.4058491	1.471858538	0.231427	6.359919	2.02E-10	4.43E-09
EGFL7	900.179465	-1.574727475	0.247603	-6.35988	2.02E-10	4.43E-09
PANCR	2.27915013	-3.925788289	0.617453	-6.35804	2.04E-10	4.48E-09
SLC24A3	363.151761	-2.083384175	0.327694	-6.35772	2.05E-10	4.49E-09
POC1A	200.580381	1.605382468	0.252529	6.357209	2.05E-10	4.50E-09
AHRR	183.096546	-1.637413368	0.257613	-6.3561	2.07E-10	4.53E-09
KRT18P5	19.9171602	2.774015428	0.436482	6.355388	2.08E-10	4.54E-09
RND1	260.200571	-2.111079729	0.332206	-6.35472	2.09E-10	4.56E-09
ZBED9	108.426821	2.549489076	0.401229	6.3542	2.10E-10	4.57E-09
EMP3	1030.20893	-1.813902049	0.285471	-6.35406	2.10E-10	4.57E-09
ADAM10	6002.30313	1.082179269	0.170489	6.347488	2.19E-10	4.76E-09
ANKEF1	197.122037	1.140883011	0.179752	6.34697	2.20E-10	4.77E-09
C2orf88	178.518076	-1.589106395	0.250415	-6.34589	2.21E-10	4.80E-09
NIBAN3	141.936759	-3.054796457	0.481459	-6.34487	2.23E-10	4.82E-09
DPT	1032.63849	-3.8594071	0.608849	-6.33886	2.31E-10	5.01E-09
CELF2	1105.18631	-2.119885423	0.33447	-6.33804	2.33E-10	5.03E-09
FOXP2	290.148633	-2.478213397	0.391078	-6.33687	2.34E-10	5.06E-09
MMP2	12843.5209	-1.959638349	0.309365	-6.3344	2.38E-10	5.14E-09
S100A14	3497.03753	2.935657498	0.463464	6.33416	2.39E-10	5.14E-09
DUXAP9	425.62643	2.985620496	0.471401	6.3335	2.40E-10	5.15E-09
CCNE2	269.748326	2.190494619	0.345862	6.333441	2.40E-10	5.15E-09
RCSD1	541.349996	-2.001798201	0.316071	-6.33339	2.40E-10	5.15E-09
NOTO	1.58570143	-3.856219458	0.608881	-6.33328	2.40E-10	5.15E-09
PRR11	435.18816	1.763354618	0.278596	6.329424	2.46E-10	5.27E-09
F13A1	2529.49028	-2.242986768	0.354467	-6.32778	2.49E-10	5.32E-09
ARHGAP	2865.64382	-1.285031415	0.203109	-6.32679	2.50E-10	5.35E-09
RNU6-529	6.4999708	-1.694175288	0.267818	-6.32584	2.52E-10	5.38E-09
MTERF3	559.690389	1.00193326	0.158442	6.32367	2.55E-10	5.44E-09
HOXC4	122.254999	2.14144842	0.338989	6.317168	2.66E-10	5.66E-09
FANCA	589.597778	1.273426926	0.201672	6.314362	2.71E-10	5.76E-09
CXCL12	1914.38893	-2.687636944	0.425694	-6.31354	2.73E-10	5.78E-09
PAQR4	321.366499	1.419574106	0.224847	6.313509	2.73E-10	5.78E-09
LINC0259	20.523625	2.658889806	0.421182	6.31292	2.74E-10	5.79E-09
SMC4	3311.31894	1.095707441	0.173573	6.31267	2.74E-10	5.79E-09
DDR2	166.00953	-2.141919143	0.339483	-6.30936	2.80E-10	5.91E-09
EFNA4	248.272364	1.270787475	0.201419	6.30916	2.81E-10	5.92E-09

HDAC4	547.369885	-1.342712724	0.21286	-6.30795	2.83E-10	5.96E-09
IL2	2.50292568	-2.961525331	0.469613	-6.30631	2.86E-10	6.02E-09
OPN4	3.30580877	-3.495574645	0.554327	-6.30598	2.86E-10	6.02E-09
HLX-AS1	6.43723509	-2.679299147	0.424928	-6.3053	2.88E-10	6.05E-09
DEPDC1B	181.56166	2.111668119	0.335126	6.301108	2.96E-10	6.21E-09
CCR6	137.895634	-2.448978628	0.389054	-6.2947	3.08E-10	6.45E-09
THBS1	15818.6429	-2.123040545	0.337285	-6.29451	3.08E-10	6.45E-09
CD79B	200.594175	-2.665526894	0.423867	-6.28859	3.20E-10	6.69E-09
FANCL	832.420387	1.036694782	0.164962	6.284447	3.29E-10	6.87E-09
SETBP1	586.391712	-1.87629224	0.298627	-6.28307	3.32E-10	6.92E-09
SIX4	312.016914	1.345881614	0.214444	6.276136	3.47E-10	7.22E-09
KIF18B	324.658917	2.040648366	0.325238	6.274319	3.51E-10	7.30E-09
PLXNA3	1765.77853	1.041504839	0.166014	6.273606	3.53E-10	7.32E-09
SERTM1	4.78200913	-4.079715212	0.650355	-6.27306	3.54E-10	7.34E-09
MS4A4E	48.4129086	-1.546074278	0.246603	-6.26949	3.62E-10	7.50E-09
TACR1	37.011991	-2.782543935	0.443965	-6.26748	3.67E-10	7.59E-09
FAM138B	2.30133377	-3.451828592	0.55095	-6.26523	3.72E-10	7.69E-09
LYPD2	48.113359	5.909700087	0.943797	6.261621	3.81E-10	7.86E-09
STAM-AS	22.1187237	1.484472318	0.237133	6.260078	3.85E-10	7.93E-09
PCNA	2627.82559	1.432442123	0.228864	6.25892	3.88E-10	7.97E-09
PRKAG2	936.95668	-1.062675044	0.169786	-6.2589	3.88E-10	7.97E-09
TENT5B	209.617849	-2.646710722	0.42333	-6.25213	4.05E-10	8.31E-09
STEAP3	1315.54925	1.268954349	0.202969	6.251968	4.05E-10	8.31E-09
NFATC1	428.425819	-1.841418966	0.294634	-6.24986	4.11E-10	8.41E-09
IL16	746.306705	-2.137024578	0.341964	-6.24926	4.12E-10	8.44E-09
DAAM2	478.275246	-2.173050834	0.347738	-6.2491	4.13E-10	8.44E-09
CKMT2	31.1486875	-2.084203188	0.333642	-6.24683	4.19E-10	8.55E-09
GEM	1013.90787	-2.297632204	0.368012	-6.24337	4.28E-10	8.73E-09
SYNE4	245.632229	2.032566533	0.32558	6.242905	4.30E-10	8.75E-09
POLQ	245.990426	2.007712235	0.321672	6.241494	4.33E-10	8.82E-09
PIMREG	123.126324	2.226878571	0.35679	6.241427	4.34E-10	8.82E-09
DUS4L	341.870021	1.037291461	0.166208	6.240942	4.35E-10	8.84E-09
TRIM59	236.142964	1.066905524	0.170956	6.24083	4.35E-10	8.84E-09
PTX3	252.128096	-2.665324008	0.427095	-6.24058	4.36E-10	8.84E-09
CCNF	378.779769	1.096776702	0.175782	6.239426	4.39E-10	8.90E-09
FZD10-AS	99.8788659	-2.004405105	0.321262	-6.23915	4.40E-10	8.90E-09
IL20RB	211.34022	2.487999873	0.398891	6.237296	4.45E-10	9.00E-09
LAMC2	3322.65739	2.326891315	0.373077	6.237027	4.46E-10	9.01E-09
LINC0163	3.89703558	-3.139176407	0.504345	-6.22427	4.84E-10	9.75E-09
TNRC6C	399.328315	-1.223115496	0.196736	-6.21703	5.07E-10	1.02E-08
TRAV41	3.06558568	-3.062305039	0.492609	-6.21651	5.08E-10	1.02E-08
SLIT2	625.20425	-2.759999613	0.443994	-6.2163	5.09E-10	1.02E-08
ANXA6	3017.86622	-2.095093135	0.337055	-6.21589	5.10E-10	1.02E-08
ZNF260	755.962143	1.008595618	0.162265	6.21574	5.11E-10	1.02E-08
CTSE	1317.77605	3.471036598	0.558484	6.21511	5.13E-10	1.03E-08
ZC3HAV1	118.783868	1.619311142	0.260581	6.214232	5.16E-10	1.03E-08
GPR55	32.9551373	-2.264017954	0.364528	-6.21082	5.27E-10	1.05E-08
SSPN	596.829945	-1.461456693	0.235349	-6.20973	5.31E-10	1.06E-08
MYADM	3988.35903	-2.110086848	0.339806	-6.20968	5.31E-10	1.06E-08
LASTR	20.1327981	3.443114586	0.554485	6.209569	5.31E-10	1.06E-08
LINC0276	8.1054312	-2.735825737	0.441001	-6.20367	5.52E-10	1.10E-08
TUBAP4	1.91934131	-3.998559643	0.644664	-6.20254	5.56E-10	1.10E-08

RYR2	228.981592	-2.411380123	0.388852	-6.20128	5.60E-10	1.11E-08
VEGFA	13793.1115	1.598803313	0.25795	6.198118	5.71E-10	1.13E-08
FAM72B	78.4509477	1.663560925	0.268433	6.19731	5.74E-10	1.14E-08
MEDAG	435.352034	-2.56579963	0.414315	-6.19287	5.91E-10	1.17E-08
TNMD	2.38158976	-3.158134828	0.509991	-6.19254	5.92E-10	1.17E-08
STXBP6	190.769981	-2.179738883	0.352008	-6.1923	5.93E-10	1.17E-08
IGSF9	912.662283	1.845331584	0.298013	6.192114	5.94E-10	1.17E-08
CHML	548.877457	1.217218454	0.196582	6.191907	5.94E-10	1.17E-08
NR2F2-AS	57.5407077	-1.491002962	0.241144	-6.18305	6.29E-10	1.23E-08
LINC0016	2.72752139	-4.065119792	0.657562	-6.18211	6.32E-10	1.24E-08
CIP2A	303.670779	1.420395006	0.229843	6.179846	6.42E-10	1.25E-08
INCENP	529.362129	1.078171455	0.174529	6.177593	6.51E-10	1.27E-08
CBX2	244.061792	1.9388731	0.314114	6.17251	6.72E-10	1.31E-08
COLEC12	661.466639	-2.344699508	0.380108	-6.16851	6.89E-10	1.34E-08
MEG3	4173.97056	-2.136670675	0.346586	-6.16491	7.05E-10	1.37E-08
CRLF2	17.1104863	-2.840031434	0.460727	-6.16424	7.08E-10	1.37E-08
CLEC4F	31.7340153	-2.136679506	0.346663	-6.16356	7.11E-10	1.38E-08
NTF3	55.0072102	-1.919832648	0.311702	-6.1592	7.31E-10	1.41E-08
NEXN-AS	14.116054	-2.54028385	0.412565	-6.15729	7.40E-10	1.43E-08
PLAAT5	28.442475	-2.600360625	0.422696	-6.15184	7.66E-10	1.47E-08
NDC80	448.000675	1.740681752	0.282978	6.151289	7.69E-10	1.48E-08
PIP5K1C	1647.0606	-1.052184942	0.171085	-6.15007	7.74E-10	1.49E-08
NOP56	3284.40457	1.003230409	0.163336	6.142109	8.14E-10	1.56E-08
MIR27A	11.4555207	-2.840226032	0.462472	-6.1414	8.18E-10	1.56E-08
TFRC	4821.86136	1.166830637	0.190022	6.140512	8.23E-10	1.57E-08
GPER1	63.6369901	-1.815502005	0.295711	-6.13945	8.28E-10	1.58E-08
TANC2	1829.47553	1.330575959	0.216748	6.138828	8.31E-10	1.58E-08
OIP5	82.1130417	1.928803077	0.31432	6.136425	8.44E-10	1.61E-08
GTF2IRD1	1257.13443	1.050849948	0.171353	6.132646	8.64E-10	1.65E-08
C5orf34-A	29.1605058	2.070820108	0.337682	6.132451	8.65E-10	1.65E-08
RANBP1	2414.50487	1.07985563	0.176151	6.130298	8.77E-10	1.67E-08
ZNF737	684.171861	1.946849046	0.317581	6.130251	8.77E-10	1.67E-08
CYP46A1	51.5119719	-1.919166959	0.313308	-6.1255	9.04E-10	1.71E-08
HSPE1	938.524714	1.232849921	0.201398	6.121475	9.27E-10	1.76E-08
FYCO1	1747.3309	-1.101835392	0.180056	-6.1194	9.39E-10	1.78E-08
LINC0092	250.366	-1.862426385	0.304405	-6.11826	9.46E-10	1.79E-08
PARM1	1662.86094	-1.926006758	0.315298	-6.10853	1.01E-09	1.90E-08
JPH2	730.828306	-3.127815624	0.512054	-6.10837	1.01E-09	1.90E-08
MYOM2	70.476839	-1.861308042	0.3048	-6.10666	1.02E-09	1.92E-08
HMGA1	5046.85473	1.226996186	0.200936	6.106393	1.02E-09	1.92E-08
ZNF253	355.762983	1.059197365	0.173557	6.102861	1.04E-09	1.95E-08
ABCB4	59.8636385	-1.65379467	0.271034	-6.10179	1.05E-09	1.97E-08
PKMYT1	238.185227	1.988896734	0.325967	6.101524	1.05E-09	1.97E-08
VNN2	300.879359	-2.260794006	0.370581	-6.10067	1.06E-09	1.98E-08
LINC0225	67.6508374	-1.709590238	0.280251	-6.10022	1.06E-09	1.98E-08
SPC25	132.495964	2.017882986	0.330876	6.098601	1.07E-09	2.00E-08
ABCC9	253.076879	-1.919034392	0.314757	-6.09687	1.08E-09	2.02E-08
KIF5A	136.237608	2.932617031	0.481053	6.096245	1.09E-09	2.03E-08
STK31	68.8665894	1.892561562	0.310572	6.093801	1.10E-09	2.06E-08
OPLAH	544.411584	1.565662881	0.256952	6.093215	1.11E-09	2.06E-08
SLC30A1(	8.69105767	4.284252316	0.70323	6.092249	1.11E-09	2.07E-08
RPP40	231.561204	1.048120519	0.172218	6.086024	1.16E-09	2.15E-08

HAP1	115.634134	2.300671169	0.378426	6.079581	1.20E-09	2.23E-08
PGM5-AS	346.424129	-4.407718154	0.725102	-6.07876	1.21E-09	2.24E-08
MNX1	36.0643877	2.963861903	0.487749	6.076609	1.23E-09	2.27E-08
ZNF391	125.881509	1.256830131	0.206875	6.075312	1.24E-09	2.28E-08
FAM189A	149.491947	-1.696446618	0.279255	-6.07491	1.24E-09	2.29E-08
TSHZ3	472.369738	-1.772365808	0.291808	-6.07374	1.25E-09	2.30E-08
SHISAL1	363.95629	-2.844516833	0.468361	-6.07334	1.25E-09	2.31E-08
KIF22	1419.96329	1.043444442	0.171836	6.072321	1.26E-09	2.32E-08
NRXN2	135.487407	-2.516149025	0.414443	-6.07116	1.27E-09	2.33E-08
HORMAD	87.1048325	3.314807707	0.546001	6.071069	1.27E-09	2.33E-08
COL16A1	3393.22025	-1.972298799	0.3249	-6.07047	1.28E-09	2.34E-08
AKNA	1745.64907	-1.431327112	0.235822	-6.06953	1.28E-09	2.35E-08
UTRN	1984.96339	-1.251003589	0.206159	-6.06814	1.29E-09	2.37E-08
NUSAP1	888.364935	1.843017003	0.303731	6.067932	1.30E-09	2.37E-08
FCN1	399.81569	-2.519254533	0.4153	-6.0661	1.31E-09	2.40E-08
SOCS3	5740.66521	-1.920127845	0.316782	-6.06135	1.35E-09	2.46E-08
PRIM1	273.714078	1.143272152	0.18862	6.061234	1.35E-09	2.46E-08
HAND1	28.4906207	-5.344267504	0.881721	-6.06118	1.35E-09	2.46E-08
ZWINT	587.61035	1.829661807	0.301937	6.05974	1.36E-09	2.48E-08
PPP1R12A	4591.8874	-1.031783137	0.170276	-6.05948	1.37E-09	2.48E-08
WWTR1	2782.39315	-1.387903121	0.229061	-6.05909	1.37E-09	2.49E-08
CCDC184	45.6778549	-1.677400088	0.276896	-6.05787	1.38E-09	2.50E-08
SLC25A23	1063.10579	-1.548448847	0.255612	-6.05782	1.38E-09	2.50E-08
CDH4	14.9200865	-3.727551712	0.615525	-6.05589	1.40E-09	2.53E-08
FLT3	56.9768326	-2.493034064	0.411713	-6.05527	1.40E-09	2.54E-08
LINC0115	4.91646966	-4.598544529	0.759681	-6.05326	1.42E-09	2.57E-08
RPS12P5	5.78095801	-2.753907294	0.454969	-6.05295	1.42E-09	2.57E-08
JUN	9998.50964	-1.370374187	0.226442	-6.05176	1.43E-09	2.59E-08
MKX-AS1	2.56925369	-4.510448697	0.745404	-6.05101	1.44E-09	2.60E-08
ARHGEF1	915.211844	1.357385201	0.224334	6.05074	1.44E-09	2.60E-08
MTCP1	45.0210252	1.235080565	0.204149	6.049911	1.45E-09	2.61E-08
LINC0286	9.0426832	-2.120332	0.35062	-6.04739	1.47E-09	2.65E-08
IL17RB	119.834032	2.149719591	0.3555	6.047025	1.48E-09	2.66E-08
ERV3-1	470.616449	1.392238907	0.230285	6.045734	1.49E-09	2.67E-08
BEGAIN	57.8492707	-2.107954184	0.348826	-6.04299	1.51E-09	2.72E-08
LTBP3	3731.83808	-1.069913867	0.177093	-6.04154	1.53E-09	2.74E-08
POLE2	167.917447	1.617797561	0.267795	6.041189	1.53E-09	2.75E-08
EHD2	2237.47667	-1.798415441	0.297885	-6.03727	1.57E-09	2.81E-08
CNGA3	15.4249954	-3.144873792	0.521145	-6.03454	1.59E-09	2.85E-08
FAM124A	92.1612613	-2.196194967	0.364024	-6.0331	1.61E-09	2.88E-08
ERI2	529.292069	1.023007538	0.169605	6.031697	1.62E-09	2.90E-08
LINC0226	8.28695758	-2.565679971	0.425447	-6.03055	1.63E-09	2.92E-08
INTS2	644.794639	1.072559112	0.177954	6.027181	1.67E-09	2.98E-08
RAC3	213.845571	1.777239799	0.294943	6.025712	1.68E-09	3.00E-08
FGF7	1358.93387	-3.076535756	0.510668	-6.02453	1.70E-09	3.02E-08
CLEC1A	121.782516	-1.571257455	0.260891	-6.02265	1.72E-09	3.05E-08
RORB-AS	2.96099526	-3.121604675	0.518435	-6.02121	1.73E-09	3.07E-08
MLXIP	2278.4423	-1.118530653	0.185895	-6.01701	1.78E-09	3.15E-08
RBM24	82.4269725	-2.783018394	0.462599	-6.01605	1.79E-09	3.17E-08
CHRDL1	548.442733	-3.559006525	0.591599	-6.01591	1.79E-09	3.17E-08
GPT2	701.575852	1.548323706	0.257402	6.015195	1.80E-09	3.18E-08
KLHL41	60.4248498	-2.645922096	0.440009	-6.01333	1.82E-09	3.21E-08

EPHA1	813.150302	1.465345682	0.243711	6.012641	1.83E-09	3.22E-08
ADRA1D	33.2752412	-2.975142154	0.494845	-6.01228	1.83E-09	3.23E-08
AJM1	71.4471413	-1.908452222	0.317628	-6.00845	1.87E-09	3.30E-08
PLD4	113.239614	-2.191781126	0.364789	-6.00835	1.87E-09	3.30E-08
ZHX3	709.068008	-1.109108107	0.184759	-6.00299	1.94E-09	3.40E-08
CENPK	246.390392	1.608508039	0.267971	6.00255	1.94E-09	3.41E-08
STARD13	3.02569674	-2.483172526	0.413771	-6.00131	1.96E-09	3.43E-08
ZBTB41	80.688928	1.669173647	0.27821	5.9997	1.98E-09	3.46E-08
CFAP251	161.03735	1.631645001	0.27206	5.997371	2.01E-09	3.51E-08
ARID5B	3007.69968	-1.213087504	0.202325	-5.99574	2.03E-09	3.54E-08
CSF1	1033.72648	-1.585035687	0.264496	-5.99267	2.06E-09	3.61E-08
F12	79.2251786	1.458146768	0.243468	5.989058	2.11E-09	3.68E-08
MAP1B	3864.41639	-2.584496981	0.431568	-5.98863	2.12E-09	3.69E-08
ANKRD5	26.9401117	-1.834452667	0.306339	-5.98832	2.12E-09	3.69E-08
CREB3L1	976.019082	-2.040818412	0.340903	-5.98651	2.14E-09	3.73E-08
CASP12	15.7627618	-1.564726394	0.261492	-5.98384	2.18E-09	3.78E-08
MIS18A	480.42412	1.198396741	0.200326	5.982246	2.20E-09	3.81E-08
RBKS	198.342715	-1.329205322	0.222237	-5.98103	2.22E-09	3.84E-08
RAB9B	211.954762	-2.223709304	0.372037	-5.97713	2.27E-09	3.93E-08
KIAA1614	37.8649775	1.257614349	0.210456	5.975674	2.29E-09	3.96E-08
PDZRN3-/	3.07314541	-3.475431111	0.581702	-5.97459	2.31E-09	3.99E-08
DUSP5P1	26.1900243	3.295401295	0.551578	5.974495	2.31E-09	3.99E-08
ASF1B	351.908195	1.781635168	0.298302	5.972584	2.34E-09	4.03E-08
HSPG2	10707.4139	-1.671566558	0.279904	-5.97194	2.34E-09	4.04E-08
LMNA	16152.9049	-1.042951516	0.174788	-5.96695	2.42E-09	4.15E-08
CCNA2	480.854936	1.698156673	0.284595	5.966929	2.42E-09	4.15E-08
SBK1	406.491306	2.267668643	0.380076	5.966361	2.43E-09	4.17E-08
LHFPL5	4.13836892	3.232688957	0.542139	5.962847	2.48E-09	4.25E-08
LINC0289	4.13827409	-2.076768374	0.348344	-5.96184	2.49E-09	4.27E-08
ESPN	353.664386	2.387330694	0.400604	5.959324	2.53E-09	4.33E-08
CACNA1(	537.782985	-2.246397965	0.376982	-5.9589	2.54E-09	4.34E-08
ZNF525	300.617559	1.020547502	0.17129	5.957993	2.55E-09	4.35E-08
HSPB8	2339.6355	-2.241202201	0.37619	-5.95763	2.56E-09	4.36E-08
PLPPR5	6.71300745	-2.912624374	0.488987	-5.95644	2.58E-09	4.39E-08
FOXH1	34.0495911	2.186994625	0.367239	5.955243	2.60E-09	4.42E-08
HSF2BP	34.779687	1.282392653	0.215379	5.954109	2.61E-09	4.44E-08
APOD	1517.98489	-2.227130968	0.37408	-5.95363	2.62E-09	4.45E-08
TGFBR3	2502.6142	-1.437945642	0.24163	-5.95103	2.66E-09	4.52E-08
FEN1	611.958869	1.382919494	0.232423	5.949999	2.68E-09	4.55E-08
SVIL	4380.97072	-1.567591617	0.263488	-5.94938	2.69E-09	4.56E-08
CDKL1	170.418436	-1.38393354	0.232652	-5.94852	2.71E-09	4.57E-08
CDK2AP2	5.44140334	1.984236373	0.333571	5.94847	2.71E-09	4.57E-08
WDR72	1394.32185	2.573313203	0.432644	5.947883	2.72E-09	4.58E-08
ZNF436-A	148.550275	1.247892591	0.209844	5.946752	2.74E-09	4.61E-08
ITGA5	6390.41943	-2.223169028	0.373857	-5.94658	2.74E-09	4.61E-08
DNAJB4	960.825409	-1.28969452	0.216972	-5.94405	2.78E-09	4.68E-08
C2CD6	40.3334296	2.628618183	0.442418	5.941476	2.82E-09	4.74E-08
MAD2L1	474.64823	1.739640186	0.292834	5.94071	2.84E-09	4.76E-08
NAPSB	262.887965	-2.240803929	0.377246	-5.9399	2.85E-09	4.78E-08
RSKR	49.1640647	1.152389995	0.194109	5.936832	2.91E-09	4.86E-08
HSPA12B	226.063127	-1.864373514	0.314116	-5.93531	2.93E-09	4.91E-08
GP1BA	8.92302582	-3.299488295	0.556012	-5.9342	2.95E-09	4.94E-08

CPEB1-AS	2.082309	-2.744185331	0.462751	-5.93015	3.03E-09	5.05E-08
ATF3	5144.96109	-1.924230358	0.324533	-5.92922	3.04E-09	5.08E-08
MLLT11	531.349033	1.833327385	0.30935	5.926394	3.10E-09	5.16E-08
NUF2	480.208462	2.213713714	0.373701	5.923758	3.15E-09	5.23E-08
MIR583H(	9.95343967	-1.591505932	0.268773	-5.92137	3.19E-09	5.30E-08
RPL32P1	16.8448222	-2.11225194	0.356742	-5.92096	3.20E-09	5.31E-08
CCDC162	189.383951	2.854179362	0.482239	5.918597	3.25E-09	5.38E-08
SLC9A3R	1197.08947	-1.081877126	0.182794	-5.91856	3.25E-09	5.38E-08
TIMP2	7247.60382	-1.982666463	0.335003	-5.91836	3.25E-09	5.39E-08
PIEZO2	287.616272	-1.646466178	0.27823	-5.91764	3.27E-09	5.41E-08
TNNI3	12.3753887	4.35694201	0.736402	5.916524	3.29E-09	5.44E-08
AQP7P4	2.55945391	-2.763629013	0.467201	-5.91529	3.31E-09	5.48E-08
ITIH6	5.80195739	2.932340721	0.495823	5.914085	3.34E-09	5.51E-08
TMSB15B	229.749931	1.685930411	0.285178	5.911859	3.38E-09	5.58E-08
FAM135B	33.5494386	-3.098674231	0.524431	-5.90864	3.45E-09	5.69E-08
JPT1	3229.80911	1.335303414	0.226003	5.908349	3.46E-09	5.69E-08
PKHD1L1	23.415077	-2.120905276	0.359018	-5.90751	3.47E-09	5.72E-08
KCNQ4	89.9875397	-2.051233535	0.347247	-5.90713	3.48E-09	5.73E-08
STUM	210.185658	-3.21102902	0.543709	-5.90578	3.51E-09	5.77E-08
SLC26A4-	23.1326405	-4.034968982	0.683267	-5.90541	3.52E-09	5.78E-08
MCAM	3686.31896	-1.6759465	0.283938	-5.9025	3.58E-09	5.88E-08
CEBPD	4730.81225	-1.272817058	0.215647	-5.90232	3.58E-09	5.88E-08
BLZF2P	18.3723393	-2.659857552	0.450722	-5.90133	3.61E-09	5.90E-08
GNAO1	288.163478	-2.729341541	0.462499	-5.90129	3.61E-09	5.90E-08
TSPAN18	624.479334	-2.039932535	0.345697	-5.90093	3.61E-09	5.91E-08
LINC0067	6.07004718	-3.457942949	0.586	-5.90093	3.61E-09	5.91E-08
PARVA	2118.70199	-1.160461107	0.196665	-5.9007	3.62E-09	5.91E-08
CD99L2	831.190133	-1.123671444	0.190491	-5.8988	3.66E-09	5.97E-08
PCDHGA	25.6714938	-1.852993909	0.314247	-5.89661	3.71E-09	6.05E-08
MIR202H(	1.57712084	-3.169396727	0.537762	-5.89368	3.78E-09	6.14E-08
COL19A1	71.410785	-3.455243898	0.586288	-5.89342	3.78E-09	6.15E-08
PTGES3L	28.0872264	-1.768204766	0.300103	-5.892	3.82E-09	6.20E-08
DARS1-A	64.8014264	1.26064262	0.213975	5.891536	3.83E-09	6.20E-08
ECSCR	290.274993	-1.665534314	0.282718	-5.89115	3.84E-09	6.21E-08
DSCC1	205.865836	1.675845075	0.284475	5.891002	3.84E-09	6.21E-08
PEAR1	360.444976	-1.336961578	0.226959	-5.89076	3.84E-09	6.22E-08
SNRPB	2394.31987	1.056454209	0.179397	5.888911	3.89E-09	6.28E-08
ETV4	568.702146	1.979047583	0.336261	5.88545	3.97E-09	6.41E-08
CKS1BP3	21.7447825	1.600095996	0.271894	5.885001	3.98E-09	6.42E-08
C14orf180	7.09643393	-3.441104957	0.584886	-5.88338	4.02E-09	6.48E-08
KNDC1	23.6529708	-2.095287513	0.35636	-5.87969	4.11E-09	6.61E-08
KIF26A	161.307768	-2.181114061	0.371068	-5.87793	4.15E-09	6.68E-08
PDE9A-AS	1.58785089	-2.732726153	0.465114	-5.87539	4.22E-09	6.76E-08
NKX3-2	7.20958933	3.089314721	0.525994	5.873289	4.27E-09	6.84E-08
MRGPRX	2.51273975	-4.596384156	0.782602	-5.87321	4.27E-09	6.84E-08
ERG	552.901838	-1.466843495	0.249819	-5.87163	4.32E-09	6.90E-08
SLC6A11	206.68458	2.660122447	0.453509	5.865647	4.47E-09	7.14E-08
KIAA0513	734.743876	-1.48436905	0.253167	-5.86319	4.54E-09	7.23E-08
FCGRT	3005.37587	-1.15745839	0.197548	-5.85911	4.65E-09	7.41E-08
GALNT14	291.261335	1.913871549	0.326662	5.85888	4.66E-09	7.41E-08
SPC24	257.624229	1.658290183	0.283091	5.85779	4.69E-09	7.45E-08
CAVIN4	35.8761809	1.702200083	0.290617	5.8572	4.71E-09	7.46E-08

SFTA1P	22.0467366	-2.869144304	0.489903	-5.85656	4.73E-09	7.49E-08
C18orf54	159.034393	1.084912665	0.185253	5.85639	4.73E-09	7.49E-08
SCNN1G	791.273404	2.817233358	0.481184	5.854791	4.78E-09	7.55E-08
H1-7	1.9268971	-2.95018836	0.503905	-5.85465	4.78E-09	7.55E-08
MIR497H(	21.3973544	-1.723745305	0.294494	-5.85324	4.82E-09	7.61E-08
ITGA9	493.22207	-1.747681463	0.298652	-5.85191	4.86E-09	7.67E-08
COL6A2	20180.5144	-2.260037022	0.386373	-5.84936	4.93E-09	7.77E-08
LRRC32	1071.69366	-1.936992514	0.331283	-5.84694	5.01E-09	7.87E-08
MAP3K14	120.207796	1.083399072	0.185523	5.83969	5.23E-09	8.20E-08
TSPOAP1-	118.926393	-1.779379115	0.30478	-5.83824	5.28E-09	8.27E-08
NIPSNAP1	1460.6411	1.183045651	0.202689	5.836749	5.32E-09	8.34E-08
HSPD1	9018.79434	1.00898613	0.172899	5.835684	5.36E-09	8.38E-08
TCL1A	60.6828848	-3.866325582	0.662587	-5.8352	5.37E-09	8.40E-08
ZSCAN9	598.03763	1.128472019	0.193486	5.832316	5.47E-09	8.54E-08
EGR2	498.48157	-2.02819048	0.347874	-5.83024	5.53E-09	8.64E-08
AHNAK	24559.0671	-1.078993743	0.185078	-5.82993	5.55E-09	8.65E-08
LTBP1	2915.1784	-1.274271685	0.218575	-5.82992	5.55E-09	8.65E-08
RNU6-101	42.1572499	-1.900968988	0.326248	-5.82675	5.65E-09	8.79E-08
TRIM61	5.65731784	-1.989449776	0.341497	-5.82567	5.69E-09	8.84E-08
ZDHHC14	412.651426	-1.059072662	0.181818	-5.82491	5.71E-09	8.87E-08
TMEM273	291.306167	-1.707805515	0.293197	-5.82477	5.72E-09	8.87E-08
UNC5C	154.370794	-1.793784092	0.308351	-5.81734	5.98E-09	9.25E-08
HYAL1	83.0290756	-1.422136776	0.244469	-5.81724	5.98E-09	9.25E-08
TBX4	114.445584	-2.545530895	0.43763	-5.81663	6.00E-09	9.28E-08
FRRS1	357.568299	1.579994865	0.271679	5.815673	6.04E-09	9.32E-08
ANKRD29	164.675548	-2.409293205	0.414431	-5.8135	6.12E-09	9.44E-08
DEFB124	9.68450709	-2.701929424	0.464823	-5.81281	6.14E-09	9.47E-08
VSTM2A	53.4780478	-3.021601173	0.51986	-5.81234	6.16E-09	9.49E-08
ZNF322	190.996521	1.494553358	0.257136	5.812298	6.16E-09	9.49E-08
GRM4	12.8834193	3.521434599	0.605926	5.811661	6.19E-09	9.52E-08
SLITRK2	16.1018584	-3.317528595	0.571122	-5.80879	6.29E-09	9.65E-08
HSP90AA	6.05872567	1.773575756	0.305366	5.808028	6.32E-09	9.69E-08
FBXL7	367.596631	-1.972764397	0.339792	-5.80579	6.41E-09	9.81E-08
ARHGEF3	350.688662	-1.337922855	0.230568	-5.80273	6.52E-09	9.98E-08
TBL1XR1	4958.08425	1.102011771	0.189978	5.800733	6.60E-09	1.01E-07
MCM8	406.857605	1.104812477	0.19047	5.800466	6.61E-09	1.01E-07
SGO2	306.539845	1.433155758	0.247105	5.799791	6.64E-09	1.01E-07
ZDHHC20	520.783289	1.208829726	0.208537	5.796722	6.76E-09	1.03E-07
DLEU1	156.465726	1.077319644	0.185851	5.796672	6.76E-09	1.03E-07
PROK1	23.2743928	-3.110576472	0.536615	-5.79667	6.76E-09	1.03E-07
CCDC138	194.917558	1.22751923	0.211778	5.796252	6.78E-09	1.03E-07
TK1	591.638541	1.865131493	0.321787	5.796175	6.78E-09	1.03E-07
ZNF92	411.575467	1.044894499	0.180331	5.794328	6.86E-09	1.04E-07
SYTL5	311.722322	2.327653557	0.401905	5.791555	6.97E-09	1.06E-07
MIR4451	3.14947687	-2.53193298	0.437255	-5.79051	7.02E-09	1.06E-07
CAMK4	163.072492	-1.538316316	0.265919	-5.78491	7.26E-09	1.10E-07
KIF17	44.3193851	-1.652910325	0.285746	-5.78455	7.27E-09	1.10E-07
EN1	89.3283951	4.856007316	0.839601	5.783711	7.31E-09	1.10E-07
RARRES2	1697.63387	-1.751080177	0.302836	-5.78228	7.37E-09	1.11E-07
TRIM7	317.965174	2.178528161	0.3768	5.781652	7.40E-09	1.11E-07
CFAP20D(	87.2153202	2.365051532	0.40913	5.780682	7.44E-09	1.12E-07
C1orf167	13.8816826	-1.626756208	0.281461	-5.77968	7.48E-09	1.12E-07

LINC0218	1.42587593	-2.695063276	0.466356	-5.77898	7.52E-09	1.13E-07
FHL5	56.0039087	-1.887270439	0.32684	-5.77429	7.73E-09	1.16E-07
SOX4	7970.1655	1.410782593	0.24439	5.772672	7.80E-09	1.17E-07
ZNF497	43.2848823	1.197552693	0.207559	5.769703	7.94E-09	1.19E-07
DNM1P46	11.1777636	-2.321909936	0.402437	-5.76963	7.94E-09	1.19E-07
RNA5SP2(	711.437744	-3.81898372	0.661968	-5.76914	7.97E-09	1.19E-07
TARS1	3125.69442	1.066866692	0.184952	5.768354	8.00E-09	1.19E-07
MFGE8	2313.27526	-1.376158444	0.238687	-5.76554	8.14E-09	1.21E-07
CDH17	19.5921796	-2.536652472	0.440024	-5.7648	8.18E-09	1.22E-07
TMEM13(	47.4282701	-2.270076631	0.39391	-5.76294	8.27E-09	1.23E-07
NDN	548.017061	-1.752935403	0.304175	-5.76292	8.27E-09	1.23E-07
SH3D21	392.819146	1.047802075	0.181827	5.762646	8.28E-09	1.23E-07
CAP2	339.772057	-1.583922674	0.274888	-5.76206	8.31E-09	1.23E-07
GINS4	255.300788	1.370703692	0.237919	5.761208	8.35E-09	1.24E-07
FXYD7	7.74621267	-2.166404139	0.376088	-5.76036	8.39E-09	1.25E-07
LRRK2-D'	3.84591634	-2.068835998	0.359252	-5.75874	8.47E-09	1.26E-07
COA6	619.822964	1.024545204	0.177981	5.756479	8.59E-09	1.27E-07
LINC0099	2.50513119	-2.392113581	0.415642	-5.75523	8.65E-09	1.28E-07
FASN	5223.34649	1.14038497	0.198262	5.751917	8.82E-09	1.30E-07
FIGNL1	319.029081	1.286202821	0.223649	5.750994	8.87E-09	1.31E-07
CYP21A2	9.65332436	-2.443502427	0.424958	-5.74999	8.93E-09	1.32E-07
TRAV25	2.68561792	-2.784273081	0.484258	-5.74957	8.95E-09	1.32E-07
FABP5	7786.66701	1.860079769	0.32372	5.745961	9.14E-09	1.34E-07
LINC0096	34.5800133	2.380683195	0.414454	5.744137	9.24E-09	1.36E-07
PTPRD-A!	19.0827258	-2.957656812	0.514928	-5.74383	9.26E-09	1.36E-07
RASA3	899.063036	-1.454792366	0.253313	-5.74305	9.30E-09	1.36E-07
DACT1	527.350882	-1.985024085	0.345707	-5.74192	9.36E-09	1.37E-07
TMEM25!	51.9315931	-2.504149124	0.43622	-5.74056	9.44E-09	1.38E-07
MIR4506	2.2014412	-3.150666883	0.548888	-5.74009	9.46E-09	1.39E-07
MAL2	5420.88047	1.632786323	0.284477	5.739612	9.49E-09	1.39E-07
RAD54B	140.6967	1.41140242	0.246005	5.737286	9.62E-09	1.41E-07
CHI3L2	217.409341	-2.732100123	0.476268	-5.73647	9.67E-09	1.41E-07
MIR23A	68.9238118	-1.953543602	0.340567	-5.73615	9.69E-09	1.41E-07
LINC0201	24.7535401	3.190064113	0.556135	5.736131	9.69E-09	1.41E-07
ZNF107	683.39739	1.030499142	0.179672	5.735438	9.73E-09	1.42E-07
LINC0096	14.5426927	-2.735403761	0.476933	-5.73541	9.73E-09	1.42E-07
DCST1-A!	33.3282444	1.572525951	0.274333	5.732173	9.92E-09	1.44E-07
JAZF1	419.394039	-1.564201975	0.2729	-5.73177	9.94E-09	1.44E-07
ATG9B	165.944559	2.366653235	0.412953	5.731048	9.98E-09	1.45E-07
GTSE1	264.616993	1.727672554	0.301554	5.729235	1.01E-08	1.46E-07
H2AW	170.318331	1.452518967	0.253734	5.72458	1.04E-08	1.50E-07
CLECL1	28.2545515	-2.1172442	0.369856	-5.72451	1.04E-08	1.50E-07
PDE7B	469.365819	-1.502084019	0.262427	-5.72382	1.04E-08	1.51E-07
LINC0135	53.4395891	-1.719499849	0.300443	-5.72321	1.05E-08	1.51E-07
ANTXR2	1985.52463	-1.464741734	0.25609	-5.71963	1.07E-08	1.54E-07
CHMP4C	846.33271	1.23656569	0.216231	5.718722	1.07E-08	1.55E-07
EML1	534.081702	-1.700011885	0.297276	-5.71863	1.07E-08	1.55E-07
MAGOHB	503.913127	1.009766053	0.176657	5.715963	1.09E-08	1.57E-07
DNAJC9	942.099008	1.267068469	0.221685	5.715635	1.09E-08	1.57E-07
AUNIP	100.53198	2.256676897	0.394833	5.715516	1.09E-08	1.57E-07
ZNF331	1423.10788	-1.327511285	0.232401	-5.71215	1.12E-08	1.60E-07
DCHS1	1299.2031	-1.983929465	0.347373	-5.71123	1.12E-08	1.61E-07

AMOTL2	1269.96288	-1.433275811	0.251115	-5.70764	1.15E-08	1.64E-07
BLM	235.230139	1.345445055	0.235755	5.706962	1.15E-08	1.64E-07
ACP6	669.130526	1.160872108	0.203424	5.706649	1.15E-08	1.65E-07
CX3CR1	96.9186358	-1.90986245	0.334688	-5.7064	1.15E-08	1.65E-07
GPAT3	175.631201	-1.846963856	0.323716	-5.7055	1.16E-08	1.66E-07
CEP76	235.291489	1.020011366	0.178836	5.703608	1.17E-08	1.67E-07
MIR3153	3.24450583	2.402106365	0.42123	5.702599	1.18E-08	1.68E-07
SCARF1	512.650587	-1.267569704	0.222516	-5.69653	1.22E-08	1.74E-07
MGARP	3.78309635	-2.103714534	0.369357	-5.69562	1.23E-08	1.75E-07
CSDC2	200.34735	-2.973808525	0.522129	-5.69555	1.23E-08	1.75E-07
KLF4	2459.36545	-1.659960086	0.291489	-5.69475	1.24E-08	1.75E-07
UBE2S	1300.20409	1.274983405	0.223912	5.694116	1.24E-08	1.76E-07
ZEB2	1299.27472	-1.803040863	0.316669	-5.69378	1.24E-08	1.76E-07
PLPP7	52.2954693	-2.025741877	0.355807	-5.69338	1.25E-08	1.76E-07
ZNF322P1	44.7156542	1.307110955	0.22971	5.69026	1.27E-08	1.79E-07
ANKRD4	32.3846144	-2.232984965	0.392457	-5.68975	1.27E-08	1.80E-07
MMS22L	505.201816	1.358197704	0.23874	5.68902	1.28E-08	1.80E-07
SORBS3	1542.96748	-1.241746982	0.218379	-5.68619	1.30E-08	1.83E-07
MYEOV	200.618679	3.01857506	0.530863	5.686163	1.30E-08	1.83E-07
RANBP1P	16.2519975	1.295343766	0.227842	5.685263	1.31E-08	1.84E-07
LMO7DN	3.31894373	-2.073376414	0.364733	-5.68464	1.31E-08	1.84E-07
KANK3	227.80283	-1.66333429	0.292612	-5.68444	1.31E-08	1.84E-07
MIRLET7	17.500579	-1.623784088	0.285784	-5.68185	1.33E-08	1.87E-07
ROBO4	549.963149	-1.550644078	0.272952	-5.68102	1.34E-08	1.88E-07
LINC0111	10.4248889	-1.460290944	0.257118	-5.67946	1.35E-08	1.89E-07
THSD4	1180.45491	-1.694047611	0.29828	-5.67938	1.35E-08	1.89E-07
PPIAP29	140.866093	1.20344457	0.211911	5.679019	1.35E-08	1.90E-07
GEN1	558.791205	1.039763214	0.183107	5.678448	1.36E-08	1.90E-07
IER2	3528.095	-1.008151701	0.177588	-5.6769	1.37E-08	1.92E-07
RNU6-212	15.4211885	-2.109211175	0.371771	-5.67342	1.40E-08	1.95E-07
CLPSL1	4.03061548	4.366066872	0.769635	5.672908	1.40E-08	1.96E-07
PDLIM7	3602.12833	-1.607021978	0.283435	-5.6698	1.43E-08	1.99E-07
PRUNE2	1297.65461	-3.17887675	0.560688	-5.6696	1.43E-08	1.99E-07
PCDHGA	21.930931	-1.703970505	0.300577	-5.66899	1.44E-08	2.00E-07
AURKB	286.26187	1.706870934	0.301103	5.668735	1.44E-08	2.00E-07
NEURL1E	1076.00789	-1.60327833	0.282876	-5.66777	1.45E-08	2.01E-07
MEIS1	1159.27418	-1.083324123	0.191186	-5.66635	1.46E-08	2.03E-07
RGS2	1882.69404	-1.71079308	0.301958	-5.66566	1.46E-08	2.03E-07
LINC0275	5.59029558	-2.008155339	0.354602	-5.66312	1.49E-08	2.06E-07
LEPR	423.708928	-1.374827291	0.242821	-5.6619	1.50E-08	2.08E-07
ZNF678	134.784152	1.006059817	0.17771	5.661258	1.50E-08	2.08E-07
PDSS1	209.097478	1.028555244	0.181733	5.659692	1.52E-08	2.10E-07
PRAMEN	2.88841383	-3.313675872	0.585635	-5.65826	1.53E-08	2.12E-07
DMRT2	16.5102747	3.63094954	0.641781	5.657611	1.53E-08	2.12E-07
CDKN1C	732.17203	-1.449952251	0.256291	-5.65745	1.54E-08	2.12E-07
NAP1L5	234.241554	-1.164821638	0.205952	-5.65579	1.55E-08	2.14E-07
NHSL2	57.3897274	-1.879068818	0.332372	-5.65351	1.57E-08	2.16E-07
DNASE1L	44.9964803	1.631381459	0.288613	5.652478	1.58E-08	2.18E-07
EPHA5	11.6187955	-2.87700944	0.509015	-5.65211	1.58E-08	2.18E-07
FAM177B	24.7993521	-1.9495343	0.344957	-5.65152	1.59E-08	2.19E-07
MEST	1488.22534	2.148558629	0.380188	5.6513	1.59E-08	2.19E-07
WFDC21F	2093.98523	3.184229098	0.563732	5.64848	1.62E-08	2.22E-07

LINC0146	10.9172582	3.997803616	0.707783	5.648346	1.62E-08	2.22E-07
GALNT15	123.621476	-2.24290493	0.397145	-5.64757	1.63E-08	2.23E-07
ADCY4	589.413408	-1.576944021	0.279234	-5.64738	1.63E-08	2.23E-07
LINC-PIN'	476.712525	-1.091185982	0.19324	-5.6468	1.63E-08	2.24E-07
GFRA3	70.9068896	-2.591225517	0.458904	-5.64655	1.64E-08	2.24E-07
CRHR2	5.08367196	-2.279804207	0.403787	-5.64606	1.64E-08	2.24E-07
GPR34	260.651568	-1.585225513	0.280801	-5.64538	1.65E-08	2.25E-07
RLN2	23.1118934	2.69812378	0.478112	5.643282	1.67E-08	2.27E-07
PLPPR1	18.6187989	3.677857737	0.651749	5.643062	1.67E-08	2.27E-07
SNORA38	5.43176679	1.414412077	0.250733	5.64111	1.69E-08	2.30E-07
MS4A15	5.73639097	4.586683269	0.813116	5.640873	1.69E-08	2.30E-07
SNORD11	1.78952819	-3.39984766	0.602726	-5.64079	1.69E-08	2.30E-07
HEPH	498.834757	-1.921156923	0.340637	-5.63989	1.70E-08	2.31E-07
STX1A	374.196983	1.157708492	0.205405	5.636217	1.74E-08	2.36E-07
PPIAP45	15.0038344	1.986348455	0.352428	5.63618	1.74E-08	2.36E-07
TSHZ3-AS	3.91076905	-2.257953281	0.400639	-5.63588	1.74E-08	2.36E-07
SPNS2	560.841379	-1.631179671	0.289479	-5.63489	1.75E-08	2.37E-07
MZT2A	1474.97909	1.01687244	0.180507	5.633432	1.77E-08	2.39E-07
GAS1RR	13.1573663	-2.622084623	0.465614	-5.63145	1.79E-08	2.42E-07
FAT4	296.757004	-1.755460604	0.311785	-5.63036	1.80E-08	2.43E-07
ACR	11.5979643	-1.780105648	0.316466	-5.62494	1.86E-08	2.50E-07
B4GALNT	429.42677	2.475253156	0.440103	5.624259	1.86E-08	2.51E-07
MOCOS	628.877747	1.369484502	0.243526	5.623555	1.87E-08	2.52E-07
TERT	35.7516001	2.586983667	0.460085	5.62284	1.88E-08	2.53E-07
DSTN	12985.8287	-1.007485129	0.179262	-5.62017	1.91E-08	2.57E-07
CD19	157.012946	-3.225527466	0.574097	-5.61844	1.93E-08	2.59E-07
PRIMA1	167.808579	-2.834885973	0.504609	-5.61799	1.93E-08	2.59E-07
TP53AIP1	51.5213242	2.206163138	0.392846	5.615841	1.96E-08	2.62E-07
LINC0151	46.8031034	4.233914459	0.754237	5.613508	1.98E-08	2.66E-07
EYA1	73.0635873	-2.635779882	0.470039	-5.60758	2.05E-08	2.74E-07
LINC0047	18.9147706	-1.734055578	0.309312	-5.60616	2.07E-08	2.76E-07
SLX4IP	70.9394419	1.235206615	0.220392	5.60459	2.09E-08	2.78E-07
LINC0273	14.0356416	-2.239211833	0.399556	-5.60424	2.09E-08	2.79E-07
SHANK3	1436.7563	-1.458136349	0.26021	-5.60369	2.10E-08	2.80E-07
CAVIN3	662.923543	-1.804515466	0.322077	-5.60275	2.11E-08	2.81E-07
FAM72A	74.1359614	1.3973407	0.249461	5.60145	2.13E-08	2.83E-07
NCAPH	298.727313	1.787820204	0.3192	5.600942	2.13E-08	2.83E-07
HOXC9	60.8302624	2.696858041	0.481508	5.600861	2.13E-08	2.83E-07
GRID2	12.8426559	-2.990096587	0.533885	-5.60063	2.14E-08	2.83E-07
SHISA2	199.12282	2.771179723	0.495635	5.591165	2.26E-08	2.99E-07
MND1	83.2231685	1.544476136	0.276468	5.586453	2.32E-08	3.06E-07
NRIP2	76.1312864	-2.202329339	0.394291	-5.58554	2.33E-08	3.08E-07
LINC0213	4.20381972	-2.693700872	0.48227	-5.58546	2.33E-08	3.08E-07
ENTPD1	1391.67464	-1.572252869	0.281586	-5.58355	2.36E-08	3.11E-07
AICDA	7.61422713	-3.635264583	0.651409	-5.58062	2.40E-08	3.16E-07
CETP	31.5290981	-1.874444027	0.335924	-5.57996	2.41E-08	3.17E-07
MGP	8411.39113	-2.23306229	0.400206	-5.57978	2.41E-08	3.17E-07
CD1B	12.3992339	-2.444909266	0.438407	-5.5768	2.45E-08	3.22E-07
LINC0140	69.5132839	1.078162088	0.193366	5.575754	2.46E-08	3.24E-07
SLC17A7	36.3771024	-1.823904203	0.327144	-5.57524	2.47E-08	3.25E-07
C1QTNF4	18.6152331	-1.460726909	0.262003	-5.57522	2.47E-08	3.25E-07
WNT3	98.4628776	1.575096799	0.282522	5.575134	2.47E-08	3.25E-07

ATP5MF	1353.40557	1.001030324	0.179564	5.574792	2.48E-08	3.25E-07
OTULIN-I	28.5627431	1.414024718	0.253705	5.573491	2.50E-08	3.27E-07
RNF150	89.8849566	-2.173105691	0.389968	-5.57253	2.51E-08	3.28E-07
PRKN	78.9000178	-1.565802959	0.281106	-5.57015	2.55E-08	3.32E-07
KCNA2	5.99547948	-2.231750937	0.400723	-5.56931	2.56E-08	3.34E-07
SEPTIN3	108.138273	2.284730601	0.4103	5.568434	2.57E-08	3.35E-07
PLCL1	146.988962	-1.803943613	0.32397	-5.56824	2.57E-08	3.35E-07
FKBP1BP	1.66906744	-2.928680658	0.525971	-5.56814	2.57E-08	3.35E-07
SLC25A45	284.793886	-1.180737732	0.21212	-5.56636	2.60E-08	3.39E-07
CDC25A	216.232661	1.702657446	0.305945	5.565233	2.62E-08	3.41E-07
MMP1	4296.82974	3.0706114	0.551835	5.564362	2.63E-08	3.42E-07
MAS1LP1	1.15520387	-3.326117555	0.598113	-5.56102	2.68E-08	3.48E-07
SYT14	23.3414366	3.669048776	0.660229	5.557234	2.74E-08	3.55E-07
PYGO1	159.870613	-1.646339296	0.296256	-5.55715	2.74E-08	3.55E-07
CD1D	134.872155	-1.744697597	0.314063	-5.55525	2.77E-08	3.58E-07
NAGS	64.7115753	1.388169294	0.249916	5.554535	2.78E-08	3.59E-07
TP53INP2	1393.52327	-1.329983138	0.239525	-5.55258	2.81E-08	3.63E-07
EMP1	5876.53844	-1.437981836	0.259037	-5.55126	2.84E-08	3.65E-07
LINC0122	68.7134402	2.87778474	0.518478	5.550451	2.85E-08	3.67E-07
DQX1	221.208028	1.813814204	0.326854	5.549318	2.87E-08	3.69E-07
FAM171A	90.217286	1.783660601	0.321446	5.54887	2.88E-08	3.70E-07
MKI67	1347.28606	1.660811293	0.29933	5.548431	2.88E-08	3.71E-07
CAVIN2-A	3.87102865	-2.263975167	0.408054	-5.54823	2.89E-08	3.71E-07
HBB	2024.44156	-2.000264268	0.36055	-5.54782	2.89E-08	3.72E-07
MIR548A	10.6557239	-2.273272161	0.409893	-5.54602	2.92E-08	3.75E-07
MRPL13	1005.54942	1.035582903	0.186728	5.545929	2.92E-08	3.75E-07
MS4A7	1072.19096	-1.339617144	0.241647	-5.54368	2.96E-08	3.80E-07
CENPE	630.616494	1.341378534	0.242004	5.542802	2.98E-08	3.82E-07
VRK1	545.265078	1.090429644	0.196735	5.54264	2.98E-08	3.82E-07
HOMER2	213.625196	1.483106429	0.267656	5.541091	3.01E-08	3.85E-07
S1PR2	78.4847212	-1.493349195	0.269516	-5.54086	3.01E-08	3.85E-07
PDE1A	211.607896	-1.983333847	0.35831	-5.53524	3.11E-08	3.97E-07
H2BC19P	18.3954865	1.153313781	0.20837	5.534924	3.11E-08	3.98E-07
KCNIP4	16.7996984	-3.142191196	0.567812	-5.53386	3.13E-08	4.00E-07
ALPK3	391.607625	-1.560414958	0.282081	-5.5318	3.17E-08	4.04E-07
KCNE4	202.843158	-2.130066428	0.385155	-5.53041	3.19E-08	4.07E-07
PTPRR	347.776185	2.225031307	0.402463	5.528536	3.23E-08	4.11E-07
PYGB	3551.66157	-1.189648705	0.215193	-5.52829	3.23E-08	4.11E-07
RNY4P36	8.67781961	-2.238207696	0.40496	-5.52699	3.26E-08	4.14E-07
KDM6B	3771.06731	-1.076705686	0.194908	-5.52418	3.31E-08	4.20E-07
NFE2L1-D	23.672338	1.243340043	0.225075	5.524111	3.31E-08	4.20E-07
PLEKHO1	1234.60255	-1.499381318	0.271437	-5.52387	3.32E-08	4.20E-07
DBNDD1	248.525532	1.575297732	0.285274	5.522044	3.35E-08	4.24E-07
ELAPOR2	717.304228	1.120804416	0.203023	5.52058	3.38E-08	4.27E-07
BMP6	139.798197	-1.706986532	0.309263	-5.51953	3.40E-08	4.30E-07
MCM2	872.536984	1.447134391	0.262189	5.519434	3.40E-08	4.30E-07
KCNIP3	80.9651862	-1.483798552	0.268905	-5.51792	3.43E-08	4.33E-07
MOB3A	997.452786	-1.103693306	0.20003	-5.51764	3.44E-08	4.34E-07
FAM43A	536.577469	-1.638260267	0.29696	-5.51676	3.45E-08	4.35E-07
FKBP5	1375.7784	-1.5735823	0.285271	-5.51611	3.47E-08	4.37E-07
CELSR3	518.605861	1.844360745	0.334453	5.514564	3.50E-08	4.40E-07
ZNF423	214.161821	-1.569156029	0.284552	-5.51448	3.50E-08	4.40E-07

LINC0123	61.0815731	-1.52174983	0.275962	-5.51435	3.50E-08	4.40E-07
RNU6-853	2.38868137	-2.469644334	0.448016	-5.5124	3.54E-08	4.45E-07
TRIM24	1624.98814	1.176124735	0.213394	5.511506	3.56E-08	4.47E-07
PCOLCE	1663.82253	-1.495937461	0.271445	-5.51102	3.57E-08	4.48E-07
FGFR4	164.265753	-1.77512644	0.322169	-5.50992	3.59E-08	4.50E-07
PPIAP9	4.23653835	2.350869004	0.426703	5.509379	3.60E-08	4.52E-07
CCDC34	565.689296	1.407657373	0.255629	5.506639	3.66E-08	4.58E-07
IFITM2	2014.9382	-1.588156298	0.288444	-5.50595	3.67E-08	4.60E-07
PIP4K2A	1184.20989	-1.084367559	0.19696	-5.50552	3.68E-08	4.60E-07
SIM2	298.460112	2.77891671	0.504773	5.505277	3.69E-08	4.61E-07
BLK	113.17092	-3.294657004	0.598571	-5.50421	3.71E-08	4.63E-07
GPX3	2462.47237	-1.934894804	0.351561	-5.50372	3.72E-08	4.64E-07
PPM1K	256.240899	-1.245493168	0.226319	-5.50326	3.73E-08	4.65E-07
DIAPH3	162.172334	1.69421499	0.307875	5.502937	3.74E-08	4.65E-07
ZBTB46	229.880976	-1.443528934	0.262501	-5.49914	3.82E-08	4.75E-07
ARHGAP2	290.658538	-1.607486979	0.292403	-5.49751	3.85E-08	4.78E-07
LMOD3	21.3202207	-2.377967621	0.432559	-5.49744	3.85E-08	4.78E-07
STRIP2	171.884175	1.608253912	0.292671	5.495084	3.91E-08	4.85E-07
TMPOP2	4.38818983	2.181634861	0.397063	5.494436	3.92E-08	4.86E-07
ERVMER3	119.445759	1.999008526	0.363865	5.493819	3.93E-08	4.87E-07
CCDC102	206.371584	-1.208917809	0.220056	-5.49369	3.94E-08	4.87E-07
FGF11	227.589915	1.710685433	0.311433	5.492942	3.95E-08	4.89E-07
ZNF607	224.234497	1.098592693	0.200047	5.491673	3.98E-08	4.92E-07
LINC0049	28.6019272	3.347120312	0.609499	5.491594	3.98E-08	4.92E-07
C20orf96	446.142839	1.019470615	0.185651	5.491336	3.99E-08	4.93E-07
HOXB6	603.671557	1.953372796	0.355791	5.490222	4.01E-08	4.96E-07
SH3RF3	337.943303	-1.659381481	0.302353	-5.48823	4.06E-08	5.01E-07
CACYBPI	17.4360556	1.068860492	0.194834	5.486009	4.11E-08	5.07E-07
SERPINA	18.5450939	-2.396050795	0.436895	-5.48427	4.15E-08	5.11E-07
S100B	131.457119	-1.985064089	0.36225	-5.47981	4.26E-08	5.23E-07
GTF2IP14	20.0510541	1.130756688	0.206376	5.479107	4.27E-08	5.25E-07
MFAP5	703.810669	-3.269302646	0.596719	-5.4788	4.28E-08	5.26E-07
GPR15	84.5242435	-2.676635644	0.488555	-5.47868	4.29E-08	5.26E-07
TRY2P	1.24076733	-3.613929735	0.659749	-5.47773	4.31E-08	5.28E-07
IGDCC4	318.824462	-1.795370971	0.327842	-5.47633	4.34E-08	5.32E-07
PPP1R15A	5111.13575	-1.029169128	0.188013	-5.47393	4.40E-08	5.39E-07
RTN4R	54.535957	1.554539627	0.284048	5.472798	4.43E-08	5.42E-07
MIR3194	1.5701218	-3.559486019	0.650602	-5.47107	4.47E-08	5.47E-07
ASNSP6	2.81514686	-2.789881572	0.510045	-5.46987	4.50E-08	5.50E-07
CCNP	23.6768569	2.072656702	0.378946	5.469524	4.51E-08	5.51E-07
FNDC5	57.4500798	-1.753338843	0.320625	-5.46851	4.54E-08	5.54E-07
SLC29A2	326.534953	1.202848004	0.219994	5.467635	4.56E-08	5.56E-07
FAM162B	78.5906562	-1.632695213	0.298704	-5.46593	4.60E-08	5.61E-07
HABP4	483.964068	-1.068665366	0.195587	-5.46389	4.66E-08	5.67E-07
GRIK1-AS	8.47138483	-1.838138626	0.336517	-5.46224	4.70E-08	5.72E-07
P4HA1	2332.71203	1.115975806	0.204347	5.461169	4.73E-08	5.75E-07
TLE1P1	9.36798538	-1.401456985	0.256652	-5.46054	4.75E-08	5.76E-07
SLC5A9	11.8910105	-2.120903856	0.38841	-5.46048	4.75E-08	5.76E-07
SNORD72	3.23962339	1.902840044	0.348749	5.456189	4.86E-08	5.90E-07
DTNA	561.643319	-2.446894267	0.4486	-5.45452	4.91E-08	5.95E-07
LINC0088	17.1745248	-1.585879591	0.290848	-5.45261	4.96E-08	6.01E-07
KCTD12	2896.20985	-1.402753086	0.257265	-5.45256	4.96E-08	6.01E-07

ALDH2	1122.02996	-1.592561196	0.292087	-5.45236	4.97E-08	6.01E-07
TRBV7-3	8.92040335	-2.549570794	0.467613	-5.45231	4.97E-08	6.01E-07
PNPLA7	308.210746	-1.49442116	0.274094	-5.45223	4.97E-08	6.01E-07
AOX3P	1.22842817	-2.885801276	0.529294	-5.45217	4.98E-08	6.01E-07
TPST1	790.842359	-1.1376837	0.208823	-5.44809	5.09E-08	6.13E-07
RN7SL689	32.1435771	-1.220146822	0.224068	-5.44544	5.17E-08	6.22E-07
MORN5	14.8031612	-3.411875041	0.626656	-5.44458	5.19E-08	6.24E-07
SLC45A1	46.2538206	-1.428918257	0.262457	-5.4444	5.20E-08	6.25E-07
DOK5	51.9635039	-2.097611656	0.385285	-5.44431	5.20E-08	6.25E-07
NR5A2	114.284119	-1.36712379	0.251207	-5.44223	5.26E-08	6.31E-07
NCOA7	3215.04608	-1.105018932	0.203062	-5.44177	5.28E-08	6.32E-07
RNF212	29.747086	-2.02527411	0.372284	-5.44013	5.32E-08	6.38E-07
RRAS	856.500664	-1.204089118	0.221343	-5.43991	5.33E-08	6.38E-07
PRR34	9.10210014	-1.494654001	0.274767	-5.43971	5.34E-08	6.38E-07
HNRNPA1	1.31434223	-3.056864363	0.561986	-5.43939	5.35E-08	6.39E-07
PYCR3	300.655088	1.042808129	0.191724	5.439103	5.35E-08	6.40E-07
COL13A1	196.539534	-1.83077294	0.336621	-5.43868	5.37E-08	6.41E-07
TNFAIP3	3071.34056	-1.61978047	0.297855	-5.43815	5.38E-08	6.42E-07
USHBP1	86.6841331	-1.430110039	0.262992	-5.43784	5.39E-08	6.43E-07
C3	13235.6331	-2.094718687	0.385375	-5.43553	5.46E-08	6.51E-07
ABCG2	171.014215	-1.24173672	0.228471	-5.43498	5.48E-08	6.53E-07
RASL11A	288.8298	-1.540719062	0.283498	-5.43467	5.49E-08	6.53E-07
FSTL3	1069.62143	-1.595802764	0.293635	-5.43464	5.49E-08	6.53E-07
NUPR1	1090.87143	-1.600054118	0.294496	-5.43319	5.54E-08	6.58E-07
LYL1	161.921533	-1.450204488	0.267336	-5.42466	5.81E-08	6.88E-07
STEAP4	911.971649	-1.995307598	0.367985	-5.42225	5.89E-08	6.97E-07
KLK6	16.9972444	4.628043461	0.853596	5.421819	5.90E-08	6.98E-07
FBXO41	601.731027	1.223311493	0.225633	5.421688	5.90E-08	6.98E-07
CELF5	28.9545068	3.267159234	0.602646	5.421361	5.91E-08	6.99E-07
GDNF-AS	11.977375	-1.797782088	0.331625	-5.42112	5.92E-08	6.99E-07
MS4A1	531.629917	-3.189530004	0.588354	-5.4211	5.92E-08	6.99E-07
IGF2	12118.9829	3.048025022	0.56253	5.418425	6.01E-08	7.09E-07
IL21-AS1	13.729216	-2.300895684	0.424678	-5.41798	6.03E-08	7.11E-07
HMGB1P1	1.49950993	3.077045464	0.567975	5.417574	6.04E-08	7.12E-07
PHKG1	54.8739856	-1.345979416	0.248483	-5.41679	6.07E-08	7.14E-07
KRT24	39.3331594	-3.528919544	0.651753	-5.41451	6.15E-08	7.23E-07
AQP7P2	3.52652696	-2.20188516	0.406711	-5.41389	6.17E-08	7.25E-07
HID1-AS1	6.79411227	-2.162985503	0.399593	-5.41297	6.20E-08	7.29E-07
SMG7-AS	7.73968229	1.313408132	0.242665	5.412439	6.22E-08	7.30E-07
LINC0181	373.137103	2.627371226	0.485582	5.410769	6.28E-08	7.37E-07
B3GALNT	1116.74304	1.353539991	0.250262	5.408486	6.36E-08	7.45E-07
IGHA2	8584.73311	-3.024963849	0.559339	-5.4081	6.37E-08	7.46E-07
LRP1	7721.62816	-1.326049484	0.245211	-5.40778	6.38E-08	7.47E-07
OCIAD2	1573.37012	1.068021738	0.197524	5.407044	6.41E-08	7.50E-07
RPL10P7	2.9668439	-3.358639909	0.621741	-5.402	6.59E-08	7.70E-07
PCLAF	235.724231	1.652080707	0.30608	5.397543	6.76E-08	7.87E-07
MIDN	4531.01479	-1.02213788	0.189375	-5.39743	6.76E-08	7.87E-07
ANGPTL2	1701.6114	-1.787765362	0.331226	-5.39742	6.76E-08	7.87E-07
CD163L1	375.307845	-1.535917606	0.284704	-5.39479	6.86E-08	7.98E-07
LINC0052	11.1221242	3.578072241	0.663358	5.393881	6.90E-08	8.01E-07
LRCH2	204.039839	-1.696563474	0.314607	-5.39265	6.94E-08	8.06E-07
NT5DC3	629.656394	-1.830363229	0.33947	-5.39183	6.97E-08	8.10E-07

TIMD4	30.8090352	-2.408685491	0.446994	-5.38863	7.10E-08	8.24E-07
ASPG	56.2609158	-2.097345862	0.389245	-5.38824	7.12E-08	8.25E-07
HES2	218.5022	2.38804101	0.443252	5.387544	7.14E-08	8.28E-07
IGFBP7	1853.62807	-1.59799706	0.296804	-5.38402	7.28E-08	8.42E-07
CSPG4P12	18.2265253	-1.683300142	0.312692	-5.38325	7.32E-08	8.45E-07
KLHL2P1	10.7340855	1.794890137	0.333449	5.382795	7.33E-08	8.47E-07
MZT1	663.291624	1.011769817	0.187997	5.38183	7.37E-08	8.51E-07
MAFF	2364.65411	-1.303984385	0.242306	-5.38156	7.38E-08	8.52E-07
CLEC11A	486.947574	-1.537078733	0.285967	-5.37501	7.66E-08	8.81E-07
PERP	14644.9011	1.300691916	0.242116	5.372189	7.78E-08	8.94E-07
KCNJ12	37.654205	-2.468463827	0.45966	-5.37019	7.87E-08	9.03E-07
MPZ	140.099707	-1.079832831	0.201143	-5.36848	7.94E-08	9.10E-07
PDE10A	712.193501	1.64431349	0.306439	5.365879	8.06E-08	9.22E-07
SDHAF3	323.907156	1.063868882	0.198349	5.363624	8.16E-08	9.33E-07
MAPK8IP	119.606711	2.176865808	0.405945	5.362464	8.21E-08	9.38E-07
PGLYRP3	11.774401	4.643482205	0.86626	5.360382	8.30E-08	9.48E-07
SLC45A2	13.8327157	2.780175779	0.518932	5.357492	8.44E-08	9.63E-07
GALNT1	6689.96343	1.241689681	0.231773	5.357348	8.45E-08	9.63E-07
ARHGEF2	814.945871	-1.666079309	0.311043	-5.35643	8.49E-08	9.68E-07
BNC2	449.398343	-2.178861673	0.406785	-5.3563	8.49E-08	9.68E-07
RNA5SP3	3.3724006	4.136674055	0.772331	5.356088	8.50E-08	9.68E-07
MIR181A2	30.8202389	1.604095801	0.299491	5.35607	8.51E-08	9.68E-07
MYCT1	344.211392	-1.522513629	0.284473	-5.35205	8.70E-08	9.89E-07
SLC22A3	194.097955	-2.286088835	0.427729	-5.34472	9.06E-08	1.03E-06
SIGLEC16	15.1463515	-1.919005121	0.359108	-5.34381	9.10E-08	1.03E-06
DNM1P47	14.6114232	-2.09110399	0.391317	-5.34376	9.10E-08	1.03E-06
STOM	4456.19519	-1.336099805	0.250084	-5.3426	9.16E-08	1.04E-06
COL6A4P	68.7495976	-1.465122744	0.274262	-5.34206	9.19E-08	1.04E-06
NKAIN1	11.1262826	2.558067603	0.4789	5.341552	9.22E-08	1.04E-06
KY	11.4129218	-2.222963638	0.416195	-5.34115	9.24E-08	1.05E-06
SMYD3	664.567498	1.131339966	0.211838	5.340596	9.26E-08	1.05E-06
SFRP5	5.67460615	-3.275635893	0.613382	-5.34029	9.28E-08	1.05E-06
LINC0223	2.63956088	2.674607907	0.500898	5.339624	9.31E-08	1.05E-06
CDKN3	234.122707	1.722896088	0.322828	5.336883	9.46E-08	1.07E-06
MUCL1	13.5199906	3.358152507	0.629455	5.335017	9.55E-08	1.08E-06
ZFHX4	458.430103	-2.093384665	0.392509	-5.33334	9.64E-08	1.09E-06
C1S	9819.64997	-1.819725687	0.341336	-5.33118	9.76E-08	1.10E-06
ITGAD	11.3729343	-2.060687808	0.386553	-5.33093	9.77E-08	1.10E-06
CLEC14A	548.20111	-1.270764599	0.238403	-5.33031	9.80E-08	1.10E-06
CCND2	2442.55372	-1.833694769	0.344038	-5.32992	9.83E-08	1.10E-06
MIR5690	11.7611426	-2.404437966	0.451152	-5.32955	9.85E-08	1.10E-06
CPA4	45.3108583	2.843740143	0.533815	5.327199	9.97E-08	1.12E-06
TRAV23D	3.32338997	-2.862389055	0.537768	-5.32272	1.02E-07	1.14E-06
GPR183	645.338495	-1.922756886	0.361449	-5.31959	1.04E-07	1.16E-06
GAPDHP1	202.667953	1.391645022	0.261765	5.316389	1.06E-07	1.18E-06
GIMAP1	274.146741	-1.70187961	0.320152	-5.31585	1.06E-07	1.18E-06
ACTG1P2	4.44196204	2.688382835	0.505833	5.314759	1.07E-07	1.19E-06
ZNF726	193.670427	1.574111759	0.296208	5.314219	1.07E-07	1.19E-06
MRPL36	701.975689	1.006123429	0.189353	5.313471	1.08E-07	1.20E-06
KLRB1	179.883249	-2.255781885	0.424567	-5.31313	1.08E-07	1.20E-06
FCRLB	396.228718	2.450000194	0.461133	5.313006	1.08E-07	1.20E-06
UNC5C-A	23.1654515	-1.958664064	0.368846	-5.31025	1.09E-07	1.21E-06

DDIAS	202.747473	1.528284692	0.28782	5.309864	1.10E-07	1.22E-06
IGFBP6	1310.41787	-1.880792766	0.354347	-5.30777	1.11E-07	1.23E-06
SAMD4A	1215.97014	-1.703505822	0.321088	-5.30542	1.12E-07	1.24E-06
C5orf34	106.185933	1.191679972	0.224618	5.305355	1.12E-07	1.24E-06
SNORD19	32.0129343	1.409633404	0.26572	5.304951	1.13E-07	1.25E-06
PCAT6	121.742337	1.461305457	0.275531	5.30359	1.14E-07	1.26E-06
DHH	18.6047216	-1.909851342	0.360108	-5.30356	1.14E-07	1.26E-06
KLHL4	26.9099528	-2.377378408	0.448397	-5.30195	1.15E-07	1.26E-06
CLPSL2	2.81320052	3.987933765	0.752198	5.301705	1.15E-07	1.27E-06
TPPP	287.842461	-1.931330427	0.364347	-5.3008	1.15E-07	1.27E-06
CYP4F8	3286.51129	3.394045099	0.640426	5.299666	1.16E-07	1.28E-06
DDN-AS1	16.443886	1.884692514	0.355625	5.299662	1.16E-07	1.28E-06
KCTD16	23.2366476	2.68970499	0.507544	5.299455	1.16E-07	1.28E-06
SMIM36	1.53727802	3.569705293	0.673992	5.296364	1.18E-07	1.30E-06
FERMT1	1302.09227	1.109834395	0.20958	5.295522	1.19E-07	1.31E-06
RMI2	353.273688	1.416855402	0.267592	5.294843	1.19E-07	1.31E-06
ZSCAN12	29.6197692	1.595280345	0.301305	5.294565	1.19E-07	1.31E-06
DSP	6327.76952	1.633999797	0.308641	5.29418	1.20E-07	1.31E-06
CALHM3	8.42230235	3.543781578	0.669585	5.292503	1.21E-07	1.32E-06
RNU4-51F	17.0404905	-1.534968917	0.290061	-5.29188	1.21E-07	1.33E-06
DIPK1A	372.628069	-1.147331638	0.216837	-5.29121	1.22E-07	1.33E-06
CSF3	343.778208	-3.067585638	0.579875	-5.29008	1.22E-07	1.34E-06
PDE4D	1119.84268	-1.216877853	0.230066	-5.28926	1.23E-07	1.34E-06
LINC0280	131.407185	-1.150498692	0.217525	-5.28905	1.23E-07	1.34E-06
CENPX	949.244015	1.114638727	0.210756	5.288753	1.23E-07	1.35E-06
MIR4664	13.143897	1.329419826	0.251606	5.283733	1.27E-07	1.38E-06
LINC0257	35.0952787	3.261781107	0.617355	5.283476	1.27E-07	1.38E-06
BMPER	109.687683	-1.957163558	0.370489	-5.28265	1.27E-07	1.39E-06
TAS2R38	4.38739389	3.297312172	0.624409	5.280691	1.29E-07	1.40E-06
RANBP3L	91.30092	-3.448817353	0.653697	-5.27586	1.32E-07	1.44E-06
FGD2	573.752318	-1.456772979	0.276149	-5.27532	1.33E-07	1.44E-06
NLGN1	128.254896	-2.248067034	0.426187	-5.27484	1.33E-07	1.44E-06
PAM	2689.80887	-1.219369366	0.231299	-5.27182	1.35E-07	1.47E-06
SCN2B	16.4839423	-2.574159305	0.488334	-5.27131	1.35E-07	1.47E-06
SHCBP1	240.705157	1.512764782	0.28705	5.27004	1.36E-07	1.48E-06
PITPNM2	590.262282	-1.176046595	0.223172	-5.26968	1.37E-07	1.48E-06
PPP1R16E	468.942162	-1.921107782	0.364601	-5.26908	1.37E-07	1.49E-06
MIR27B	17.5300226	-1.706783005	0.323943	-5.26877	1.37E-07	1.49E-06
PTGER1	36.2218483	-1.624787292	0.308508	-5.2666	1.39E-07	1.50E-06
LINC0227	18.8520795	-2.094838476	0.398013	-5.26324	1.42E-07	1.53E-06
KIAA004(	20.4941302	-1.046182774	0.198827	-5.26179	1.43E-07	1.54E-06
EMSLR	26.3646568	2.32904972	0.442657	5.261519	1.43E-07	1.54E-06
ZNF385D	141.40365	-2.501105222	0.475482	-5.26014	1.44E-07	1.55E-06
KIF26B	475.036838	1.96622153	0.373905	5.258618	1.45E-07	1.56E-06
NUDT1	373.133992	1.079378923	0.205277	5.258154	1.46E-07	1.57E-06
LINC0281	7.9389432	-2.600156258	0.494876	-5.25416	1.49E-07	1.60E-06
CX3CL1	1051.94774	-1.706994669	0.325025	-5.25189	1.51E-07	1.62E-06
HYLS1	179.037684	1.052822585	0.200466	5.251863	1.51E-07	1.62E-06
TBX5-AS1	34.4066922	-2.006336242	0.382032	-5.25175	1.51E-07	1.62E-06
RAG1	51.8255934	1.537688959	0.292848	5.250802	1.51E-07	1.63E-06
GGCT	1904.61385	1.085085886	0.206655	5.250707	1.52E-07	1.63E-06
ST8SIA1	135.031645	-1.974423531	0.376051	-5.25041	1.52E-07	1.63E-06

P2RY8	310.232349	-2.023083	0.385348	-5.25002	1.52E-07	1.63E-06
RAET1K	6.21402104	2.161110425	0.411708	5.249137	1.53E-07	1.64E-06
RSPO3	217.389866	-2.790824124	0.532092	-5.245	1.56E-07	1.67E-06
LINC0191	11.4558116	-1.706617584	0.325417	-5.2444	1.57E-07	1.68E-06
ERC2	36.5785461	2.559376708	0.488065	5.243922	1.57E-07	1.68E-06
GNGT1	68.5934497	2.198393048	0.41928	5.243257	1.58E-07	1.68E-06
ADAM2	3.79554815	4.773250144	0.910526	5.242299	1.59E-07	1.69E-06
DHRS7C	0.81997067	-3.26995179	0.623946	-5.24076	1.60E-07	1.71E-06
TMEM191	30.2430951	1.337653516	0.255251	5.240552	1.60E-07	1.71E-06
CTD-3080	9.08940283	-2.507312275	0.478498	-5.23996	1.61E-07	1.71E-06
CBFA2T3	209.467468	-1.522219901	0.290713	-5.23616	1.64E-07	1.75E-06
FCMR	421.100359	-1.801581934	0.344086	-5.23585	1.64E-07	1.75E-06
PALM	603.043412	-1.910496011	0.365074	-5.23318	1.67E-07	1.77E-06
ADIPOQ	23.4243631	-4.964473362	0.948819	-5.23226	1.67E-07	1.78E-06
CCDC178	33.3377863	-2.299268223	0.439594	-5.23044	1.69E-07	1.80E-06
LINC0148	1.40327982	-2.576042405	0.492525	-5.23028	1.69E-07	1.80E-06
ASCL5	3.04620634	2.385758227	0.456146	5.230254	1.69E-07	1.80E-06
ZNF761	685.602125	1.011216637	0.193473	5.226644	1.73E-07	1.83E-06
CLEC17A	45.0669889	-2.974211268	0.56941	-5.22332	1.76E-07	1.86E-06
CMTM5	10.5039149	-2.619912437	0.50158	-5.22332	1.76E-07	1.86E-06
CADM3-A	82.9109299	-2.713219486	0.519532	-5.22243	1.77E-07	1.87E-06
C8orf88	133.437048	-1.920728093	0.367852	-5.22147	1.78E-07	1.87E-06
PRR5-ARI	9.81369355	1.443115671	0.276405	5.221021	1.78E-07	1.88E-06
LINC0270	1.0589808	-3.706481908	0.710106	-5.21961	1.79E-07	1.89E-06
ZNF165	263.392135	1.442639538	0.276412	5.219166	1.80E-07	1.89E-06
RN7SKP1	12.2115176	-1.959977195	0.375549	-5.21897	1.80E-07	1.89E-06
USP51	100.568885	-1.072647972	0.205534	-5.21883	1.80E-07	1.89E-06
NETO2	412.946737	1.449696024	0.277794	5.218607	1.80E-07	1.90E-06
VWF	4239.24029	-1.527173511	0.292914	-5.21372	1.85E-07	1.94E-06
EBF3	124.035516	-1.76939156	0.339446	-5.21259	1.86E-07	1.95E-06
LINC0202	2.94506855	-3.239573533	0.621736	-5.21053	1.88E-07	1.97E-06
MMP10	956.247529	2.918063961	0.560406	5.207054	1.92E-07	2.01E-06
FAM83B	274.818163	1.461125196	0.280712	5.205066	1.94E-07	2.03E-06
RPSAP69	10.1868798	1.792155372	0.344497	5.202237	1.97E-07	2.06E-06
SFRP2	4053.7895	-2.987782355	0.574383	-5.20173	1.97E-07	2.07E-06
LY86	123.808363	-1.527100002	0.293608	-5.20115	1.98E-07	2.07E-06
SNORD14	24.4121435	1.838768869	0.353683	5.198912	2.00E-07	2.10E-06
ALOXE3	17.6735517	2.324681049	0.447213	5.198147	2.01E-07	2.10E-06
PPP1R14E	84.4567087	1.532909869	0.29491	5.197899	2.02E-07	2.10E-06
RHPN1-A	20.7164436	1.688040309	0.324835	5.196602	2.03E-07	2.11E-06
TRARG1	4.71060064	-3.50803059	0.67527	-5.195	2.05E-07	2.13E-06
GHRL	31.3489804	-1.318881402	0.253939	-5.19368	2.06E-07	2.14E-06
ACTN1-A	10.9629159	-1.824826648	0.351398	-5.19305	2.07E-07	2.15E-06
NXPH1	2.10680714	4.243940256	0.818169	5.187118	2.14E-07	2.22E-06
CASKIN1	73.2953801	1.838440342	0.354427	5.187081	2.14E-07	2.22E-06
MPEG1	1520.50644	-1.810406241	0.349067	-5.18641	2.14E-07	2.22E-06
PRRX2	121.995419	-1.764761594	0.340334	-5.18538	2.16E-07	2.24E-06
ZYX	5091.99046	-1.09427271	0.211057	-5.18472	2.16E-07	2.24E-06
TP73	157.313806	1.621695302	0.312803	5.184403	2.17E-07	2.24E-06
FBN2	477.763991	2.460109589	0.474617	5.183353	2.18E-07	2.26E-06
RTL5	254.987978	-1.573263181	0.303772	-5.17909	2.23E-07	2.31E-06
TCAM1P	55.0875078	3.60884314	0.69697	5.177901	2.24E-07	2.32E-06

MYRIP	73.1188779	-1.855535635	0.358439	-5.17671	2.26E-07	2.33E-06
RADX	495.05436	1.57875496	0.305052	5.175365	2.27E-07	2.35E-06
BNC2-AS1	8.6808523	-2.244426578	0.433766	-5.17428	2.29E-07	2.36E-06
IGHV1OR	38.7237997	-2.219193422	0.42902	-5.1727	2.31E-07	2.37E-06
MIOX	11.0514165	2.596561588	0.50208	5.171612	2.32E-07	2.39E-06
MIR23AH	2087.0605	-1.340954695	0.259306	-5.17132	2.32E-07	2.39E-06
SLAMF9	29.5429685	3.201173808	0.6191	5.170692	2.33E-07	2.40E-06
ZNF610	122.363524	1.305344524	0.252499	5.169709	2.34E-07	2.41E-06
ANKRD44	273.333424	-1.361006953	0.263289	-5.16925	2.35E-07	2.41E-06
H19	39469.0111	2.788990587	0.539632	5.168318	2.36E-07	2.42E-06
THEM5	9.20515119	3.44178361	0.666057	5.167403	2.37E-07	2.43E-06
GPR158	57.8133078	2.860466181	0.554105	5.16232	2.44E-07	2.50E-06
CYCSP6	4.62495792	3.960907052	0.76747	5.160992	2.46E-07	2.51E-06
ELOCP2	26.6934347	1.006921694	0.195113	5.160698	2.46E-07	2.52E-06
SELP	506.932829	-2.241616369	0.434366	-5.16067	2.46E-07	2.52E-06
RN7SKP2	2.46490024	-2.832174165	0.548809	-5.16058	2.46E-07	2.52E-06
CLSPN	292.088272	1.520456427	0.294892	5.155981	2.52E-07	2.58E-06
GRHL2	1615.16402	1.177125073	0.22831	5.155819	2.53E-07	2.58E-06
HLA-DPB	3754.57493	-1.649776126	0.320029	-5.15508	2.54E-07	2.58E-06
ABCA9	362.811801	-1.811611028	0.351552	-5.15319	2.56E-07	2.61E-06
STAB1	3525.12439	-1.669422052	0.324059	-5.1516	2.58E-07	2.62E-06
GULP1	409.146852	-1.563287403	0.303509	-5.1507	2.60E-07	2.64E-06
GZMM	52.2445708	-2.007207463	0.389806	-5.14925	2.62E-07	2.65E-06
ACVR1C	28.3637127	2.347062445	0.456172	5.145121	2.67E-07	2.71E-06
LINC0189	0.96107451	-2.748741458	0.534678	-5.14093	2.73E-07	2.77E-06
NFKBIZ	2387.74885	-1.075026146	0.20916	-5.13972	2.75E-07	2.78E-06
TMEM132	22.3228611	-1.88293122	0.366381	-5.13927	2.76E-07	2.79E-06
JAML	770.420837	-1.834537458	0.356965	-5.13926	2.76E-07	2.79E-06
ALPL	350.801652	-1.554480963	0.302525	-5.13836	2.77E-07	2.80E-06
CCL2	2291.11618	-2.059392028	0.400803	-5.13817	2.77E-07	2.80E-06
NMUR1	25.8215071	-2.049426929	0.398902	-5.13767	2.78E-07	2.81E-06
CREM	897.311825	-1.144133105	0.222884	-5.13332	2.85E-07	2.87E-06
CNGB3	28.8233601	2.481329549	0.48338	5.133288	2.85E-07	2.87E-06
ADAMTS	8.25926757	-1.440465788	0.280657	-5.13248	2.86E-07	2.88E-06
ENKUR	8.02241071	1.913333543	0.372889	5.131114	2.88E-07	2.90E-06
KCNIP1	9.84205263	-2.613897211	0.509537	-5.12994	2.90E-07	2.91E-06
FABP5P7	1418.27444	1.812120222	0.353265	5.129628	2.90E-07	2.91E-06
GRIN2D	161.41151	2.265361928	0.441763	5.128005	2.93E-07	2.94E-06
ZFPM2-AS	236.234058	2.18636473	0.426411	5.127368	2.94E-07	2.94E-06
EPHX4	81.2555952	1.841287796	0.35914	5.126937	2.94E-07	2.95E-06
FOS	17572.8111	-1.373128588	0.267925	-5.12505	2.97E-07	2.98E-06
TVP23A	34.4682262	-1.979021566	0.386185	-5.12454	2.98E-07	2.98E-06
FLI1	682.112843	-1.485619961	0.289981	-5.12316	3.00E-07	3.00E-06
CABCOC	20.4150914	-1.89083619	0.369196	-5.1215	3.03E-07	3.03E-06
RNF216-I	13.2239556	-1.525093469	0.297855	-5.12025	3.05E-07	3.04E-06
CD4	1439.17419	-1.572611503	0.307144	-5.12012	3.05E-07	3.04E-06
FER1L5	14.8217905	-1.831503342	0.357734	-5.11974	3.06E-07	3.05E-06
CHRNA1	13.4851732	2.586677463	0.505418	5.117901	3.09E-07	3.08E-06
LYPD6	475.417612	1.581117437	0.309085	5.115473	3.13E-07	3.11E-06
SIX1	261.397517	1.590907129	0.311	5.115462	3.13E-07	3.11E-06
RNF125	288.211847	-1.420189839	0.277639	-5.11523	3.13E-07	3.12E-06
RASGRP4	118.370538	-1.874705914	0.366812	-5.11081	3.21E-07	3.18E-06

AGMAT	119.781793	1.123732355	0.219891	5.11041	3.21E-07	3.19E-06
INSC	25.9727033	-1.854150159	0.36295	-5.10855	3.25E-07	3.22E-06
LINC0084	517.323559	1.169581173	0.229076	5.105638	3.30E-07	3.27E-06
CDH19	192.64736	-3.095568462	0.606317	-5.10553	3.30E-07	3.27E-06
RNU6-790	7.55446733	-1.56912244	0.307375	-5.10491	3.31E-07	3.28E-06
SYPL1P2	12.9990685	1.458949755	0.285812	5.104586	3.32E-07	3.28E-06
ZNF841	764.322017	1.127776707	0.221019	5.102628	3.35E-07	3.31E-06
AHCTF1P	59.6750613	1.033251557	0.202534	5.101622	3.37E-07	3.33E-06
PNMA2	120.242408	-1.836182692	0.359955	-5.10115	3.38E-07	3.33E-06
KISS1R	14.7587738	2.602666543	0.510239	5.100875	3.38E-07	3.34E-06
ZNF252P-	27.9198001	1.240676689	0.243236	5.100718	3.38E-07	3.34E-06
CPVL	987.124106	-1.592719666	0.312318	-5.09967	3.40E-07	3.35E-06
TP63	3107.30304	1.619029525	0.31749	5.09946	3.41E-07	3.36E-06
PLSCR4	486.225386	-1.589866948	0.311968	-5.09625	3.46E-07	3.41E-06
SLCO1A2	72.1742868	3.956330526	0.776393	5.095784	3.47E-07	3.42E-06
FABP5P1	16.3331784	2.362712254	0.463713	5.095203	3.48E-07	3.42E-06
SCRG1	71.2859904	-3.47981694	0.683057	-5.09447	3.50E-07	3.44E-06
NAALAD:	71.5882883	-2.039650484	0.400411	-5.09389	3.51E-07	3.44E-06
FBLN1	24493.2585	-1.815843602	0.356502	-5.09351	3.51E-07	3.45E-06
MNX1-AS	22.3486227	2.577905878	0.506389	5.090762	3.57E-07	3.49E-06
MFNG	291.59395	-1.575784115	0.309597	-5.08979	3.58E-07	3.51E-06
TPO	39.5709911	-3.183672372	0.625623	-5.08881	3.60E-07	3.53E-06
SLC6A8	3287.3607	1.614524311	0.317293	5.088438	3.61E-07	3.53E-06
UCN2	54.0159428	1.819622463	0.357754	5.086237	3.65E-07	3.57E-06
RD3L	1.89743153	-4.386219598	0.862437	-5.08585	3.66E-07	3.58E-06
RBMS3-A	3.21170507	-2.038128916	0.40078	-5.0854	3.67E-07	3.58E-06
AKAP12	2564.317	-1.782677028	0.350629	-5.08423	3.69E-07	3.60E-06
DBI	5101.69684	1.30715025	0.257221	5.081827	3.74E-07	3.64E-06
RERE-AS	11.2050904	-1.244301238	0.244918	-5.08049	3.76E-07	3.67E-06
SLC24A4	19.8944393	-2.033126288	0.400385	-5.07793	3.82E-07	3.71E-06
NBL1	730.663986	-1.201005743	0.236518	-5.07787	3.82E-07	3.71E-06
RNASE7	56.0829564	2.263946999	0.445891	5.077356	3.83E-07	3.72E-06
FGF16	2.59260122	-2.912240302	0.573614	-5.077	3.83E-07	3.72E-06
ITGB2-AS	161.544684	-2.00749241	0.395695	-5.07333	3.91E-07	3.79E-06
VCL	6045.47702	-1.126136351	0.222084	-5.07076	3.96E-07	3.84E-06
PODXL2	655.906929	1.638372079	0.323224	5.068851	4.00E-07	3.87E-06
MIR4653	25.5027674	1.816339464	0.358352	5.068586	4.01E-07	3.87E-06
DSCAML	27.5595039	-2.36159019	0.466151	-5.06615	4.06E-07	3.92E-06
RPL4P7	21.8433027	-2.126443408	0.419763	-5.06582	4.07E-07	3.93E-06
LINC0142	7.70101598	1.580346034	0.311975	5.065611	4.07E-07	3.93E-06
PCDHB9	95.9842822	1.512121577	0.298532	5.065184	4.08E-07	3.94E-06
LHCGR	2.69105524	-2.404009979	0.474695	-5.06433	4.10E-07	3.95E-06
HELLS	781.098508	1.003718406	0.198236	5.063249	4.12E-07	3.97E-06
ST13P15	15.7915327	-1.077729724	0.213019	-5.05932	4.21E-07	4.05E-06
LINC0282	5.9050654	-2.227810645	0.440419	-5.05839	4.23E-07	4.07E-06
ARHGEF1	420.026192	-1.505311711	0.297634	-5.0576	4.25E-07	4.08E-06
SNORD11	1.4354719	-3.037754497	0.600769	-5.05644	4.27E-07	4.10E-06
HTR2B	53.6269735	-1.570538305	0.310631	-5.05597	4.28E-07	4.10E-06
NMB	335.780123	1.186207678	0.234645	5.055319	4.30E-07	4.12E-06
CNR2	41.801051	-2.802520965	0.554388	-5.05516	4.30E-07	4.12E-06
TFIP11-D	101.747357	1.134201206	0.224407	5.054213	4.32E-07	4.13E-06
HOXB8	355.381263	2.410733363	0.476976	5.054201	4.32E-07	4.13E-06

NEIL3	42.340477	1.806604976	0.35745	5.054144	4.32E-07	4.13E-06
GAD1	201.595375	2.183370256	0.432022	5.053845	4.33E-07	4.14E-06
SPACA6P-	13.5970673	1.792699517	0.354795	5.052778	4.35E-07	4.15E-06
RNU6-10F	3.39473465	-2.035489168	0.40291	-5.05197	4.37E-07	4.17E-06
ZNF730	30.513771	2.231746409	0.44178	5.051718	4.38E-07	4.17E-06
IFFO1	480.475265	-1.561646309	0.309142	-5.05154	4.38E-07	4.17E-06
AMACR	67.7531806	1.693968377	0.335467	5.049577	4.43E-07	4.21E-06
HBA1	158.140612	-2.014188787	0.398929	-5.04899	4.44E-07	4.22E-06
PTRH2	805.239524	1.017112272	0.201458	5.048751	4.45E-07	4.23E-06
NEB	143.338675	1.719586456	0.340789	5.045894	4.51E-07	4.29E-06
HPR	1.01540761	-3.195999356	0.633949	-5.04141	4.62E-07	4.38E-06
SNORA60	9.56737403	1.086282948	0.215501	5.040733	4.64E-07	4.39E-06
ARHGAP1	319.212365	-1.657803898	0.329018	-5.03864	4.69E-07	4.44E-06
ADTRP	168.342463	-1.831362977	0.363568	-5.03719	4.72E-07	4.47E-06
ACTN1	8263.76828	-1.4235078	0.282639	-5.03648	4.74E-07	4.48E-06
NBPF6	15.8034375	3.187950374	0.63315	5.035061	4.78E-07	4.51E-06
SORCS2	198.026078	-2.297930416	0.45648	-5.03402	4.80E-07	4.53E-06
AMIGO2	2788.08036	1.239300289	0.246199	5.033736	4.81E-07	4.54E-06
HNRNPCI	2.61611925	-2.402243914	0.477266	-5.03335	4.82E-07	4.55E-06
BRCA2	227.159936	1.178700142	0.234199	5.032898	4.83E-07	4.55E-06
CRH	1088.84743	3.918774393	0.77866	5.032717	4.84E-07	4.55E-06
DPF1	28.550734	2.367101614	0.470344	5.032703	4.84E-07	4.55E-06
NEURL1	146.779227	-2.801740301	0.556751	-5.0323	4.85E-07	4.56E-06
LSR	3812.75427	1.080907343	0.214808	5.031971	4.85E-07	4.57E-06
PDE4B	696.137493	-1.754289605	0.348655	-5.0316	4.86E-07	4.57E-06
TAF7L	32.4133663	1.861774497	0.370263	5.028246	4.95E-07	4.65E-06
DNMBP-A	6.48113383	-1.668746689	0.332082	-5.0251	5.03E-07	4.72E-06
LINC0067	3.54079533	-2.147498731	0.427377	-5.02484	5.04E-07	4.73E-06
GIMAP5	571.554344	-1.637544835	0.325915	-5.02446	5.05E-07	4.73E-06
DLL1	382.954308	-1.291218259	0.25699	-5.02439	5.05E-07	4.73E-06
LINC0289	104.391481	1.702462601	0.338893	5.023595	5.07E-07	4.75E-06
ANO4	29.0274081	-1.999556035	0.398345	-5.01966	5.18E-07	4.84E-06
MRGPRF-	16.034807	-2.300437399	0.45845	-5.01786	5.22E-07	4.88E-06
CDCA7	390.352985	1.453993436	0.289893	5.015627	5.29E-07	4.93E-06
NFIL3	1044.48837	-1.167681562	0.232816	-5.01547	5.29E-07	4.93E-06
MMP19	1109.21303	-1.619440707	0.323006	-5.01365	5.34E-07	4.97E-06
RADIL	46.1650017	-2.237299287	0.446251	-5.01355	5.34E-07	4.97E-06
CADM4	708.716526	1.077135257	0.214853	5.013367	5.35E-07	4.98E-06
SMIM24	12.7254837	1.919250347	0.382837	5.013225	5.35E-07	4.98E-06
PCDHB18	14.9734851	-1.25553599	0.250463	-5.01287	5.36E-07	4.99E-06
RFTN2	116.066534	-1.112333773	0.221925	-5.01221	5.38E-07	5.00E-06
PYCR1	729.656705	1.614183518	0.322095	5.011509	5.40E-07	5.01E-06
CERS3	102.419266	2.609941352	0.520805	5.011356	5.40E-07	5.02E-06
AADAT	399.692574	1.042310882	0.207992	5.011297	5.41E-07	5.02E-06
NDUFA4L	1360.43287	2.093726573	0.417856	5.010636	5.43E-07	5.03E-06
IL1RL2	90.1540764	1.124958808	0.224559	5.009633	5.45E-07	5.06E-06
PXYLP1	599.699055	1.026504498	0.204948	5.0086	5.48E-07	5.08E-06
PANK1	180.975942	1.013095197	0.202304	5.007783	5.51E-07	5.10E-06
IGSF21	48.3558345	-1.894197732	0.378352	-5.00644	5.54E-07	5.13E-06
PWWP3B	169.958051	2.45229058	0.489924	5.005451	5.57E-07	5.16E-06
RFC3	409.521997	1.043723895	0.208558	5.004467	5.60E-07	5.18E-06
E2F1	411.632738	1.46703878	0.293147	5.004443	5.60E-07	5.18E-06

PCDH18	448.046116	-1.38575375	0.276911	-5.00433	5.61E-07	5.18E-06
TBX15	49.3225124	2.236086168	0.446984	5.002604	5.66E-07	5.22E-06
ADH1A	2.756306	-2.595593889	0.519065	-5.00052	5.72E-07	5.27E-06
REN	134.02708	-2.418924248	0.483964	-4.99815	5.79E-07	5.33E-06
HHIP	331.460071	-2.250864546	0.450442	-4.99701	5.82E-07	5.36E-06
GAL	28.3784788	3.282384463	0.657272	4.993948	5.92E-07	5.44E-06
RBP5	73.4147651	-1.448402878	0.290193	-4.99116	6.00E-07	5.51E-06
IGLVIVOI	1.79069865	3.613686318	0.72411	4.990519	6.02E-07	5.52E-06
LSAMP	100.189902	-1.496082098	0.299796	-4.99034	6.03E-07	5.53E-06
LY9	116.815075	-2.159990854	0.433182	-4.98634	6.15E-07	5.64E-06
H2AC11	55.7862895	1.822799629	0.365592	4.985882	6.17E-07	5.65E-06
ARHGAP2	582.375382	-1.320141842	0.264993	-4.98181	6.30E-07	5.76E-06
LRRC15	223.060958	3.125846904	0.627456	4.98178	6.30E-07	5.76E-06
LDLRAD4	1.03217281	-3.443017359	0.691239	-4.98094	6.33E-07	5.78E-06
DGUOK-A	61.2906002	1.263725202	0.253858	4.978077	6.42E-07	5.85E-06
XYLB	182.116374	1.126770683	0.226422	4.976429	6.48E-07	5.90E-06
OLFM2	258.431333	1.893337914	0.380678	4.973591	6.57E-07	5.98E-06
OR7E94P	1.14799248	-2.862744507	0.575655	-4.97302	6.59E-07	5.99E-06
COL6A1	15110.569	-1.804465738	0.362865	-4.97283	6.60E-07	6.00E-06
HOXB5	340.079272	1.938614846	0.390013	4.970645	6.67E-07	6.06E-06
FOXI2	19.368506	-2.222939131	0.447332	-4.96933	6.72E-07	6.10E-06
SYNE1-AS	0.89821711	-2.761205476	0.555719	-4.96871	6.74E-07	6.11E-06
C4B	318.454247	-1.910793326	0.384627	-4.96792	6.77E-07	6.14E-06
OLFML2A	741.026374	-1.368240944	0.275513	-4.96616	6.83E-07	6.19E-06
PSMD10P	8.07097425	-1.56694846	0.315539	-4.96594	6.84E-07	6.19E-06
RNA5-8SP	126.792671	-1.592424512	0.32079	-4.96407	6.90E-07	6.25E-06
SENCR	20.3300398	-1.535230264	0.309269	-4.96405	6.90E-07	6.25E-06
ARL5AP2	1.95703745	-4.161382429	0.838708	-4.96166	6.99E-07	6.32E-06
SOAT2	5.76927767	-1.894473966	0.382076	-4.95837	7.11E-07	6.42E-06
DCHS2	23.2267284	-2.150474273	0.433748	-4.95788	7.13E-07	6.43E-06
OR52N4	4.37213338	-1.99607263	0.402916	-4.95407	7.27E-07	6.55E-06
HOXB9	165.440453	2.217987181	0.447751	4.953615	7.28E-07	6.56E-06
GRID1	31.0436956	-1.814073578	0.366286	-4.95262	7.32E-07	6.59E-06
PGAP1	781.056874	1.007525451	0.203575	4.949164	7.45E-07	6.70E-06
KRT79	6.88766568	2.449868589	0.495183	4.947396	7.52E-07	6.75E-06
ENPP1	145.376839	-1.664249963	0.336535	-4.94526	7.60E-07	6.82E-06
JCHAIN	10891.2733	-2.653803111	0.536747	-4.94423	7.64E-07	6.85E-06
LCN2	9459.7597	-2.941094426	0.595109	-4.94211	7.73E-07	6.92E-06
ARL5C	2.19959098	-2.239666134	0.45328	-4.94102	7.77E-07	6.96E-06
MSL3P1	159.815486	1.178449329	0.238713	4.93668	7.95E-07	7.11E-06
RIC3	327.890235	-2.011863403	0.407546	-4.93653	7.95E-07	7.11E-06
ADGRE5	1797.66735	-1.081614669	0.219106	-4.93649	7.95E-07	7.11E-06
RAMP2	379.347375	-1.264023842	0.25624	-4.93296	8.10E-07	7.23E-06
CLEC4GP	22.7120969	-1.908296805	0.387023	-4.9307	8.19E-07	7.31E-06
PDE6C	1.28037786	-3.627079088	0.735859	-4.92904	8.26E-07	7.36E-06
MIR4258	3.78733129	1.385400308	0.281071	4.929004	8.26E-07	7.36E-06
FMOD	1038.23277	-1.700337702	0.344996	-4.92858	8.28E-07	7.37E-06
MYOZ3	24.3917204	-1.649482795	0.334684	-4.92847	8.29E-07	7.38E-06
SRGAP3	766.556619	-1.254072238	0.254513	-4.92734	8.34E-07	7.41E-06
ADORA2I	1.53641136	-2.841091143	0.576823	-4.92541	8.42E-07	7.48E-06
CFAP53	77.0040447	1.219404847	0.247591	4.925081	8.43E-07	7.49E-06
SUMO2P1	60.6639539	1.057794943	0.214916	4.921907	8.57E-07	7.60E-06

EFEMP2	1357.95562	-1.058801338	0.215221	-4.91961	8.67E-07	7.68E-06
VPREB3	38.760904	-2.444754603	0.49699	-4.91913	8.69E-07	7.69E-06
H2BW2	5.63374594	4.279415967	0.870059	4.918534	8.72E-07	7.71E-06
FABP6	260.353098	2.137790702	0.43474	4.917396	8.77E-07	7.75E-06
LINC0261	12.5784744	-1.920961597	0.390698	-4.91675	8.80E-07	7.77E-06
ITGA1	1755.61541	-1.34640848	0.273998	-4.91394	8.93E-07	7.88E-06
NGB	3.81784493	4.403264328	0.896187	4.913333	8.95E-07	7.90E-06
PCDHB8	41.2652898	2.102749733	0.428054	4.91235	9.00E-07	7.94E-06
ULBP1	26.0278541	2.136933701	0.435158	4.910709	9.07E-07	8.00E-06
FAM111B	252.933051	1.664206417	0.339015	4.908942	9.16E-07	8.06E-06
ATP2A1-A	30.2487307	1.482907826	0.302121	4.908319	9.19E-07	8.08E-06
GRHL1	2201.58691	1.388998567	0.283004	4.908052	9.20E-07	8.09E-06
FAM238A	4.13039998	-1.892317911	0.38565	-4.90683	9.26E-07	8.13E-06
SH2D3C	333.532373	-1.512127937	0.308171	-4.90678	9.26E-07	8.13E-06
OTOF	48.9887842	1.716625458	0.349852	4.906723	9.26E-07	8.13E-06
DSG3	1060.71547	3.467803442	0.706793	4.906395	9.28E-07	8.14E-06
NDRG1	14467.2998	1.388606061	0.283049	4.905878	9.30E-07	8.16E-06
KIF4B	3.25750264	1.620366666	0.330307	4.905635	9.31E-07	8.16E-06
DKK3	1844.53442	-1.248392028	0.254662	-4.90215	9.48E-07	8.30E-06
MIF	598.343445	1.31296626	0.267836	4.902134	9.48E-07	8.30E-06
CPQ	1012.75292	-1.299314075	0.265075	-4.90169	9.50E-07	8.31E-06
MEG9	51.4301252	-2.251556911	0.459435	-4.90071	9.55E-07	8.35E-06
FOXP4-AS	10.3410983	1.853478113	0.378254	4.90009	9.58E-07	8.37E-06
SLC46A2	90.1918663	-2.276199907	0.464555	-4.89974	9.60E-07	8.38E-06
FIRRE	71.2548739	2.046757761	0.417819	4.898675	9.65E-07	8.42E-06
ZNF367	230.078768	1.381978254	0.282121	4.898524	9.66E-07	8.43E-06
JAM3	1055.17774	-1.932182996	0.394455	-4.89836	9.66E-07	8.43E-06
NRROS	165.797217	-1.58973506	0.324654	-4.8967	9.75E-07	8.50E-06
NBEAP3	7.05822293	2.719080023	0.555402	4.895699	9.80E-07	8.53E-06
TRBV23-1	5.23225839	-2.494156615	0.509779	-4.89263	9.95E-07	8.66E-06
GBGT1	126.372598	-1.271575653	0.260023	-4.89024	1.01E-06	8.76E-06
USP2	100.303581	-1.524966097	0.311914	-4.88906	1.01E-06	8.80E-06
KBTBD13	2.11071695	-2.664575395	0.545218	-4.88718	1.02E-06	8.88E-06
GLI1	155.806165	-1.782419773	0.364799	-4.88603	1.03E-06	8.93E-06
ITGB1BP2	83.527934	-1.20891631	0.247467	-4.88516	1.03E-06	8.96E-06
MXRA8	1266.05493	-1.604818878	0.328528	-4.88488	1.03E-06	8.97E-06
CYSLTR1	191.34888	-1.667337495	0.341383	-4.88406	1.04E-06	9.01E-06
CD209	433.794998	-1.953746176	0.400037	-4.88391	1.04E-06	9.01E-06
PLPP4	21.5562006	2.565766411	0.525483	4.882681	1.05E-06	9.06E-06
CBLN1	37.7921186	-3.518115585	0.720664	-4.88177	1.05E-06	9.10E-06
GLT8D2	447.485799	-1.652475763	0.338637	-4.87979	1.06E-06	9.18E-06
EPM2A	250.658644	-1.048876276	0.214984	-4.87885	1.07E-06	9.22E-06
DNASE1L	144.283392	-2.038948379	0.418015	-4.87769	1.07E-06	9.27E-06
LINC0281	3.89285521	-2.896432529	0.593923	-4.87678	1.08E-06	9.30E-06
FAM241B	156.923759	1.103030293	0.226252	4.875234	1.09E-06	9.37E-06
TENT5C	851.266236	-1.620553429	0.332697	-4.87096	1.11E-06	9.56E-06
CLIC2	459.758671	-1.337926698	0.274704	-4.87043	1.11E-06	9.59E-06
NMU	66.4132206	2.085302073	0.428204	4.869882	1.12E-06	9.61E-06
DHCR7	816.704659	1.188924536	0.244196	4.868735	1.12E-06	9.66E-06
SULT4A1	54.1711776	2.889301389	0.593508	4.86818	1.13E-06	9.68E-06
WNT5B	284.283759	-1.535022327	0.315341	-4.86782	1.13E-06	9.70E-06
FCRL1	79.2992238	-3.255051158	0.668954	-4.86588	1.14E-06	9.78E-06

TOB2P1	15.0444272	1.495752943	0.307646	4.861926	1.16E-06	9.97E-06
ZNF254	834.260533	1.062460699	0.218532	4.861797	1.16E-06	9.97E-06
IL3RA	283.751176	-1.453974266	0.299069	-4.86167	1.16E-06	9.98E-06
ZNF239	205.389478	1.284171718	0.264145	4.861612	1.16E-06	9.98E-06
NUP62CL	47.3483012	1.285678079	0.264494	4.860893	1.17E-06	1.00E-05
TRAPPC2	0.91786966	-2.953912889	0.607764	-4.86029	1.17E-06	1.00E-05
CD37	867.736157	-1.995828066	0.410759	-4.85888	1.18E-06	1.01E-05
CDC42EP	1754.75607	-1.235643557	0.254359	-4.85787	1.19E-06	1.01E-05
TBC1D27	34.805104	-2.728953059	0.561832	-4.85724	1.19E-06	1.02E-05
HEY1	411.619075	1.66406659	0.342706	4.855667	1.20E-06	1.02E-05
DNM1P51	4.41552685	-2.364808436	0.487202	-4.85386	1.21E-06	1.03E-05
EBI3	86.6092424	-1.715697489	0.353472	-4.85384	1.21E-06	1.03E-05
SNX29P2	4.45330433	-2.137951465	0.440736	-4.85087	1.23E-06	1.05E-05
PPIAP54	7.58377632	1.475576351	0.304228	4.850233	1.23E-06	1.05E-05
EBF2	45.2458077	-2.043927549	0.421498	-4.8492	1.24E-06	1.06E-05
TRIM50	3.9577522	-1.801367132	0.371478	-4.84919	1.24E-06	1.06E-05
CD79A	386.963273	-2.542840112	0.524385	-4.84919	1.24E-06	1.06E-05
RPLP0P2	59.4936051	2.472502396	0.510037	4.847692	1.25E-06	1.06E-05
TCEAL7	126.684748	-1.753055775	0.36169	-4.84685	1.25E-06	1.07E-05
TGFB3	1071.15488	-1.866938452	0.385192	-4.84678	1.25E-06	1.07E-05
SRRM3	257.007476	1.414886856	0.291925	4.846746	1.26E-06	1.07E-05
RMI1	347.012523	1.07636262	0.222127	4.845709	1.26E-06	1.07E-05
MSC	368.141088	-1.994801989	0.411831	-4.84374	1.27E-06	1.08E-05
CXCR1	98.9523472	-2.143065098	0.442656	-4.84138	1.29E-06	1.09E-05
DGKG	126.852609	-1.698727927	0.350941	-4.8405	1.30E-06	1.10E-05
ZNF528-A	311.259661	1.332849693	0.275361	4.840376	1.30E-06	1.10E-05
ADAMTS	1651.50089	-2.10920167	0.435932	-4.83837	1.31E-06	1.11E-05
SYNE3	93.7999688	-1.458674317	0.301601	-4.83644	1.32E-06	1.12E-05
DLX6	81.0354423	2.18065518	0.451088	4.834209	1.34E-06	1.13E-05
RGS20	33.5739466	2.711306814	0.560905	4.833804	1.34E-06	1.13E-05
CAV1	4263.06086	-1.522629833	0.315063	-4.83278	1.35E-06	1.13E-05
ADGRL4	753.360687	-1.259873899	0.260732	-4.83206	1.35E-06	1.14E-05
BEND4	14.6505426	-2.141410481	0.443167	-4.83206	1.35E-06	1.14E-05
AKT3-IT1	22.9253428	-2.031266373	0.420429	-4.83141	1.36E-06	1.14E-05
TRAJ38	1.27510706	-3.007857465	0.622584	-4.83125	1.36E-06	1.14E-05
HMGCS1	3055.95619	1.156640887	0.23954	4.828601	1.37E-06	1.15E-05
RERG-IT1	2.15437962	-2.184847064	0.452523	-4.82814	1.38E-06	1.16E-05
PSAT1	699.412381	1.311315759	0.271665	4.826962	1.39E-06	1.16E-05
SLC43A1	220.983451	-1.105504495	0.229034	-4.82681	1.39E-06	1.16E-05
SGCD	351.212419	-2.395701697	0.496377	-4.82637	1.39E-06	1.17E-05
COL4A4	231.646382	-1.996296912	0.413668	-4.82584	1.39E-06	1.17E-05
ZNF887P	9.90076691	1.990386284	0.412491	4.825288	1.40E-06	1.17E-05
LINC0119	21.9426544	-1.717500378	0.356011	-4.8243	1.40E-06	1.18E-05
MYLK2	9.1536513	1.814885693	0.376371	4.822065	1.42E-06	1.19E-05
PPM1N	360.535128	2.113573264	0.438744	4.817331	1.45E-06	1.22E-05
VENTX	68.6706553	-1.724347769	0.358062	-4.81578	1.47E-06	1.22E-05
TEK	291.414336	-1.473445081	0.30597	-4.81566	1.47E-06	1.22E-05
TMTC1	584.569196	-1.522440973	0.316161	-4.81539	1.47E-06	1.23E-05
CYP27A1	424.841274	-1.540239175	0.319865	-4.81527	1.47E-06	1.23E-05
CHDH	107.931447	1.225686855	0.25461	4.813978	1.48E-06	1.23E-05
SOBP	523.797424	-1.879210281	0.390616	-4.81089	1.50E-06	1.25E-05
ABCC8	17.1506825	-2.833820146	0.589143	-4.81007	1.51E-06	1.26E-05

COLEC11	131.967246	-1.423803421	0.29602	-4.80982	1.51E-06	1.26E-05
IGFL2	8.95466499	2.964937665	0.616859	4.806505	1.54E-06	1.28E-05
SNORA77	22.7598899	-1.582724613	0.329309	-4.80619	1.54E-06	1.28E-05
RBMY2JP	3.16952194	3.373783076	0.702121	4.805133	1.55E-06	1.28E-05
TRBV5-4	6.3535798	-2.407531306	0.501095	-4.80454	1.55E-06	1.29E-05
CALHM5	182.103328	-1.581228848	0.329204	-4.80319	1.56E-06	1.30E-05
FGF13-AS	4.77351246	-1.924002933	0.400626	-4.8025	1.57E-06	1.30E-05
NUDCP2	5.94080682	1.249659457	0.260237	4.802	1.57E-06	1.30E-05
LINC0225	3.45951426	2.834467145	0.590594	4.799352	1.59E-06	1.32E-05
VDAC2P2	5.2143767	-2.445102123	0.509634	-4.79776	1.60E-06	1.33E-05
HTR2C	43.7773976	2.952084662	0.615555	4.79581	1.62E-06	1.34E-05
GIMAP6	623.295627	-1.346444444	0.280838	-4.79437	1.63E-06	1.35E-05
MIR938	12.9982008	-1.481238324	0.308979	-4.79398	1.64E-06	1.35E-05
ZC3H12D	189.217767	-1.997597023	0.416777	-4.79296	1.64E-06	1.35E-05
WDFY4	384.184105	-1.812912594	0.378253	-4.79286	1.64E-06	1.36E-05
LINC0167	30.6526212	-1.791493638	0.374086	-4.78899	1.68E-06	1.38E-05
ZP3	66.3236463	1.122739113	0.234482	4.788172	1.68E-06	1.39E-05
TNFRSF1:	44.5441136	-2.848925144	0.595378	-4.78507	1.71E-06	1.41E-05
FSTL1	8044.71798	-1.293994808	0.27045	-4.7846	1.71E-06	1.41E-05
RTKN2	322.488682	1.317485686	0.275462	4.782828	1.73E-06	1.42E-05
NXPE4	0.90892853	-2.64520478	0.553178	-4.78183	1.74E-06	1.43E-05
SYDE1	470.09941	-1.204135375	0.251885	-4.7805	1.75E-06	1.43E-05
MIR4652	2.27807769	2.481444031	0.519221	4.779166	1.76E-06	1.44E-05
CD48	467.6721	-1.901389535	0.397924	-4.77827	1.77E-06	1.45E-05
PITX2	260.078637	-1.690893926	0.353919	-4.77763	1.77E-06	1.45E-05
PDIA2	27.432222	2.256684387	0.472537	4.775674	1.79E-06	1.46E-05
PRR36	240.627985	1.916048363	0.401223	4.775518	1.79E-06	1.46E-05
RNU6-762	6.86379495	1.422094608	0.297805	4.775256	1.79E-06	1.47E-05
TSPAN32	87.1596785	-1.97569525	0.413776	-4.77479	1.80E-06	1.47E-05
VIM	20145.9162	-1.583375872	0.331662	-4.77407	1.81E-06	1.47E-05
TPD52	2230.83768	1.072547621	0.224664	4.774015	1.81E-06	1.47E-05
GRTP1-AS	1.61591878	-2.297476558	0.481255	-4.77392	1.81E-06	1.47E-05
C3orf70	319.188146	-1.933950689	0.405159	-4.77331	1.81E-06	1.48E-05
CCNE1	333.664836	1.289808867	0.270248	4.772684	1.82E-06	1.48E-05
CLIC4	5126.41842	-1.401662204	0.293704	-4.77236	1.82E-06	1.48E-05
HSPE1P2	47.5868133	1.655334139	0.346862	4.772311	1.82E-06	1.48E-05
PABPC3	79.2669925	1.08319808	0.227068	4.770377	1.84E-06	1.49E-05
STAP1	77.4886543	-2.273060476	0.476676	-4.76856	1.86E-06	1.51E-05
CNTN1	635.282464	-2.403765787	0.504164	-4.76783	1.86E-06	1.51E-05
TAL1	110.724771	-1.234909627	0.259217	-4.76401	1.90E-06	1.54E-05
RN7SL32I	2.01463599	-2.062899711	0.433176	-4.76227	1.91E-06	1.55E-05
BCL2L15	117.722009	-1.806556956	0.379444	-4.76107	1.93E-06	1.56E-05
C1orf162	338.567201	-1.285867325	0.270086	-4.76096	1.93E-06	1.56E-05
RNA5SP9:	9.20069612	-1.909891547	0.401251	-4.75984	1.94E-06	1.57E-05
KIAA1549	165.468972	2.143256762	0.450311	4.759508	1.94E-06	1.57E-05
C4A	358.757583	-1.819235126	0.382328	-4.75831	1.95E-06	1.58E-05
GRB7	1513.32843	1.361304844	0.286143	4.757432	1.96E-06	1.58E-05
RNASE6	277.940809	-1.451236082	0.305202	-4.755	1.98E-06	1.60E-05
RAB11FIF	1261.55274	1.138293451	0.239506	4.752679	2.01E-06	1.62E-05
MIR370	0.99059863	-3.074361991	0.64688	-4.7526	2.01E-06	1.62E-05
IL6	781.845834	-2.478092458	0.521553	-4.75137	2.02E-06	1.63E-05
TRPM2-A	68.3068791	2.484695439	0.523105	4.749896	2.04E-06	1.64E-05

ZNF521	251.475805	-1.642727303	0.346002	-4.74774	2.06E-06	1.65E-05
CCND2-A	3.2925933	-1.964045146	0.413702	-4.74748	2.06E-06	1.66E-05
GPR78	282.735064	2.730037221	0.575195	4.746283	2.07E-06	1.66E-05
DCT	5.81710899	-2.179830993	0.459299	-4.74599	2.07E-06	1.67E-05
CFHR3	32.6781513	1.852647115	0.390362	4.745972	2.08E-06	1.67E-05
GIMAP8	483.124928	-1.391099963	0.293158	-4.74523	2.08E-06	1.67E-05
NKAIN2	75.0679992	2.446703906	0.515736	4.744097	2.09E-06	1.68E-05
GPR135	61.8197713	-1.008837397	0.212709	-4.74279	2.11E-06	1.69E-05
NOVA2	138.683854	-1.364575018	0.287719	-4.74273	2.11E-06	1.69E-05
ADAMTS	112.872691	-1.323301207	0.279077	-4.74171	2.12E-06	1.69E-05
IGHV2-5	401.063978	-3.012917764	0.635425	-4.74158	2.12E-06	1.69E-05
RN7SL574	2.69917801	-1.638262966	0.345514	-4.74152	2.12E-06	1.69E-05
LINC0112	6.85797157	-2.203856526	0.464863	-4.74087	2.13E-06	1.70E-05
STING1	1276.85206	-1.033077417	0.21791	-4.74084	2.13E-06	1.70E-05
FAM180A	61.6602224	-2.473935861	0.521858	-4.74063	2.13E-06	1.70E-05
UGT1A10	499.529628	2.774721506	0.585557	4.738604	2.15E-06	1.72E-05
SEMA6B	601.884199	-1.556266594	0.328507	-4.73739	2.16E-06	1.72E-05
CLMP	362.691894	-1.688492464	0.356516	-4.73609	2.18E-06	1.73E-05
THEM6	1481.50459	1.074268704	0.226851	4.735565	2.18E-06	1.74E-05
TMEM255	187.635852	-1.288774976	0.272155	-4.73545	2.19E-06	1.74E-05
ATP8B2	1074.01548	-1.511647336	0.319279	-4.73456	2.20E-06	1.74E-05
FAM234B	479.983888	1.115130421	0.235703	4.731092	2.23E-06	1.77E-05
KCNN3	44.1710851	-1.585409227	0.335126	-4.73079	2.24E-06	1.77E-05
GAB2	812.450893	-1.090612662	0.230624	-4.72897	2.26E-06	1.79E-05
TXN	5533.87347	1.016488218	0.215097	4.72572	2.29E-06	1.81E-05
RFLNB	659.140432	-1.353647108	0.28648	-4.72511	2.30E-06	1.82E-05
RN7SKP1	4.11251082	2.099639344	0.444616	4.722367	2.33E-06	1.84E-05
LINC0239	19.9146497	-2.776639594	0.588121	-4.72121	2.34E-06	1.85E-05
YWHAZP	62.2034914	1.000754428	0.211989	4.720774	2.35E-06	1.85E-05
CD5	232.759139	-2.091876247	0.443257	-4.71933	2.37E-06	1.86E-05
HSPE1P18	19.1630074	-2.269449568	0.481039	-4.71781	2.38E-06	1.87E-05
PLVAP	2316.39123	-1.170942045	0.248258	-4.71663	2.40E-06	1.88E-05
PPFIA3	288.899653	1.118091696	0.237107	4.715563	2.41E-06	1.89E-05
ROR2	448.080669	-1.994702724	0.423148	-4.71396	2.43E-06	1.91E-05
RNU6-583	1.05954553	2.837123236	0.601954	4.713187	2.44E-06	1.91E-05
LINC0157	51.5041941	1.199894408	0.25459	4.713048	2.44E-06	1.91E-05
SLC25A5I	6.24853421	2.246071599	0.476719	4.711525	2.46E-06	1.92E-05
FBXO43	28.9055221	1.433741199	0.304363	4.710631	2.47E-06	1.93E-05
ADAMTS	34.5032545	-1.233121975	0.261898	-4.7084	2.50E-06	1.95E-05
LINC0131	54.6902567	1.126807795	0.23935	4.707788	2.50E-06	1.96E-05
ACBD7	25.6380303	1.692369677	0.35953	4.707173	2.51E-06	1.96E-05
NFIB	2747.45288	-1.337048034	0.284128	-4.70579	2.53E-06	1.97E-05
FAM216B	1.92804312	-2.740579233	0.582426	-4.70546	2.53E-06	1.98E-05
SYT7	597.947543	1.67961192	0.356978	4.705091	2.54E-06	1.98E-05
EEPD1	348.920577	-1.08969221	0.231611	-4.70484	2.54E-06	1.98E-05
MTFP1	20.5017798	1.084095887	0.230522	4.702795	2.57E-06	2.00E-05
P2RY13	137.442852	-1.56144308	0.332205	-4.70024	2.60E-06	2.02E-05
BTLA	45.6057512	-1.985542491	0.422472	-4.69982	2.60E-06	2.02E-05
METTL11	4.99194052	4.104716855	0.87377	4.697711	2.63E-06	2.04E-05
ENG	3093.01956	-1.214075872	0.258459	-4.69736	2.64E-06	2.04E-05
SFN	6348.80793	1.504156551	0.320256	4.696735	2.64E-06	2.05E-05
RGL4	33.317585	-1.691286684	0.360262	-4.6946	2.67E-06	2.07E-05

KRT78	14.5789974	2.262327898	0.482127	4.69239	2.70E-06	2.09E-05
RNF180	138.017215	-1.456884514	0.310632	-4.69007	2.73E-06	2.11E-05
SPATA17	76.1141394	1.490248945	0.31779	4.689421	2.74E-06	2.12E-05
MGAT4EI	3.38744216	2.26840253	0.483929	4.68747	2.77E-06	2.14E-05
DACT3-A	5.80872563	-2.441724436	0.520994	-4.68666	2.78E-06	2.14E-05
IRAK3	549.823882	-1.329615557	0.283715	-4.68644	2.78E-06	2.14E-05
PIK3R5	378.518102	-1.628786863	0.347586	-4.68599	2.79E-06	2.15E-05
KIFC2	2873.42975	1.012312117	0.216095	4.68458	2.81E-06	2.16E-05
FAM110B	155.909215	-1.740227759	0.371533	-4.68392	2.81E-06	2.17E-05
BHLHE22	91.2159389	-1.652065525	0.352712	-4.6839	2.81E-06	2.17E-05
RDH16	43.4320172	1.263876744	0.269932	4.682201	2.84E-06	2.18E-05
CXCR5	84.2012883	-2.801769247	0.598466	-4.68159	2.85E-06	2.19E-05
CNTNAP1	522.956539	-1.293355348	0.276353	-4.68009	2.87E-06	2.20E-05
LINC0035	15.3883721	3.554857721	0.759853	4.678351	2.89E-06	2.22E-05
ODAD1	35.7123663	1.580814928	0.338023	4.676644	2.92E-06	2.24E-05
SNORD11	0.94913696	-2.722557281	0.582595	-4.67315	2.97E-06	2.27E-05
BMP8B	253.209592	1.413008074	0.302509	4.670956	3.00E-06	2.30E-05
LINC0141	1.26646784	-2.582876502	0.553239	-4.66864	3.03E-06	2.32E-05
DPY19L2	194.82556	-1.603753827	0.343555	-4.66812	3.04E-06	2.33E-05
PRR7	131.850775	1.007286564	0.215813	4.667395	3.05E-06	2.33E-05
CYP4A22-	20.0491638	1.537135232	0.329367	4.666937	3.06E-06	2.34E-05
ZNF467	164.109549	-1.175258511	0.251866	-4.66621	3.07E-06	2.34E-05
PELI2	551.96739	-1.359286147	0.291474	-4.66349	3.11E-06	2.37E-05
GLDC	166.917597	2.228486088	0.477912	4.662962	3.12E-06	2.38E-05
PKP3	1917.55796	1.048610738	0.224895	4.662675	3.12E-06	2.38E-05
CGB5	4.15540882	2.633968091	0.565204	4.660211	3.16E-06	2.40E-05
CD160	13.4133986	-1.994055146	0.42792	-4.65988	3.16E-06	2.41E-05
MMP23A	8.46365989	-1.359965012	0.291849	-4.65982	3.16E-06	2.41E-05
TRAJ39	1.10197274	-2.550467682	0.547394	-4.65929	3.17E-06	2.41E-05
GPM6B	313.804303	-1.738751372	0.373221	-4.65877	3.18E-06	2.42E-05
CD28	166.217033	-1.773853772	0.380763	-4.65868	3.18E-06	2.42E-05
IGHV5-78	3.75193276	-3.321764182	0.713388	-4.65632	3.22E-06	2.44E-05
PCP2	74.4375548	1.406560004	0.302089	4.656111	3.22E-06	2.44E-05
ABCA12	131.38787	2.231640233	0.479555	4.653567	3.26E-06	2.47E-05
EPYC	10.5611669	3.904397863	0.83929	4.652024	3.29E-06	2.49E-05
IL33	1221.42054	-1.791285521	0.385057	-4.652	3.29E-06	2.49E-05
EPHA4	636.907337	1.141525751	0.24547	4.650368	3.31E-06	2.51E-05
TNFSF8	79.5072864	-1.805618397	0.388313	-4.6499	3.32E-06	2.51E-05
ADAM19	1481.42152	-1.961421106	0.421996	-4.64796	3.35E-06	2.54E-05
PDGFC	620.167233	-1.169234687	0.251611	-4.647	3.37E-06	2.54E-05
TCF4	2831.73857	-1.313184124	0.282592	-4.64692	3.37E-06	2.54E-05
SNORA70	4.4581361	-1.510566823	0.325093	-4.64657	3.38E-06	2.55E-05
HIGD1B	56.9337855	-1.211441861	0.260721	-4.64651	3.38E-06	2.55E-05
LINC0063	3.42726149	2.230112299	0.480106	4.645046	3.40E-06	2.56E-05
RANBP3-I	14.4823404	1.067393312	0.229845	4.64397	3.42E-06	2.58E-05
TNFRSF1.	57.608764	-1.801184828	0.388028	-4.6419	3.45E-06	2.60E-05
TAGAP	309.534933	-1.595340053	0.343728	-4.64129	3.46E-06	2.60E-05
GRHL3-A	10.8142303	1.732795714	0.37338	4.64084	3.47E-06	2.61E-05
RN7SL494	3.25739224	-2.305791193	0.496869	-4.64065	3.47E-06	2.61E-05
CACNA1(	3.12864519	-3.189592497	0.68764	-4.63846	3.51E-06	2.63E-05
NFATC4	1242.99461	-1.031265735	0.222355	-4.63793	3.52E-06	2.64E-05
ZNF812P	652.455923	2.250171629	0.485174	4.637867	3.52E-06	2.64E-05

KLRG1	57.1335159	-1.349772979	0.291054	-4.63753	3.53E-06	2.64E-05
KIAA0895	3.99103966	3.891608819	0.839243	4.637045	3.53E-06	2.65E-05
HTRA1	2168.5995	-1.287138681	0.277636	-4.63607	3.55E-06	2.66E-05
RNASE1	2869.28515	-1.364021604	0.294406	-4.63313	3.60E-06	2.69E-05
ASPHD1	93.0792784	1.712262446	0.369591	4.632864	3.61E-06	2.70E-05
ZNF736P9	29.1780881	3.05685561	0.659907	4.63225	3.62E-06	2.70E-05
MPP2	170.71477	-1.52237785	0.328841	-4.62952	3.67E-06	2.74E-05
EPHA6	18.7584305	-2.128501453	0.45983	-4.62888	3.68E-06	2.74E-05
SLAMF1	144.793176	-1.991222969	0.430219	-4.6284	3.69E-06	2.75E-05
TMPO-AS	79.8982357	1.00071568	0.216221	4.628199	3.69E-06	2.75E-05
CCL19	439.183758	-2.820523418	0.609428	-4.62815	3.69E-06	2.75E-05
MAP4K1	336.093046	-1.633796372	0.353057	-4.62757	3.70E-06	2.76E-05
CITED1	12.7283016	-1.544425799	0.33404	-4.62347	3.77E-06	2.81E-05
ITGB6	3346.67953	1.32025089	0.285587	4.622936	3.78E-06	2.81E-05
USP18	250.733218	1.325517475	0.286757	4.622436	3.79E-06	2.82E-05
SLCO6A1	16.0073565	3.01484918	0.652317	4.621758	3.81E-06	2.83E-05
CXCR2	283.089949	-1.417826369	0.306788	-4.62152	3.81E-06	2.83E-05
HLA-DPA	5455.23749	-1.516983925	0.328298	-4.62075	3.82E-06	2.84E-05
CPLX1	151.84104	-1.28015875	0.277177	-4.61856	3.86E-06	2.87E-05
EIF2S2P3	29.7993919	-1.194060744	0.258598	-4.61744	3.89E-06	2.88E-05
CABYR	150.892677	1.324018697	0.28686	4.615557	3.92E-06	2.90E-05
CLEC18C	7.37213767	2.229771218	0.483134	4.615224	3.93E-06	2.91E-05
MANEA-I	40.3265449	1.090779838	0.236445	4.613248	3.96E-06	2.93E-05
GRASLNI	30.1233503	2.125788995	0.460837	4.612889	3.97E-06	2.94E-05
PARD3B	438.40652	-1.353919963	0.293533	-4.6125	3.98E-06	2.94E-05
PLEKHA4	758.624733	-1.416196151	0.307035	-4.61249	3.98E-06	2.94E-05
SULT1C4	67.1315774	-1.525787453	0.330849	-4.61174	3.99E-06	2.95E-05
CD200	455.659813	-1.545707691	0.335177	-4.61161	4.00E-06	2.95E-05
MIR4731	0.80817569	-2.722418776	0.590399	-4.61115	4.00E-06	2.96E-05
HOXC13-/	42.5910951	2.1025205	0.455996	4.610831	4.01E-06	2.96E-05
CENPM	125.46967	1.342026917	0.291073	4.610624	4.01E-06	2.96E-05
ARHGAP4	1115.18207	-1.439023263	0.312192	-4.60941	4.04E-06	2.98E-05
MAGEA1	49.801096	2.950868293	0.640324	4.608396	4.06E-06	2.99E-05
TRAV13-1	13.1535771	-1.992347279	0.432337	-4.60832	4.06E-06	2.99E-05
KDF1	417.444479	1.02610629	0.222668	4.60824	4.06E-06	2.99E-05
TRHDE-A	519.137251	2.432140258	0.527835	4.607767	4.07E-06	3.00E-05
HGF	389.958683	-1.912382726	0.415147	-4.60652	4.09E-06	3.01E-05
S1PR4	132.736846	-1.567370896	0.340261	-4.60637	4.10E-06	3.01E-05
LAD1	4724.34064	1.183607085	0.256959	4.606211	4.10E-06	3.01E-05
SEZ6L2	840.204443	1.467939775	0.318701	4.606007	4.10E-06	3.02E-05
LIPK	14.3487576	3.511733131	0.76257	4.605129	4.12E-06	3.03E-05
ARHGEF1	1440.89307	-1.071059241	0.232626	-4.60422	4.14E-06	3.04E-05
CD200R1	89.1547173	-1.563879339	0.339734	-4.60325	4.16E-06	3.05E-05
SOD2	13548.6312	-1.239449851	0.269328	-4.60201	4.18E-06	3.07E-05
HSPE1P4	8.61250831	1.49310569	0.324491	4.601381	4.20E-06	3.08E-05
TAS2R5	31.3139026	1.250729114	0.271888	4.60016	4.22E-06	3.09E-05
LINC0141	3.50603155	4.206385334	0.914551	4.599397	4.24E-06	3.10E-05
HIC2	384.423079	1.133856172	0.246627	4.597462	4.28E-06	3.13E-05
VCX	61.1157477	3.473400469	0.75552	4.597365	4.28E-06	3.13E-05
FLT3LG	106.097929	-1.059497052	0.230522	-4.59607	4.31E-06	3.15E-05
TRAV20	4.96258844	-2.33406127	0.507944	-4.59512	4.33E-06	3.16E-05
RSPO4	48.0021448	2.819215396	0.613702	4.593788	4.35E-06	3.17E-05

KCNMB2-	76.2405428	1.858234622	0.404602	4.592746	4.37E-06	3.19E-05
FMNL1-D	293.205503	-1.518368145	0.330619	-4.5925	4.38E-06	3.19E-05
FAM153B	11.3999592	-1.91885375	0.417925	-4.59139	4.40E-06	3.21E-05
LINC0098	4.86677412	-1.887447585	0.411101	-4.5912	4.41E-06	3.21E-05
FZD5	484.082426	1.063455242	0.231659	4.590611	4.42E-06	3.22E-05
GEMIN8P	14.3963385	1.090671404	0.237606	4.590258	4.43E-06	3.22E-05
LINC0153	5.15263091	3.517066784	0.766242	4.59002	4.43E-06	3.22E-05
RN7SKP1	3.6440477	3.429376384	0.747175	4.589793	4.44E-06	3.22E-05
TIE1	635.324351	-1.454842687	0.316999	-4.58942	4.44E-06	3.23E-05
PKD1	2655.60941	-1.034915977	0.225518	-4.58906	4.45E-06	3.23E-05
ITK	287.606541	-1.840809225	0.401153	-4.58879	4.46E-06	3.24E-05
MRC2	3225.95027	-1.310702217	0.285707	-4.58757	4.48E-06	3.25E-05
SPEF1	9.92557262	1.964162182	0.428215	4.586855	4.50E-06	3.26E-05
MIR431	1.4089824	-3.218850205	0.702017	-4.58514	4.54E-06	3.29E-05
SLC10A4	10.0319884	1.792328828	0.391012	4.583823	4.57E-06	3.30E-05
LINC0184	4.95602595	2.498342725	0.545096	4.583308	4.58E-06	3.31E-05
PCMTD1F	2.03545265	2.504755343	0.546552	4.582828	4.59E-06	3.32E-05
PROS1	789.448498	-1.314326346	0.286827	-4.5823	4.60E-06	3.32E-05
KIF12	56.9748586	1.953312816	0.426379	4.581167	4.62E-06	3.34E-05
TRAV3	9.00697381	-2.271902367	0.49598	-4.58063	4.64E-06	3.34E-05
TAFA2	70.2416439	-1.156495177	0.252488	-4.5804	4.64E-06	3.34E-05
RPL38P4	1.37110917	-2.279627016	0.497714	-4.5802	4.65E-06	3.35E-05
LINC0269	2.75931317	-1.893236382	0.413509	-4.57846	4.68E-06	3.37E-05
CRYZL2P	10.7351665	1.500971124	0.327852	4.578202	4.69E-06	3.37E-05
CDCP1	1233.19779	1.023566992	0.223575	4.578179	4.69E-06	3.37E-05
SEPTIN6	1119.75051	-1.340249052	0.29276	-4.57798	4.69E-06	3.38E-05
SMPD4P1	34.7917184	3.806208686	0.831419	4.577965	4.70E-06	3.38E-05
SUMO2P3	6.98927568	1.220313635	0.266587	4.577549	4.70E-06	3.38E-05
MYO10	1516.70432	1.097865697	0.23985	4.577293	4.71E-06	3.38E-05
NCR3	15.2921293	-2.058412195	0.44983	-4.57598	4.74E-06	3.40E-05
NUDT8	153.403819	1.137544065	0.24867	4.574516	4.77E-06	3.43E-05
BTK	262.376064	-1.608610274	0.351693	-4.5739	4.79E-06	3.43E-05
AMOTL1	1471.57369	-1.21460628	0.265584	-4.57334	4.80E-06	3.44E-05
COX4I2	76.5184828	-1.273931722	0.278616	-4.57236	4.82E-06	3.46E-05
COL27A1	1930.35533	1.169597573	0.255883	4.570829	4.86E-06	3.48E-05
PEAK3	10.5406607	-1.754680852	0.383909	-4.57056	4.86E-06	3.48E-05
CCDC116	7.63367457	-1.033886866	0.226214	-4.57039	4.87E-06	3.49E-05
ZNF823	426.454049	1.097222279	0.24009	4.57004	4.88E-06	3.49E-05
IL34	121.696069	-1.403021459	0.307099	-4.56863	4.91E-06	3.51E-05
CSPG4	1207.37317	-1.666653965	0.364901	-4.56741	4.94E-06	3.53E-05
MIR30C2	2.25710209	-2.059905378	0.451047	-4.56694	4.95E-06	3.54E-05
PIF1	227.403428	1.051547215	0.230255	4.566889	4.95E-06	3.54E-05
SLC47A1I	3.35937237	-2.451390092	0.536817	-4.56652	4.96E-06	3.54E-05
ADGRB3	71.7926575	-2.142334215	0.469156	-4.56636	4.96E-06	3.54E-05
LINC0113	21.8354141	3.041268915	0.666153	4.565423	4.98E-06	3.56E-05
LINC0137	45.5190616	1.098163421	0.240546	4.565295	4.99E-06	3.56E-05
PPP1R14C	141.616648	2.342015675	0.513129	4.564185	5.01E-06	3.58E-05
IKZF2	1202.87104	1.214274967	0.26606	4.563906	5.02E-06	3.58E-05
SCARA3	781.591465	-1.51127327	0.331174	-4.56338	5.03E-06	3.59E-05
SPINK5	514.067126	1.874394551	0.410759	4.563241	5.04E-06	3.59E-05
PTHLH	1015.4755	2.370165155	0.519525	4.562175	5.06E-06	3.61E-05
NCKAP1L	836.759213	-1.691174476	0.370746	-4.56154	5.08E-06	3.62E-05

CGNL1	405.996262	-1.584603771	0.347441	-4.56078	5.10E-06	3.63E-05
TYMS	898.339773	1.199212571	0.262976	4.560161	5.11E-06	3.63E-05
SEPTIN14	2.54924972	2.788611923	0.611734	4.55854	5.15E-06	3.66E-05
VCX2	2.90935764	2.910916258	0.638576	4.558449	5.15E-06	3.66E-05
IRF8	586.528538	-1.687676687	0.370327	-4.55725	5.18E-06	3.68E-05
MYH13	5.83506242	-2.53274739	0.555799	-4.55694	5.19E-06	3.68E-05
LILRA2	49.7812326	-1.815984859	0.398623	-4.55564	5.22E-06	3.71E-05
STK32A	349.734745	1.809679626	0.397287	4.555096	5.24E-06	3.71E-05
KDM4A-A	15.3656399	1.314385848	0.288682	4.553052	5.29E-06	3.75E-05
LINC0243	44.6311055	2.627413743	0.577151	4.552383	5.30E-06	3.76E-05
BLID	1.18230122	-2.461209598	0.54065	-4.55232	5.31E-06	3.76E-05
RN7SL69I	1.87771227	-2.034934075	0.44724	-4.54999	5.36E-06	3.79E-05
SLC24A2	2.888398	2.466359076	0.542171	4.549041	5.39E-06	3.81E-05
LINC0255	10.7604626	-1.545236351	0.339742	-4.54827	5.41E-06	3.82E-05
ACVR2B	63.6005202	1.132787377	0.249109	4.547365	5.43E-06	3.83E-05
CMTM2	25.5882323	-1.548716013	0.340624	-4.5467	5.45E-06	3.84E-05
PRICKLE2	1.22256306	-2.70209676	0.594401	-4.54591	5.47E-06	3.86E-05
CELSR2	843.708621	1.047392416	0.230423	4.545522	5.48E-06	3.86E-05
RIBC2	56.5190046	1.554115787	0.342011	4.544057	5.52E-06	3.89E-05
S100A16	3157.89612	1.190082549	0.261902	4.544002	5.52E-06	3.89E-05
LINC0275	4.81959368	-1.801510315	0.396576	-4.54266	5.55E-06	3.91E-05
EHF	2632.12031	1.279961184	0.281785	4.542339	5.56E-06	3.91E-05
GLIPR2	711.880308	-1.506014859	0.331691	-4.54041	5.61E-06	3.95E-05
LHX6	105.957116	-1.233455417	0.271767	-4.53865	5.66E-06	3.98E-05
MIR23B	6.01737968	-2.011324444	0.443249	-4.53769	5.69E-06	3.99E-05
HSPE1P26	6.60998555	-1.101841957	0.242888	-4.53642	5.72E-06	4.01E-05
LHB	6.58934029	1.691670409	0.373001	4.535294	5.75E-06	4.03E-05
TIMP3	11799.5448	-1.289549348	0.284502	-4.53266	5.82E-06	4.08E-05
LINC0066	837.960839	1.065059682	0.235083	4.530562	5.88E-06	4.12E-05
DNAH14	312.824545	1.267650187	0.27984	4.52991	5.90E-06	4.13E-05
LINGO3	2.38670847	-2.068115175	0.456572	-4.52966	5.91E-06	4.13E-05
THBS4	125.586669	-2.247264064	0.496535	-4.52589	6.01E-06	4.20E-05
TRBV5-6	5.8441376	-2.111005197	0.466467	-4.52552	6.02E-06	4.21E-05
GIMAP7	563.36912	-1.561776216	0.345192	-4.52436	6.06E-06	4.23E-05
TMEFF2	17.1624923	-2.458317287	0.543437	-4.52365	6.08E-06	4.24E-05
OTX1	74.5011553	2.160116499	0.477553	4.523298	6.09E-06	4.25E-05
PHACTR1	133.256699	-1.468313249	0.324694	-4.52214	6.12E-06	4.27E-05
POPDC3	52.7230006	2.569429957	0.56822	4.521896	6.13E-06	4.27E-05
LYPLAL1	1.11860859	-3.200061103	0.707747	-4.52147	6.14E-06	4.28E-05
LILRA4	16.9114289	-2.175803855	0.481443	-4.51934	6.20E-06	4.32E-05
CHRM3-A	41.8553109	-1.895827956	0.419507	-4.51918	6.21E-06	4.32E-05
TMEM24(	38.9282907	-1.098586746	0.243152	-4.51811	6.24E-06	4.34E-05
FBXW11P	9.19861189	1.292624077	0.286199	4.516522	6.29E-06	4.37E-05
MIR924H(	19.0221108	2.148547683	0.475792	4.51573	6.31E-06	4.38E-05
TUB-AS1	1.02732322	-3.262828989	0.722563	-4.51563	6.31E-06	4.38E-05
TTC23L	64.4814013	1.532796277	0.339459	4.51541	6.32E-06	4.38E-05
PRSS16	335.783345	1.246995955	0.276179	4.515171	6.33E-06	4.39E-05
RN7SKP7:	16.0982112	-1.800646614	0.39888	-4.51425	6.35E-06	4.40E-05
TACC3	651.926294	1.021746981	0.226415	4.512713	6.40E-06	4.43E-05
RHOH	235.90606	-1.790468772	0.396945	-4.51062	6.46E-06	4.46E-05
RPS29P14	11.9468555	-1.068264416	0.236839	-4.5105	6.47E-06	4.46E-05
RPS6KA6	77.3201546	1.36918613	0.30356	4.51043	6.47E-06	4.46E-05

AKT3	747.421133	-1.388169477	0.308012	-4.50687	6.58E-06	4.53E-05
ACAP2-IT	10.3420361	1.318835491	0.292669	4.50623	6.60E-06	4.54E-05
GIMAP4	771.052482	-1.349556934	0.299488	-4.50621	6.60E-06	4.54E-05
ADAMTS	374.617823	-1.448681407	0.321563	-4.50512	6.63E-06	4.56E-05
TRAV35	3.27079577	-1.977960795	0.43941	-4.5014	6.75E-06	4.64E-05
RASD1	313.632789	-1.446758557	0.321407	-4.50133	6.75E-06	4.64E-05
RRM2	952.65326	1.451125227	0.322417	4.500766	6.77E-06	4.65E-05
C6orf52	17.3654698	1.256721733	0.279288	4.499729	6.80E-06	4.67E-05
PTPN5	27.7871563	-2.157515904	0.479486	-4.49965	6.81E-06	4.67E-05
PNPLA1	9.27262285	1.904037343	0.423202	4.499125	6.82E-06	4.68E-05
TTC28	857.180634	-1.037316622	0.23056	-4.49912	6.82E-06	4.68E-05
ZNF192P1	39.1934383	1.165761242	0.259155	4.498313	6.85E-06	4.69E-05
POU2AF1	590.632887	-1.899454241	0.422273	-4.49817	6.85E-06	4.69E-05
C22orf34	56.1075366	-1.326079194	0.29488	-4.49701	6.89E-06	4.72E-05
LINC0125	0.78795825	-2.476344667	0.550767	-4.49617	6.92E-06	4.73E-05
GRAP2	171.195761	-1.593753509	0.354491	-4.49589	6.93E-06	4.74E-05
CDH5	993.889607	-1.312252485	0.291892	-4.49567	6.94E-06	4.74E-05
7SK	3285.35459	-1.986933434	0.442117	-4.49413	6.99E-06	4.77E-05
CYP21A1]	15.2782921	-1.585577167	0.352985	-4.49191	7.06E-06	4.81E-05
IGF2-AS	20.0885504	2.446939014	0.544777	4.491635	7.07E-06	4.82E-05
PTPRG-A!	30.3730589	1.940544072	0.432171	4.490224	7.11E-06	4.85E-05
MIR766	1.49222401	-2.752166662	0.612974	-4.48986	7.13E-06	4.85E-05
NFYAP1	3.75524333	3.737664151	0.832946	4.487284	7.21E-06	4.91E-05
RPL21P44	51.0442525	-1.113033128	0.248134	-4.48562	7.27E-06	4.94E-05
KLF2-DT	1.41001857	-2.437081887	0.54334	-4.48537	7.28E-06	4.95E-05
CYP27B1	65.0528698	1.199465214	0.267424	4.485263	7.28E-06	4.95E-05
BMERB1	632.978194	-1.321491554	0.294727	-4.48378	7.33E-06	4.98E-05
RPL21P13	3.85124482	-1.877722882	0.418936	-4.48213	7.39E-06	5.01E-05
THOC1-D'	8.76426424	1.345631888	0.300263	4.481508	7.41E-06	5.02E-05
MIR2052F	11.3694238	2.765208968	0.617148	4.480625	7.44E-06	5.04E-05
B4GALNT	4.10278813	4.76091298	1.062559	4.480609	7.44E-06	5.04E-05
KRT18P1	2.39142214	3.467702859	0.774089	4.479724	7.47E-06	5.06E-05
UFL1-AS1	6.99416804	1.556086315	0.347401	4.479223	7.49E-06	5.07E-05
HOXC6	63.9450478	2.055637066	0.459084	4.477693	7.55E-06	5.10E-05
FXYD2	3.80634664	-1.915940447	0.427892	-4.47762	7.55E-06	5.10E-05
IGLV3-27	103.177116	-3.112403334	0.695225	-4.47683	7.58E-06	5.12E-05
ADAMTS!	743.072474	-1.563442668	0.349261	-4.47643	7.59E-06	5.13E-05
AGGF1P1	4.10540906	1.765404925	0.394517	4.474848	7.65E-06	5.16E-05
CD2AP-D'	51.6954789	1.237704471	0.276641	4.474045	7.68E-06	5.17E-05
MIR4538	5.12838536	-3.210420489	0.717844	-4.47231	7.74E-06	5.21E-05
RUNX1T1	267.195555	-1.867403299	0.417557	-4.47221	7.74E-06	5.21E-05
TMC8	869.021515	-1.538465871	0.344016	-4.47208	7.75E-06	5.21E-05
NNMT	1575.53997	-1.627640844	0.363962	-4.47201	7.75E-06	5.21E-05
AKR7A3	49.23237	1.059163122	0.236846	4.471953	7.75E-06	5.21E-05
WAS	438.359922	-1.542993702	0.345096	-4.47121	7.78E-06	5.23E-05
CSF2RA	369.099138	-1.47331197	0.329572	-4.47038	7.81E-06	5.25E-05
SERPING]	5182.31358	-1.511761953	0.338205	-4.46996	7.82E-06	5.25E-05
LRRC70	20.97318	-1.196213092	0.267636	-4.46956	7.84E-06	5.26E-05
CCR4	179.948585	-2.062499521	0.461555	-4.46859	7.87E-06	5.28E-05
C1QTNF7	4.11238428	-1.759132413	0.393786	-4.46723	7.92E-06	5.31E-05
SNX5P2	1.77912168	-2.358287024	0.528156	-4.46514	8.00E-06	5.36E-05
ZSCAN23	45.7034815	1.586799349	0.355389	4.464965	8.01E-06	5.36E-05

TLE6	100.498689	1.440807254	0.322724	4.464523	8.02E-06	5.37E-05
PHYHD1	179.797665	-1.350141227	0.302591	-4.46194	8.12E-06	5.43E-05
SNRPCP1	1.95235906	-1.869979809	0.419145	-4.46141	8.14E-06	5.44E-05
CCDC110	27.9527103	-1.749652141	0.392313	-4.45984	8.20E-06	5.47E-05
ZNF831	79.5197347	-1.998291105	0.448173	-4.45875	8.24E-06	5.50E-05
SLC8A1-A	23.3398807	2.437949894	0.546828	4.458351	8.26E-06	5.50E-05
GPR162	75.6191041	-1.301724543	0.292119	-4.45615	8.34E-06	5.55E-05
SNORD11	2.7575093	-1.996386094	0.448031	-4.45591	8.35E-06	5.56E-05
LINC0218	17.3895534	-1.479167428	0.331984	-4.45553	8.37E-06	5.57E-05
OSBPL10	1094.75815	-1.132240529	0.254168	-4.45469	8.40E-06	5.59E-05
IMPDH1P	8.0850697	1.344099767	0.301792	4.45373	8.44E-06	5.61E-05
MTSS1	1724.75729	1.032923134	0.232078	4.450749	8.56E-06	5.68E-05
SLC38A5	449.585772	1.450329809	0.325864	4.450723	8.56E-06	5.68E-05
MS4A4A	489.345847	-1.413631662	0.317702	-4.44956	8.60E-06	5.71E-05
IL17B	9.62303115	-1.646098959	0.369979	-4.44916	8.62E-06	5.72E-05
CD6	362.196116	-1.838997824	0.413409	-4.44837	8.65E-06	5.74E-05
VNN3	33.7260775	-1.734295901	0.389946	-4.44753	8.69E-06	5.76E-05
LINC0191	6.88004321	-1.903872382	0.428114	-4.44712	8.70E-06	5.77E-05
SELPLG	530.809619	-1.552046257	0.349113	-4.44569	8.76E-06	5.80E-05
CA3	66.2665242	-1.614130974	0.363087	-4.44558	8.77E-06	5.80E-05
PRPH	25.0607925	-2.226689475	0.501072	-4.44386	8.84E-06	5.84E-05
HMGN1P4	6.66365741	1.212155449	0.272811	4.443205	8.86E-06	5.85E-05
MROH6	499.565895	1.180134604	0.265617	4.442986	8.87E-06	5.86E-05
TFAP2A	949.714877	1.20512959	0.27131	4.441894	8.92E-06	5.88E-05
IL10RA	999.20893	-1.626503544	0.36633	-4.43999	9.00E-06	5.93E-05
GRM8	19.3634216	-1.381506497	0.311197	-4.43933	9.02E-06	5.95E-05
SLC1A2	33.911699	-1.297616343	0.292459	-4.43692	9.13E-06	6.01E-05
LINC0086	457.548786	-1.301781781	0.293441	-4.43626	9.15E-06	6.02E-05
NWD1	46.7616544	-2.070447439	0.466956	-4.43392	9.25E-06	6.09E-05
PCDHGB7	92.2191203	-1.513864102	0.341441	-4.43375	9.26E-06	6.09E-05
PCDHB15	75.5270832	-1.116978752	0.251997	-4.43251	9.31E-06	6.12E-05
GJA4	267.744628	-1.394590473	0.314815	-4.42987	9.43E-06	6.19E-05
CORO1A	1881.76262	-1.470541079	0.331995	-4.42941	9.45E-06	6.20E-05
HOXC8	35.6276773	2.126534024	0.480454	4.426094	9.60E-06	6.29E-05
COX4I1P2	1.27799731	2.727855702	0.616385	4.425574	9.62E-06	6.30E-05
EVI2B	537.584431	-1.609164497	0.363607	-4.42556	9.62E-06	6.30E-05
KCNH2	258.770462	-2.149872049	0.485804	-4.42539	9.63E-06	6.30E-05
ABRA	2.03942855	-2.38854432	0.53982	-4.42471	9.66E-06	6.32E-05
CEACAM	27.5260613	-1.725915406	0.390114	-4.42413	9.68E-06	6.34E-05
LARP4P	25.6755964	-1.169140313	0.26427	-4.42404	9.69E-06	6.34E-05
SNORD11	2.98866586	-2.295396851	0.519074	-4.4221	9.77E-06	6.39E-05
LINC0180	14.2798563	3.594494698	0.81286	4.422035	9.78E-06	6.39E-05
DDN	13.5957586	2.013595526	0.45552	4.42043	9.85E-06	6.44E-05
NT5C1A	0.79398044	-2.447989738	0.553828	-4.42013	9.86E-06	6.45E-05
PTCH1	727.705056	-1.073539832	0.242945	-4.41886	9.92E-06	6.48E-05
HSPA2	663.262608	-1.252675099	0.283485	-4.41884	9.92E-06	6.48E-05
NPTX1	76.2638164	-1.971201397	0.446428	-4.4155	1.01E-05	6.57E-05
NRIP3	153.644782	1.187590027	0.269055	4.413931	1.02E-05	6.61E-05
NINL	511.306135	1.056074917	0.239341	4.412433	1.02E-05	6.65E-05
HOXC-AS	5.91971451	2.491958286	0.564859	4.411645	1.03E-05	6.67E-05
WDR87BP	7.73082043	3.213206532	0.728643	4.409851	1.03E-05	6.73E-05
TESPA1	149.616654	-1.650884078	0.374879	-4.40378	1.06E-05	6.91E-05

FAM83C	190.233639	1.937125355	0.439889	4.403665	1.06E-05	6.91E-05
SCARNA5	19.9126686	-2.200629051	0.499908	-4.40207	1.07E-05	6.96E-05
EVX2	6.10420726	-2.906997176	0.660486	-4.4013	1.08E-05	6.98E-05
PCDHGC3	444.899155	-1.332122064	0.302766	-4.39984	1.08E-05	7.02E-05
NCF4	519.488408	-1.154305971	0.262382	-4.39934	1.09E-05	7.04E-05
IL6R	689.146006	-1.260757531	0.286706	-4.39739	1.10E-05	7.10E-05
TULP2	4.82031638	-1.776896467	0.404388	-4.39403	1.11E-05	7.20E-05
TMEM20(	281.773158	-1.438924498	0.327584	-4.39253	1.12E-05	7.24E-05
SHISA8	5.07456702	-2.320339776	0.52831	-4.392	1.12E-05	7.26E-05
RNU6-313	9.25236274	-1.606690143	0.365868	-4.39145	1.13E-05	7.27E-05
VN1R42P	4.22685369	1.165117353	0.26534	4.391039	1.13E-05	7.28E-05
TBXA2R	52.1502282	-1.550163071	0.353039	-4.39092	1.13E-05	7.28E-05
MAB21L2	2.88729244	-3.67306831	0.83665	-4.39021	1.13E-05	7.30E-05
MEOX2	96.2340391	-2.442419165	0.556338	-4.39017	1.13E-05	7.30E-05
PROC	39.8699308	1.482759445	0.337808	4.389355	1.14E-05	7.33E-05
TUBB6	2113.00416	-1.522112124	0.34681	-4.3889	1.14E-05	7.34E-05
LINC0228	2.9755281	-1.929621305	0.439704	-4.38845	1.14E-05	7.35E-05
ADAMTS	283.763344	-1.182303714	0.269415	-4.38841	1.14E-05	7.35E-05
HPDL	43.7569761	1.866840984	0.425408	4.388352	1.14E-05	7.35E-05
HASPIN	42.194511	1.33702082	0.304728	4.387584	1.15E-05	7.38E-05
ATP8B4	200.925743	-1.319753226	0.300825	-4.38712	1.15E-05	7.39E-05
MS4A6A	1312.33564	-1.311555338	0.298989	-4.38664	1.15E-05	7.40E-05
MNDA	556.276583	-1.497398499	0.341455	-4.38535	1.16E-05	7.44E-05
MYO7B	21.1303876	-1.471110948	0.335816	-4.3807	1.18E-05	7.59E-05
SLC12A8	221.284463	1.212543702	0.276793	4.38068	1.18E-05	7.59E-05
CPNE4	31.3583882	2.127035531	0.485639	4.379869	1.19E-05	7.61E-05
DPP4	189.010544	-1.72820421	0.394738	-4.3781	1.20E-05	7.67E-05
TFDP1P2	9.02372476	1.844183688	0.421248	4.3779	1.20E-05	7.68E-05
COX20P1	20.9744591	1.212958616	0.277099	4.377354	1.20E-05	7.69E-05
PDZPH1P	0.69094898	-2.397935676	0.547865	-4.37688	1.20E-05	7.71E-05
ADAMTS	910.937276	-1.619415856	0.370063	-4.37605	1.21E-05	7.73E-05
HMGA2	170.287664	3.031974596	0.692872	4.375955	1.21E-05	7.73E-05
NBPF4	45.1273564	2.497443257	0.571018	4.373671	1.22E-05	7.81E-05
C9orf163	12.2789814	1.18531088	0.271097	4.372274	1.23E-05	7.85E-05
LINC0216	14.6281747	3.55908043	0.814184	4.371349	1.23E-05	7.88E-05
LINC0161	22.5607954	3.234517948	0.739954	4.37124	1.24E-05	7.88E-05
WWTR1-I	41.2486344	-1.342348178	0.307264	-4.36872	1.25E-05	7.97E-05
DIRC3	30.5758649	-1.717223228	0.393113	-4.36827	1.25E-05	7.98E-05
CBS	758.591759	1.54781745	0.354348	4.36807	1.25E-05	7.99E-05
KRT5	22037.956	2.502171885	0.572946	4.367202	1.26E-05	8.01E-05
GP5	5.1491419	-1.525858509	0.349401	-4.36707	1.26E-05	8.02E-05
SMPDL3A	640.632463	-1.044172298	0.239123	-4.36668	1.26E-05	8.03E-05
LBH	488.644547	-1.235667139	0.282986	-4.36652	1.26E-05	8.03E-05
CD200R1I	3.86364826	2.758089702	0.63196	4.364342	1.28E-05	8.10E-05
LRFN2	26.0980081	2.403802715	0.550791	4.364272	1.28E-05	8.10E-05
SCIMP	166.550826	-1.721434396	0.39457	-4.36281	1.28E-05	8.14E-05
PLA2G12	5.12211376	1.136135664	0.260422	4.362677	1.28E-05	8.15E-05
RNFT2	103.048697	1.188939419	0.272537	4.362482	1.29E-05	8.15E-05
ENO2	1499.48423	1.167083585	0.267568	4.361822	1.29E-05	8.17E-05
IKZF1	566.605757	-1.689792906	0.387543	-4.36028	1.30E-05	8.22E-05
PTCHD3P	3.12175791	2.441086645	0.559873	4.360075	1.30E-05	8.22E-05
PRAM1	104.426963	-1.432675057	0.32876	-4.35782	1.31E-05	8.30E-05

PLEK2	589.674191	1.129348989	0.259216	4.356791	1.32E-05	8.34E-05
FMO2	59.2194195	-2.295052008	0.527176	-4.35348	1.34E-05	8.45E-05
LINC0220	6.99840781	-1.82745422	0.419792	-4.35324	1.34E-05	8.46E-05
RNU6-401	1.19946041	-3.350694091	0.769767	-4.35287	1.34E-05	8.47E-05
SERPINE2	1624.37339	-1.693670319	0.389095	-4.35285	1.34E-05	8.47E-05
VPS37D	91.6386435	1.268248926	0.291411	4.352092	1.35E-05	8.50E-05
EMX1	11.5521004	3.050688725	0.701238	4.350434	1.36E-05	8.55E-05
NAP1L4P2	1.60192887	3.052300304	0.701625	4.350328	1.36E-05	8.55E-05
FLJ31356	58.5510878	1.334995799	0.306932	4.349486	1.36E-05	8.58E-05
INHBB	362.408834	-1.222456309	0.281124	-4.34846	1.37E-05	8.61E-05
CYP1B1	717.086839	-1.686931467	0.388053	-4.34717	1.38E-05	8.65E-05
LINC0119	13.5318176	-2.74441355	0.631404	-4.34652	1.38E-05	8.67E-05
FFAR1	1.38270913	-2.863908315	0.658985	-4.34594	1.39E-05	8.69E-05
SALL4	175.227514	1.590599588	0.366115	4.34454	1.40E-05	8.74E-05
SNORA80	10.7707681	-1.873202004	0.43123	-4.34386	1.40E-05	8.76E-05
PPIAP16	6.48223924	-1.461260957	0.336421	-4.34355	1.40E-05	8.77E-05
LPAR1	908.922495	-1.137388133	0.261968	-4.34171	1.41E-05	8.84E-05
DRAXIN	22.4383072	1.51793563	0.34962	4.341668	1.41E-05	8.84E-05
CACHD1	410.886104	-1.083938818	0.24973	-4.34045	1.42E-05	8.88E-05
CD33	116.924279	-1.408948241	0.324719	-4.33898	1.43E-05	8.93E-05
KIF19	63.2548885	-1.94821779	0.449019	-4.33883	1.43E-05	8.94E-05
APLN	113.682672	1.514461098	0.349077	4.338478	1.43E-05	8.95E-05
SH2B3	1034.83925	-1.194886247	0.275523	-4.33679	1.45E-05	9.01E-05
SGCG	38.2337914	-2.750178656	0.634172	-4.33665	1.45E-05	9.02E-05
TRGV4	4.46381641	-2.140046231	0.493595	-4.33563	1.45E-05	9.06E-05
ZNF366	60.2153706	-1.141841481	0.263411	-4.33482	1.46E-05	9.08E-05
HBG2	4.74525885	-1.369698516	0.315976	-4.33482	1.46E-05	9.08E-05
MIR127	1.02375373	-2.841852762	0.655741	-4.3338	1.47E-05	9.12E-05
STC1	1419.70008	-1.254522337	0.289514	-4.3332	1.47E-05	9.14E-05
LST1	337.991892	-1.380191023	0.31855	-4.33273	1.47E-05	9.15E-05
NOS1	11.9100619	-1.897574679	0.437983	-4.33253	1.47E-05	9.16E-05
MYOSLID	9.08893543	2.380192536	0.549465	4.331839	1.48E-05	9.18E-05
BTBD6P1	4.15865194	-2.707104472	0.625049	-4.33103	1.48E-05	9.21E-05
SPAG17	301.584363	1.359718307	0.314088	4.329101	1.50E-05	9.28E-05
FYN	1326.7588	-1.318904016	0.304675	-4.32889	1.50E-05	9.28E-05
ALOX12	118.054869	2.124093886	0.490809	4.327741	1.51E-05	9.33E-05
KRT8P15	15.1427274	-1.333838644	0.308223	-4.32751	1.51E-05	9.34E-05
VXN	28.2272945	-1.101769282	0.254637	-4.32683	1.51E-05	9.36E-05
BCL6B	372.759339	-1.334295582	0.308527	-4.32473	1.53E-05	9.44E-05
CHAC2	84.9298445	1.003122573	0.231952	4.324692	1.53E-05	9.44E-05
FSIP2-AS1	30.5125178	1.387026851	0.320736	4.324511	1.53E-05	9.44E-05
LIF	1711.30534	-1.820596522	0.421081	-4.32362	1.53E-05	9.48E-05
AATK	119.5691	-1.157113438	0.267665	-4.32299	1.54E-05	9.50E-05
TRBV4-1	7.14277975	-1.746072662	0.403999	-4.32197	1.55E-05	9.54E-05
CKS1BP2	5.13546388	1.661396471	0.384428	4.321738	1.55E-05	9.54E-05
DSEL	248.520311	-1.273132185	0.294612	-4.32138	1.55E-05	9.56E-05
TUB	285.747621	-1.440060487	0.333296	-4.32067	1.56E-05	9.59E-05
LAMA4	2399.6538	-1.235994714	0.286235	-4.31811	1.57E-05	9.69E-05
CARMIL3	73.6857167	1.280521641	0.296553	4.318024	1.57E-05	9.69E-05
PLAGL1	973.248563	-1.234581776	0.285975	-4.3171	1.58E-05	9.73E-05
PHOSPHC	23.8833383	-1.909707671	0.442439	-4.31632	1.59E-05	9.76E-05
LUM	13674.9174	-1.559117142	0.361243	-4.31598	1.59E-05	9.77E-05

CXCL2	510.850153	-1.910673298	0.442708	-4.31588	1.59E-05	9.77E-05
IGLC7	166.485477	-2.769973976	0.642249	-4.31293	1.61E-05	9.88E-05
SCG5	135.917063	1.750553914	0.405901	4.312757	1.61E-05	9.89E-05
H2BC5	494.367456	1.336930176	0.310078	4.311591	1.62E-05	9.94E-05
NLRP3	144.951305	-1.453285302	0.337216	-4.30966	1.64E-05	0.0001
SCUBE3	192.329256	-1.747539094	0.405504	-4.30955	1.64E-05	0.0001
IL36G	25.5665499	2.60476701	0.604645	4.307925	1.65E-05	0.000101
IATPR	1.92403459	-2.764331949	0.641715	-4.30772	1.65E-05	0.000101
GPRC5B	709.930643	-1.043377614	0.242233	-4.30733	1.65E-05	0.000101
PRSS23	3471.22974	-1.044208154	0.242593	-4.30436	1.67E-05	0.000102
RNU6-549	8.01474163	-1.273555156	0.295955	-4.3032	1.68E-05	0.000103
RN7SKP4(	1.80682234	-2.174196257	0.505307	-4.30272	1.69E-05	0.000103
EXTL1	12.8043604	-1.717648958	0.39921	-4.30262	1.69E-05	0.000103
WIPF3	131.307614	-1.674506818	0.389298	-4.30135	1.70E-05	0.000104
COMTD1	511.543555	1.005681981	0.23381	4.301279	1.70E-05	0.000104
MIR4322	1.16571023	-2.24079673	0.520992	-4.30102	1.70E-05	0.000104
TRPM3	5.11907013	-2.367399292	0.550473	-4.30066	1.70E-05	0.000104
MIR337	1.16794381	-3.070547908	0.714164	-4.2995	1.71E-05	0.000104
MIR4768	22.342782	-1.275186421	0.296634	-4.29886	1.72E-05	0.000105
DPYSL3	5499.44915	-1.802276494	0.419396	-4.29731	1.73E-05	0.000105
LRRC34	77.8569935	1.182518057	0.275225	4.296552	1.73E-05	0.000106
CH25H	151.55994	-1.670392684	0.388983	-4.29426	1.75E-05	0.000107
FSD1	69.5842303	1.962768523	0.457145	4.293532	1.76E-05	0.000107
CSPG4P11	31.0570657	-1.684997396	0.392656	-4.29128	1.78E-05	0.000108
RNU6-942	26.5270521	-1.196996874	0.278986	-4.29053	1.78E-05	0.000108
OLFM4	6904.09176	-3.192652259	0.744331	-4.28929	1.79E-05	0.000109
RPL10P19	11.1819193	-1.349225712	0.314575	-4.28904	1.79E-05	0.000109
KIAA2012	1.80781893	-1.685720757	0.393107	-4.2882	1.80E-05	0.000109
RNU5E-8I	1.03676987	-3.368753098	0.785623	-4.288	1.80E-05	0.000109
CST6	140.944328	1.736048685	0.404875	4.287858	1.80E-05	0.000109
HAR1A	8.32044926	-1.566914624	0.365624	-4.28559	1.82E-05	0.000111
AREG	121.396469	-1.675620797	0.390993	-4.28555	1.82E-05	0.000111
SNRPFP1	5.79301252	1.277608588	0.298233	4.283931	1.84E-05	0.000111
HVCN1	236.617257	-1.075988606	0.251368	-4.28054	1.86E-05	0.000113
GPR155	580.448106	-1.341201193	0.31345	-4.27884	1.88E-05	0.000114
RPL29P19	34.704502	1.901146665	0.444424	4.277775	1.89E-05	0.000114
MRAS	566.340891	-1.231226301	0.28788	-4.27688	1.90E-05	0.000114
SNORA14	4.80778957	1.438762703	0.336536	4.275207	1.91E-05	0.000115
RNU6-125	22.077334	-1.028060733	0.240503	-4.27463	1.91E-05	0.000115
STPG1	492.025771	1.189759747	0.278362	4.274142	1.92E-05	0.000116
BCO2	127.171338	-1.068797507	0.250064	-4.2741	1.92E-05	0.000116
NPM1P49	2.33033702	-1.509872291	0.353308	-4.27353	1.92E-05	0.000116
RPL11P3	73.2665989	-1.022993181	0.23938	-4.27351	1.92E-05	0.000116
MRPL37P	36.5549094	-1.387098719	0.324808	-4.27052	1.95E-05	0.000117
LINC0272	1.82569001	-2.081297115	0.48757	-4.26872	1.97E-05	0.000118
CBARP-D	2.02756846	2.708068454	0.634601	4.267353	1.98E-05	0.000119
RN7SKP9.	5.29344345	1.589662785	0.372544	4.267048	1.98E-05	0.000119
DNAH17	19.9628365	2.270823609	0.532217	4.266725	1.98E-05	0.000119
LINC0178	8.98582462	-2.493237718	0.584423	-4.26616	1.99E-05	0.000119
FAM83A	2225.41492	2.19729715	0.515111	4.265674	1.99E-05	0.000119
FABP5P1(	2.00996171	2.287244857	0.536745	4.261327	2.03E-05	0.000122
CSTL1	5.35101827	2.256198365	0.529463	4.261293	2.03E-05	0.000122

PRRT2	281.96579	-1.557642471	0.365549	-4.2611	2.03E-05	0.000122
FLNC-AS1	1.65932916	-3.769285113	0.884621	-4.2609	2.04E-05	0.000122
RPS29P20	3.79802696	-1.552473215	0.364355	-4.26089	2.04E-05	0.000122
LINC0136	2.14200633	-1.676323974	0.393479	-4.26026	2.04E-05	0.000122
HSD17B1	135.983561	1.176679407	0.276353	4.257891	2.06E-05	0.000123
LDLRAD2	31.383946	-1.280823587	0.300884	-4.25687	2.07E-05	0.000124
LRRC18	5.0764689	-1.683383696	0.395629	-4.25496	2.09E-05	0.000124
GNG7	313.537093	-1.668721213	0.39248	-4.25173	2.12E-05	0.000126
CHRM3	256.815568	-2.174086841	0.511413	-4.25114	2.13E-05	0.000126
VWC2	6.48105892	-2.369743264	0.557509	-4.25059	2.13E-05	0.000127
ASGR2	28.0027868	-1.676482136	0.394557	-4.24903	2.15E-05	0.000127
LINC0210	4.13368573	-2.073395038	0.488035	-4.24846	2.15E-05	0.000128
CPO	6.88355111	-1.021474288	0.240488	-4.24751	2.16E-05	0.000128
ABI3	253.220993	-1.230923904	0.2898	-4.24749	2.16E-05	0.000128
ACTN3	3.40302139	-1.420815411	0.334513	-4.24741	2.16E-05	0.000128
XCL1	77.2783894	1.840694942	0.433391	4.247188	2.16E-05	0.000128
FMNL1	1339.86102	-1.410905245	0.332238	-4.24667	2.17E-05	0.000128
MADCAM	31.6338465	-1.618076234	0.381075	-4.24609	2.18E-05	0.000129
PTPRCAP	497.238839	-1.325041009	0.312114	-4.24537	2.18E-05	0.000129
E2F8	302.731635	1.278507569	0.30121	4.244573	2.19E-05	0.00013
RNU6-806	3.78514069	-2.77685648	0.654262	-4.24426	2.19E-05	0.00013
ARHGAP3	664.198701	-1.261250023	0.297168	-4.24423	2.19E-05	0.00013
LINC0165	1.37747812	3.041664185	0.716728	4.243818	2.20E-05	0.00013
KCNJ8	186.328073	-1.374579912	0.323939	-4.24333	2.20E-05	0.00013
PSMD12P	5.44871165	1.341569183	0.316211	4.242642	2.21E-05	0.00013
GFPT2	524.225643	-2.086568949	0.491898	-4.24187	2.22E-05	0.000131
CSTB	12282.9975	1.125893573	0.265465	4.241206	2.22E-05	0.000131
GAPDHP6	2.44869491	1.99978748	0.471515	4.241193	2.22E-05	0.000131
IGKV1-27	447.674644	-2.956056661	0.697078	-4.24064	2.23E-05	0.000131
ABCD2	45.531685	-1.76341524	0.415903	-4.23997	2.24E-05	0.000132
GJB7	86.6641848	1.876559944	0.442629	4.239574	2.24E-05	0.000132
PRSS21	94.4059557	2.423683949	0.571794	4.238738	2.25E-05	0.000132
CRNDE	343.905287	1.32297488	0.312139	4.238418	2.25E-05	0.000132
MYCN	102.590324	2.190617622	0.51692	4.237825	2.26E-05	0.000133
SLITRK6	2817.65917	1.714613274	0.404634	4.237445	2.26E-05	0.000133
NCF1B	93.984106	-1.456996179	0.343918	-4.23646	2.27E-05	0.000133
RPL10P1	1.66977186	-2.218239383	0.523697	-4.23573	2.28E-05	0.000134
AIF1L	419.049197	-1.279472602	0.302067	-4.23572	2.28E-05	0.000134
THPO	6.12050724	-1.515177843	0.357789	-4.23484	2.29E-05	0.000134
USH2A	3.98221673	2.232474515	0.527213	4.234485	2.29E-05	0.000134
MAPRE2	962.259767	-1.12537854	0.265774	-4.23435	2.29E-05	0.000134
PTGES3P1	24.6698824	1.074462033	0.253769	4.234016	2.30E-05	0.000134
TMEM176	1879.15526	-1.409818588	0.333031	-4.23329	2.30E-05	0.000135
TNPO1P1	9.86224595	-1.08588819	0.256546	-4.23272	2.31E-05	0.000135
BDNF	169.405701	-1.947978089	0.460695	-4.22834	2.35E-05	0.000138
MATN1	5.52414881	-1.093039347	0.258587	-4.22697	2.37E-05	0.000138
HBEGF	1397.57159	-1.322665745	0.312928	-4.22674	2.37E-05	0.000138
ANG	82.2945392	-1.02813318	0.243285	-4.22604	2.38E-05	0.000139
NFE2L3	735.597243	1.102784424	0.261017	4.224958	2.39E-05	0.000139
RASGRF2	237.040546	-1.139210636	0.269701	-4.22398	2.40E-05	0.00014
GGT5	548.23219	-1.372722137	0.324996	-4.22382	2.40E-05	0.00014
LINC0054	0.84838982	-2.348174927	0.556042	-4.22302	2.41E-05	0.00014

CD24P4	2054.35785	1.40979178	0.333849	4.222839	2.41E-05	0.00014
KCNA3	33.4892126	-1.722161805	0.407875	-4.22228	2.42E-05	0.000141
PLAC1	69.9684435	2.594180564	0.614608	4.220868	2.43E-05	0.000142
MT1XP1	4.27206028	-1.162674954	0.275485	-4.22047	2.44E-05	0.000142
DIRAS2	97.6629002	2.742897754	0.649967	4.220056	2.44E-05	0.000142
H2BC11	77.2254481	1.738226035	0.412058	4.218404	2.46E-05	0.000143
ADH7	176.558631	3.431937557	0.813757	4.217399	2.47E-05	0.000144
SACS-AS1	1.28261673	-3.553174616	0.84263	-4.21677	2.48E-05	0.000144
MAP1LC3	6.48037159	-1.985865877	0.47111	-4.21529	2.49E-05	0.000145
SLC22A1(	1.21479313	-2.477262437	0.58769	-4.21526	2.49E-05	0.000145
IL26	7.99993806	-2.913168719	0.691478	-4.21296	2.52E-05	0.000146
NES	1561.28069	-1.429146445	0.339296	-4.21209	2.53E-05	0.000146
MANEAL	302.578261	1.337932224	0.317717	4.211084	2.54E-05	0.000147
TRBV2	12.1694139	-2.055155019	0.488051	-4.21095	2.54E-05	0.000147
SNX29P1	17.6050754	-1.260729645	0.299593	-4.20814	2.57E-05	0.000149
PHF21B	7.04823979	-1.780276659	0.423133	-4.20736	2.58E-05	0.000149
CDC7	469.963226	1.043156476	0.247963	4.206897	2.59E-05	0.000149
ST6GALN	140.368478	-1.80808859	0.429934	-4.2055	2.61E-05	0.00015
MID1IP1-/	2.16938626	1.723763523	0.409883	4.205501	2.61E-05	0.00015
LURAP1	85.413689	-1.028739168	0.244674	-4.20453	2.62E-05	0.000151
HOXB4	333.911371	1.003453217	0.238709	4.203663	2.63E-05	0.000151
VSIG4	586.6416	-1.434348454	0.341277	-4.20289	2.64E-05	0.000152
SRMS	90.406619	1.550417911	0.368935	4.20241	2.64E-05	0.000152
RPSAP52	6.73226509	3.977019083	0.946509	4.201776	2.65E-05	0.000152
RASSF2	1195.43255	-1.161194702	0.276533	-4.19911	2.68E-05	0.000154
SORD	1877.15245	1.11176094	0.264815	4.19826	2.69E-05	0.000154
H3P47	52.175992	1.209648347	0.288144	4.198062	2.69E-05	0.000154
PLIN4	1008.21813	-1.412237678	0.336427	-4.19775	2.70E-05	0.000155
LGI2	126.745158	-1.431538236	0.341186	-4.19577	2.72E-05	0.000156
H3C4	20.3682521	1.602403659	0.382027	4.194476	2.74E-05	0.000157
BNC1	115.714871	3.223976684	0.76885	4.193245	2.75E-05	0.000157
ZNF114	35.5791445	1.613510453	0.385091	4.189951	2.79E-05	0.00016
BARX1-D	1.97503827	3.452327468	0.824084	4.18929	2.80E-05	0.00016
SLC16A2	343.389903	-1.265094759	0.302056	-4.18828	2.81E-05	0.000161
FRZB	369.70808	-1.076015333	0.256915	-4.18822	2.81E-05	0.000161
TRAT1	66.6220724	-1.982556388	0.473383	-4.18806	2.81E-05	0.000161
SPNS3	31.5167376	-1.387466011	0.331296	-4.188	2.81E-05	0.000161
RUNX3	565.108084	-1.611812378	0.384894	-4.18768	2.82E-05	0.000161
P2RY10	86.9747574	-1.890562444	0.451487	-4.18742	2.82E-05	0.000161
LYPD1	80.3646081	1.976539496	0.47213	4.186426	2.83E-05	0.000162
ABCC2	48.4533331	-1.090682217	0.26058	-4.1856	2.84E-05	0.000162
S100A3	249.746001	1.754824044	0.419313	4.184994	2.85E-05	0.000162
HEG1	1811.88885	-1.199718491	0.286739	-4.18402	2.86E-05	0.000163
ELMO1	476.408522	-1.388168082	0.331785	-4.18394	2.86E-05	0.000163
DGCR5	10.4073714	1.804953073	0.431501	4.182963	2.88E-05	0.000164
H2BC13	8.43670011	1.980533223	0.473601	4.181859	2.89E-05	0.000164
RPL21P12	15.6753277	-1.195743639	0.28597	-4.18135	2.90E-05	0.000165
NMRK2	1.45644556	-2.657297564	0.635724	-4.17995	2.92E-05	0.000166
EMID1	262.480575	-1.282519602	0.306841	-4.17975	2.92E-05	0.000166
TTLL7	629.852449	-1.455245574	0.348227	-4.17902	2.93E-05	0.000166
CCDC102	297.897705	-1.021379494	0.244468	-4.17797	2.94E-05	0.000167
EVA1A	58.8393473	1.809471357	0.433264	4.176369	2.96E-05	0.000168

TSLP	143.223093	-1.370811218	0.328235	-4.1763	2.96E-05	0.000168
COX6CP1	6.50499864	-1.639206031	0.392743	-4.17374	3.00E-05	0.00017
GPC6	425.414231	-1.850981757	0.443634	-4.17232	3.02E-05	0.00017
COPZ2	267.547333	-1.342966314	0.321907	-4.1719	3.02E-05	0.000171
GNA14	292.597956	-1.496734928	0.358791	-4.17161	3.02E-05	0.000171
RASL11B	128.007445	1.702681267	0.408172	4.171485	3.03E-05	0.000171
GRAP	156.226397	-1.18963639	0.285188	-4.17141	3.03E-05	0.000171
TCF7	468.45846	-1.186352059	0.284412	-4.17125	3.03E-05	0.000171
TH	98.297455	2.340300113	0.561184	4.170294	3.04E-05	0.000172
LINC0271	4.1641397	-1.636123473	0.392408	-4.16945	3.05E-05	0.000172
FAM131C	16.5903045	1.799781661	0.431744	4.168635	3.06E-05	0.000173
SPON2	1635.83525	-1.41462522	0.339361	-4.1685	3.07E-05	0.000173
TRPV2	317.852449	-1.296634632	0.311086	-4.16809	3.07E-05	0.000173
GLIS2	498.496399	-1.297567428	0.311386	-4.16708	3.09E-05	0.000174
TMEM229	3.95466552	-2.402486321	0.576859	-4.16477	3.12E-05	0.000175
DNM3OS	90.3032469	-1.699802386	0.40817	-4.16445	3.12E-05	0.000176
ACAP1	615.113127	-1.59530364	0.383097	-4.16423	3.12E-05	0.000176
MIR9-3HC	33.613836	1.60719127	0.386169	4.161883	3.16E-05	0.000177
GPR20	12.8554837	-1.697330146	0.408105	-4.15905	3.20E-05	0.000179
FSIP2	226.486944	1.764456344	0.424253	4.15897	3.20E-05	0.00018
FAM78A	250.809467	-1.443108828	0.347022	-4.15855	3.20E-05	0.00018
ZEB2-AS1	15.5840182	-1.492286616	0.35891	-4.15783	3.21E-05	0.00018
SAMD11	341.504493	-1.36829963	0.329127	-4.15736	3.22E-05	0.000181
LILRA1	49.1533737	-1.796397399	0.432153	-4.15686	3.23E-05	0.000181
KLHL38	2.86481842	-2.121095589	0.51035	-4.15616	3.24E-05	0.000181
NAP1L2	107.41501	-1.400175442	0.336893	-4.15615	3.24E-05	0.000181
TAS2R6P	6.38781227	1.434904257	0.345272	4.155863	3.24E-05	0.000181
NALT1	88.9502493	-1.265941137	0.304636	-4.15559	3.24E-05	0.000182
MIR3155A	6.56426513	-1.832811846	0.441116	-4.15495	3.25E-05	0.000182
RN7SL138	23.1884193	-1.326511509	0.319304	-4.15439	3.26E-05	0.000182
APBB1IP	494.691819	-1.501173175	0.361402	-4.15374	3.27E-05	0.000183
BTG1P1	3.11910842	-1.839106456	0.442779	-4.15355	3.27E-05	0.000183
CAMK2A	42.5078296	-2.277315383	0.548292	-4.15347	3.27E-05	0.000183
LINC0089	12.7038001	-1.685244454	0.405802	-4.15287	3.28E-05	0.000183
KLF7-IT1	19.5121871	-1.089002283	0.262306	-4.15164	3.30E-05	0.000184
FHAD1-A	9.90821341	-1.904010333	0.45864	-4.15143	3.30E-05	0.000184
TRBV3-1	10.2021935	-1.827962395	0.44066	-4.14824	3.35E-05	0.000187
RNU6-102	2.73595263	-1.435571382	0.346078	-4.14811	3.35E-05	0.000187
AADAC	111.950338	2.129789033	0.513492	4.147655	3.36E-05	0.000187
XXYLT1-	7.29800518	-1.805133151	0.43536	-4.1463	3.38E-05	0.000188
LINC0234	8.39168815	-1.972604417	0.475976	-4.14433	3.41E-05	0.00019
REV3L-IT	6.04988344	-1.688733205	0.407486	-4.14427	3.41E-05	0.00019
ITGAL	902.50873	-1.727595818	0.41691	-4.14381	3.42E-05	0.00019
HGH1	26.1686428	1.034374295	0.249628	4.143658	3.42E-05	0.00019
SNAP23P	1.81216786	2.289418806	0.552779	4.141653	3.45E-05	0.000192
LINC0242	10.9732085	-2.072159335	0.500397	-4.14103	3.46E-05	0.000192
RFTN1	893.19726	-1.097120802	0.265038	-4.13948	3.48E-05	0.000193
RASIP1	406.695157	-1.160946065	0.280565	-4.13789	3.51E-05	0.000194
MAGED4	158.97541	1.679651912	0.40604	4.136666	3.52E-05	0.000195
BATF3	81.6365118	-1.284215913	0.310527	-4.13561	3.54E-05	0.000196
FOXD2-A	52.9269174	1.299267157	0.314427	4.132177	3.59E-05	0.000199
ZNF45-AS	8.85394307	1.704542584	0.412514	4.132081	3.59E-05	0.000199

HOXB3	1467.92265	1.243921821	0.301147	4.130618	3.62E-05	0.0002
ZNF710-A	75.9387594	-1.255964117	0.30411	-4.12996	3.63E-05	0.0002
MT-TF	4.99943213	-1.062100213	0.25718	-4.1298	3.63E-05	0.0002
DNAJC22	48.8560596	1.964969495	0.475805	4.129783	3.63E-05	0.0002
COX6B2	62.1779646	1.674494642	0.405487	4.129593	3.63E-05	0.000201
LINC0017	26.894051	-1.70427724	0.412712	-4.12946	3.64E-05	0.000201
HSF5	4.98684372	-1.56683199	0.379462	-4.12909	3.64E-05	0.000201
SCNN1B	1463.38875	1.783103498	0.432104	4.126564	3.68E-05	0.000203
AGTR1	139.502896	-2.154747808	0.52226	-4.12581	3.69E-05	0.000204
TNFRSF11	1442.72839	-1.368696663	0.331806	-4.12499	3.71E-05	0.000204
TRAV16	4.34460341	-1.92471952	0.4666	-4.12499	3.71E-05	0.000204
ZAP70	422.850646	-1.613688743	0.391256	-4.12438	3.72E-05	0.000205
PPP1R26P	2.16830301	-1.348133763	0.327063	-4.12194	3.76E-05	0.000207
RNU6-603	1.68835338	-2.107425964	0.511324	-4.12151	3.76E-05	0.000207
VN1R108I	9.07108022	1.197925274	0.290658	4.121428	3.77E-05	0.000207
SLC2A13	248.834793	-1.066589199	0.258812	-4.1211	3.77E-05	0.000207
BCKDHA	8.97908098	1.10247113	0.267588	4.120037	3.79E-05	0.000208
IHO1	27.5162109	-1.447633837	0.351456	-4.11896	3.81E-05	0.000209
SLC2A3P2	3.99274239	-1.967667575	0.477816	-4.11805	3.82E-05	0.00021
IL36RN	37.9811672	2.742291685	0.666176	4.116465	3.85E-05	0.000211
IGKV2OR	1.3780232	3.064418551	0.744725	4.114834	3.87E-05	0.000212
MIR342	2.76457036	-1.746104019	0.424713	-4.11126	3.94E-05	0.000215
MIR581	13.2757383	-1.145124518	0.27861	-4.11013	3.95E-05	0.000216
TBC1D10	386.690882	-1.65726581	0.403215	-4.11013	3.95E-05	0.000216
FAM20C	878.329488	-1.575623508	0.383434	-4.10924	3.97E-05	0.000217
PRG4	25.4575027	-1.509000727	0.367307	-4.10828	3.99E-05	0.000218
UBASH3A	80.7834661	-1.733212265	0.421992	-4.10722	4.00E-05	0.000218
TRBC1	2.11693744	-2.290915096	0.558292	-4.10343	4.07E-05	0.000222
TNFSF14	142.527305	-1.763196782	0.429761	-4.10274	4.08E-05	0.000222
RNU6-132	4.24414399	-1.252450903	0.305306	-4.10228	4.09E-05	0.000223
PIK3CD	648.503527	-1.298458787	0.316608	-4.10115	4.11E-05	0.000224
LINC0032	6.29000058	-1.32291701	0.322576	-4.1011	4.11E-05	0.000224
HMGB1P2	14.0544812	-1.20194905	0.293082	-4.10107	4.11E-05	0.000224
TMEM71	136.5231	-1.429969934	0.348725	-4.10057	4.12E-05	0.000224
NCF1	201.264286	-1.385851219	0.33813	-4.09857	4.16E-05	0.000226
LRRTM1	13.9470891	-2.511888868	0.613043	-4.09741	4.18E-05	0.000227
TMEM150	99.7020831	-1.273393643	0.310846	-4.09654	4.19E-05	0.000228
MMRN2	1878.38237	-1.136872785	0.277626	-4.09497	4.22E-05	0.000229
C4orf54	26.4063064	-3.49133208	0.852645	-4.09471	4.23E-05	0.000229
COBLP1	2.00107477	3.237273133	0.791084	4.092201	4.27E-05	0.000232
CCDC136	246.115274	-1.515423023	0.370356	-4.09181	4.28E-05	0.000232
GPR137C	86.576448	1.391461182	0.340179	4.090374	4.31E-05	0.000233
GLS2	130.119615	1.293719387	0.316375	4.089195	4.33E-05	0.000234
MIR221	14.2968117	-1.31785647	0.322385	-4.08784	4.35E-05	0.000235
PTGER3	156.131566	-1.996217475	0.488555	-4.08596	4.39E-05	0.000237
RIN3	693.436991	-1.053995268	0.257967	-4.08578	4.39E-05	0.000237
ECM2	354.114407	-1.388559882	0.339954	-4.08455	4.42E-05	0.000238
MAFG-DT	67.6450321	1.111662639	0.272383	4.081249	4.48E-05	0.000241
TRBV29-1	19.8049926	-1.954337361	0.47887	-4.08114	4.48E-05	0.000241
IQANK1	1059.71026	1.14042166	0.279477	4.080554	4.49E-05	0.000242
TMEM171	33.8867709	1.533811702	0.375887	4.080517	4.49E-05	0.000242
ATP8A1	345.855478	-1.081779415	0.26511	-4.08049	4.49E-05	0.000242

PTCH2	99.5640984	-1.204174185	0.295155	-4.0798	4.51E-05	0.000242
TMEM121	40.3026824	-1.072620853	0.26291	-4.0798	4.51E-05	0.000242
CTSK	2623.99881	-1.330000121	0.32602	-4.0795	4.51E-05	0.000242
LINC0254	29.9456461	1.385739963	0.339727	4.078983	4.52E-05	0.000243
PLCB4	561.043882	-1.361420932	0.333933	-4.07693	4.56E-05	0.000245
ZHX1-C8c	5.5753263	1.12280074	0.275447	4.07628	4.58E-05	0.000245
CD180	135.589826	-1.507461828	0.369836	-4.07603	4.58E-05	0.000246
DPP4-DT	3.03516283	-2.255306049	0.553371	-4.07557	4.59E-05	0.000246
NECTIN4-	3.98239066	1.690982355	0.414993	4.074726	4.61E-05	0.000247
PRPH2	39.4302765	-1.171784121	0.287611	-4.0742	4.62E-05	0.000247
GVINP1	159.478548	-1.370507106	0.336425	-4.07373	4.63E-05	0.000248
C12orf56	60.7481561	2.046024689	0.502283	4.073453	4.63E-05	0.000248
HTR3E	2.00888391	3.197968544	0.785085	4.073404	4.63E-05	0.000248
LINC0288	485.012162	2.012638868	0.494106	4.073295	4.64E-05	0.000248
SLC1A7	78.9685791	2.545851637	0.625025	4.073202	4.64E-05	0.000248
PPM1F-AS	50.1760108	-1.161575265	0.285191	-4.07298	4.64E-05	0.000248
LINC0155	37.4658498	-1.476080122	0.362576	-4.07109	4.68E-05	0.00025
NME8	18.1706099	-1.545553567	0.379675	-4.07073	4.69E-05	0.00025
NCAM1	935.090182	-2.223586442	0.546472	-4.06898	4.72E-05	0.000252
NFE2	34.4466779	-1.661924103	0.408464	-4.06871	4.73E-05	0.000252
RN7SL589	10.0221717	-1.380669625	0.339365	-4.0684	4.73E-05	0.000253
LMNB1	954.055295	1.024743268	0.251978	4.066794	4.77E-05	0.000254
NKPD1	30.5764519	1.480865116	0.364304	4.064917	4.80E-05	0.000256
SLC2A1	10994.0753	1.471113539	0.361943	4.064486	4.81E-05	0.000257
HOXB2	537.692092	1.246459719	0.306749	4.06345	4.84E-05	0.000258
GSDMA	66.2770114	1.574282675	0.387439	4.06331	4.84E-05	0.000258
GLI2	157.667213	-1.789398848	0.440434	-4.0628	4.85E-05	0.000258
TRAV14D	8.16824477	-1.888898046	0.464976	-4.06236	4.86E-05	0.000259
KNOP1P4	1.60966801	-2.540535	0.62556	-4.06121	4.88E-05	0.00026
GSTM3	4584.18266	1.827284513	0.449991	4.060712	4.89E-05	0.00026
MIR645	2.5426108	-1.384972727	0.341139	-4.05985	4.91E-05	0.000261
ADCYAP1	220.175852	-2.297258534	0.566055	-4.05837	4.94E-05	0.000263
MIR1296	1.79910478	-1.732273221	0.42689	-4.05789	4.95E-05	0.000263
SERPINB1	180.327879	-2.607529022	0.643014	-4.05517	5.01E-05	0.000266
TMEM151	13.3618648	3.049013582	0.752087	4.054072	5.03E-05	0.000267
SNORD12	2.1023724	1.849842703	0.456334	4.053702	5.04E-05	0.000267
LINC0089	6.76291268	1.657021783	0.408833	4.05305	5.06E-05	0.000268
PPM1K-D	1.53021282	-1.581264519	0.39028	-4.05162	5.09E-05	0.000269
SPAG4	478.472174	1.064654863	0.262783	4.051459	5.09E-05	0.000269
MTND4P3	2.00822199	1.81260425	0.447416	4.051274	5.09E-05	0.00027
PRDM16-I	29.6108499	-1.945194769	0.480178	-4.05099	5.10E-05	0.00027
BTBD19	573.848757	-1.15170317	0.284305	-4.05094	5.10E-05	0.00027
CPXM1	926.670887	-1.805128345	0.445697	-4.05012	5.12E-05	0.000271
MAPK11	387.859595	-1.012242847	0.250009	-4.04883	5.15E-05	0.000272
EEF1E1P1	6.64745595	1.554249255	0.38396	4.047944	5.17E-05	0.000273
WIPF1	2018.0267	-1.264743894	0.312661	-4.0451	5.23E-05	0.000276
DSG2	4696.36198	1.015784034	0.251163	4.044318	5.25E-05	0.000277
ADORA1	35.2248597	-1.363442095	0.337158	-4.04393	5.26E-05	0.000277
RNA5SP5	1.92678064	-1.826326353	0.451712	-4.04312	5.27E-05	0.000278
ITGA2	2177.92815	1.115087701	0.275807	4.043006	5.28E-05	0.000278
MYH16	5.72194767	1.985786401	0.491267	4.042176	5.30E-05	0.000279
CFTR	57.1580447	-2.689339237	0.66555	-4.04078	5.33E-05	0.00028

SHROOM	628.855531	-1.218129624	0.301529	-4.03984	5.35E-05	0.000281
RUNDC3/	36.3969221	1.29443417	0.32046	4.039299	5.36E-05	0.000282
CSF1R	1677.02477	-1.412067901	0.349695	-4.038	5.39E-05	0.000283
CCT4P2	49.5530421	-1.404445387	0.348105	-4.03455	5.47E-05	0.000287
P4HA3	214.167051	-1.768999038	0.438514	-4.03407	5.48E-05	0.000287
RSU1P3	1.43943689	-2.51726911	0.624003	-4.03407	5.48E-05	0.000287
RN7SL47	1.1921744	3.148741331	0.780802	4.032702	5.51E-05	0.000289
ISLR	1041.33432	-1.813383214	0.449834	-4.03123	5.55E-05	0.00029
ATP2B4	4009.17104	-1.070080959	0.265575	-4.0293	5.59E-05	0.000293
TMEM20	468.202191	-1.062551396	0.263829	-4.02742	5.64E-05	0.000295
TXLNB	54.0276461	-1.329420819	0.330094	-4.0274	5.64E-05	0.000295
FCGR3B	298.493685	-1.641702946	0.407759	-4.02616	5.67E-05	0.000296
MTNR1A	3.00738717	-2.904402421	0.721529	-4.02535	5.69E-05	0.000297
IGHV3-72	97.1671624	-2.375128482	0.590181	-4.02441	5.71E-05	0.000298
IGHA1	19448.8105	-2.206596801	0.548316	-4.02431	5.71E-05	0.000298
LINC0033	4.7588738	1.588398509	0.394759	4.023722	5.73E-05	0.000299
ALDOC	588.167274	1.042020292	0.258976	4.02362	5.73E-05	0.000299
EVI2A	188.187783	-1.383017284	0.343756	-4.02325	5.74E-05	0.000299
LINC0215	2.93336728	2.269632079	0.564217	4.022624	5.76E-05	0.0003
C3orf86	5.96686686	-2.112715008	0.525438	-4.02086	5.80E-05	0.000302
AIM2	510.852032	1.903527733	0.473572	4.019507	5.83E-05	0.000304
DSC3	1664.09112	2.175548102	0.541306	4.019071	5.84E-05	0.000304
EPHX3	340.651944	1.507277419	0.375054	4.018831	5.85E-05	0.000304
ICAM2	508.041611	-1.046101597	0.260307	-4.01872	5.85E-05	0.000304
STX11	152.041179	-1.179992602	0.293626	-4.01869	5.85E-05	0.000304
MAGOH2	13.5457089	-1.258612758	0.313251	-4.01791	5.87E-05	0.000305
LGSN	38.4726913	3.344342387	0.832442	4.017506	5.88E-05	0.000306
SHISA6	14.6045331	-2.103411465	0.524104	-4.01335	5.99E-05	0.000311
BAALC	289.763387	1.866041999	0.465062	4.012459	6.01E-05	0.000312
PADI4	36.1924917	-1.659903219	0.413836	-4.01101	6.05E-05	0.000314
LINC0183	119.077882	2.480198887	0.618377	4.010819	6.05E-05	0.000314
MYBPC1	386.414065	2.502363398	0.623933	4.010628	6.06E-05	0.000314
MUC12	33.0155972	-1.288882444	0.321443	-4.00967	6.08E-05	0.000315
CERS3-AS	3.81488365	-1.966632338	0.490527	-4.00922	6.09E-05	0.000316
FABP5P2	11.909638	1.714688313	0.427722	4.008882	6.10E-05	0.000316
DOCK11	477.879214	-1.140720063	0.284586	-4.00834	6.11E-05	0.000317
PRTN3	0.62986197	-2.170883896	0.541925	-4.00587	6.18E-05	0.00032
FAM24B	5.62058427	1.634526009	0.408078	4.005423	6.19E-05	0.00032
RPSAP70	8.03467071	-1.399198825	0.349423	-4.00431	6.22E-05	0.000322
AGGF1P2	5.76076776	1.551230879	0.387467	4.003522	6.24E-05	0.000323
IGFBP4	13242.0523	-1.001200569	0.2501	-4.0032	6.25E-05	0.000323
RPS3AP2	4.03487978	2.953709016	0.738088	4.001837	6.29E-05	0.000325
LINC0024	18.075842	-1.570358701	0.39244	-4.00153	6.29E-05	0.000325
GTF2A1L	0.95997815	-2.713312604	0.678092	-4.00139	6.30E-05	0.000325
SNORD88	3.1861376	1.263176196	0.315732	4.000784	6.31E-05	0.000326
KCNK1	666.050323	1.090785471	0.272712	3.999775	6.34E-05	0.000327
BFSP2	3.41247671	-2.052561427	0.513205	-3.9995	6.35E-05	0.000327
FAM240C	2.0817408	-1.789331537	0.447565	-3.99793	6.39E-05	0.000329
NGFR	256.913309	-1.63889026	0.409994	-3.99735	6.41E-05	0.00033
FGF13	113.519133	-1.358318414	0.339824	-3.99712	6.41E-05	0.00033
SRPK2P	4.65612867	1.262618664	0.316148	3.993758	6.50E-05	0.000335
DMRT1	2.72160353	3.593110698	0.899928	3.992667	6.53E-05	0.000336

ISL2	43.0887023	1.639665335	0.41073	3.992081	6.55E-05	0.000337
POU2F2	661.011911	-1.293299205	0.32397	-3.99203	6.55E-05	0.000337
TEX46	3.36491168	1.548740308	0.387989	3.991708	6.56E-05	0.000337
GNG2	571.598831	-1.343146768	0.336539	-3.99105	6.58E-05	0.000338
SETP12	6.79355944	-1.575352845	0.394756	-3.9907	6.59E-05	0.000338
TTC39C-A	5.99464091	-1.615103769	0.404751	-3.99036	6.60E-05	0.000339
RGS6	74.2148991	1.437056907	0.360151	3.990153	6.60E-05	0.000339
RPL21P7	9.01502573	-1.751593329	0.439079	-3.98925	6.63E-05	0.00034
KLHL35	40.3335409	1.133075172	0.284104	3.988244	6.66E-05	0.000341
BVES	231.630624	-1.756781752	0.440596	-3.98728	6.68E-05	0.000342
AIF1	746.50355	-1.212503952	0.304147	-3.98657	6.70E-05	0.000343
LINC0239	2.41481171	2.407244168	0.604047	3.985193	6.74E-05	0.000345
PKIB	236.929783	1.529331175	0.383774	3.984974	6.75E-05	0.000345
H4C11	11.074661	1.613464266	0.404976	3.984095	6.77E-05	0.000347
SNORA71	16.7946821	1.004214038	0.252268	3.980749	6.87E-05	0.000351
HEMGN	3.18262686	-1.885100641	0.473659	-3.97987	6.90E-05	0.000352
KIF4CP	2.13450936	-2.346508505	0.589626	-3.97965	6.90E-05	0.000352
FTOP1	2.20669173	-1.637255316	0.411577	-3.97801	6.95E-05	0.000354
TCF7L1	375.546594	-1.202893468	0.302389	-3.97796	6.95E-05	0.000354
GAPDHP4	5.14768605	1.358388951	0.341512	3.977574	6.96E-05	0.000355
ARMC12	14.1392353	-1.282887259	0.322588	-3.97686	6.98E-05	0.000356
ACKR4	80.2828125	-1.531215456	0.385073	-3.97643	7.00E-05	0.000356
CCDC26	1.32834316	-1.900729722	0.478159	-3.9751	7.04E-05	0.000358
ZNF285	109.387469	1.073679066	0.270344	3.97153	7.14E-05	0.000363
TRBV5-1	19.3533162	-1.908038788	0.480536	-3.97065	7.17E-05	0.000364
LINC0048	3.61989086	-1.226940638	0.30904	-3.97017	7.18E-05	0.000365
HTRA3	1078.81785	-1.720394494	0.433433	-3.96923	7.21E-05	0.000366
FCRL2	65.7429018	-2.453588804	0.618255	-3.96857	7.23E-05	0.000367
LOXL3	203.975186	-1.040353048	0.26215	-3.96855	7.23E-05	0.000367
THORLN(	12.0246766	1.020323548	0.257129	3.96814	7.24E-05	0.000368
RNU6-742	1.23505926	-1.981198087	0.499442	-3.96683	7.28E-05	0.000369
CDH20	5.68509869	-1.799553829	0.453744	-3.96601	7.31E-05	0.00037
HILPDA	1309.62257	1.55837919	0.392954	3.965808	7.31E-05	0.00037
SMPDL3E	169.564007	1.257770729	0.31736	3.963227	7.39E-05	0.000374
RNU6-132	10.2818784	-1.40284265	0.354057	-3.96219	7.43E-05	0.000376
C1GALT1	0.86936673	-2.285540081	0.576921	-3.96162	7.44E-05	0.000377
NUGGC	63.1408857	-1.720504867	0.434298	-3.96158	7.45E-05	0.000377
RGS18	99.3749199	-1.361134528	0.343675	-3.96053	7.48E-05	0.000378
VSNL1	137.25223	2.023684516	0.511028	3.960028	7.49E-05	0.000379
GXYLT2	144.445043	-1.628632027	0.411764	-3.95525	7.65E-05	0.000385
TIFAB	27.2937907	-1.846679881	0.46695	-3.95477	7.66E-05	0.000386
PPATP1	3.35499139	1.210678263	0.306193	3.953977	7.69E-05	0.000387
ELOVL2	47.179668	-1.510054391	0.381988	-3.95315	7.71E-05	0.000388
TRAF3IP3	472.644976	-1.548917657	0.392033	-3.95098	7.78E-05	0.000391
RNU6-5P	3.16003707	-1.449320359	0.366855	-3.95067	7.79E-05	0.000392
CXCR4	1715.47777	-1.32151781	0.33459	-3.94967	7.83E-05	0.000393
TARDBPF	3.70392307	2.3692749	0.600036	3.948557	7.86E-05	0.000395
SVOPL	36.6377409	1.420698412	0.359922	3.947243	7.91E-05	0.000397
TUBB3	46.4479602	1.342838764	0.34029	3.946158	7.94E-05	0.000398
LINGO4	4.71734676	-1.472519012	0.373354	-3.94403	8.01E-05	0.000402
LINC0181	23.9585753	1.607007674	0.407671	3.941927	8.08E-05	0.000405
SLC9A7P1	24.2122957	-1.233523223	0.313002	-3.94095	8.12E-05	0.000406

TDRD5	136.677344	1.979940026	0.502596	3.939429	8.17E-05	0.000408
SLC26A1(	133.478517	-1.374026615	0.348794	-3.93936	8.17E-05	0.000408
TRAJ42	1.27291293	-2.497716339	0.634082	-3.9391	8.18E-05	0.000408
CFL1P5	4.89052652	1.143936692	0.290689	3.935256	8.31E-05	0.000414
SLC12A3	11.1172981	-1.610566946	0.409374	-3.93422	8.35E-05	0.000416
ITIH3	28.7587882	-1.622369915	0.412385	-3.93412	8.35E-05	0.000416
SCML4	62.1266968	-1.747190243	0.444165	-3.93366	8.37E-05	0.000417
LINC0144	1.25415937	-2.827035058	0.71873	-3.93338	8.38E-05	0.000417
DIO2	388.005518	1.700194681	0.432386	3.932126	8.42E-05	0.000419
LINC0150	4.98754696	-1.843354304	0.468941	-3.93088	8.46E-05	0.000421
BFSP2-AS	2.05624948	-1.878554613	0.477936	-3.93055	8.48E-05	0.000421
CNN3-DT	122.642605	-1.057254921	0.269018	-3.93005	8.49E-05	0.000422
CFAP74	10.8196499	1.664580989	0.423571	3.929874	8.50E-05	0.000422
TRAV2	5.43785302	-1.958455091	0.498362	-3.92979	8.50E-05	0.000422
DPF3	25.5122449	-1.23484043	0.31442	-3.92736	8.59E-05	0.000426
RN7SL11I	3.41422686	-1.495847492	0.381103	-3.92505	8.67E-05	0.00043
CASP4LP	116.487616	-1.167728031	0.297515	-3.92494	8.68E-05	0.00043
SMIM10	274.221363	-1.003007433	0.25559	-3.92429	8.70E-05	0.000431
SNAI3	52.2604047	-1.185360405	0.302073	-3.92408	8.71E-05	0.000431
CST7	236.928866	-1.595450282	0.406635	-3.92354	8.73E-05	0.000432
RN7SL521	6.96261031	1.038509451	0.264927	3.919978	8.86E-05	0.000438
VAV1	422.630269	-1.174102631	0.299598	-3.91893	8.89E-05	0.00044
FLJ12825	2.05551594	2.805831181	0.716145	3.917966	8.93E-05	0.000441
RN7SKP1'	6.64617421	1.218763339	0.311139	3.917103	8.96E-05	0.000443
GIPC3	101.540736	-1.172428754	0.299402	-3.91591	9.01E-05	0.000445
CR1	179.358704	-1.74195462	0.444858	-3.91575	9.01E-05	0.000445
ZNF492	25.6058133	2.054812362	0.524904	3.914643	9.05E-05	0.000447
RPL15P5	51.3367459	1.618239659	0.413444	3.914051	9.08E-05	0.000448
GNAZ	137.296543	-1.717679051	0.438996	-3.91274	9.13E-05	0.00045
H2BW1	4.90574687	3.118426503	0.797363	3.910925	9.19E-05	0.000453
ADAMTS:	38.0295003	-1.248565305	0.319289	-3.91045	9.21E-05	0.000454
CYP4F23I	346.293331	1.678072972	0.42934	3.908497	9.29E-05	0.000457
SIX3-AS1	3.93583625	3.697902568	0.946429	3.907214	9.34E-05	0.000459
BIRC3	1923.69185	-1.268097405	0.32461	-3.90653	9.36E-05	0.00046
LINC0282	18.5298311	2.050772545	0.525073	3.90569	9.40E-05	0.000462
EDNRA	528.950583	-1.322900675	0.338792	-3.90475	9.43E-05	0.000463
TREH	10.9525183	-1.504133158	0.385359	-3.9032	9.49E-05	0.000465
RPL17P50	48.7563471	1.028766994	0.263585	3.902974	9.50E-05	0.000465
NR2F1-AS	159.122067	-1.175666664	0.30128	-3.90224	9.53E-05	0.000467
DISP3	16.9892715	1.839843834	0.471637	3.900972	9.58E-05	0.000469
HAS3	4548.76711	1.402081515	0.35959	3.899112	9.65E-05	0.000472
ZNF540	91.3435725	-1.02815231	0.26369	-3.89909	9.66E-05	0.000472
FAM87B	2.86213068	-1.536902634	0.394385	-3.89696	9.74E-05	0.000476
FAM149A	252.387066	-1.357410425	0.348344	-3.89675	9.75E-05	0.000476
TRAV38-1	2.24104639	-2.230347495	0.572567	-3.89534	9.81E-05	0.000478
ZNF826P	72.7886375	1.214629936	0.311916	3.894097	9.86E-05	0.000481
TFF2	45.4794686	2.187061141	0.561744	3.893344	9.89E-05	0.000482
MDFI	1056.44555	1.168671055	0.300248	3.892348	9.93E-05	0.000484
LINC0098	84.3272741	-1.014325764	0.260618	-3.89201	9.94E-05	0.000484
POU5F1P(	5.2336438	-1.389584449	0.3572	-3.89021	0.0001	0.000487
ANKRD5(	2257.88207	1.004456175	0.258206	3.890135	0.0001	0.000487
LAGE3P1	3.06931201	1.340794809	0.344854	3.888007	0.000101	0.000491

COPDA1	11.6460161	-2.714845771	0.698419	-3.88713	0.000101	0.000493
LINC0096	123.564194	1.303557395	0.335635	3.88385	0.000103	0.000499
TBCAP1	23.9101888	1.021313387	0.262992	3.883445	0.000103	0.0005
KIAA0895	146.671261	1.11699379	0.287652	3.883149	0.000103	0.0005
TRAV36D	3.50686685	-2.018787912	0.519922	-3.88287	0.000103	0.000501
ZNF300	326.985527	1.288470052	0.331998	3.880953	0.000104	0.000504
SPOCK2	1050.13062	-1.43081811	0.368694	-3.88077	0.000104	0.000504
GLRA3	12.352343	1.969502643	0.50754	3.880485	0.000104	0.000505
LAYN	251.859278	-1.061067053	0.273543	-3.87898	0.000105	0.000508
C11orf86	2.51776137	3.023601749	0.77959	3.878453	0.000105	0.000509
YWHAZP	7.40645542	1.04762908	0.270147	3.878003	0.000105	0.000509
MFAP2	624.435838	1.226663839	0.316519	3.875482	0.000106	0.000514
SLC38A4	394.018336	1.89118594	0.488065	3.874864	0.000107	0.000515
NPAS4	21.6554814	-2.244625141	0.579287	-3.87481	0.000107	0.000515
IFI27	6149.04381	1.570335043	0.405277	3.874719	0.000107	0.000515
FAM96AP	7.85881972	2.019948205	0.521376	3.874265	0.000107	0.000516
PLEKHG4	185.159128	2.145766566	0.55386	3.874205	0.000107	0.000516
LYPLA1P	16.8992434	1.037660578	0.267844	3.874117	0.000107	0.000516
PRR7-AS1	11.1762245	1.227008763	0.316802	3.873104	0.000107	0.000518
LINC0123	46.486613	-1.614833746	0.416936	-3.8731	0.000107	0.000518
THEMIS2	855.934647	-1.201103251	0.310187	-3.87219	0.000108	0.00052
MACORIS	5.85891169	-1.544637647	0.399017	-3.87111	0.000108	0.000522
HNF4A	15.9943832	2.552747952	0.659676	3.8697	0.000109	0.000525
DCLK2	251.68301	-1.310916627	0.338793	-3.86937	0.000109	0.000525
KIRREL1-	8.21740552	-1.687195453	0.436314	-3.86693	0.00011	0.00053
SHOX2	45.0013089	2.043307227	0.528509	3.866175	0.000111	0.000532
MROH7	380.176756	-1.429498815	0.36975	-3.86612	0.000111	0.000532
TBXAS1	391.552092	-1.141689586	0.295452	-3.86422	0.000111	0.000535
LINC0064	29.2509176	1.481504833	0.383428	3.863845	0.000112	0.000536
PTGER4P	4.04629784	1.488249478	0.385304	3.86253	0.000112	0.000539
OFCC1	19.8055902	2.720880397	0.704646	3.861342	0.000113	0.000541
MIR5195	1.7103343	-2.615420752	0.677484	-3.86049	0.000113	0.000543
ZNF878	8.22864331	1.18930349	0.308128	3.859771	0.000113	0.000544
CD74	37581.7753	-1.218594935	0.315801	-3.85875	0.000114	0.000546
COL8A2	406.310809	-1.433137053	0.371444	-3.85828	0.000114	0.000547
CASC8	55.6170187	1.66813017	0.432442	3.857467	0.000115	0.000549
SGMS2	570.805794	-1.070219996	0.277487	-3.85682	0.000115	0.00055
MIR559	5.38728315	1.6372395	0.424538	3.856519	0.000115	0.00055
PTH2R	69.5957209	2.260408684	0.586132	3.856481	0.000115	0.00055
HS3ST1	1228.55834	-1.014195791	0.263084	-3.85502	0.000116	0.000553
SNORA15	0.84748117	1.875426873	0.486671	3.853579	0.000116	0.000556
RIPOR2	551.464811	-1.469260009	0.381408	-3.8522	0.000117	0.000559
PNPLA3	23.0017019	1.571825742	0.408043	3.852106	0.000117	0.000559
TOX3	629.05707	1.834004146	0.476258	3.850863	0.000118	0.000562
SNORA26	14.3759151	1.020042927	0.264937	3.850128	0.000118	0.000563
CAGE1	3.86436461	1.985443301	0.515884	3.848621	0.000119	0.000566
ADGRF4	450.020032	1.07924036	0.280627	3.845817	0.00012	0.000572
RNU6-786	4.65077777	-1.044743312	0.271687	-3.8454	0.00012	0.000572
GJA3	17.0717022	1.735977859	0.451525	3.8447	0.000121	0.000574
LHX8	3.6921408	-1.914118811	0.497895	-3.84442	0.000121	0.000574
DTX1	73.7187866	-1.681007579	0.437342	-3.84369	0.000121	0.000576
RPS25P3	7.56818824	-1.024993705	0.266741	-3.84266	0.000122	0.000578

PCAT19	194.207978	-1.160888158	0.302123	-3.84244	0.000122	0.000578
LIPG	574.440401	1.661123653	0.432375	3.841856	0.000122	0.00058
LRRC77P	5.83180397	-1.543072804	0.401696	-3.84139	0.000122	0.00058
C10orf88E	17.3664809	1.225246726	0.319095	3.83976	0.000123	0.000584
DLG2	102.90353	-1.149149259	0.299314	-3.83927	0.000123	0.000585
TRAV12-1	8.48539544	-1.980468466	0.515967	-3.83836	0.000124	0.000587
THRSP	1.71659544	-2.739934167	0.713833	-3.83834	0.000124	0.000587
DUSP5	3312.79529	-1.122526385	0.29253	-3.83731	0.000124	0.000589
SNORD11	1.02337288	-2.657355437	0.69273	-3.83606	0.000125	0.000592
SGCE	712.03577	-1.212022657	0.316037	-3.83507	0.000126	0.000594
LINC0276	121.110694	-1.016155439	0.264983	-3.8348	0.000126	0.000594
AEBP1	6104.53524	-1.549594286	0.404166	-3.83405	0.000126	0.000596
RNU1-47F	1.09518032	-2.675437093	0.697996	-3.83303	0.000127	0.000598
SCEL	197.722176	2.376448415	0.620136	3.832144	0.000127	0.0006
CD27	147.691713	-1.664305473	0.434317	-3.832	0.000127	0.0006
LINC0134	1.79321431	-2.52928528	0.66007	-3.83184	0.000127	0.000601
GSTM4	1252.33386	1.245643776	0.325089	3.831705	0.000127	0.000601
TYMSOS	42.6172944	1.154226054	0.301262	3.831303	0.000127	0.000602
ABHD14A	2.17086332	1.779864403	0.464564	3.831258	0.000127	0.000602
CFAP47	8.42997596	2.165058915	0.565537	3.828326	0.000129	0.000608
C16orf95-I	10.6318009	1.146970517	0.299607	3.828248	0.000129	0.000608
RPS26P21	6.89750868	-1.354102454	0.353831	-3.82697	0.00013	0.000611
MIR205H(	4153.24325	1.410095886	0.368527	3.826304	0.00013	0.000613
C1QTNF9	4.53168825	-1.943397113	0.507918	-3.8262	0.00013	0.000613
S1PR3	623.154591	-1.17465662	0.307137	-3.82454	0.000131	0.000617
VPS35P1	1.31512021	1.987750044	0.519749	3.824442	0.000131	0.000617
LINC0051	49.8544235	1.792463356	0.468718	3.824186	0.000131	0.000617
MIR126	6.6760724	-1.783787487	0.466506	-3.82372	0.000131	0.000618
LINC0114	6.37705581	-1.103066541	0.288481	-3.8237	0.000131	0.000618
CACNG4	141.629257	2.058942391	0.538496	3.823505	0.000132	0.000619
RASGEF1	177.625272	1.180667974	0.30885	3.822792	0.000132	0.00062
STAC	75.3148497	-1.664164101	0.435359	-3.82251	0.000132	0.000621
THEMIS	84.9351777	-1.623114374	0.424678	-3.82199	0.000132	0.000622
CATSPER	44.4896009	1.455121716	0.380945	3.819764	0.000134	0.000627
KLF15	179.815256	-1.187524034	0.310889	-3.81976	0.000134	0.000627
RBP4	66.2039976	-1.936372207	0.506958	-3.81959	0.000134	0.000627
RASAL1	129.046818	1.399943997	0.366517	3.819589	0.000134	0.000627
RPS20P20	9.79204897	-1.743157866	0.456415	-3.81924	0.000134	0.000627
TNS1-AS1	4.26555425	-1.463279745	0.38321	-3.81848	0.000134	0.000629
TMEM132	1.08970179	-3.221502187	0.843676	-3.81841	0.000134	0.000629
TDGF1	3.96298961	-1.883414095	0.493374	-3.81741	0.000135	0.000632
NALCN	57.794613	-1.431048634	0.374877	-3.81739	0.000135	0.000632
UBE2L4	5.00147961	1.613834875	0.422792	3.817087	0.000135	0.000632
HIGD1AP	10.2731263	-1.239303783	0.324708	-3.81667	0.000135	0.000633
PRSS35	42.6965486	-1.903610966	0.498876	-3.8158	0.000136	0.000635
HOXC-AS	9.57033328	2.86395471	0.750668	3.81521	0.000136	0.000636
SUMO2P6	16.7387865	1.05180592	0.275794	3.813743	0.000137	0.000639
COL6A5	180.707981	-1.639069238	0.42986	-3.81303	0.000137	0.000641
NECTIN4	4055.85386	1.177868933	0.308926	3.812784	0.000137	0.000642
RN7SL753	4.43737926	-1.325072946	0.347622	-3.81182	0.000138	0.000644
MPP6	189.652868	1.127524774	0.295819	3.811531	0.000138	0.000645
BNIP3P1	9.95848966	1.483233912	0.389333	3.80968	0.000139	0.000649

CSNK1A1	1.20945063	-1.913007671	0.502211	-3.80917	0.000139	0.00065
CD52	713.099028	-1.466408655	0.385105	-3.80781	0.00014	0.000653
RPL36AP3	3.67037268	-1.835742663	0.482288	-3.80632	0.000141	0.000656
KATNAL1	271.878524	-1.117472673	0.293723	-3.80451	0.000142	0.000661
EXOSC10	11.0596054	1.114985755	0.293084	3.804317	0.000142	0.000661
VCAM1	1329.26931	-1.452849611	0.382015	-3.80312	0.000143	0.000664
DOK2	257.037496	-1.366900154	0.359635	-3.8008	0.000144	0.00067
RPSAP51	2.28147044	-1.700912618	0.447564	-3.80038	0.000144	0.00067
LINC0024	45.5996053	1.136829509	0.299146	3.800252	0.000145	0.00067
LPO	3.42648331	2.310147196	0.607969	3.799775	0.000145	0.000672
PCDH7	1683.96937	-1.524564721	0.401269	-3.79936	0.000145	0.000672
LRRC25	169.915362	-1.413701613	0.372093	-3.79933	0.000145	0.000672
PARVG	600.411436	-1.31072418	0.345003	-3.79917	0.000145	0.000673
TRAJ54	0.90349488	-2.151089503	0.566201	-3.79916	0.000145	0.000673
GPR171	127.226529	-1.567208534	0.412553	-3.7988	0.000145	0.000673
COL4A3	113.981214	-1.751033108	0.461004	-3.7983	0.000146	0.000674
LINC0283	3.68796667	3.116129638	0.820685	3.796988	0.000146	0.000677
TCP10L	1.47009377	-1.386671523	0.365335	-3.79561	0.000147	0.000681
RNU4-78P	11.766251	1.141997934	0.300903	3.795238	0.000148	0.000681
MYO1F	1009.88217	-1.38804812	0.365835	-3.79419	0.000148	0.000684
MARCHF	15.0798792	1.714287819	0.451834	3.794067	0.000148	0.000684
RPL35AP2	1.47118768	2.425624637	0.639399	3.793602	0.000148	0.000685
CLVS1	13.1249971	1.534184945	0.404532	3.792495	0.000149	0.000688
LINC0127	7.08538935	1.320997689	0.348406	3.791548	0.00015	0.00069
MIR1289-	1.06800248	2.734897203	0.721472	3.790716	0.00015	0.000692
HLA-DMB	943.655898	-1.180493988	0.311449	-3.79033	0.00015	0.000692
TMEM45A	1105.50372	1.473786104	0.38884	3.790207	0.000151	0.000693
HLA-DQA	1368.97466	-1.478737604	0.390181	-3.78987	0.000151	0.000693
ANO1	975.985757	1.203926231	0.317781	3.788537	0.000152	0.000697
ITGA11	583.163442	-1.374538763	0.362889	-3.78777	0.000152	0.000699
GASK1B	1515.0253	-1.039164139	0.274384	-3.78727	0.000152	0.0007
HACD1	208.495022	-1.297214341	0.342525	-3.78721	0.000152	0.0007
LAT2	343.316576	-1.237514262	0.326868	-3.78598	0.000153	0.000703
FBXO32	1576.75634	-1.089792695	0.287947	-3.7847	0.000154	0.000707
IGKV6-21	56.9260016	-2.654824286	0.701617	-3.78386	0.000154	0.000708
ARHGAP2	3.29373742	-1.483644643	0.392132	-3.78354	0.000155	0.000709
CCDC170	130.137865	-1.193287253	0.315526	-3.7819	0.000156	0.000713
ZNF350-A	397.804922	2.638190345	0.697688	3.781335	0.000156	0.000715
LINC0259	34.2126445	1.846455067	0.488371	3.780847	0.000156	0.000716
CD3E	495.899202	-1.528108164	0.404211	-3.78047	0.000157	0.000717
ITPRIP-AS	5.15963832	1.497518322	0.396201	3.779695	0.000157	0.000719
PRSS27	118.438745	1.033309292	0.273463	3.778614	0.000158	0.000722
DENND1C	326.507525	-1.466998552	0.388238	-3.77861	0.000158	0.000722
LINC0101	10.8188553	-1.589452997	0.420683	-3.77827	0.000158	0.000723
NCF1C	134.366329	-1.416100214	0.374903	-3.77724	0.000159	0.000725
TBX20	28.5605592	-2.12017435	0.561454	-3.77622	0.000159	0.000727
LINC0246	11.7052602	3.006075097	0.796202	3.775517	0.00016	0.000729
PTPRC	2268.62391	-1.449559304	0.384061	-3.77429	0.00016	0.000732
LINC0085	2.01280895	-1.805630863	0.478404	-3.77428	0.00016	0.000732
ALOX15B	106.088914	1.931797422	0.51184	3.774225	0.000161	0.000732
LINC0042	58.3962822	-1.541322273	0.408756	-3.77076	0.000163	0.000742
PABPN1L	2.51485408	-1.723711253	0.457152	-3.77054	0.000163	0.000742

SLC18A2	231.855026	-1.503269538	0.398703	-3.7704	0.000163	0.000743
TRIM63	25.2739929	-1.887518311	0.500834	-3.76875	0.000164	0.000747
SCOCP1	4.60480329	-1.079605238	0.286496	-3.76831	0.000164	0.000748
HAPLN2	9.04987385	-1.400176606	0.371655	-3.7674	0.000165	0.000751
DGAT2	1098.46499	1.105833775	0.293735	3.764736	0.000167	0.000758
IGLL1	0.8916123	-2.117975625	0.562595	-3.76465	0.000167	0.000758
HCG20	3.39864643	-1.045618925	0.277764	-3.76441	0.000167	0.000759
TOX	151.267795	-1.392235961	0.369864	-3.76418	0.000167	0.000759
WDR64	3.81641743	-1.36304454	0.362182	-3.76342	0.000168	0.000761
H2BC18	48.5722961	1.46620191	0.389707	3.762321	0.000168	0.000764
SNTG2	26.35641	-2.031882342	0.540081	-3.76218	0.000168	0.000764
NCALD	392.777274	-1.264744346	0.336187	-3.76202	0.000169	0.000765
RTP5	8.13515315	-2.086676388	0.554701	-3.7618	0.000169	0.000765
TMEM72-	6.32073239	-1.086581609	0.288853	-3.76171	0.000169	0.000765
CEP295NI	43.1867321	-1.627352624	0.432668	-3.76121	0.000169	0.000766
PLA2G2D	97.2351602	-2.513846548	0.668549	-3.76016	0.00017	0.000769
MYO1G	491.400942	-1.394689396	0.371078	-3.75848	0.000171	0.000774
ASTL	98.9286006	-1.749865795	0.465814	-3.75657	0.000172	0.00078
RN7SKP9	15.1347803	-1.265065634	0.336942	-3.75455	0.000174	0.000785
GLIPR1	1098.22682	-1.191543972	0.317534	-3.75249	0.000175	0.00079
RNU7-13F	1.57051932	-2.352681464	0.626977	-3.75242	0.000175	0.00079
MIX23P3	6.11905317	1.17406341	0.312883	3.752404	0.000175	0.00079
SEMA3B	998.839205	-1.057756081	0.281959	-3.75145	0.000176	0.000793
CPNE5	225.715575	-1.279555077	0.341092	-3.75135	0.000176	0.000793
C6orf141	205.22977	1.457000892	0.388415	3.751149	0.000176	0.000793
IGF2BP3	299.52162	2.202485895	0.587254	3.750482	0.000176	0.000795
MIR136	0.78538335	-2.917515508	0.777938	-3.75032	0.000177	0.000795
MYOM3	33.8830483	1.509925136	0.402722	3.749297	0.000177	0.000798
DSG1-AS1	12.635012	2.486700769	0.663274	3.749133	0.000177	0.000799
MT1L	47.5039807	-1.333303739	0.355683	-3.74857	0.000178	0.0008
MIR548P	2.14694082	-1.954247213	0.521373	-3.74827	0.000178	0.000801
PLIN1	74.4822957	-1.293934223	0.345227	-3.74806	0.000178	0.000802
BIN2	435.761338	-1.355059485	0.361554	-3.74788	0.000178	0.000802
PAPSS2	689.536717	-1.089020308	0.290768	-3.74532	0.00018	0.00081
DCLK1	255.956924	-1.888148059	0.504201	-3.74483	0.000181	0.000811
MIR548AI	2.69877165	2.137358233	0.570958	3.743459	0.000182	0.000815
IGLV5-48	3.4111335	-2.10231864	0.561643	-3.74316	0.000182	0.000816
AMPD1	13.6905942	-1.910100715	0.510357	-3.74267	0.000182	0.000816
RNU6-658	3.1195092	1.281335893	0.342423	3.741969	0.000183	0.000818
RNU4-80F	14.6412384	-1.07510074	0.287375	-3.7411	0.000183	0.000821
PRKCB	903.032894	-1.547725251	0.413763	-3.74061	0.000184	0.000822
CCDC74B	4.01620416	2.428597389	0.649323	3.740202	0.000184	0.000823
SEMA3A	280.30717	-1.367662358	0.36591	-3.7377	0.000186	0.000831
RGN	85.9282132	-1.226309496	0.328235	-3.73607	0.000187	0.000836
SLCO2B1	1297.77016	-1.240872992	0.332202	-3.7353	0.000187	0.000838
COLGAL1	113.98512	-1.657951528	0.44395	-3.73455	0.000188	0.00084
HSPA7	258.668864	-1.150382337	0.308051	-3.73439	0.000188	0.000841
RNY1	11.3081875	-2.083646998	0.558024	-3.73397	0.000188	0.000841
ANKRD36	882.656438	-1.041231169	0.278855	-3.73395	0.000189	0.000841
HMGN1P1	3.61232403	2.715859585	0.727436	3.733468	0.000189	0.000843
CRIP1	68.6730753	-1.029823521	0.275893	-3.73269	0.000189	0.000845
MIR1293	2.41931972	-1.728351079	0.463061	-3.73245	0.00019	0.000846

LINC0085	9.34860132	1.110844294	0.297628	3.732327	0.00019	0.000846
PCDH8	1.22742018	-3.125796122	0.837923	-3.73041	0.000191	0.000852
LINC0174	100.170601	1.577185526	0.422816	3.730193	0.000191	0.000853
LINC0116	0.57598257	-2.280671292	0.61143	-3.73006	0.000191	0.000853
STX2	514.627604	-1.018442457	0.273052	-3.72985	0.000192	0.000854
ZNF608	522.392115	1.218642166	0.3268	3.72901	0.000192	0.000856
DEFB126	11.4997561	2.567670002	0.688568	3.729002	0.000192	0.000856
LINC0031	63.3844853	1.363737096	0.365729	3.728815	0.000192	0.000857
SLC47A2	4.78830351	2.701406703	0.724635	3.727955	0.000193	0.000859
NACAD	116.893372	-1.353373875	0.363147	-3.72679	0.000194	0.000863
AOAH-IT	1.40775532	-2.084306836	0.559411	-3.7259	0.000195	0.000866
GRIN3B	15.7213347	1.193057905	0.32022	3.72575	0.000195	0.000866
LINC0258	5.52649315	1.124017885	0.301773	3.724707	0.000196	0.000869
GGT2	40.420445	2.243862214	0.60279	3.722463	0.000197	0.000876
SFMBT2	308.097651	-1.092599623	0.293729	-3.71975	0.000199	0.000885
LINC0175	1.73686351	-2.050513542	0.55127	-3.71961	0.0002	0.000886
MIR493	0.73474786	-2.721160255	0.731692	-3.719	0.0002	0.000888
TRBV9	10.2699004	-1.782898838	0.47956	-3.71778	0.000201	0.000891
IGLV3-10	441.325471	-2.556785383	0.68773	-3.71772	0.000201	0.000892
RNU6-108	1.41789134	-2.221899294	0.597782	-3.7169	0.000202	0.000894
CCDC71L	667.195571	-1.050402959	0.282632	-3.7165	0.000202	0.000895
ZNF451-A	9.48274719	-1.318594697	0.354817	-3.71627	0.000202	0.000896
YWHAZP	23.6048602	1.108528782	0.298313	3.715989	0.000202	0.000896
PYHIN1	125.319881	-1.558447029	0.419487	-3.71513	0.000203	0.000899
TRAV22	3.43171967	-1.843600743	0.496287	-3.71478	0.000203	0.0009
CGREF1	110.605448	1.543036521	0.415455	3.71409	0.000204	0.000902
LINC0111	3.60563665	-1.371235348	0.369232	-3.71375	0.000204	0.000903
MMP25	165.511079	-1.380728978	0.371953	-3.7121	0.000206	0.000908
GRM5	3.99773502	2.357733135	0.63515	3.712089	0.000206	0.000908
RHCE	10.1052787	1.179184511	0.317709	3.711521	0.000206	0.00091
DDX10P1	22.4608678	-1.163035849	0.313368	-3.71141	0.000206	0.000911
TRAV1-2	5.76848226	-1.81782552	0.489928	-3.71039	0.000207	0.000914
MCIDAS	44.0217847	1.472068497	0.396805	3.709803	0.000207	0.000916
ZFP57	165.064014	2.948234846	0.794729	3.709737	0.000207	0.000916
GRB14	44.6836998	1.164301324	0.313893	3.709229	0.000208	0.000917
PCED1B-A	200.850473	-1.348379733	0.363796	-3.70642	0.00021	0.000926
LINC0209	3.89890962	-1.830583505	0.494223	-3.70396	0.000212	0.000935
SLC30A4	178.737772	-1.013410493	0.273651	-3.7033	0.000213	0.000937
GPR65	146.922761	-1.360704104	0.36745	-3.7031	0.000213	0.000937
S100A5	26.6786228	1.563703165	0.42228	3.703002	0.000213	0.000938
PEBP4	8.63566425	-1.374718336	0.37125	-3.70295	0.000213	0.000938
CEACAM	40.8226376	-1.372527962	0.37071	-3.70243	0.000214	0.000939
FAM133C	53.3905823	-1.213936293	0.327876	-3.70243	0.000214	0.000939
LINC0145	12.0316358	3.940571794	1.064355	3.702311	0.000214	0.000939
TLR7	147.903373	-1.174289198	0.31725	-3.70146	0.000214	0.000942
MIR1183	5.59934282	-1.153141732	0.31162	-3.70047	0.000215	0.000945
MATN3	57.2626035	1.054486454	0.28497	3.700344	0.000215	0.000946
RHOT1P3	4.65620194	1.153537558	0.311812	3.69946	0.000216	0.000948
GUCA1C	1.52981492	-3.32556903	0.899067	-3.69891	0.000217	0.00095
SASH3	445.998124	-1.502474039	0.406229	-3.69859	0.000217	0.000951
CCDC185	1.1223387	2.352387487	0.636086	3.698224	0.000217	0.000953
JAZF1-AS	5.33255171	-1.861722746	0.503495	-3.6976	0.000218	0.000955

CNIH3-AS	7.71299707	2.624539545	0.709984	3.696618	0.000218	0.000958
LINC0135	13.5746073	-1.285444822	0.347784	-3.6961	0.000219	0.00096
CORO6	198.173698	-1.277210641	0.345566	-3.696	0.000219	0.00096
EPCAM	3471.32962	1.100039973	0.297704	3.695073	0.00022	0.000963
SCN8A	56.352873	1.363102822	0.368901	3.695038	0.00022	0.000963
SNORD12	8.51920075	1.146902905	0.310459	3.694215	0.000221	0.000966
LINC0130	1.94188978	2.31903303	0.627957	3.69298	0.000222	0.000969
RNA5SP4	4.6524234	-1.228145192	0.332651	-3.692	0.000222	0.000972
HOXB13	90.952841	-1.668385297	0.451963	-3.69142	0.000223	0.000974
ZBTB32	34.2775582	-1.750492229	0.474242	-3.69114	0.000223	0.000975
IRX3	601.782317	1.651474921	0.447419	3.691116	0.000223	0.000975
NDUFS5P	1.25818273	3.113199279	0.843447	3.691045	0.000223	0.000975
CROCC2	8.11919378	-1.638790148	0.444043	-3.69062	0.000224	0.000976
IGDCC3	57.9270559	2.289810726	0.620464	3.690483	0.000224	0.000977
IGKV2D-	50.1768749	-2.271463007	0.616289	-3.68571	0.000228	0.000993
CD93	2173.40992	-1.151767751	0.312554	-3.68502	0.000229	0.000996
NRAD1	2.85813748	-1.369781795	0.371826	-3.68394	0.00023	0.001
KIF28P	5.27672994	2.31590938	0.628745	3.683386	0.00023	0.001001
ANXA10	1772.10118	2.151941682	0.584358	3.682574	0.000231	0.001004
ATP6V0D	15.4750543	1.944710153	0.528103	3.682442	0.000231	0.001004
CSF2RB	909.453164	-1.516379486	0.412057	-3.68002	0.000233	0.001013
ENPP2	1207.68968	-1.078994214	0.293208	-3.67996	0.000233	0.001013
SSBL2P	6.32650916	1.088102135	0.295725	3.679442	0.000234	0.001015
DPYS	3.60124114	-2.158760393	0.586924	-3.67809	0.000235	0.001019
TRIM15	4.10670829	3.03906623	0.826309	3.677881	0.000235	0.00102
TMEM15	116.506142	-1.451331465	0.394691	-3.67713	0.000236	0.001022
SNORD11	0.81749778	-2.246758018	0.611017	-3.67708	0.000236	0.001022
LINC0052	8.01106645	1.237116836	0.336444	3.677031	0.000236	0.001022
TRBV20-1	30.7571877	-1.726917659	0.469687	-3.67674	0.000236	0.001023
NXN	1092.70222	-1.074461139	0.292389	-3.67477	0.000238	0.001031
HSD11B1-	9.83959651	-1.13552845	0.309008	-3.67476	0.000238	0.001031
SELENOP	8933.97175	-1.069944248	0.291199	-3.67427	0.000239	0.001032
ITPKB-IT	4.33897003	-1.406858157	0.383129	-3.67202	0.000241	0.001041
KCTD9P4	7.51814092	-1.07984038	0.294078	-3.67195	0.000241	0.001041
GPR18	25.0433859	-1.829455759	0.498238	-3.67185	0.000241	0.001041
APCDD1	1742.93546	-1.057715426	0.288087	-3.67152	0.000241	0.001042
MRPS24	14.2204119	-1.168653457	0.318308	-3.67145	0.000241	0.001042
ZSCAN5A	4.88213376	1.159803697	0.31599	3.670381	0.000242	0.001046
LILRA5	103.57958	-1.582169773	0.431088	-3.67018	0.000242	0.001046
EPB41L4E	492.77896	1.057258419	0.28807	3.670139	0.000242	0.001046
MPO	9.1000978	-1.283965763	0.34989	-3.66963	0.000243	0.001048
C1QL4	1.80531672	2.135677773	0.582184	3.66839	0.000244	0.001053
MIR483	2.37142487	3.608837704	0.983884	3.66795	0.000245	0.001054
PGA3	1.18957068	-2.962182046	0.807873	-3.66664	0.000246	0.001059
ANGPTL6	21.6572295	-1.161451208	0.316809	-3.66609	0.000246	0.001061
ZNF793-A	63.6487664	1.115599864	0.304324	3.665832	0.000247	0.001061
MT2A	2495.51197	-1.235488266	0.337291	-3.66297	0.000249	0.001072
HS6ST2	378.359148	1.262817086	0.34478	3.662673	0.00025	0.001073
ARHGAP	244.882304	-1.122749124	0.30655	-3.66253	0.00025	0.001074
GTF2IP7	14.0774497	-1.189978697	0.324918	-3.66239	0.00025	0.001074
OR7E108I	3.40421656	2.221837219	0.60667	3.662349	0.00025	0.001074
LINC0088	1.77166297	-1.822937054	0.497921	-3.6611	0.000251	0.001078

IGLV2-18	18.2477793	-2.563739946	0.700554	-3.65959	0.000253	0.001084
RPE65	3.8751931	-2.247703605	0.614354	-3.65865	0.000254	0.001088
GNGT2	37.4643224	-1.137947699	0.311115	-3.65764	0.000255	0.001091
CACNA1(	1.89013942	-4.161393395	1.137788	-3.65744	0.000255	0.001092
WFIKKN2	4.20102874	-1.599019742	0.437457	-3.65526	0.000257	0.0011
ELAVL2	28.772445	1.961192041	0.536777	3.653646	0.000259	0.001106
HHIPL1	97.0786751	-1.172970486	0.321056	-3.65348	0.000259	0.001106
OR1H1P	5.00410011	2.199190114	0.602024	3.652995	0.000259	0.001108
EPHB6	2126.88904	1.25904982	0.344729	3.652289	0.00026	0.001111
DYSF	910.571571	-1.221376702	0.334602	-3.65023	0.000262	0.001119
GALNT9	3.66124661	-1.727828554	0.473348	-3.65023	0.000262	0.001119
IL17RD	259.153742	-1.055519304	0.289179	-3.65006	0.000262	0.001119
C12orf75	467.114969	1.191160424	0.326367	3.649762	0.000262	0.00112
SLC2A3	2911.97022	-1.307276474	0.358215	-3.64942	0.000263	0.001122
SLCO2A1	1279.44661	-1.434050141	0.392977	-3.6492	0.000263	0.001122
TMEM108	87.9101832	-1.793175511	0.491517	-3.64825	0.000264	0.001126
FOSL1	983.047669	-1.209149536	0.331466	-3.64788	0.000264	0.001128
PTGIR	91.49029	-1.279333054	0.350811	-3.64679	0.000266	0.001132
NTNG2	82.9970277	-1.228185607	0.336835	-3.64625	0.000266	0.001134
LSMEM2	5.65206432	-1.171217064	0.321331	-3.6449	0.000267	0.00114
COL18A1	4645.09836	-1.176728128	0.322843	-3.64489	0.000268	0.00114
GAS1	368.031874	-1.786492112	0.490441	-3.64263	0.00027	0.001149
SERPINB1	4.19490663	3.898965733	1.0705	3.642191	0.00027	0.001151
H3P36	4.91970608	1.241779634	0.34095	3.642116	0.00027	0.001151
LINC0231	33.6026648	2.846813344	0.781717	3.641745	0.000271	0.001152
PGAM1P1	0.85529069	-2.765930685	0.759723	-3.64071	0.000272	0.001156
LINC0289	72.4360704	2.211176598	0.607427	3.640232	0.000272	0.001158
ZDHHC20	1.99805243	-1.538679879	0.422724	-3.63992	0.000273	0.001159
GJC1	656.051381	-1.278875247	0.351364	-3.63974	0.000273	0.001159
KCNA7	1.76509984	3.167249216	0.870575	3.638112	0.000275	0.001165
NRP2	1020.87885	-1.269208742	0.34887	-3.63806	0.000275	0.001165
CBX6	2562.02758	-1.030498157	0.283273	-3.63783	0.000275	0.001166
ARRDC5	34.2505552	-1.683434769	0.462779	-3.63766	0.000275	0.001166
SLAMF6	182.530146	-1.620167037	0.445425	-3.63735	0.000275	0.001167
LRP4	464.883647	1.070025176	0.294222	3.6368	0.000276	0.00117
FTLP14	52.2230241	1.055221408	0.290174	3.636509	0.000276	0.001171
UCA1	2698.72197	1.938450385	0.533214	3.635405	0.000278	0.001175
SDC1	20192.1923	1.035293063	0.284809	3.635042	0.000278	0.001177
LINC0212	3.04982832	3.106458846	0.854652	3.634766	0.000278	0.001178
LINC0153	70.261984	1.242748106	0.341984	3.633935	0.000279	0.001181
MPPED1	20.924028	-2.091130887	0.575687	-3.63241	0.000281	0.001187
FCRL4	9.76479958	-2.872072865	0.790935	-3.63124	0.000282	0.001191
FOXD4	25.5624102	1.147334528	0.316034	3.630418	0.000283	0.001195
SNORD11	0.91373535	-2.337764275	0.644018	-3.62997	0.000283	0.001197
CACTIN-A	8.19770428	-1.004824645	0.276833	-3.62972	0.000284	0.001198
CYTIP	445.425879	-1.328306312	0.366005	-3.6292	0.000284	0.001199
DCDC1	8.26826052	1.466757538	0.404155	3.629196	0.000284	0.001199
HCST	227.748147	-1.280562754	0.352872	-3.62897	0.000285	0.0012
LINC0156	3.69660319	-1.160145542	0.319746	-3.62833	0.000285	0.001202
KLRG2	24.1573249	1.92034232	0.5293	3.628079	0.000286	0.001203
ASS1P1	10.728641	-1.974094998	0.544251	-3.62718	0.000287	0.001206
SNAI1	142.614178	-1.312948284	0.36198	-3.62713	0.000287	0.001206

CHRDL2	400.216289	-2.257151395	0.622534	-3.62575	0.000288	0.001213
PDLIM4	692.165849	-1.181127783	0.325915	-3.62404	0.00029	0.00122
MEIS3P2	36.2768438	-1.311114516	0.361795	-3.62391	0.00029	0.00122
CCL13	153.843827	-1.424609912	0.393229	-3.62285	0.000291	0.001225
GTSF1	75.6245738	2.283372729	0.630284	3.622768	0.000291	0.001225
TATDN1F	2.7768257	1.173228903	0.324015	3.620911	0.000294	0.001233
MYL7	1.80286326	-1.968419372	0.54373	-3.62022	0.000294	0.001236
LINC0040	21.9119683	-2.35955719	0.651937	-3.6193	0.000295	0.00124
VAT1L	66.3573915	-1.422906475	0.393176	-3.619	0.000296	0.001241
MEFV	87.2546938	-1.362062686	0.37637	-3.61894	0.000296	0.001241
ALOX12P	226.393196	1.375900797	0.380207	3.618822	0.000296	0.001241
IGHV1-58	77.6158811	-3.093553897	0.854873	-3.61873	0.000296	0.001241
SSC4D	18.3608778	1.008278338	0.278651	3.618433	0.000296	0.001242
CAND2	142.560696	-1.198904159	0.331359	-3.61814	0.000297	0.001243
DPY19L2I	16.4297662	1.731037921	0.478439	3.618094	0.000297	0.001243
F3	3141.18504	-1.207529451	0.333775	-3.6178	0.000297	0.001245
ELFN1-AS	18.0418401	1.794622745	0.496185	3.616838	0.000298	0.001249
PCDHGA9	33.0239643	-1.082359253	0.299447	-3.61453	0.000301	0.001259
PSLNR	15.6385324	3.094040717	0.856057	3.614291	0.000301	0.00126
TEX11	14.0343894	-1.025482071	0.283731	-3.61427	0.000301	0.00126
G2E3-AS1	20.7337875	2.816087761	0.779207	3.614043	0.000301	0.001261
BEND7P1	3.1976532	-1.3332509	0.368967	-3.61347	0.000302	0.001263
CLEC4E	85.5528504	-1.547798493	0.428433	-3.6127	0.000303	0.001266
LGALS2	85.1837637	-1.750976455	0.484854	-3.61135	0.000305	0.001272
GAB3	205.168038	-1.162360562	0.32187	-3.61127	0.000305	0.001272
SEPTIN1	90.0947896	-1.0785795	0.298746	-3.61036	0.000306	0.001276
IL37	3.6543213	1.65725216	0.459138	3.609485	0.000307	0.00128
LINC0182	2.47395102	2.448781267	0.678585	3.608657	0.000308	0.001283
C5orf67	0.95823378	-2.196567896	0.60876	-3.60827	0.000308	0.001285
NGF-AS1	1.04517031	-2.271807159	0.629627	-3.60818	0.000308	0.001285
WNT4	135.791242	-1.158366594	0.321124	-3.60722	0.000309	0.001288
TRPC4	40.3627085	-1.331523277	0.36924	-3.60612	0.000311	0.001293
KLHL10	4.24610382	-1.240955172	0.344144	-3.60592	0.000311	0.001294
JCAD	1046.3811	-1.234060142	0.342262	-3.60561	0.000311	0.001295
BOLA2P3	2.95157372	1.731251714	0.480215	3.605161	0.000312	0.001297
HSPA9P1	4.03382985	1.154159096	0.32016	3.604944	0.000312	0.001298
SLCO1C1	15.6670658	-1.184463861	0.328624	-3.60431	0.000313	0.0013
ZNF556	23.0764502	2.367647303	0.657151	3.602898	0.000315	0.001307
RNY3P16	4.34758881	-1.150804238	0.319534	-3.6015	0.000316	0.001313
GDF3	1.80619409	-1.550072336	0.430409	-3.60139	0.000317	0.001314
FAM183A	113.668792	1.218158956	0.338308	3.600741	0.000317	0.001317
DPY19L3-	13.0639634	1.052666416	0.292407	3.600007	0.000318	0.00132
S100A2	9615.4684	1.57105048	0.437197	3.593463	0.000326	0.001352
TRBV6-5	10.9372604	-1.57566047	0.43852	-3.59313	0.000327	0.001353
LINC0083	150.198604	1.737551592	0.483609	3.592884	0.000327	0.001354
TRAJ32	1.15243722	-2.403718474	0.669144	-3.59223	0.000328	0.001357
CDHR4	8.26054115	-1.135757944	0.316374	-3.58993	0.000331	0.001366
CLU	12694.8427	-1.467673225	0.408904	-3.58928	0.000332	0.001369
RPL17P13	2.56522388	2.033892093	0.56697	3.587299	0.000334	0.001379
MDK	5059.19271	1.034420777	0.2884	3.586762	0.000335	0.001382
TRAJ4	1.84502117	-1.993306945	0.555746	-3.58672	0.000335	0.001382
MIR3660	0.98194962	2.927777385	0.816348	3.586432	0.000335	0.001383

RPSAP3	10.4032523	1.008147998	0.281202	3.585139	0.000337	0.001389
TNFAIP8I	78.5848259	-1.149658062	0.320716	-3.58466	0.000338	0.001391
NR1I2	8.73479326	1.409378212	0.393255	3.583875	0.000339	0.001395
RPL17P22	1.560585	-1.945916776	0.543026	-3.58347	0.000339	0.001397
NPY	1.87357563	-2.568946572	0.716905	-3.58339	0.000339	0.001397
SIT1	73.6050141	-1.399741166	0.390635	-3.58324	0.000339	0.001398
ZDHHC20	1.13904299	1.995146539	0.556808	3.583187	0.000339	0.001398
ZNF454	26.6722368	-1.33364821	0.372282	-3.58236	0.000341	0.001402
RNU7-48F	2.0496286	2.149578337	0.600112	3.581959	0.000341	0.001403
PCDHA3	5.26878255	-1.815078405	0.506963	-3.5803	0.000343	0.001411
HNRNPA1	2.92134776	-2.46380201	0.688249	-3.57981	0.000344	0.001414
FAM135A	3.21236899	1.162097747	0.32463	3.579762	0.000344	0.001414
RORA	606.084872	-1.027578619	0.287198	-3.57794	0.000346	0.001423
MKRN2O:	15.3738921	1.478603965	0.413603	3.574933	0.00035	0.001437
TRAV9-2	10.5358492	-1.836472988	0.513838	-3.57403	0.000352	0.001442
DCDC2C	0.98664918	-2.925731589	0.818638	-3.5739	0.000352	0.001442
LRRC37A	12.721785	1.328852467	0.371833	3.573785	0.000352	0.001442
LINC0120	2.62375871	2.665113016	0.746057	3.572266	0.000354	0.00145
RGS1	2104.32741	-1.091123222	0.30561	-3.57031	0.000357	0.00146
ACER1	2.1469969	-1.608869168	0.450638	-3.5702	0.000357	0.00146
LYPLAL1	4.84146685	-1.430714772	0.401019	-3.5677	0.00036	0.001473
KLHL33	5.49790243	-2.022901044	0.567075	-3.56726	0.000361	0.001475
LRRC7	15.6937475	-1.920608799	0.538621	-3.56579	0.000363	0.001481
LTA	27.2207586	-1.503376974	0.421637	-3.56557	0.000363	0.001482
KRT7	31061.2972	1.191058992	0.334087	3.565115	0.000364	0.001484
ESYT3	54.7540188	1.514820298	0.424919	3.564966	0.000364	0.001485
ADM	1760.75384	1.147404528	0.321863	3.564886	0.000364	0.001485
LCK	406.681832	-1.255252544	0.352175	-3.56428	0.000365	0.001488
DHCR24	3601.29633	1.285981635	0.360853	3.563731	0.000366	0.00149
GSN-AS1	1.44998589	-2.147004969	0.602488	-3.56357	0.000366	0.001491
LINC0068	8.26330982	-1.922229312	0.539494	-3.56302	0.000367	0.001493
SHISAL2/	33.8993192	-1.339449622	0.376001	-3.56235	0.000368	0.001497
HCK	484.693376	-1.246296233	0.349924	-3.56162	0.000369	0.0015
NAP1L3	94.2524164	-1.501810826	0.421718	-3.56118	0.000369	0.001502
IL7R	1266.14498	-1.413693509	0.397023	-3.56074	0.00037	0.001505
CACNA1I	168.210065	1.663086703	0.467109	3.560379	0.00037	0.001506
TMEM52I	28.4921831	1.441494968	0.404909	3.560043	0.000371	0.001508
CBLN4	2.92305871	-2.13057064	0.59847	-3.56003	0.000371	0.001508
TAFA5	128.338814	-1.271891681	0.357352	-3.55921	0.000372	0.001512
FAM83C-/	3.47640875	1.299682834	0.365183	3.558991	0.000372	0.001513
TRBV19	19.4046225	-1.719865266	0.483257	-3.5589	0.000372	0.001513
TMEM20(	40.4707702	-1.410911173	0.396662	-3.55696	0.000375	0.001523
PDE3A	207.835786	-1.047678604	0.294584	-3.55647	0.000376	0.001525
MSX2P1	1.11824642	-2.837317857	0.798021	-3.55544	0.000377	0.00153
LY6D	5592.41614	2.14022847	0.60202	3.555079	0.000378	0.001532
RNY3	3.43423516	-2.933363191	0.825166	-3.55488	0.000378	0.001533
BNIP3	885.734188	1.107123929	0.311501	3.554155	0.000379	0.001537
ZNF483	44.4711939	-1.236945037	0.348035	-3.55408	0.000379	0.001537
CKB	2829.02512	-1.274772464	0.358835	-3.55253	0.000382	0.001545
ACTN2	53.2386389	-1.935887534	0.545076	-3.55159	0.000383	0.001551
TSPEAR-/	32.0814935	2.13069542	0.600001	3.551155	0.000384	0.001553
SAMD14	91.8414978	-1.064015871	0.299651	-3.55084	0.000384	0.001554

CCNYL2	65.0798211	2.785657091	0.784715	3.549894	0.000385	0.001559
BRWD1-A	3.49057167	1.238144967	0.348855	3.549164	0.000386	0.001563
TRAV8-6	9.69440483	-1.82481741	0.514197	-3.54887	0.000387	0.001564
LMO3	564.781815	-1.85687754	0.523236	-3.54883	0.000387	0.001564
EEF1A2	432.334023	1.646411583	0.464024	3.548119	0.000388	0.001568
BEX2	386.321743	1.024966076	0.288991	3.546707	0.00039	0.001576
LINC0257	1.99288942	3.185822414	0.898373	3.546214	0.000391	0.001578
IGHV1-46	213.212519	-2.168989575	0.611701	-3.54583	0.000391	0.001579
CD3G	190.74372	-1.335657158	0.376729	-3.5454	0.000392	0.001582
LRRC4C	42.4628079	-1.499438672	0.422943	-3.54525	0.000392	0.001582
CD14	1634.83934	-1.17532702	0.331638	-3.54401	0.000394	0.001589
COL15A1	3008.28654	-1.358735795	0.383714	-3.54101	0.000399	0.001604
TRAJ53	1.05460319	-2.422703316	0.68426	-3.54062	0.000399	0.001605
C5AR2	73.0954679	-1.36097942	0.384561	-3.53904	0.000402	0.001615
SDK1-AS1	8.44006168	-1.44555238	0.408602	-3.5378	0.000403	0.001621
RNU6ATA	12.4561047	-1.146864123	0.324347	-3.53591	0.000406	0.001631
FUT2	337.33768	-1.366229577	0.386448	-3.53535	0.000407	0.001634
RNU6-122	0.51151894	-2.200077356	0.622445	-3.53457	0.000408	0.001638
TLR10	117.857136	-1.687246802	0.47741	-3.53417	0.000409	0.00164
TMEFF1	3.73249167	2.258396835	0.639042	3.534033	0.000409	0.001641
TRBV6-1	9.04788895	-1.806874676	0.511399	-3.5332	0.000411	0.001645
LINC0242	5.32394451	-1.189058821	0.336587	-3.53269	0.000411	0.001648
ENOX1	87.0307267	-1.26025271	0.356886	-3.53124	0.000414	0.001655
ACTBP2	11.8990959	-1.12589098	0.318998	-3.52946	0.000416	0.001665
IQSEC3	52.2486704	-1.48161407	0.42007	-3.52707	0.00042	0.001679
ONECUT1	6.15060235	2.665167812	0.75575	3.526522	0.000421	0.001681
EVPL	4453.84961	1.036397064	0.294002	3.525134	0.000423	0.00169
ECM1	789.00909	-1.030729599	0.292401	-3.52506	0.000423	0.00169
ZNF415P1	16.2119345	-1.386523318	0.393381	-3.52463	0.000424	0.001692
H2AC10P	0.97492281	2.161085759	0.613144	3.524597	0.000424	0.001692
SVIL2P	11.4117964	-1.332682159	0.378235	-3.52342	0.000426	0.001699
PCAT7	71.0080606	1.375359452	0.390467	3.522341	0.000428	0.001704
KBTBD11	176.269484	-1.090246919	0.309577	-3.52173	0.000429	0.001707
AACSP1	6.92046105	2.61549931	0.743082	3.5198	0.000432	0.001719
DYNLT5	37.5782534	-1.38360996	0.393163	-3.51918	0.000433	0.001722
ANKFN1	82.8179769	-1.719096919	0.488502	-3.51912	0.000433	0.001722
FBN1	2676.41979	-1.392951437	0.39587	-3.51871	0.000434	0.001724
RHAG	1.00249515	-2.376341764	0.675591	-3.51743	0.000436	0.001733
COSMOC	80.8571221	1.137163544	0.323348	3.516837	0.000437	0.001736
MIR378A	3.94852484	-1.175758379	0.33444	-3.51561	0.000439	0.001744
AOAH	311.755782	-1.343530216	0.382181	-3.51543	0.000439	0.001744
SIRPB2	61.4747845	-1.132072882	0.322034	-3.51538	0.000439	0.001745
HOXC11	40.8792098	1.692525513	0.481506	3.515065	0.00044	0.001746
RNU6-856	5.3581432	1.00176845	0.28503	3.514607	0.00044	0.001749
SNORD39	50.8760712	-1.672105745	0.475762	-3.51459	0.00044	0.001749
RNU6-925	5.22598279	1.190015857	0.338614	3.514372	0.000441	0.00175
SEMA3E	306.941275	-1.393843858	0.396624	-3.51427	0.000441	0.00175
GPR68	270.471364	-1.395630187	0.397184	-3.51381	0.000442	0.001753
LIN28B	39.7098265	3.860326064	1.098781	3.513282	0.000443	0.001756
CCR2	137.784134	-1.598881067	0.455426	-3.51074	0.000447	0.001772
VIM-AS1	94.1713254	-1.281879245	0.365237	-3.50972	0.000449	0.001778
OR1Q1	2.86613638	2.400838855	0.684463	3.507623	0.000452	0.00179

CYP26A1	7.02479096	1.851506931	0.527963	3.506886	0.000453	0.001794
KSR2	133.480845	1.245867534	0.355293	3.506589	0.000454	0.001795
FERMT3	617.708746	-1.129226848	0.322063	-3.50623	0.000454	0.001797
DLGAP2	2.54332113	-1.525562259	0.435101	-3.50622	0.000455	0.001797
RIMKLA	37.4890453	1.472780313	0.420104	3.505751	0.000455	0.001799
HCAR1	204.528692	1.284912923	0.366585	3.505086	0.000456	0.001803
CCDC78	58.5382729	1.135238249	0.323925	3.504635	0.000457	0.001806
LINC0198	93.6013448	3.096796458	0.88391	3.50352	0.000459	0.001812
SNORD53	1.93266378	1.629011327	0.464994	3.503299	0.00046	0.001813
PROK2	39.3093066	-1.684604725	0.48091	-3.50295	0.00046	0.001815
CD3D	241.953779	-1.298662093	0.370874	-3.50162	0.000462	0.001823
TRBV6-6	4.7160641	-1.979654864	0.565531	-3.50053	0.000464	0.00183
SLC9A4	205.758871	1.797937376	0.513639	3.500389	0.000465	0.00183
C1QA	2524.7448	-1.150528417	0.328776	-3.49943	0.000466	0.001836
UBE2Q2P	5.69622061	1.289022738	0.368398	3.498998	0.000467	0.001839
SCARF2	362.455065	-1.134170071	0.32425	-3.49783	0.000469	0.001846
TTC24	24.8329125	-1.682823962	0.481247	-3.4968	0.000471	0.001852
RN7SL40I	2.24411801	-1.257463116	0.359612	-3.49672	0.000471	0.001852
CRP	1.29999149	-1.613210757	0.461381	-3.49649	0.000471	0.001853
ZNF559-Z	3.90227859	1.399352921	0.400263	3.496083	0.000472	0.001855
PCDH11X	7.88166783	-1.708514594	0.489019	-3.49376	0.000476	0.00187
ANKRD3:	16.7018179	-1.373905456	0.393547	-3.49109	0.000481	0.001888
CLCA3P	16.18477	1.98953582	0.570146	3.48952	0.000484	0.001898
RPL21P99	2.66969899	-1.90084796	0.544809	-3.48902	0.000485	0.001901
CEP250-A	18.7810946	-1.297698503	0.371989	-3.48854	0.000486	0.001904
OSR2	237.177656	-1.256064759	0.360108	-3.48803	0.000487	0.001907
MIR335	1.05397939	2.989627342	0.857278	3.487351	0.000488	0.001911
TRAV26-1	7.07428854	-1.822068674	0.522508	-3.48716	0.000488	0.001912
XYLT1	334.297586	-1.086137123	0.311616	-3.4855	0.000491	0.001923
HNRNPA1	0.9973096	-1.879313362	0.539323	-3.48458	0.000493	0.001928
ATP5PBP	3.90437212	-1.115268246	0.320122	-3.48389	0.000494	0.001932
DNM1	640.631652	-1.140199283	0.327446	-3.4821	0.000498	0.001944
CACNA2I	379.645163	-1.342491946	0.38606	-3.47742	0.000506	0.001976
TRAV27	3.67166081	-1.804754847	0.519004	-3.47734	0.000506	0.001977
RBBP8NL	222.402994	1.239343457	0.356412	3.47728	0.000507	0.001977
TRBV11-2	9.37627444	-1.851827793	0.532629	-3.47677	0.000507	0.00198
LINC0045	2.75931664	2.368637673	0.681312	3.476583	0.000508	0.001981
GPR141	17.3699342	-1.203934539	0.346372	-3.47584	0.000509	0.001986
LINC0100	6.99655882	-1.186138204	0.341315	-3.4752	0.00051	0.001991
TFPI2-DT	7.25021296	1.388706209	0.399691	3.474449	0.000512	0.001996
MRPS18C	2.3094799	-1.406434783	0.405302	-3.47009	0.00052	0.002026
LINC0193	20.9196715	-1.55748991	0.448972	-3.46901	0.000522	0.002033
TRAJ3	4.53470682	-1.833400675	0.528521	-3.46893	0.000523	0.002034
CD226	46.5851379	-1.204498042	0.347264	-3.46853	0.000523	0.002036
ART4	12.1525762	-1.766444643	0.509452	-3.46734	0.000526	0.002045
CFLAR-A	3.54802736	-1.430261566	0.412503	-3.46727	0.000526	0.002045
SNAP91	58.006868	1.730688202	0.499365	3.465779	0.000529	0.002055
ALX3	8.22163552	2.4590093	0.709725	3.464737	0.000531	0.002061
LINC0225	26.2697242	2.878482499	0.831035	3.463732	0.000533	0.002068
ZNF98	18.9682652	1.872971661	0.540815	3.46324	0.000534	0.002071
L1TD1	9.78496872	-1.395028912	0.402881	-3.46263	0.000535	0.002075
KCNS1	25.5093434	1.309521429	0.378191	3.462597	0.000535	0.002075

SLC27A6	52.1259019	-1.437445648	0.415144	-3.46252	0.000535	0.002076
AMHR2	3.40578927	-1.615934149	0.466708	-3.46241	0.000535	0.002076
KIF1C-AS	11.2517215	-1.126808462	0.325441	-3.46241	0.000535	0.002076
RNU6-501	6.72956608	-1.834996297	0.53022	-3.46082	0.000539	0.002086
FAM242C	12.9368285	1.022403531	0.295457	3.460417	0.000539	0.002089
DPYD	731.480151	-1.042066723	0.301203	-3.45968	0.000541	0.002093
ITGBL1	384.591738	-1.318792121	0.381239	-3.45923	0.000542	0.002096
HSPB3	15.5609188	-2.572701155	0.743758	-3.45906	0.000542	0.002097
APOBR	744.739591	-1.099133348	0.317767	-3.45893	0.000542	0.002098
DDX11L1	3.47246707	1.806823148	0.522427	3.458515	0.000543	0.002101
TRAV17	12.498075	-1.721081311	0.497824	-3.4572	0.000546	0.002111
IGHV1-17	1.39021684	-2.923370004	0.845595	-3.45717	0.000546	0.002111
LINC0170	5.21168704	3.161138725	0.914575	3.456403	0.000547	0.002116
CD53	1187.96361	-1.273792085	0.368533	-3.45639	0.000547	0.002116
TRAV8-3	11.5362969	-1.63409888	0.472966	-3.455	0.00055	0.002124
COQ7-DT	4.86996626	1.33305447	0.385891	3.454487	0.000551	0.002127
OR11H13I	1.07497448	2.766790887	0.801184	3.453377	0.000554	0.002135
LINC0163	3.98893473	1.436056409	0.415848	3.453324	0.000554	0.002135
IRF4	308.657045	-1.56204478	0.452499	-3.45204	0.000556	0.002143
IRX5	103.259107	1.446635782	0.419111	3.451676	0.000557	0.002145
MYB	178.255211	1.181138786	0.342313	3.450466	0.00056	0.002154
LMO7DN-	1.38300387	-1.485223348	0.430452	-3.45038	0.00056	0.002154
LINC0167	3.53712625	1.514439282	0.438969	3.449988	0.000561	0.002157
PRKAA2	216.702784	-1.378992177	0.399778	-3.44939	0.000562	0.002162
PKMP5	1.33408397	-1.564679123	0.453792	-3.44801	0.000565	0.002171
RNU6-31F	0.7159733	-1.779922815	0.51641	-3.44672	0.000567	0.00218
SNORD11	0.94846907	-2.213035051	0.642081	-3.44666	0.000568	0.00218
POTEKP	12.7486564	2.760180623	0.800976	3.446023	0.000569	0.002185
POF1B	1544.77186	1.073707423	0.311712	3.444547	0.000572	0.002196
IGHV3-53	157.418006	-2.338771267	0.679011	-3.44438	0.000572	0.002196
TRBV11-1	1.73188063	-2.522737381	0.732502	-3.444	0.000573	0.002199
GDPD3	496.968684	1.124797153	0.326714	3.442758	0.000576	0.002208
TNFRSF8	36.0926725	-1.511876471	0.439256	-3.4419	0.000578	0.002214
MIR381H(	0.7226085	-2.772467373	0.805614	-3.44144	0.000579	0.002217
CYTH4	648.820876	-1.323822001	0.384694	-3.44124	0.000579	0.002219
RPS3P7	3.98393829	-1.314851241	0.38212	-3.44094	0.00058	0.002221
MAGED4	44.2541876	1.350342854	0.392558	3.439859	0.000582	0.002229
LINC0123	24.4108819	2.528390838	0.735211	3.439002	0.000584	0.002235
ALOX15	147.562573	1.271334605	0.369722	3.438627	0.000585	0.002238
PLCXD2	39.3116331	1.083385716	0.315089	3.438345	0.000585	0.00224
TLX3	6.67486499	3.136831214	0.912463	3.437763	0.000587	0.002244
SACS	468.831815	-1.150422563	0.334766	-3.43649	0.000589	0.002252
LIFR	835.431617	-1.232900025	0.358863	-3.43557	0.000591	0.002259
PAEP	3.41961672	3.351853741	0.975762	3.435115	0.000592	0.002262
ZNRF3-AS	1.03122157	-1.809318744	0.526762	-3.4348	0.000593	0.002264
LINC0109	4.31431493	2.52221534	0.734506	3.433892	0.000595	0.002271
SPN	341.432773	-1.329676199	0.387372	-3.43256	0.000598	0.00228
KCNG1	190.300818	1.425479862	0.415421	3.431407	0.0006	0.002288
TMEM17(	1182.32071	-1.318547161	0.384297	-3.43106	0.000601	0.00229
TRAV12-3	8.83523187	-1.553319761	0.453036	-3.42869	0.000607	0.002307
CD69	470.137153	-1.371968891	0.40034	-3.42701	0.00061	0.00232
CORIN	72.2428818	-1.451023405	0.42341	-3.42699	0.00061	0.00232

E2F3-IT1	3.62798333	1.51845854	0.443115	3.426783	0.000611	0.002322
MIR4271	1.50875436	-1.743063231	0.508864	-3.4254	0.000614	0.002333
ALMS1P1	23.2757063	1.287428997	0.37601	3.423918	0.000617	0.002345
CEACAM	31.3693448	-1.477284258	0.431479	-3.42377	0.000618	0.002346
H2BC15	18.7288976	1.086342128	0.31731	3.423601	0.000618	0.002347
IL6R-AS1	5.4036637	-1.133846121	0.331233	-3.42311	0.000619	0.002349
SPAG16-D	3.19550688	1.221530301	0.357116	3.420546	0.000625	0.00237
PAX5	178.343085	-2.156200381	0.630393	-3.42041	0.000625	0.002371
PHF24	7.0007144	-1.726807638	0.50488	-3.42024	0.000626	0.002371
IGHV7-56	1.18785856	-2.766032513	0.808728	-3.42023	0.000626	0.002371
MAG	2.60170041	-2.016471305	0.590222	-3.41646	0.000634	0.0024
ERVE-1	264.942756	1.607088449	0.470445	3.416101	0.000635	0.002402
THSD7A	132.888878	-1.153038945	0.337616	-3.41524	0.000637	0.002409
HOXC5	7.88893973	1.677227266	0.491261	3.414124	0.00064	0.002418
GPR82	33.5817097	-1.250813289	0.366372	-3.41406	0.00064	0.002418
GBP1P1	213.437404	1.149054287	0.336612	3.413588	0.000641	0.002422
CIITA	984.667859	-1.062505324	0.311486	-3.41109	0.000647	0.002442
LINC0268	11.4715165	-1.374311901	0.402918	-3.4109	0.000647	0.002443
HOXC10	49.553443	2.166650295	0.635252	3.410693	0.000648	0.002444
LINC0257	46.5395589	2.318378382	0.679857	3.410098	0.000649	0.002449
SNORD53	2.02960128	1.425514789	0.418102	3.409491	0.000651	0.002454
TMPRSS3	114.151327	1.356681568	0.398028	3.408512	0.000653	0.00246
RPL21P65	3.06776309	1.382957147	0.405778	3.408165	0.000654	0.002462
LANCL3	71.2756901	1.014000517	0.297545	3.407887	0.000655	0.002463
SSTR5-AS	65.9935087	2.353659207	0.690788	3.40721	0.000656	0.002468
TRGV2	3.4436866	-2.023811717	0.594222	-3.40582	0.00066	0.00248
CYP7A1	2.01923131	-1.457557131	0.428134	-3.40444	0.000663	0.002491
RN7SL45I	2.77686485	1.434842612	0.421495	3.404174	0.000664	0.002493
PDZD7	38.8551025	1.014828071	0.298135	3.403924	0.000664	0.002495
MCOLN2	120.467049	-1.231916596	0.361919	-3.40384	0.000664	0.002495
SIGLEC8	49.0466973	-1.191582469	0.350077	-3.40377	0.000665	0.002495
LSAMP-A	3.28827243	2.168431099	0.63731	3.402474	0.000668	0.002506
RN7SKP1	1.28202709	-2.201125374	0.647084	-3.40161	0.00067	0.002513
ZNF486	1419.21841	1.104647936	0.324774	3.401281	0.000671	0.002515
LINC0197	14.1928086	1.227456206	0.360974	3.400403	0.000673	0.002523
AADACP1	36.6408329	1.816302139	0.534177	3.400186	0.000673	0.002524
CORO1A-	20.421825	-1.214321514	0.357199	-3.39956	0.000675	0.002529
RBP1	523.828292	-1.090095737	0.320721	-3.39889	0.000677	0.002535
HOXC-AS	10.2875598	2.245390515	0.660699	3.398509	0.000678	0.002538
ISG15	2948.27987	1.232243641	0.362634	3.39804	0.000679	0.002541
M1AP	25.8852842	1.546610917	0.455235	3.397389	0.00068	0.002546
ACSBG1	167.86832	1.758488786	0.517898	3.395432	0.000685	0.002561
RBM20	55.6039299	-1.240364851	0.36551	-3.39352	0.00069	0.002578
ZNF711	317.971647	1.063980101	0.313546	3.393375	0.00069	0.002578
TENM1	120.576784	2.073292797	0.611029	3.393115	0.000691	0.002581
SHROOM	2893.35716	-1.211482383	0.357057	-3.39297	0.000691	0.002582
LINC0196	6.30019241	3.126010168	0.921418	3.392607	0.000692	0.002584
APOB	9.45125575	-1.784864686	0.526403	-3.39068	0.000697	0.002599
TTTY10	6.01364095	-1.228760515	0.362418	-3.39045	0.000698	0.0026
CBY2	19.1238723	1.361153164	0.401475	3.39038	0.000698	0.0026
RBM17P4	17.6793995	-1.025235439	0.302482	-3.38941	0.0007	0.002608
ZNF849P	1.85375484	2.288460154	0.675206	3.389279	0.000701	0.002609

IL1RAP	621.604035	1.007599935	0.297297	3.389206	0.000701	0.002609
HSPA5P1	4.12541118	1.29068603	0.380872	3.388768	0.000702	0.002613
PABPC5-A	1.25737549	-2.29188381	0.676407	-3.38832	0.000703	0.002616
LINC0031	5.49786263	-1.846960064	0.545165	-3.38789	0.000704	0.00262
CABP4	139.394037	1.186355527	0.350399	3.385726	0.00071	0.002637
TENT5D	1.33005185	2.643197775	0.780782	3.385319	0.000711	0.00264
CLDN1	4105.5688	1.064338527	0.314414	3.385151	0.000711	0.002641
LINC0252	3.75246838	-1.485007217	0.438741	-3.38471	0.000713	0.002645
PARP11-A	2.54256207	-2.087443197	0.616832	-3.38414	0.000714	0.002649
LINC0030	10.2933133	-1.206860467	0.356629	-3.38408	0.000714	0.002649
OR9K1P	9.89760814	2.836554204	0.838335	3.383559	0.000716	0.002653
PLB1	308.273888	1.001166874	0.296029	3.381991	0.00072	0.002667
SMCR5	0.80172213	-2.431348158	0.719009	-3.38153	0.000721	0.002671
TRBV5-5	1.88851143	-1.93353685	0.571981	-3.38042	0.000724	0.002681
AZU1	4.1376284	-1.110835424	0.328653	-3.37996	0.000725	0.002685
LINC0021	1.97107943	-1.782480576	0.527411	-3.37968	0.000726	0.002687
C1QC	2530.15092	-1.100162867	0.325632	-3.37854	0.000729	0.002697
RN7SKP2	2.12513928	2.099002287	0.621391	3.377911	0.00073	0.002702
OR7E121P	5.15850854	2.214222745	0.655528	3.377769	0.000731	0.002703
EIF4A1P1	0.52858919	-2.355526159	0.697382	-3.37767	0.000731	0.002703
FGFR3	6635.41617	1.368294283	0.405122	3.377486	0.000732	0.002705
PLOD2	2009.63934	1.090282654	0.322995	3.375537	0.000737	0.002721
PCDH11Y	18.1078352	-1.907421706	0.565597	-3.3724	0.000745	0.002751
RN7SL801	4.71283938	-1.424513605	0.42242	-3.37227	0.000746	0.002752
MSI1	68.7796768	1.41270806	0.419047	3.371236	0.000748	0.00276
SPI1	794.445796	-1.170434213	0.347215	-3.37092	0.000749	0.002762
ACSL6	34.1794436	-1.373874627	0.407686	-3.36994	0.000752	0.002769
C7orf57	4.62953468	1.375174997	0.40815	3.369289	0.000754	0.002775
LINC0194	4.60172818	-1.16279516	0.345165	-3.36881	0.000755	0.002779
TRAV24	1.9756254	-1.95248349	0.57974	-3.36786	0.000758	0.002788
SLC14A2-	3.13015381	-1.25138473	0.37157	-3.36783	0.000758	0.002788
COL4A6	1717.61472	-1.13155843	0.336169	-3.36604	0.000763	0.002806
DHCR24-I	4.69282697	1.248779337	0.371168	3.364455	0.000767	0.002819
LINC0288	2.34837162	3.589736129	1.066977	3.364399	0.000767	0.002819
RPL7P47	2.35197932	1.193024956	0.354661	3.36385	0.000769	0.002824
EPHA5-AS	1.01361374	-1.972647641	0.58657	-3.36302	0.000771	0.002832
TAB3-AS1	2.99427433	2.872185335	0.854244	3.362255	0.000773	0.002839
RNU6-979	2.30171036	-1.729069256	0.514291	-3.36205	0.000774	0.00284
TRIM54	10.3385477	1.746090785	0.519699	3.359811	0.00078	0.002861
AVPR1A	15.3130121	-1.275874097	0.379785	-3.35947	0.000781	0.002865
MIR31	1.15148308	-1.944829709	0.579096	-3.35839	0.000784	0.002875
SYCE3	5.69091793	1.198717823	0.356959	3.358139	0.000785	0.002877
RNU6-109	3.51309378	-1.362907861	0.405908	-3.35768	0.000786	0.002881
METTL21	2.79748957	1.375665572	0.409833	3.356646	0.000789	0.002891
LY86-AS1	2.67859991	-2.452395072	0.730815	-3.3557	0.000792	0.0029
APOL1	11913.2224	1.158137945	0.345213	3.354849	0.000794	0.002908
SRGN	4125.38558	-1.192678741	0.355655	-3.35347	0.000798	0.002922
GPR143	65.4870329	1.113479843	0.332122	3.352619	0.000801	0.002928
PDPN	1432.88903	-1.176824521	0.351025	-3.35253	0.000801	0.002928
NTRK1	20.1937757	-1.322399939	0.39451	-3.35201	0.000802	0.002933
ACP7	46.4437923	1.698451341	0.506737	3.351738	0.000803	0.002936
CLEC4M	6.07227591	-2.31823556	0.692012	-3.34999	0.000808	0.002953

TMC1	55.0697266	-1.15119971	0.343693	-3.3495	0.00081	0.002958
SLC5A12	101.916882	1.327298479	0.396346	3.348835	0.000812	0.002964
HRC	48.5503227	-1.243243731	0.371316	-3.34821	0.000813	0.00297
DRC1	6.1861712	1.448240776	0.432551	3.34814	0.000814	0.00297
NRK	93.3946818	-1.271761471	0.379851	-3.34805	0.000814	0.002971
RNF128	1801.6937	1.146814333	0.342667	3.346729	0.000818	0.002983
LILRB3	77.4340312	-1.33601001	0.39928	-3.34605	0.00082	0.002989
FOXD3-A	13.480606	-1.493062824	0.446414	-3.34457	0.000824	0.003002
WSCD1	53.5191356	-1.37176963	0.410171	-3.34438	0.000825	0.003003
KPNA4P1	13.569857	-1.007713	0.301414	-3.34328	0.000828	0.003013
HLA-DQA	410.619426	-1.486267619	0.444627	-3.34273	0.00083	0.003019
PPIAP72	11.7377484	-1.100635108	0.329314	-3.3422	0.000831	0.003024
SNX20	234.82702	-1.332351743	0.398669	-3.342	0.000832	0.003026
C19orf38	59.5439843	-1.106824479	0.331225	-3.34161	0.000833	0.003028
LINC0259	106.74245	-1.202042826	0.359728	-3.34153	0.000833	0.003028
LILRA6	79.3042494	-1.297570424	0.388359	-3.34116	0.000834	0.003032
MIR1207	1.08687526	1.703116218	0.509844	3.340468	0.000836	0.003038
NDST1-A	3.2753382	-1.108948184	0.33201	-3.3401	0.000837	0.003041
LINC0133	0.61887223	2.622461145	0.785187	3.339919	0.000838	0.003043
CNTNAP	62.9085185	-1.140955578	0.341682	-3.33923	0.00084	0.003049
RNU6-403	1.34541687	2.091202771	0.626352	3.338704	0.000842	0.003054
IDO1	696.024036	1.747635679	0.52346	3.338625	0.000842	0.003054
PKIA	1085.33431	1.104448604	0.330827	3.338445	0.000842	0.003055
IGHV3-43	248.000801	-2.400830914	0.719406	-3.33724	0.000846	0.003067
TRAJ2	5.33644426	-1.92951982	0.578279	-3.33666	0.000848	0.003073
HLA-DRA	15242.9417	-1.047159029	0.313873	-3.33625	0.000849	0.003076
FHAD1	278.66792	1.010016352	0.302829	3.335274	0.000852	0.003087
NDUFB1P	6.67879192	-1.037555735	0.311119	-3.33492	0.000853	0.00309
MIR656	1.5310909	-2.253683652	0.675936	-3.33417	0.000856	0.003097
CAPN11	21.8147686	-1.031143722	0.309762	-3.32882	0.000872	0.00315
RNU6-606	0.65596191	-1.937145691	0.582121	-3.32774	0.000876	0.003161
SERPINI2	3.16259744	-1.534320856	0.461161	-3.32708	0.000878	0.003166
SERBP1P	8.40180739	-1.056423014	0.317522	-3.32708	0.000878	0.003166
ADAMTS	288.051636	1.513915721	0.455127	3.326357	0.00088	0.003173
HS6ST3	18.4828731	1.906732465	0.573269	3.326067	0.000881	0.003175
TMEM59I	22.2111603	-1.443301407	0.434393	-3.32257	0.000892	0.003209
ULK4P1	2.00498975	-1.592831321	0.47947	-3.32207	0.000894	0.003213
ABCA9-A	4.15286977	2.775618796	0.835549	3.32191	0.000894	0.003215
ADH4	6.20058812	-1.772796458	0.533694	-3.32175	0.000895	0.003216
LARP1P1	0.93150779	2.364176285	0.712206	3.319514	0.000902	0.003239
CYSRT1	86.5242442	1.276320884	0.384604	3.318529	0.000905	0.003249
HDAC11-	2.59677686	1.381884272	0.416431	3.318396	0.000905	0.00325
HLA-DOB	64.4777176	-1.330286264	0.400976	-3.31762	0.000908	0.003258
GREB1L	30.9232243	2.002281158	0.603665	3.316874	0.00091	0.003266
LINC0243	1.56231709	3.820082621	1.151889	3.316363	0.000912	0.003271
TRAV39	2.41474508	-1.619616855	0.489089	-3.3115	0.000928	0.003323
LINC0084	37.3144681	1.729343089	0.522226	3.311485	0.000928	0.003323
SEMA6A	1083.69235	1.059306547	0.320093	3.309368	0.000935	0.003345
H1-2	1126.14174	1.112683228	0.336399	3.307631	0.000941	0.003364
LINC0099	58.3795032	-1.204667604	0.364266	-3.30711	0.000943	0.00337
TBR1	1.52426644	1.808273819	0.546835	3.306801	0.000944	0.003373
LINC0057	1.30980123	-1.762005257	0.533106	-3.30517	0.000949	0.003391

LCNL1	31.8523705	-1.626978944	0.492376	-3.30434	0.000952	0.003399
FCRL3	85.9819496	-1.79566551	0.543567	-3.30349	0.000955	0.003407
RUBCNL	45.1358515	-1.394715868	0.422252	-3.30304	0.000956	0.003411
IGHV1OR	7.64811367	-3.159938713	0.956845	-3.30246	0.000958	0.003417
HLA-V	64.6867548	1.514148017	0.458607	3.301627	0.000961	0.003427
CCT7P2	0.47333217	-2.028561058	0.614522	-3.30104	0.000963	0.003433
LYPD3	1730.61557	1.124354863	0.340655	3.300569	0.000965	0.003438
FAM124B	33.4304674	-1.018271153	0.308698	-3.2986	0.000972	0.003459
RNU6-59F	4.03693146	-1.104501037	0.334858	-3.29842	0.000972	0.003461
RNA5SP1:	1.51701225	-1.857702307	0.56342	-3.29719	0.000977	0.003474
CA2	522.858928	1.469454827	0.446044	3.294419	0.000986	0.003503
SPTBN4	66.6187015	-1.181206533	0.35873	-3.29275	0.000992	0.003523
SLC2A12	93.8928274	-1.141489481	0.346756	-3.29191	0.000995	0.003533
TEKT5	36.7522905	1.267104408	0.38507	3.290586	0.001	0.003547
KRT16P4	1.58681177	3.496950794	1.063075	3.289466	0.001004	0.003561
ZNF233	33.888812	1.047488544	0.318479	3.289031	0.001005	0.003566
RN7SL33:	3.47734305	1.316092622	0.400196	3.288623	0.001007	0.00357
ZNF80	14.19185	-1.526265011	0.464135	-3.2884	0.001008	0.003572
TIGD3	19.8184271	1.004133898	0.305369	3.288263	0.001008	0.003573
HSPE1P5	2.80952042	1.785454253	0.543034	3.287922	0.001009	0.003577
FPR1	440.947252	-1.342397061	0.408343	-3.28743	0.001011	0.003582
HOXA13	546.11469	-1.017880014	0.309661	-3.28707	0.001012	0.003586
CFAP157	39.2795718	1.338521792	0.407552	3.284295	0.001022	0.003618
LINC0213	1.18669844	-1.872226283	0.570164	-3.28366	0.001025	0.003624
PKD1L1	100.149992	1.197177525	0.364602	3.283515	0.001025	0.003625
SLC2A9-A	7.32151858	1.446034698	0.44041	3.283381	0.001026	0.003626
SLC4A9	15.4300531	1.084000446	0.3302	3.282856	0.001028	0.003631
RHOG2P	5.69731201	-1.122491986	0.341943	-3.28269	0.001028	0.003632
C4BPA	8.21913813	-2.100063267	0.639789	-3.28243	0.001029	0.003635
GTSCR1	0.81187203	-2.256295563	0.68751	-3.28184	0.001031	0.003642
TTC6	213.255064	1.016273742	0.309687	3.281618	0.001032	0.003644
PNCK	747.974571	1.483261958	0.452026	3.281365	0.001033	0.003646
LINC0190	4.19121332	2.970004303	0.905168	3.281164	0.001034	0.003647
ARSF	9.05718225	-2.107396712	0.642487	-3.28006	0.001038	0.003661
LILRB1	387.353857	-1.321504471	0.402951	-3.27957	0.00104	0.003666
CYBB	1860.2001	-1.152979127	0.351567	-3.27955	0.00104	0.003666
RPS23P6	10.0905572	-1.007590047	0.307292	-3.27894	0.001042	0.003673
CLDN9	27.8132927	1.461495427	0.44591	3.277558	0.001047	0.003689
VWDE	115.654338	1.578128772	0.481665	3.276405	0.001051	0.003702
C2CD4B	258.600603	-1.556880497	0.475213	-3.27617	0.001052	0.003704
MIR210H(	302.51342	1.115616581	0.340659	3.274877	0.001057	0.003719
NFAM1	247.850627	-1.20290345	0.367376	-3.27431	0.001059	0.003726
OR7E110F	2.1514452	2.051261476	0.626683	3.273204	0.001063	0.003739
LINC0277	2.74374184	-1.307729004	0.399549	-3.27301	0.001064	0.003741
TSACC	9.60892006	1.289454744	0.394058	3.272245	0.001067	0.00375
RPL21P54	3.59964417	1.572222848	0.480542	3.271772	0.001069	0.003755
UGT2B11	2.85679238	2.072548065	0.633466	3.27176	0.001069	0.003755
PLK5	7.04465034	-1.056417374	0.322952	-3.27113	0.001071	0.003762
HLA-DQB	179.722964	-1.301010145	0.397898	-3.26971	0.001077	0.003779
SAMSN1	582.917427	-1.242171387	0.379946	-3.26934	0.001078	0.003782
CRYBB3	13.5675497	1.104799841	0.337945	3.26917	0.001079	0.003784
WNT1	1.23329146	-1.667457253	0.510183	-3.26835	0.001082	0.003793

PIK3CG	171.531914	-1.009472576	0.308872	-3.26826	0.001082	0.003793
LINC0185	10.5877151	-1.760529119	0.538897	-3.26691	0.001087	0.003807
CLEC4C	11.3290303	-1.82585706	0.559035	-3.26609	0.00109	0.003818
CMKLR1	335.560202	-1.136726195	0.348048	-3.266	0.001091	0.003818
IGSF11	63.3993598	1.245445567	0.381373	3.265693	0.001092	0.003821
SLC5A8	2.46013291	-2.618944583	0.802102	-3.2651	0.001094	0.003828
EEF1A1P3	1.42320298	-1.529153845	0.468389	-3.26471	0.001096	0.003832
PAX9	177.011922	1.202443825	0.368676	3.261522	0.001108	0.003871
VASH2	178.901845	1.035512511	0.317525	3.261201	0.001109	0.003874
SEMA5A	2456.26332	1.053240016	0.323055	3.260255	0.001113	0.003885
HLA-J	40.4346082	-1.08405443	0.332641	-3.25893	0.001118	0.003901
LINC0066	19.7102619	1.467117233	0.450238	3.258538	0.00112	0.003906
EXD1	1.35064414	1.919120941	0.589009	3.258221	0.001121	0.003909
DUSP2	1572.56248	-1.108830209	0.340373	-3.25769	0.001123	0.003916
MRPL23-A	8.99237	3.049889474	0.936536	3.256562	0.001128	0.00393
GABRR1	15.5847299	1.966453166	0.60385	3.256528	0.001128	0.00393
FOXL2NE	13.9643668	2.602482133	0.799368	3.255674	0.001131	0.00394
MIR5581	3.89694567	1.212120025	0.372336	3.255444	0.001132	0.003943
CYP2AB1	6.66044813	-1.674482102	0.514563	-3.25418	0.001137	0.003958
RNU7-193	1.66751033	-1.192642689	0.366565	-3.25356	0.00114	0.003966
FAM187B	1.92510993	-1.196839076	0.367862	-3.2535	0.00114	0.003966
RN7SL127	2.78001886	-1.324622888	0.407258	-3.25254	0.001144	0.003978
RNU6-858	2.81831663	-1.415535699	0.435407	-3.25106	0.00115	0.003997
BECN2	0.86214416	2.391352497	0.73575	3.250223	0.001153	0.004008
PRG2	3.19775352	-1.376521184	0.423611	-3.24949	0.001156	0.004017
UBE2CP2	3.65967944	1.152984246	0.354921	3.248563	0.00116	0.004029
LRP1B	84.258679	1.886048926	0.580791	3.247378	0.001165	0.004044
OVOL1-A	3.19335607	1.505845117	0.463725	3.247283	0.001165	0.004045
PGF	470.023539	1.104999702	0.34029	3.247228	0.001165	0.004045
DPYD-IT1	2.87888616	-1.288321508	0.396834	-3.2465	0.001168	0.004054
COL4A2	9996.93791	-1.078034691	0.332088	-3.24623	0.001169	0.004057
MIR138-1	1.22844206	2.460025581	0.758665	3.24257	0.001185	0.004102
CALB1	293.168442	3.013032666	0.930093	3.239496	0.001197	0.004142
FAR2	265.026489	1.04193418	0.321656	3.239286	0.001198	0.004144
CPZ	38.9634132	1.42035253	0.438498	3.239132	0.001199	0.004146
GPR1	33.0313957	-1.587621876	0.490385	-3.2375	0.001206	0.004167
CKMT1B	222.831937	1.025932553	0.317068	3.235688	0.001213	0.004192
KIRREL1	1129.23096	-1.076766208	0.332893	-3.23457	0.001218	0.004207
CXXC4	116.699055	1.186477661	0.366912	3.233681	0.001222	0.004219
FOXD2	34.823681	1.341843844	0.414973	3.233568	0.001223	0.00422
RN7SL265	4.374484	-1.828835588	0.565697	-3.23289	0.001225	0.004228
RN7SL629	1.09688566	-1.971222276	0.609775	-3.2327	0.001226	0.00423
OR7E12P	0.85284782	-2.882394407	0.891785	-3.23216	0.001229	0.004237
RN7SL229	0.82701981	-2.842204199	0.879468	-3.23173	0.00123	0.004242
FUT3	567.518899	1.304512022	0.403661	3.231703	0.001231	0.004242
ZNF861P	4.50125171	2.391377416	0.740027	3.231472	0.001232	0.004245
RNU6-883	1.21474234	-1.797045251	0.556134	-3.23132	0.001232	0.004247
IGHV3-49	276.534859	-2.187647519	0.677339	-3.22977	0.001239	0.004267
C3AR1	339.538934	-1.049777572	0.325111	-3.22898	0.001242	0.004277
TRBV28	43.870257	-1.713664441	0.530738	-3.22883	0.001243	0.004278
MACROD	490.837016	1.275998619	0.395192	3.228806	0.001243	0.004278
TRBV21-1	2.23305097	-1.830821499	0.567081	-3.2285	0.001244	0.004281

MIR181B2	0.59284209	2.260062335	0.700337	3.227105	0.00125	0.004301
ST6GAL2	141.083147	1.5998783	0.495795	3.226895	0.001251	0.004303
ADAMTS	313.071907	-1.33382462	0.413347	-3.22689	0.001251	0.004303
GRIN1	20.173434	1.386127978	0.429663	3.226083	0.001255	0.004313
FRAS1	336.08978	1.224575004	0.379611	3.225872	0.001256	0.004315
LINC0105	1.65177731	-1.628010441	0.504678	-3.22584	0.001256	0.004315
OCM	7.71185802	-1.044114972	0.323745	-3.22511	0.001259	0.004325
TMC3	2.5705537	-2.6057137	0.808434	-3.22316	0.001268	0.004352
IGKV1-16	336.207458	-1.926397499	0.597756	-3.22271	0.00127	0.004358
PRB3	15.2656446	1.174157563	0.36435	3.222612	0.00127	0.004359
MT1P1	2.5371405	1.335832043	0.414653	3.221563	0.001275	0.004373
NXPE2	3.29402416	-1.419273432	0.44061	-3.22116	0.001277	0.004377
SERPIND1	31.0598708	-1.621946527	0.503646	-3.22041	0.00128	0.004387
SCT	10.8668565	-1.096867994	0.340616	-3.22025	0.001281	0.004389
PURPL	12.9573858	1.913825315	0.594804	3.21757	0.001293	0.004426
MTDHP1	6.16083563	1.454844071	0.452317	3.216426	0.001298	0.004441
LRRIQ4	19.2299911	1.878094542	0.583912	3.216399	0.001298	0.004441
EPDR1	257.328672	-1.136792868	0.353437	-3.2164	0.001298	0.004441
CASS4	64.5170717	-1.061166631	0.329959	-3.21605	0.0013	0.004445
GAPDHP6	3.63219045	1.071839492	0.333375	3.215116	0.001304	0.004458
SMTNL2	56.3890464	-1.190036237	0.370172	-3.21482	0.001305	0.004462
IGLV10-54	53.2367404	-1.926225566	0.599181	-3.21476	0.001306	0.004462
GDPD5	304.494712	-1.00695666	0.313523	-3.21175	0.001319	0.004503
SNORD11	1.1276574	-2.208506569	0.687723	-3.21133	0.001321	0.004509
OR14L1P	0.61260188	-1.874658434	0.583794	-3.21116	0.001322	0.00451
SLC13A2	4.58094747	-2.016845336	0.628146	-3.21079	0.001324	0.004515
ANKRD20	15.4420342	-1.467635544	0.45721	-3.20998	0.001327	0.004528
MSC-AS1	162.202266	-1.289531879	0.402059	-3.20732	0.00134	0.004565
APOL4	1444.93902	1.091557404	0.340361	3.207058	0.001341	0.004568
NLRC4	48.0471317	-1.171959615	0.365456	-3.20684	0.001342	0.004571
RPS2P4	13.466069	1.129284609	0.352193	3.206437	0.001344	0.004576
RASGRF2	7.98095999	-1.342256338	0.4187	-3.20577	0.001347	0.004585
KIF25	6.55603437	-1.143424467	0.356962	-3.20321	0.001359	0.004622
ATOH7	6.03634255	1.423979647	0.444656	3.202429	0.001363	0.004633
SLC16A4	254.856282	-1.237271649	0.386545	-3.20084	0.00137	0.004656
SPIN2P1	1.96944767	1.419984831	0.443636	3.20079	0.001371	0.004656
LINC0160	26.9203632	1.178992873	0.368646	3.198171	0.001383	0.004696
DOCK2	670.501921	-1.239503458	0.387861	-3.19574	0.001395	0.004731
ARHGAP9	524.064178	-1.248381948	0.390646	-3.19568	0.001395	0.004731
PGK1P1	3.40046223	1.095199466	0.342714	3.195666	0.001395	0.004731
IMPA1P1	2.28046815	1.701296307	0.532437	3.195304	0.001397	0.004736
FAM47C	0.89462841	2.107920644	0.660041	3.193619	0.001405	0.00476
ZNF732	9.92297981	1.477063082	0.462543	3.193355	0.001406	0.004763
LINC0232	1.51562334	-2.100209018	0.657749	-3.19303	0.001408	0.004767
ATP10A	184.454113	-1.179923157	0.369538	-3.19297	0.001408	0.004768
REEP2	112.358938	-1.235229183	0.387068	-3.19125	0.001417	0.004791
MTND6P1	0.67002497	-1.962999263	0.61531	-3.19026	0.001421	0.004805
LILRB2	439.85851	-1.280654815	0.401555	-3.18924	0.001426	0.00482
TRAJ43	0.75222036	-2.074706688	0.650675	-3.18854	0.00143	0.004831
PAX8	347.868937	1.709504523	0.536501	3.186393	0.001441	0.004864
RIPPLY3	92.4649813	1.19441962	0.37491	3.18588	0.001443	0.004871
RPL10P2	0.831262	1.858989594	0.583632	3.185211	0.001446	0.00488

TRMT112	1.2196316	1.816204272	0.570382	3.18419	0.001452	0.004897
GUCY2D	13.8163799	1.565247824	0.491725	3.183175	0.001457	0.004914
ATRNL1	36.2806576	-1.059813021	0.333047	-3.18218	0.001462	0.004927
MEPE	1.58234257	2.321357995	0.72956	3.181862	0.001463	0.004931
LINC0280	3.41844512	1.474351551	0.463477	3.181065	0.001467	0.004943
ITGA4	401.651796	-1.075970036	0.33827	-3.18081	0.001469	0.004946
PSTPIP1	275.58147	-1.08128539	0.339955	-3.18067	0.001469	0.004948
ASIC1	106.000516	-1.056942269	0.332574	-3.17807	0.001483	0.004988
GPR21	3.14426635	-1.13187515	0.356314	-3.17662	0.00149	0.005011
KIAA2012	2.77060078	-1.541153473	0.485175	-3.17649	0.001491	0.005013
CKS1BP7	2.45537258	1.188630394	0.374229	3.176213	0.001492	0.005017
TRAJ12	0.81831891	-2.081203167	0.655477	-3.1751	0.001498	0.005032
LINC0256	23.4309375	1.073350156	0.338055	3.175078	0.001498	0.005032
SAMD9	1270.25006	1.021895361	0.321852	3.175051	0.001498	0.005032
GRIP2	91.8204481	-1.11988589	0.352825	-3.17405	0.001503	0.005045
BTF3P6	3.98051765	1.196636964	0.377012	3.174002	0.001504	0.005046
MYCL	2295.15359	1.211372982	0.381761	3.17312	0.001508	0.00506
NUCB1-A	5.77830727	-1.128190066	0.355715	-3.17161	0.001516	0.005081
HAGLROS	47.112558	1.000952309	0.315854	3.169033	0.001529	0.005123
DLX6-AS	203.896189	1.383692892	0.436638	3.168968	0.00153	0.005123
LINC0244	0.93358077	3.180253872	1.003878	3.167968	0.001535	0.00514
FOXO6-A	3.37054569	-1.592515615	0.50274	-3.16767	0.001537	0.005143
TEX38	2.4738715	1.391078759	0.439188	3.167391	0.001538	0.005147
FAM187B	0.58984929	-1.719353778	0.542997	-3.16642	0.001543	0.005162
GLTPD2	5.48515053	1.831518061	0.578726	3.164743	0.001552	0.00519
RPL37P2	58.0794248	-1.266323098	0.400146	-3.16465	0.001553	0.005191
HSPD1P5	0.67655098	1.833159913	0.579325	3.164305	0.001555	0.005196
MIR4637	0.83337189	-2.039105981	0.644419	-3.16425	0.001555	0.005196
LTB	528.83536	-1.459099978	0.46113	-3.16418	0.001555	0.005197
MCF2L-A	66.2938796	1.101849978	0.348422	3.1624	0.001565	0.005225
CEBPA-D	7.9849892	1.224988874	0.387415	3.161958	0.001567	0.005231
EPHA7	311.482065	-1.356608128	0.429084	-3.16164	0.001569	0.005236
H2AC13	12.5392988	1.339251363	0.423624	3.161412	0.00157	0.005239
IQGAP2	557.039463	-1.050498291	0.332305	-3.16125	0.001571	0.00524
RN7SL368	36.2670576	-1.245180692	0.393912	-3.16106	0.001572	0.005242
CLIC4P1	13.1834718	-1.043619874	0.33026	-3.16	0.001578	0.005256
RNU7-30F	0.97993566	-1.577795639	0.499857	-3.1565	0.001597	0.005313
GJC2	76.0371053	-1.092493352	0.346134	-3.15627	0.001598	0.005315
L3MBTL4	99.9198084	-1.044649328	0.331077	-3.15531	0.001603	0.00533
LINC0184	6.97210031	-1.376625967	0.436831	-3.15139	0.001625	0.005393
JAK3	1129.64088	-1.148734637	0.364632	-3.1504	0.00163	0.005409
DEPDC1-/	0.95670321	2.216671907	0.703763	3.149741	0.001634	0.00542
SLA2	120.48795	-1.223407064	0.388451	-3.14945	0.001636	0.005425
STAB2	32.7744497	-2.081798771	0.661287	-3.1481	0.001643	0.005447
RP1L1	18.8137709	1.440302589	0.457663	3.147083	0.001649	0.005462
TRAV38-2	3.60072665	-1.498353456	0.476135	-3.14691	0.00165	0.005464
CSRP3-AS	1.45650822	1.716883793	0.54577	3.145803	0.001656	0.005482
SLC9A2	592.657152	1.406526776	0.447366	3.14402	0.001666	0.005512
GPR37L1	8.95689031	1.333023632	0.42409	3.143254	0.001671	0.005524
C16orf54	228.179121	-1.270494281	0.404435	-3.1414	0.001681	0.005555
P2RX7	88.737422	-1.023444905	0.325796	-3.14137	0.001682	0.005555
ZRANB2-/	1.82904985	-1.171960656	0.373178	-3.14048	0.001687	0.005571

PLAG1	383.926422	1.025408098	0.326574	3.139893	0.00169	0.00558
IL23R	8.92722407	-1.317940801	0.419774	-3.13964	0.001692	0.005584
RAB44	31.1732507	-1.141203404	0.363489	-3.13958	0.001692	0.005584
TRBV24-1	5.39434099	-1.86542087	0.594369	-3.13849	0.001698	0.005603
TNFRSF1'	39.8659563	-1.709831899	0.545073	-3.13689	0.001708	0.005627
ATP6V1B	57.5395004	1.261113436	0.40216	3.13585	0.001714	0.005643
GRAPL	5.40529467	-1.042669196	0.332548	-3.1354	0.001716	0.005651
KCNA6	3.62130782	-1.577029854	0.502979	-3.13538	0.001716	0.005651
TMEM97I	2.07324109	1.587666945	0.506419	3.135086	0.001718	0.005655
SNORD11	0.60501059	-2.068977221	0.659977	-3.13492	0.001719	0.005657
CASC9	250.420815	1.718892477	0.548368	3.134563	0.001721	0.005663
IGHV3OR	9.57514613	-2.247605635	0.717193	-3.13389	0.001725	0.005674
AQP12B	3.00785264	4.190430063	1.337475	3.13309	0.00173	0.005687
RN7SL12I	2.47139718	1.402835478	0.447773	3.13292	0.001731	0.005689
RPSAP29	1.29522237	2.291993411	0.731759	3.13217	0.001735	0.005702
FUT7	27.0381981	-1.381817716	0.441207	-3.1319	0.001737	0.005706
SHANK1	27.3084174	-1.31291284	0.419236	-3.13168	0.001738	0.005709
NDUFB4P	11.0637116	1.058094565	0.337891	3.131469	0.001739	0.005711
SNORD11	0.59859386	-2.559292696	0.817453	-3.13081	0.001743	0.005719
HIKESHII	1.32056347	-1.387210924	0.443189	-3.13007	0.001748	0.005732
TRAV4	12.9483852	-1.537251712	0.49125	-3.12927	0.001752	0.005747
RNU7-77F	4.1256571	-1.206010834	0.385619	-3.12746	0.001763	0.005779
NLRC3	274.800061	-1.281089002	0.409983	-3.12473	0.00178	0.005827
RPS3AP31	0.98391861	-2.14216405	0.685663	-3.12422	0.001783	0.005835
FAM153A	38.9816241	-1.36710962	0.437585	-3.12421	0.001783	0.005835
PCBP3	46.5195803	-1.187022563	0.379953	-3.12413	0.001783	0.005836
LINC0224	3.7851175	1.899419894	0.608175	3.123148	0.001789	0.005851
RNU7-43F	0.7575899	-2.518278005	0.806338	-3.1231	0.00179	0.005851
KBTBD11	1.83503954	-1.616647241	0.517908	-3.1215	0.001799	0.005876
IGKV2OR	5.8335833	-3.791382194	1.214708	-3.12123	0.001801	0.005881
RPL23AP:	0.72111651	1.918706104	0.614747	3.121131	0.001802	0.005881
LINC0118	5.38470935	1.340950802	0.429681	3.120806	0.001804	0.005887
NKAIN4	8.40921091	1.184949931	0.379708	3.120685	0.001804	0.005887
SERPINA:	877.180702	-1.753404403	0.562111	-3.11932	0.001813	0.005912
C6orf118	2.21367558	1.682653166	0.539449	3.119209	0.001813	0.005914
LINC0227	1.04675728	-2.509182862	0.804591	-3.11858	0.001817	0.005925
CEACAM	3.71129459	-1.996843474	0.64035	-3.11836	0.001819	0.005929
NRG2	123.643368	-1.298380963	0.416381	-3.11825	0.001819	0.00593
PPIAP77	4.30770992	1.501939028	0.481707	3.11795	0.001821	0.005935
HBG1	0.72879682	-1.955308829	0.627204	-3.1175	0.001824	0.005943
DLG1-AS	6.3620657	1.166986215	0.374421	3.116777	0.001828	0.005955
SDC2	1931.79953	-1.024715016	0.328815	-3.11638	0.001831	0.00596
LINC0273	2.7818543	-1.409310375	0.452232	-3.11635	0.001831	0.00596
LAPTM4I	1.51120957	1.678473185	0.538625	3.11622	0.001832	0.005962
MIR378D:	1.1131552	1.44118953	0.462588	3.115492	0.001836	0.005975
RPL11P4	0.67630715	-2.540091184	0.815507	-3.11474	0.001841	0.005989
RN7SL44:	0.64747438	-2.04693741	0.657247	-3.11441	0.001843	0.005994
RN7SKP1:	2.2426467	-1.218697093	0.391417	-3.11355	0.001849	0.00601
ZP1	11.9306178	1.281514347	0.411841	3.111674	0.00186	0.006046
IGLC6	14.4895201	-2.269184493	0.729599	-3.11018	0.00187	0.006074
IGLV1-36	49.2934036	-2.206161296	0.709433	-3.10975	0.001872	0.006082
RPL7P51	1.93181989	-1.946688972	0.626039	-3.10953	0.001874	0.006085

RPSAP13	2.32675245	1.207800052	0.388464	3.109167	0.001876	0.006092
KIAA1755	201.756113	-1.520393757	0.489028	-3.10901	0.001877	0.006093
SETP11	1.35762668	-1.626186582	0.523294	-3.1076	0.001886	0.006118
TMEM132	24.901897	-1.030462074	0.33175	-3.10614	0.001895	0.006146
C5orf52	1.95845928	-1.951236641	0.628261	-3.10578	0.001898	0.006152
RNY4	1.65525538	-2.807148729	0.90404	-3.10511	0.001902	0.006164
SUGCT-A	4.88854447	2.194129996	0.70663	3.105062	0.001902	0.006164
DNAJB13	72.2043809	1.587468478	0.511358	3.104419	0.001907	0.006175
MNX1-AS	3.63724661	2.45208243	0.789953	3.104088	0.001909	0.00618
EIF1P3	2.81864505	-1.123774639	0.362073	-3.10372	0.001911	0.006186
GPR132	169.294885	-1.235149703	0.398045	-3.10304	0.001915	0.006199
STAT4	235.151781	-1.025217949	0.330724	-3.09992	0.001936	0.006256
C1QTNF1	601.766352	-1.100627668	0.355196	-3.09865	0.001944	0.006279
MIR33B	1.57878299	2.167728554	0.699768	3.097781	0.00195	0.006295
RPL21P35	1.22278345	-1.83017359	0.590835	-3.0976	0.001951	0.006296
LINC0099	28.7019618	1.147296502	0.370388	3.097554	0.001951	0.006296
LINC0197	19.0571842	1.125347136	0.363314	3.097455	0.001952	0.006297
TNFRSF10	106.360063	-1.00331013	0.32393	-3.0973	0.001953	0.0063
PRDX3P1	1.27471687	-1.939123948	0.626115	-3.09707	0.001954	0.006303
RN7SL817	0.77668726	-1.61134437	0.520478	-3.09589	0.001962	0.006323
ADGRG5	91.5547932	-1.399388984	0.45249	-3.09264	0.001984	0.006385
ATP2A3	1723.29536	-1.009078983	0.326573	-3.0899	0.002002	0.00644
NKX6-1	24.3080232	2.231746285	0.722539	3.088754	0.00201	0.006464
SHH	1617.45672	-1.362685126	0.441571	-3.086	0.002029	0.006517
FRMPD3	29.8481201	-1.109521714	0.359688	-3.08468	0.002038	0.006543
IL12RB1	149.428647	-1.205731257	0.391227	-3.08192	0.002057	0.006594
IDI2-AS1	13.3091641	1.259949992	0.408966	3.080819	0.002064	0.006616
NOS3	470.268782	-1.064579723	0.345566	-3.08069	0.002065	0.006619
RNU7-119	0.94037772	-3.272695995	1.062398	-3.08048	0.002067	0.006622
MARCO	130.578516	-1.597230281	0.518526	-3.08033	0.002068	0.006625
CSF3R	1093.81032	-1.148864183	0.372997	-3.08009	0.002069	0.006629
TDRD6	40.7135651	-1.030553246	0.334605	-3.07991	0.002071	0.006632
CACNB4	151.696736	-1.174595749	0.381583	-3.07822	0.002082	0.006666
LINC0016	0.89140737	2.589155728	0.841347	3.077392	0.002088	0.006683
ADGRV1	407.880746	1.020670778	0.331739	3.076725	0.002093	0.006696
TRIM31	3091.82769	-1.412702376	0.459321	-3.07564	0.002101	0.006719
RN7SL385	2.24259025	-1.37244116	0.446305	-3.07512	0.002104	0.006727
RNU6-140	3.74882526	2.846083562	0.925557	3.074994	0.002105	0.006729
POM121L	1.63951204	3.651624642	1.187672	3.074608	0.002108	0.006736
ANKRD34	10.2180806	1.714132542	0.557599	3.074131	0.002111	0.006745
RCCD1-A	2.62534789	1.023674092	0.333025	3.07387	0.002113	0.00675
PXDN	2235.0396	-1.114514008	0.36262	-3.0735	0.002116	0.006757
RNU7-61P	1.04053708	2.983327269	0.970742	3.073246	0.002117	0.006762
SNORD11	0.75710065	-2.94968318	0.960242	-3.07181	0.002128	0.006789
RGS11	251.537057	-1.296918701	0.422288	-3.07117	0.002132	0.006803
APOA1-A	0.99852689	-1.807954091	0.588799	-3.07058	0.002136	0.006815
ANGPT1	157.89222	-1.035629188	0.337322	-3.07015	0.00214	0.006823
AKR1E2	99.8720264	1.029916395	0.335501	3.069791	0.002142	0.006827
SNORD11	1.71526568	-1.921988589	0.62614	-3.06958	0.002144	0.006829
IGFBP5	16526.6299	-1.325260848	0.431838	-3.06889	0.002149	0.006843
HSP90AA	2.55005129	1.031311929	0.336097	3.068494	0.002151	0.006849
TRBV7-6	2.82174728	-1.850850566	0.603341	-3.06767	0.002157	0.006866

CXCR6	163.358721	-1.277409837	0.416441	-3.06745	0.002159	0.00687
CPNE7	233.917638	1.413266725	0.460734	3.067427	0.002159	0.00687
CD8A	439.581874	-1.216181457	0.396574	-3.06672	0.002164	0.006884
RPL34P20	2.10878637	-1.255616594	0.40955	-3.06584	0.002171	0.006901
IGHD	247.484303	-2.023832579	0.660242	-3.06529	0.002175	0.00691
PCDH10	92.1699828	-1.616639309	0.527571	-3.06431	0.002182	0.006932
CACNA1(	15.0728128	-1.075814636	0.351132	-3.06385	0.002185	0.00694
NKX2-8	16.4352179	2.2067601	0.720321	3.063578	0.002187	0.006945
LINC0031	1.19718694	-1.53460509	0.501186	-3.06195	0.002199	0.006979
SEC14L5	18.3993822	-1.014072121	0.331273	-3.06114	0.002205	0.006994
XKR9	28.5839483	1.181883443	0.386139	3.060775	0.002208	0.007001
GPR142	5.78013945	-1.66666625	0.544556	-3.06059	0.002209	0.007005
LINC0183	13.1160322	1.423611752	0.465264	3.059791	0.002215	0.007021
IGHV1-24	481.33598	-2.459432269	0.803902	-3.05937	0.002218	0.007028
IGHV5-51	819.542367	-2.030848671	0.663908	-3.05893	0.002221	0.007037
LDHAP3	2.47990661	1.251255058	0.409175	3.057992	0.002228	0.007052
LINC0217	0.87096368	-3.303817407	1.081578	-3.05463	0.002253	0.007122
H3C14	4.22575688	1.659315934	0.543548	3.052752	0.002268	0.007166
LINC0270	2.78183359	2.454064242	0.804041	3.052163	0.002272	0.007179
NPIPA3	4.65542578	1.064741087	0.348953	3.051249	0.002279	0.007197
TRAJ35	0.68339559	-2.362963329	0.774667	-3.0503	0.002286	0.007217
DMKN	1476.62409	1.049510829	0.344105	3.049973	0.002289	0.007222
PDCD1	68.6100582	-1.401395461	0.45989	-3.04724	0.00231	0.007286
NUDT19P	17.1261336	-1.049087843	0.344373	-3.04637	0.002316	0.007304
GASK1A	92.2604923	-1.083728833	0.35585	-3.04546	0.002323	0.00732
DNAH17-.	3.75351183	2.330660546	0.765365	3.045161	0.002326	0.007326
IL1RL1	156.876838	-1.203540819	0.395375	-3.04405	0.002334	0.007351
LINC0187	39.5451957	-1.272732799	0.418172	-3.04356	0.002338	0.007361
CRACD	131.44974	1.001538944	0.329131	3.042978	0.002342	0.007374
TXNP4	8.33134584	1.12437463	0.369603	3.042117	0.002349	0.007394
HAUS6P3	2.01213512	1.186337465	0.390124	3.040926	0.002359	0.00742
LINC0239	4.7791538	-1.413341856	0.464796	-3.04078	0.00236	0.007422
LINC0209	0.71138274	-2.698462336	0.887984	-3.03886	0.002375	0.007462
BPIFB4	6.16411292	2.340911454	0.770727	3.037278	0.002387	0.0075
PDGFRB	2462.86154	-1.075992775	0.354427	-3.03587	0.002398	0.00753
AGT	179.89734	-1.323183255	0.435957	-3.03512	0.002404	0.007544
SEC14L4	23.5661208	1.374165707	0.452786	3.034913	0.002406	0.007548
CBX1P3	0.4309466	-1.800499133	0.593356	-3.03443	0.00241	0.007559
BNIP3P41	0.86896033	-2.769478245	0.913085	-3.0331	0.002421	0.007586
RPL31P15	2.69414346	1.221150924	0.402724	3.03223	0.002428	0.007603
GABBR2	213.614703	1.728289479	0.570229	3.030871	0.002438	0.007634
BICC1	339.943812	-1.093487224	0.3608	-3.03073	0.00244	0.007637
ZSCAN5D	3.48030869	3.027226993	0.999085	3.029998	0.002446	0.007653
TRAV19	12.2653982	-1.350581008	0.445739	-3.02998	0.002446	0.007653
KRT18P2(	3.54174368	-1.077078405	0.35558	-3.02908	0.002453	0.007671
OVOL3	1.54196319	1.37318706	0.453349	3.028983	0.002454	0.007672
LINC0176	45.9443636	1.783869252	0.589433	3.026417	0.002475	0.007729
MIR328	1.14664863	-1.739660102	0.57516	-3.02465	0.002489	0.00777
MTND1P3	5.19253435	1.010726911	0.33435	3.022963	0.002503	0.00781
IGHV3-73	92.4598714	-1.92447204	0.637007	-3.02112	0.002518	0.007854
LINC0228	1.84446461	-1.895494741	0.627445	-3.02097	0.00252	0.007857
IYD	51.9611068	1.423490811	0.471397	3.019728	0.00253	0.007883

GSDMC	238.940351	1.389444195	0.460145	3.019577	0.002531	0.007885
PRXL2AP	1.2057572	-1.751460582	0.580173	-3.01886	0.002537	0.007901
KCNK17	32.6309591	-1.21951209	0.40405	-3.01822	0.002543	0.007917
LINC0196	2.68618767	-1.150201304	0.381263	-3.01681	0.002554	0.00795
SEC1P	2.14610404	-1.252767952	0.415464	-3.01534	0.002567	0.007985
TPI1P3	0.75743222	1.701777869	0.56447	3.014823	0.002571	0.007997
UROC1	0.93515179	-1.898451071	0.629935	-3.01372	0.002581	0.008021
P3H2-AS1	2.3662659	-1.305268948	0.433145	-3.01347	0.002583	0.008024
RN7SL53	5.97336892	1.138156717	0.377921	3.011628	0.002599	0.008068
IGLV5-45	42.4697798	-1.99923406	0.664007	-3.01086	0.002605	0.008085
MIR4539	1.8111244	-2.422012978	0.804441	-3.0108	0.002606	0.008086
TASL	97.8533774	-1.146723322	0.380907	-3.01051	0.002608	0.008093
PRDX1P1	2.01164042	2.033676302	0.675605	3.010154	0.002611	0.0081
HTR4	1.92077882	-1.404183188	0.466502	-3.01003	0.002612	0.008102
LINC0056	8.48729614	-1.184680861	0.39358	-3.01001	0.002612	0.008102
LINC0241	2.63124145	-1.624534349	0.539922	-3.00883	0.002623	0.008131
CFI	939.568359	-1.056784125	0.35128	-3.00838	0.002626	0.008142
LINC0187	60.8036004	1.215517897	0.404113	3.007866	0.002631	0.008153
SLCO4A1	4.26998005	2.34654797	0.780139	3.007859	0.002631	0.008153
HLA-DQB	760.39438	-1.295563571	0.43083	-3.00714	0.002637	0.008168
KRT12	17.0277194	2.067439724	0.68844	3.003078	0.002673	0.00826
ROCK1P1	32.3399741	1.003513372	0.334259	3.002205	0.00268	0.008281
ZIC2	42.6264849	2.201521625	0.733747	3.000382	0.002696	0.008327
RPL26P3	3.4804297	3.526723508	1.175451	3.000314	0.002697	0.008328
GALR2	5.02027857	1.212203067	0.404119	2.999621	0.002703	0.008345
SPECC1L	3.25827281	-1.283328036	0.42789	-2.9992	0.002707	0.008356
PTPN22	255.876933	-1.178666022	0.393049	-2.99878	0.002711	0.008364
ZPLD2P	14.0910701	1.0333312	0.344694	2.997826	0.002719	0.008385
CD247	238.497879	-1.13885994	0.379946	-2.99743	0.002723	0.008394
IGFL2-AS	96.3366252	2.291212173	0.765349	2.993684	0.002756	0.008491
CYLD-AS	12.821101	-1.075540927	0.359291	-2.99351	0.002758	0.008495
HLA-DRB	4740.90344	-1.222773118	0.408594	-2.99264	0.002766	0.008517
KIF5C	789.161807	1.193796983	0.399052	2.991581	0.002775	0.008542
RN7SKP2	1.81690925	-1.154671831	0.385994	-2.99142	0.002777	0.008544
PLA2G4C	270.839757	-1.116992313	0.373514	-2.9905	0.002785	0.008567
BARX2	103.119508	1.553123569	0.519628	2.988916	0.0028	0.008605
PLD5	3.71288046	-2.300983363	0.769846	-2.98889	0.0028	0.008605
MYBL2	844.259622	1.272407449	0.42581	2.988206	0.002806	0.008622
RGPD4	3.6505736	1.089340647	0.364717	2.986807	0.002819	0.008657
PCDHB6	50.5134663	1.173066716	0.392845	2.986082	0.002826	0.008673
LINC0195	3.18273761	2.786975055	0.933592	2.985219	0.002834	0.008695
IGHV3-66	34.5958497	-2.30226753	0.771499	-2.98415	0.002844	0.008722
RPL7L1P8	5.67322648	1.117042982	0.374329	2.984124	0.002844	0.008722
ADAM29	1.30014973	-1.797181298	0.602352	-2.98361	0.002849	0.008735
CAMKV	2.07121673	2.067347433	0.693047	2.982985	0.002855	0.00875
ZAR1	1.16966566	1.905138676	0.638702	2.98283	0.002856	0.008754
DUOX2	4516.96065	-1.393625308	0.467328	-2.98212	0.002863	0.008771
OR2B6	9.21382865	1.953768413	0.655566	2.980277	0.00288	0.008813
ARL14EP	1.03190839	1.837616588	0.616683	2.979838	0.002884	0.008822
CNTNAP2	78.6399543	1.933377876	0.649327	2.97751	0.002906	0.008879
TNFAIP6	393.914678	-1.432281967	0.481053	-2.97739	0.002907	0.008882
ALK	3.36582396	-1.514024035	0.508701	-2.97626	0.002918	0.008911

LY6G6C	16.1832662	1.414195674	0.475305	2.975344	0.002927	0.008934
DOC2A	89.474612	1.24690446	0.419093	2.975249	0.002928	0.008936
VAX2	17.6268755	1.195285048	0.401753	2.975175	0.002928	0.008937
ARSI	105.508672	-1.282131971	0.431064	-2.97434	0.002936	0.008958
OPTC	1.03626059	-2.655592014	0.893018	-2.97373	0.002942	0.008975
KRT17P3	3.93966577	1.623074679	0.54582	2.973645	0.002943	0.008976
IGHV4-61	225.46473	-2.263661437	0.761256	-2.97359	0.002943	0.008977
IGLV6-57	379.878174	-1.943923711	0.653774	-2.97339	0.002945	0.008981
KCTD9P1	1.42838135	-1.323952777	0.44535	-2.97284	0.002951	0.008993
ANKRD62	9.94263576	2.020928618	0.679897	2.972403	0.002955	0.009005
RN7SL842	0.75545067	-2.797687663	0.94124	-2.97234	0.002955	0.009005
LINC0232	3.80087036	-1.467451912	0.494329	-2.96857	0.002992	0.00911
LINC0217	8.28642603	2.636029998	0.888307	2.967475	0.003003	0.009136
LINC0164	0.79643705	-2.484137897	0.83719	-2.96723	0.003005	0.009143
SNORD11	0.59841673	-2.485553445	0.837849	-2.96659	0.003011	0.009156
DOK6	209.927622	-1.313539505	0.442826	-2.96627	0.003014	0.009162
BPIFA2	5.04605947	1.918648383	0.646964	2.965616	0.003021	0.009178
GRHL3	1778.72861	1.06889672	0.360529	2.964801	0.003029	0.009196
GOLGA8U	1.13706994	1.415429642	0.477468	2.96445	0.003032	0.009202
SNRPCP1	0.50516378	-1.584020911	0.534434	-2.96392	0.003037	0.009215
OR7E13P	0.80935618	-2.104895954	0.710603	-2.96213	0.003055	0.009256
ABCA13	113.759963	1.47820722	0.499087	2.961823	0.003058	0.009264
TRAV13-2	7.83414961	-1.385828414	0.467931	-2.96161	0.00306	0.009268
RPSAP45	1.78762454	1.195567483	0.40383	2.960573	0.003071	0.009297
SMKR1	16.2139256	1.19636051	0.404271	2.959304	0.003083	0.009331
TRBV7-4	0.7001271	-1.762925378	0.595726	-2.95929	0.003084	0.009331
MIR544B	3.26617078	1.059142913	0.357905	2.959285	0.003084	0.009331
KDM3AP1	3.0279128	-1.26425894	0.427379	-2.95817	0.003095	0.009361
FAM155B	60.5396771	1.117083775	0.377657	2.957935	0.003097	0.009367
MIR3065	0.76181978	-1.567059604	0.529942	-2.95704	0.003106	0.009389
PSMD10P	1.29593141	1.974451539	0.667738	2.956926	0.003107	0.00939
RHBG	200.728386	1.450772012	0.490638	2.956907	0.003107	0.00939
LINC0199	1.24440539	-3.239893485	1.096455	-2.95488	0.003128	0.009446
FPR2	81.2860916	-1.356296263	0.45919	-2.95367	0.00314	0.009481
B3GAT1	251.573321	1.566311757	0.530525	2.95238	0.003153	0.009518
GLT1D1	35.7566627	-1.379872739	0.467462	-2.95184	0.003159	0.00953
KLHL13	265.804369	-1.048535755	0.355401	-2.95029	0.003175	0.009571
RPH3A	5.0247059	-1.463788072	0.496194	-2.95003	0.003177	0.009578
FAM90A1	18.2146472	1.087567666	0.36876	2.949254	0.003185	0.009596
LINC0086	138.542021	-1.359987707	0.461167	-2.94901	0.003188	0.009601
IGHV3-48	576.040965	-1.997994032	0.678309	-2.94555	0.003224	0.009694
TRGV8	2.96955307	-1.496308729	0.508187	-2.94441	0.003236	0.009727
COL6A3	14758.3976	-1.092620655	0.371103	-2.94425	0.003237	0.009731
GH1	1.00169959	-1.446080486	0.491377	-2.94292	0.003251	0.009768
MRPL36P	0.78541541	1.692141738	0.575042	2.942639	0.003254	0.009776
IGKV1-9	214.281544	-1.745155792	0.593328	-2.9413	0.003268	0.009814
CROCCP5	0.94770727	1.745141556	0.593451	2.940668	0.003275	0.009832
ATP5MGL	0.70334835	-1.877368881	0.638781	-2.93899	0.003293	0.009882
BRS3	2.64236934	2.265727708	0.77177	2.935754	0.003327	0.009977
IGKV3-11	1306.49297	-1.832469039	0.624354	-2.93498	0.003336	0.01
LINC0191	0.52364222	-2.735722738	0.932217	-2.93464	0.003339	0.010007
MRPL40P	6.82914961	1.222344243	0.416812	2.932603	0.003361	0.010064

LINC0121	44.7886302	1.405433853	0.479319	2.932144	0.003366	0.010077
RBM22P2	15.2939972	-1.013612179	0.345859	-2.93071	0.003382	0.010113
LGALS8-/	7.09628995	1.052664695	0.359261	2.930085	0.003389	0.01013
PCNPP3	0.89832994	2.606334825	0.889527	2.930023	0.003389	0.01013
SSXP10	0.53731396	-1.997833415	0.681979	-2.92946	0.003395	0.010146
CHRNA3	20.8083117	-1.351169716	0.461436	-2.92818	0.00341	0.010184
IGHV3-74	254.44572	-1.46018186	0.49871	-2.92792	0.003412	0.010192
SPDYE4	0.80382089	-1.500556948	0.512526	-2.92777	0.003414	0.010193
RPS6P12	3.4690196	1.764016035	0.602865	2.926056	0.003433	0.010247
SNORD12	15.2711968	1.063941396	0.363956	2.92327	0.003464	0.010326
LINC0082	1.53509432	-1.502109139	0.514152	-2.92153	0.003483	0.01038
SELE	1654.33241	-1.622976865	0.556094	-2.91853	0.003517	0.010467
GPM6A	56.1109829	-1.587147253	0.543913	-2.91802	0.003523	0.010481
ANKRD1(	98.8900241	1.933292568	0.662607	2.917708	0.003526	0.010487
DHX35-D'	5.09622775	1.233361577	0.422779	2.917271	0.003531	0.010499
EDAR	62.3221788	-1.059662099	0.36341	-2.91588	0.003547	0.01054
CCDC42	1.37314987	-1.263343283	0.433288	-2.91572	0.003549	0.010545
DSCR9	2.20357381	1.233092751	0.422979	2.91526	0.003554	0.010555
SH3TC2-I	14.5143494	1.165451882	0.399776	2.91526	0.003554	0.010555
LINC0169	1.56334023	2.51064912	0.861383	2.914671	0.003561	0.01057
RPS17	1.74518745	1.954917786	0.670734	2.914596	0.003561	0.010571
TNNC1	140.617708	1.163256235	0.399274	2.913431	0.003575	0.010604
LINC0284	0.57890644	-2.496967877	0.857644	-2.91143	0.003598	0.01067
SLC25A24	2.8754781	2.139566341	0.735607	2.908574	0.003631	0.010762
WNT9B	1.99878152	-1.587878057	0.546313	-2.90654	0.003655	0.010825
ACTG1P2	1.77322792	1.235395933	0.425074	2.906308	0.003657	0.010831
ARMH2	0.60230214	-2.021545517	0.695614	-2.90613	0.003659	0.010835
SLC25A34	1.66495361	-1.282710247	0.441381	-2.90613	0.003659	0.010835
LINC0122	3.66096285	-1.034612904	0.35609	-2.90548	0.003667	0.010854
CSTA	2072.03137	1.118892029	0.385213	2.904608	0.003677	0.010882
EXOSC3P	3.6701027	-1.325265904	0.45642	-2.90361	0.003689	0.010911
KLRC2	14.3754808	1.455264695	0.501695	2.900697	0.003723	0.011002
NT5C3AP	26.9201979	-1.083087614	0.373409	-2.90054	0.003725	0.011005
RN7SL83	3.03439587	-1.175166783	0.405171	-2.90042	0.003727	0.011007
FAM153C	8.98238764	-1.148270425	0.395953	-2.90001	0.003731	0.01102
LINC0211	2.39330612	1.423464431	0.490879	2.899826	0.003734	0.011024
PRAC2	23.6053691	-1.634454906	0.563689	-2.89957	0.003737	0.011032
ASIP	3.04506419	-1.029884955	0.355343	-2.89828	0.003752	0.011069
CLUHP6	2.30357431	-1.205316429	0.415948	-2.89775	0.003758	0.011085
RPS27P21	3.68716811	1.005067033	0.346982	2.896597	0.003772	0.01112
OGDHL	45.6442526	1.387610524	0.479182	2.895791	0.003782	0.011146
RNU6-118	1.09618677	-2.157511323	0.745107	-2.89557	0.003785	0.011151
NBEA	463.853733	-1.019415592	0.352332	-2.89334	0.003812	0.011225
RNU6-124	1.04335431	2.024716047	0.699812	2.89323	0.003813	0.011227
GPRACR	1.81903737	-1.636453725	0.56595	-2.89152	0.003834	0.011286
H2AC18	44.7028561	1.062688212	0.367589	2.890965	0.003841	0.011303
FABP6-AS	11.6478642	1.524893345	0.527999	2.888061	0.003876	0.011398
P2RX5	95.9433664	-1.352460083	0.468678	-2.88569	0.003906	0.011472
SNX31	1845.71509	1.382216778	0.479064	2.885244	0.003911	0.011487
MXRA5Y	31.3206719	-1.267951465	0.439515	-2.88489	0.003916	0.011499
LINC0106	3.67616344	1.302477828	0.451761	2.88311	0.003938	0.011556
LINC0205	13.9158012	2.590119686	0.898946	2.881286	0.003961	0.011618

MIR554	5.34074352	1.461594728	0.507378	2.880684	0.003968	0.011637
CECR7	46.2476754	1.555232698	0.539927	2.88045	0.003971	0.011642
IGKV1-17	173.293358	-1.93006656	0.670147	-2.88006	0.003976	0.011655
RPL23AP7	1.49902791	2.044432944	0.710195	2.878691	0.003993	0.011703
GSTM2P1	3.09261307	1.702563142	0.591631	2.877745	0.004005	0.011733
RNU6-121	2.63582009	-1.45999125	0.507355	-2.87765	0.004006	0.011735
LINC0139	4.19934505	1.545542062	0.53721	2.876979	0.004015	0.011756
RNU2-57F	1.0899438	-2.372075104	0.824753	-2.8761	0.004026	0.011781
LACTB2-/	4.02221434	-1.064619045	0.370622	-2.87252	0.004072	0.011903
C20orf197	25.1933109	1.152630457	0.401317	2.872118	0.004077	0.011914
MT3	5.59429863	1.841881681	0.641453	2.871423	0.004086	0.011937
ATP5MDI	0.82539723	1.459306577	0.508357	2.870633	0.004097	0.011963
PRAME	136.283834	2.352018388	0.819826	2.868923	0.004119	0.012019
RN7SKP9	2.84320569	1.290242717	0.449887	2.867927	0.004132	0.012054
RNU6-625	0.99339729	-1.863943928	0.65005	-2.86739	0.004139	0.012068
FEZF1-AS	36.6270577	1.862704707	0.64962	2.867378	0.004139	0.012068
CFAP77	3.79132028	1.86213275	0.650071	2.864505	0.004177	0.012159
PARP15	241.900661	-1.121363746	0.391509	-2.86421	0.00418	0.012168
IGKV2D-2	152.672085	-1.598488484	0.558606	-2.86157	0.004216	0.012257
RNF217-A	24.4952504	-1.157406722	0.404468	-2.86155	0.004216	0.012257
PCARE	0.82729755	-1.825739499	0.639016	-2.85711	0.004275	0.012413
IGLV3-19	724.93742	-1.84056181	0.644236	-2.85697	0.004277	0.012417
LINC0232	1.37885269	1.782834117	0.624305	2.855711	0.004294	0.012456
PRICKLE	256.031286	-1.098452658	0.384898	-2.85388	0.004319	0.012517
CYP4F11	823.95485	1.296094508	0.454281	2.853065	0.00433	0.012547
KC6	59.2508836	1.815281618	0.636268	2.853013	0.004331	0.012547
H3P1	1.57451813	1.264974088	0.443564	2.851844	0.004347	0.01259
OR2C3	1.08012842	-1.75584522	0.615692	-2.85182	0.004347	0.01259
ZNF154	166.105579	-1.017617198	0.357083	-2.8498	0.004375	0.012658
AMH	97.6919591	1.41298484	0.496094	2.84822	0.004396	0.012709
RNU6-377	4.81584845	1.119486143	0.393106	2.847796	0.004402	0.01272
BACH1-IT	3.87660657	-1.157685472	0.406622	-2.84708	0.004412	0.012746
RN7SL213	1.62929004	1.180172962	0.414656	2.846147	0.004425	0.012782
CXorf65	27.2876108	-1.391082032	0.488862	-2.84555	0.004433	0.012801
SLC16A1(	61.013988	1.248253221	0.438772	2.844877	0.004443	0.012824
TRGV10	8.84110664	-1.315712876	0.46279	-2.843	0.004469	0.012892
MIR4511	0.75213154	-1.520250991	0.534804	-2.84263	0.004474	0.012903
SPIC	1.37078058	-1.772614644	0.623612	-2.84249	0.004476	0.012905
CCIN	12.8451743	-1.547010916	0.544474	-2.84129	0.004493	0.012949
GCNT4	204.619949	1.041246612	0.36691	2.837881	0.004541	0.013076
MTHFD2I	1.35265829	1.498683372	0.528174	2.837479	0.004547	0.013086
SLC15A1	332.179425	1.171089821	0.41277	2.837151	0.004552	0.013094
KRT8P38	0.98576862	2.866032716	1.0102	2.837094	0.004553	0.013094
IGKV1-33	386.417014	-1.748773915	0.616427	-2.83695	0.004555	0.013098
RPL7AP3	28.2193258	1.113079777	0.392359	2.836895	0.004555	0.013099
CHODL	68.4494048	-1.321968448	0.466041	-2.83659	0.00456	0.013107
SUMO2P2	6.22319181	1.152501814	0.406346	2.836259	0.004565	0.013119
POLD2P1	1.47968053	2.124171453	0.749114	2.835579	0.004574	0.013142
SNORD60	4.14826082	1.084087846	0.382586	2.83358	0.004603	0.013214
LINC0085	12.1756016	2.379635065	0.840334	2.831773	0.004629	0.013283
TEX26	1.23899319	-1.751849294	0.6187	-2.8315	0.004633	0.013292
PTPRN2-/	37.6936401	-1.111481014	0.392584	-2.8312	0.004637	0.013302

DKK2	33.1619141	1.25896224	0.444792	2.830455	0.004648	0.013328
MESP1	163.518293	1.725538492	0.60968	2.830236	0.004651	0.013336
RN7SKP1:	0.71040152	-2.239562857	0.791525	-2.82943	0.004663	0.013366
PM20D1	1904.90916	1.824333906	0.644894	2.828888	0.004671	0.013386
BCHE	168.108164	-1.474802225	0.521413	-2.82847	0.004677	0.0134
RASAL3	557.586882	-1.067797356	0.377519	-2.82846	0.004677	0.0134
E2F2	139.728702	1.009789855	0.357028	2.828323	0.004679	0.013404
SEPTIN7P	1.34390356	-1.360090487	0.481128	-2.82688	0.0047	0.013457
SLC39A1:	1.63499469	-1.372828973	0.48565	-2.82679	0.004702	0.013459
RNU6-123	2.95879267	1.058876542	0.374715	2.825815	0.004716	0.013495
DNAAF3	16.9463017	1.13485673	0.401625	2.825661	0.004718	0.013499
RNU6-431	2.60873705	1.100836984	0.38995	2.823019	0.004757	0.013606
TREML4	1.29154829	-2.203738967	0.780839	-2.82227	0.004768	0.013633
COLEC10	11.8477265	1.00248455	0.355219	2.822162	0.00477	0.013636
SLA	672.145225	-1.024807178	0.363329	-2.82061	0.004793	0.013697
ANKRD1ɛ	2.58579145	-1.180482027	0.418553	-2.82039	0.004797	0.013705
HMGN1P:	1.15243653	2.057943365	0.72968	2.820337	0.004797	0.013706
GAGE10	0.74343339	-1.484859768	0.526541	-2.82003	0.004802	0.013716
CMAHP	463.742138	-1.041461025	0.369407	-2.81928	0.004813	0.013743
LINC0147	4.11332298	-1.309797176	0.464716	-2.81849	0.004825	0.013775
DELEC1	4.76980469	1.965603352	0.697439	2.818314	0.004828	0.013781
GOLGA7I	65.4286044	1.018357225	0.361462	2.817326	0.004843	0.01382
RFPL4A	0.93833441	-2.262067162	0.803116	-2.81661	0.004853	0.013848
ANXA3	584.202354	1.11465498	0.395841	2.815917	0.004864	0.013876
RNVU1-2	1.4743242	1.601228823	0.568682	2.815686	0.004867	0.013882
EFHD1	161.951459	-1.035428547	0.367834	-2.81494	0.004879	0.013909
FAM78B	120.572157	1.233844193	0.438343	2.814789	0.004881	0.013912
EMBP1	9.48011059	1.215252023	0.431813	2.814299	0.004888	0.013932
RAB3B	192.785795	1.275370803	0.453412	2.812828	0.004911	0.013991
THEG	8.23301115	1.627860352	0.578999	2.811507	0.004931	0.014037
HYAL4	11.0269523	1.374671578	0.48903	2.811015	0.004939	0.014053
SLC19A3	23.3604878	-1.204549637	0.42873	-2.80958	0.004961	0.014115
LIMS4	2.61597637	-1.111075976	0.395471	-2.8095	0.004962	0.014116
LINC0042	1.06856981	-1.789977142	0.637182	-2.80921	0.004966	0.014127
RNU6-562	2.16099427	1.355289528	0.482541	2.80865	0.004975	0.014149
TRIM17	199.658697	1.061603869	0.378375	2.805695	0.005021	0.014262
SHOC1	30.4672251	1.337472401	0.476775	2.805251	0.005028	0.014279
SLFN13	655.681247	1.024887377	0.365828	2.801554	0.005086	0.01443
WNT10B	31.6545263	-1.112194437	0.397323	-2.79922	0.005123	0.014526
KLK11	74.5141256	1.803183649	0.644362	2.7984	0.005136	0.014558
SLC15A5	2.56396127	3.022935599	1.081184	2.795948	0.005175	0.014658
VGLL2	0.72483386	-1.812402631	0.648257	-2.79581	0.005177	0.01466
APOBEC3	155.953694	-1.155211644	0.413448	-2.79409	0.005205	0.014719
RN7SL12	7.69278552	-1.773157821	0.634717	-2.79362	0.005212	0.014736
UBE2V1P	0.5853987	-2.289234725	0.819825	-2.79235	0.005233	0.014785
TMPRSS1	2.11027222	2.728164683	0.977426	2.791173	0.005252	0.014834
ENO1P3	3.74904536	-1.079587135	0.386823	-2.79091	0.005256	0.014844
MIR5687	4.04418462	-1.037955023	0.37222	-2.78855	0.005294	0.014937
SLC25A6I	1.65863189	1.895969901	0.679965	2.788335	0.005298	0.014944
CYP2J2	782.781504	1.039144618	0.372858	2.78697	0.00532	0.014996
LINC0191	3.44754018	1.78589042	0.640826	2.786858	0.005322	0.015
SNORD11	1.22388176	-2.384923623	0.855945	-2.78631	0.005331	0.015023

MYADM-	6.06497127	-1.22029468	0.438057	-2.7857	0.005341	0.015043
CCR9	1.62270938	-1.720398553	0.61774	-2.78499	0.005353	0.015067
IGLV3-9	41.3552759	-1.880167086	0.675386	-2.78384	0.005372	0.015113
DDX11L1	1.06036635	1.532751183	0.55088	2.782371	0.005396	0.015175
PDE1B	271.520536	-1.228497758	0.441546	-2.78227	0.005398	0.015178
MIR5003	1.49970576	-1.690683556	0.607804	-2.78162	0.005409	0.015206
CHRNA7	42.7765996	-1.076882149	0.387172	-2.7814	0.005412	0.015214
LINC0152	2.29388613	-1.491571324	0.536811	-2.77858	0.00546	0.015324
TXN2P1	1.99394869	-1.295155013	0.466443	-2.77666	0.005492	0.015398
MIR4530	0.87885873	-2.090586566	0.753086	-2.77603	0.005503	0.015423
LINC0200	2.36584211	1.485193904	0.535044	2.775834	0.005506	0.015429
G3BP1P1	2.78191834	1.213574447	0.437194	2.775826	0.005506	0.015429
ADAMTS:	210.937677	-1.268653781	0.457262	-2.77446	0.005529	0.015489
UTS2B	7.9681803	-1.104458936	0.398136	-2.77408	0.005536	0.015502
NR2E1	11.9897681	2.211684495	0.797693	2.772599	0.005561	0.015563
FMO6P	1.56336886	3.135229964	1.131703	2.770364	0.005599	0.015656
APOA2	1.1091657	1.977169729	0.713708	2.770277	0.005601	0.015659
SPOCD1	2127.69167	1.178272318	0.425336	2.770217	0.005602	0.01566
DDIT4	8290.97128	1.151674494	0.415756	2.770073	0.005604	0.015661
HS3ST4	20.956173	-1.555264446	0.561816	-2.76828	0.005635	0.015739
PCDHB2	89.163307	1.102986428	0.398634	2.766916	0.005659	0.015799
CENPCP1	1.22634728	1.22649573	0.443405	2.766082	0.005673	0.015832
MIR99AH	118.310085	-1.312277538	0.474462	-2.76582	0.005678	0.015841
SMIM17	11.290062	1.001361704	0.362063	2.76571	0.00568	0.015845
IFNG-AS1	15.7087256	-1.382769402	0.500076	-2.76512	0.00569	0.01587
LINC0153	0.55058262	-2.624860753	0.94956	-2.76429	0.005705	0.015905
MAL	432.162733	1.445286151	0.522851	2.764242	0.005706	0.015906
LINC0176	9.09411594	2.825173539	1.024236	2.758324	0.00581	0.016165
WASIR2	27.4147725	1.186045582	0.430103	2.757585	0.005823	0.016198
FAM181A	2.51024813	2.410944855	0.87448	2.757005	0.005833	0.016223
RN7SL39	1.55602153	1.736633912	0.630175	2.755795	0.005855	0.016279
SIGLEC22	2.85316401	-1.353128988	0.491023	-2.75574	0.005856	0.016281
LINC0122	0.47029799	-2.058188224	0.747472	-2.75353	0.005896	0.016372
PPIAL4G	1.82549762	-1.279816432	0.464829	-2.7533	0.0059	0.01638
CAMK2N	40.1977846	1.279502727	0.464813	2.752723	0.00591	0.016405
CLDN10	53.3121964	1.773926258	0.645544	2.747954	0.005997	0.016617
CHST13	13.8990984	-1.04070039	0.378726	-2.7479	0.005998	0.016618
RNY3P11	1.69041713	2.524693897	0.918892	2.747542	0.006004	0.016634
SUPT20H	0.56267817	-1.802726234	0.65652	-2.74588	0.006035	0.016709
MIRLET7	2.006327	-1.148666539	0.418335	-2.7458	0.006036	0.016711
OR7E158I	1.5261609	2.341289792	0.852719	2.745676	0.006039	0.016716
LCT-AS1	2.21838506	-1.554771838	0.566598	-2.74405	0.006069	0.016791
RASGRP1	236.514399	-1.075317074	0.391925	-2.74368	0.006075	0.016806
LINC0232	10.2845209	1.054274645	0.384676	2.74068	0.006131	0.016941
FAM189A	134.387853	1.643062812	0.599835	2.739189	0.006159	0.017016
ACSM2B	1.47513738	-1.678777224	0.61298	-2.73871	0.006168	0.017037
GSTM1	4408.41555	2.430406126	0.887892	2.737275	0.006195	0.0171
MANCR	16.9378981	1.298995526	0.474919	2.735194	0.006234	0.017191
RTL9	3.67110901	-1.136262764	0.415534	-2.73446	0.006248	0.017226
SULT1D1	1.00479616	2.695035002	0.98611	2.732996	0.006276	0.017299
TRBV7-7	0.76210639	-1.972329097	0.721698	-2.7329	0.006278	0.0173
CHMP5P1	1.58249756	1.178850831	0.431664	2.730948	0.006315	0.017391

ICAM4	50.1573423	-1.071556574	0.392394	-2.73082	0.006318	0.017396
MIR155H(	70.0150091	-1.050020413	0.384563	-2.73042	0.006325	0.017415
ICAM1	3012.49994	-1.08513233	0.397464	-2.73014	0.006331	0.017426
PCDHB17	4.60309914	1.475333393	0.540484	2.729654	0.00634	0.017449
FREM2	307.886309	1.60978002	0.590298	2.727065	0.00639	0.017579
MIR5190	15.8238707	-1.029025303	0.377359	-2.72692	0.006393	0.017585
STMND1	1.91213164	-2.108187306	0.77356	-2.7253	0.006424	0.017659
COL6A4P	39.1295428	1.141790317	0.418965	2.725266	0.006425	0.017659
MYOZ2	8.27515343	-1.452843908	0.533133	-2.72511	0.006428	0.017664
KCNK3	159.829783	-1.456914603	0.534914	-2.72364	0.006457	0.017734
VSTM2A-	2.19787821	-2.145458803	0.787866	-2.72313	0.006467	0.017754
LINC0199	0.64374682	-2.221489251	0.816018	-2.72235	0.006482	0.017787
LINC0224	3.06309833	-1.49210574	0.548322	-2.72122	0.006504	0.017843
BEND3P1	1.62409955	-1.113327692	0.409267	-2.72029	0.006522	0.017887
SYT9	18.1098148	1.339233535	0.492352	2.720072	0.006527	0.017893
MRGPRE	1.15482079	-1.92607623	0.708544	-2.71836	0.006561	0.017967
MIR3612	0.81484609	-2.670503768	0.982411	-2.71832	0.006562	0.017967
IGKV2-28	213.755303	-1.620053812	0.595982	-2.71829	0.006562	0.017967
PTCRA	4.20233802	-1.273405071	0.468754	-2.71658	0.006596	0.01805
SNRPGP9	1.45101527	-1.442921537	0.531363	-2.71551	0.006617	0.018102
SNORD11	0.628629	-1.788055068	0.658527	-2.71523	0.006623	0.018115
SLC12A5-	3.46357825	1.865476478	0.687417	2.71375	0.006653	0.018182
TATDN2F	1.37634566	-1.139306323	0.419853	-2.71358	0.006656	0.018187
HSPA8P8	0.83130088	1.347394627	0.496964	2.711253	0.006703	0.018306
RTL3	2.51200871	-1.784951048	0.658397	-2.71106	0.006707	0.018314
LINC0178	1.79197545	2.170750504	0.801083	2.709769	0.006733	0.018375
CPHL1P	25.3391823	1.213958959	0.448086	2.709209	0.006744	0.018404
BTF3L4P3	1.64336103	1.169312237	0.431926	2.707207	0.006785	0.018497
RN7SL471	3.60754727	2.166028961	0.800152	2.707022	0.006789	0.018505
CDH11	1671.38804	-1.013303016	0.374381	-2.70661	0.006797	0.018526
LINC0152	1.10694713	1.53950349	0.569459	2.703448	0.006862	0.018691
SP140	210.922861	-1.106169469	0.409237	-2.703	0.006872	0.018712
LINC0267	31.8380935	2.237253756	0.82807	2.701768	0.006897	0.018773
COL1A2	44029.5847	-1.077578026	0.398879	-2.70152	0.006902	0.018783
NDUFAF4	0.54383189	2.354954242	0.871827	2.701173	0.00691	0.0188
UBA52P5	0.74933185	2.180250075	0.807168	2.70111	0.006911	0.018802
PLEK	863.31565	-1.061485605	0.393127	-2.70011	0.006932	0.018854
IVL	1270.59919	1.522195703	0.564209	2.697927	0.006977	0.01897
EIF5AP2	1.8005936	-1.688612749	0.625997	-2.69748	0.006987	0.018991
MYRF-AS	0.58317569	1.954107481	0.724447	2.69738	0.006989	0.018995
IGKV4-1	1608.98791	-1.616852454	0.599546	-2.69679	0.007001	0.019022
C11orf72	1.6421923	1.28211927	0.475476	2.696494	0.007007	0.019037
TCF15	27.3076847	1.143863036	0.424274	2.69605	0.007017	0.019058
KL	98.0901283	-1.007472492	0.373726	-2.69575	0.007023	0.019073
MYL10	1.06546043	-1.838465242	0.682015	-2.69564	0.007025	0.019076
ZIC4	1.02517489	2.602253051	0.965362	2.695624	0.007026	0.019076
ICOS	112.508219	-1.284716818	0.476729	-2.69486	0.007042	0.019107
B3GAT2	12.8930488	-1.560685165	0.57938	-2.69372	0.007066	0.019166
SCUBE2	2721.84354	-1.060723548	0.393789	-2.69364	0.007068	0.019169
RGMA	167.320938	-1.176090263	0.43666	-2.69338	0.007073	0.019179
SMARCE1	1.94713588	2.73994733	1.017437	2.69299	0.007081	0.0192
PCYT1B	29.2759667	1.478057414	0.549249	2.691053	0.007123	0.019303

BMS1P16	1.79772206	1.344444708	0.499713	2.690431	0.007136	0.01933
LINC0264	1.80278417	-1.618278641	0.60155	-2.69018	0.007141	0.019341
MAOB	505.139526	-1.353707798	0.503802	-2.68698	0.00721	0.019492
CYP3A7	37.4442967	1.529976129	0.569415	2.686924	0.007211	0.019493
TRAJ1	2.97117107	-1.596349168	0.594299	-2.68611	0.007229	0.019539
MIR557	0.60502859	2.130789871	0.793362	2.685771	0.007236	0.019556
IGSF1	48.028268	1.048067375	0.390286	2.685381	0.007245	0.019575
LINC0255	7.11482123	1.174907334	0.437537	2.685272	0.007247	0.019577
SNORA74	4.26999195	1.036992102	0.386248	2.68478	0.007258	0.019597
LINC0142	22.683816	1.046130843	0.389697	2.684469	0.007265	0.01961
DLG3-AS	4.37421232	1.286609161	0.479282	2.68445	0.007265	0.01961
MIR5000	1.84048761	-1.183434051	0.440861	-2.68437	0.007267	0.01961
KRT8P5	3.29704842	1.120384568	0.417455	2.683848	0.007278	0.019637
LINC0243	2.00247955	-1.457399258	0.543317	-2.68241	0.007309	0.019713
MIR3170	3.25112192	-1.004910479	0.374656	-2.68222	0.007313	0.019721
CLEC4D	14.8555149	-1.298764142	0.484255	-2.68198	0.007319	0.019733
PLSCR3	2.76636268	1.00276107	0.374348	2.67869	0.007391	0.019911
IFITM3P9	1.81310614	2.81254935	1.050002	2.678614	0.007393	0.019912
C5orf64	1.34580729	-1.245758111	0.4652	-2.6779	0.007409	0.019949
LAX1	174.092773	-1.213638091	0.453444	-2.67649	0.00744	0.020018
LINC0027	0.99105538	1.685362094	0.629914	2.675544	0.007461	0.020065
FMO9P	937.322041	1.53911605	0.575305	2.675306	0.007466	0.020075
CATSPER	2.48010847	1.084937016	0.405671	2.674424	0.007486	0.020121
KCNJ3	20.1070672	-1.607928184	0.601442	-2.67345	0.007507	0.020175
FAM30A	72.0608271	-1.589480142	0.594791	-2.67233	0.007533	0.020229
ALAS2	3.90293129	-1.522982579	0.569993	-2.67193	0.007542	0.020249
LINC0287	1.10846244	1.743532669	0.652684	2.671327	0.007555	0.020277
ONECUT	529.131524	1.524233416	0.570865	2.670043	0.007584	0.020343
DNAH8-A	1.14102114	-1.745780739	0.653901	-2.66979	0.00759	0.020355
RN7SL757	1.18224132	-1.422232172	0.532717	-2.66977	0.00759	0.020355
IGKV3D-2	135.128407	-1.862853002	0.697817	-2.66954	0.007595	0.02036
CFAP58-D	9.71887679	1.055150748	0.395365	2.668803	0.007612	0.020396
KCNE1	40.1642557	-1.015281723	0.380547	-2.66795	0.007631	0.020437
AOX2P	0.56579424	-1.847568531	0.69253	-2.66785	0.007634	0.02044
SNORD58	1.54449469	1.498948111	0.561916	2.667565	0.00764	0.020456
LINC0270	2.56027613	-1.158554383	0.434415	-2.66693	0.007655	0.02049
IQCM	6.63643144	1.353375112	0.508164	2.663264	0.007739	0.02069
AIMP1P1	8.06469434	-1.149382394	0.431604	-2.66305	0.007744	0.020701
GNG8	5.07628718	-1.230069377	0.462148	-2.66163	0.007776	0.020779
LINC0185	1.47973348	3.532500254	1.327387	2.661243	0.007785	0.020798
GULOP	2.58894487	-1.086533654	0.408488	-2.65989	0.007817	0.020873
SPRR2F	1.62319705	2.422064201	0.910595	2.65987	0.007817	0.020873
RPL9P16	6.37258905	-1.0818097	0.406717	-2.65986	0.007817	0.020873
RN7SL172	11.7721171	-1.223210746	0.460221	-2.65788	0.007864	0.020968
RPS3AP46	3.55194272	-1.222193456	0.459877	-2.65765	0.007869	0.02098
RPS16P5	2.09042783	-1.229523132	0.462723	-2.65715	0.00788	0.021009
BET1P1	0.5632642	-2.160717459	0.813407	-2.65638	0.007898	0.02105
PCDH9	75.5217309	-1.438477195	0.541555	-2.6562	0.007903	0.021059
MIR1972-	1.56379505	-2.702294155	1.017368	-2.65616	0.007904	0.021059
KRT8P36	3.8550176	1.120961202	0.422089	2.655746	0.007913	0.021076
F2	2.75792594	-1.120712618	0.422238	-2.65422	0.007949	0.021166
MYO16	11.2148457	-1.005256095	0.378969	-2.65261	0.007987	0.021247

ALPP	14.3551711	1.616889814	0.609931	2.65094	0.008027	0.02134
RPL39P15	72.3995598	1.010457762	0.381267	2.650266	0.008043	0.021376
TMEM97	3941.8576	1.074762845	0.405543	2.650183	0.008045	0.021379
HLA-DRB	2180.11928	-1.530850032	0.577797	-2.64946	0.008062	0.02142
IL19	9.67805158	-1.376978542	0.51992	-2.64844	0.008086	0.02148
CATSPER	3.76979963	-1.227554948	0.463596	-2.6479	0.008099	0.021512
VSTM2B	0.40686391	-1.813656587	0.685572	-2.64547	0.008158	0.021641
XIAPP3	0.84309075	1.711340194	0.647014	2.644982	0.00817	0.021664
SNRPCP2	1.13726235	1.299718642	0.49147	2.644556	0.00818	0.021688
SNORA80	0.9972639	1.595625755	0.603497	2.643968	0.008194	0.021721
FCRLA	77.6335984	-1.474325193	0.557679	-2.64368	0.008201	0.021737
C5AR1	747.444152	-1.03037251	0.389753	-2.64366	0.008202	0.021737
CTLA4	182.748355	-1.175417264	0.444676	-2.64331	0.00821	0.021756
RNU6-705	3.67319234	1.876241199	0.71028	2.641552	0.008253	0.021856
MT2P1	3.89094484	-1.149619744	0.435217	-2.64149	0.008254	0.021857
CEACAM	1531.63135	-2.115201049	0.801089	-2.64041	0.008281	0.021918
RNU7-47F	2.69849364	-1.173612421	0.444613	-2.63963	0.0083	0.021957
ZNF208	90.2348568	1.398852391	0.530073	2.638979	0.008316	0.021994
RNA5SP2!	4.74582282	-1.434995551	0.544426	-2.6358	0.008394	0.02216
DIP2C-AS	31.8611856	-1.143983783	0.434189	-2.63476	0.00842	0.022216
GJB4	399.35657	1.089906247	0.413712	2.634459	0.008427	0.02223
BEST2	0.61773863	1.867702622	0.708958	2.634433	0.008428	0.02223
LINC0130	19.5780348	1.660371178	0.631296	2.630099	0.008536	0.022472
ENPP7P2	7.56313207	1.561129237	0.593655	2.629693	0.008546	0.022494
MAPK4	87.0897065	-1.332888171	0.506912	-2.62943	0.008553	0.022504
KRT16P3	8.86932977	1.566794939	0.596166	2.628118	0.008586	0.022584
FDPSP8	0.72649705	2.13105815	0.81099	2.627725	0.008596	0.022607
FAM222A	2.77009284	1.12611112	0.428615	2.627327	0.008606	0.022624
HBQ1	1.9454379	2.693272584	1.025158	2.627179	0.00861	0.022629
SHISAL2I	2.316626	1.475733418	0.561814	2.626729	0.008621	0.02265
PIK3R5-D	4.5549476	-1.061039546	0.404101	-2.62568	0.008648	0.022714
LIX1-AS1	1.22608779	2.264255057	0.86242	2.625465	0.008653	0.022723
RNY3P15	2.02585183	1.072003481	0.40833	2.625334	0.008656	0.02273
EZH2P1	1.36468062	1.415203352	0.540024	2.620631	0.008777	0.023014
HTRA4	28.0268289	1.036836999	0.395672	2.620444	0.008782	0.023019
RNU6-316	1.45191405	1.691479152	0.645526	2.620311	0.008785	0.023025
SYNPR-A	7.76537383	1.472206655	0.56186	2.620238	0.008787	0.023028
PSME2P6	1.41772944	1.275983235	0.487047	2.619835	0.008797	0.023045
LGMNP1	2.08410765	-1.131239213	0.432084	-2.6181	0.008842	0.023145
SNORD11	1.41128713	1.266540417	0.484363	2.614856	0.008927	0.023341
RNU11-6F	1.43216029	-1.275463191	0.487802	-2.61471	0.00893	0.023348
GAPDHP2	2.720205	1.976272661	0.75589	2.614498	0.008936	0.023358
LINC0283	0.75510108	2.125216964	0.814326	2.609788	0.00906	0.023642
LINC0253	1.06291492	2.746084194	1.052275	2.609663	0.009063	0.023645
LINC0192	1.27442608	-1.975158446	0.756992	-2.60922	0.009075	0.023668
RFX8	32.1407905	1.077203226	0.4129	2.608871	0.009084	0.023685
RPS24P17	0.93119128	-1.542474251	0.591348	-2.6084	0.009097	0.02371
PPIAP3	2.24690711	1.073402492	0.411662	2.607487	0.009121	0.023763
PAX6	28.0304307	1.72174394	0.660336	2.607377	0.009124	0.023768
HNRNPLI	2.61353719	1.06310738	0.407882	2.606412	0.00915	0.02383
BAALC-A	2.72206573	1.33522617	0.51237	2.60598	0.009161	0.023855
SGCZ	4.89415903	-1.402123715	0.538068	-2.60585	0.009165	0.023862

NFIA-AS2	2.93605985	-1.591998453	0.610961	-2.60573	0.009168	0.023867
FAM221B	12.2921575	-1.151791351	0.442052	-2.60556	0.009173	0.023877
SNORD13	10.679175	-1.016029692	0.389989	-2.60528	0.00918	0.023891
TPTEP1	269.39398	-1.20470051	0.462845	-2.60282	0.009246	0.024058
KRT8P9	2.40471559	-1.326270396	0.509762	-2.60174	0.009275	0.024123
LINC0202	3.87202557	1.303589684	0.501385	2.599976	0.009323	0.024233
RNU6-878	0.90435049	-1.901873836	0.731961	-2.59833	0.009368	0.024328
ARHGEF4	370.859314	1.004696086	0.386697	2.598145	0.009373	0.024337
IL2RG	688.86935	-1.003139663	0.386344	-2.59649	0.009418	0.024435
TOMM20l	16.1282539	-1.366262396	0.526371	-2.59563	0.009442	0.024494
CPB2	1.1590797	2.145996779	0.826813	2.595504	0.009445	0.024501
MRGPRX:	9.08974863	-1.710524412	0.659439	-2.59391	0.009489	0.024604
SNORA15	0.62122934	1.650028623	0.636446	2.592567	0.009526	0.024692
KCND2	80.8931994	1.003872805	0.387244	2.592352	0.009532	0.024705
IGKV1-6	166.930849	-1.658872666	0.639947	-2.5922	0.009536	0.024708
LINC0189	2.19417241	-1.546643822	0.596718	-2.59192	0.009544	0.024721
IMP3P2	0.64735997	-1.823076791	0.703533	-2.59132	0.009561	0.024761
LINC0141	3.33374383	1.304779714	0.503954	2.589086	0.009623	0.024898
C1DP5	2.92120996	-1.075043398	0.415417	-2.58786	0.009657	0.024971
RN7SL62:	0.92263631	-1.378317279	0.53271	-2.58737	0.009671	0.025002
MIR199A:	1.04056111	-1.610971641	0.622776	-2.58676	0.009688	0.025038
SMC1B	47.2802898	1.443037201	0.557952	2.58631	0.009701	0.025065
HRH2	62.4111186	-1.196977844	0.462875	-2.58596	0.009711	0.025085
SERPINB7	20.0980097	1.739400536	0.672748	2.585516	0.009723	0.025115
NBPF5P	1.10485491	2.412899772	0.933456	2.584911	0.00974	0.025151
IGLV5-37	1.42613917	-2.303092222	0.891341	-2.58385	0.00977	0.025215
FOXL2	24.6643717	1.899048389	0.735002	2.583732	0.009774	0.025219
LINC0142	5.45751514	2.004450141	0.775824	2.58364	0.009776	0.025223
MEP1B	1.19824735	1.694645833	0.656151	2.582707	0.009803	0.025274
SLC7A11-	2.61152405	1.910485184	0.7399	2.582086	0.009821	0.025315
DUOXA2	735.732311	-1.53763771	0.59559	-2.5817	0.009831	0.025338
SERPINB:	2846.09999	1.971956958	0.763849	2.581605	0.009834	0.025343
SALL4P7	0.4623924	1.754812023	0.679771	2.581475	0.009838	0.025349
IGHV1-2	363.393899	-1.84911214	0.716319	-2.58141	0.00984	0.025352
LINC0249	1.41926846	-1.494885711	0.579198	-2.58096	0.009853	0.025379
PPEF2	1.22661561	-1.072807108	0.415796	-2.58013	0.009876	0.025435
ADAMTS	83.1393571	-1.354243318	0.52492	-2.5799	0.009883	0.025441
MGC2738	2.62664875	-1.245479068	0.482824	-2.57957	0.009892	0.025463
CCDC38	4.73130632	1.055305417	0.409135	2.579355	0.009899	0.025476
RNASEH1	1.00203295	1.47519853	0.572368	2.577359	0.009956	0.025606
OR7E116I	2.36970372	2.521384606	0.978538	2.576684	0.009975	0.025644
ZNF969P	5.55662417	1.362326774	0.528905	2.575752	0.010002	0.025702
RPL9P33	0.66016308	-1.94888873	0.756843	-2.57502	0.010023	0.025746
LINC0251	13.3053101	1.030039303	0.400035	2.574871	0.010028	0.025752
RN7SL66:	0.88243407	-1.903277447	0.739258	-2.57458	0.010036	0.025762
PCDHGB1	21.5339735	1.101758524	0.428029	2.574025	0.010052	0.025801
SLC7A4	97.7378844	1.068153467	0.41499	2.573925	0.010055	0.025804
RGS9BP	11.5575387	-1.045706389	0.4063	-2.57373	0.010061	0.02581
LINC0212	1.26508439	-1.268742901	0.493095	-2.57302	0.010082	0.025857
CD7	277.448812	-1.032836014	0.401424	-2.57293	0.010084	0.025861
RNU6-729	1.32006556	-1.190084334	0.46266	-2.57227	0.010104	0.025904
GAST	1.02909208	3.064269724	1.191301	2.572205	0.010105	0.025904

ST3GAL6-	6.58967859	-1.052122094	0.409056	-2.57207	0.010109	0.02591
DHX40P1	1.74459053	1.262953588	0.491205	2.571132	0.010137	0.025971
RN7SL398	2.82553737	-1.276506358	0.496488	-2.57107	0.010138	0.025973
KIR3DL2	2.12884646	-1.52870166	0.594616	-2.5709	0.010143	0.025983
RPL4P1	3.29887133	-1.080178731	0.420362	-2.56964	0.01018	0.026072
TATDN2P	1.29271565	-1.850437817	0.72055	-2.56809	0.010226	0.026175
ATP13A4	65.0754178	1.317199442	0.512963	2.567825	0.010234	0.02619
MIR944	1.49738484	1.563729646	0.609123	2.567184	0.010253	0.026227
RNA5SP3-	0.54370972	2.101518292	0.818633	2.567107	0.010255	0.026228
KCTD19	4.49719619	-1.144042319	0.445834	-2.56607	0.010286	0.026295
ITGB2	1994.96189	-1.004009534	0.391293	-2.56588	0.010292	0.0263
PIK3CD-A	1.97797347	-1.397449922	0.544878	-2.5647	0.010326	0.026374
SH2D1A	140.313736	-1.222078794	0.476639	-2.56395	0.010349	0.026429
MIR1302-:	1.17946673	1.511788672	0.589807	2.563191	0.010371	0.026472
SLC9C1	6.47752258	1.021339526	0.39858	2.562445	0.010394	0.026515
VSX1	1.4910492	-1.676497984	0.654264	-2.56242	0.010395	0.026515
HOTTIP	248.241467	-1.060732514	0.41402	-2.56203	0.010406	0.02654
TRGV3	5.35971629	-1.26573332	0.494102	-2.56169	0.010417	0.026557
CASC20	4.07559915	2.458840468	0.960145	2.560905	0.01044	0.026595
H3P4	2.85323318	-1.538604187	0.600819	-2.56085	0.010442	0.026596
LINC0048	75.8793791	1.1053836	0.431759	2.560185	0.010462	0.026642
ACRV1	5.24822765	1.658518216	0.647837	2.560085	0.010465	0.026646
CASC19	3.15278329	2.412219433	0.942442	2.559542	0.010481	0.026682
RPL7P11	0.92726388	1.728717349	0.675559	2.558942	0.010499	0.02672
MOGAT1	1.20788934	1.738839257	0.679722	2.558161	0.010523	0.026772
E2F3P2	1.89777634	1.126921067	0.440825	2.556394	0.010576	0.026897
RNU1-72P	1.26319973	1.377607827	0.538921	2.556233	0.010581	0.026907
MIR5706	0.72779259	-1.528665423	0.598186	-2.5555	0.010603	0.02695
MEGF10	12.7073833	1.040999629	0.407358	2.555491	0.010604	0.02695
LINC0156	1.83944419	2.466780581	0.965411	2.555162	0.010614	0.026973
TMEM178	263.974053	1.002521203	0.392434	2.554621	0.01063	0.027004
SNORA49	5.56641574	-1.527351884	0.598029	-2.55398	0.01065	0.027048
HNF1A	1.47350433	-1.37313564	0.537915	-2.5527	0.010689	0.027136
TIGIT	285.774616	-1.120977908	0.439244	-2.55206	0.010709	0.027174
TRIM34	2.98030352	1.018568629	0.399277	2.551033	0.01074	0.027252
FAM163B	5.21698569	-1.333556412	0.522785	-2.55087	0.010746	0.027262
PLA1A	108.519948	-1.02332168	0.401215	-2.55056	0.010755	0.02728
MIR4689	0.71671217	1.766009899	0.693321	2.547177	0.01086	0.027505
PPIAP75	0.61751022	2.02859835	0.796465	2.547001	0.010865	0.027512
MAFA-AS	3.12477561	1.607232298	0.631177	2.546405	0.010884	0.027543
PPY2P	2.56950883	3.062284082	1.202663	2.546253	0.010889	0.027551
ANO5	127.994812	-1.07944736	0.424134	-2.54506	0.010926	0.027636
DIPK1C	4.96295386	-1.668270541	0.655712	-2.54421	0.010952	0.027692
MIRLET7.	1.69029567	-1.199401516	0.471513	-2.54373	0.010968	0.027727
LINC0168	2.11291818	3.078896281	1.210548	2.543391	0.010978	0.027746
MIR222	6.38429377	-1.134308307	0.446265	-2.54178	0.011029	0.027854
TRAJ19	2.70997663	-1.429275858	0.562377	-2.54149	0.011038	0.027874
LINC0162	0.71664192	2.549475622	1.003182	2.541389	0.011041	0.027879
OR2S1P	1.35240143	-1.228634126	0.483762	-2.53975	0.011093	0.027996
LNC-LBC:	11.6015494	1.002563248	0.394953	2.538435	0.011135	0.028092
NEU4	13.403199	-1.17302823	0.462302	-2.53736	0.011169	0.028161
KRT15	4670.42899	1.273815695	0.50246	2.535158	0.01124	0.028316

CPP	6.6305208	1.35758217	0.535597	2.53471	0.011254	0.028349
PSD2	19.4468979	1.101211785	0.43464	2.533618	0.011289	0.028411
MINAR2	1.43144658	-1.378104056	0.544321	-2.53178	0.011348	0.028525
FAM53B-A	0.54967528	-2.040127208	0.806071	-2.53095	0.011375	0.028587
B3GNTL1	2.50613528	-1.257391242	0.496955	-2.53019	0.0114	0.028643
MIR1277	0.68372022	-1.616996836	0.639243	-2.52955	0.011421	0.028685
MZB1	587.347468	-1.490979833	0.589589	-2.52885	0.011444	0.028729
RNU4-62P	24.3460642	-1.036541815	0.409931	-2.52858	0.011453	0.028745
UTF1	1.55222065	-1.471774893	0.583616	-2.52182	0.011675	0.029237
RNA5SP4(	2.39132289	1.00746219	0.399504	2.521782	0.011676	0.029237
LINC0188	2.61026516	1.262998251	0.500958	2.521166	0.011697	0.029279
RPS6KA2-	3.75667752	-1.028031807	0.407805	-2.52089	0.011706	0.029299
IGKV2-30	159.550421	-1.458613118	0.578693	-2.52053	0.011718	0.02932
DRC7	4.49301506	1.131107333	0.448814	2.520215	0.011728	0.029344
IGHV7-81	0.99492143	-2.182288478	0.866648	-2.51808	0.0118	0.029504
TBX21	53.7704504	-1.076038484	0.42756	-2.5167	0.011846	0.029596
MIPEPP3	1.59559591	1.133212466	0.450343	2.51633	0.011858	0.029624
FAT2	950.533875	1.151623034	0.458121	2.513798	0.011944	0.029819
PPP2R2C	58.5834443	1.193896929	0.475158	2.512631	0.011983	0.029899
RNA5SP4(	2.71621029	1.323677771	0.52686	2.512391	0.011992	0.029916
CD300E	242.250029	-1.18990611	0.47369	-2.51199	0.012005	0.029947
NLRP10	0.80639345	1.916638929	0.763091	2.511677	0.012016	0.029965
SLC51B	5.84100922	-1.59248356	0.634388	-2.51027	0.012064	0.030076
TUBB7P	1.39438493	1.620367763	0.645849	2.508894	0.012111	0.03018
OR7E22P	22.4668281	1.255095462	0.500365	2.508359	0.012129	0.03022
RNA5SP2!	0.52694879	1.740643354	0.694434	2.506563	0.012191	0.030358
LINC0128	4.34285614	1.128344546	0.45035	2.505483	0.012228	0.030436
TRAJ33	0.67920207	-1.744362547	0.696368	-2.50494	0.012247	0.030476
STOML3	1.51069084	1.395368978	0.557072	2.504827	0.012251	0.030483
LINC0275	1.6448352	-1.247342426	0.49841	-2.50264	0.012327	0.030643
RGPD1	22.876126	1.03810453	0.414893	2.502099	0.012346	0.03068
DYRK3-A	2.49314931	1.042755088	0.416755	2.502079	0.012347	0.03068
LINC0067	1.60699953	1.087122489	0.434604	2.501411	0.01237	0.030719
LINC0153	1.2182666	-1.614965794	0.645668	-2.50123	0.012376	0.030731
LINC0198	0.41528369	-1.915860198	0.766555	-2.49931	0.012443	0.030882
TRAV26-2	2.17751423	-1.488053441	0.595389	-2.4993	0.012444	0.030882
CRYGD	0.78614982	-2.387142682	0.955158	-2.49921	0.012447	0.030885
NUS1P2	7.95008788	1.878107692	0.75158	2.498878	0.012459	0.030911
MANSC4	3.36484286	1.187075054	0.475495	2.496505	0.012542	0.031088
LINC0118	0.81490335	-1.78277539	0.714111	-2.4965	0.012543	0.031088
KRT18P54	2.07791511	1.315268044	0.526944	2.496028	0.012559	0.031123
HMGB1P1	2.48964791	2.023467469	0.810739	2.49583	0.012566	0.031134
LINC0125	1.84812875	1.991298746	0.797895	2.495689	0.012571	0.03114
LINC0167	108.045472	1.274044815	0.510738	2.494516	0.012613	0.031218
TRAJ26	0.64970919	-1.984561896	0.796184	-2.49259	0.012681	0.031362
PPP4R4	34.8270012	1.307962872	0.524757	2.49251	0.012684	0.031366
LINC0049	11.3532754	-1.659863995	0.666105	-2.4919	0.012706	0.031417
RNA5SP3:	1.80857104	1.406592969	0.564713	2.49081	0.012745	0.031503
SNORD71	3.42976262	1.033870273	0.415156	2.490317	0.012763	0.031541
RNU6-298	1.44952539	1.409928802	0.566295	2.489741	0.012784	0.031582
SIAH3	2.75823997	-1.257417509	0.505189	-2.489	0.01281	0.031629
SREK1IP1	1.73278556	1.192919586	0.479324	2.488753	0.012819	0.031648

LIPC-AS1	0.60313631	1.738587377	0.698974	2.487343	0.01287	0.031756
SNORD67	1.62951513	1.214676631	0.48845	2.4868	0.01289	0.031787
IFIT1P1	2.3434996	1.110753572	0.446687	2.48665	0.012895	0.031797
NEFM	54.4891234	-1.399036702	0.562634	-2.48658	0.012898	0.0318
SLC22A2	2.75920806	-1.666409243	0.670277	-2.48615	0.012913	0.031835
OR7E130I	0.84721693	1.902873747	0.766122	2.483773	0.013	0.032029
RNU6-123	2.42402798	-1.122316466	0.452047	-2.48274	0.013038	0.032103
RN7SL67(	0.57564705	-1.555677933	0.626599	-2.48273	0.013038	0.032103
AMPH	82.1934287	-1.010742258	0.407132	-2.48259	0.013043	0.032113
ZNF622P1	0.95009935	-1.203182669	0.48467	-2.48248	0.013047	0.032117
NSG1	900.106447	1.028770021	0.414511	2.481891	0.013069	0.03216
LINC0116	0.53892434	-1.74978239	0.705456	-2.48036	0.013125	0.032286
COX8C	1.42310785	1.174052481	0.473364	2.480234	0.01313	0.032293
YTHDF1P	3.91911214	1.488853323	0.6004	2.479768	0.013147	0.032332
LINC0237	5.09428761	3.314590107	1.337056	2.479022	0.013174	0.032397
AGMO	69.8270258	1.189976159	0.480029	2.478969	0.013176	0.032398
LEP	11.0850687	-1.486072735	0.599507	-2.47882	0.013182	0.032405
MIR4520-	3.45634	1.094478465	0.441758	2.47755	0.013229	0.032501
RSPH10B	2.48629491	1.003490344	0.405175	2.476682	0.013261	0.032567
SNORD11	0.8215345	-3.32851321	1.345151	-2.47445	0.013344	0.032746
DIO2-AS1	1.6258786	2.917444737	1.179486	2.473487	0.01338	0.032824
ESX1	1.09138362	2.765644239	1.118274	2.473137	0.013393	0.032846
OSTN-AS	2.46362193	-1.251574872	0.506269	-2.47216	0.01343	0.03293
IL18RAP	68.2248344	-1.055069045	0.426975	-2.47103	0.013472	0.03302
CDHR1	59.9892959	1.07519065	0.435436	2.469225	0.013541	0.033174
LINC0138	3.2954284	1.348934824	0.546366	2.468921	0.013552	0.033192
RPL30P13	1.33542324	-1.261613218	0.511386	-2.46705	0.013623	0.033343
IGFL3	1.28206937	3.63148308	1.472867	2.465587	0.013679	0.033456
TRGC2	62.3667489	-1.084410633	0.439847	-2.46543	0.013685	0.033459
RGS17	79.286079	1.015316858	0.411826	2.465404	0.013686	0.033459
TRAV29D	5.77294625	-1.217642764	0.49403	-2.46471	0.013712	0.033517
SPDEF	132.528069	1.06299595	0.431352	2.464334	0.013727	0.033542
SLC25A6I	0.83179563	1.437458919	0.583403	2.463922	0.013743	0.033574
IGHV3-23	2056.9187	-1.504106491	0.61049	-2.46377	0.013749	0.033585
LSM1P1	0.51601558	-2.257346462	0.916384	-2.46332	0.013766	0.033624
TRAV6	4.41810361	-1.225273096	0.497489	-2.46291	0.013781	0.033652
AKR1C7P	4.91144941	1.194349541	0.485007	2.462539	0.013796	0.033677
RPS27P25	1.19196995	2.353276453	0.955981	2.461635	0.013831	0.033749
MIR490	0.47057281	-1.68757431	0.685659	-2.46124	0.013846	0.033776
LINC0062	0.84633767	2.599002286	1.056369	2.460317	0.013881	0.033839
RPS26P49	3.89268174	1.277348962	0.519213	2.460164	0.013887	0.03385
RPS2P25	0.71933777	2.548431831	1.036606	2.458439	0.013954	0.033996
LINC0141	0.67435055	2.069838112	0.842116	2.457903	0.013975	0.03404
TUBB3P1	1.15489924	2.43061373	0.989283	2.456945	0.014012	0.034121
LRCOL1	3.60870845	-1.10056451	0.448122	-2.45595	0.014051	0.034209
FTH1P5	5.07245236	-1.20865355	0.492171	-2.45576	0.014059	0.03422
WDR49	5.03034664	-1.053550051	0.429674	-2.45197	0.014208	0.034554
TMEM23(	4.82832937	-1.293920239	0.527711	-2.45195	0.014209	0.034554
KRT8P35	1.22176144	2.058340653	0.839906	2.450679	0.014259	0.034658
LINC0199	3.96617437	1.432075077	0.584449	2.450298	0.014274	0.034692
KCNG2	4.71254495	-1.15921621	0.473371	-2.44885	0.014331	0.034814
RNA5SP3	0.88172595	1.607739215	0.656686	2.448262	0.014355	0.03486

PRDM13	2.09027022	2.465243596	1.007634	2.446566	0.014422	0.034988
NXT1-AS	1.26716228	1.132828557	0.463159	2.445873	0.01445	0.035047
TWIST1	261.850328	-1.002842987	0.410223	-2.44463	0.0145	0.035151
GHRHR	2.70019409	1.771956243	0.724913	2.444372	0.01451	0.035166
MIR4673	0.40810772	1.662772585	0.680677	2.442822	0.014573	0.035299
SCN1A-A	19.5504603	1.414464381	0.579232	2.441967	0.014607	0.035369
TLR12P	0.62688635	-1.924960657	0.788655	-2.44081	0.014654	0.035464
ASB11	1.22331001	-1.320106924	0.540954	-2.44033	0.014674	0.035504
RN7SL34	1.36180617	-1.01887774	0.417702	-2.43924	0.014718	0.03558
LINC0208	5.29554337	-1.12248021	0.460428	-2.43791	0.014773	0.03569
SLC25A4	1.46434784	-1.79199773	0.735103	-2.43775	0.014779	0.035703
LINC0173	0.68787381	-2.253466861	0.924681	-2.43702	0.014809	0.035757
RETN	13.2788901	-1.437802207	0.590436	-2.43515	0.014886	0.035899
IGHV3-13	105.010166	-1.732582439	0.711616	-2.43472	0.014904	0.035936
SOX21-AS	23.7867487	1.403101608	0.576357	2.434433	0.014915	0.035957
RNU6-128	1.32087041	1.310685786	0.538469	2.434099	0.014929	0.035983
ZIC1	8.37670708	2.530656465	1.039884	2.433594	0.01495	0.036022
CXCL6	323.719844	-1.375000542	0.565015	-2.43357	0.014951	0.036022
RPS8P4	0.48791599	-2.07021114	0.851039	-2.43257	0.014992	0.036114
SNORD11	2.81517186	-1.30101351	0.534919	-2.43217	0.015009	0.036148
BRWD1P	3.19176544	1.348036261	0.554977	2.428994	0.015141	0.036421
TMEM13	3.14209995	1.027896559	0.423362	2.42794	0.015185	0.036517
WNT3A	10.7918594	1.327940608	0.546993	2.427711	0.015194	0.036536
UBL5P2	1.0418368	1.294703884	0.533417	2.427187	0.015216	0.036585
YWHAQP	2.36964127	1.224533623	0.504596	2.426762	0.015234	0.036624
PMCH	4.42541058	-1.384328692	0.57068	-2.42575	0.015277	0.036691
LINC0043	1.79136805	1.112273385	0.458674	2.424976	0.015309	0.036751
TRAV34	1.1721244	-1.325553621	0.546982	-2.42339	0.015376	0.036893
RNU4-9P	1.20172755	-1.665454659	0.68749	-2.42251	0.015414	0.036957
RNU6-342	1.08366741	-1.136150016	0.469808	-2.41833	0.015592	0.037359
RNU2-42F	1.03025802	-1.351569205	0.558996	-2.41785	0.015613	0.037394
DNMT3L	1.13781575	2.354738603	0.973986	2.41763	0.015622	0.037409
TMED7-T	0.51849856	1.644858249	0.68048	2.417205	0.01564	0.037446
IGHV3-35	0.69467447	-2.46305468	1.019199	-2.41666	0.015664	0.037487
TSHR	8.64947613	-1.259719352	0.521403	-2.41602	0.015691	0.037539
SAMD3	80.2015043	-1.006272817	0.416612	-2.41537	0.015719	0.037598
NOX1	92.7252529	-1.556604299	0.644528	-2.41511	0.015731	0.037621
RNA5SP4	0.97611328	1.288526631	0.533646	2.414571	0.015754	0.037666
SUN3	6.37057059	1.04601254	0.433238	2.414405	0.015761	0.037679
IGLV3-21	442.784409	-1.656144741	0.686086	-2.4139	0.015783	0.037726
GPR31	1.92097415	-1.217452564	0.504735	-2.41206	0.015862	0.037881
VAV3-AS	0.60605611	-1.840657354	0.764134	-2.40881	0.016004	0.038172
RNU7-194	1.25875405	-1.467982261	0.609535	-2.40837	0.016024	0.038207
CHRM1	0.47412809	-2.247000523	0.933624	-2.40675	0.016095	0.038358
LINC0151	0.74546689	-1.291116886	0.536559	-2.40629	0.016115	0.038403
MIR657	0.52846975	-1.662324062	0.691319	-2.40457	0.016192	0.038558
MTND1P	0.81919838	1.522335262	0.633196	2.404208	0.016208	0.038581
SEPTIN7F	3.86911943	-1.092787174	0.454854	-2.4025	0.016283	0.038754
CLIC4P3	0.9247552	-1.254041298	0.522078	-2.40202	0.016305	0.038786
BBOX1	9.26624264	1.175499814	0.489434	2.401754	0.016317	0.03881
LINC0064	4.70696835	-1.111248876	0.462787	-2.40121	0.016341	0.038864
BCRP9	1.81363241	3.909518884	1.6286	2.40054	0.016371	0.038928

PDE6A	6.34940679	1.087659173	0.453224	2.399827	0.016403	0.038992
LINC0252	0.88362877	1.476451496	0.615332	2.39944	0.01642	0.039026
PSME2P5	1.04198766	-1.533908916	0.639599	-2.39823	0.016474	0.039132
CYP26C1	1.17337756	-1.293344221	0.539496	-2.39732	0.016516	0.039207
LINC0155	1.05475776	1.356251678	0.566222	2.395264	0.016608	0.0394
POTEF	15.9484344	1.972422074	0.823935	2.393906	0.01667	0.039519
C9orf92	0.87845295	-1.747948393	0.730176	-2.39387	0.016671	0.039519
LINC0197	0.47716985	-1.890470038	0.790051	-2.39284	0.016718	0.0396
H2BS1	3.14485147	1.032577982	0.431588	2.392507	0.016734	0.039628
TRAJ55	0.73630075	-1.933781615	0.808431	-2.39202	0.016756	0.039673
CXCL10	959.007625	1.201404381	0.502311	2.391752	0.016768	0.039694
DYDC2	1.698309	1.437962988	0.601428	2.390914	0.016806	0.039766
PHC2-AS1	4.72662962	1.122788287	0.470005	2.388884	0.0169	0.039963
IGHV4-34	373.201603	-1.614028507	0.67656	-2.38564	0.017049	0.040259
ARHGAP1	0.4576417	-1.98070217	0.830415	-2.3852	0.01707	0.040304
LINC0134	6.37723201	1.081754913	0.453559	2.385035	0.017078	0.040317
COP1P1	1.06152	-1.527547118	0.640537	-2.38479	0.017089	0.040336
RPSAP22	0.61689119	-1.590057471	0.666873	-2.38435	0.017109	0.040369
APOBEC2	3.16547334	-1.309358689	0.549262	-2.38385	0.017132	0.040416
RN7SL619	0.75078441	-1.436044583	0.602543	-2.38331	0.017158	0.040472
IAPP	0.80009529	2.56103111	1.075361	2.381555	0.01724	0.040637
RNA5SP1	1.59101631	2.469280315	1.037276	2.380544	0.017287	0.040736
FAM170B	0.48547447	-1.604120534	0.674459	-2.37838	0.017389	0.040941
NDUFA5P	1.44110766	1.255804344	0.528031	2.378276	0.017394	0.040949
IGHV3-64	28.5393346	-1.905960431	0.801548	-2.37785	0.017414	0.040988
LRRC14B	0.94513301	1.784268376	0.750857	2.376309	0.017487	0.041142
IGHV1-18	565.048357	-1.632019089	0.687061	-2.37536	0.017532	0.041226
KRT7-AS	48.0590364	1.026480713	0.432436	2.373717	0.01761	0.04139
AMD1P1	2.21908998	-1.124461157	0.473819	-2.37319	0.017635	0.041438
LINC0011	4.66461344	-1.113366013	0.469446	-2.37166	0.017708	0.041593
TCF4-AS1	1.07962363	2.328612109	0.982802	2.369361	0.017819	0.041827
PTN	2356.409	1.280688916	0.541529	2.364948	0.018033	0.042249
FAM166C	12.3531435	1.21924269	0.515624	2.364597	0.01805	0.042281
IGHM	2563.46855	-1.390287046	0.587968	-2.36456	0.018051	0.042281
SNORD11	0.46159443	-1.90356918	0.805784	-2.36238	0.018158	0.042498
SCAT1	14.3421271	1.206079519	0.510585	2.362153	0.018169	0.04252
RNU6-530	0.72976454	-2.257608672	0.955992	-2.36153	0.018199	0.042587
MIR4632	0.49572768	-1.507854452	0.638714	-2.36077	0.018237	0.042659
SLC25A24	22.8440651	1.423896221	0.603454	2.359577	0.018296	0.04278
LINC0084	1.06945844	-1.418970799	0.601757	-2.35805	0.018371	0.042915
RELN	111.11416	-1.501436602	0.636945	-2.35725	0.018411	0.042995
PCDHA8	1.14100806	2.24307243	0.951856	2.356526	0.018447	0.043063
RNA5SP2	0.67598866	1.49870092	0.636502	2.354589	0.018543	0.043242
FAM155A	8.33454256	-1.178569788	0.500561	-2.3545	0.018548	0.043249
RPS3AP54	2.76891267	1.270830897	0.539929	2.353698	0.018588	0.043329
LINC0171	4.76422252	1.348305615	0.573448	2.351224	0.018712	0.043552
SLC35F3	18.385032	1.182665147	0.503074	2.350876	0.018729	0.043573
LINC0284	2.37903917	-1.084795078	0.461444	-2.35087	0.01873	0.043573
USP9YP14	1.47861251	-1.597223156	0.679459	-2.35073	0.018737	0.043585
BTNL8	9.14126755	-1.013774874	0.431405	-2.34994	0.018776	0.043657
SEPTIN12	2.34610846	1.195924626	0.509309	2.348132	0.018868	0.04384
GALR3	2.22792912	1.124736585	0.479151	2.347353	0.018907	0.043915

OR7E91P	47.6400799	1.065787272	0.454261	2.346199	0.018966	0.044026
INMT-MII	0.38725621	-1.786113803	0.76142	-2.34577	0.018988	0.044073
NHLH2	11.0580451	1.444262351	0.615749	2.345537	0.019	0.04409
ODF4	4.07027459	1.560019313	0.665107	2.345516	0.019001	0.04409
TRAV5	5.78979331	-1.218314518	0.519638	-2.34455	0.01905	0.044188
RPS3AP4(	1.51010472	-1.372703669	0.58568	-2.34378	0.019089	0.044271
BVES-AS1	18.6047555	-1.342858632	0.573159	-2.34291	0.019134	0.044353
RNU6-167	0.65561256	2.104613018	0.898638	2.342003	0.019181	0.044452
KRT3	1.14966426	1.891224875	0.807617	2.341735	0.019194	0.04448
LCT	1.88915194	1.803940175	0.770588	2.340992	0.019233	0.044556
RPL10P13	2.99700776	1.462028597	0.624543	2.340959	0.019234	0.044556
RNU6-122	0.84661847	-1.819006823	0.777277	-2.34023	0.019272	0.044638
RHOT1P2	53.8533356	-1.090521758	0.466322	-2.33856	0.019358	0.044806
SLC38A3	7.69306548	1.100063558	0.47055	2.337823	0.019396	0.044884
LIMS3	0.84351567	-1.356958289	0.580477	-2.33766	0.019405	0.0449
LINC0221	3.08933244	2.733409449	1.16944	2.337367	0.01942	0.044926
IGHV1OR	10.6153677	-1.440050865	0.61676	-2.33486	0.019551	0.045173
OR7E161I	1.23990727	1.786863636	0.765495	2.334259	0.019582	0.045241
LAPTM4A	1.2688637	1.069559774	0.458242	2.334049	0.019593	0.045259
GAPDHP3	1.10679213	1.291193531	0.553287	2.333676	0.019613	0.045299
MIRLET7	1.23119625	-1.314422272	0.563319	-2.33335	0.01963	0.045334
RN7SL16I	3.23840926	1.082256519	0.463897	2.332968	0.01965	0.045372
SRP14P4	0.57080867	1.896442434	0.813243	2.33195	0.019703	0.045487
FABP5P11	1.03611841	1.435682166	0.615951	2.330837	0.019762	0.045596
LINC0188	0.95951381	-1.557678874	0.669035	-2.32825	0.019899	0.045867
LINC0267	0.88079339	-1.422246301	0.61087	-2.32823	0.0199	0.045867
RNU6-103	1.19920762	-1.325935562	0.570144	-2.32562	0.020039	0.04614
SLC4A4	394.282046	-1.052552788	0.45302	-2.32341	0.020157	0.046359
LUZP2	131.215152	1.096151973	0.471863	2.323032	0.020177	0.046397
BAGE2	12.6241385	3.389300879	1.460416	2.320778	0.020299	0.046637
PTPN20	10.9177452	1.172238104	0.505127	2.320679	0.020304	0.04664
CAMTA1-	0.46648907	-1.668316418	0.718931	-2.32055	0.020311	0.046652
FOLR3	10.5241146	-1.485493447	0.640458	-2.31942	0.020372	0.046761
CXCR3	60.6056536	-1.097992415	0.473422	-2.31927	0.020381	0.046776
OOEP	8.67955169	1.553121794	0.669762	2.318915	0.0204	0.046802
LINC0121	2.4466343	1.526863566	0.658814	2.317594	0.020471	0.04694
NPFFR2	19.2192405	-1.171548293	0.505941	-2.31558	0.020581	0.047165
SNORA30	0.91164563	1.143948592	0.494129	2.31508	0.020609	0.047211
MIR921	0.63388469	1.986728616	0.858186	2.315032	0.020611	0.047212
CGB3	0.70867775	2.222335135	0.960076	2.314749	0.020627	0.047231
MIR4804	0.48976719	-1.711278185	0.739441	-2.31429	0.020652	0.04727
RAD17P1	5.16027839	-1.895285916	0.819102	-2.31386	0.020675	0.047316
UNQ6494	4.45072492	-1.167395155	0.504525	-2.31385	0.020676	0.047316
MIR3684	0.67905056	1.40254053	0.606212	2.313614	0.020689	0.047332
SMIM15P	1.59633499	1.169964442	0.505697	2.313567	0.020691	0.047334
TRGV5P	3.64302162	-1.201872584	0.519709	-2.31259	0.020745	0.047421
CLCA2	2455.24027	1.225563457	0.531307	2.306697	0.021072	0.048068
ANKRD2(	583.299823	-1.009120307	0.438166	-2.30305	0.021276	0.048457
CCT5P1	1.72515745	1.110608477	0.482254	2.302955	0.021281	0.048465
ARAP1-A:	0.84034131	-1.301886358	0.565367	-2.30273	0.021294	0.048477
TMEM35A	102.907446	-1.178285844	0.51172	-2.3026	0.021302	0.048485
RN7SKP2	1.29112629	-1.372782163	0.596191	-2.30259	0.021302	0.048485

IGKV3-20	2193.21726	-1.325870053	0.575876	-2.30236	0.021315	0.048506
DTNB-AS	17.194406	-1.426022629	0.619593	-2.30155	0.021361	0.0486
ATP5F1CI	1.61657877	1.09601283	0.47628	2.301195	0.021381	0.048641
BCAN	5.07786308	1.135134682	0.493402	2.300627	0.021413	0.048696
MTND2P2	3.53088604	1.042771052	0.453358	2.300104	0.021442	0.048745
FOXJ1	344.641055	1.048248013	0.45641	2.296726	0.021634	0.049163
SNTG1	36.9210004	1.475554306	0.643152	2.294253	0.021776	0.049437
HTR1D	10.2406899	-1.000636147	0.436374	-2.29307	0.021844	0.049566
LINC0259	0.77349274	-1.238519722	0.540436	-2.2917	0.021923	0.04972
STK19B	1.67935802	-1.203055721	0.525212	-2.29061	0.021986	0.049842
FOXA3	10.8126285	1.205901485	0.52648	2.290497	0.021992	0.049843
DNAJA1P	1.16688607	1.209951475	0.528827	2.28799	0.022138	0.050121
SNORD42	1.74193933	-1.099590849	0.480798	-2.28701	0.022195	0.050238
RAD51AP	10.0895556	1.314583333	0.575225	2.285339	0.022293	0.05043
CD5L	0.6777313	-1.584281804	0.693391	-2.28483	0.022323	0.050492
GAPDHP3	1.76042104	1.351689657	0.591767	2.284158	0.022362	0.050568
RNU7-29F	1.26911993	-1.081620728	0.473566	-2.28399	0.022372	0.050585
RNU6-48F	0.93153128	1.119986196	0.490397	2.283837	0.022381	0.050601
AQP5	26.9986567	1.53358803	0.671672	2.283241	0.022416	0.050662
SPATA3-A	1.4582597	1.560988989	0.683816	2.28276	0.022445	0.050721
CCR12P	0.4311338	-2.294242365	1.005529	-2.28163	0.022511	0.050863
LMO1	4.70791583	-1.573308604	0.689994	-2.28018	0.022597	0.051033
RNU4-2	20.8588086	-1.160510467	0.509454	-2.27795	0.02273	0.051276
PLA2G2C	2.26771675	-1.045434255	0.459164	-2.27682	0.022797	0.051403
GTF3AP1	0.51159577	1.829473994	0.803569	2.276687	0.022805	0.051416
C21orf62	1.71104435	-1.613822995	0.709517	-2.27454	0.022934	0.051683
IMPDH1P	1.47895934	-1.153682214	0.507596	-2.27284	0.023036	0.051885
CPS1	82.309962	1.094814168	0.482001	2.271396	0.023123	0.052052
LINC0210	0.39930938	-2.258268241	0.994529	-2.27069	0.023166	0.052138
PRELID3I	1.17939926	1.84853497	0.814577	2.269318	0.023249	0.052302
BCAP31P2	1.13448408	1.229726801	0.542459	2.266949	0.023393	0.052568
GOT2P7	3.81706592	-1.225568954	0.540763	-2.26637	0.023429	0.052638
RNA5SP3	0.51383066	-1.75205405	0.773547	-2.26496	0.023515	0.052813
RNVU1-34	0.60956047	1.940328876	0.857126	2.263761	0.023589	0.052944
GNAT2	1.89186696	1.014993024	0.448385	2.263662	0.023595	0.052953
HLA-Z	0.58617123	1.429642872	0.631572	2.263626	0.023597	0.052953
C7orf65	1.19247141	1.440201182	0.636284	2.263458	0.023607	0.052966
SNORD11	0.51330864	-1.810318363	0.799893	-2.2632	0.023623	0.052992
LINC0173	1.25217544	1.244995393	0.550182	2.26288	0.023643	0.053027
GOSR2-D	3.17552967	1.000625715	0.442288	2.262387	0.023674	0.05309
ST8SIA2	9.15220174	1.719108821	0.760061	2.261803	0.02371	0.053157
GPR25	10.2419982	-1.246354739	0.551285	-2.26082	0.023771	0.053274
P2RX6P	1.08493144	-1.854917183	0.820822	-2.25983	0.023832	0.053401
OR2L13	0.56524236	-1.76425045	0.780749	-2.25969	0.023841	0.053416
LINC0068	5.75695228	-1.054308553	0.466768	-2.25874	0.023899	0.053538
RN7SKP24	6.34520348	1.357291321	0.601048	2.258207	0.023933	0.053593
MUC2	334.900412	1.116054582	0.494363	2.25756	0.023973	0.053659
CDKN2A	606.997244	1.10929601	0.491461	2.257139	0.023999	0.053708
LINC0264	1.51114003	-1.006915795	0.446225	-2.25652	0.024038	0.053779
PLA2G2F	1697.4794	1.170141894	0.519184	2.253809	0.024208	0.05413
MS4A3	4.58518247	-1.299458719	0.576745	-2.25309	0.024253	0.054197
SPIB	76.7867126	-1.372465333	0.609452	-2.25196	0.024325	0.054345

NRG3	4.90649571	-1.326789771	0.589488	-2.25075	0.024401	0.054487
POTEI	2.39849071	2.048542544	0.910293	2.250422	0.024422	0.054524
CFAP100	1.16852221	1.818097825	0.808153	2.249695	0.024468	0.054617
EGOT	9.72244232	-1.071917822	0.476565	-2.24926	0.024496	0.054674
HSPB2	1.31383415	-1.063641584	0.4729	-2.24919	0.0245	0.054678
EFNA2	23.1535186	1.267598535	0.564049	2.247321	0.02462	0.054926
LNCARSF	0.58690835	-1.556729632	0.693041	-2.24623	0.024689	0.055045
H3C12	4.56590702	1.127055256	0.501792	2.246063	0.0247	0.055063
LINC0238	30.3924996	-1.078351716	0.480134	-2.24594	0.024708	0.055076
SMILR	24.1561798	1.030514403	0.459112	2.244583	0.024795	0.055245
IGLV7-43	82.6949498	-1.505871339	0.671369	-2.24299	0.024898	0.055443
RN7SL21	0.77320286	1.47848599	0.659234	2.242734	0.024914	0.055469
RSL24D1I	1.24294484	1.124353531	0.501565	2.241689	0.024981	0.055604
C5orf60	1.01296124	1.139836016	0.508481	2.241649	0.024984	0.055605
ERVFRD-	1.91429998	1.722350003	0.768365	2.241579	0.024989	0.055607
ANP32BP	0.38584403	-1.81346404	0.809017	-2.24156	0.02499	0.055607
HPN	28.3287955	1.146516998	0.511667	2.240747	0.025042	0.055715
SERPINB	634.716525	1.72132946	0.768241	2.240612	0.025051	0.055729
PKP4-AS1	3.46320777	1.041857898	0.465083	2.240153	0.025081	0.05578
FEM1AP2	0.91651033	2.038789115	0.910183	2.239977	0.025092	0.055801
TMEM30I	0.43231903	1.611258586	0.71956	2.239226	0.025141	0.055894
NLRP2	522.262282	1.370269058	0.612778	2.236161	0.025341	0.056308
LKAAEAI	2.64091387	1.044927981	0.467598	2.234672	0.025439	0.056478
RNA5SP2	0.77093822	1.812090266	0.81133	2.233482	0.025517	0.056637
RNU6-100	1.26899196	-1.35251016	0.605833	-2.23248	0.025583	0.056768
ENPP7	1.09266753	-1.296636993	0.581208	-2.23094	0.025685	0.056969
POM121L	0.66958031	-1.24945322	0.560268	-2.2301	0.025741	0.057076
TCP11	7.96940188	1.028319924	0.461124	2.230031	0.025745	0.057081
BTG4	0.95934816	1.449836418	0.651121	2.226678	0.025969	0.057529
NCAM2	56.988219	-1.223119773	0.549691	-2.22511	0.026074	0.057731
FAM83A-	0.77354083	1.463723062	0.658098	2.22417	0.026137	0.057855
TRAJ23	0.61803581	-1.658404234	0.745652	-2.2241	0.026142	0.05786
IGHV3-21	1268.66659	-1.570109396	0.706371	-2.22278	0.02623	0.058014
IGF2BP2	572.113458	1.0713206	0.482012	2.222603	0.026243	0.058025
SLCO1B1	2.61155827	2.406493723	1.082795	2.222482	0.026251	0.058038
GABARA	0.87040059	-1.239587657	0.557893	-2.22191	0.02629	0.058119
LINC0172	1.39512175	-1.650434016	0.743029	-2.22122	0.026336	0.058194
IGLV3-25	602.617018	-1.513731068	0.681668	-2.22063	0.026376	0.058268
IGLC1	1.32659464	-1.534636278	0.692227	-2.21696	0.026626	0.058751
TFAP2B	55.7548152	1.374706806	0.620184	2.21661	0.02665	0.058787
CNNM1	65.9992851	-1.045891088	0.471975	-2.21599	0.026692	0.058849
GABRR3	4.2765123	1.58025298	0.713163	2.215836	0.026703	0.058867
ELFN2	13.3504575	1.007069123	0.454974	2.213466	0.026866	0.059177
IRX4-AS1	1.51276432	2.513074534	1.136272	2.211685	0.026988	0.059416
IFNL2	0.70301586	2.212861471	1.001018	2.210611	0.027063	0.059547
C17orf64	2.55275057	1.265076079	0.572529	2.209628	0.027131	0.059681
FFAR3	4.4200471	-1.331679311	0.602948	-2.20862	0.027201	0.05982
MRPS9-A	1.37646831	1.258521971	0.570232	2.207036	0.027312	0.060051
RNU6-26F	2.01229621	-1.141941821	0.517512	-2.2066	0.027342	0.060102
NF1P6	1.25620608	2.614462177	1.185569	2.205239	0.027437	0.060301
ZSWIM5P	0.56316974	-2.806886509	1.273125	-2.20472	0.027474	0.060375
DNAJB6P	0.72912089	-1.387811563	0.629533	-2.20451	0.027489	0.060402

F11	1.28955455	-1.359875499	0.617007	-2.20399	0.027525	0.060472
SKA2P1	1.56449225	1.029205278	0.467452	2.201733	0.027684	0.060767
LINC0228	3.90856365	-1.179853598	0.536065	-2.20095	0.027739	0.060872
LINC0038	1.85274783	-1.369597682	0.622425	-2.20042	0.027777	0.060949
C4orf51	1.49169779	1.905894598	0.866202	2.200288	0.027786	0.060959
LINC0227	0.948919	1.840695014	0.83673	2.199868	0.027816	0.061007
RN7SL38	0.37761822	-1.698969191	0.772804	-2.19845	0.027917	0.06121
LINC0185	1.1712077	2.127921428	0.969115	2.195737	0.028111	0.061543
IGHG3	5968.41515	-1.25173223	0.570373	-2.19459	0.028193	0.061701
IL22RA2	14.7065916	-1.215107899	0.553879	-2.19381	0.028249	0.061811
TRAV8-2	10.5817904	-1.143227607	0.521237	-2.1933	0.028286	0.061882
GOLGA6I	0.93424901	2.240440666	1.021782	2.19268	0.02833	0.061968
BCAP31P	0.61520154	1.910905383	0.871518	2.192616	0.028335	0.061972
C7orf33	0.81193102	2.200931423	1.004523	2.191021	0.02845	0.062197
MUC5AC	515.662832	1.62886511	0.743571	2.190597	0.028481	0.062247
ZNF32-AS	0.65836252	1.559187608	0.712445	2.188504	0.028633	0.062512
MIR3136	1.44824579	1.110104927	0.508611	2.182621	0.029064	0.063362
OR7E100I	1.13754215	1.138542859	0.521758	2.182128	0.0291	0.063418
SORCS3	3.04170048	-1.610103375	0.738184	-2.18117	0.029171	0.063539
RN7SKP2	0.49879512	1.598246255	0.733386	2.179271	0.029312	0.063799
IGHG2	4258.48894	-1.164938214	0.534653	-2.17887	0.029342	0.063859
RPSAP55	0.46284972	1.776946413	0.815845	2.178044	0.029403	0.063953
LINC0159	0.84106852	-1.500010018	0.688819	-2.17765	0.029432	0.064003
TRIM10	1.37452796	1.870707299	0.859342	2.176906	0.029488	0.064101
NXPH2	0.83696995	2.456821018	1.12895	2.176201	0.02954	0.064206
CCBE1	17.1736503	-1.04269236	0.479246	-2.17569	0.029578	0.064282
MCRIP2P	2.31401088	1.460784308	0.671666	2.174867	0.02964	0.064408
LINC0176	8.17349846	1.110270503	0.510588	2.174494	0.029668	0.064449
OR8R1P	2.08946349	1.259179901	0.579172	2.174103	0.029697	0.064501
MTHFD2I	3.44165606	-1.584350231	0.728794	-2.17393	0.02971	0.064523
ANKRD26	0.79962887	-2.031193104	0.934393	-2.17381	0.029719	0.064535
OR2C1	0.59403302	-1.572334026	0.723326	-2.17376	0.029723	0.064535
RPS15P5	1.7771711	1.093559066	0.50364	2.171312	0.029908	0.064894
CXorf49	2.07757755	1.492291545	0.687818	2.169604	0.030037	0.065146
GJA1P1	2.00849805	1.273287216	0.586964	2.169277	0.030062	0.065194
RPL15P20	0.73235272	1.322241705	0.60989	2.168	0.030159	0.065377
PAPOLB	0.48829014	-1.954715096	0.901627	-2.16799	0.03016	0.065377
TTC9B	0.86337486	1.684713469	0.777867	2.165811	0.030326	0.065688
KRT20	3302.67082	1.345936541	0.621511	2.165589	0.030343	0.065715
IGHJ2	0.64441374	-1.98091031	0.915366	-2.16406	0.030459	0.065895
ACRP1	1.25757164	-1.113552105	0.51458	-2.164	0.030464	0.065897
LINC0215	1.30444347	1.846263856	0.853493	2.163185	0.030527	0.066015
TSEN15P	0.67230967	1.203015568	0.556182	2.162988	0.030542	0.066036
BTBD7P1	12.511395	1.091293774	0.504674	2.162375	0.030589	0.066121
ANAPC1P	0.94136583	1.196052372	0.553361	2.161432	0.030662	0.06626
CYCSP23	0.98333305	-1.354412879	0.626689	-2.16122	0.030678	0.066278
CLDN14	10.2535831	1.230738577	0.569967	2.159315	0.030826	0.066555
GAPDHP3	1.36474323	1.361831347	0.631068	2.15798	0.030929	0.066732
LINC0054	15.5356806	1.212535022	0.562013	2.157487	0.030968	0.0668
CFHR4	1.67296362	1.32589674	0.614939	2.156144	0.031072	0.066981
TRAJ48	0.45585566	-1.966771783	0.912204	-2.15607	0.031078	0.066988
CGB8	0.93209685	2.197451471	1.019639	2.155127	0.031152	0.067117

CYP4F62I	2.38802862	2.062241904	0.957512	2.15375	0.03126	0.067307
TRAV12-2	12.2978356	-1.172243867	0.544321	-2.15359	0.031272	0.067317
EIF4EBP2	0.56425742	-1.324382093	0.615249	-2.15259	0.031351	0.067462
RNU6-133	0.45960383	1.913849594	0.889506	2.151586	0.03143	0.067613
PPIAP40	1.42109923	1.055356552	0.490623	2.151053	0.031472	0.067668
PCK1	3.84711119	-1.346249227	0.626739	-2.14802	0.031712	0.06809
POSTN	6291.21716	1.116440279	0.520551	2.144727	0.031975	0.068593
TYRP1	16.9809643	-1.19224371	0.556544	-2.14223	0.032175	0.068981
PIMREGP	0.47297387	-1.710775189	0.799172	-2.14068	0.032299	0.069198
SCTR-AS1	1.3125284	-1.389177614	0.649211	-2.13979	0.032371	0.069328
OR10V3P	0.52754317	-1.821481578	0.851408	-2.13938	0.032405	0.069388
CPA6	102.052337	-1.193175341	0.557876	-2.13878	0.032453	0.06946
MIR4518	2.076874	-1.514763183	0.708672	-2.13747	0.03256	0.069634
IGLC5	0.45387926	-2.330616687	1.090413	-2.13737	0.032568	0.069639
SYP-AS1	0.89060602	1.327138222	0.621578	2.135112	0.032752	0.069989
LINC0139	1.65684761	1.35124238	0.63288	2.135069	0.032755	0.069991
LINC0182	2.88675714	-1.184597198	0.555256	-2.13343	0.03289	0.070235
MIR563	0.49319691	1.871007933	0.87723	2.13286	0.032936	0.070328
SPATC1	9.21717371	-1.059299131	0.496751	-2.13246	0.032969	0.070386
RPSAP43	17.4401987	-1.28991025	0.60494	-2.13229	0.032983	0.070409
MYBPHL	3.35865887	1.086677817	0.509665	2.132143	0.032995	0.070423
LINC0278	11.5032651	1.204816904	0.56542	2.130836	0.033103	0.070634
RN7SL491	0.5751823	-1.363679249	0.640023	-2.13067	0.033116	0.07065
FAR2P1	152.472482	1.817374007	0.853885	2.12836	0.033307	0.070996
OR7E62P	32.6505845	1.098498832	0.516277	2.127733	0.033359	0.071088
CYP4F22	568.565149	1.014150572	0.476655	2.12764	0.033367	0.071098
RN7SL337	0.50448623	-1.668299416	0.784245	-2.12727	0.033398	0.071151
DLX1	12.7186238	1.099336133	0.516902	2.126781	0.033438	0.071219
EVX1-AS	102.025503	-1.152044825	0.542184	-2.12482	0.033601	0.071548
SERTM2	25.31232	-1.415663195	0.666455	-2.12417	0.033656	0.071645
LINC0182	1.1364202	-1.370834811	0.646159	-2.12151	0.033879	0.072056
LINC0248	1.06430463	1.872162914	0.883004	2.120221	0.033987	0.072231
TRAJ41	0.75724337	-1.495944077	0.705634	-2.12	0.034006	0.072264
CCL21	388.257069	-1.382260026	0.652027	-2.11994	0.034011	0.072269
PCDHA4	20.9597846	1.296673753	0.611687	2.119833	0.03402	0.072282
LINC0134	9.34845745	1.011778735	0.477856	2.117329	0.034232	0.072662
LINC0179	0.3999041	-1.882776291	0.889437	-2.11682	0.034275	0.072742
GLP1R	5.14840002	1.392357364	0.65854	2.11431	0.034489	0.073153
IL17C	10.0157374	-1.098074364	0.519361	-2.11428	0.034491	0.073153
RNU6-436	1.24630547	-1.266472905	0.599582	-2.11226	0.034664	0.07349
FREM3	1.80615916	-1.482055183	0.701714	-2.11205	0.034682	0.073522
IGLJCOR1	0.89704322	2.045578792	0.968624	2.111839	0.0347	0.073547
ADAMDE	318.640206	-1.059163224	0.501827	-2.11061	0.034805	0.073745
TRAJ25	0.4000367	-1.923753485	0.911693	-2.11009	0.034851	0.073809
RPS3AP15	1.8723979	1.919902797	0.910211	2.109295	0.034919	0.073909
H1-1	9.24440645	1.56236691	0.740794	2.109044	0.034941	0.073948
LINC0259	0.70141338	-1.416247901	0.671652	-2.1086	0.034979	0.074009
RPL5P12	0.73595341	1.481874999	0.703456	2.106564	0.035155	0.074322
FKBP6	0.60554256	2.13581359	1.01474	2.104789	0.03531	0.074599
TRAJ5	1.72826762	-1.417522383	0.673794	-2.10379	0.035397	0.074743
UPK3A	1216.47503	-1.082250647	0.514566	-2.10323	0.035446	0.074802
CYP19A1	76.2259676	-1.519481082	0.7225	-2.10309	0.035458	0.074822

LINC0089	2.11883846	2.106779614	1.002085	2.102396	0.035519	0.074896
CCDC144.	4.54443643	-1.144362645	0.544363	-2.1022	0.035535	0.074908
MIR1245A	17.5011137	-1.177285708	0.560078	-2.102	0.035553	0.074912
LINC0051	1.15704296	-1.447394665	0.688694	-2.10165	0.035584	0.074951
RNA5SP2(	0.92552731	-1.40675128	0.66956	-2.10101	0.03564	0.075057
RPL32P20	0.96806877	1.955186741	0.930941	2.100225	0.035709	0.075163
PROKR1	1.17440158	1.5339507	0.730771	2.099085	0.035809	0.075329
SOX2	582.217957	1.254171355	0.598027	2.097181	0.035978	0.075618
TSPEAR	3.17207716	-1.062424596	0.5072	-2.09468	0.036199	0.076024
E2F6P4	0.68734961	2.33149791	1.113272	2.094275	0.036236	0.076081
BSN-DT	3.17368998	1.119239871	0.534482	2.094063	0.036254	0.076114
LINC0051	0.70889466	1.49740862	0.715216	2.093645	0.036292	0.076179
VSTM2L	62.6277604	1.020407706	0.487662	2.092449	0.036398	0.076371
AQP12A	0.85682237	2.785079481	1.331287	2.09202	0.036437	0.076444
ACTBP7	0.91542141	1.401875993	0.670171	2.091817	0.036455	0.076463
IGHV3-20	93.164237	-1.633773785	0.781332	-2.09101	0.036527	0.076593
EIF5P1	0.97619053	1.012157246	0.484067	2.090946	0.036533	0.076594
UBE2Q2P	0.68145336	-1.653279146	0.790966	-2.0902	0.0366	0.07672
CFAP97D	0.55079317	-1.269572559	0.607612	-2.08945	0.036668	0.076837
OR2W6P	2.02504611	1.617682224	0.774424	2.088884	0.036718	0.076916
RNU6ATA	1.34744263	-1.101294004	0.527281	-2.08863	0.036741	0.076958
PRSS57	0.75302853	-1.545469657	0.740056	-2.08831	0.036769	0.07701
RNA5SP9(	0.66530668	1.648704383	0.78986	2.087338	0.036858	0.077175
SINHCAF	0.72243399	1.214176597	0.582254	2.085305	0.037042	0.0775
H1-5	11.2898234	1.045977161	0.501942	2.08386	0.037173	0.077735
CTB-178M	2.93444783	1.012218641	0.485955	2.082947	0.037256	0.077874
H2BC16P	1.23912349	1.561399525	0.749766	2.082515	0.037295	0.077944
GOLGA5I	1.38899867	-1.26949499	0.60966	-2.0823	0.037315	0.077979
OR2AG2	0.57360799	1.930910308	0.927528	2.08178	0.037363	0.078058
LDHAL6A	7.76081189	2.116516599	1.016818	2.08151	0.037387	0.078103
CPB1	4.53616238	-1.326527193	0.63757	-2.0806	0.037471	0.078237
SOHLH1	1.28192837	2.16990148	1.043438	2.079568	0.037565	0.078405
TAS2R3	1.35047199	1.086474514	0.522681	2.078656	0.037649	0.078556
KRT8P51	0.64768214	2.106670161	1.01364	2.078322	0.03768	0.078591
SPECC1P1	0.29497981	-1.563238357	0.752172	-2.0783	0.037682	0.078591
RFESDP1	1.31535462	1.468813521	0.707042	2.077406	0.037764	0.078728
LINC0064	34.7191436	1.689232446	0.813247	2.077145	0.037788	0.078765
SHISA5P1	1.35843621	-1.167739319	0.562233	-2.07697	0.037805	0.078793
MIR606	3.31951105	1.098906247	0.529292	2.076182	0.037877	0.078924
SSX5	0.7392346	-1.823923654	0.878748	-2.07559	0.037932	0.07901
ULK4P3	0.91056846	-1.277784801	0.615724	-2.07526	0.037963	0.079055
H2AC5P	0.91043242	1.448307894	0.698452	2.073597	0.038117	0.079338
DNTT	0.48283078	-1.846658674	0.89065	-2.07338	0.038137	0.07937
MMP3	443.765149	-1.229985378	0.593297	-2.07313	0.03816	0.079397
MEIS1-AS	0.358343	-1.665042893	0.803231	-2.07293	0.038179	0.07943
IGKV3OR	6.74221005	-1.513282765	0.730064	-2.07281	0.03819	0.079438
MIR3193	1.25949572	-1.649219097	0.796389	-2.07087	0.038371	0.079769
SSMEM1	0.56446971	-1.131471078	0.546524	-2.07031	0.038424	0.079858
MIR3192	1.27559021	1.077117085	0.520616	2.068928	0.038553	0.080079
TRBV4-2	7.15513482	-1.182661142	0.573164	-2.06339	0.039076	0.081061
LINC0086	0.73947039	1.51098032	0.733007	2.061346	0.03927	0.081416
SPATA4	2.32771927	-1.388300265	0.673556	-2.06115	0.039289	0.081441

PIANP	32.6702414	-1.07441076	0.522122	-2.05778	0.039612	0.082006
HMX2	1.3732608	2.01216795	0.978256	2.056893	0.039697	0.082147
DPPA2P4	1.29430141	-1.60300139	0.779633	-2.0561	0.039773	0.082284
SUGT1P4-	0.58381756	-1.358717447	0.661199	-2.05493	0.039886	0.08251
GPR182	1.08796145	-1.542536715	0.750871	-2.05433	0.039944	0.082595
FCAR	47.5392264	-1.078817872	0.525244	-2.05394	0.039982	0.08266
C19orf67	0.8203616	1.694056474	0.825339	2.052559	0.040115	0.082845
RSPH6A	2.1085264	1.266137092	0.616964	2.052204	0.04015	0.082898
RNU6-101	1.09495585	-1.104199977	0.538219	-2.05158	0.040211	0.08299
RNU4-36F	0.81465751	1.320845596	0.645275	2.046949	0.040663	0.083784
EDRF1-AS	0.48524118	-1.378498133	0.674372	-2.04412	0.040942	0.084266
GUCY1B2	57.7392689	1.171712645	0.573691	2.042412	0.041111	0.084571
PSMC1P5	0.92412514	1.049492282	0.51421	2.040979	0.041253	0.084803
RARS1P1	1.4087783	1.181730375	0.579406	2.039554	0.041395	0.085062
DNAJB6P	1.15195414	1.261601138	0.618844	2.038641	0.041486	0.085199
SLC25A5I	0.90497386	1.079421653	0.529586	2.038238	0.041526	0.085268
FUT6	90.5114359	1.157075676	0.568123	2.036664	0.041684	0.085512
LINC0252	0.53856787	1.965839751	0.965675	2.035716	0.041779	0.085686
LINC0139	3.02401933	1.671696562	0.822021	2.033643	0.041988	0.086034
UGT8	116.671389	1.191709688	0.586092	2.033315	0.042021	0.086095
LINC0277	1.13840811	1.707667802	0.840181	2.0325	0.042103	0.086227
RPS27P16	1.27796533	1.321505447	0.650412	2.031798	0.042174	0.086352
LINC0108	1.24450498	-1.29009101	0.635208	-2.03097	0.042258	0.086488
RNVU1-24	3.12347871	-1.163668321	0.572975	-2.03092	0.042263	0.086489
GVINP2	0.49441873	-1.486132993	0.732496	-2.02886	0.042472	0.086852
WFDC5	17.6082109	1.118462226	0.55134	2.028625	0.042497	0.086894
GAPDHP6	0.68947732	1.14476668	0.564534	2.027808	0.04258	0.087014
PNMA8A	118.938091	-1.0252225	0.505676	-2.02743	0.042618	0.087071
KRT18P28	0.87723653	1.112801906	0.549252	2.026031	0.042762	0.087341
RD3	3.84906984	-1.212397622	0.598536	-2.02561	0.042805	0.087408
SNORD11	1.29469449	-1.066008122	0.526864	-2.02331	0.043042	0.087832
LINC0197	8.69821349	1.268807578	0.627163	2.02309	0.043064	0.087871
SPAG6	6.30706979	1.016283969	0.502362	2.023011	0.043072	0.08788
RNU6-554	0.51330986	1.393431264	0.689593	2.020658	0.043315	0.088272
MIR4504	0.61864331	1.34673817	0.666839	2.019585	0.043426	0.08847
FXYD4	117.094308	1.212861139	0.601273	2.017155	0.043679	0.088918
LINC0138	1.03368155	-1.278765766	0.634039	-2.01686	0.04371	0.088966
AKR1C2	11000.0821	1.013316699	0.502518	2.01648	0.04375	0.089032
ANKS4B	0.75183075	1.264482674	0.627342	2.015618	0.04384	0.089163
RPS3AP14	1.21030959	-1.108905361	0.550523	-2.01428	0.04398	0.08942
IL12B	6.63004339	-1.022953919	0.508306	-2.01248	0.04417	0.089774
GPR61	0.97241229	1.208855832	0.600899	2.011747	0.044247	0.089893
CARM1P1	6.31600656	2.628931076	1.307183	2.011143	0.04431	0.09
ARL13A	0.65168778	1.893227139	0.941661	2.010519	0.044376	0.090104
USP8P2	1.12155803	1.728232404	0.859877	2.00986	0.044446	0.090231
LINC0150	11.7293779	1.06562547	0.530262	2.009622	0.044471	0.090271
RPS4XP8	0.77638758	1.481646344	0.737389	2.009315	0.044504	0.090318
NCAM1-A	0.74410789	-1.582038783	0.787516	-2.0089	0.044548	0.090393
ZBBX	1.8512888	1.456627745	0.725701	2.0072	0.044728	0.090668
TRBV6-4	1.22794694	-1.517327893	0.756503	-2.00571	0.044887	0.090921
RPS29P5	0.81451012	1.071081488	0.534182	2.005086	0.044954	0.091034
LINC0177	0.37778222	-1.832150987	0.91405	-2.00443	0.045024	0.091153

RNF126P1	2.56506247	1.709211657	0.852934	2.003921	0.045079	0.091226
SNRPD2P	0.35880954	-1.394020892	0.696052	-2.00275	0.045204	0.091419
CDK8P2	1.0797462	1.263203208	0.631988	1.998777	0.045633	0.092141
OR2I1P	1.25086471	1.386217444	0.69389	1.997749	0.045744	0.092343
BHMT	1424.23146	1.229333154	0.615724	1.996565	0.045872	0.092556
LINC0241	0.63470863	-1.663034149	0.833	-1.99644	0.045886	0.092577
RPL23AP:	0.7715457	1.227662435	0.615421	1.994832	0.046061	0.092876
LINC0213	1.5426679	1.132214759	0.567747	1.994226	0.046127	0.092963
DDX11L9	1.21812619	1.178262138	0.590962	1.993802	0.046174	0.093034
FAM133A	41.0889877	1.364650834	0.685484	1.990786	0.046504	0.093591
GUCA2A	8.29628767	1.748500637	0.878722	1.989823	0.04661	0.093766
MRPS36P	1.22493245	-1.141789706	0.573885	-1.98958	0.046637	0.093797
ZNF885P	0.4991087	1.575740061	0.792013	1.989539	0.046642	0.093799
UBE2Q2P	0.63991187	-1.19725554	0.601856	-1.98927	0.046671	0.093846
ENTHD1	6.15617804	-1.132182281	0.569184	-1.98913	0.046687	0.093866
A2ML1-A	0.68509959	-1.085184016	0.546171	-1.98689	0.046934	0.094278
TRBV1	0.33810177	-1.813143217	0.912933	-1.98606	0.047026	0.094447
MIR572	0.83073426	1.435494673	0.723176	1.984986	0.047146	0.094635
IGHV3-62	3.98816192	-1.825476413	0.91968	-1.9849	0.047155	0.094636
HABP2	13.1199823	1.326963165	0.669024	1.983431	0.047319	0.094915
TET2-AS1	0.87914493	-1.384008752	0.697791	-1.98341	0.047321	0.094915
SRP14P3	1.04760884	1.125809537	0.567958	1.982204	0.047456	0.095119
OR56A5	0.73889976	2.021238348	1.020044	1.981522	0.047533	0.095237
MACC1-A	0.5129237	-1.7874479	0.902651	-1.98022	0.047679	0.095467
HAVCR1	39.3266669	1.233214072	0.623111	1.979125	0.047802	0.095651
CDRT15P	0.46299103	1.622745815	0.820201	1.978474	0.047875	0.095774
RNU6-502	0.4105667	-1.596257909	0.807441	-1.97693	0.048049	0.096074
RSL24D1I	1.30671593	1.162618778	0.588237	1.976446	0.048104	0.096176
TSIX	5.43866857	-2.01259875	1.018652	-1.97575	0.048183	0.096304
TPPP2	2.55655481	1.228370556	0.622396	1.973614	0.048426	0.096677
MIR4639	1.09030929	1.066327742	0.540788	1.971804	0.048632	0.097025
RNU105B	0.88007837	-1.102013372	0.558965	-1.97152	0.048664	0.097073
RHCG	226.964713	1.075360879	0.545545	1.971168	0.048705	0.097139
SLITRK5	21.8956535	1.340205494	0.679925	1.971107	0.048712	0.097145
OR7E145I	0.7767688	2.039040473	1.035101	1.969895	0.04885	0.097382
CLDN10-/	0.69656674	1.7306049	0.879532	1.967642	0.049109	0.097826
PYDC1	0.64913685	2.105432101	1.070162	1.967396	0.049138	0.09785
NDUFB10	2.53792565	1.284103742	0.652731	1.967278	0.049151	0.097869
TUBB8P7	2.90163825	1.174379007	0.597244	1.966329	0.049261	0.098047
TRPC5OS	0.75332499	1.138493773	0.579231	1.965525	0.049354	0.098216
LINC0105	0.61246494	-1.553870488	0.790679	-1.96524	0.049387	0.09825
RPS3AP37	2.45225268	1.0698051	0.544463	1.96488	0.049428	0.098308
HMGB3P(	0.6542522	1.512186483	0.769819	1.964341	0.049491	0.098392
GLYATL2	4.71342603	-1.101100368	0.561168	-1.96216	0.049744	0.098832
PARM1-A	6.28128191	1.365693186	0.696123	1.961857	0.049779	0.098886
TMEM252	0.42988429	-1.570438887	0.800551	-1.9617	0.049798	0.098906
