# Supplementary material for: Identification of Potential Biomarkers for Progression and Prognosis of Bladder Cancer by Comprehensive Bioinformatics Analysis
Source: J Oncol. 2022 Apr 19;2022:1802706. doi: 10.1155/2022/1802706 (PMC9042640; doi:10.1155/2022/1802706)
Supplement: Supplementary Materials — Supplementary Figure 1: WGCNA analysis of the TCGA dataset. Supplementary Figure 2: WGCNA analysis of the GSE133624 dataset. Supplementary Figures 3–7: clinical relevance of SMYD2, GAPDHP1, CILP, ATP1A2, and THSD4. Supplementary Table 1: primer sequences in the study. Supplementary Table 2: DEGs in the TCGA dataset. Supplementary Table 3: DEGs in the GSE133624 dataset. Supplementary Table 4: DEGs coexisting in the TCGA and GSE133624 datasets. Supplementary Table 5: feature genes were selected with the SVM-RFE algorithm. Supplementary Table 6: the correlation between the characteristic genes and immune cells. Supplementary Table 7: single-gene GSEA for prognostic genes. [file 1802706.f1.zip › 1802706.f1/Supplementary Table 4.pdf]

| Brown module gene(TCGA) | Pink module gene(GSE133624) | BLCA-related DEGs |
|---|---|---|
| HLF | PI16 | COMTD1 |
| PLP1 | CFD | EVA1C |
| SCARA5 | SCARA5 | PRIMA1 |
| PI16 | GLP2R | PRKCB |
| C1QTNF7 | FAM107A | HSPG2 |
| PGM5P4 | LRRN4CL | CH25H |
| ADAMTS9-AS1 | GFRA1 | AGTR1 |
| MYOC | ESM1 | HOXB6 |
| TCF21 | TCF21 | HOXB5 |
| ADAMTSL3 | TNXB | CFD |
| GLP2R | PGR | FAXDC2 |
| LINC01082 | ADCY5 | FAM149A |
| ATP1A2 | C1QTNF7 | PLEK2 |
| PYGM | ITIH5 | NGFR |
| ADH1B | AFF3 | STARD13 |
| TNXB | MYOC | NR2F1 |
| BHMT2 | FENDRR | ABCA8 |
| MYOCD | HAND2 | GRIN2D |
| ECRG4 | INMT | MEX3A |
| ASPA | FOXF1 | CILP |
| RERGL | NALCN-AS1 | GJB6 |
| FXYD1 | ITGA8 | FXYD1 |
| VIT | PRAC1 | DCHS1 |
| TMEM252 | GPBAR1 | GSTM5 |
| RBFOX3 | TARID | STON1 |
| NEGR1 | ELANE | TCF21 |
| ITGA8 | GSN | PTH1R |
| KLHL41 | PLPP3 | HIF3A |
| PGM5-AS1 | PAMR1 | C1QTNF2 |
| OGN | LRRC3B | C1QTNF7 |
| SLC2A4 | ADAMTSL3 | ADAMTSL3 |
| SCN7A | SVEP1 | BMERB1 |
| MYOM1 | KLC3 | GPRASP1 |
| MICU3 | NTRK3 | MYOC |
| FGF10 | LGI4 | MAMDC2 |
| ADGRD1 | ADAMTS8 | PLCB4 |
| SGCG | LINC01081 | COL21A1 |
| MIR1-1HG-AS1 | ADAMTS9-AS2 | PAFAH1B3 |
| FAIM2 | SCN4B | MGLL |
| HAND2-AS1 | FBXL21P | SLC29A2 |
| TPPP | ADRA1A | TPSD1 |
| CHRM2 | HMGCLL1 | NFIX |
| ABCA8 | LTBP4 | ADCY5 |
| AFF3 | RCAN2 | FENDRR |
| ITIH5 | SPARCL1 | GSN |
| FENDRR | HAAO | SAMD4A |
| PDZRN4 | WFDC1 | NFIA |
| FILIP1 | CNTN2 | PLCD4 |
| SVEP1 | LRRC4B | CASC9 |
| PGM5 | ODAD2 | CLU |
| PLCD4 | PODN | SLC39A4 |

| | | |
|---|---|---|
| CPED1 | PRRG3 | SLC9A9 |
| CAVIN2 | SRPX | ROR1 |
| RBM24 | STON1 | IL6ST |
| AOX1 | LINC02884 | PFKFB4 |
| ANGPTL7 | FXYD1 | PARM1 |
| NPY6R | NR2F1 | SMYD2 |
| ASB5 | HPGDS | NDN |
| GFRA1 | PCOLCE2 | LRRC4B |
| VEGFD | ENPP6 | ZBTB16 |
| XPNPEP2 | BARX1 | IGSF10 |
| GNG7 | CRB2 | RCAN2 |
| PID1 | CYP4F24P | VPS9D1-AS1 |
| GPRASP1 | SLIT3 | CPQ |
| SHISAL1 | EFCC1 | SYNE1 |
| RANBP3L | MAS1L | ADAMTS1 |
| PRIMA1 | TNS1 | MDK |
| MBNL1-AS1 | BDKRB2 | ADAMTS8 |
| FAXDC2 | TACR3 | SNORD14E |
| CFD | OR7E47P | GPX3 |
| SYNPO2 | PDGFRA | INMT |
| HSPB6 | MAMDC2 | PARD3B |
| PPP1R12B | LDB3 | HAND2 |
| MORN5 | GAS6 | ITGA8 |
| KCNMA1 | LINC01798 | DSG2 |
| ADAM33 | ZCCHC24 | ASPA |
| ABI3BP | RGS13 | ITIH5 |
| ADRB3 | PLPPR4 | SLC24A3 |
| ZBTB16 | BMP5 | PCOLCE2 |
| HMCN2 | MMP23B | CCDC69 |
| PDE2A | SCUBE1 | NTN1 |
| CGNL1 | IGSF10 | THSD4 |
| RCAN2 | GFRA2 | TBX1 |
| SYNE1 | FBLN2 | PPP1R14B |
| SOBP | ACACB | PCDH7 |
| SYNM | TRPA1 | PPP1R14B-AS1 |
| LRFN5 | TNNT1 | TSPAN18 |
| C5orf66-AS1 | KCNS2 | PI16 |
| ACACB | PLCD4 | OCIAD2 |
| P2RY14 | CYGB | CDO1 |
| GREM2 | NFIA | HGH1 |
| MEN1 | PRCD | ANTXR2 |
| TMOD1 | CSRNP1 | RPS6KA2 |
| PCOLCE2 | MCM4 | OXER1 |
| P2RX1 | TMEM119 | PTGDS |
| PTGFR | GPRASP1 | SYNPO |
| PAMR1 | LAMC3 | SOX4 |
| IGSF10 | ZBTB16 | TNS1 |
| CBX7 | CBX7 | MIF |
| MOGS | PAPPA | PLPP3 |
| NR3C2 | KLF2 | EPHA3 |
| PRDM6 | GPR17 | SLC22A3 |
| ANK2 | ORC6 | TSHZ3 |

| | | |
|---|---|---|
| C7 | ADAMTS1 | TMTC1 |
| RNF150 | LAMA2 | IGSF9 |
| CRY2 | KLF9 | GAPDHP1 |
| ADCY5 | NR4A1 | APOD |
| GNAL | TMEM88 | NMB |
| FOXF1 | CYS1 | TMEM74B |
| ASB2 | MUSK | FOXF1 |
| SLIT3 | A2M | PDE2A |
| SORBS2 | SORBS2 | PYCR3 |
| ADAMTS1 | TNS2 | WFDC1 |
| EPM2A | PNMA8B | CX3CL1 |
| SLC52A2 | VIPR2 | ESM1 |
| DIXDC1 | CEP85 | FCER1A |
| PDK4 | GPR146 | TNFAIP8L3 |
| KCNMB1 | HEPACAM | PSD |
| NCAM1 | PER1 | TSC22D3 |
| DOK6 | RPS6KA2 | GFRA1 |
| CADM3 | TEDC2 | NME1 |
| GALNT17 | ROR1 | CDCP1 |
| DTNA | LRRC2 | MICU3 |
| ADAMTS8 | PDE2A | CSTB |
| KCND3 | EDNRB | TNXB |
| PER2 | CALML3-AS1 | HAAO |
| FHL1 | LINC00092 | PRICKLE2 |
| AGTR1 | NDNF | PHYHIP |
| DMD | CD302 | KIAA0513 |
| TMEM220 | SNED1 | PERP |
| SRF | OXER1 | APBB1 |
| NFASC | SELENOI | IL33 |
| NME1 | PHYHIP | VEGFD |
| GATA5 | VEGFD | SORBS2 |
| RAB9B | FOLR2 | PER1 |
| LIMS2 | TRIP13 | NUDT8 |
| PSD | TSC22D3 | STEAP3 |
| PDE1A | CDC25C | PRDM6 |
| HGH1 | CCDC69 | ARHGAP39 |
| POLR2H | CYBRD1 | RTL5 |
| SMTN | AQP1 | NR3C2 |
| ALG3 | NDC1 | CCND2 |
| TCEAL2 | ASB2 | PCAT6 |
| CHRDL1 | TAMALIN | PALM |
| MAMDC2 | TONSL | SVEP1 |
| HPSE2 | LIN9 | PAMR1 |
| CSRP1 | LINC01719 | SCARA3 |
| LDB3 | PRICKLE2 | SCARA5 |
| TMEM100 | FGL2 | NPR1 |
| RAI2 | P2RY12 | LTBP4 |
| FBXL22 | HIF3A | RASGRP2 |
| PSMG3 | TNFAIP8L3 | SEZ6L2 |
| PTH1R | METTL24 | ACACB |
| ACOX2 | IL6ST | EFNA4 |
| SGCA | FAM110D | CACNA1H |

CASQ2
MYH11
TACR2
TCEAL7
FAM189A2
WFDC1
LMOD1
TBX20
LGI4
GATA6
FLNC
TNS1
CFL2
AARD
PHYHIP
HAND2
PRUNE2
CACNA1H
MYLK
SORBS1
SLIT2
REEP1
FBXL6
CSGALNACT1
HAAO
CACNB2
EPHA3
LTBP4
PLCB4
NECAB1
SLC66A1
FGF2
PPP1R14B
PDE4D
CCDC69
PLPP3
MMRN1
DACT3
GNAO1
PDE5A
CLEC3A
EPHA7
LEPR
SLC25A39
PRKG1
SLMAP
LINC00641
STUM
ACTA2-AS1
AOC3
CNN1
LMO3
MTBP
DNMT3B
ANLN
FRMD6-AS2
CIDEC
NFIX
RNF112
ATAD2
BIN1
NPR1
NXPH4
RASA3-IT1
TM4SF19
DARS2
RPH3AL-AS1
MEF2C-AS1
LINC01778
AURKA
PREX2
MEX3A
TNFSF12
MCM3AP-AS1
CD34
CALML3
PSRC1
SMYD2
PLK1
EME1
KCNAB1
TESMIN
SLC44A5
MASP1
GBP6
EXO1
CLDN5
GSTM5
CCT5
INTS8
PSD
DIO3OS
CDO1
NR4A3
DUSP1
C21orf58
KIF20A
GYPE
AVPR2
ABCA8
DMRTA2
TPSG1
PPFIA4
MGLL
RAI2
ATP5MF
NIBAN1
TFAP2A
CBX7
ISG15
GNAL
ULBP2
LAMC3
HSPB8
LDB3
DBI
AFF3
TMEM220
ALDH2
CA2
GLP2R
ARHGEF19
ETV4
GREM2
C5orf66-AS1
ACOX2
MMRN1
SCN4B
RBMS3
ATP1A2
GNG7
NT5DC2
SLIT3
CRYM
LGI4
ACTA2-AS1
ASB2
SNORD60

| | |
|---|---|
| PRICKLE2 | CDCA4 |
| ITPKB | MAP3K21 |
| HIF3A | GJB6 |
| COASY | CCDC150 |
| GSN | SLC38A1 |
| NXPH3 | ENPP3 |
| PAFAH1B3 | C1QTNF2 |
| ITPR1 | TTK |
| TARBP2 | UBE2T |
| ACTC1 | CEP72 |
| POP7 | NLN |
| LAMC3 | ADRA2A |
| SH3BGR | TPSB2 |
| DUS1L | NEK2 |
| MEF2D | C6orf223 |
| RBMS3 | CENPF |
| BCHE | WDHD1 |
| PER1 | LGI1 |
| NBEA | SKP2 |
| ITGB1BP2 | RASGRP2 |
| YDJC | ACTA2-AS1 |
| PTGS1 | ALKAL2 |
| NACC2 | C5orf66-AS1 |
| HSPB2 | OAF |
| DSTN | LCN6 |
| LPP | RECQL4 |
| CCDC137 | E2F7 |
| PARD3B | KIF14 |
| OMD | GREM2 |
| MPZ | CIT |
| C1QTNF2 | FAM227A |
| AUP1 | ASPM |
| ZFPM2 | BUB1 |
| PELI2 | CENPO |
| LSM4 | ATP1B2 |
| ANGPTL1 | ATP1A2 |
| TBX5 | KIF2C |
| THSD4 | KNSTRN |
| DNAJB5 | NAV3 |
| POPDC2 | SOX17 |
| MAOB | RAD54L |
| MAP3K20 | B3GNT4 |
| PDZD4 | RNU1-149P |
| STON1 | CENPI |
| FLAD1 | MAST1 |
| LRRC45 | DTL |
| FNBP1 | GGTA1 |
| CMA1 | ZWILCH |
| OLFM1 | GINS1 |
| KANK2 | ARHGAP20 |
| TP53INP2 | SQLE |
| RHOB | RGS5 |

| | |
|---|---|
| PPP1R14BP3 | PTTG1 |
| DPP3 | SLC39A4 |
| ZEB1 | HMMR |
| ROR1 | GAS7 |
| RERG | EGR3 |
| SMUG1 | C12orf73 |
| GHR | SPRY1 |
| RBPMS2 | TOX2 |
| MAP1B | IQGAP3 |
| ACTG2 | ARHGEF39 |
| OXLD1 | CHRNA5 |
| SCN4B | PACC1 |
| HAND1 | CDC42EP2 |
| MIX23 | TSPAN11 |
| SEMA3E | RCOR2 |
| CH25H | ANGPTL5 |
| INMT | RFC5 |
| PLN | LINC01896 |
| PJA2 | PTGDS |
| ARHGAP39 | FCER1A |
| AGRN | ZNF117 |
| TNFAIP8L3 | AATBC |
| LYVE1 | CCNB1 |
| EFNA4 | FANCD2 |
| PPP1R14A | MCM10 |
| PRKCB | TIMELESS |
| TMUB1 | TBC1D31 |
| NFIA | TROAP |
| CTSG | RPL7L1P9 |
| MAP1A | SORCS1 |
| BLOC1S3 | NCAPD3 |
| RGN | FOSB |
| HSPB7 | UBE2C |
| SPEG | PLK4 |
| CPEB2 | CD1C |
| ATP5MF | MAGI2-AS3 |
| LINC00865 | BRIP1 |
| TPM1 | PRDM6 |
| PPP1CA | LINC01985 |
| MTURN | SRD5A1 |
| MEIS1 | MIR433 |
| LYPLA2 | ZBTB20 |
| SLC24A3 | MAPK13 |
| SLC9A9 | TRABD2B |
| AP1S1 | RNASEH2A |
| KCNH2 | VSTM4 |
| SNHG1 | PPAT |
| NCALD | ARID5A |
| ZNF710-AS1 | ACOX2 |
| TMEM223 | PKD1L2 |
| FERMT2 | TICRR |
| PCP4 | RBMS3 |

| | |
|---|---|
| STARD13 | CORO2B |
| GSTM5 | PDE1C |
| CACNA1C | BDKRB1 |
| SFRP1 | CDCA2 |
| NIBAN1 | KIF4A |
| LRCH2 | TOP2A |
| PFDN6 | ST6GALNAC3 |
| RASGRP2 | ACY1 |
| CNTN1 | KREMEN2 |
| TMEM35A | KIF15 |
| NAA40 | TCF24 |
| SAMD4A | MELK |
| CHRM3 | PPP1R1A |
| BAG2 | DNAH9 |
| SBSPON | GGH |
| ATP6V0B | TBX1 |
| TSC22D1 | MEF2C |
| TSPAN18 | DNA2 |
| MFSD3 | PTH1R |
| ITGA9 | HROB |
| ITGA7 | XRCC2 |
| IER5L | MTFR1 |
| PRKAG2 | VASN |
| DNAJB4 | CD1E |
| JPH2 | HMGB3 |
| PPP4C | SNORA72 |
| FYCO1 | ECT2 |
| MATN2 | DCAF13 |
| NAP1L5 | SLC9A9 |
| TSPYL2 | TSPAN4 |
| UBALD2 | KCNK2 |
| NR2C2AP | CSE1L |
| SLC39A4 | FANCB |
| ITPA | CDK1 |
| KCNK3 | EPHA3 |
| NAP1L2 | CRYM |
| CRYM | C1orf112 |
| COL21A1 | BRCA1 |
| ILK | GRK5 |
| MXRA7 | E2F3 |
| RAB23 | PFKFB4 |
| KLHL13 | EVA1C |
| C3orf70 | RGS9 |
| DES | EZH2 |
| KAT2A | RAB37 |
| PPP1R12A | DEPDC1 |
| FCER1A | ACTL6A |
| NFIX | FANCI |
| EDEM2 | NCAPG2 |
| RTL5 | CLEC10A |
| MIIP | GNAL |
| GFUS | PAICS |

| | |
|---|---|
| MGLL | FCER2 |
| RCAN1 | CHTF18 |
| ANTXR2 | HJURP |
| C2CD4B | LILRB5 |
| DENND2A | MN1 |
| OCIAD2 | RACGAP1 |
| AKAP12 | CD300LG |
| GSS | STIL |
| SLC37A4 | SERPINF1 |
| RPS6KA2 | ACKR1 |
| MRPS26 | GPD2 |
| TSEN54 | DSC2 |
| CNPY2 | SPAG5 |
| PPP1R35 | FLJ40194 |
| WWTR1 | NUP155 |
| ZDHHC12 | CDC6 |
| TENT5B | CHEK1 |
| BID | PRC1 |
| FOXN3 | PUS7 |
| PYCR3 | MIR770 |
| NPR2 | SEMA3G |
| MANF | SHE |
| BMERB1 | MT1JP |
| SSPN | LINC02461 |
| BRMS1 | ZNF93 |
| RNF122 | RHOJ |
| FZD7 | SCD |
| LRRC4B | SAPCD2 |
| SNHG12 | SYNE1 |
| HSPB8 | DNAH3 |
| SNORD99 | CKS1B |
| PNKP | ZNF724 |
| RSPO3 | C1R |
| CBX8 | PAFAH1B3 |
| TTLL7 | CDCA3 |
| SLC50A1 | ZNF572 |
| OXER1 | RAI2 |
| GPM6B | SYNDIG1L |
| RASL11A | CABP1 |
| PLA2G5 | EIF4EBP1 |
| HIPK3 | LDB2 |
| SMYD2 | RCVRN |
| TPSD1 | ARL6IP1 |
| ABL1 | CDC45 |
| SNRPF | CKAP2 |
| MGAT4B | CENPH |
| OSBPL10 | MIR25 |
| ENTPD6 | GINS2 |
| CPEB4 | TMEM220 |
| PSMB3 | LATS2 |
| DCHS1 | RGL1 |
| RHBDF2 | NT5DC2 |

| | |
|---|---|
| PRKAR2B | RNF139-AS1 |
| STK40 | CKS1BP1 |
| ARHGEF25 | NFATC2 |
| MRM1 | PBK |
| BCL2 | KPNA2 |
| MITF | ZNF695 |
| NPR1 | PRKAG2-AS1 |
| IL6ST | MCM7 |
| CILP | CCDC141 |
| SDF2L1 | CNTN4 |
| KIAA0513 | XRCC3 |
| TUB | FAXDC2 |
| PGP | ACSM5 |
| BVES | NR3C2 |
| GNAZ | RAD51AP1 |
| LIFR | CD164L2 |
| MYDGF | CKS2 |
| THRA | STARD13 |
| NT5DC3 | BORA |
| APBB1 | MMRN1 |
| RASSF3 | KIF11 |
| NCBP2AS2 | SYT15 |
| PABPC1L | TADA1 |
| LAMTOR2 | MAPK15 |
| SVIL | SIGLEC17P |
| MRPL12 | HSPH1 |
| CKLF | IGSF9B |
| DAAM2 | H2BU1 |
| MEIS2 | BEND3 |
| TLN1 | CDCA8 |
| CEP131 | CCN6 |
| FAM149A | SIGLEC11 |
| RGS19 | CACNA1H |
| DMPK | EPB41L2 |
| PFKFB4 | MIR22HG |
| C4orf48 | MYL3 |
| ABRACL | RAB3IL1 |
| PCDH18 | CEP55 |
| SLC25A23 | DNAH11 |
| PKDCC | CACYBP |
| UQCC3 | NR4A1AS |
| TSHZ3 | RBL1 |
| SLC25A22 | TGM1 |
| DUSP8 | ERCC6L |
| EML1 | PDCD2L |
| GFRA3 | FZD6 |
| VAMP8 | LRRK2 |
| PDXK | ABCB1 |
| PLEKHJ1 | ASPA |
| NR2F1 | GPC2 |
| TSPAN2 | KIF24 |
| SLC25A4 | DPYSL2 |

| | |
|---|---|
| CASP6 | ARHGAP11A |
| SNORD104 | CRHBP |
| LPAR1 | NIBAN1 |
| INPP5A | SCN1B |
| ARID5B | PTGER2 |
| TEAD1 | MTFR2 |
| CDO1 | BUB1B |
| PLSCR4 | KIF18A |
| TBX4 | THOC3 |
| HSPA2 | PARPBP |
| LSM7 | MOXD1 |
| ADARB1 | H2BC20P |
| C8orf88 | ADCY9 |
| NCS1 | TPX2 |
| CAP2 | STARD8 |
| PPP1R16A | LINC00877 |
| EVA1C | CENPA |
| PARVA | DLC1 |
| ADCK5 | TPSD1 |
| SH3D19 | HCN3 |
| GALE | COL25A1 |
| SOX4 | KCNA5 |
| PCK2 | CEROX1 |
| TCEAL1 | ZSCAN2 |
| KANK1 | OMG |
| EXOSC4 | LINC01355 |
| ESM1 | ATAD5 |
| VCL | RNU6-1176P |
| CKB | KIFC1 |
| PTGDS | RFC4 |
| TMEM160 | CFP |
| CACNA2D1 | HOOK1 |
| SYNPO | TFR2 |
| ROMO1 | CDCA5 |
| ALDH1B1 | SETBP1-DT |
| ETV4 | RN7SL749P |
| NFIC | LINC01410 |
| TENT5A | FAM72D |
| PAXX | CKAP2L |
| TNNT2 | ESPL1 |
| NFATC4 | SOX18 |
| COMTD1 | CDC20 |
| S100A11 | SPDL1 |
| RUSC2 | SGO1 |
| RPS6KA1 | RORB |
| C1orf21 | AXL |
| REEP4 | KIF23 |
| C1orf53 | GTF2IP5 |
| MFSD10 | TRAIP |
| PCGF2 | SH3RF3-AS1 |
| RAD9A | MICU3 |
| PARM1 | TUBA5P |

| | |
|---|---|
| SMIM10 | FBXO45 |
| ALDH2 | INSYN1 |
| CCND2 | ACVRL1 |
| TSPAN7 | ZC3H8 |
| UTRN | C19orf48 |
| ISG15 | RAD51 |
| CES1 | ZDHHC23 |
| ARHGEF26 | NME1 |
| SNHG3 | CCNB2 |
| PER3 | ADCYAP1R1 |
| TMTC1 | LINC01695 |
| MIF | IQCC |
| TOMM34 | KNL1 |
| SNHG25 | EBP |
| PCAT6 | DUXAP8 |
| FBXO6 | TMEM52 |
| HOXB7 | NSUN2 |
| PPP1R14B-AS1 | LINC00924 |
| ARHGEF37 | CHAF1B |
| TGFBR2 | ORC1 |
| PPP1R3B | CENPL |
| TSC22D3 | LRP8 |
| IL33 | MASTL |
| ATP2B4 | GDPD2 |
| CLSTN3 | LINC00958 |
| ARMCX1 | CDT1 |
| ITGA1 | CDK15 |
| ULBP2 | ULBP2 |
| MIR7111 | SKA1 |
| HILPDA | ECE2 |
| CPQ | NTN1 |
| NTN1 | S1PR1 |
| PBXIP1 | ESRP1 |
| NFIB | KCP |
| HOMER3 | CENPU |
| CD200 | PSMC3IP |
| PSME2 | CILP |
| PTGS2 | SDC3 |
| PNRC1 | STMN1 |
| NMB | IL22RA1 |
| TNFRSF25 | CAPN6 |
| COL4A6 | KNTC1 |
| NUDT8 | APBB1 |
| AKT3 | SYNPO |
| HSBP1L1 | SNORD73B |
| MCAM | PABPC5 |
| PARP12 | DLGAP5 |
| MIR4477B | FAM180B |
| SLC25A10 | LINC01346 |
| SLC29A2 | DIPK2B |
| PITX2 | VPS9D1-AS1 |
| EFNA1 | POU6F1 |

| | |
|---|---|
| MEX3A | ZNF738 |
| CCDC3 | NCAPG |
| SGCB | CTSV |
| SLC22A3 | MCMDC2 |
| PCCA-DT | FRGCA |
| TBL1X | SNORD114-4 |
| PHYHD1 | GRIK3 |
| MAPRE2 | WDR62 |
| SH3BGRL | SKA3 |
| AMOTL1 | TRPC3 |
| MIEN1 | NLRP6 |
| TACC1 | PPP1R14B |
| GABARAPL1 | ARHGAP39 |
| AHNAK | BIRC5 |
| CLU | DDX12P |
| PBX1 | ZNF736 |
| PCDH7 | SASS6 |
| MGP | CENPW |
| IGSF9 | TMEM74B |
| REEP2 | SCIRT |
| HACD1 | CCM2L |
| CX3CL1 | AADACL4 |
| CDC42EP3 | INA |
| CDIP1 | PKP1 |
| DBI | SLC17A8 |
| MSX1 | VSIR |
| SELENOP | ESCO2 |
| TSTD1 | ENAM |
| JCAD | CREB3L4 |
| ZFP36L1 | TMEM132A |
| WNT9A | DUXAP10 |
| SNORD14E | CLEC9A |
| DPYSL3 | COL21A1 |
| DYNC1I1 | ZNF681 |
| MDK | ZNF74 |
| PALM | FOXM1 |
| STEAP3 | PLEKHO2 |
| CDCP1 | HDC |
| GPX3 | TMEM220-AS1 |
| NDN | DIO3 |
| SGCE | LY6K |
| CAPG | NPM1P9 |
| NT5DC2 | RPL23AP32 |
| PYGB | PAPOLA-DT |
| WLS | EGFL7 |
| GRIN2D | SLC24A3 |
| SNORA33 | POC1A |
| APOD | KRT18P59 |
| SPRY2 | ZBED9 |
| MPP7 | EMP3 |
| LYNX1 | ADAM10 |
| PLA2G4A | ANKEF1 |

| | |
|---|---|
| TMEM74B | CELF2 |
| COL7A1 | MMP2 |
| ARHGEF19 | S100A14 |
| NEURL1B | DUXAP9 |
| PNMA1 | CCNE2 |
| CBX6 | PRR11 |
| CSPG4 | F13A1 |
| DUX4L50 | MTERF3 |
| SNORD60 | FANCA |
| OAS3 | PAQR4 |
| FAM50B | LINC02598 |
| S1PR5 | SMC4 |
| FNDC10 | DDR2 |
| SELENBP1 | EFNA4 |
| CTSF | IL2 |
| GNA14 | HLX-AS1 |
| FBXO32 | DEPDC1B |
| DSG2 | THBS1 |
| KIT | FANCL |
| HSPG2 | SETBP1 |
| CDH3 | SIX4 |
| STEAP4 | KIF18B |
| MDFI | PLXNA3 |
| NRN1 | TACR1 |
| FSCN1 | STAM-AS1 |
| PLEK2 | PCNA |
| PYCARD | STEAP3 |
| OAS1 | NFATC1 |
| VPS9D1-AS1 | CKMT2 |
| SLC16A3 | GEM |
| PLA2G2A | SYNE4 |
| CD79B | POLQ |
| NGFR | PIMREG |
| CA9 | DUS4L |
| LRIG1 | TRIM59 |
| ETV7 | PTX3 |
| SCARA3 | CCNF |
| CYBA | FZD10-AS1 |
| TFAP2A | IL20RB |
| LAMC2 | ZNF260 |
| ACKR3 | CTSE |
| CSTB | ZC3HAV1L |
| FBLN1 | MYADM |
| HOXB5 | LINC02765 |
| GALNT14 | VEGFA |
| HES4 | FAM72B |
| CDKN1C | MEDAG |
| HOXB6 | TNMD |
| NNAT | STXBP6 |
| CASC9 | IGSF9 |
| SEZ6L2 | CHML |
| GJB2 | NR2F2-AS1 |

PERP
GJB6
CCL19
MMP1
TBX1
CASP14
AHNAK2
FAM83A
GAPDHP1
CA2
TCN1

CIP2A
INCENP
CBX2
COLEC12
CRLF2
NTF3
NEXN-AS1
NDC80
NOP56
MIR27A
TFRC
GPER1
TANC2
OIP5
GTF2IRD1
C5orf34-AS1
RANBP1
CYP46A1
HSPE1
PARM1
MYOM2
ZNF253
ABCB4
PKMYT1
SPC25
ABCC9
STK31
OPLAH
RPP40
HAP1
MNX1
ZNF391
TSHZ3
KIF22
NRXN2
COL16A1
AKNA
NUSAP1
SOCS3
PRIM1
ZWINT
CCDC184
CDH4
RPS12P5
ARHGEF19
MTCP1
LINC02861
IL17RB
ERV3-1
BEGAIN
POLE2
EHD2

CNGA3
FAM124A
ERI2
LINC02268
INTS2
RAC3
GPT2
EPHA1
ADRA1D
PLD4
CENPK
ZBTB41
CFAP251
CSF1
CREB3L1
MIS18A
DUSP5P1
ASF1B
HSPG2
CCNA2
SBK1
LHFPL5
LINC02891
ESPN
ZNF525
HSPB8
PLPPR5
FOXH1
HSF2BP
APOD
FEN1
CDK2AP2P2
WDR72
ZNF436-AS1
ITGA5
C2CD6
MAD2L1
RSKR
MLLT11
NUF2
RPL32P1
CCDC162P
PIEZO2
TNNI3
ITIH6
TMSB15B
JPT1
TSPAN18
DSCC1
PEAR1
SNRPB
ETV4

CKS1BP3
KNDC1
KIF26A
PDE9A-AS1
NKX3-2
ERG
SLC6A11
KIAA0513
SPC24
CAVIN4
C18orf54
H1-7
LRRC32
MAP3K14-AS1
TSPOAP1-AS1
NIPSNAP1
HSPD1
ZSCAN9
RNU6-1010P
TRIM61
UNC5C
FRRS1
ANKRD29
VSTM2A
ZNF322
HSP90AA3P
TBL1XR1
MCM8
SGO2
ZDHHC20
DLEU1
CCDC138
TK1
ZNF92
SYTL5
CFAP20DC
C1orf167
LINC02182
SOX4
ZNF497
DNM1P46
TARS1
CDH17
TMEM130
NDN
GINS4
COA6
LINC00994
FASN
FIGNL1
CYP21A2
FABP5

LINC00964
RASA3
DACT1
TMEM255A
MAL2
RAD54B
MIR23A
ZNF107
LINC00968
DCST1-AS1
JAZF1
ATG9B
GTSE1
H2AW
PDE7B
LINC01359
ANTXR2
CHMP4C
MAGOHB
DNAJC9
AUNIP
DCHS1
BLM
ACP6
CX3CR1
GPAT3
CEP76
MIR3153
SCARF1
UBE2S
ZEB2
ZNF322P1
ANKRD44-IT1
MMS22L
RANBP1P1
KANK3
ROBO4
LINC01119
THSD4
PPIAP29
GEN1
AURKB
ZNF678
PDSS1
DNASE1L2
EPHA5
MEST
WFDC21P
GALNT15
ADCY4
CRHR2
RLN2

PLPPR1
SNORA38B
TSHZ3-AS1
MZT2A
FAT4
ACR
B4GALNT4
MOCOS
TERT
PRIMA1
TP53AIP1
SLX4IP
SHANK3
CAVIN3
FAM72A
NCAPH
HOXC9
GRID2
SHISA2
MND1
NRIP2
LINC02135
ENTPD1
LINC01409
C1QTNF4
WNT3
ATP5MF
OTULIN-DT
PRKN
KCNA2
SEPTIN3
CDC25A
SYT14
LINC01224
DQX1
FAM171A2
MKI67
MIR548AA2
MRPL13
CENPE
VRK1
HOMER2
S1PR2
H2BC19P
PTPRR
NFE2L1-DT
DBNDD1
ELAPOR2
MCM2
KCNIP3
FAM43A
CELSR3

ZNF423
TRIM24
PCOLCE
PPIAP9
CCDC34
SIM2
GPX3
PPM1K
DIAPH3
ZBTB46
STRIP2
TMPOP2
ERVMER34-1
ZNF607
LINC00491
C20orf96
HOXB6
SH3RF3
CACYBPP2
SERPINA5
S100B
GTF2IP14
GPR15
RTN4R
ASNSP6
CCNP
SLC29A2
GRIK1-AS1
SLC5A9
SNORD72
KCTD12
ALDH2
PNPLA7
DOK5
RNF212
PYCR3
USHBP1
C3
NUPR1
KLK6
FBXO41
CELF5
IL21-AS1
KRT24
AQP7P2
HID1-AS1
SMG7-AS1
LINC01819
B3GALNT1
LRP1
OCIAD2
PCLAF

ANGPTL2
KLHL2P1
MZT1
CLEC11A
PERP
KCNJ12
PDE10A
SDHAF3
MAPK8IP2
SLC45A2
GALNT1
MIR181A2HG
SLC22A3
DNM1P47
STOM
NKAIN1
KY
SMYD3
SFRP5
CDKN3
C1S
CCND2
CPA4
GAPDHP1
ACTG1P25
ZNF726
MRPL36
UNC5C-AS1
DDIAS
IGFBP6
SAMD4A
C5orf34
SNORD19C
PCAT6
DHH
DDN-AS1
FERMT1
RMI2
ZSCAN12P1
DSP
CSF3
CENPX
MIR4664
BMPER
FGD2
SHCBP1
EMSLR
ZNF385D
NUDT1
CX3CL1
HYLS1
RAG1

GGCT
RAET1K
GNGT1
TMEM191C
CBFA2T3
PALM
CCDC178
LINC01489
ASCL5
ZNF761
CMTM5
PRR5-ARHGAP8
ZNF165
RN7SKP184
NETO2
VWF
FAM83B
RPSAP69
SNORD14E
ALOXE3
PPP1R14B-AS1
RHPN1-AS1
CASKIN1
FBN2
RTL5
TCAM1P
MYRIP
RADX
ZNF610
ANKRD44
GPR158
ELOCP2
CLSPN
GRHL2
ABCA9
NMUR1
CNGB3
ADAMTS7P4
ENKUR
KCNIP1
FABP5P7
GRIN2D
EPHX4
TVP23A
CHRNA1
SIX1
AGMAT
LINC00847
SYPL1P2
ZNF841
AHCTF1P1
PNMA2

KISS1R
ZNF252P-AS1
TP63
SLCO1A2
FABP5P1
MNX1-AS1
TPO
RBMS3-AS3
DBI
SLC24A4
PODXL2
MIR4653
DSCAML1
RPL4P7
LINC01424
PCDHB9
LHCGR
HELLS
ARHGEF15
NMB
TFIP11-DT
HOXB8
NEIL3
GAD1
SPACA6P-AS
RNU6-10P
ZNF730
IFFO1
AMACR
PTRH2
NEB
SNORA60
NBPF6
SORCS2
AMIGO2
BRCA2
CRH
DPF1
LSR
TAF7L
LINC00671
LINC02894
ANO4
MRGPRF-AS1
CDCA7
MMP19
CADM4
SMIM24
PYCR1
CERS3
AADAT
NDUFA4L2

IL1RL2
PXYLP1
PANK1
IGSF21
PWWP3B
RFC3
E2F1
TBX15
ADH1A
HHIP
GAL
LSAMP
H2AC11
DGUOK-AS1
XYLB
OLFM2
HOXB5
FOXI2
C4B
SENCR
SOAT2
OR52N4
GRID1
PGAP1
ENPP1
ARL5C
MSL3P1
RIC3
CLEC4GP1
MIR4258
MYOZ3
CFAP53
SUMO2P1
FABP6
PCDHB8
ULBP1
FAM111B
ATP2A1-AS1
GRHL1
SH2D3C
OTOF
KIF4B
MIF
CPQ
MEG9
FOXP4-AS1
FIRRE
ZNF367
NBEAP3
GBGT1
GLI1
DNASE1L3

FAM241B
NMU
DHCR7
WNT5B
TOB2P1
ZNF254
ZNF239
NUP62CL
HEY1
DNM1P51
SNX29P2
PPIAP54
EBF2
TGFB3
SRRM3
RMI1
MSC
CXCR1
DGKG
ZNF528-AS1
ADAMTS4
SYNE3
DLX6
RGS20
BEND4
HMGCS1
RERG-IT1
PSAT1
ZNF887P
LINC01197
MYLK2
VENTX
TEK
TMTC1
CYP27A1
CHDH
ABCC8
FGF13-AS1
NUDCP2
LINC02257
VDAC2P2
HTR2C
GIMAP6
LINC01678
ZP3
RTKN2
MIR4652
PDIA2
RNU6-762P
VIM
TPD52
CCNE1

HSPE1P2
PABPC3
RN7SL32P
C4A
GRB7
RAB11FIP4
IL6
TRPM2-AS
CCND2-AS1
GPR78
NOVA2
STING1
FAM180A
UGT1A10
SEMA6B
THEM6
TMEM255B
ATP8B2
FAM234B
TXN
RN7SKP116
YWHAZP3
HSPE1P18
PPFIA3
ROR2
LINC01572
SLC25A5P5
FBXO43
LINC01311
ACBD7
SYT7
MTFP1
SFN
SPATA17
IRAK3
KIFC2
FAM110B
BHLHE22
RDH16
LINC00355
ODAD1
BMP8B
PRR7
CYP4A22-AS1
PKP3
CD160
PCP2
IL33
EPHA4
LINC00634
RANBP3-DT
GRHL3-AS1

RN7SL494P
ZNF812P
HTRA1
ASPHD1
TMPO-AS1
ITGB6
USP18
SLCO6A1
CXCR2
CABYR
CLEC18C
MANEA-DT
PARD3B
PLEKHA4
SULT1C4
CENPM
MAGEA11
KDF1
HGF
LAD1
SEZ6L2
CD200R1
HSPE1P4
TAS2R5
HIC2
VCX
KCNMB2-AS1
FAM153B
FZD5
GEMIN8P4
TIE1
MRC2
SPEF1
SLC10A4
LINC01842
PCMTD1P3
PROS1
KIF12
LINC02691
CRYZL2P-SEC16B
CDCP1
SEPTIN6
SMPD4P1
SUMO2P3
MYO10
NUDT8
COL27A1
PEAK3
ZNF823
IL34
PIF1
ADGRB3

LINC01376
PPP1R14C
IKZF2
SCARA3
SPINK5
TYMS
SEPTIN14P12
VCX2
MYH13
LILRA2
STK32A
KDM4A-AS1
SLC24A2
ACVR2B
CELSR2
RIBC2
S100A16
LINC02752
EHF
MIR23B
LHB
TIMP3
LINC00665
DNAH14
TMEFF2
OTX1
PHACTR1
FBXW11P1
MIR924HG
TTC23L
PRSS16
RN7SKP78
TACC3
ACAP2-IT1
ADAMTS10
RRM2
C6orf52
PTPN5
PNPLA1
ZNF192P1
CDH5
CYP21A1P
PTPRG-AS1
CYP27B1
BMERB1
THOC1-DT
MIR2052HG
UFL1-AS1
HOXC6
FXYD2
ADAMTS9
AGGF1P1

CD2AP-DT
NNMT
AKR7A3
SERPING1
ZSCAN23
TLE6
LINC02185
IMPDH1P8
MTSS1
LINC01914
PRPH
HMGN1P4
MROH6
TFAP2A
PCDHGB7
GJA4
HOXC8
SNORD114-2
LINC01807
DDN
NRIP3
NINL
HOXC-AS2
WDR87BP
FAM83C
PCDHGC3
VN1R42P
TBXA2R
HPDL
HASPIN
SLC12A8
CPNE4
DPP4
TFDP1P2
COX20P1
ADAMTSL4
NBPF4
C9orf163
CBS
GP5
CD200R1L-AS1
PLA2G12AP1
RNFT2
ENO2
PTCHD3P2
PLEK2
LINC02207
SERPINE2
VPS37D
EMX1
FLJ31356
LINC01198

SALL4
DRAXIN
KIF19
APLN
SH2B3
TRGV4
ZNF366
SPAG17
FYN
ALOX12
BCL6B
CHAC2
FSIP2-AS1
LIF
CKS1BP2
CARMIL3
PHOSPHO1
CXCL2
SCG5
H2BC5
NLRP3
IL36G
RN7SKP40
COMTD1
LRRC34
CH25H
FSD1
CST6
HAR1A
SNRPFP1
SNORA14B
STPG1
MRPL37P1
CBARP-DT
RN7SKP95
FABP5P10
CSTL1
LINC01366
HSD17B1
GNG7
ACTN3
E2F8
ARHGAP31
PSMD12P1
CSTB
GAPDHP63
GJB7
CRNDE
SLITRK6
THPO
USH2A
PTGES3P3

TMEM176B
NFE2L3
RASGRF2
GGT5
CD24P4
PLAC1
DIRAS2
H2BC11
ADH7
MAP1LC3C
MANEAL
PHF21B
CDC7
MID1IP1-AS1
HOXB4
SORD
H3P47
LGI2
H3C4
ZNF114
LYPD1
HEG1
ELMO1
DGCR5
H2BC13
COX6CP17
COPZ2
RASL11B
FAM131C
DNM3OS
GPR20
FSIP2
ZEB2-AS1
SAMD11
TAS2R6P
MIR3155A
RN7SL138P
XXYLT1-AS2
LINC02344
HGH1
SNAP23P1
RFTN1
BATF3
FOXD2-AS1
ZNF45-AS1
HOXB3
DNAJC22
LINC00173
AGTR1
PPP1R26P1
RNU6-603P
VN1R108P

BCKDHA
IL36RN
FAM20C
TMEM71
LRRTM1
GPR137C
GLS2
RIN3
MAFG-DT
IQANK1
LINC02542
PLCB4
ZHX1-C8orf76
NECTIN4-AS1
C12orf56
LINC02888
LINC01550
LMNB1
NKPD1
GSDMA
ADCYAP1
SNORD127
LINC00896
SPAG4
MTND4P32
BTBD19
CPXM1
EEF1E1P1
DSG2
CCT4P2
LINC00337
ALDOC
C3orf86
EPHX3
SHISA6
PADI4
LINC01833
FABP5P2
DOCK11
FAM24B
AGGF1P2
SNORD88A
NGFR
FGF13
SRPK2P
ISL2
TEX46
GNG2
SETP12
KLHL35
PKIB
H4C11

SNORA71C
TCF7L1
GAPDHP40
ACKR4
CCDC26
ZNF285
THORLNC
SMPDL3B
PPATP1
SVOPL
TUBB3
LINC01814
TDRD5
CFL1P5
ITIH3
BFSP2-AS1
CFAP74
DPF3
SNAI3
RN7SL521P
RN7SKP173
GIPC3
RPL15P5
LINC02820
RPL17P50
DISP3
HAS3
FAM149A
ZNF826P
ANKRD50
LAGE3P1
LINC00960
TBCAP1
KIAA0895
YWHAZP5
MFAP2
IFI27
FAM96AP2
PLEKHG4B
LYPLA1P3
PRR7-AS1
KIRREL1-IT1
MROH7
PTGER4P2
OFCC1
ZNF878
CASC8
MIR559
PTH2R
SNORA15B-2
RIPOR2
SNORA26

CAGE1
ADGRF4
LIPG
LRRC77P
C10orf88B
SCEL
GSTM4
TYMSOS
CFAP47
C16orf95-DT
MIR205HG
VPS35P1
MIR126
RASGEF1A
STAC
RASAL1
RPS20P20
NALCN
UBE2L4
SUMO2P6
NECTIN4
BNIP3P1
EXOSC10-AS1
LINC00240
LPO
PCDH7
RNU4-78P
CLVS1
ANO1
ZNF350-AS1
LINC02595
ITPRIP-AS1
PRSS27
LINC02466
TOX
H2BC18
SNTG2
RTP5
MIX23P3
IGF2BP3
DSG1-AS1
DCLK1
MIR548AN
RNU6-658P
PRKCB
COLGALT2
HSPA7
LINC00853
LINC01748
DEFB126
LINC00319
SLC47A2

NACAD
GRIN3B
LINC02585
GGT2
YWHAZP2
CGREF1
RHCE
MCIDAS
GRB14
PEBP4
MATN3
RHOT1P3
CNIH3-AS2
EPCAM
SCN8A
SNORD12
CROCC2
CD93
KIF28P
SSBL2P
DPYS
LINC00525
ZSCAN5A-AS1
EPB41L4B
ZNF793-AS1
MT2A
HS6ST2
OR7E108P
RPE65
ELAVL2
HHIPL1
EPHB6
DYSF
GALNT9
C12orf75
SLC2A3
SLCO2A1
H3P36
NRP2
FTLP14
SDC1
LINC01535
FOXD4
DCDC1
KLRG2
CHRDL2
MEIS3P2
TATDN1P1
ALOX12P2
SSC4D
DPY19L2P1
ELFN1-AS1

PSLNR
G2E3-AS1
IL37
BOLA2P3
HSPA9P1
ZNF556
FAM183A
DPY19L3-DT
CLU
RPL17P13
MDK
TRAJ4
RPSAP3
NR1I2
ZNF454
RNU7-48P
PCDHA3
FAM135A-AS1
MKRN2OS
LRRC37A6P
ACER1
KLHL33
ESYT3
DHCR24
LINC00689
NAP1L3
TMEM52B
CBLN4
TAFA5
FAM83C-AS1
TMEM200C
BNIP3
BRWD1-AS1
EEF1A2
BEX2
LRRC4C
C5AR2
TMEFF1
IQSEC3
EVPL
ZNF415P1
PCAT7
COSMOC
RNU6-856P
RNU6-925P
GPR68
VIM-AS1
CYP26A1
DLGAP2
RIMKLA
CCDC78
LINC01980

SNORD53
UBE2Q2P2
RN7SL40P
CRP
ZNF559-ZNF177
TFPI2-DT
AMHR2
RNU6-501P
FAM242C
APOBR
DDX11L10
COQ7-DT
LINC01633
MYB
LINC01675
POTEKP
POF1B
ALOX15
PLCXD2
KCNG1
TMEM176A
E2F3-IT1
MIR4271
ALMS1P1
H2BC15
SPAG16-DT
PHF24
MAG
HOXC5
GBP1P1
LINC02577
SNORD53B
TMPRSS3
RPL21P65
LANCL3
TRGV2
RN7SL45P
PDZD7
SIGLEC8
ZNF486
LINC01979
ISG15
ZNF711
HSPA5P1
LINC00311
CABP4
FGFR3
PLOD2
MSI1
ACSL6
C7orf57
DHCR24-DT

RPL7P47
AVPR1A
SYCE3
METTL21AP1
GPR143
SLC5A12
HRC
DRC1
NRK
RNF128
WSCD1
MIR1207
PKIA
FHAD1
HS6ST3
CYSRT1
HDAC11-AS1
H1-2
TBR1
LINC00570
LYPD3
CA2
ZNF233
RN7SL333P
TIGD3
CFAP157
SLC2A9-AS1
TTC6
MIR210HG
OR7E110P
TSACC
RPL21P54
UGT2B11
CRYBB3
PAX9
VASH2
LINC00664
GABRR1
FOXL2NB
MIR5581
UBE2CP2
OVOL1-AS1
CALB1
FAR2
GPR1
CKMT1B
CXXC4
FOXD2
FUT3
ADAMTS15
GRIN1
FRAS1

PRB3
MT1P1
MTDHP1
LRRIQ4
EPDR1
GAPDHP62
MSC-AS1
APOL4
RPS2P4
ATOH7
PGK1P1
IMPA1P1
ZNF732
RIPPLY3
LINC02800
CKS1BP7
LINC02560
BTF3P6
DLX6-AS1
TEX38
GLTPD2
MCF2L-AS1
CEBPA-DT
H2AC13
STAB2
CSRP3-AS1
GPR37L1
C16orf54
P2RX7
PLAG1
RAB44
ATP6V1B1
TMEM97P1
CASC9
RN7SL12P
SHANK1
NDUFB4P12
FAM153A
PCBP3
LINC01186
SERPINA3
NRG2
DLG1-AS1
LAPTM4BP2
MIR378D2HG
RPSAP13
DNAJB13
LINC00992
NKX6-1
IDI2-AS1
NOS3
MARCO

CSF3R
ADGRV1
RCCD1-AS1
IGFBP5
HSP90AA4P
PCDH10
XKR9
GPR142
H3C14
NPIPA3
DMKN
CRACD
TXNP4
HAUS6P3
RPL31P15
OVOL3
MTND1P32
KCNK17
P3H2-AS1
RN7SL535P
LINC01876
ROCK1P1
ZIC2
GALR2
ZPLD2P
IGFL2-AS1
PLA2G4C
MYBL2
RGPD4
RPL7L1P8
OR2B6
TNFAIP6
VAX2
GOLGA8UP
ABCA13
RPSAP45
SMKR1
MIR544B
GLT1D1
RPH3A
TRGV8
MRPL40P1
RBM22P2
LGALS8-AS1
RPS6P12
GPM6A
ANKRD19P
DHX35-DT
DSCR9
RPS17
WNT9B
CSTA

RPS27P21
GPRACR
H2AC18
MXRA5Y
LINC01068
MIR554
FEZF1-AS1
CYP4F11
H3P1
ZNF154
AMH
RNU6-377P
RN7SL213P
SLC16A10
MTHFD2P7
SLC15A1
RPL7AP31
SUMO2P21
SNORD60
MESP1
E2F2
RNU6-1237P
DNAAF3
RNU6-431P
COLEC10
EMBP1
RAB3B
TRIM17
SLFN13
WNT10B
RN7SL124P
CYP2J2
PDE1B
LINC02004
G3BP1P1
ADAMTSL1
NR2E1
HS3ST4
CENPCP1
SMIM17
SIGLEC22P
CAMK2N2
CHMP5P1
COL6A4P1
MYOZ2
HSPA8P8
BTF3L4P3
C11orf72
RGMA
BMS1P16
SNORA74D
DLG3-AS1

CATSPER4
KCNJ3
ONECUT2
RN7SL757P
KCNE1
IQCM
RPL9P16
KRT8P36
RPL39P15
SNRPCP2
RNA5SP296
DIP2C-AS1
GJB4
FAM222A-AS1
SHISAL2B
RNY3P15
EZH2P1
SYNPR-AS1
PSME2P6
PPIAP3
PAX6
HNRNPLP1
NFIA-AS2
TPTEP1
LINC02026
SMC1B
HRH2
FOXL2
LINC02516
ST3GAL6-AS1
ATP13A4
MIR944
KCTD19
MIR1302-3
H3P4
ACRV1
E2F3P2
RNU1-72P
TRIM34
ANO5
DIPK1C
LNC-LBCS
KRT15
RNA5SP464
DRC7
MIPEPP3
FAT2
RNA5SP465
LINC01285
LINC02757
DYRK3-AS1
LINC00677

KRT18P54
SNORD71
RNU6-298P
SREK1IP1P2
SNORD67
IFIT1P1
COX8C
MIR4520-1
RSPH10B2
LINC01998
KCNG2
NXT1-AS1
SOX21-AS1
RNU6-1285P
BRWD1P2
TMEM139-AS1
UBL5P2
YWHAQP6
SUN3
PDE6A
LINC01556
POTEF
H2BS1
PHC2-AS1
LINC01347
APOBEC2
NDUFA5P12
SLC25A24P1
RELN
GALR3
NHLH2
LAPTM4A-DT
RN7SL16P
PTPN20
LINC01214
CCT5P1
ATP5F1CP1
MTND2P2
FOXJ1
SNTG1
FOXA3
DNAJA1P3
RNU6-48P
GNAT2
GOSR2-DT
H3C12
SMILR
NCAM2
FFAR3
SKA2P1
MIR3136
LINC01767

LINC00543
PPIAP40
PCK1
FAR2P1
SERTM2
CCL21
H1-1
CYP19A1
BSN-DT
H1-5
CTB-178M22.2
TAS2R3
MIR606
GUCY1B2
PSMC1P5
SLC25A5P2
FUT6
WFDC5
AKR1C2
RPS29P5
ENTHD1
RSL24D1P1
MIR4639
RPS3AP37
