# Supplementary material for: Identification of Potential Biomarkers for Progression and Prognosis of Bladder Cancer by Comprehensive Bioinformatics Analysis
Source: J Oncol. 2022 Apr 19;2022:1802706. doi: 10.1155/2022/1802706 (PMC9042640; doi:10.1155/2022/1802706)
Supplement: Supplementary Materials — Supplementary Figure 1: WGCNA analysis of the TCGA dataset. Supplementary Figure 2: WGCNA analysis of the GSE133624 dataset. Supplementary Figures 3–7: clinical relevance of SMYD2, GAPDHP1, CILP, ATP1A2, and THSD4. Supplementary Table 1: primer sequences in the study. Supplementary Table 2: DEGs in the TCGA dataset. Supplementary Table 3: DEGs in the GSE133624 dataset. Supplementary Table 4: DEGs coexisting in the TCGA and GSE133624 datasets. Supplementary Table 5: feature genes were selected with the SVM-RFE algorithm. Supplementary Table 6: the correlation between the characteristic genes and immune cells. Supplementary Table 7: single-gene GSEA for prognostic genes. [file 1802706.f1.zip › 1802706.f1/Supplementary Table 5.pdf]

| LASSO | SVM-RFP | Feature genes |
|---|---|---|
| C1QTNF7 | ESM1 | C1QTNF7 |
| ATP1A2 | THSD4 | ATP1A2 |
| ASPA | FXYD1 | FXYD1 |
| FXYD1 | CFD | CFD |
| VEGFD | SMYD2 | LGI4 |
| GNG7 | ACTA2-AS1 | ACTA2-AS1 |
| PRIMA1 | LGI4 | PER1 |
| CFD | CILP | THSD4 |
| LDB3 | PER1 | SMYD2 |
| LGI4 | NMB | CILP |
| ACTA2-AS1 | STON1 | ESM1 |
| PER1 | ADAMTS1 | ULBP2 |
| THSD4 | C1QTNF7 | NMB |
| SMYD2 | HAND2 | GAPDHP1 |
| CILP | ATP1A2 | |
| PFKFB4 | ULBP2 | |
| SOX4 | CPQ | |
| ESM1 | GAPDHP1 | |
| ULBP2 | SLC29A2 | |
| NMB | SNORD14E | |
| NUDT8 | | |
| HSPG2 | | |
| GAPDHP1 | | |
| CA2 | | |
