# Supplementary material for: Identification of Potential Biomarkers for Progression and Prognosis of Bladder Cancer by Comprehensive Bioinformatics Analysis
Source: J Oncol. 2022 Apr 19;2022:1802706. doi: 10.1155/2022/1802706 (PMC9042640; doi:10.1155/2022/1802706)
Supplement: Supplementary Materials — Supplementary Figure 1: WGCNA analysis of the TCGA dataset. Supplementary Figure 2: WGCNA analysis of the GSE133624 dataset. Supplementary Figures 3–7: clinical relevance of SMYD2, GAPDHP1, CILP, ATP1A2, and THSD4. Supplementary Table 1: primer sequences in the study. Supplementary Table 2: DEGs in the TCGA dataset. Supplementary Table 3: DEGs in the GSE133624 dataset. Supplementary Table 4: DEGs coexisting in the TCGA and GSE133624 datasets. Supplementary Table 5: feature genes were selected with the SVM-RFE algorithm. Supplementary Table 6: the correlation between the characteristic genes and immune cells. Supplementary Table 7: single-gene GSEA for prognostic genes. [file 1802706.f1.zip › 1802706.f1/Supplementary Table 6.pdf]

**Immune cell**	**Gene**	**cor**	**p.value**
Activated B cell	SMYD2	0.056086	0.261308
Activated CD4 T cell	SMYD2	0.090718	0.068875
Activated CD8 T cell	SMYD2	0.034924	0.484473
Activated dendritic cell	SMYD2	0.052669	0.291534
CD56bright natural killer cell	SMYD2	-0.09625	0.053531
CD56dim natural killer cell	SMYD2	-0.10838	0.0296
Central memory CD4 T cell	SMYD2	-0.03259	0.51422
Central memory CD8 T cell	SMYD2	-0.02129	0.669997
Effector memory CD4 T cell	SMYD2	0.064852	0.193872
Effector memory CD8 T cell	SMYD2	0.018474	0.711571
Eosinophil	SMYD2	-0.09479	0.057264
Gamma delta T cell	SMYD2	0.046359	0.35327
Immature B cell	SMYD2	0.018763	0.707275
Immature dendritic cell	SMYD2	0.032987	0.509041
Macrophage	SMYD2	-0.02953	0.554441
Mast cell	SMYD2	-0.06055	0.225154
MDSC	SMYD2	0.00568	0.90949
Memory B cell	SMYD2	-0.09128	0.067177
Monocyte	SMYD2	-0.16106	0.001177
Natural killer cell	SMYD2	0.014077	0.77815
Natural killer T cell	SMYD2	0.001683	0.973133
Neutrophil	SMYD2	-0.01822	0.715439
Plasmacytoid dendritic cell	SMYD2	-0.04045	0.418056
Regulatory T cell	SMYD2	0.038986	0.435095
T follicular helper cell	SMYD2	0.00603	0.903951
Type 1 T helper cell	SMYD2	0.039725	0.426434
Type 17 T helper cell	SMYD2	-0.01175	0.81415
Type 2 T helper cell	SMYD2	-0.02388	0.632686
Activated B cell	GAPDHP1	-0.17807	0.000328
Activated CD4 T cell	GAPDHP1	-0.02842	0.569445
Activated CD8 T cell	GAPDHP1	-0.08122	0.103511
Activated dendritic cell	GAPDHP1	-0.06354	0.203046
CD56bright natural killer cell	GAPDHP1	0.117623	0.01817
CD56dim natural killer cell	GAPDHP1	0.008441	0.865858
Central memory CD4 T cell	GAPDHP1	-0.03671	0.462403
Central memory CD8 T cell	GAPDHP1	-0.1501	0.002519
Effector memory CD4 T cell	GAPDHP1	-0.15996	0.001274
Effector memory CD8 T cell	GAPDHP1	-0.07772	0.119275
Eosinophil	GAPDHP1	-0.11026	0.026879
Gamma delta T cell	GAPDHP1	-0.10999	0.027258
Immature B cell	GAPDHP1	-0.14757	0.002983
Immature dendritic cell	GAPDHP1	-0.10494	0.035207
Macrophage	GAPDHP1	-0.11193	0.024634
Mast cell	GAPDHP1	-0.19601	7.46E-05
MDSC	GAPDHP1	-0.10516	0.034822
Memory B cell	GAPDHP1	-0.09871	0.047682
Monocyte	GAPDHP1	-0.09209	0.064763
Natural killer cell	GAPDHP1	-0.14193	0.004305
Natural killer T cell	GAPDHP1	-0.08712	0.080657
Neutrophil	GAPDHP1	-0.00393	0.937364
Plasmacytoid dendritic cell	GAPDHP1	-0.10395	0.036979

Regulatory T cell	GAPDHP1	-0.08463	0.089746
T follicular helper cell	GAPDHP1	-0.11494	0.021004
Type 1 T helper cell	GAPDHP1	-0.18039	0.000273
Type 17 T helper cell	GAPDHP1	-0.15263	0.002123
Type 2 T helper cell	GAPDHP1	-0.01585	0.751107
Activated B cell	ATP1A2	0.352722	2.99E-13
Activated CD4 T cell	ATP1A2	-0.07074	0.156352
Activated CD8 T cell	ATP1A2	0.084125	0.091693
Activated dendritic cell	ATP1A2	0.042481	0.395023
CD56bright natural killer cell	ATP1A2	-0.10484	0.035386
CD56dim natural killer cell	ATP1A2	0.145903	0.003329
Central memory CD4 T cell	ATP1A2	0.182796	0.000225
Central memory CD8 T cell	ATP1A2	0.258019	1.50E-07
Effector memory CD4 T cell	ATP1A2	0.429804	1.51E-19
Effector memory CD8 T cell	ATP1A2	0.112485	0.023929
Eosinophil	ATP1A2	0.293707	1.85E-09
Gamma delta T cell	ATP1A2	0.067577	0.175757
Immature B cell	ATP1A2	0.240138	1.08E-06
Immature dendritic cell	ATP1A2	0.155596	0.00173
Macrophage	ATP1A2	0.27415	2.22E-08
Mast cell	ATP1A2	0.472808	7.76E-24
MDSC	ATP1A2	0.18415	0.000201
Memory B cell	ATP1A2	0.324767	2.37E-11
Monocyte	ATP1A2	0.380653	2.42E-15
Natural killer cell	ATP1A2	0.295838	1.39E-09
Natural killer T cell	ATP1A2	0.089357	0.073157
Neutrophil	ATP1A2	-0.04496	0.368023
Plasmacytoid dendritic cell	ATP1A2	0.297839	1.06E-09
Regulatory T cell	ATP1A2	0.11815	0.017654
T follicular helper cell	ATP1A2	0.268132	4.59E-08
Type 1 T helper cell	ATP1A2	0.266681	5.46E-08
Type 17 T helper cell	ATP1A2	-0.01326	0.790707
Type 2 T helper cell	ATP1A2	0.040155	0.421438
Activated B cell	CILP	0.546684	8.78E-33
Activated CD4 T cell	CILP	0.238546	1.27E-06
Activated CD8 T cell	CILP	0.337287	3.53E-12
Activated dendritic cell	CILP	0.376735	4.89E-15
CD56bright natural killer cell	CILP	-0.07592	0.128107
CD56dim natural killer cell	CILP	0.189899	0.000125
Central memory CD4 T cell	CILP	0.46849	2.23E-23
Central memory CD8 T cell	CILP	0.580188	1.25E-37
Effector memory CD4 T cell	CILP	0.579874	1.40E-37
Effector memory CD8 T cell	CILP	0.443056	8.33E-21
Eosinophil	CILP	0.334208	5.68E-12
Gamma delta T cell	CILP	0.391902	3.03E-16
Immature B cell	CILP	0.485772	2.97E-25
Immature dendritic cell	CILP	0.358708	1.11E-13
Macrophage	CILP	0.569815	4.55E-36
Mast cell	CILP	0.611016	1.31E-42
MDSC	CILP	0.529151	1.86E-30
Memory B cell	CILP	0.422668	6.80E-19
Monocyte	CILP	0.270571	3.43E-08

Natural killer cell	CILP	0.578404	2.35E-37
Natural killer T cell	CILP	0.480132	1.25E-24
Neutrophil	CILP	0.20002	5.26E-05
Plasmacytoid dendritic cell	CILP	0.537289	1.61E-31
Regulatory T cell	CILP	0.505148	1.74E-27
T follicular helper cell	CILP	0.55879	1.80E-34
Type 1 T helper cell	CILP	0.568702	6.63E-36
Type 17 T helper cell	CILP	0.036818	0.461084
Type 2 T helper cell	CILP	0.243748	7.31E-07
Activated B cell	THSD4	0.152966	0.002074
Activated CD4 T cell	THSD4	0.182796	0.000225
Activated CD8 T cell	THSD4	0.136621	0.006014
Activated dendritic cell	THSD4	0.272068	2.86E-08
CD56bright natural killer cell	THSD4	0.214346	1.42E-05
CD56dim natural killer cell	THSD4	-0.13764	0.005644
Central memory CD4 T cell	THSD4	0.39038	4.03E-16
Central memory CD8 T cell	THSD4	0.251139	3.26E-07
Effector memory CD4 T cell	THSD4	0.158887	0.001374
Effector memory CD8 T cell	THSD4	0.250867	3.36E-07
Eosinophil	THSD4	0.184896	0.00019
Gamma delta T cell	THSD4	0.25917	1.31E-07
Immature B cell	THSD4	0.228751	3.49E-06
Immature dendritic cell	THSD4	0.239968	1.09E-06
Macrophage	THSD4	0.259058	1.33E-07
Mast cell	THSD4	0.239869	1.11E-06
MDSC	THSD4	0.227024	4.15E-06
Memory B cell	THSD4	0.289266	3.30E-09
Monocyte	THSD4	-0.00872	0.861528
Natural killer cell	THSD4	0.382537	1.72E-15
Natural killer T cell	THSD4	0.339681	2.43E-12
Neutrophil	THSD4	0.315014	9.82E-11
Plasmacytoid dendritic cell	THSD4	0.298056	1.03E-09
Regulatory T cell	THSD4	0.335327	4.78E-12
T follicular helper cell	THSD4	0.314181	1.11E-10
Type 1 T helper cell	THSD4	0.204248	3.61E-05
Type 17 T helper cell	THSD4	0.121944	0.014303
Type 2 T helper cell	THSD4	0.340494	2.14E-12
