# Supplementary material for: Identification of Potential Biomarkers for Progression and Prognosis of Bladder Cancer by Comprehensive Bioinformatics Analysis
Source: J Oncol. 2022 Apr 19;2022:1802706. doi: 10.1155/2022/1802706 (PMC9042640; doi:10.1155/2022/1802706)
Supplement: Supplementary Materials — Supplementary Figure 1: WGCNA analysis of the TCGA dataset. Supplementary Figure 2: WGCNA analysis of the GSE133624 dataset. Supplementary Figures 3–7: clinical relevance of SMYD2, GAPDHP1, CILP, ATP1A2, and THSD4. Supplementary Table 1: primer sequences in the study. Supplementary Table 2: DEGs in the TCGA dataset. Supplementary Table 3: DEGs in the GSE133624 dataset. Supplementary Table 4: DEGs coexisting in the TCGA and GSE133624 datasets. Supplementary Table 5: feature genes were selected with the SVM-RFE algorithm. Supplementary Table 6: the correlation between the characteristic genes and immune cells. Supplementary Table 7: single-gene GSEA for prognostic genes. [file 1802706.f1.zip › 1802706.f1/Supplementary Table 7.docx]

**Supplementary Table 7 Single-gene GSEA for prognostic genes**

**TOP10 GO(BP) terms positively associated with SYMD2**

| NAME | SIZE | ES | NES | NOM p-val | FDR q-val |
| --- | --- | --- | --- | --- | --- |
| GOBP_DNA_DEPENDENT_DNA_REPLICATION | 157 | 0.6869145 | 2.3971193 | 0 | 0 |
| GOBP_REGULATION_OF_CHROMOSOME_SEPARATION | 71 | 0.7401152 | 2.3960028 | 0 | 0 |
| GOBP_CHROMOSOME_SEPARATION | 95 | 0.70341927 | 2.3779294 | 0 | 0 |
| GOBP_REGULATION_OF_CHROMOSOME_SEGREGATION | 89 | 0.6981637 | 2.3630087 | 0 | 0 |
| GOBP_METAPHASE_ANAPHASE_TRANSITION_OF_CELL_CYCLE | 64 | 0.73954666 | 2.350153 | 0 | 0 |
| GOBP_MITOTIC_SISTER_CHROMATID_SEGREGATION | 164 | 0.67063516 | 2.3410053 | 0 | 0 |
| GOBP_SISTER_CHROMATID_SEGREGATION | 199 | 0.65915227 | 2.323867 | 0 | 0 |
| GOBP_NEGATIVE_REGULATION_OF_CHROMOSOME_ORGANIZATION | 91 | 0.68475705 | 2.3235853 | 0 | 0 |
| GOBP_REGULATION_OF_MITOTIC_SISTER_CHROMATID_SEGREGATION | 45 | 0.7485886 | 2.3079128 | 0 | 0 |
| GOBP_RNA_EXPORT_FROM_NUCLEUS | 142 | 0.6658238 | 2.3046973 | 0 | 0 |

**TOP10 GO(BP) terms negatively associated with SYMD2**

| NAME | SIZE | ES | NES | NOM p-val | FDR q-val |
| --- | --- | --- | --- | --- | --- |
| GOBP_REGULATION_OF_CARDIAC_MUSCLE_CONTRACTION_BY_REGULATION_OF_THE_RELEASE_OF_SEQUESTERED_CALCIUM_ION | 21 | -0.7301497 | -2.5087314 | 0 | 6.69E-05 |
| GOBP_PURINERGIC_NUCLEOTIDE_RECEPTOR_SIGNALING_PATHWAY | 31 | -0.62511 | -2.4775774 | 0 | 1.94E-04 |
| GOBP_RESPONSE_TO_EPINEPHRINE | 15 | -0.7826165 | -2.3994226 | 0 | 4.56E-04 |
| GOBP_REGULATION_OF_CARDIAC_MUSCLE_CONTRACTION_BY_CALCIUM_ION_SIGNALING | 26 | -0.6619197 | -2.4093387 | 0 | 4.67E-04 |
| GOBP_REGULATION_OF_B_CELL_PROLIFERATION | 61 | -0.5348282 | -2.392561 | 0 | 5.02E-04 |
| GOBP_ARACHIDONIC_ACID_METABOLIC_PROCESS | 59 | -0.5177111 | -2.3595603 | 0 | 6.50E-04 |
| GOBP_POSITIVE_T_CELL_SELECTION | 36 | -0.56436765 | -2.2779233 | 0 | 0.001302346 |
| GOBP_REGULATION_OF_SMOOTH_MUSCLE_CONTRACTION | 57 | -0.5139168 | -2.2623854 | 0 | 0.001681242 |
| GOBP_CELLULAR_EXTRAVASATION | 69 | -0.48371178 | -2.2438946 | 0 | 0.002201475 |
| GOBP_MICROGLIAL_CELL_ACTIVATION | 46 | -0.52576286 | -2.2422507 | 0 | 0.002256648 |

**TOP10 KEGG pathways positively associated with SYMD2**

| NAME | SIZE | ES | NES | NOM p-val | FDR q-val |
| --- | --- | --- | --- | --- | --- |
| KEGG_SPLICEOSOME | 127 | 0.6848615 | 2.3566709 | 0 | 0 |
| KEGG_CELL_CYCLE | 125 | 0.6734158 | 2.3148675 | 0 | 0 |
| KEGG_DNA_REPLICATION | 36 | 0.7329832 | 2.172229 | 0 | 0 |
| KEGG_AMINOACYL_TRNA_BIOSYNTHESIS | 41 | 0.71407604 | 2.1414945 | 0 | 0 |
| KEGG_RNA_DEGRADATION | 59 | 0.6635347 | 2.135223 | 0 | 0 |
| KEGG_HOMOLOGOUS_RECOMBINATION | 28 | 0.7334271 | 2.058621 | 0 | 0 |
| KEGG_N_GLYCAN_BIOSYNTHESIS | 46 | 0.65692943 | 2.000312 | 0 | 1.77E-04 |
| KEGG_NUCLEOTIDE_EXCISION_REPAIR | 44 | 0.665116 | 2.009496 | 0 | 2.02E-04 |
| KEGG_MISMATCH_REPAIR | 23 | 0.7106127 | 1.9580894 | 0 | 3.88E-04 |
| KEGG_PROTEIN_EXPORT | 24 | 0.6955826 | 1.9139985 | 0 | 7.64E-04 |

**TOP10 KEGG pathways negatively associated with SYMD2**

| NAME | SIZE | ES | NES | NOM p-val | FDR q-val |
| --- | --- | --- | --- | --- | --- |
| KEGG_DRUG_METABOLISM_CYTOCHROME_P450 | 69 | -0.5255989 | -2.4174473 | 0 | 0 |
| KEGG_HEMATOPOIETIC_CELL_LINEAGE | 85 | -0.4696112 | -2.3045268 | 0 | 0 |
| KEGG_METABOLISM_OF_XENOBIOTICS_BY_CYTOCHROME_P450 | 67 | -0.5025386 | -2.2745352 | 0 | 0 |
| KEGG_COMPLEMENT_AND_COAGULATION_CASCADES | 69 | -0.47579688 | -2.1684 | 0 | 0.0017680 |
| KEGG_CYTOKINE_CYTOKINE_RECEPTOR_INTERACTION | 264 | -0.36878476 | -2.1483748 | 0 | 0.0020525 |
| KEGG_INTESTINAL_IMMUNE_NETWORK_FOR_IGA_PRODUCTION | 46 | -0.5015504 | -2.0572155 | 0 | 0.0024139 |
| KEGG_VASCULAR_SMOOTH_MUSCLE_CONTRACTION | 115 | -0.4020118 | -2.0823379 | 0 | 0.0028163 |
| KEGG_ARACHIDONIC_ACID_METABOLISM | 58 | -0.44960433 | -1.915489 | 0 | 0.0063562 |
| KEGG_PRIMARY_BILE_ACID_BIOSYNTHESIS | 16 | -0.6073254 | -1.9827852 | 0.004115226 | 0.0063668 |
| KEGG_JAK_STAT_SIGNALING_PATHWAY | 155 | -0.36325815 | -1.9047868 | 0 | 0.0065860 |

**TOP10 GO(BP) terms positively associated with GAPDHP1**

| NAME | SIZE | ES | NES | NOM p-val | FDR q-val |
| --- | --- | --- | --- | --- | --- |
| GOBP_COTRANSLATIONAL_PROTEIN_TARGETING_TO_MEMBRANE | 105 | 0.7273343 | 3.2639859 | 0 | 0 |
| GOBP_NUCLEAR_TRANSCRIBED_MRNA_CATABOLIC_PROCESS_NONSENSE_MEDIATED_DECAY | 120 | 0.67877746 | 3.1768892 | 0 | 0 |
| GOBP_ESTABLISHMENT_OF_PROTEIN_LOCALIZATION_TO_ENDOPLASMIC_RETICULUM | 119 | 0.6598618 | 3.0786881 | 0 | 0 |
| GOBP_ATP_SYNTHESIS_COUPLED_ELECTRON_TRANSPORT | 100 | 0.65633273 | 2.9146495 | 0 | 0 |
| GOBP_MITOCHONDRIAL_ELECTRON_TRANSPORT_NADH_TO_UBIQUINONE | 55 | 0.71212995 | 2.899876 | 0 | 0 |
| GOBP_OXIDATIVE_PHOSPHORYLATION | 148 | 0.6005411 | 2.8874934 | 0 | 0 |
| GOBP_NADH_DEHYDROGENASE_COMPLEX_ASSEMBLY | 65 | 0.68055713 | 2.8753214 | 0 | 0 |
| GOBP_RESPIRATORY_ELECTRON_TRANSPORT_CHAIN | 116 | 0.62011296 | 2.8600113 | 0 | 0 |
| GOBP_RIBOSOMAL_LARGE_SUBUNIT_ASSEMBLY | 28 | 0.7923857 | 2.7657728 | 0 | 0 |
| GOBP_VIRAL_GENE_EXPRESSION | 198 | 0.5450685 | 2.7512865 | 0 | 0 |

**TOP10 GO(BP) terms negatively associated with GAPDHP1**

| NAME | SIZE | ES | NES | NOM p-val | FDR q-val |
| --- | --- | --- | --- | --- | --- |
| GOBP_HUMORAL_IMMUNE_RESPONSE_MEDIATED_BY_CRCULATING_IMMUNOGLOBULIN | 145 | -0.65109617 | -2.84951 | 0 | 0 |
| GOBP_EXTERNAL_ENCAPSULATING_STRUCTURE_ORGANIZATION | 395 | -0.5799015 | -2.8445876 | 0 | 0 |
| GOBP_B_CELL_RECEPTOR_SIGNALING_PATHWAY | 120 | -0.6612018 | -2.83014 | 0 | 0 |
| GOBP_COMPLEMENT_ACTIVATION | 167 | -0.62857324 | -2.791081 | 0 | 0 |
| GOBP_REGULATION_OF_COMPLEMENT_ACTIVATION | 114 | -0.656909 | -2.769768 | 0 | 0 |
| GOBP_PHAGOCYTOSIS | 368 | -0.5663411 | -2.744901 | 0 | 0 |
| GOBP_REGULATION_OF_HUMORAL_IMMUNE_RESPONSE | 137 | -0.6297668 | -2.734765 | 0 | 0 |
| GOBP_B_CELL_MEDIATED_IMMUNITY | 215 | -0.59492093 | -2.7260127 | 0 | 0 |
| GOBP_REGULATION_OF_B_CELL_ACTIVATION | 182 | -0.6003947 | -2.6961575 | 0 | 0 |
| GOBP_ADAPTIVE_IMMUNE_RESPONSE_BASED_ON_SOMATIC_RECOMBINATION_OF_IMMUNE_RECEPTORS_BUILT_FROM_IMMUNOGLOBULIN_SUPERFAMILY_DOMAINS | 352 | -0.55762786 | -2.680746 | 0 | 0 |

**TOP10 KEGG pathways positively associated with GAPDHP1**

| NAME | SIZE | ES | NES | NOM p-val | FDR q-val |
| --- | --- | --- | --- | --- | --- |
| KEGG_RIBOSOME | 88 | 0.81820124 | 3.6774728 | 0 | 0 |
| KEGG_OXIDATIVE_PHOSPHORYLATION | 132 | 0.5782379 | 2.6773612 | 0 | 0 |
| KEGG_PARKINSONS_DISEASE | 130 | 0.5191677 | 2.4435852 | 0 | 0 |
| KEGG_HUNTINGTONS_DISEASE | 182 | 0.43263546 | 2.141868 | 0 | 5.84E-04 |
| KEGG_ALZHEIMERS_DISEASE | 166 | 0.40375885 | 1.9567242 | 0 | 0.003313565 |
| KEGG_PENTOSE_PHOSPHATE_PATHWAY | 27 | 0.44287968 | 1.4837104 | 0.025125628 | 0.1073528 |
| KEGG_RNA_POLYMERASE | 29 | 0.438074 | 1.5146813 | 0.0275 | 0.116396 |
| KEGG_SPLICEOSOME | 127 | 0.3181105 | 1.4848939 | 0.006451613 | 0.122208044 |
| KEGG_GLYCOLYSIS_GLUCONEOGENESIS | 61 | 0.34209734 | 1.4156017 | 0.030985916 | 0.13582298 |

**TOP10 KEGG pathways negatively associated with GAPDHP1**

| NAME | SIZE | ES | NES | NOM p-val | FDR q-val |
| --- | --- | --- | --- | --- | --- |
| KEGG_CELL_ADHESION_MOLECULES_CAMS | 131 | -0.6171659 | -2.6501849 | 0 | 0 |
| KEGG_FOCAL_ADHESION | 199 | -0.57529515 | -2.6255612 | 0 | 0 |
| KEGG_CHEMOKINE_SIGNALING_PATHWAY | 188 | -0.5758844 | -2.6225147 | 0 | 0 |
| KEGG_ECM_RECEPTOR_INTERACTION | 84 | -0.6309948 | -2.5645366 | 0 | 0 |
| KEGG_HEMATOPOIETIC_CELL_LINEAGE | 85 | -0.61104995 | -2.4833536 | 0 | 0 |
| KEGG_VIRAL_MYOCARDITIS | 68 | -0.6291743 | -2.4674459 | 0 | 0 |
| KEGG_CYTOKINE_CYTOKINE_RECEPTOR_INTERACTION | 264 | -0.51444227 | -2.4247308 | 0 | 0 |
| KEGG_LEISHMANIA_INFECTION | 70 | -0.6240387 | -2.4042954 | 0 | 0 |
| KEGG_T_CELL_RECEPTOR_SIGNALING_PATHWAY | 108 | -0.5657799 | -2.376625 | 0 | 0 |
| KEGG_LEUKOCYTE_TRANSENDOTHELIAL_MIGRATION | 116 | -0.55989146 | -2.370735 | 0 | 0 |

**TOP10 GO(BP) terms positively associated with ATP1A2**

| NAME | SIZE | ES | NES | NOM p-val | FDR q-val |
| --- | --- | --- | --- | --- | --- |
| GOBP_MUSCLE_CONTRACTION | 349 | 0.66335684 | 2.6744623 | 0 | 0 |
| GOBP_MUSCLE_SYSTEM_PROCESS | 442 | 0.6526347 | 2.654838 | 0 | 0 |
| GOBP_SMOOTH_MUSCLE_CONTRACTION | 98 | 0.72866744 | 2.566993 | 0 | 0 |
| GOBP_SMOOTH_MUSCLE_CELL_DIFFERENTIATION | 65 | 0.7565376 | 2.505642 | 0 | 0 |
| GOBP_REGULATION_OF_MUSCLE_SYSTEM_PROCESS | 235 | 0.63992107 | 2.501061 | 0 | 0 |
| GOBP_MUSCLE_ORGAN_DEVELOPMENT | 315 | 0.6250648 | 2.4959784 | 0 | 0 |
| GOBP_REGULATION_OF_BLOOD_CIRCULATION | 285 | 0.6277481 | 2.49331 | 0 | 0 |
| GOBP_MUSCLE_CELL_DIFFERENTIATION | 352 | 0.6136963 | 2.4720044 | 0 | 0 |
| GOBP_REGULATION_OF_HEART_CONTRACTION | 241 | 0.62792283 | 2.471795 | 0 | 0 |
| GOBP_ADENYLATE_CYCLASE_MODULATING_G_PROTEIN_COUPLED_RECEPTOR_SIGNALING_PATHWAY | 229 | 0.6349739 | 2.4509838 | 0 | 0 |

**TOP10 GO(BP) terms negatively associated with ATP1A2**

| NAME | SIZE | ES | NES | NOM p-val | FDR q-val |
| --- | --- | --- | --- | --- | --- |
| GOBP_MITOCHONDRIAL_TRANSLATION | 134 | -0.70690984 | -2.966476 | 0 | 0 |
| GOBP_MITOCHONDRIAL_GENE_EXPRESSION | 165 | -0.6823308 | -2.9083676 | 0 | 0 |
| GOBP_RIBOSOME_BIOGENESIS | 306 | -0.6218264 | -2.863776 | 0 | 0 |
| GOBP_MITOCHONDRIAL_TRANSLATIONAL_TERMINATION | 89 | -0.72765106 | -2.8397355 | 0 | 0 |
| GOBP_NCRNA_PROCESSING | 399 | -0.59216285 | -2.819241 | 0 | 0 |
| GOBP_NCRNA_METABOLIC_PROCESS | 491 | -0.5786241 | -2.8059266 | 0 | 0 |
| GOBP_TRNA_METABOLIC_PROCESS | 179 | -0.6417783 | -2.7746289 | 0 | 0 |
| GOBP_TRANSLATIONAL_TERMINATION | 105 | -0.6873529 | -2.75169 | 0 | 0 |
| GOBP_RRNA_METABOLIC_PROCESS | 237 | -0.610936 | -2.6965866 | 0 | 0 |
| GOBP_RIBONUCLEOPROTEIN_COMPLEX_BIOGENESIS | 468 | -0.5560288 | -2.6667538 | 0 | 0 |

**TOP10 KEGG pathways positively associated with ATP1A2**

| NAME | SIZE | ES | NES | NOM p-val | FDR q-val |
| --- | --- | --- | --- | --- | --- |
| KEGG_VASCULAR_SMOOTH_MUSCLE_CONTRACTION | 115 | 0.6871425 | 2.4916773 | 0 | 0 |
| KEGG_CALCIUM_SIGNALING_PATHWAY | 178 | 0.6551782 | 2.478916 | 0 | 0 |
| KEGG_DILATED_CARDIOMYOPATHY | 90 | 0.6947938 | 2.392802 | 0 | 0 |
| KEGG_HYPERTROPHIC_CARDIOMYOPATHY_HCM | 83 | 0.6926632 | 2.3590188 | 0 | 0 |
| KEGG_FOCAL_ADHESION | 199 | 0.58809614 | 2.236146 | 0 | 0 |
| KEGG_NEUROACTIVE_LIGAND_RECEPTOR_INTERACTION | 270 | 0.5616726 | 2.2110288 | 0 | 0 |
| KEGG_ARRHYTHMOGENIC_RIGHT_VENTRICULAR_CARDIOMYOPATHY_ARVC | 74 | 0.63178074 | 2.1336625 | 0 | 1.34E-04 |
| KEGG_ECM_RECEPTOR_INTERACTION | 84 | 0.57831305 | 1.9991424 | 0 | 3.65E-04 |
| KEGG_MAPK_SIGNALING_PATHWAY | 267 | 0.4978775 | 1.9732958 | 0 | 5.40E-04 |
| KEGG_COMPLEMENT_AND_COAGULATION_CASCADES | 69 | 0.56626505 | 1.8693268 | 0.001497006 | 0.0044643 |

**TOP10 KEGG pathways negatively associated with ATP1A2**

| NAME | SIZE | ES | NES | NOM p-val | FDR q-val |
| --- | --- | --- | --- | --- | --- |
| KEGG_PROTEASOME | 46 | -0.73400277 | -2.4993339 | 0 | 0 |
| KEGG_SPLICEOSOME | 127 | -0.5744571 | -2.4141784 | 0 | 0 |
| KEGG_GLYCOSYLPHOSPHATIDYLINOSITOL_GPI_ANCHOR_BIOSYNTHESIS | 25 | -0.7240833 | -2.162423 | 0 | 0 |
| KEGG_OXIDATIVE_PHOSPHORYLATION | 132 | -0.5033724 | -2.1154656 | 0 | 0 |
| KEGG_N_GLYCAN_BIOSYNTHESIS | 46 | -0.6029818 | -2.0940716 | 0 | 0 |
| KEGG_FRUCTOSE_AND_MANNOSE_METABOLISM | 34 | -0.6447426 | -2.0764956 | 0 | 0 |
| KEGG_AMINOACYL_TRNA_BIOSYNTHESIS | 41 | -0.63298917 | -2.075563 | 0 | 0 |
| KEGG_STEROID_BIOSYNTHESIS | 17 | -0.7559989 | -2.0436585 | 0 | 0 |
| KEGG_RIBOSOME | 88 | -0.52487296 | -2.0312085 | 0 | 0 |
| KEGG_AMINO_SUGAR_AND_NUCLEOTIDE_SUGAR_METABOLISM | 44 | -0.5756931 | -1.9843166 | 0 | 6.49E-04 |

**TOP10 GO(BP) terms positively associated with CILP**

| NAME | SIZE | ES | NES | NOM p-val | FDR q-val |
| --- | --- | --- | --- | --- | --- |
| GOBP_COMPLEMENT_ACTIVATION | 167 | 0.7746067 | 3.1335175 | 0 | 0 |
| GOBP_HUMORAL_IMMUNE_RESPONSE_MEDIATED_BY_CIRCULATING_IMMUNOGLOBULIN | 145 | 0.7752715 | 3.0659354 | 0 | 0 |
| GOBP_PHAGOCYTOSIS_RECOGNITION | 87 | 0.8193141 | 3.0614266 | 0 | 0 |
| GOBP_REGULATION_OF_COMPLEMENT_ACTIVATION | 114 | 0.77048063 | 2.9722133 | 0 | 0 |
| GOBP_B_CELL_RECEPTOR_SIGNALING_PATHWAY | 120 | 0.76083666 | 2.9585454 | 0 | 0 |
| GOBP_EXTERNAL_ENCAPSULATING_STRUCTURE_ORGANIZATION | 395 | 0.6722058 | 2.9440806 | 0 | 0 |
| GOBP_REGULATION_OF_HUMORAL_IMMUNE_RESPONSE | 137 | 0.74669456 | 2.9294586 | 0 | 0 |
| GOBP_MUSCLE_CONTRACTION | 349 | 0.6560256 | 2.8692572 | 0 | 0 |
| GOBP_MUSCLE_SYSTEM_PROCESS | 442 | 0.6282867 | 2.7747357 | 0 | 0 |
| GOBP_PHAGOCYTOSIS | 368 | 0.6237279 | 2.736269 | 0 | 0 |

**TOP10 GO(BP) terms negatively associated with CILP**

| NAME | SIZE | ES | NES | NOM p-val | FDR q-val |
| --- | --- | --- | --- | --- | --- |
| GOBP_MITOCHONDRIAL_TRANSLATION | 134 | -0.7504385 | -3.268101 | 0 | 0 |
| GOBP_MITOCHONDRIAL_GENE_EXPRESSION | 165 | -0.7338003 | -3.2613893 | 0 | 0 |
| GOBP_RIBOSOME_BIOGENESIS | 306 | -0.6720805 | -3.2278771 | 0 | 0 |
| GOBP_NCRNA_PROCESSING | 399 | -0.64971244 | -3.1955836 | 0 | 0 |
| GOBP_RIBONUCLEOPROTEIN_COMPLEX_BIOGENESIS | 468 | -0.6303252 | -3.1305604 | 0 | 0 |
| GOBP_NCRNA_METABOLIC_PROCESS | 491 | -0.62747484 | -3.106177 | 0 | 0 |
| GOBP_RRNA_METABOLIC_PROCESS | 237 | -0.65809464 | -3.0896232 | 0 | 0 |
| GOBP_MITOCHONDRIAL_TRANSLATIONAL_TERMINATION | 89 | -0.75765187 | -3.0725935 | 0 | 0 |
| GOBP_TRANSLATIONAL_TERMINATION | 105 | -0.73086447 | -3.0645232 | 0 | 0 |
| GOBP_DNA_DEPENDENT_DNA_REPLICATION | 157 | -0.6785791 | -3.0040622 | 0 | 0 |

**TOP10 KEGG pathways positively associated with CILP**

| NAME | SIZE | ES | NES | NOM p-val | FDR q-val |
| --- | --- | --- | --- | --- | --- |
| KEGG_HEMATOPOIETIC_CELL_LINEAGE | 85 | 0.7280056 | 2.6893537 | 0 | 0 |
| KEGG_CYTOKINE_CYTOKINE_RECEPTOR_INTERACTION | 264 | 0.63255686 | 2.669707 | 0 | 0 |
| KEGG_COMPLEMENT_AND_COAGULATION_CASCADES | 69 | 0.729632 | 2.6106884 | 0 | 0 |
| KEGG_HYPERTROPHIC_CARDIOMYOPATHY_HCM | 83 | 0.7083019 | 2.6009786 | 0 | 0 |
| KEGG_DILATED_CARDIOMYOPATHY | 90 | 0.6954823 | 2.5759547 | 0 | 0 |
| KEGG_FOCAL_ADHESION | 199 | 0.6254977 | 2.5597777 | 0 | 0 |
| KEGG_ECM_RECEPTOR_INTERACTION | 84 | 0.6892402 | 2.5564492 | 0 | 0 |
| KEGG_CELL_ADHESION_MOLECULES_CAMS | 131 | 0.6421135 | 2.5092413 | 0 | 0 |
| KEGG_INTESTINAL_IMMUNE_NETWORK_FOR_IGA_PRODUCTION | 46 | 0.74939543 | 2.4786594 | 0 | 0 |
| KEGG_VASCULAR_SMOOTH_MUSCLE_CONTRACTION | 115 | 0.6380463 | 2.4746451 | 0 | 0 |

**TOP10 KEGG pathways negatively associated with CILP**

| NAME | SIZE | ES | NES | NOM p-val | FDR q-val |
| --- | --- | --- | --- | --- | --- |
| KEGG_SPLICEOSOME | 127 | -0.6943121 | -2.9737551 | 0 | 0 |
| KEGG_OXIDATIVE_PHOSPHORYLATION | 132 | -0.56515867 | -2.4422128 | 0 | 0 |
| KEGG_RNA_DEGRADATION | 59 | -0.65014625 | -2.4044201 | 0 | 0 |
| KEGG_DNA_REPLICATION | 36 | -0.7070205 | -2.359502 | 0 | 0 |
| KEGG_CELL_CYCLE | 125 | -0.55085784 | -2.3383348 | 0 | 0 |
| KEGG_PROTEASOME | 46 | -0.6592447 | -2.3342698 | 0 | 0 |
| KEGG_BASE_EXCISION_REPAIR | 35 | -0.6829949 | -2.2403193 | 0 | 0 |
| KEGG_GLYCOSYLPHOSPHATIDYLINOSITOL_GPI_ANCHOR_BIOSYNTHESIS | 25 | -0.7341179 | -2.2201488 | 0 | 0 |
| KEGG_MISMATCH_REPAIR | 23 | -0.7397844 | -2.2083504 | 0 | 0 |
| KEGG_HOMOLOGOUS_RECOMBINATION | 28 | -0.6869579 | -2.2006428 | 0 | 0 |

**TOP10 GO(BP) terms positively associated with THSD4**

| NAME | SIZE | ES | NES | NOM p-val | FDR q-val |
| --- | --- | --- | --- | --- | --- |
| GOBP_EXTERNAL_ENCAPSULATING_STRUCTURE_ORGANIZATION | 395 | 0.6033739 | 3.1342905 | 0 | 0 |
| GOBP_CELL_SUBSTRATE_ADHESION | 355 | 0.59125596 | 3.0571973 | 0 | 0 |
| GOBP_SMOOTH_MUSCLE_CELL_PROLIFERATION | 150 | 0.65190303 | 3.0528018 | 0 | 0 |
| GOBP_MUSCLE_CELL_PROLIFERATION | 209 | 0.62215245 | 3.052363 | 0 | 0 |
| GOBP_CELL_SUBSTRATE_JUNCTION_ORGANIZATION | 106 | 0.6919395 | 3.0180058 | 0 | 0 |
| GOBP_CELL_MATRIX_ADHESION | 226 | 0.60513955 | 2.978413 | 0 | 0 |
| GOBP_MUSCLE_SYSTEM_PROCESS | 442 | 0.55806404 | 2.9467077 | 0 | 0 |
| GOBP_MUSCLE_CONTRACTION | 349 | 0.56450915 | 2.9169872 | 0 | 0 |
| GOBP_ACTOMYOSIN_STRUCTURE_ORGANIZATION | 188 | 0.60526776 | 2.8961937 | 0 | 0 |
| GOBP_POSITIVE_REGULATION_OF_CELL_ADHESION | 422 | 0.5527788 | 2.8914363 | 0 | 0 |

**TOP10 GO(BP) terms negatively associated with THSD4**

| NAME | SIZE | ES | NES | NOM p-val | FDR q-val |
| --- | --- | --- | --- | --- | --- |
| GOBP_MITOCHONDRIAL_TRANSLATION | 134 | -0.69033647 | -3.0422595 | 0 | 0 |
| GOBP_MITOCHONDRIAL_RESPIRATORY_CHAIN_COMPLEX_ASSEMBLY | 100 | -0.7088288 | -3.0357893 | 0 | 0 |
| GOBP_MITOCHONDRIAL_GENE_EXPRESSION | 165 | -0.66254973 | -2.9899905 | 0 | 0 |
| GOBP_OXIDATIVE_PHOSPHORYLATION | 148 | -0.6517669 | -2.8908813 | 0 | 0 |
| GOBP_MITOCHONDRIAL_TRANSLATIONAL_TERMINATION | 89 | -0.70444775 | -2.8867867 | 0 | 0 |
| GOBP_ATP_SYNTHESIS_COUPLED_ELECTRON_TRANSPORT | 100 | -0.6762037 | -2.8277016 | 0 | 0 |
| GOBP_TRANSLATIONAL_TERMINATION | 105 | -0.65320575 | -2.7394202 | 0 | 0 |
| GOBP_NADH_DEHYDROGENASE_COMPLEX_ASSEMBLY | 65 | -0.6956019 | -2.7320776 | 0 | 0 |
| GOBP_RESPIRATORY_ELECTRON_TRANSPORT_CHAIN | 116 | -0.6313353 | -2.6939745 | 0 | 0 |
| GOBP_TRNA_PROCESSING | 130 | -0.5993658 | -2.5942905 | 0 | 0 |

**TOP10 KEGG pathways positively associated with THSD4**

| NAME | SIZE | ES | NES | NOM p-val | FDR q-val |
| --- | --- | --- | --- | --- | --- |
| KEGG_FOCAL_ADHESION | 199 | 0.64669734 | 3.1577487 | 0 | 0 |
| KEGG_CYTOKINE_CYTOKINE_RECEPTOR_INTERACTION | 264 | 0.60409033 | 3.0134974 | 0 | 0 |
| KEGG_HEMATOPOIETIC_CELL_LINEAGE | 85 | 0.6904344 | 2.9369833 | 0 | 0 |
| KEGG_CELL_ADHESION_MOLECULES_CAMS | 131 | 0.6231619 | 2.8498888 | 0 | 0 |
| KEGG_ECM_RECEPTOR_INTERACTION | 84 | 0.67251706 | 2.8230336 | 0 | 0 |
| KEGG_HYPERTROPHIC_CARDIOMYOPATHY_HCM | 83 | 0.64490443 | 2.776618 | 0 | 0 |
| KEGG_ALLOGRAFT_REJECTION | 35 | 0.7679644 | 2.7481906 | 0 | 0 |
| KEGG_REGULATION_OF_ACTIN_CYTOSKELETON | 213 | 0.55455774 | 2.740288 | 0 | 0 |
| KEGG_GRAFT_VERSUS_HOST_DISEASE | 37 | 0.7662956 | 2.731345 | 0 | 0 |
| KEGG_JAK_STAT_SIGNALING_PATHWAY | 155 | 0.57617825 | 2.7306585 | 0 | 0 |

**TOP10 KEGG pathways negatively associated with THSD4**

| NAME | SIZE | ES | NES | NOM p-val | FDR q-val |
| --- | --- | --- | --- | --- | --- |
| KEGG_OXIDATIVE_PHOSPHORYLATION | 132 | -0.63268757 | -2.774487 | 0 | 0 |
| KEGG_SPLICEOSOME | 127 | -0.5798407 | -2.5441349 | 0 | 0 |
| KEGG_RIBOSOME | 88 | -0.6089506 | -2.5297556 | 0 | 0 |
| KEGG_PARKINSONS_DISEASE | 130 | -0.56530666 | -2.4973984 | 0 | 0 |
| KEGG_HUNTINGTONS_DISEASE | 182 | -0.50292087 | -2.2798932 | 0 | 0 |
| KEGG_BASE_EXCISION_REPAIR | 35 | -0.66108865 | -2.2659519 | 0 | 0 |
| KEGG_ALZHEIMERS_DISEASE | 166 | -0.47327936 | -2.124252 | 0 | 0 |
| KEGG_DNA_REPLICATION | 36 | -0.61465853 | -2.1194303 | 0 | 0 |
| KEGG_HOMOLOGOUS_RECOMBINATION | 28 | -0.65915847 | -2.103265 | 0 | 0 |
| KEGG_RNA_POLYMERASE | 29 | -0.619099 | -1.993832 | 0 | 6.69E-04 |
