# Supplementary figures and images for: Identification of Potential Biomarkers for Progression and Prognosis of Bladder Cancer by Comprehensive Bioinformatics Analysis
Source: J Oncol. 2022 Apr 19;2022:1802706. doi: 10.1155/2022/1802706 (PMC9042640; doi:10.1155/2022/1802706)

A

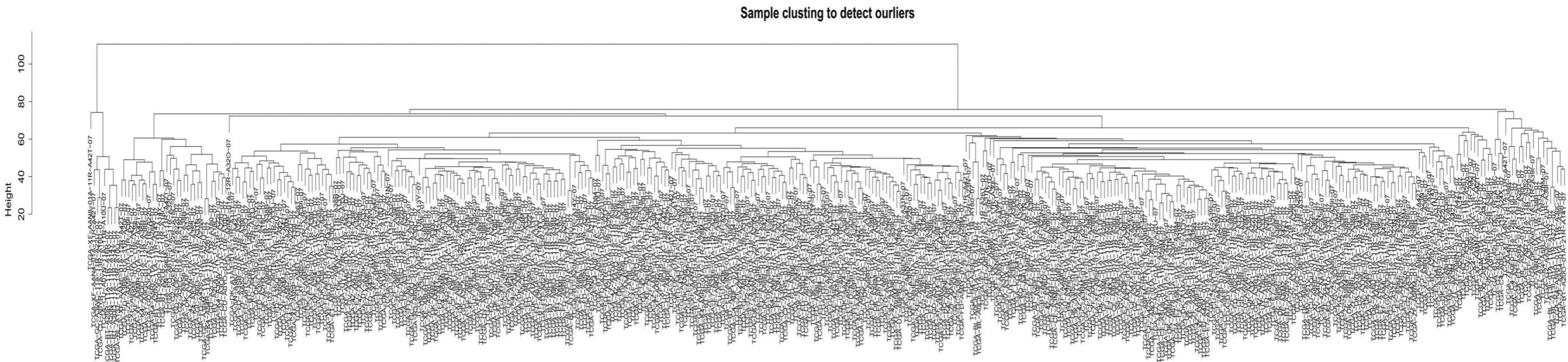

B

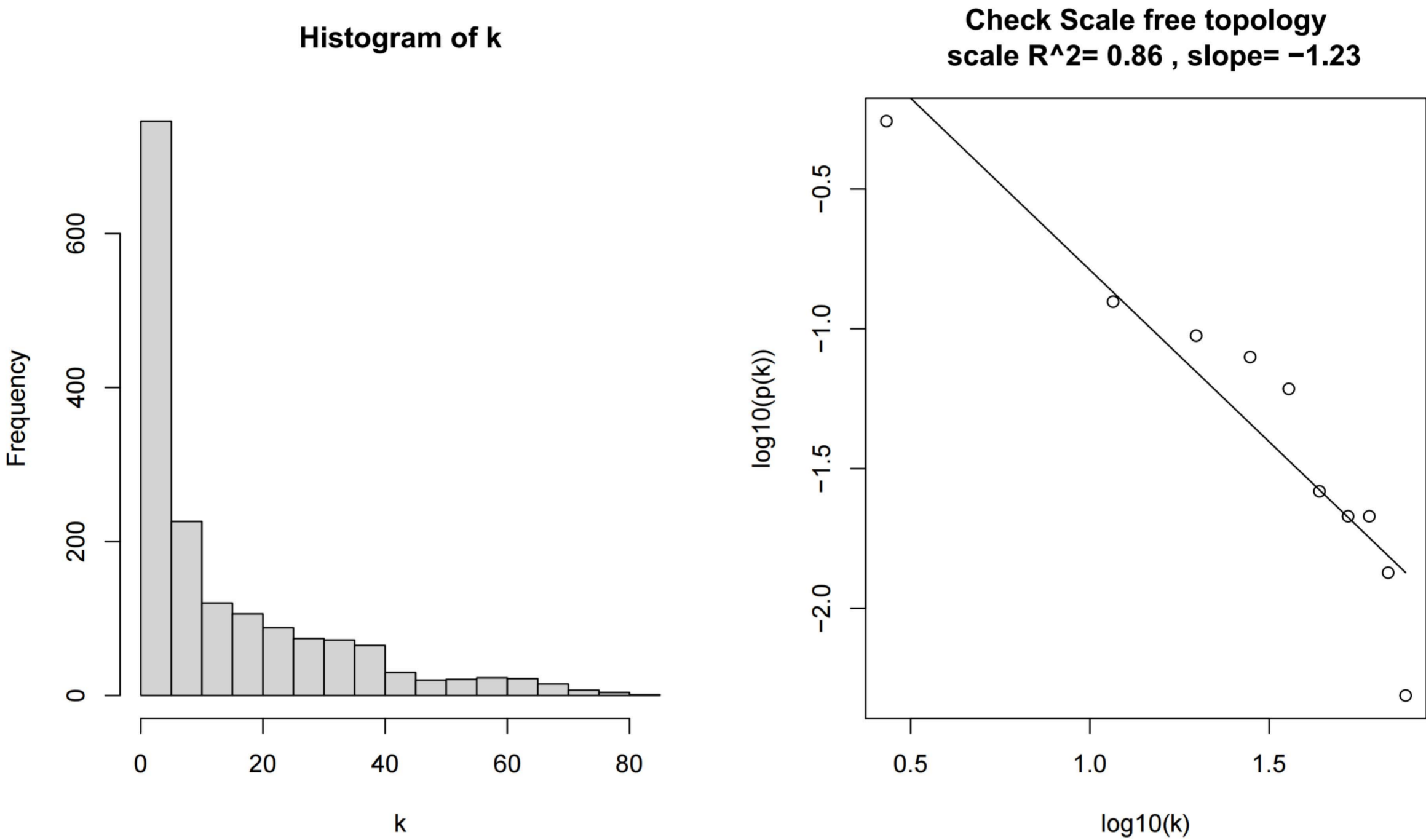

C

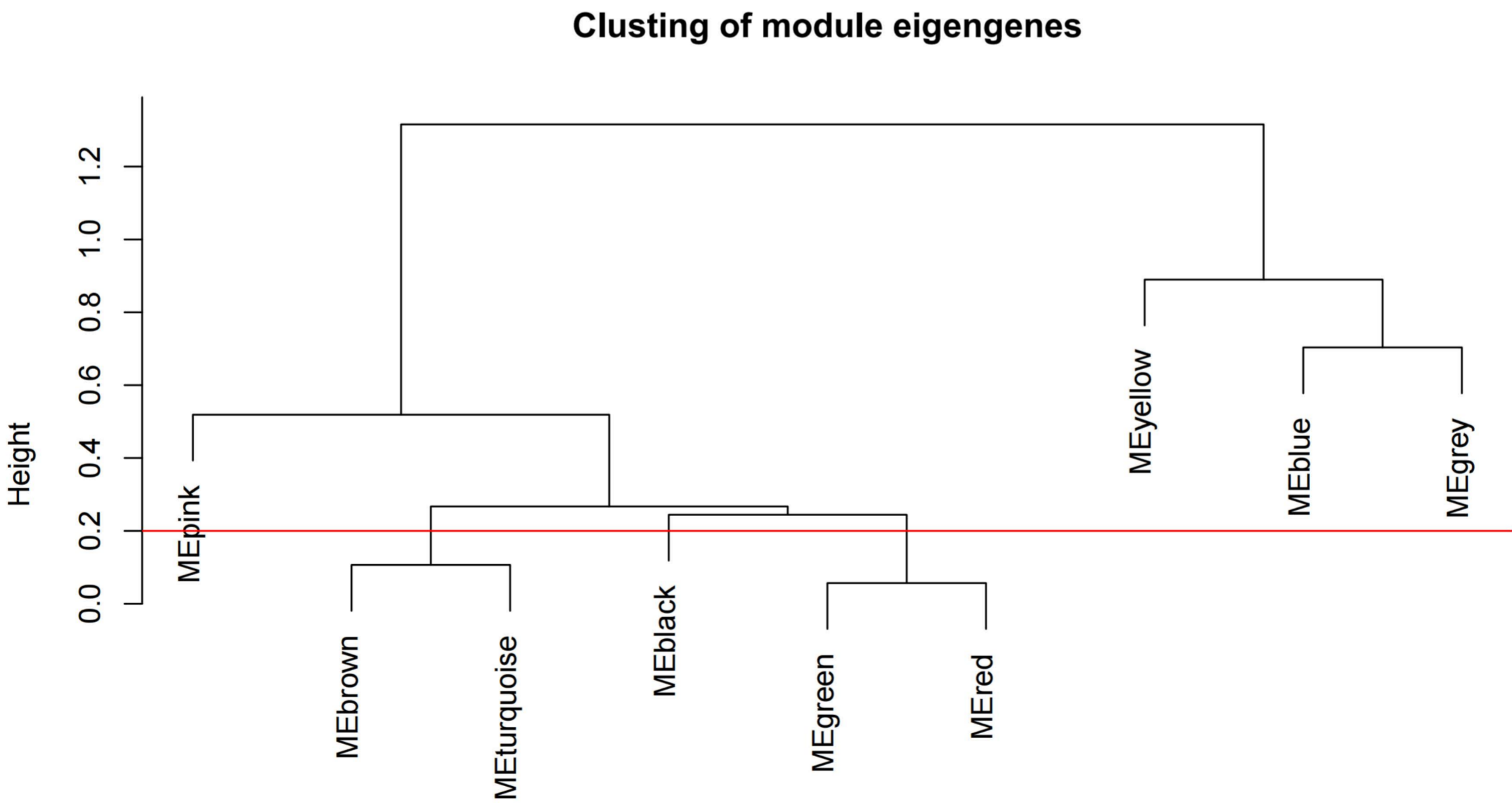

Supplement: Supplementary Materials — Supplementary Figure 1: WGCNA analysis of the TCGA dataset. Supplementary Figure 2: WGCNA analysis of the GSE133624 dataset. Supplementary Figures 3–7: clinical relevance of SMYD2, GAPDHP1, CILP, ATP1A2, and THSD4. Supplementary Table 1: primer sequences in the study. Supplementary Table 2: DEGs in the TCGA dataset. Supplementary Table 3: DEGs in the GSE133624 dataset. Supplementary Table 4: DEGs coexisting in the TCGA and GSE133624 datasets. Supplementary Table 5: feature genes were selected with the SVM-RFE algorithm. Supplementary Table 6: the correlation between the characteristic genes and immune cells. Supplementary Table 7: single-gene GSEA for prognostic genes. [file 1802706.f1.zip › 1802706.f1/Supplementary Figure 1.pdf]

A

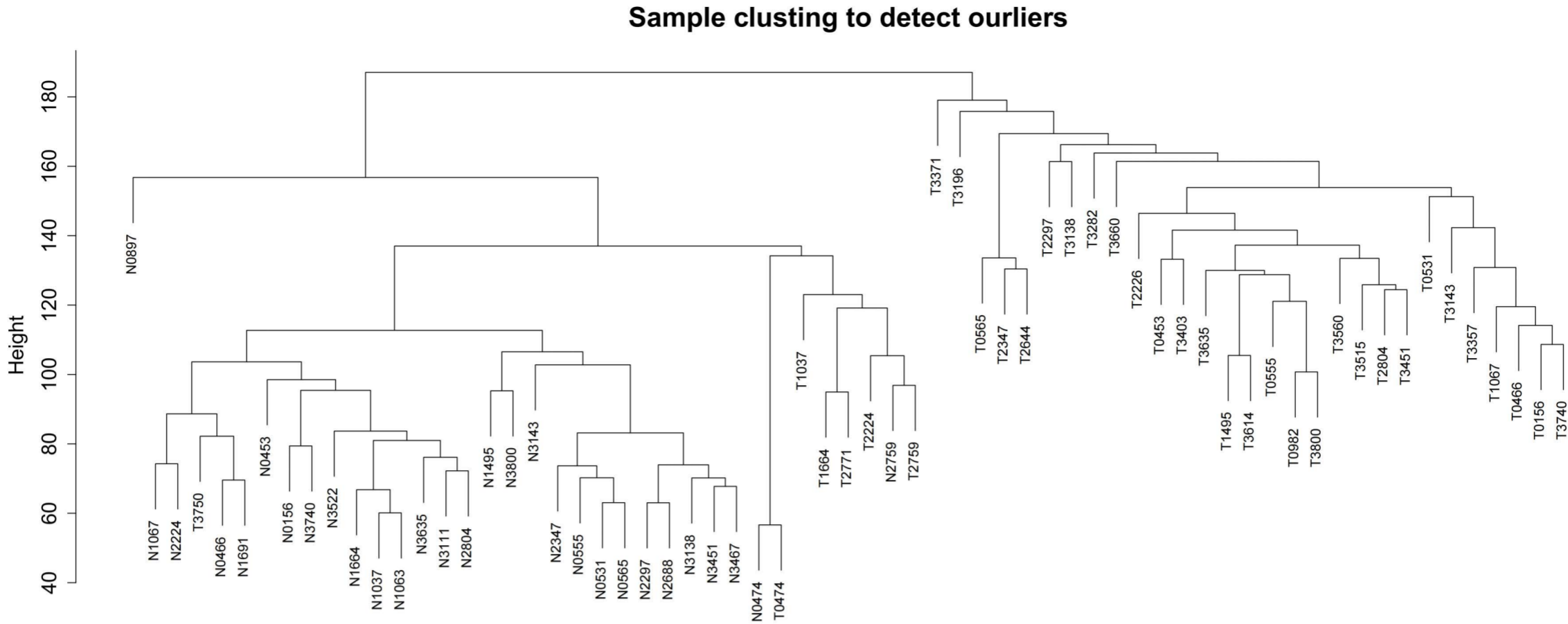

B

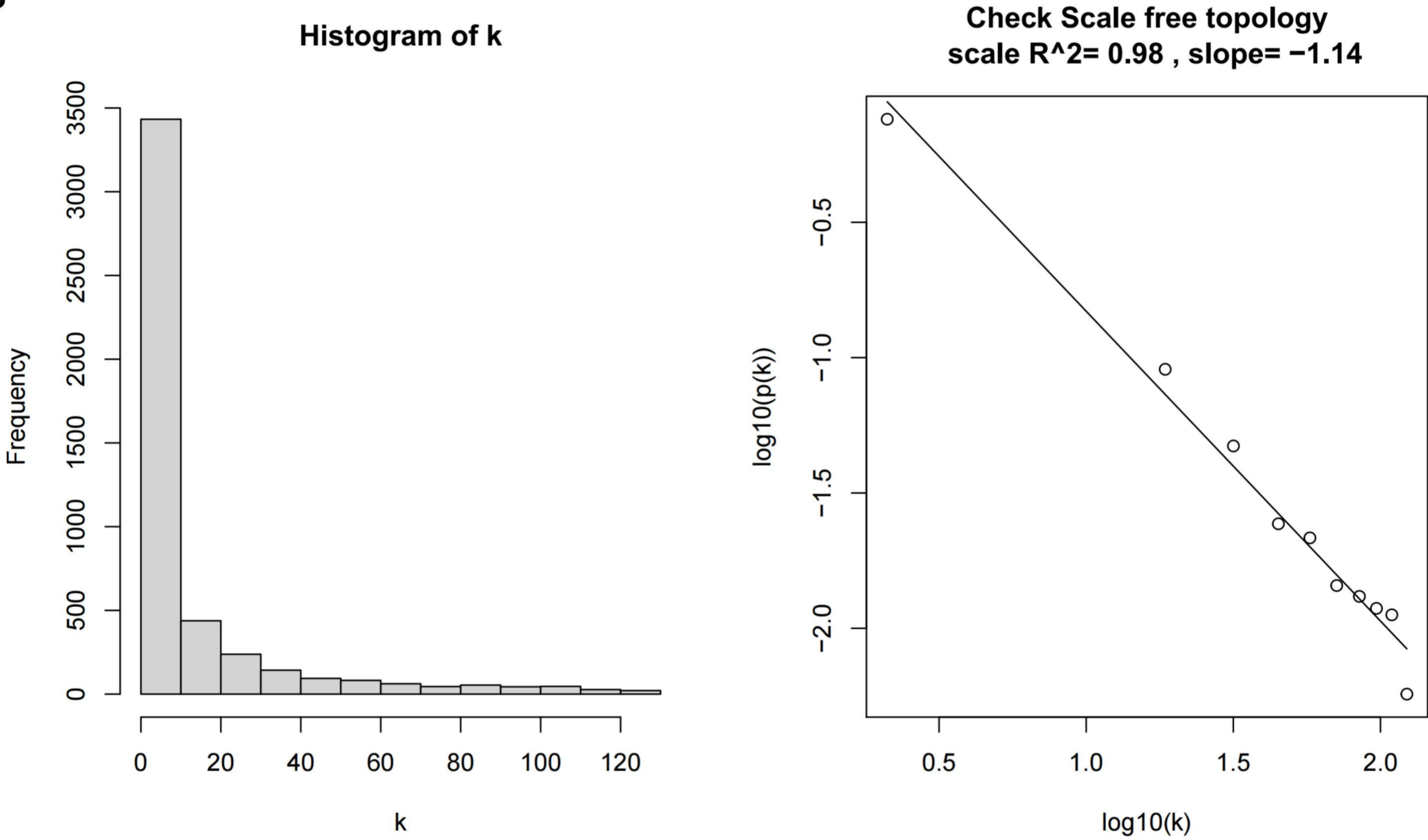

C

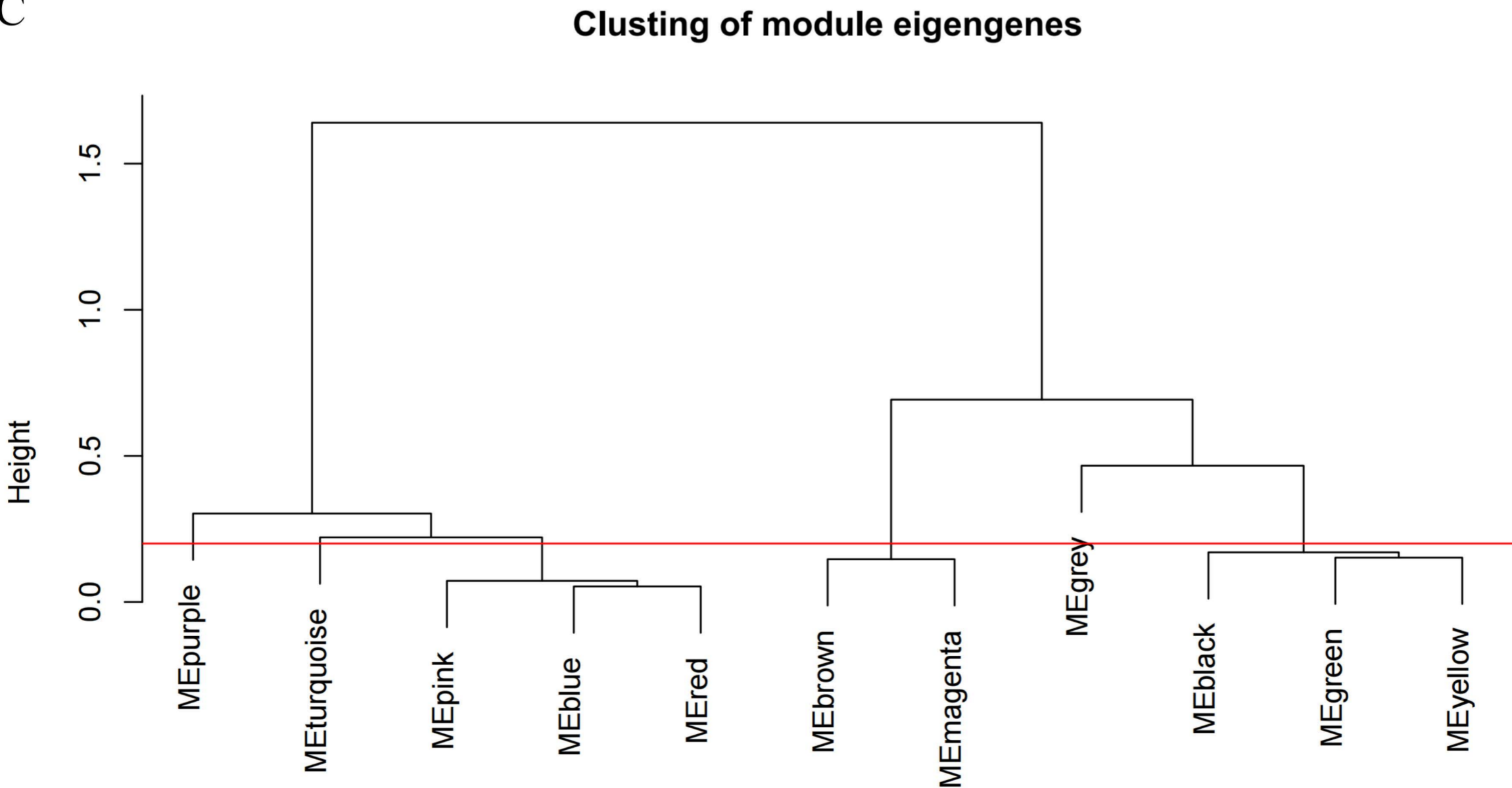

Supplement: Supplementary Materials — Supplementary Figure 1: WGCNA analysis of the TCGA dataset. Supplementary Figure 2: WGCNA analysis of the GSE133624 dataset. Supplementary Figures 3–7: clinical relevance of SMYD2, GAPDHP1, CILP, ATP1A2, and THSD4. Supplementary Table 1: primer sequences in the study. Supplementary Table 2: DEGs in the TCGA dataset. Supplementary Table 3: DEGs in the GSE133624 dataset. Supplementary Table 4: DEGs coexisting in the TCGA and GSE133624 datasets. Supplementary Table 5: feature genes were selected with the SVM-RFE algorithm. Supplementary Table 6: the correlation between the characteristic genes and immune cells. Supplementary Table 7: single-gene GSEA for prognostic genes. [file 1802706.f1.zip › 1802706.f1/Supplementary Figure 2.pdf]

A

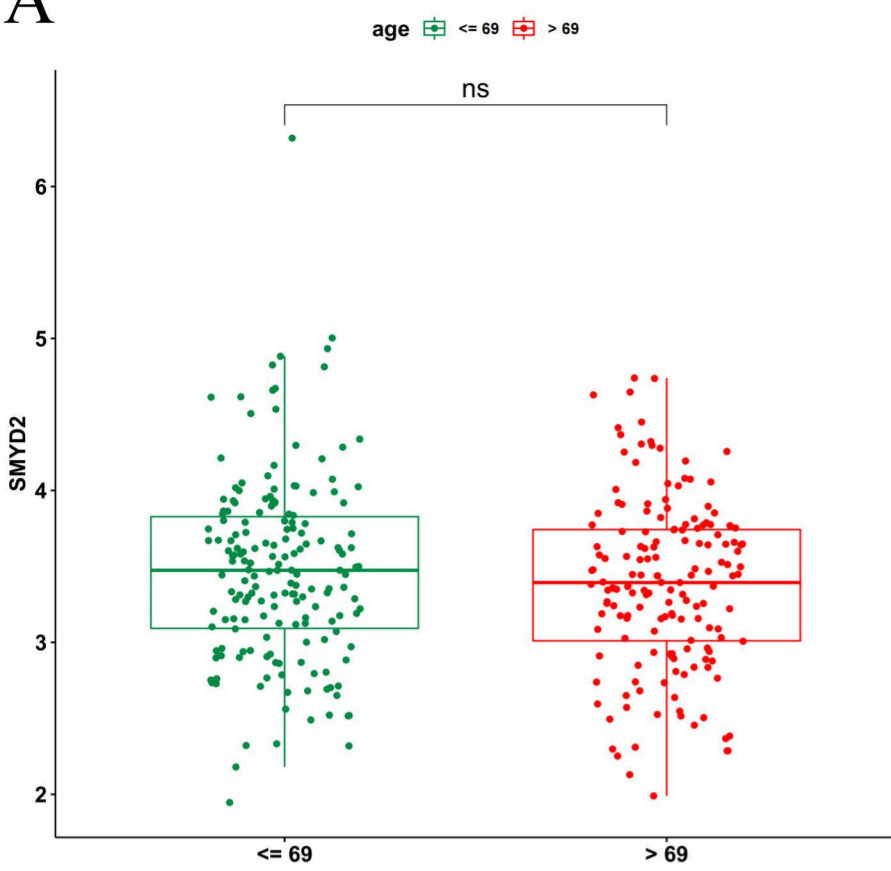

B

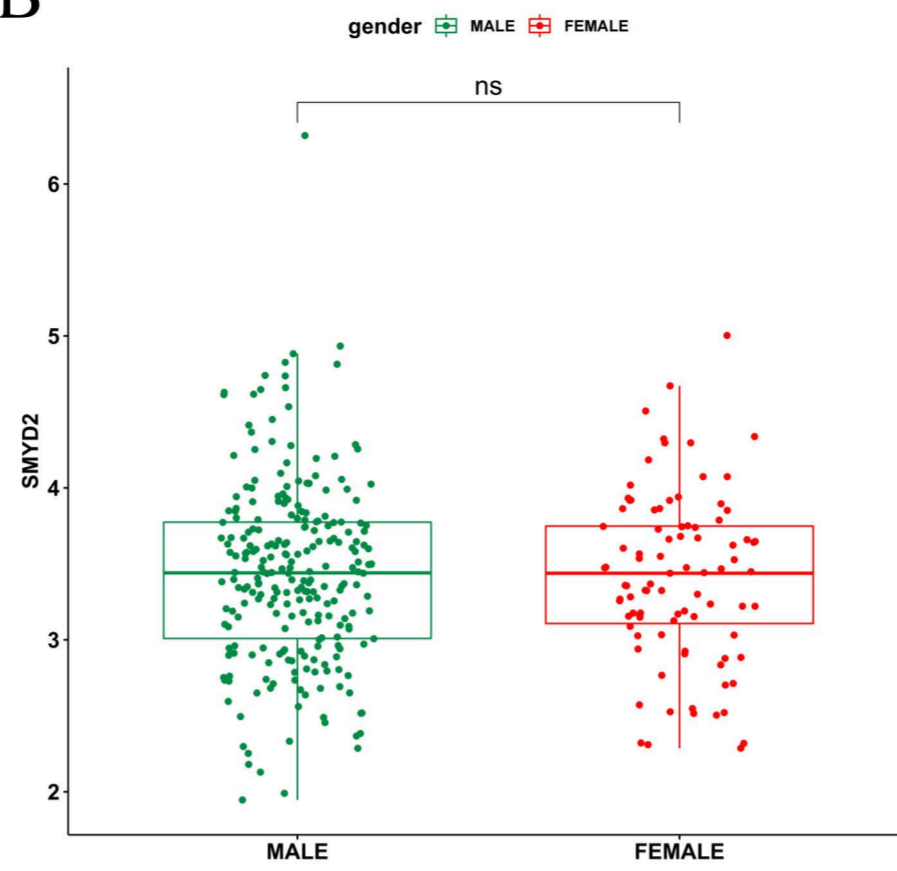

C

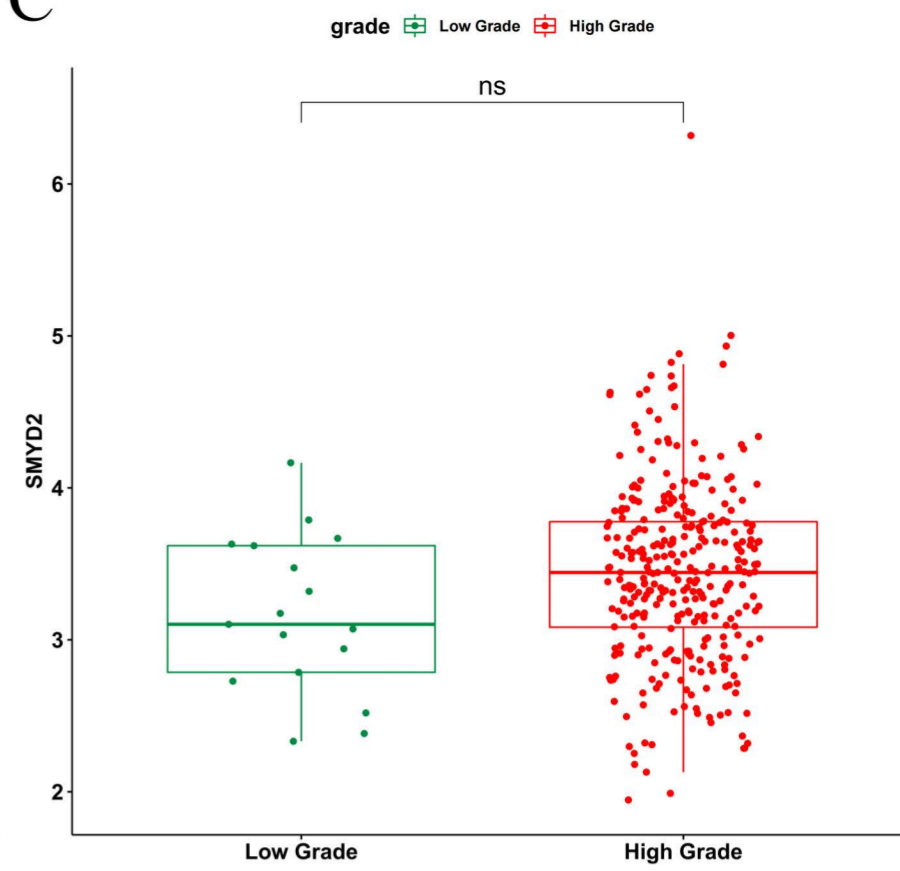

D

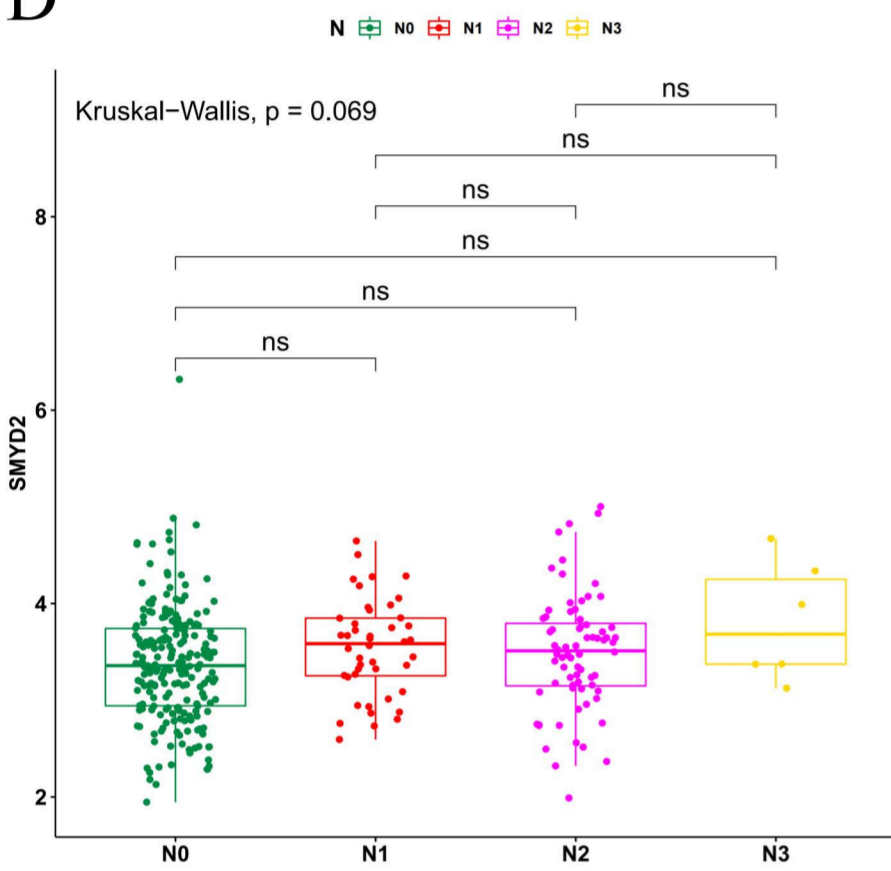

E

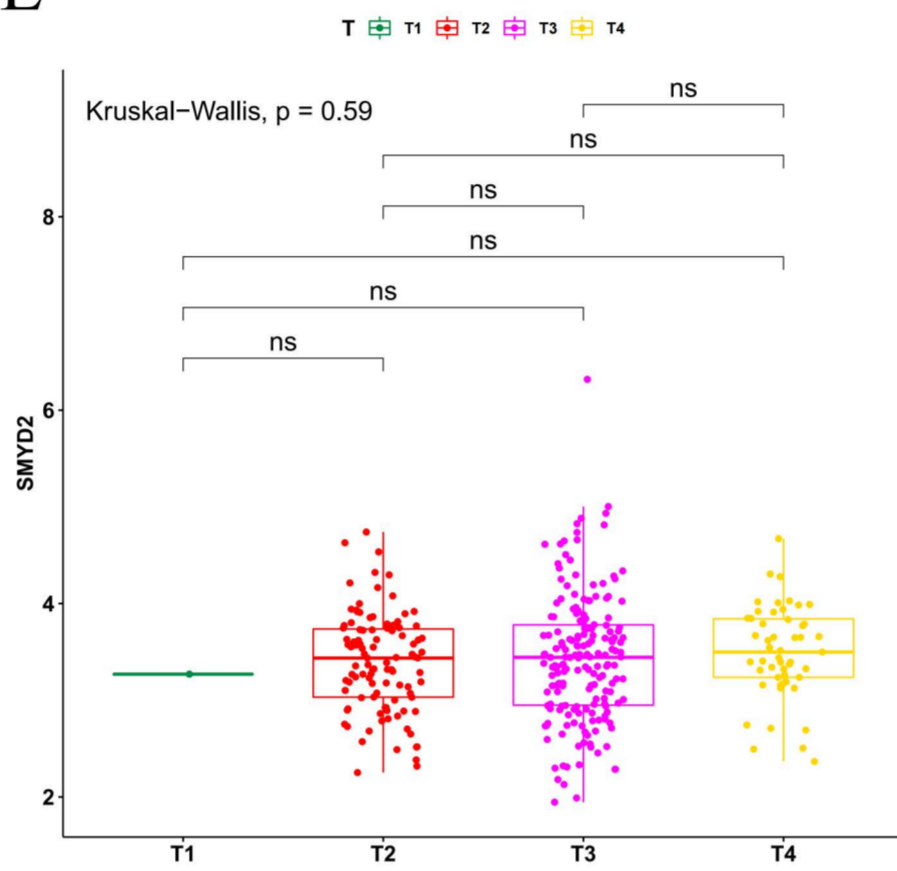

Supplement: Supplementary Materials — Supplementary Figure 1: WGCNA analysis of the TCGA dataset. Supplementary Figure 2: WGCNA analysis of the GSE133624 dataset. Supplementary Figures 3–7: clinical relevance of SMYD2, GAPDHP1, CILP, ATP1A2, and THSD4. Supplementary Table 1: primer sequences in the study. Supplementary Table 2: DEGs in the TCGA dataset. Supplementary Table 3: DEGs in the GSE133624 dataset. Supplementary Table 4: DEGs coexisting in the TCGA and GSE133624 datasets. Supplementary Table 5: feature genes were selected with the SVM-RFE algorithm. Supplementary Table 6: the correlation between the characteristic genes and immune cells. Supplementary Table 7: single-gene GSEA for prognostic genes. [file 1802706.f1.zip › 1802706.f1/Supplementary Figure 3.pdf]

A

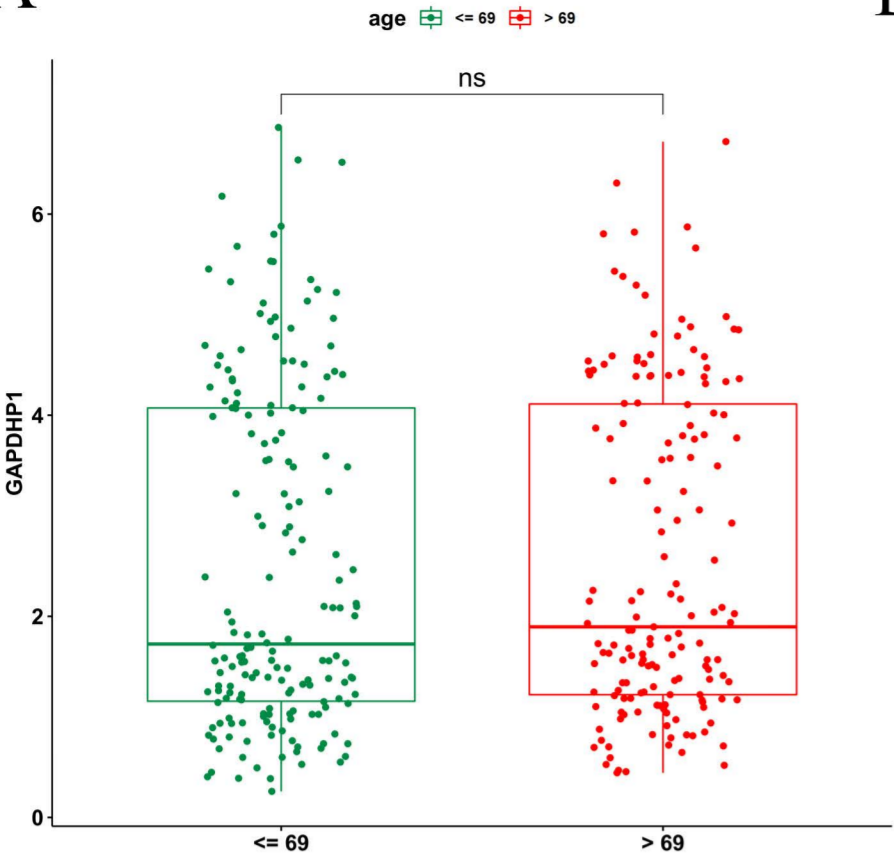

B

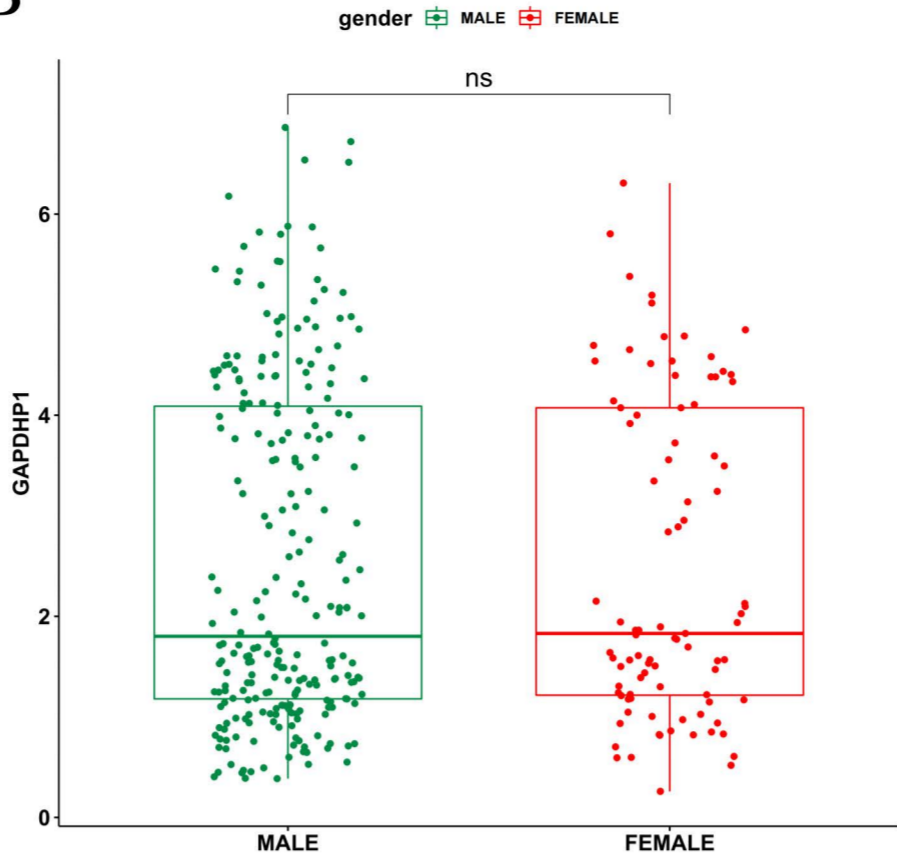

C

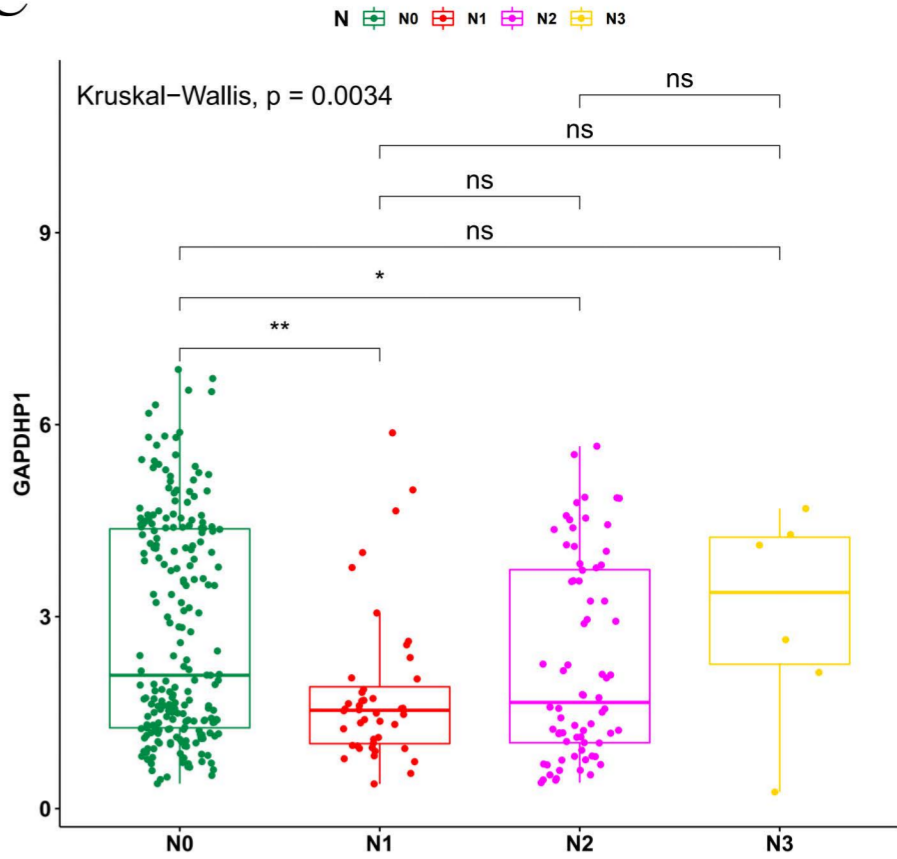

Supplement: Supplementary Materials — Supplementary Figure 1: WGCNA analysis of the TCGA dataset. Supplementary Figure 2: WGCNA analysis of the GSE133624 dataset. Supplementary Figures 3–7: clinical relevance of SMYD2, GAPDHP1, CILP, ATP1A2, and THSD4. Supplementary Table 1: primer sequences in the study. Supplementary Table 2: DEGs in the TCGA dataset. Supplementary Table 3: DEGs in the GSE133624 dataset. Supplementary Table 4: DEGs coexisting in the TCGA and GSE133624 datasets. Supplementary Table 5: feature genes were selected with the SVM-RFE algorithm. Supplementary Table 6: the correlation between the characteristic genes and immune cells. Supplementary Table 7: single-gene GSEA for prognostic genes. [file 1802706.f1.zip › 1802706.f1/Supplementary Figure 4.pdf]

A

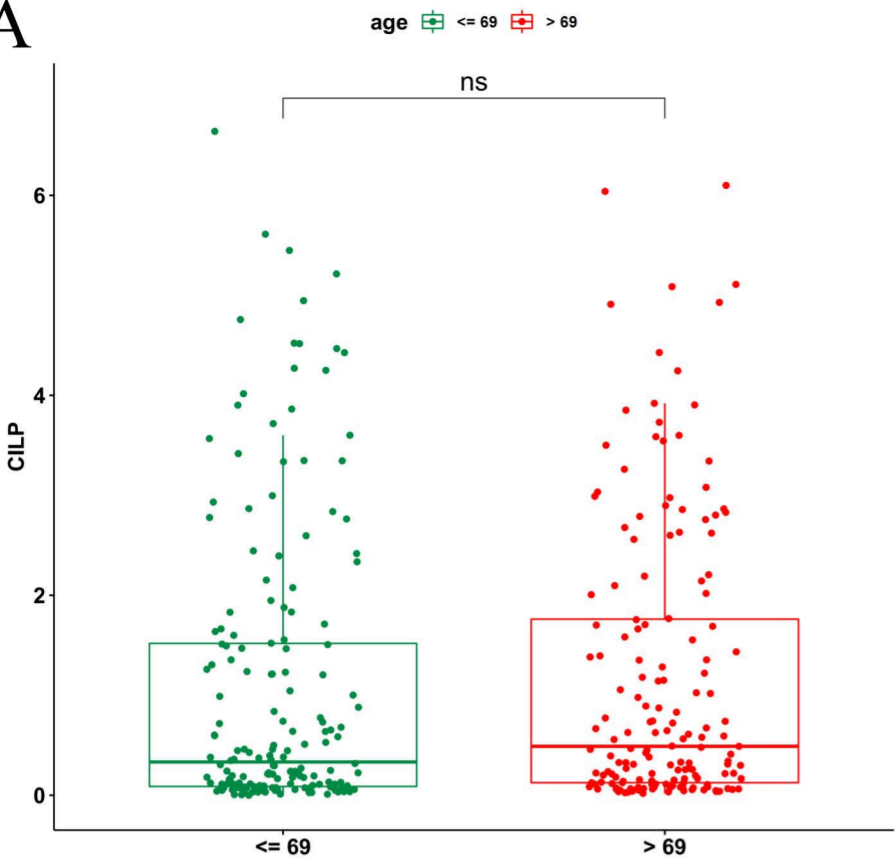

B

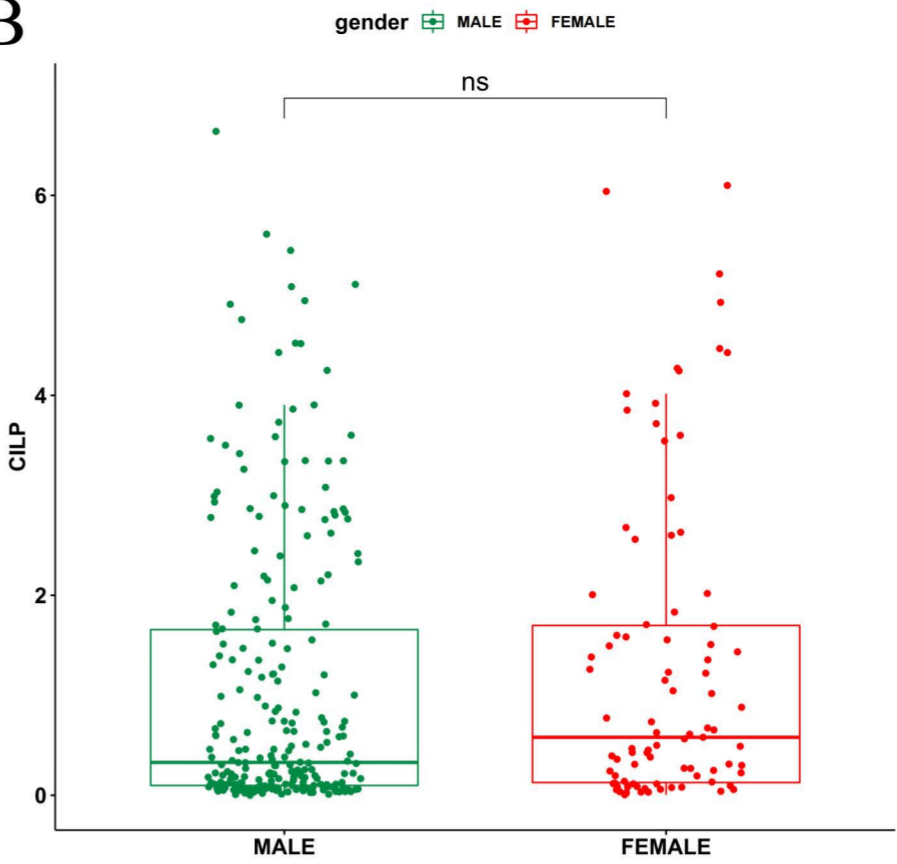

Supplement: Supplementary Materials — Supplementary Figure 1: WGCNA analysis of the TCGA dataset. Supplementary Figure 2: WGCNA analysis of the GSE133624 dataset. Supplementary Figures 3–7: clinical relevance of SMYD2, GAPDHP1, CILP, ATP1A2, and THSD4. Supplementary Table 1: primer sequences in the study. Supplementary Table 2: DEGs in the TCGA dataset. Supplementary Table 3: DEGs in the GSE133624 dataset. Supplementary Table 4: DEGs coexisting in the TCGA and GSE133624 datasets. Supplementary Table 5: feature genes were selected with the SVM-RFE algorithm. Supplementary Table 6: the correlation between the characteristic genes and immune cells. Supplementary Table 7: single-gene GSEA for prognostic genes. [file 1802706.f1.zip › 1802706.f1/Supplementary Figure 5.pdf]

A

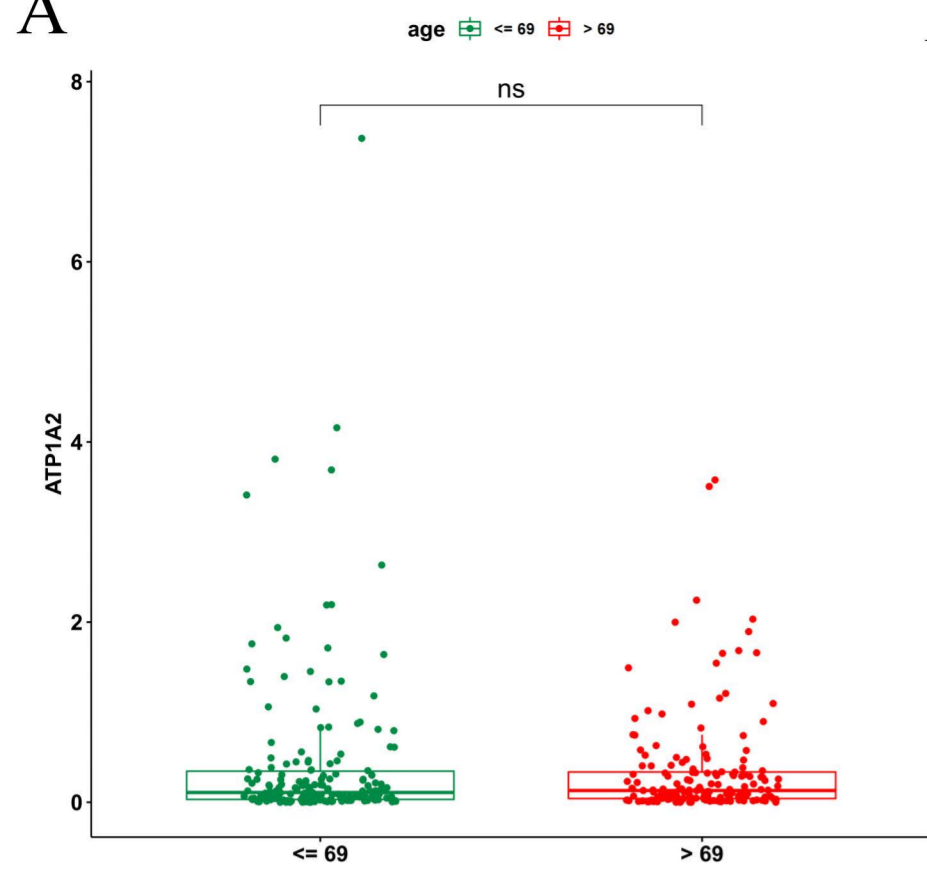

B

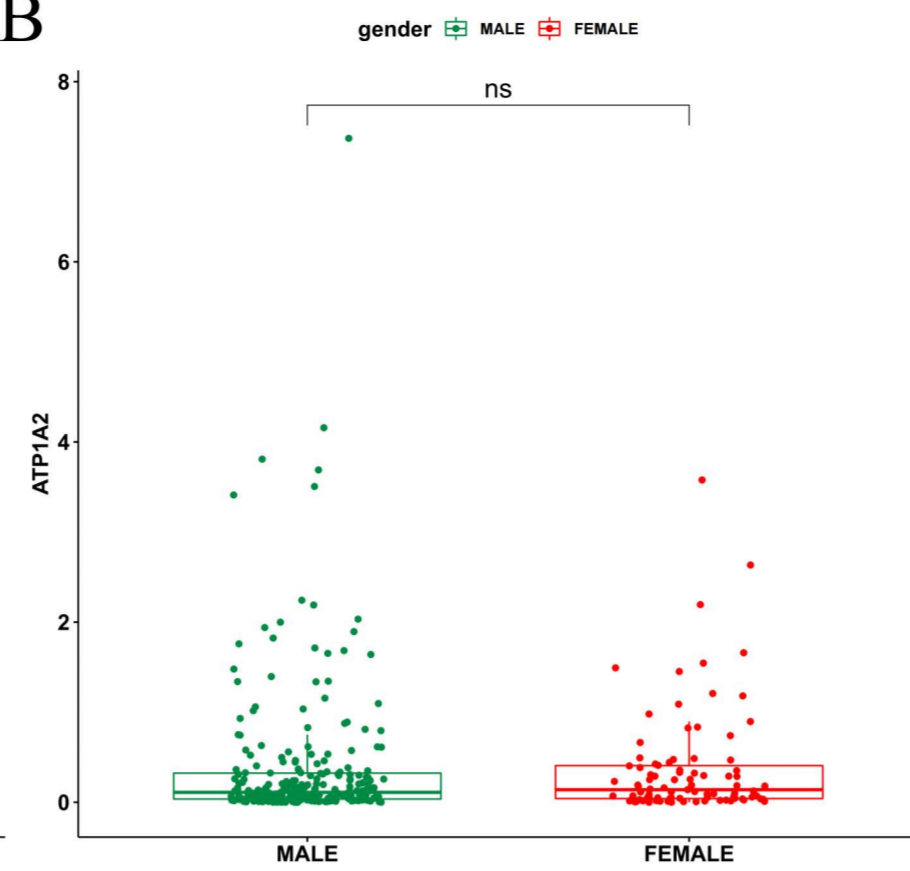

C

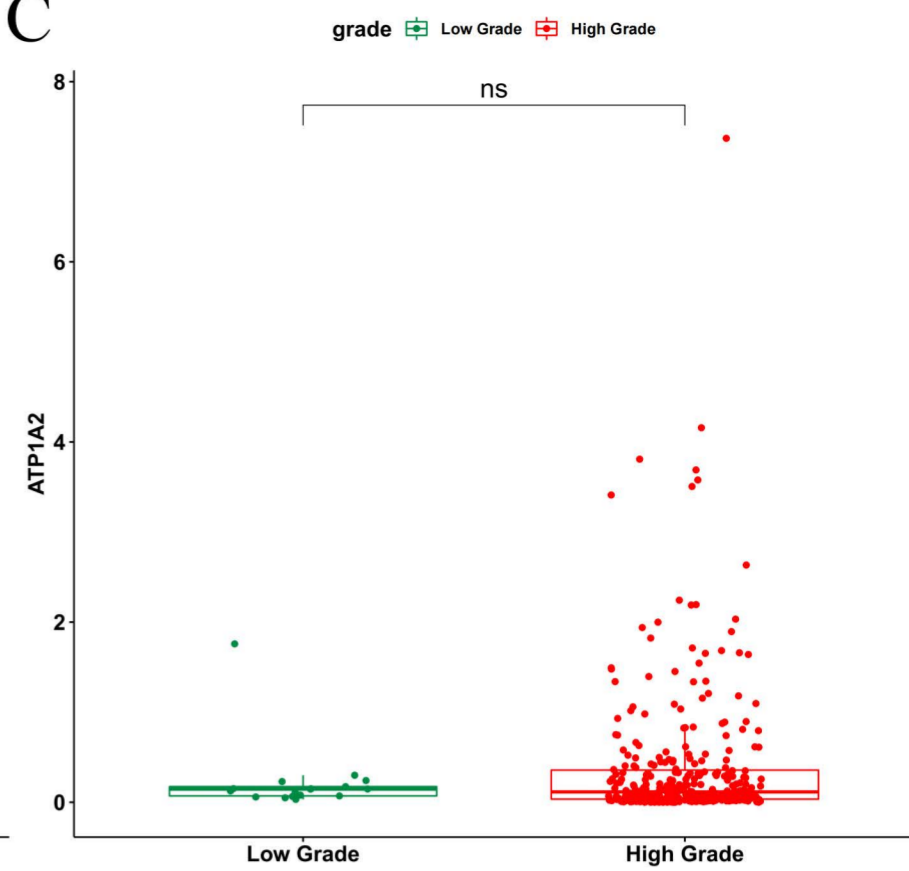

D

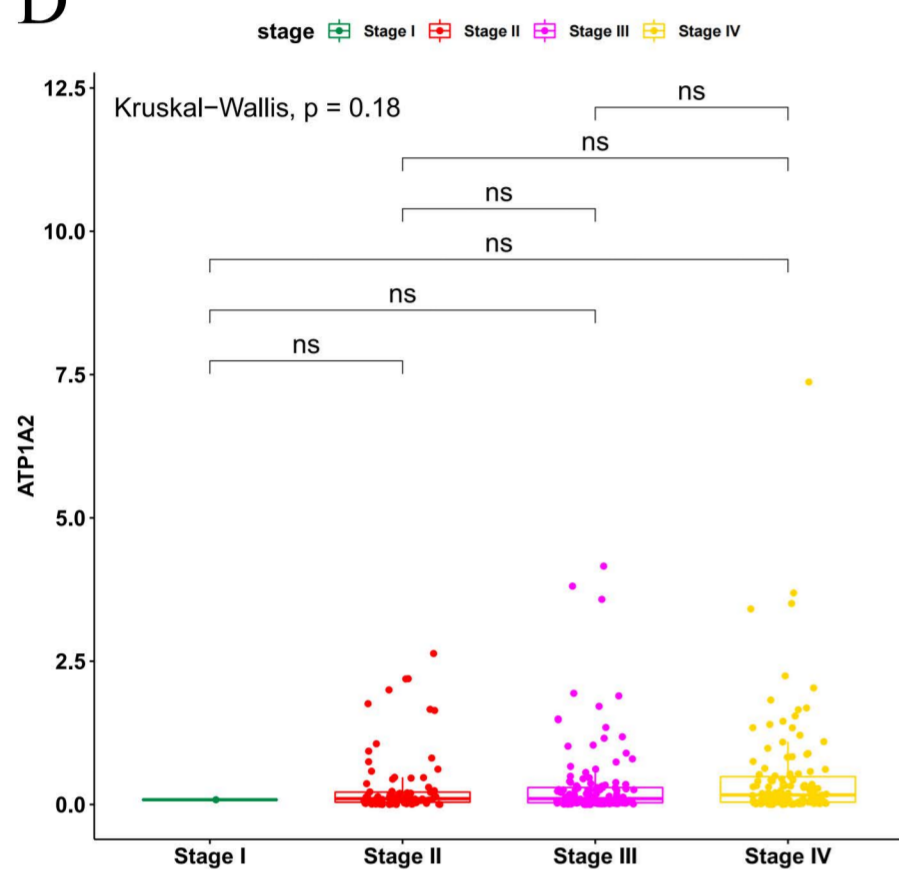

E

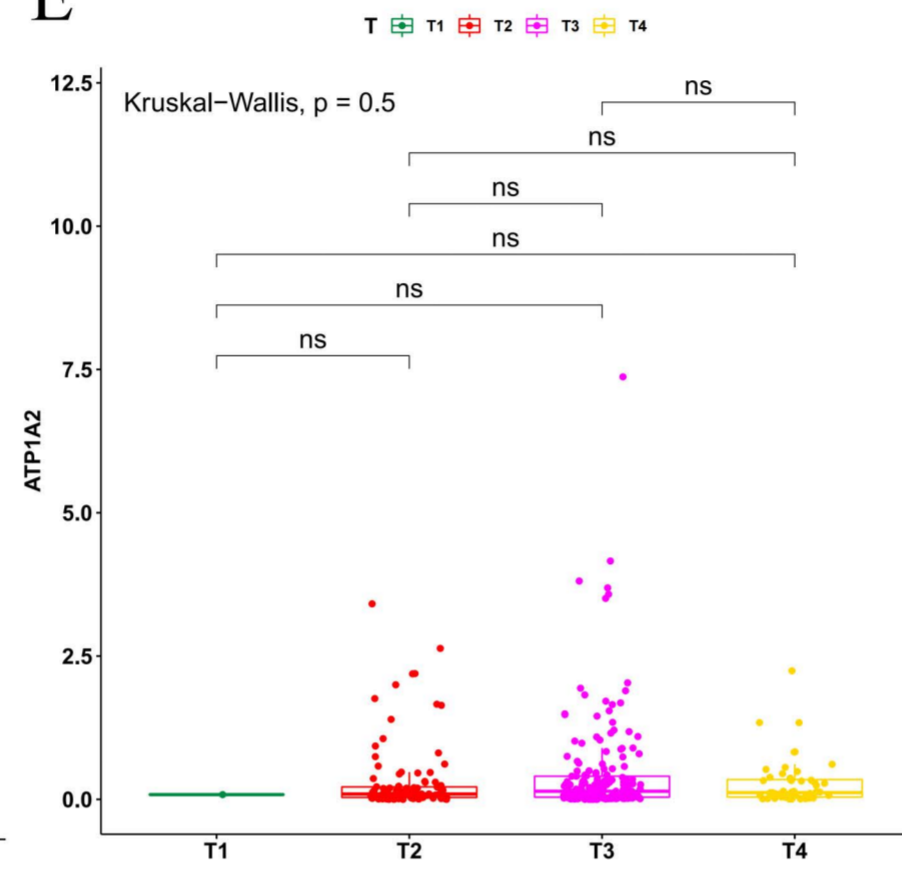

Supplement: Supplementary Materials — Supplementary Figure 1: WGCNA analysis of the TCGA dataset. Supplementary Figure 2: WGCNA analysis of the GSE133624 dataset. Supplementary Figures 3–7: clinical relevance of SMYD2, GAPDHP1, CILP, ATP1A2, and THSD4. Supplementary Table 1: primer sequences in the study. Supplementary Table 2: DEGs in the TCGA dataset. Supplementary Table 3: DEGs in the GSE133624 dataset. Supplementary Table 4: DEGs coexisting in the TCGA and GSE133624 datasets. Supplementary Table 5: feature genes were selected with the SVM-RFE algorithm. Supplementary Table 6: the correlation between the characteristic genes and immune cells. Supplementary Table 7: single-gene GSEA for prognostic genes. [file 1802706.f1.zip › 1802706.f1/Supplementary Figure 6.pdf]

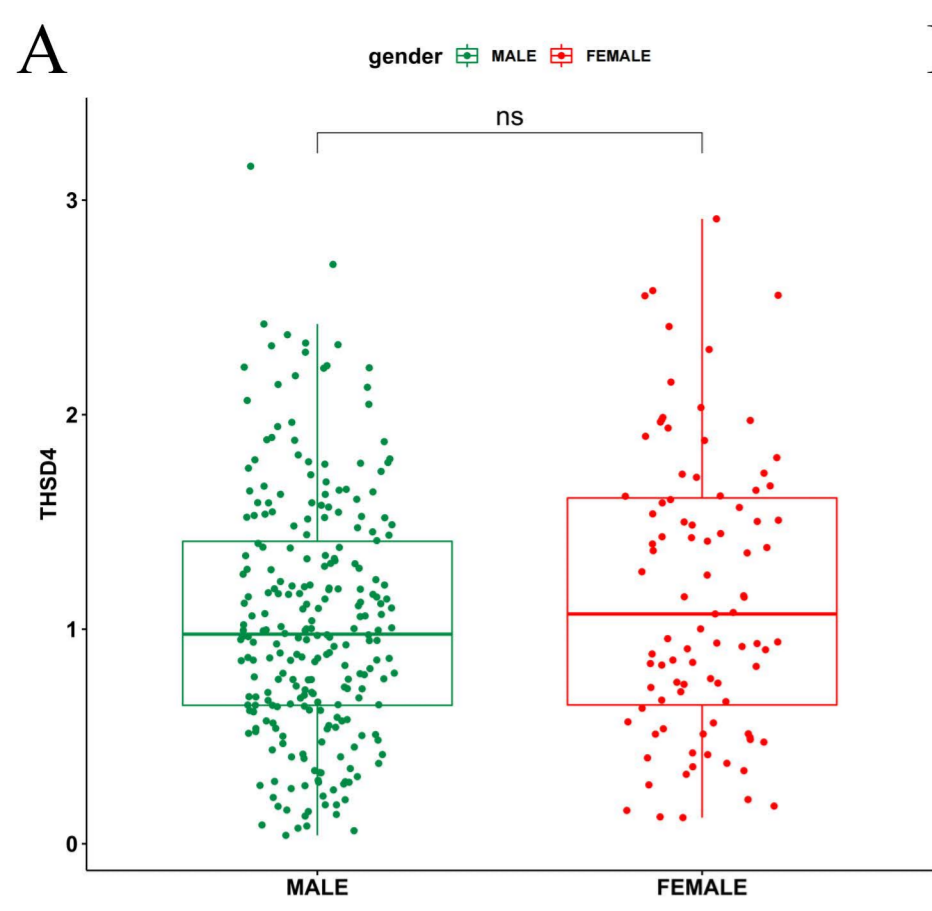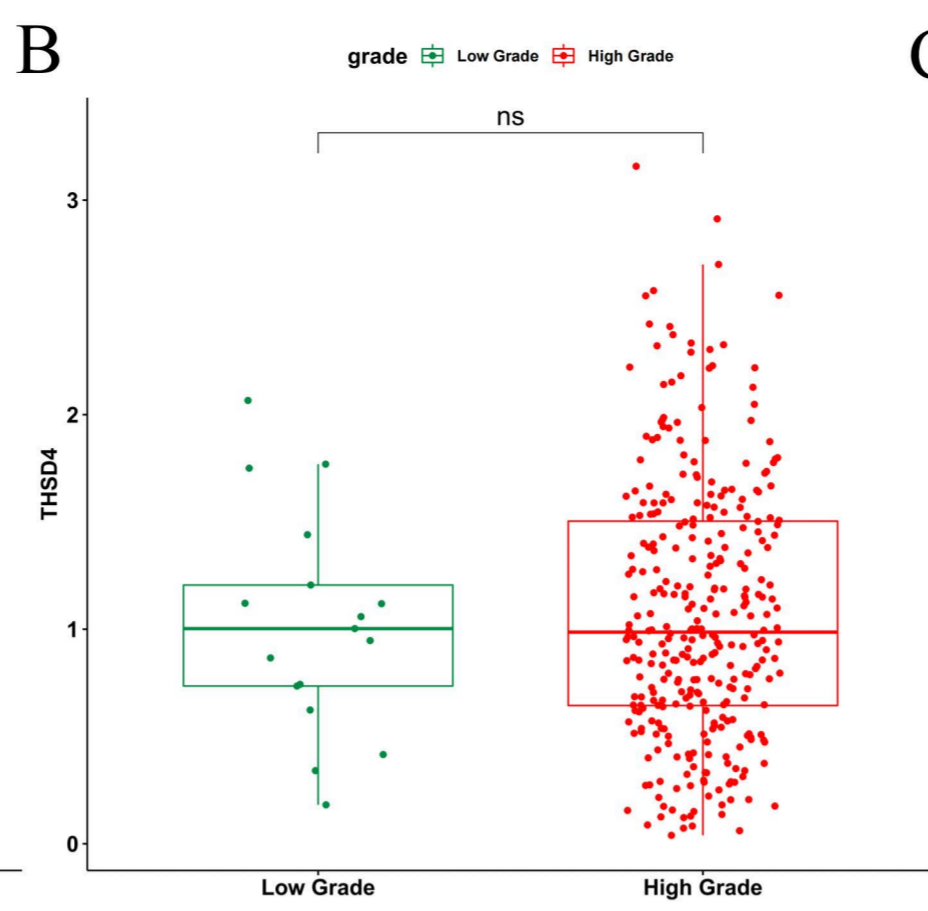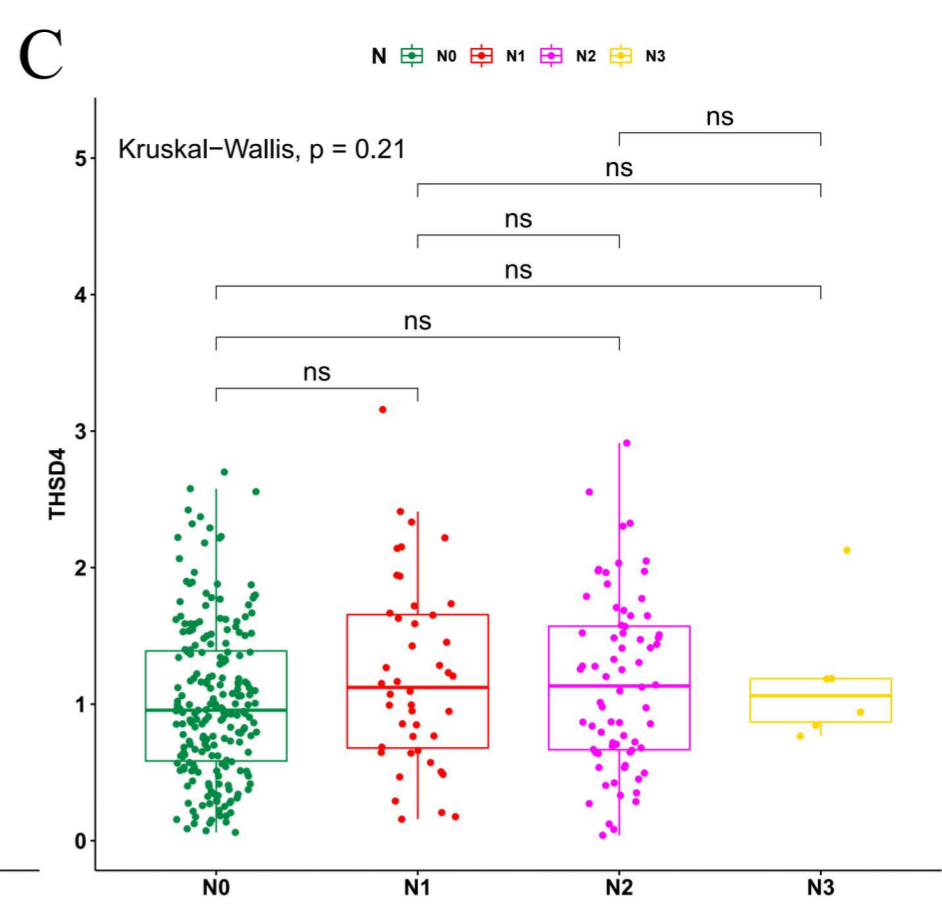

Supplement: Supplementary Materials — Supplementary Figure 1: WGCNA analysis of the TCGA dataset. Supplementary Figure 2: WGCNA analysis of the GSE133624 dataset. Supplementary Figures 3–7: clinical relevance of SMYD2, GAPDHP1, CILP, ATP1A2, and THSD4. Supplementary Table 1: primer sequences in the study. Supplementary Table 2: DEGs in the TCGA dataset. Supplementary Table 3: DEGs in the GSE133624 dataset. Supplementary Table 4: DEGs coexisting in the TCGA and GSE133624 datasets. Supplementary Table 5: feature genes were selected with the SVM-RFE algorithm. Supplementary Table 6: the correlation between the characteristic genes and immune cells. Supplementary Table 7: single-gene GSEA for prognostic genes. [file 1802706.f1.zip › 1802706.f1/Supplementary Figure 7.pdf]
